# Effects of updated demography, disability weights, and cervical cancer burden on estimates of human papillomavirus vaccination impact at the global, regional, and national levels: a PRIME modelling study

**DOI:** 10.1016/S2214-109X(20)30022-X

**Published:** 2020-02-24

**Authors:** Kaja M Abbas, Kevin van Zandvoort, Marc Brisson, Mark Jit

**Affiliations:** a Department of Infectious Disease Epidemiology, London School of Hygiene & Tropical Medicine, London, UK; b Centre de recherche du CHU de Québec and Department of Social and Preventive Medicine, Université Laval, Quebec, QC, Canada; c Modelling and Economics Unit, Public Health England, London, UK; d School of Public Health, University of Hong Kong, Hong Kong Special Administrative Region, China

## Abstract

**Background:**

The Papillomavirus Rapid Interface for Modelling and Economics (PRIME) has been used around the world to assess the health impact and cost-effectiveness of human papillomavirus (HPV) vaccination in girls. We updated PRIME with new data and methods for demography, disability weights, and cervical cancer burden, and generated revised estimates of the health impact of HPV vaccination at the global, regional, and national levels for 177 countries.

**Methods:**

PRIME was updated with population demography of the UN World Population Prospects (UNWPP) 2019 revision, disability weights of the Global Burden of Disease (GBD) 2017 study, and cervical cancer burden from the Global Cancer Incidence, Mortality and Prevalence (GLOBOCAN) 2018 database. We estimated the lifetime health benefits for bivalent or quadrivalent and nonavalent vaccination of 9-year-old and 12-year-old girls at 90% coverage during 2020–29 in 177 countries. Health impact was presented in terms of cervical cancer cases, deaths, or disability-adjusted life-years (DALYs) averted per 1000 vaccinated girls in comparison with the counterfactual scenario of no vaccination, and the number of girls needed to be vaccinated to prevent a single case, death, or DALY.

**Findings:**

In estimating the health impact of HPV vaccination of 9-year-old girls, the combined updates to demography, disability weights, cervical cancer burden estimates resulted in a 26% increase in the estimated number of cases averted, a 51% increase in deaths averted, and a 72% increase in DALYs averted per 1000 vaccinated girls for both the bivalent or quadrivalent and nonavalent vaccines, compared with previous estimates. With the updated model, the bivalent or quadrivalent HPV vaccine was estimated to avert 15 cases, 12 deaths, and 243 DALYs per 1000 vaccinated girls, and the nonavalent HPV vaccine was estimated to avert 19 cases, 14 deaths, and 306 DALYs per 1000 vaccinated girls. The health benefits of vaccination of 12-year-old girls were estimated to be similar but slightly decreased in comparison with vaccination of 9-year-old girls.

**Interpretation:**

HPV vaccination provides greater health benefits and is more cost-effective than was previously estimated. The demography update, which incorporates population aging, has the largest effect on the health impact estimates. The WHO African region is expected to gain the greatest health benefits and should be prioritised for HPV vaccination.

**Funding:**

Gavi, the Vaccine Alliance; Bill & Melinda Gates Foundation.

## Introduction

Cervical cancer has the fourth greatest global burden of cancer among women for both incidence and mortality, and is the leading cause of cancer death among women in 42 countries.^1^ WHO estimated that 570 000 new cases occurred and 311 000 women died from cervical cancer globally in 2018, with nearly 90% of these deaths occurring in low-income and middle-income countries.^2^, ^3^

Cervical cancer is caused by infection with high-risk genotypes of human papillomavirus (HPV). Three HPV vaccines (bivalent, quadrivalent, and nonavalent) are widely available in the global market, and are highly efficacious in preventing persistent infection and associated disease from the high-risk HPV 16/18 genotypes, which cause 70% of all cervical cancers.^4^, ^5^, ^6^, ^7^ In addition to HPV 16/18, the nonavalent vaccine also protects against high-risk HPV types 31/33/45/52/58 which cause 18·5% of HPV-positive cervical cancers.^8^

WHO recommends that all countries introduce HPV vaccination for primary prevention of cervical cancer, prioritising the primary target group of young adolescent girls, aged 9–14 years.^9^ Secondary prevention includes screening and treatment of precancerous lesions, and tertiary prevention includes diagnosis and treatment of invasive cervical cancer and palliative care.

In May, 2018, the WHO Director-General called for global action to eliminate cervical cancer as a public health problem through improved coverage for HPV vaccination, high-precision screening tests, and treatment and care.^10^ The proposed 90–70–90 targets for 2030 comprise 90% coverage of HPV vaccination among girls by 15 years of age; 70% coverage of screening among women at 35 and 45 years of age and 90% treatment of precancer lesions; and 90% coverage of treatment and care among women diagnosed with cervical cancer. Achieving this aim will require up-to-date estimates of the impact and cost-effectiveness of HPV vaccination at the global, regional, and national levels.

**Research in context**

**Evidence before this study**

The Papillomavirus Rapid Interface for Modelling and Economics (PRIME) was developed in 2014 in collaboration with WHO to assess the health impact and cost-effectiveness of human papillomavirus (HPV) vaccination in girls around the world. It was used to show that vaccinating 12-year-old girls against HPV was cost-effective in almost every country for the bivalent and quadrivalent vaccines. Since its development and the introduction of a nonavalent vaccine in 2014, new data and methods for demography from the UN World Population Prospects 2019 revision, disability weights from the Global Burden of Disease 2017 study, and cervical cancer burden from the Global Cancer Incidence, Mortality and Prevalence 2018 database have become available, and were used to update the model.

**Added value of this study**

The combined PRIME updates for demography, disability weights, and cervical cancer burden improve the HPV vaccination impact estimates in comparison with previous forecasts and suggest greater health benefits in preventing cases, deaths, and disability-adjusted life-years (DALYs) due to cervical cancer at the global, regional, and national levels. The demography update has the largest effect on the health impact estimates; population aging leads to increasing life expectancy among women and an increase in lifetime risk of cervical cancer incidence and mortality without vaccination. HPV vaccination can avert this potential increase in new cases and deaths, and because the number of girls needed to be vaccinated to prevent a single case, death, or DALY is lower than previous forecasts, HPV vaccination both provides greater health benefits and is more cost-effective than was previously estimated. Because of a higher burden of cervical cancer before vaccination in the WHO African region compared with other regions, HPV vaccination will provide the greatest health benefits in this region.

**Implications of all the available evidence**

Reaching the WHO goal of global elimination of cervical cancer as a public health problem will take decades. Therefore, HPV vaccine impact models, such as PRIME, need to regularly integrate new knowledge and updated data sources. With such updates, HPV vaccination remains highly cost-effective globally, and efforts to increase coverage and equity should be continued. Countries in the WHO African region should be prioritised for HPV vaccine introduction and scale-up.

The Papillomavirus Rapid Interface for Modelling and Economics (PRIME) is a model to assess the health impact and cost-effectiveness of HPV vaccination of girls for prevention of cervical cancer.^11^, ^12^ It was developed in 2014 in collaboration with WHO and has been validated against 17 published studies set in low-income and middle-income countries. It is endorsed by the WHO Immunization and Vaccines Implementation Research Advisory Committee to provide a conservative estimate of the health impact and cost-effectiveness of vaccinating girls before sexual debut.^11^, ^13^ PRIME has been used to inform the impact of vaccine investments by Gavi, the Vaccine Alliance, in 97 countries, as well as at the national level.^11^, ^12^, ^14^

Since PRIME was developed, the nonavalent HPV vaccine was licensed in 2014, and new data and methods for demography, disability weights, and cervical cancer burden have become available. We have updated vaccine impact estimates generated by PRIME to reflect almost a decade of new data and assessed the effects of these updates on estimates at the global, regional, and national levels.

## Methods

### Data inputs and modelling process in PRIME

PRIME is a static proportional impact model that estimates the impact of both single-age and multiple-age cohort vaccination. Vaccination impact is estimated in terms of reduction in age-dependent cervical cancer incidence, prevalence, and mortality in direct proportion to vaccine coverage, vaccine efficacy, and distribution of high-risk HPV types (HPV 16/18 for the bivalent and quadrivalent vaccines and HPV 16/18/31/33/45/52/58 for the nonavalent vaccine).^5^ Herd effects and cross-protection are not considered; therefore, the estimated health benefits of HPV vaccination of 9–14-year-old girls are conservative. Vaccinating girls before sexual debut fully protects them from developing cervical cancer caused by high-risk HPV types, in accordance with the efficacy observed in vaccine trials.^15^ Waning of vaccine-induced immunity against high-risk HPV types has not been empirically observed in follow-up studies since HPV vaccine introduction. The model assumes a two-dose schedule with perfect timeliness to the target ages given in the coverage estimates. Model equations and parameters have been extensively described elsewhere.^11^ Instructions for accessing the PRIME model and software are provided in appendix 1 (p 2).

PRIME data inputs include country and age-specific cervical cancer incidence, prevalence, and mortality among females from the Global Cancer Incidence, Mortality and Prevalence (GLOBOCAN) database.^16^ We updated PRIME with new data and methods for demography, disability weights, and cervical cancer burden and assessed the impact of these updates on health impact estimates generated by the model for the bivalent, quadrivalent, and nonavalent vaccines. All data were from secondary sources in the public domain, and therefore ethics approval was not required.

PRIME uses country-specific life tables to model the annual population size of longitudinal birth cohorts. The life tables were previously generated from population size of birth cohorts and all-cause female mortality estimates by age from the WHO 2009 Global Health Observatory, which remained static over time.^17^ We updated the life tables to use population size and all-cause female mortality estimates from the UN World Population Prospects (UNWPP) 2019 revision, which varies dynamically over time during 1950–2100.^18^

Disability weights were updated from estimates of the Global Burden of Disease (GBD) 2001 study^19^ to estimates of the GBD 2017 study.^20^, ^21^ Disability weights and GBD-prescribed durations for the different phases of cervical cancer (ie, diagnosis and primary treatment phase, controlled phase, metastatic phase, and terminal phase) in the GBD 2001 and GBD 2017 studies are presented in appendix 1 (pp 3, 4).^22^ In the GBD 2001 study, the morbidity impact of years lived with disability (YLD) was attributed to the specific year of incidence of new cases. In the GBD 2017 study, the morbidity impact was attributed to the specific years of prevalence of women experiencing the different phases of cervical cancer.

Cervical cancer burden was updated from the International Agency for Research on Cancer estimates for GLOBOCAN 2012^23^ to GLOBOCAN 2018.^1^ The GLOBOCAN 2012 and 2018 databases provide country-specific cancer burden data by 5-year age groups and sex for 28 types of cancer in 184 countries (GLOBOCAN 2012) and 36 types of cancer in 185 countries (GLOBOCAN 2018).

HPV vaccine impact on cervical cancer burden (incidence, prevalence, and mortality) averted is calculated as the proportional reduction in age-specific incidence, prevalence, and mortality in each country at single-year age intervals from 0–100 years. Cervical cancer burden was calculated as follows: let *v* be the age at vaccination and *i* any given age, where *v* is less than or equal to *i*; then cervical cancer burden averted at age *i* is the product of five estimates: cervical cancer burden caused by all HPV genotypes at age *i* before vaccination, country-specific proportion of cervical cancer caused by high-risk HPV types, two-dose vaccine coverage at age *v*, vaccine efficacy against high-risk HPV types, and proportion of female population that has not experienced sexual debut by age *v.*

### Comparative scenarios

We analysed five scenarios to assess the health impact of HPV vaccination using PRIME (appendix 1 p 5). The base scenario (S1) is based on demography from WHO 2009 life tables, disability weights from the GBD 2001 study, and cervical cancer burden estimates from the GLOBOCAN 2012 database. In comparison with S1, scenario 2 (S2) incorporates demography from UNWPP 2019 revision, scenario 3 (S3) incorporates disability weights from the GBD 2017 study, and scenario 4 (S4) incorporates cervical cancer burden estimates from the GLOBOCAN 2018 database. Scenario 5 (S5) shows the combined effect of using all three of the updated demography, disability weight, and cervical cancer burden estimates. We estimated the lifetime impact of routine vaccination of girls at 90% coverage (as recommended in the WHO cervical cancer elimination strategy) for the 10-year period of 2020–29 in comparison with the counterfactual scenario of no vaccination in 177 countries for bivalent or quadrivalent and nonavalent vaccination of 9-year-old and 12-year-old girls.

### Vaccine impact metrics

We present vaccine impact metrics in terms of cervical cancer burden averted per 1000 vaccinated girls and number of girls needed to be vaccinated to prevent a single cervical cancer case, death, YLD, year of life lost (YLL), or disability-adjusted life-year (DALY). These two vaccine impact metrics are not affected by vaccination coverage, with the limitation that PRIME includes only direct effects of HPV vaccination and excludes indirect herd effects.

### Role of the funding source

The funders of this study had no role in study design, data collection, data analysis, data interpretation, or writing of the manuscript. All authors had full access to data in the study, and final responsibility for the decision to submit for publication.

## Results

**Figure 1:**
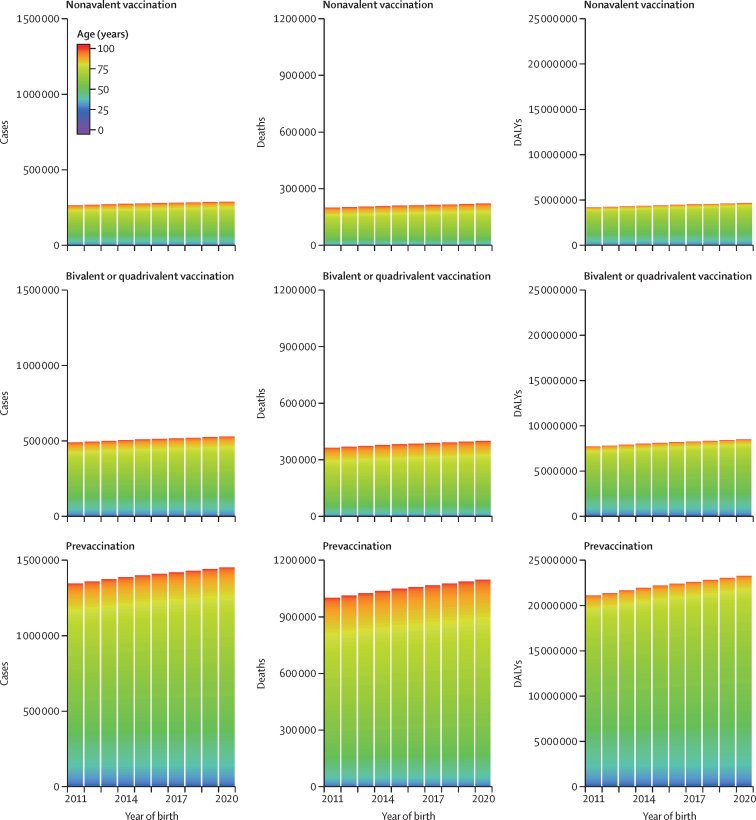
Lifetime cervical cancer burden before and after vaccination of 9-year-old girls at the global level Lifetime cervical cancer burden is shown in terms of cases, deaths, and DALYs, before and after bivalent or quadrivalent and nonavalent HPV vaccination of 9-year-old girls at 90% coverage in 177 countries during 2020–29, which relates to birth cohorts of 2011–2020 (estimates after the combined PRIME updates for demography, disability weights, and cervical cancer burden). DALY=disability-adjusted life-year. HPV=human papillomavirus. PRIME=Papillomavirus Rapid Interface for Modelling and Economics.

PRIME was updated with population demography from the UNWPP 2019 revision, disability weights from the GBD 2017 study, and cervical cancer burden from the GLOBOCAN 2018 database. Figure 1 presents the updated global estimates for lifetime burden of all cervical cancer before and after vaccination of 9-year-old girls with bivalent or quadrivalent and nonavalent vaccines at 90% coverage during 2020–29 in 177 countries. The equivalent estimates for 12-year-old girls are presented in appendix 1 (p 6). In addition, the estimates of lifetime burden of cervical cancer before and after vaccination at 90% coverage of 9-year-old and 12-year-old girls by HPV genotype (HPV 16/18 and HPV 16/18/31/33/45/52/58) are presented at the global, regional, and national levels in the appendices (global, appendix 1 pp 7–14; regional, appendix 1 pp 15–135; national, appendix 1 p 136 and appendix 2).

Table 1 presents the health impact of bivalent or quadrivalent and nonavalent HPV vaccination of 9-year-old girls at 90% coverage during 2020–29 on the lifetime burden of cervical cancer in 177 countries for the five comparative scenarios (vaccine impact estimates for 12-year-old girls are presented in appendix 1 pp 137, 138). Compared with the vaccination impact in the base scenario (S1), the demography update with the UNWPP 2019 revision (S2) had the largest effect on estimates, resulting in a 33% increase in cases averted, a 50% increase in deaths averted, a 37% increase in YLD averted, a 76% increase in YLL averted, and a 73% increase in DALYs averted per 1000 vaccinated girls for the bivalent or quadrivalent and nonavalent vaccines. Updating disability weights with values from the GBD 2017 study (S3) resulted in a 54% decrease in YLD and a 4% decrease in DALYs per 1000 vaccinated girls for the bivalent or quadrivalent and nonavalent vaccines compared with S1, whereas it had no effect on cases, deaths, or YLL averted. Updating cervical cancer burden estimates with values from the GLOBOCAN 2018 database (S4) resulted in a 7% decrease in cases and a 6% decrease in YLD averted per 1000 vaccinated girls compared with S1, and resulted in a 2% increase in deaths, a 2% increase in YLL, and a 1% increase in DALYs averted per 1000 vaccinated girls compared with S1 for the bivalent or quadrivalent and nonavalent vaccines. The combined effect of updating the demography, disability weights, and cervical cancer burden estimates (S5) was an 18% decrease in YLD, a 26% increase in cases, a 51% increase in deaths, a 79% increase in YLL, and a 72% increase in DALYs averted per 1000 vaccinated girls for the bivalent or quadrivalent and nonavalent vaccines. Because the relative contribution of HPV 16/18 and HPV 16/18/31/33/45/52/58 to cervical cancer cases remains the same irrespective of updates to demography, disability weights, and cervical cancer burden, the percentage changes in the health impact of HPV vaccination are the same for the bivalent or quadrivalent and nonavalent vaccines.

**Table 1:** Global estimates of the impact of HPV vaccination of 9-year-old girls

	**Bivalent or quadrivalent HPV vaccination impact on cervical cancer caused by HPV 16/18**					**Nonavalent HPV vaccination impact on cervical cancer caused by HPV 16/18/31/33/45/52/58**				
	Cases	Deaths	YLDs	YLLs	DALYs	Cases	Deaths	YLDs	YLLs	DALYs
S1	12·2	7·6	9·6	131	141	15·4	9·6	12	166	178
S2	16·3	11·4	13·1	231	244	20·5	14·4	16·4	291	307
S3	12·2	7·6	4·4	131	136	15·4	9·6	5·5	166	171
S4	11·4	7·8	9	134	143	14·4	9·8	11·3	169	180
S5	15·4	11·5	7·9	235	243	19·3	14·4	9·9	296	306

Values are calculated on the basis of HPV vaccination of 9-year-old girls at 90% coverage during 2020–29, and they reflect the averted lifetime burden of cervical cancer in 177 countries for five comparative scenarios. All values are numbers averted per 1000 vaccinated girls. S1=Base scenario: WHO 2009 demography, GBD 2001 disability weights, GLOBOCAN 2012 cervical cancer burden. S2=Scenario 2: UNWPP 2019 demography, GBD 2001 disability weights, GLOBOCAN 2012 cervical cancer burden. S3=Scenario 3: WHO 2009 demography, GBD 2017 disability weights, GLOBOCAN 2012 cervical cancer burden. S4=Scenario 4: WHO 2009 demography, GBD 2001 disability weights, GLOBOCAN 2018 cervical cancer burden. S5=Scenario 5: UNWPP 2019 demography, GBD 2017 disability weights, GLOBOCAN 2018 cervical cancer burden. HPV=human papillomavirus. YLD=year lived with disability. YLL=year of life lost. DALY=disability-adjusted life-year. GBD=Global Burden of Disease study. GLOBOCAN=Global Cancer Incidence, Mortality and Prevalence. UNWPP=UN World Population Prospects.

In the base scenario (S1) with the bivalent or quadrivalent vaccine, the numbers of 9-year-old girls that were needed to be vaccinated were 82 to prevent one case of cervical cancer, 132 to prevent one death, and seven to prevent one DALY; with the nonavalent vaccine, the numbers were 65 to prevent one case, 105 to prevent one death, and six to prevent one DALY (table 2). In the fully updated model (S5), for the bivalent or quadrivalent vaccine, the numbers of girls that needed to be vaccinated were 65 to prevent one case, 87 to prevent one death, and four to prevent one DALY; for the nonavalent vaccine, the numbers needed were 52 girls to prevent one case, 69 to prevent one death, and three to prevent one DALY.

**Table 2:** Numbers of 9-year-old girls that need to be vaccinated to prevent cervical cancer

	Bivalent or quadrivalent HPV vaccine*					**Nonavalent HPV vaccine**†				
	1 case	1 death	1 YLD	1 YLL	1 DALY	1 case	1 death	1 YLD	1 YLL	1 DALY
S1	82	132	105	7·6	7·1	65	105	83	6	5·6
S2	61	88	77	4·3	4·1	49	70	61	3·4	3·3
S3	82	132	229	7·6	7·4	65	105	181	6	5·8
S4	88	129	112	7·5	7	70	102	89	5·9	5·6
S5	65	87	127	4·2	4·1	52	69	101	3·4	3·3

S1=Base scenario: WHO 2009 demography, GBD 2001 disability weights, GLOBOCAN 2012 cervical cancer burden. S2=Scenario 2: UNWPP 2019 demography, GBD 2001 disability weights, GLOBOCAN 2012 cervical cancer burden. S3=Scenario 3: WHO 2009 demography, GBD 2017 disability weights, GLOBOCAN 2012 cervical cancer burden. S4=Scenario 4: WHO 2009 demography, GBD 2001 disability weights, GLOBOCAN 2018 cervical cancer burden. S5=Scenario 5: UNWPP 2019 demography, GBD 2017 disability weights, GLOBOCAN 2018 cervical cancer burden. HPV=human papillomavirus. YLD=years lived with disability. YLL=years of life lost. DALY=disability-adjusted life-year. GBD=Global Burden of Disease study. GLOBOCAN=Global Cancer Incidence, Mortality and Prevalence. UNWPP=UN World Population Prospects.

* Values are numbers of girls needed to be vaccinated to prevent cervical cancer caused by HPV 16/18.

† Values are numbers of girls needed to be vaccinated to prevent cervical cancer caused by HPV 16/18/31/33/45/52/58.

The health impact of bivalent or quadrivalent and nonavalent HPV vaccination of 12-year-old girls at 90% coverage during 2020–29 on the averted lifetime burden of cervical cancer in 177 countries for the five comparative scenarios is presented in appendix 1 (p 139). The estimated numbers of 12-year-old girls needed to be vaccinated to prevent cervical cancer for the different scenarios are also presented (appendix 1 p 140). The estimated health benefits of vaccination of 12-year-old girls are similar but slightly decreased compared with vaccination of 9-year-old girls.

**Figure 2:**
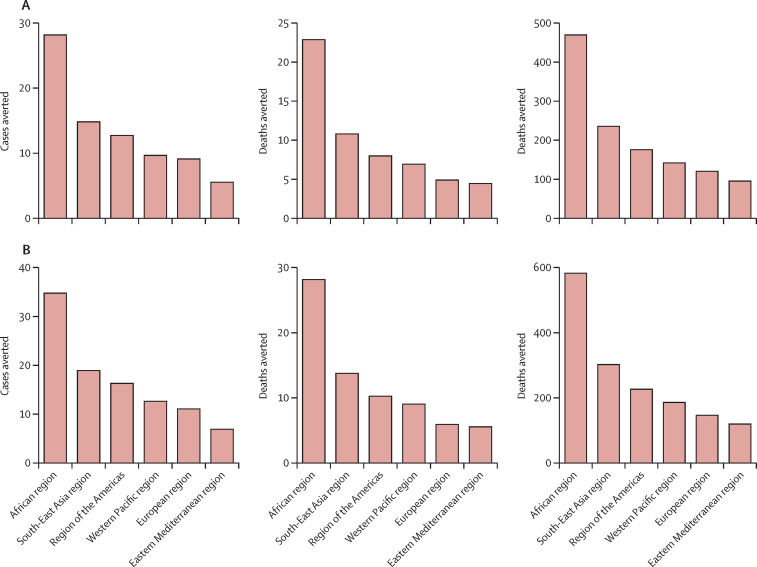
Vaccination impact per 1000 vaccinated 9-year-old girls at the regional level (A) Bivalent or quadrivalent vaccine. (B) Nonavalent vaccine. Values reflect lifetime health impact of HPV vaccination of 9-year-old girls during 2020–29 in the six WHO regions (estimates made after the combined PRIME updates for demography, disability weights, and cervical cancer burden). All values are numbers averted per 1000 vaccinated girls. DALY=disability-adjusted life-year. HPV=human papillomavirus. PRIME=Papillomavirus Rapid Interface for Modelling and Economics.

The lifetime health impact of HPV vaccination of 9-year-old girls during 2020–29 on disease burden averted in the six WHO regions is presented in figure 2 and table 3 (regional vaccine impact estimates for 12-year-old girls and for comparative scenarios are presented in appendix 1 [pp 141–67]). We estimate that the African region will gain the greatest health benefits from HPV vaccination, with 28 cases, 23 deaths, and 470 DALYs averted per 1000 vaccinated girls for the bivalent or quadrivalent vaccine, and 35 cases, 28 deaths, and 581 DALYs averted per 1000 vaccinated girls for the nonavalent vaccine. The African region has a high per-capita disease burden before vaccination, and therefore high numbers of cases, deaths, and DALYs would be averted after vaccination compared with other regions with lower per-capita disease burdens.

**Table 3:** Vaccination impact per 1000 vaccinated 9-year-old girls at the regional level

	**Bivalent or quadrivalent HPV vaccine**					**Nonavalent HPV vaccine**				
	Cases	Deaths	YLDs	YLLs	DALYs	Cases	Deaths	YLDs	YLLs	DALYs
African region	28	23	15	455	470	35	28	18	562	581
South-East Asia region	15	11	8	229	236	19	14	10	292	302
Region of the Americas	13	8	6	170	176	16	10	8	218	226
Western Pacific region	10	7	5	137	143	13	9	7	179	186
European region	9	5	4	117	121	11	6	5	141	147
Eastern Mediterranean region	6	4	3	93	96	7	6	4	116	120

Values reflect lifetime health impact of HPV vaccination of 9-year-old girls during 2020–29 in the six WHO regions (estimates made after the combined PRIME updates for demography, disability weights, and cervical cancer burden). All values are numbers averted per 1000 vaccinated girls. HPV=human papillomavirus. YLD=years lived with disability. YLL=years of life lost. DALY=disability-adjusted life-year. PRIME=Papillomavirus Rapid Interface for Modelling and Economics.

**Figure 3:**
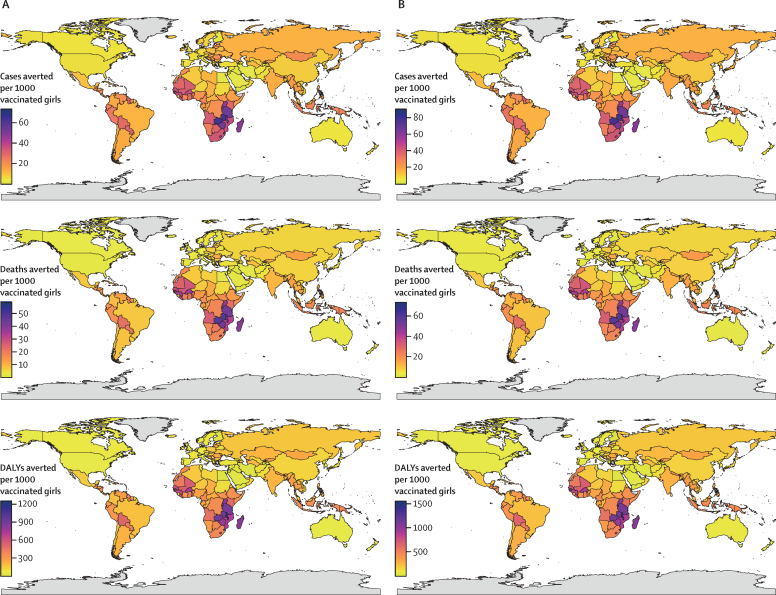
Impact of HPV vaccination of 9-year-old girls at the national level (A) Bivalent or quadrivalent vaccine. (B) Nonavalent vaccine. DALY=disability-adjusted life-year. HPV=human papillomavirus. Grey shading indicates no data were available.

Figure 3 presents the national estimates for the lifetime health impact of HPV vaccination of 9-year old girls during 2020–29 on disease burden averted per 1000 vaccinated girls in 177 countries, after the combined PRIME updates for demography, disability weights, and cervical cancer burden (see appendix 1 [pp 168, 169] for national estimates of vaccine impact for 12-year-old girls). Based on country-specific lifetime estimates, we estimated that a mean of 17 cases (median 13 [2·5th–97·5th percentile 3–51]), 12 deaths (9 [2–43]), 9 YLD (7 [2–27]), 252 YLL (188 [40–849]), and 261 DALYs (195 [42–876]) would be averted per 1000 vaccinated girls in these 177 countries for the bivalent or quadrivalent vaccine. We estimated that a mean of 21 cases (16 [4–63]), 15 deaths (10 [3–53]), 11 YLD (8 [2–33]), 314 YLL (237 [50–1049]), and 325 DALYs (244 [52–1082]) would be averted per 1000 vaccinated girls for the nonavalent vaccine. National estimates of HPV vaccination impact for the comparative scenarios are presented in the appendices (appendix 1 pp 170–211; appendix 2). Updated national estimates of HPV vaccination impact for bivalent or quadrivalent and nonavalent vaccination of 9-year-old and 12-year-old girls are presented in appendix 1 (pp 212–39).

In comparison with the counterfactual scenario of no vaccination, routine vaccination of 9-year-old girls during 2020–29 would have the greatest health benefits in eSwatini, Zambia, Malawi, Tanzania, Zimbabwe, and Burundi. 72, 63, 56, 54, and 53 cases of cervical cancer would be averted for the bivalent or quadrivalent vaccine and 88, 78, 69, 67, and 66 cases averted for the nonavalent vaccine per 1000 vaccinated girls in eSwatini, Zambia, Malawi, Tanzania, and Zimbabwe, respectively. 58, 47, 45, 44, and 43 deaths would be averted for the bivalent or quadrivalent vaccine and 72, 59, 55, 54, and 54 deaths averted for the nonavalent vaccine per 1000 vaccinated girls in eSwatini, Zambia, Malawi, Burundi, and Zimbabwe, respectively. 1228, 966, 947, 903, and 897 DALYs would be averted for the bivalent or quadrivalent vaccine and 1517, 1194, 1171, 1117, and 1108 DALYs averted for the nonavalent vaccine per 1000 vaccinated girls in Malawi, Burundi, eSwatini, Zambia, and Tanzania, respectively. The countries to potentially gain the largest HPV vaccination impact are the same for bivalent or quadrivalent and nonavalent vaccination of 9-year-old and 12-year-old girls.

The countries that will experience the largest health benefits through HPV vaccination are all in the WHO African region. Within the other regions, the countries that will experience the greatest number of DALYs averted through HPV vaccination are the Maldives, Indonesia, and Nepal in the South-East Asia region; Bolivia, Jamaica, and Belize in the region of the Americas; Fiji, the Solomon Islands, and Papua New Guinea in the Western Pacific region; Kyrgyzstan, Romania, and Lithuania in the European region; and Morocco, Somalia, and Djibouti in the Eastern Mediterranean region.

In comparison with previous estimates, the updated estimates resulted in increases in the numbers of cervical cancer cases averted per 1000 vaccinated girls in most countries, with the greatest increases in eSwatini, Namibia, Lesotho, Burkina Faso, and Iceland. The greatest increases in the numbers of deaths averted per 1000 vaccinated girls were in Malta, eSwatini, Namibia, Lebanon, and Samoa, and in DALYs averted per 1000 vaccinated girls were in Iceland, Malta, Bahrain, Maldives, and eSwatini. These increases in cases, deaths, and DALYs averted are primarily due to the demography update of UNWPP 2019, which includes increasing life expectancy and population aging among women in these countries, and a relative increase in cervical cancer burden estimates from the GLOBOCAN 2018 database.

The updated HPV vaccination impact estimates for cases, deaths, and DALYs averted per 1000 vaccinated girls are lower than the previous estimates for 49, 18, and eight countries, respectively. These decreases are primarily due to lower estimates of cervical cancer burden for these countries in the GLOBOCAN 2018 database compared with the GLOBOCAN 2012 database. The changes in vaccination impact between the different comparative scenarios (S2–5) compared with the base scenario (S1) for vaccination of 9-year-old and 12-year-old girls are provided in appendix 1 (pp 240–320).

## Discussion

We updated PRIME with demography from the UNWPP 2019 revision, disability weights from the GBD 2017 study, and cervical cancer burden from the GLOBOCAN 2018 database. We assessed the effects of these updates on the health impact estimates generated by the model for bivalent or quadrivalent and nonavalent vaccination of 9-year-old and 12-year-old girls at 90% coverage during 2020–29 at the global, regional, and national levels for 177 countries. After these updates, we found that HPV vaccination provides larger health benefits than previously estimated, because the number of girls needed to be vaccinated to prevent a single case, death, or DALY are lower than previous forecasts.^11^ Vaccination is also more cost-effective than previously estimated, because the number of cases, deaths, and DALYs averted per vaccinated girl is higher than our earlier estimates. Furthermore, because of a higher burden of cervical cancer before vaccination in the WHO African region compared with other regions, HPV vaccination will provide the greatest relative health benefits in this region and the countries within this region should be prioritised for HPV vaccine introduction and scale-up. Based on WHO-initiated guidance on priority setting in health care,^24^ the African region has the greatest potential to benefit from HPV vaccination, followed by the South-East Asia region, the region of the Americas, Western Pacific region, European region, and Eastern Mediterranean region.

We found that the demography update from the WHO 2009 life tables to the UNWPP 2019 revision was the major driver for change in the health impact estimates of PRIME, in comparison with the updates to disability weights or cervical cancer burden estimates. Because UNWPP mortality estimates are declining over time and incorporate population aging (unlike static WHO life tables), using UNWPP 2019 estimates led to an increasing life expectancy among women and a subsequent increase in lifetime risk of cervical cancer incidence and mortality without vaccination. Therefore, HPV vaccination provides greater benefits in preventing new cases and deaths than previously estimated.

HPV vaccination could have an even greater impact and be more cost-effective than predicted in this study, because some assumptions in PRIME are conservative and likely to underestimate the impact of vaccination. These conservative assumptions and study limitations are that herd effects are not considered; vaccination is considered to have no effect on women after sexual debut; cervical cancer incidence is assumed to remain constant in the absence of preventive interventions; and cross-protection against non-vaccine HPV genotypes is excluded. Other limitations are exclusion of transmission dynamics of HPV infection and natural history of precancer lesions; exclusion of changes in coverage and effectiveness of non-vaccine interventions (ie, screening and treatment of precancer lesions); and exclusion of changes in coverage and effectiveness of treatment and care of cervical cancer cases. The combined impact of vaccine and non-vaccine interventions are significant strategic considerations, and the working draft of the WHO 90–70–90 cervical cancer elimination strategy has set targets of achieving 90% coverage of HPV vaccination; 70% coverage for screening and 90% treatment of precancer lesions; and 90% treatment and care of cervical cancer cases.^10^ PRIME complements models that include additional features to analyse the potential impact of comprehensive cervical cancer prevention and control programmes and supports strategic decision making.

The structural uncertainty and variability between HPV vaccine impact models warrants a comparison of the models to identify key drivers of the conclusions on the effectiveness and cost-effectiveness of vaccination.^25^, ^26^ Evidence synthesis from different models will improve the fidelity of the decision-making process in formulating the optimal strategy for cervical cancer elimination within a century.^27^, ^28^ Although we infer that demographic assumptions are key drivers of HPV vaccine impact predictions in this study, demography is a critical input to modelling the health and economic impact of vaccination in general.

Sexual debut data by age for 67 countries are included in this study and future directions include estimation for other countries. Although we have not explored the impact of changes in sexual behaviour, countries that are transitioning from traditional age-specific sexual behaviour to gender-similar age-specific sexual behaviour, with increases in the average number of partners and the number of partners women have before marriage, are likely to result in increased prevalence of HPV infection and incidence of cervical cancer.^29^

PRIME updates for demography, disability weights, and cervical cancer burden improve the fidelity of the projections of HPV vaccination impact at the global, regional, and national levels, and provide essential information for decision making in the era of achieving cervical cancer elimination. Given that cervical cancer elimination is likely to take many decades, HPV vaccine impact models must be continually updated to reflect new knowledge about parameter estimates and disease burden.^11^, ^30^, ^31^ The updated PRIME estimates for HPV vaccination impact suggest greater health benefits and therefore increased cost-effectiveness of vaccination in comparison with previous estimates. Investments to increase HPV vaccination coverage and equity need to be continued, and countries in the WHO African region should be prioritised for HPV vaccine introduction and scale-up.
