# Correction to *Lancet Glob Health* 2020; 8: e536–44

**DOI:** 10.1016/S2214-109X(21)00041-3

**Published:** 2021-02-16

*Abbas KM, van Zandvoort K, Brisson M, Jit M. Effects of updated demography, disability weights, and cervical cancer burden on estimates of human papillomavirus vaccination impact at the global, regional, and national levels: a PRIME modelling study*. Lancet Glob Health *2020; **8:** e536–44—*In figure 2 of this Article, the vertical axes in the right-hand graphs should have been labelled as DALYs averted. This correction has been made as of Feb 16, 2021.

