# Supplementary material for: Effects of updated demography, disability weights, and cervical cancer burden on estimates of human papillomavirus vaccination impact at the global, regional, and national levels: a PRIME modelling study
Source: Lancet Glob Health. 2020 Feb 24;8(4):e536–44. doi: 10.1016/S2214-109X(20)30022-X (PMC7083230; doi:10.1016/S2214-109X(20)30022-X)

THE LANCET
Global Health

Supplementary appendix 1

This appendix formed part of the original submission and has been peer reviewed. We post it as supplied by the authors.

Supplement to: Abbas KM, van Zandvoort K, Brisson, Mark Jit M. Effects of updated demography, disability weights, and cervical cancer burden on estimates of human papillomavirus vaccination impact at the global, regional, and national levels: a PRIME modelling study. *Lancet Glob Health* 2020; published online Feb 24. http://dx.doi.org/10.1016/S2214-109X(20)30022-X.

Appendix

Table of contents

A1. PRIME model and software	2
A2. Disability weights and morbidity attribution	3
A3. Comparative scenarios to assess the health impact of HPV vaccination using PRIME	5
A4. Cervical cancer burden pre- and post-vaccination of 12-year-old girls at the global level	6
A5. Cervical cancer burden pre- and post-vaccination at the global level	7
A6. Cervical cancer burden pre- and post-vaccination at the regional level	15
A7. Cervical cancer burden pre- and post-vaccination at the national level	136
A8. Vaccination impact per 1000 vaccinated girls at the global level	137
A9. Global estimates of HPV vaccination impact of 12-year-old girls	139
A10. Number of 12-year-old girls needed to be vaccinated to prevent cervical cancer	140
A11. Vaccination impact per 1000 vaccinated 12-year-old girls at the regional level	141
A12. Vaccination impact per 1000 vaccinated 12-year-old girls at the regional level	142
A13. Vaccination impact per 1000 vaccinated girls at the regional level	143
A14. HPV vaccination impact of 12-year-old girls at the national level	168
A15. National estimates of HPV vaccination impact for comparative scenarios	170
A16. Vaccination impact per 1000 vaccinated girls at the national level	211
A17. Vaccination impact per 1000 vaccinated girls at the national level – updated PRIME estimates	212
A18. National estimates of HPV vaccination impact	233
A19. Vaccination impact in comparative scenarios	240

A1. PRIME model and software

The Excel-based version of Papillomavirus Rapid Interface for Modelling and Economics (PRIME) and documentation are accessible online (http://primetool.org/) for use by country program managers and planners to facilitate country-specific decision-making in low and middle-income countries.[31] We have also developed an R package (*prime*) which was used to generate the vaccine impact estimates used in this study and is accessible on GitHub (https://github.com/lshtm-vimc/prime). The program for this study analysis is accessible on GitHub (https://github.com/vaccine-impact/prime_update) to facilitate reproducibility and reusability.

A2. Disability weights and morbidity attribution

Table A2.1. Disability weights and durations for different phases of cervical cancer in GBD 2001 and 2017 studies.

Cervical cancer phase/ sequela	Health state description[23]	Disability weights		Duration	
		GBD 2001	GBD 2017	GBD 2001	GBD 2017
Diagnosis and primary therapy phase	has pain, nausea, fatigue, weight loss and high anxiety	0.08	0.288 (0.193 - 0.399)	1 year	4.8 months
Controlled phase	has a chronic disease that requires medication every day but minimal interference with daily activities	0.04 (A)<br>0.11 (B)<br>0.13 (C)<br>0.17 (D/E)<br>(A, B, C, D, E - WHO mortality stratums)	0.049 (0.031 - 0.072)	4 years	remainder of time after attributing other sequelae
Metastatic phase	has severe pain, extreme fatigue, weight loss and high anxiety	0.78	0.451 (0.307 - 0.6)	6 months	9.21 months
Terminal phase	has lost a lot of weight and regularly uses strong medication to avoid constant pain	0.78	0.54 (0.377 - 0.687)	6 months	1 month

Table A2.2. Morbidity attribution

Morbidity attribution by age of incidence	Morbidity attribution by age of prevalence
**YLD = I * DW * L**<br>I = incidence (new cases in a given year)<br>DW = disability weight<br>L = average duration of case until remission or death (years)	**YLD = P * DW * L**<br>P = prevalence (in a given year)<br>DW = disability weight<br>L = average duration of case in a given year (maximum of 1 year)

Incidence, prevalence and mortality for a given age and specific country are defined as follows:

- Incidence at age *a* in country *c* is the proportion in the specified population who were diagnosed with cervical cancer (at age *a*).
- Prevalence at age *a* in country *c* is the proportion in the specified population who had been diagnosed with cervical cancer within the last 5 years and are still alive.
- Mortality at age *a* in country *c* is the proportion in the specified population who died due to cervical cancer.

Morbidity attribution to the age of incidence

In the Global Burden of Disease (GBD) 2001 study, morbidity (i.e., YLDs - years lived with disability) was attributed to the age of incidence using GBD 2001-specific disability weights and durations for different phases of cervical cancer.

$$\begin{aligned} YLD_{a,\,c} = {} & (\text{incidence}_{a,\,c} - \text{mortality}_{a,\,c}) * (\text{duration}_{\textit{diagnosis phase}} * \text{disability weight}_{\textit{diagnosis phase}} + \\ & \text{duration}_{\textit{control phase}} * \text{disability weight}_{\textit{control phase}}) + \\ & \text{mortality}_{a,\,c} * (\text{duration}_{\textit{diagnosis phase}} * \text{disability weight}_{\textit{diagnosis phase}} + \\ & \text{duration}_{\textit{metastatic phase}} * \text{disability weight}_{\textit{metastatic phase}} + \\ & \text{duration}_{\textit{terminal phase}} * \text{disability weight}_{\textit{terminal phase}}) \end{aligned}$$

Morbidity attribution to the age of prevalence

In GBD 2017 study, morbidity was attributed to the age of prevalence using GBD 2017-specific disability weights and durations for different phases of cervical cancer.

$$\begin{aligned} YLD_{a,\,c} = {} & \text{incidence}_{a,\,c} * (\text{duration}_{\textit{diagnosis phase}} * \text{disability weight}_{\textit{diagnosis phase}}) + \\ & \text{prevalence}_{a,\,c} * (\text{disability weight}_{\textit{control phase}}) + \\ & \text{mortality}_{a,\,c} * (\text{duration}_{\textit{metastatic phase}} * \text{disability weight}_{\textit{metastatic phase}} + \\ & \text{duration}_{\textit{terminal phase}} * \text{disability weight}_{\textit{terminal phase}}) \end{aligned}$$

A3. Comparative scenarios to assess the health impact of HPV vaccination using PRIME

Scenario	Demography	Disability weights	Cervical cancer burden
s1	WHO (2009)	GBD 2001	GLOBOCAN 2012
s2	UNWPP (2019)	GBD 2001	GLOBOCAN 2012
s3	WHO (2009)	GBD 2017	GLOBOCAN 2012
s4	WHO (2009)	GBD 2001	GLOBOCAN 2018
s5	UNWPP (2019)	GBD 2017	GLOBOCAN 2018

A4. Cervical cancer burden pre- and post-vaccination of 12-year-old girls at the global level

Lifetime cervical cancer burden in terms of cases, deaths and DALYs pre and post bivalent/quadrivalent and nonavalent HPV vaccination of 12-year-old girls at 90% coverage in 177 countries during 2020-2029, which relates to birth cohorts of 2008-2017 (estimates after the combined PRIME updates for demography, disability weights and cervical cancer burden).

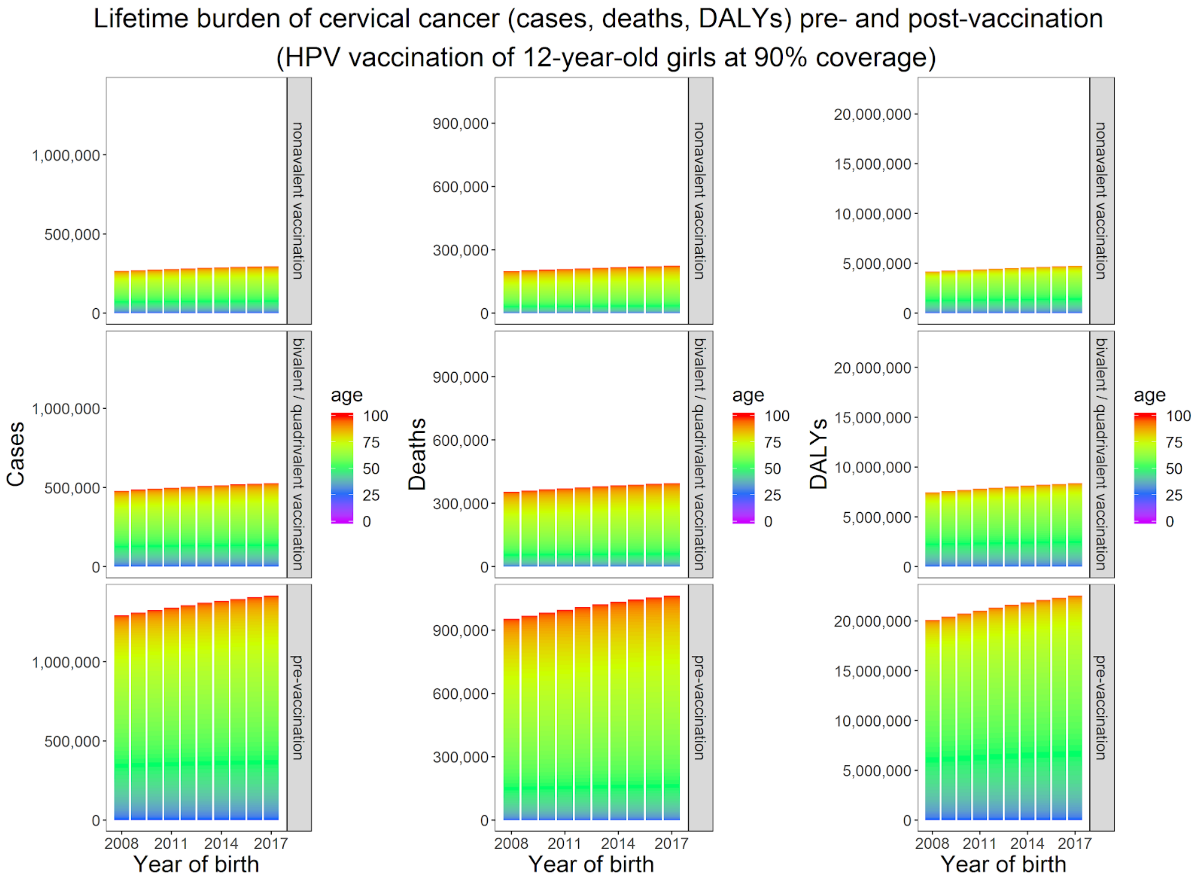

A5. Cervical cancer burden pre- and post-vaccination at the global level

The lifetime burden of cervical cancer caused by HPV 16/18 and HPV 16/18/31/33/45/52/58 in terms of cases, deaths, YLDs, YLLs and DALYs pre- and post-vaccination at 90% coverage during 2020-2029 in 177 countries for bivalent/quadrivalent and nonavalent vaccination of 9-year-old and 12-year-old girls. The bivalent/quadrivalent vaccine protects against cervical cancers caused by HPV 16/18 while the nonavalent vaccine protects against cervical cancers caused by HPV 16/18/31/33/45/52/58.

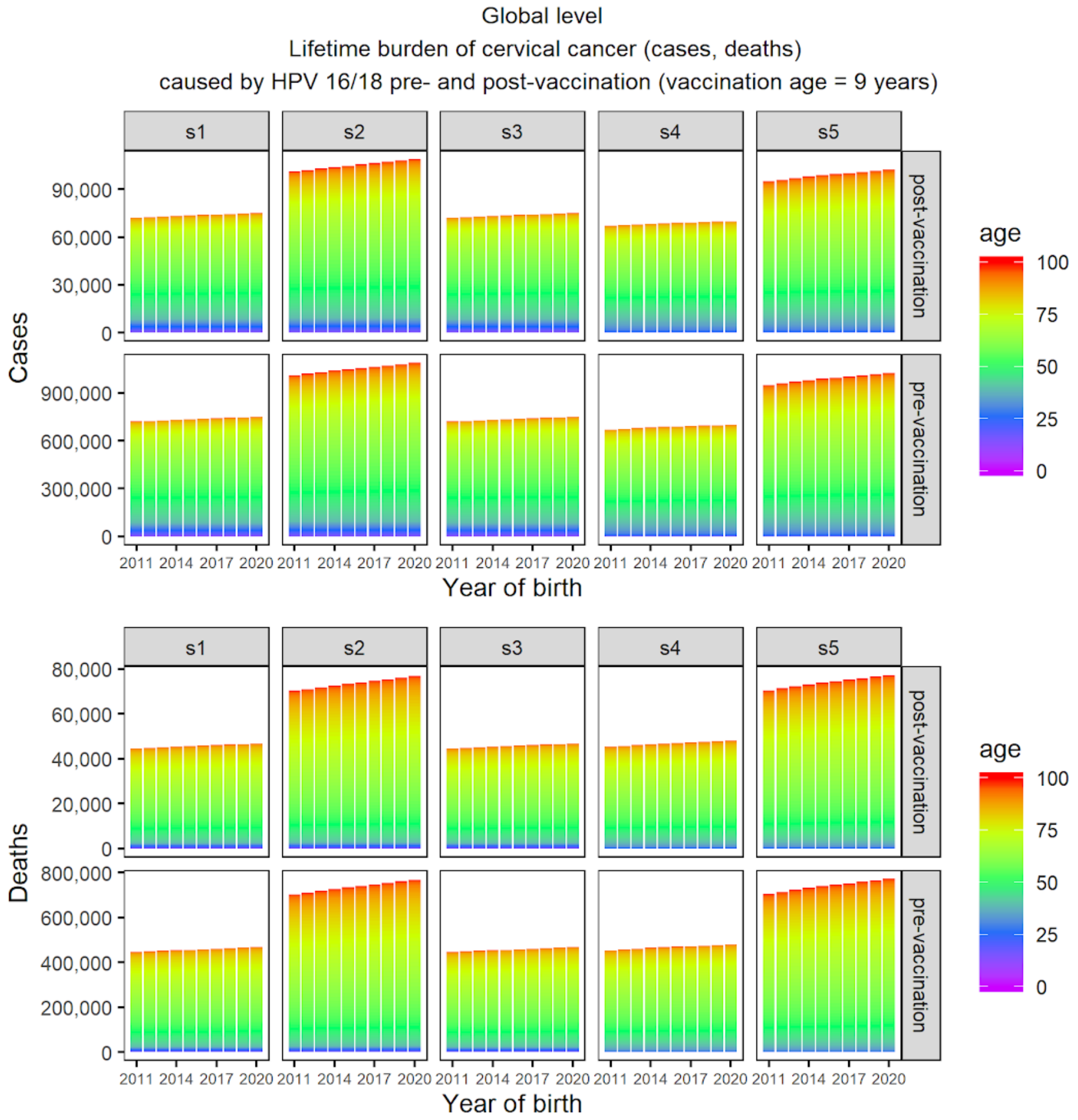

s1 - WHO 2009 demography, GBD 2001 disability weights, GLOBOCAN 2012 cervical cancer burden
s2 - UNWPP 2019 demography, GBD 2001 disability weights, GLOBOCAN 2012 cervical cancer burden
s3 - WHO 2009 demography, GBD 2017 disability weights, GLOBOCAN 2012 cervical cancer burden
s4 - WHO 2009 demography, GBD 2001 disability weights, GLOBOCAN 2018 cervical cancer burden
s5 - UNWPP 2019 demography, GBD 2017 disability weights, GLOBOCAN 2018 cervical cancer burden

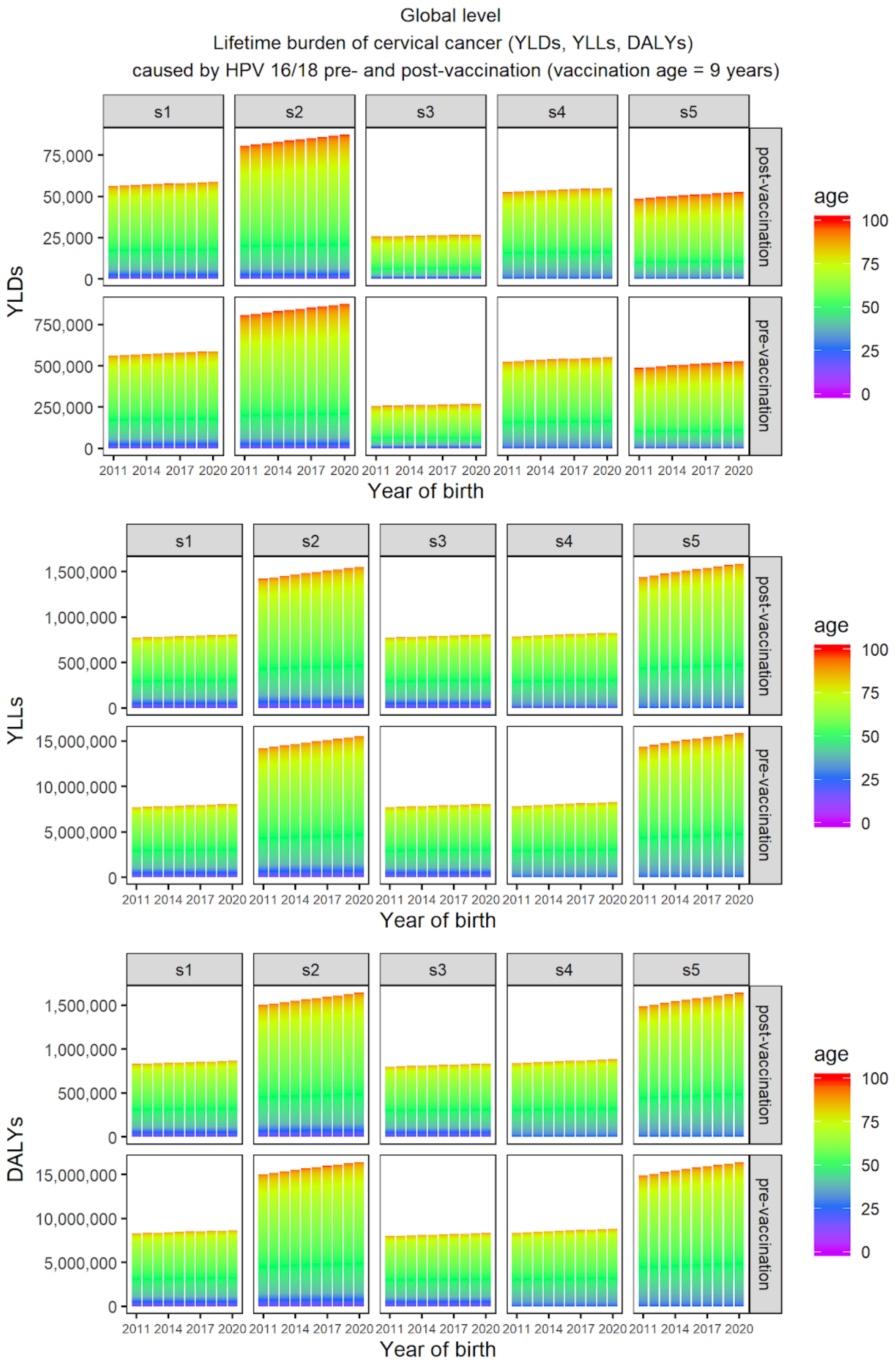
Global level
Lifetime burden of cervical cancer (YLDs, YLLs, DALYs)
caused by HPV 16/18 pre- and post-vaccination (vaccination age = 9 years)
s1
s2
s3
s4
s5
post-vaccination
pre-vaccination
YLDs
75,000
50,000
25,000
0
750,000
500,000
250,000
2011 2014 2017 2020
Year of birth
age
100
75
50
25
0
YLLs
1,500,000
1,000,000
500,000
15,000,000
10,000,000
5,000,000
DALYs

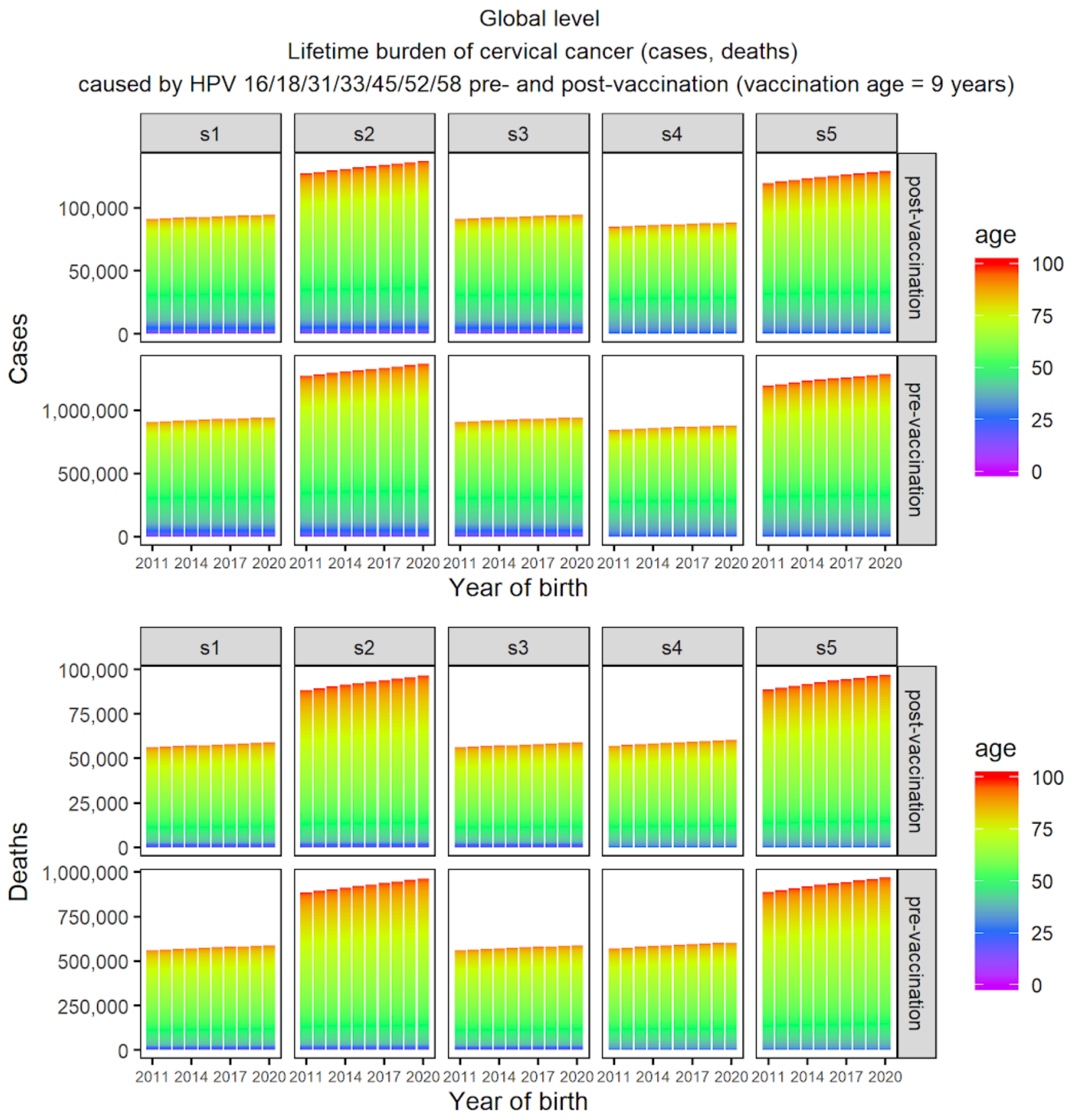
Global level
Lifetime burden of cervical cancer (cases, deaths)
caused by HPV 16/18/31/33/45/52/58 pre- and post-vaccination (vaccination age = 9 years)
s1
s2
s3
s4
s5
post-vaccination
pre-vaccination
Cases
100,000
50,000
0
1,000,000
500,000
0
2011
2014
2017
2020
Year of birth
age
100
75
50
25
0
Deaths
100,000
75,000
50,000
25,000
0
1,000,000
750,000
500,000
250,000
0
Year of birth

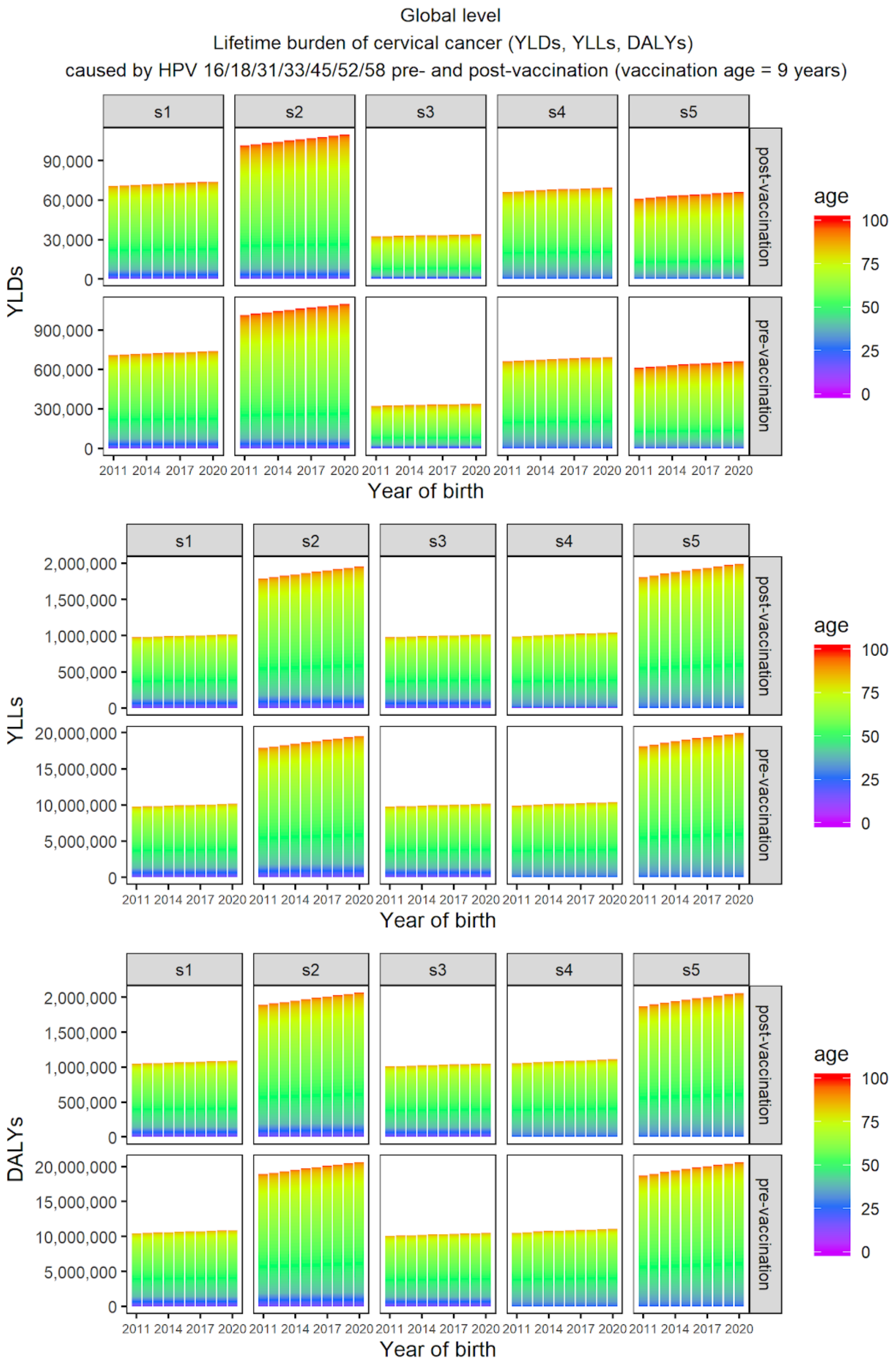

Global level
Lifetime burden of cervical cancer (YLDs, YLLs, DALYs)
caused by HPV 16/18/31/33/45/52/58 pre- and post-vaccination (vaccination age = 9 years)
s1
s2
s3
s4
s5
post-vaccination
pre-vaccination
YLDs
90,000
60,000
30,000
0
900,000
600,000
300,000
2011 2014 2017 2020
Year of birth
age
100
75
50
25
YLLs
2,000,000
1,500,000
1,000,000
500,000
20,000,000
15,000,000
10,000,000
5,000,000
DALYs

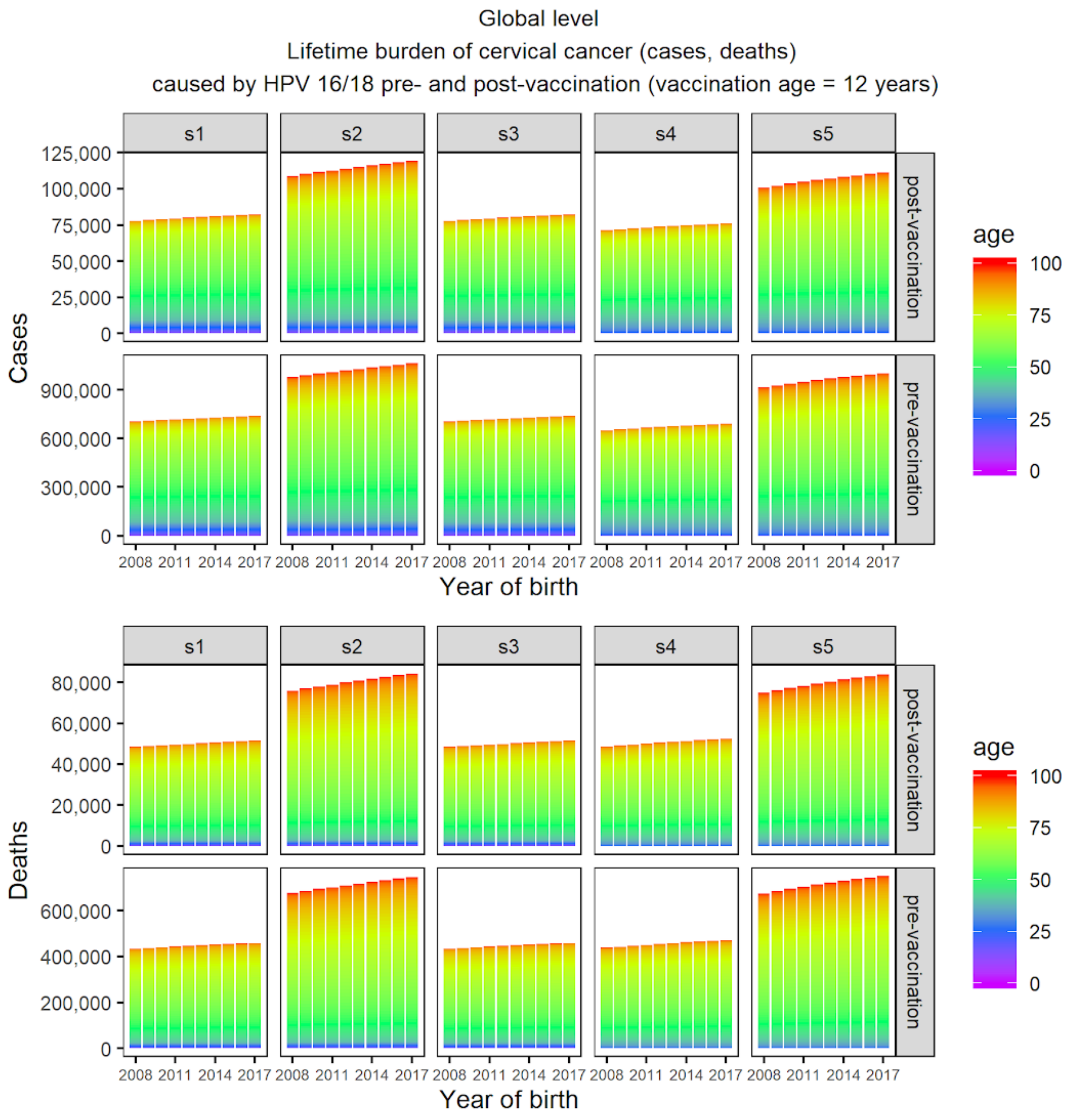
Global level
Lifetime burden of cervical cancer (cases, deaths)
caused by HPV 16/18 pre- and post-vaccination (vaccination age = 12 years)
s1
s2
s3
s4
s5
post-vaccination
pre-vaccination
Cases
Deaths
age
100
75
50
25
0
125,000
100,000
75,000
50,000
25,000
900,000
600,000
300,000
80,000
60,000
40,000
20,000
400,000
200,000
2008 2011 2014 2017
Year of birth

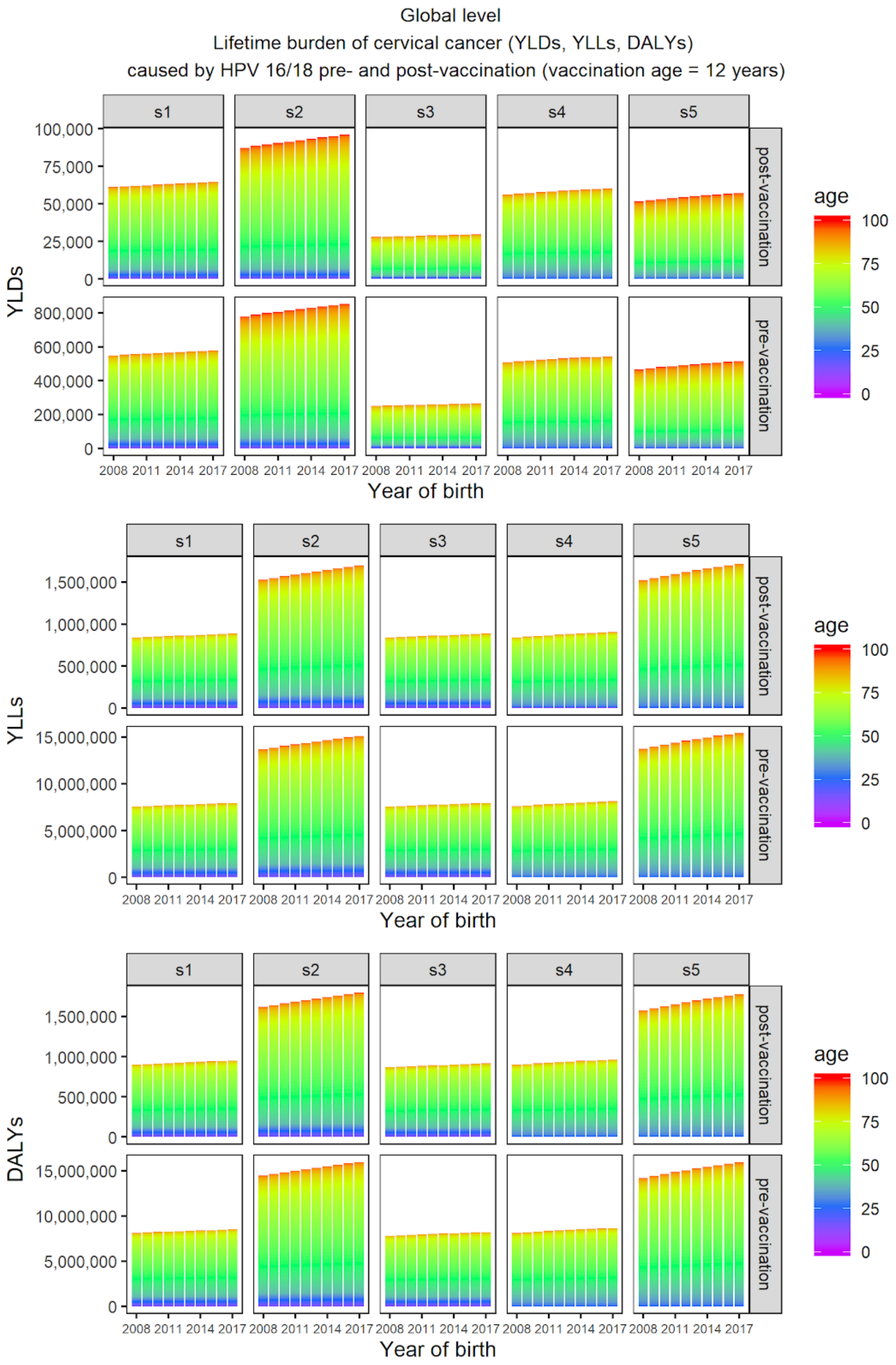

Global level
Lifetime burden of cervical cancer (YLDs, YLLs, DALYs)
caused by HPV 16/18 pre- and post-vaccination (vaccination age = 12 years)
s1
s2
s3
s4
s5
post-vaccination
pre-vaccination
YLDs
YLLs
DALYs
Year of birth
age
100
75
50
25
0
2008 2011 2014 2017
100,000
75,000
50,000
25,000
800,000
600,000
400,000
200,000
1,500,000
1,000,000
500,000
15,000,000
10,000,000
5,000,000

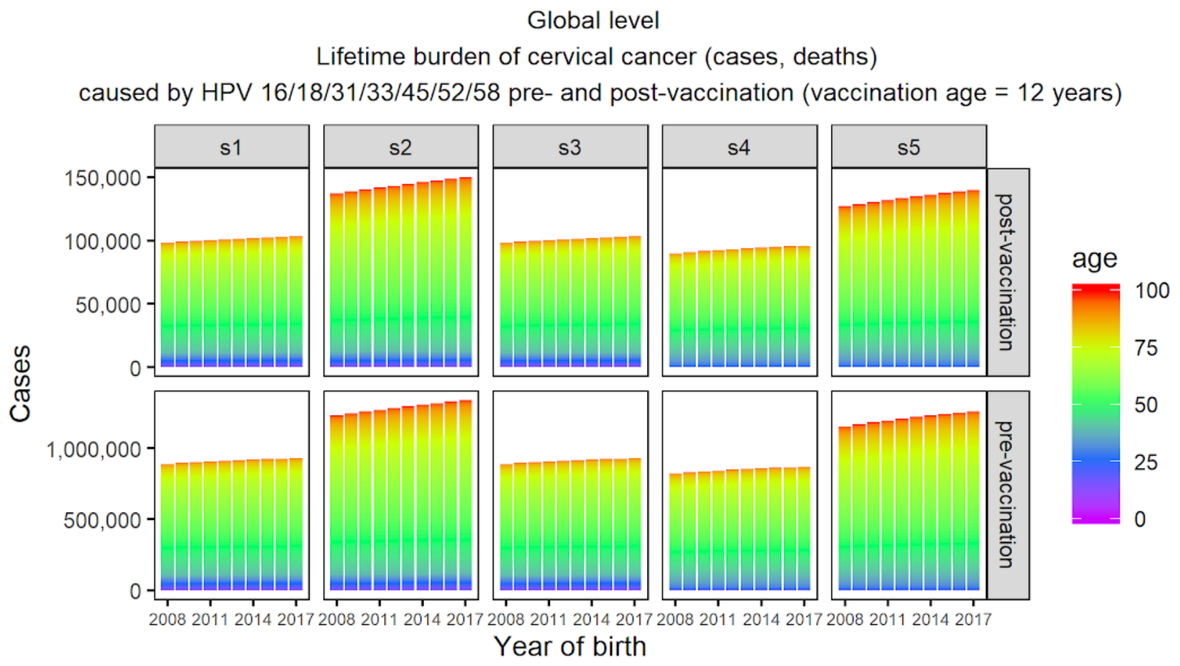

Global level
Lifetime burden of cervical cancer (cases, deaths)
caused by HPV 16/18/31/33/45/52/58 pre- and post-vaccination (vaccination age = 12 years)
s1
s2
s3
s4
s5
Cases
150,000
100,000
50,000
0
1,000,000
500,000
0
post-vaccination
pre-vaccination
age
100
75
50
25
0
2008 2011 2014 2017
Year of birth

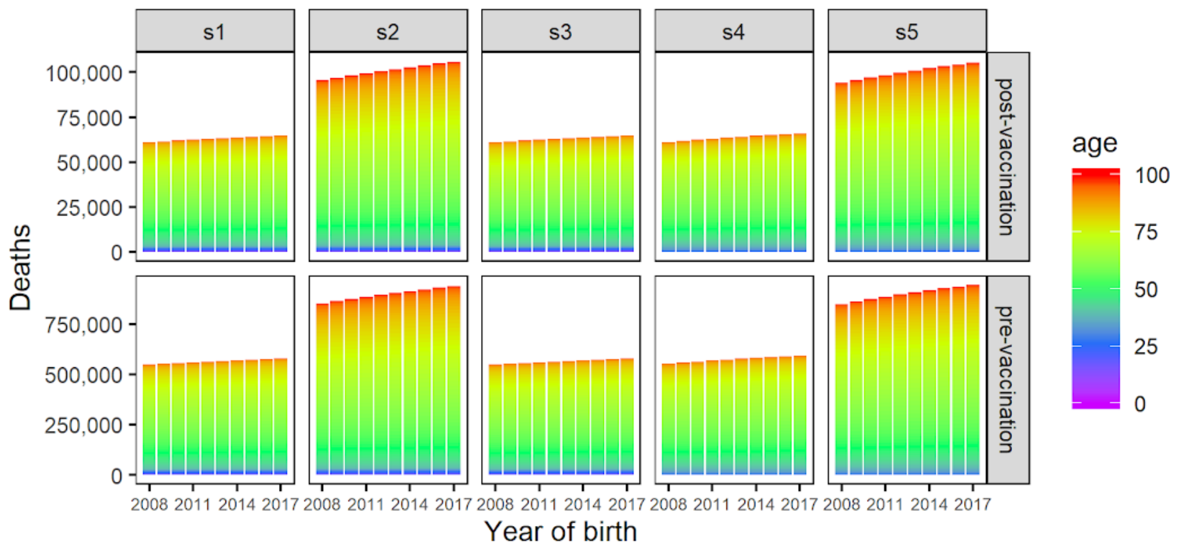

s1
s2
s3
s4
s5
Deaths
100,000
75,000
50,000
25,000
0
750,000
500,000
250,000
0
post-vaccination
pre-vaccination
age
100
75
50
25
0
2008 2011 2014 2017
Year of birth

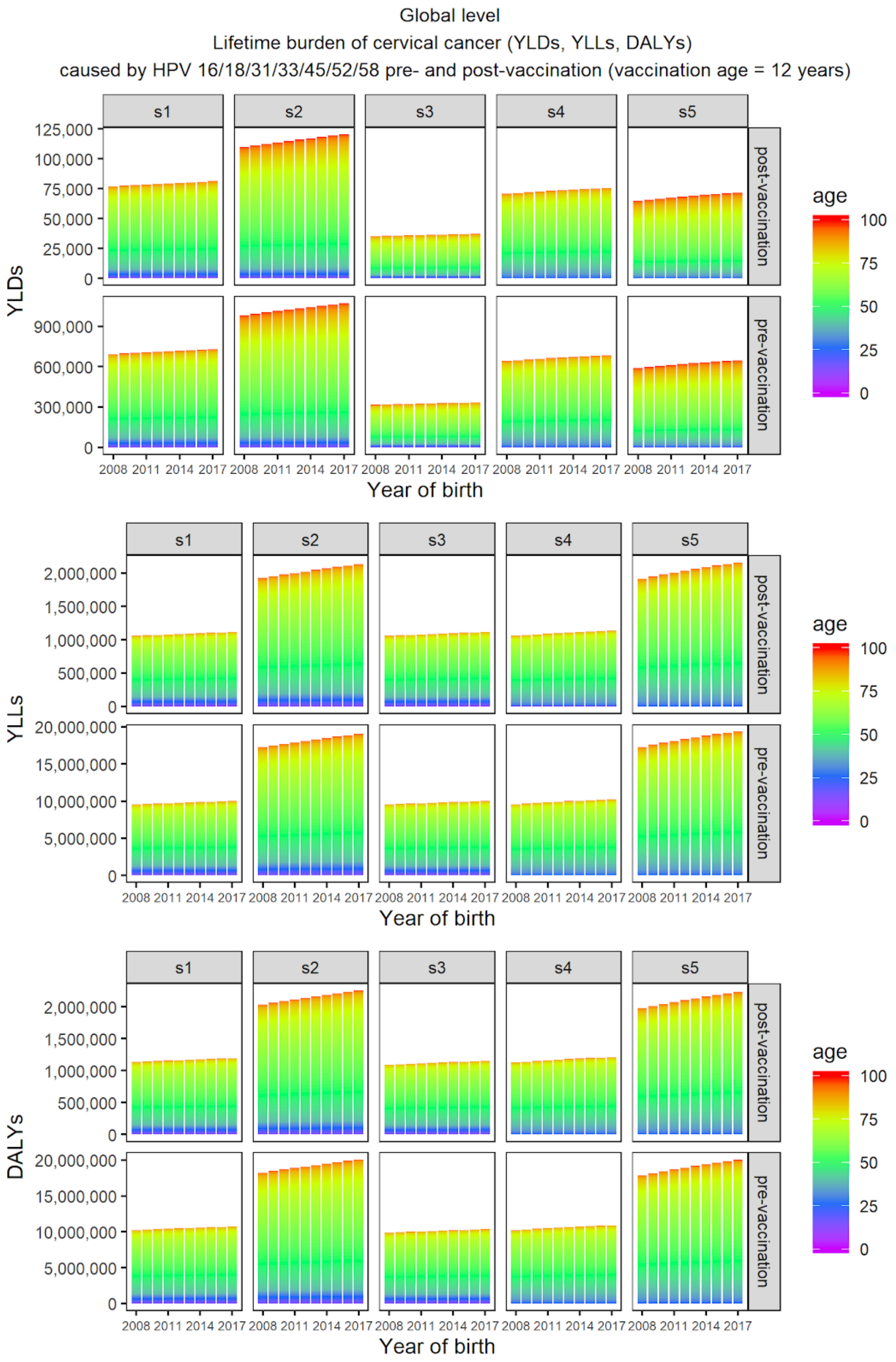
Global level
Lifetime burden of cervical cancer (YLDs, YLLs, DALYs)
caused by HPV 16/18/31/33/45/52/58 pre- and post-vaccination (vaccination age = 12 years)
s1
s2
s3
s4
s5
post-vaccination
pre-vaccination
YLDs
125,000
100,000
75,000
50,000
25,000
0
900,000
600,000
300,000
0
YLLs
2,000,000
1,500,000
1,000,000
500,000
20,000,000
15,000,000
10,000,000
5,000,000
DALYs
2008 2011 2014 2017
Year of birth
age
100
75
50
25
0

A6. Cervical cancer burden pre- and post-vaccination at the regional level

The lifetime burden of cervical cancer caused by HPV 16/18 and HPV 16/18/31/33/45/52/58 in terms of cases, deaths, YLDs, YLLs and DALYs pre- and post-vaccination at 90% coverage during 2020-2029 in the 6 WHO regions for bivalent/quadrivalent and nonavalent vaccination of 9-year-old and 12-year-old girls.

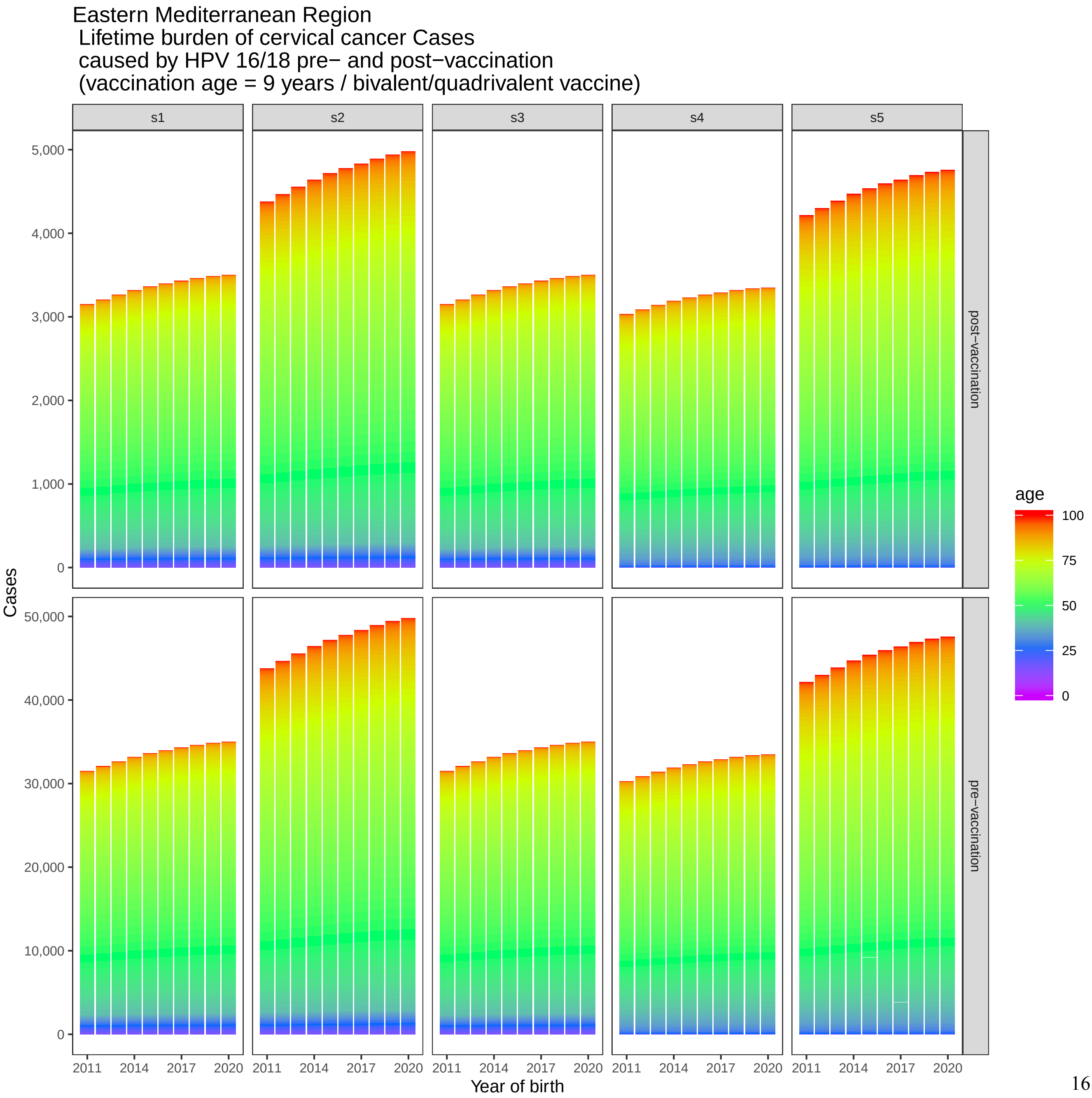

Eastern Mediterranean Region
Lifetime burden of cervical cancer Cases
caused by HPV 16/18 pre− and post−vaccination
(vaccination age = 9 years / bivalent/quadrivalent vaccine)
s1
s2
s3
s4
s5
post−vaccination
pre−vaccination
Cases
Year of birth
age
0
25
50
75
100
5,000
4,000
3,000
2,000
1,000
50,000
40,000
30,000
20,000
10,000
2011
2014
2017
2020

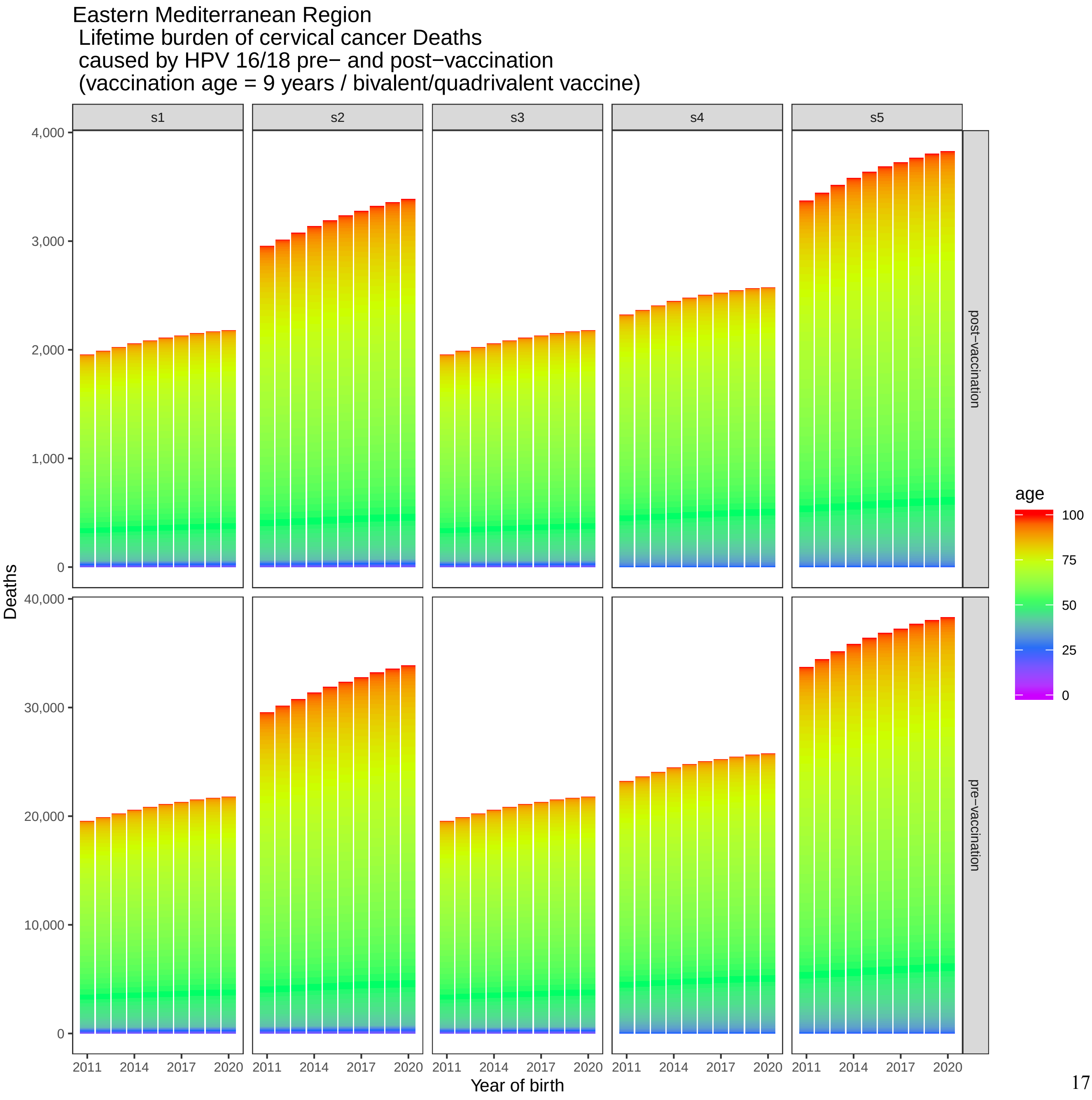
Eastern Mediterranean Region
Lifetime burden of cervical cancer Deaths
caused by HPV 16/18 pre− and post−vaccination
(vaccination age = 9 years / bivalent/quadrivalent vaccine)
s1
s2
s3
s4
s5
post−vaccination
pre−vaccination
Deaths
0
1,000
2,000
3,000
4,000
10,000
20,000
30,000
40,000
Year of birth
2011
2014
2017
2020
age
0
25
50
75
100

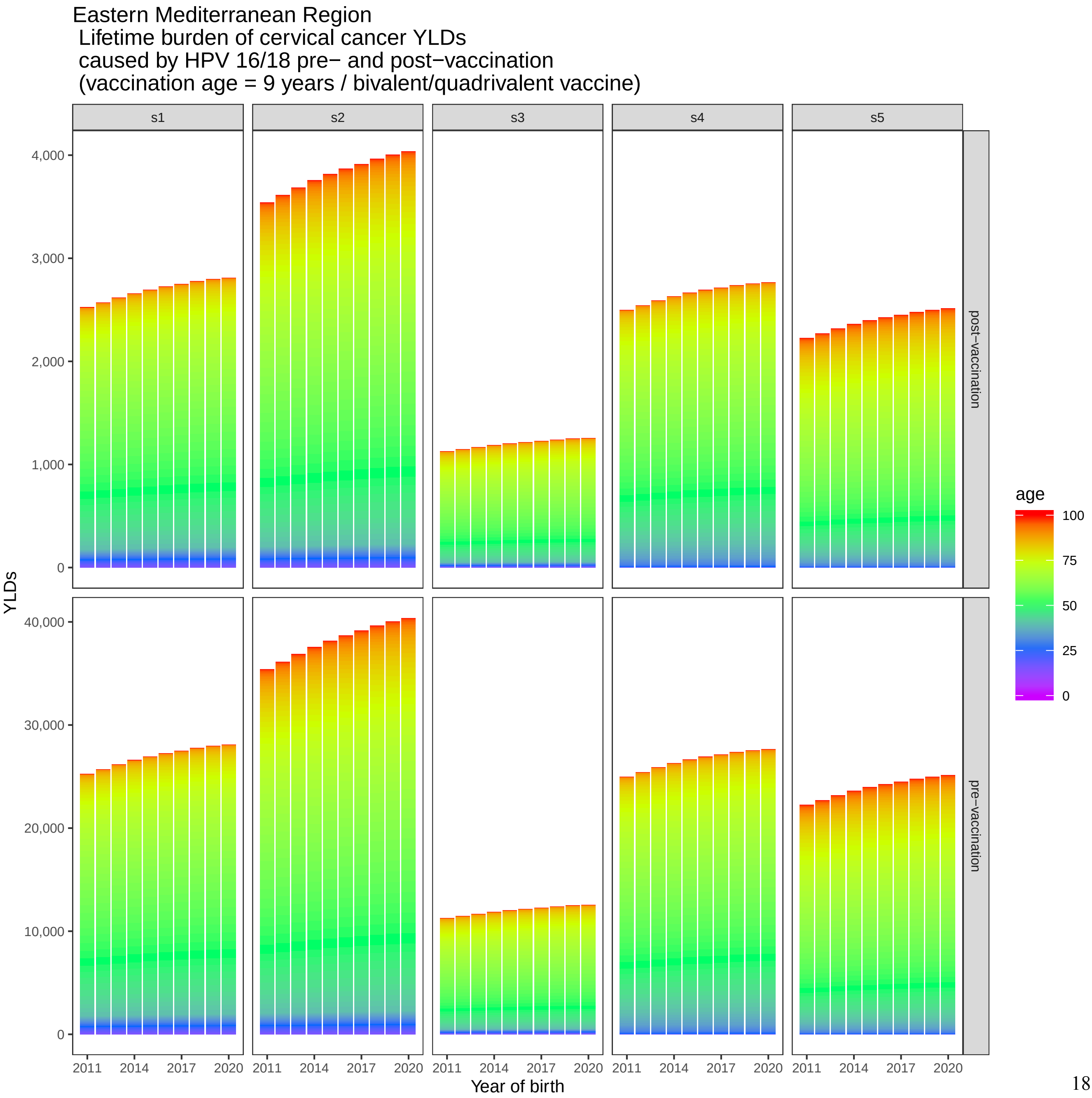

Eastern Mediterranean Region
Lifetime burden of cervical cancer YLDs
caused by HPV 16/18 pre− and post−vaccination
(vaccination age = 9 years / bivalent/quadrivalent vaccine)
s1
s2
s3
s4
s5
post−vaccination
pre−vaccination
YLDs
Year of birth
age
4,000
3,000
2,000
1,000
0
40,000
30,000
20,000
10,000
2011
2014
2017
2020
100
75
50
25

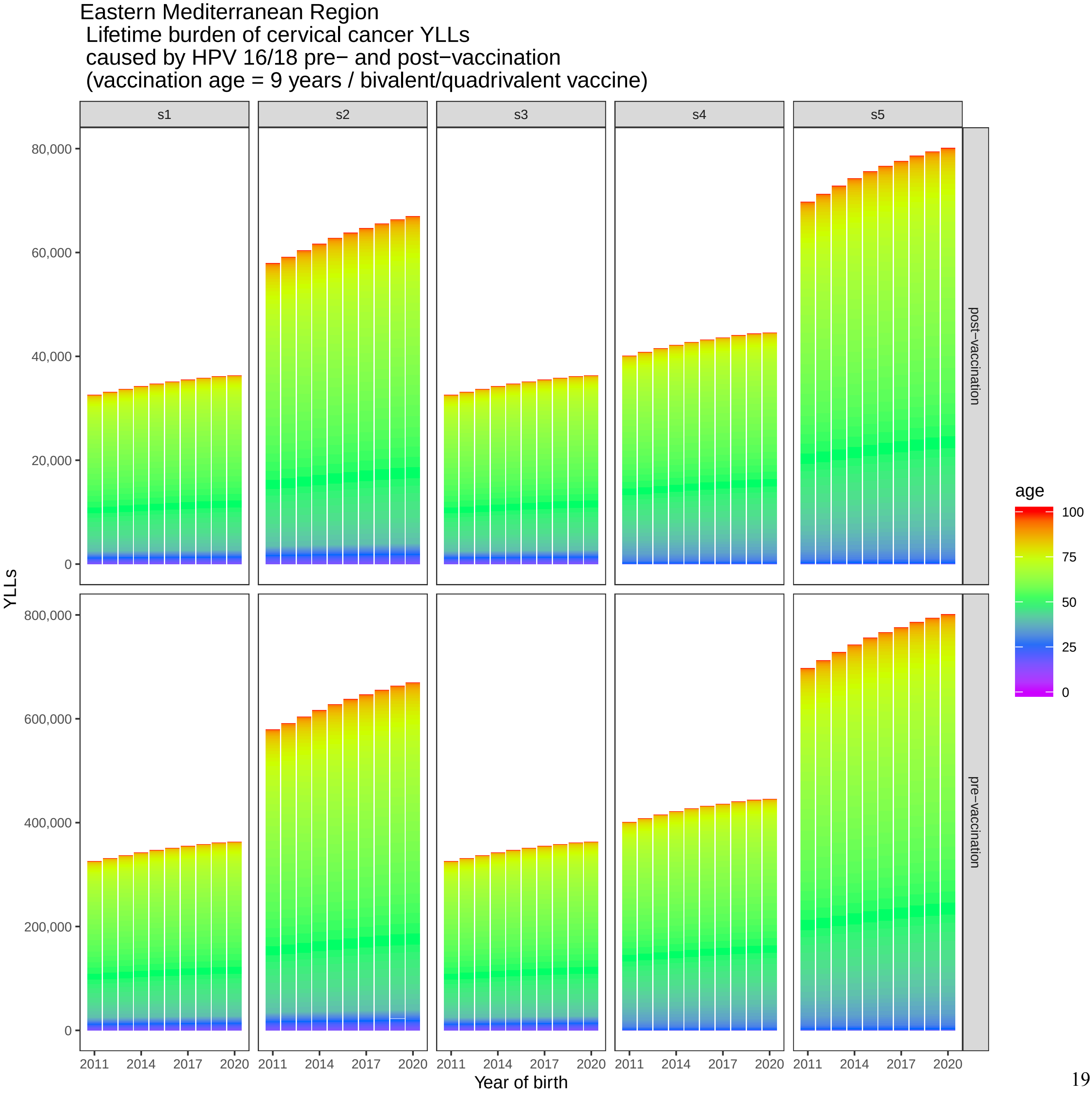

Eastern Mediterranean Region
Lifetime burden of cervical cancer YLLs
caused by HPV 16/18 pre− and post−vaccination
(vaccination age = 9 years / bivalent/quadrivalent vaccine)
s1
s2
s3
s4
s5
post−vaccination
pre−vaccination
YLLs
80,000
60,000
40,000
20,000
0
800,000
600,000
400,000
200,000
0
2011
2014
2017
2020
Year of birth
age
100
75
50
25
0

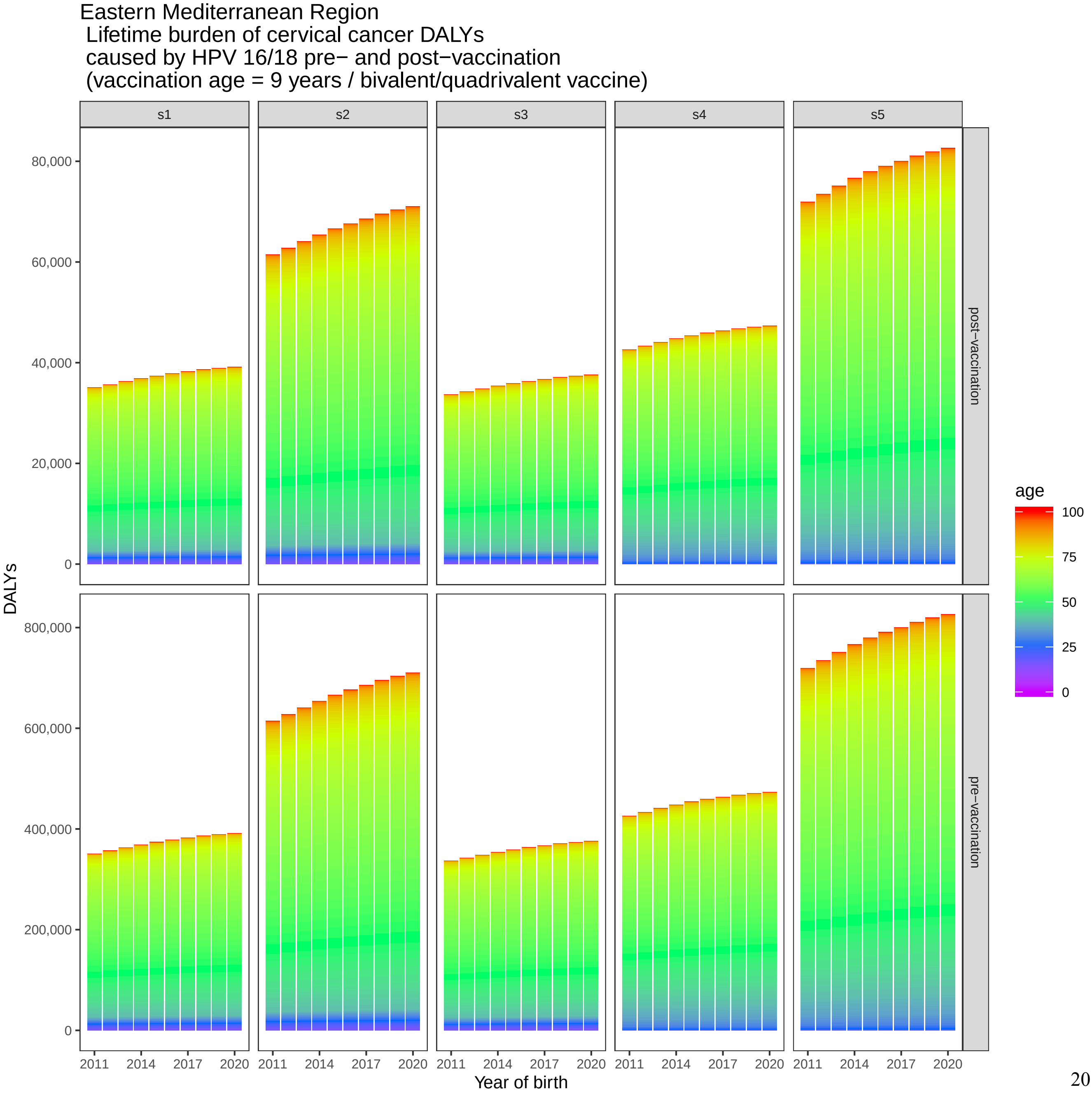
Eastern Mediterranean Region
Lifetime burden of cervical cancer DALYs
caused by HPV 16/18 pre− and post−vaccination
(vaccination age = 9 years / bivalent/quadrivalent vaccine)
s1
s2
s3
s4
s5
post−vaccination
pre−vaccination
DALYs
Year of birth
age
0
25
50
75
100
80,000
60,000
40,000
20,000
800,000
600,000
400,000
200,000
2011
2014
2017
2020

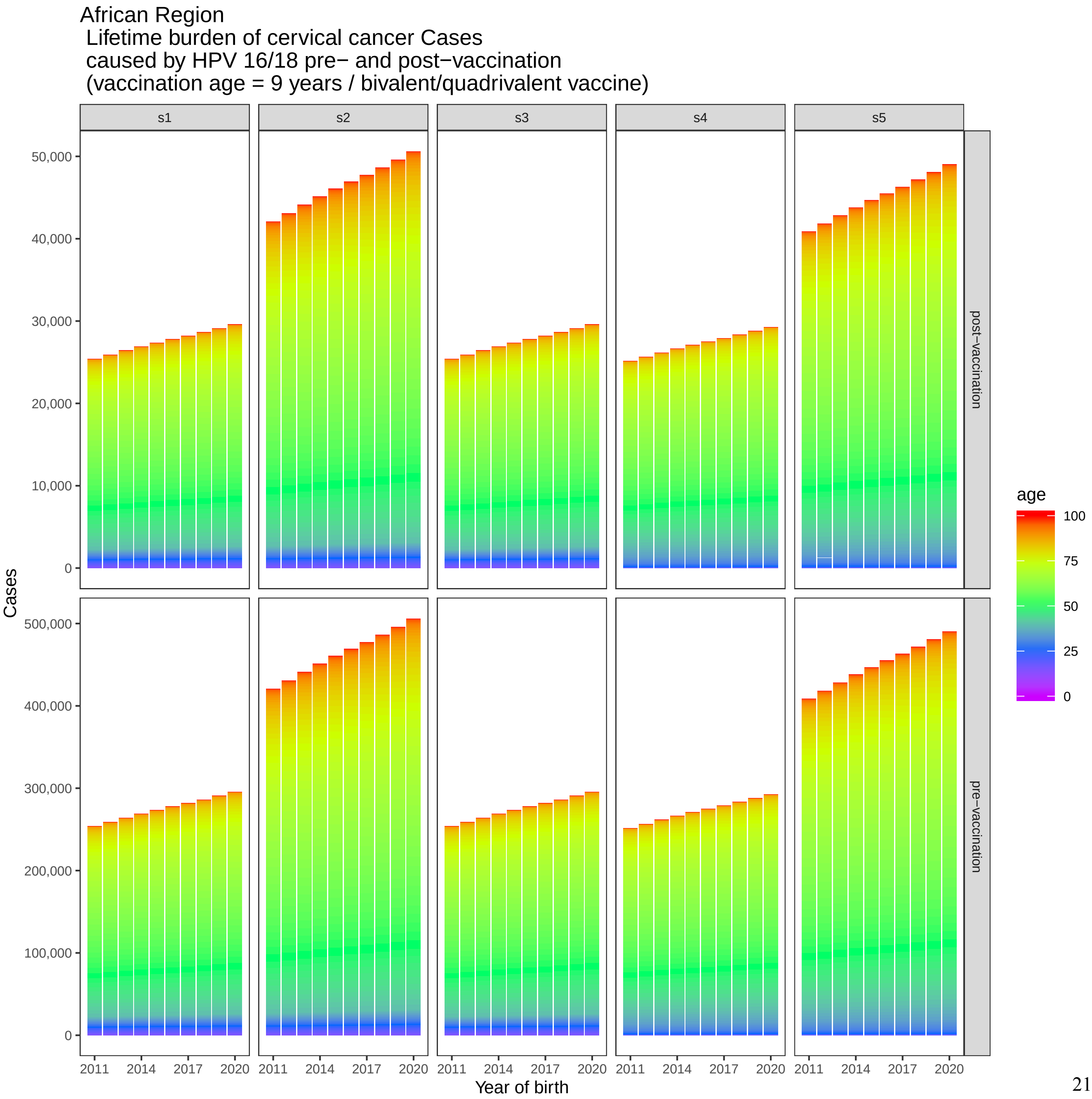

African Region
Lifetime burden of cervical cancer Cases
caused by HPV 16/18 pre− and post−vaccination
(vaccination age = 9 years / bivalent/quadrivalent vaccine)
s1
s2
s3
s4
s5
post−vaccination
pre−vaccination
Cases
50,000
40,000
30,000
20,000
10,000
0
500,000
400,000
300,000
200,000
100,000
0
2011
2014
2017
2020
Year of birth
age
100
75
50
25
0

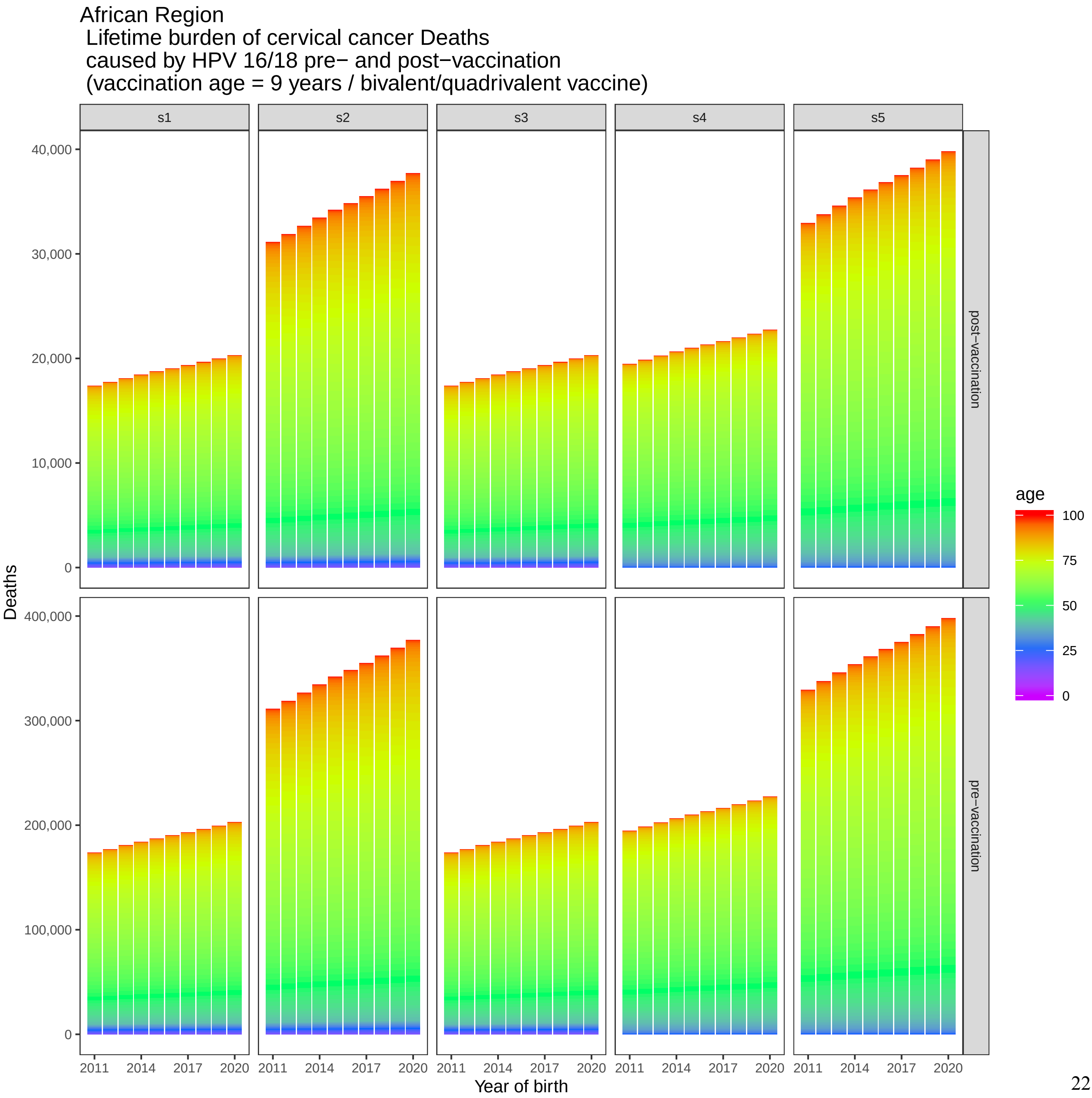

African Region
Lifetime burden of cervical cancer Deaths
caused by HPV 16/18 pre− and post−vaccination
(vaccination age = 9 years / bivalent/quadrivalent vaccine)
s1
s2
s3
s4
s5
post−vaccination
pre−vaccination
Deaths
Year of birth
0
10,000
20,000
30,000
40,000
100,000
200,000
300,000
400,000
2011
2014
2017
2020
age
0
25
50
75
100

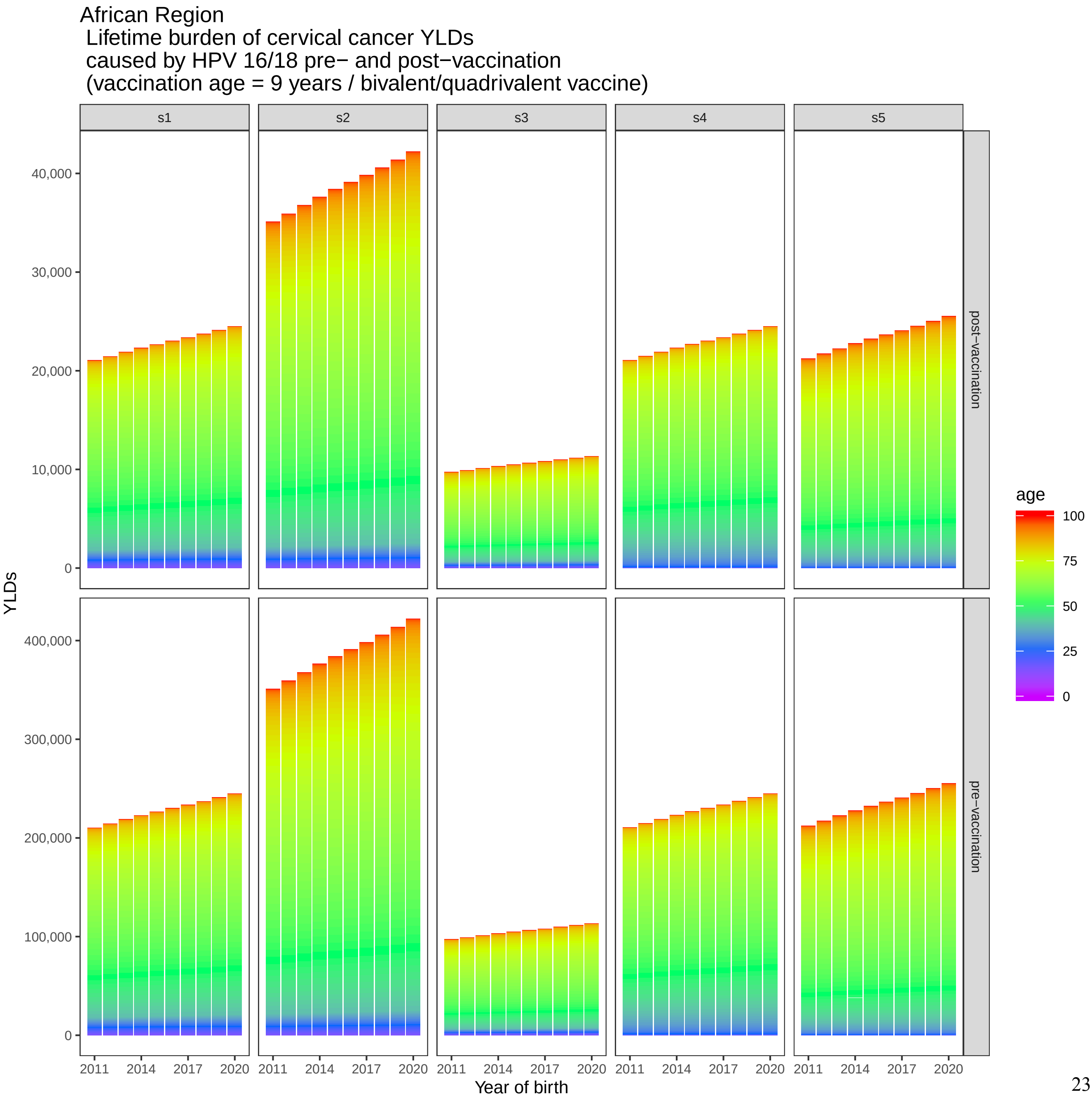

African Region
Lifetime burden of cervical cancer YLDs
caused by HPV 16/18 pre− and post−vaccination
(vaccination age = 9 years / bivalent/quadrivalent vaccine)
s1
s2
s3
s4
s5
post−vaccination
pre−vaccination
YLDs
Year of birth
age
0
25
50
75
100
0
10,000
20,000
30,000
40,000
100,000
200,000
300,000
400,000
2011
2014
2017
2020

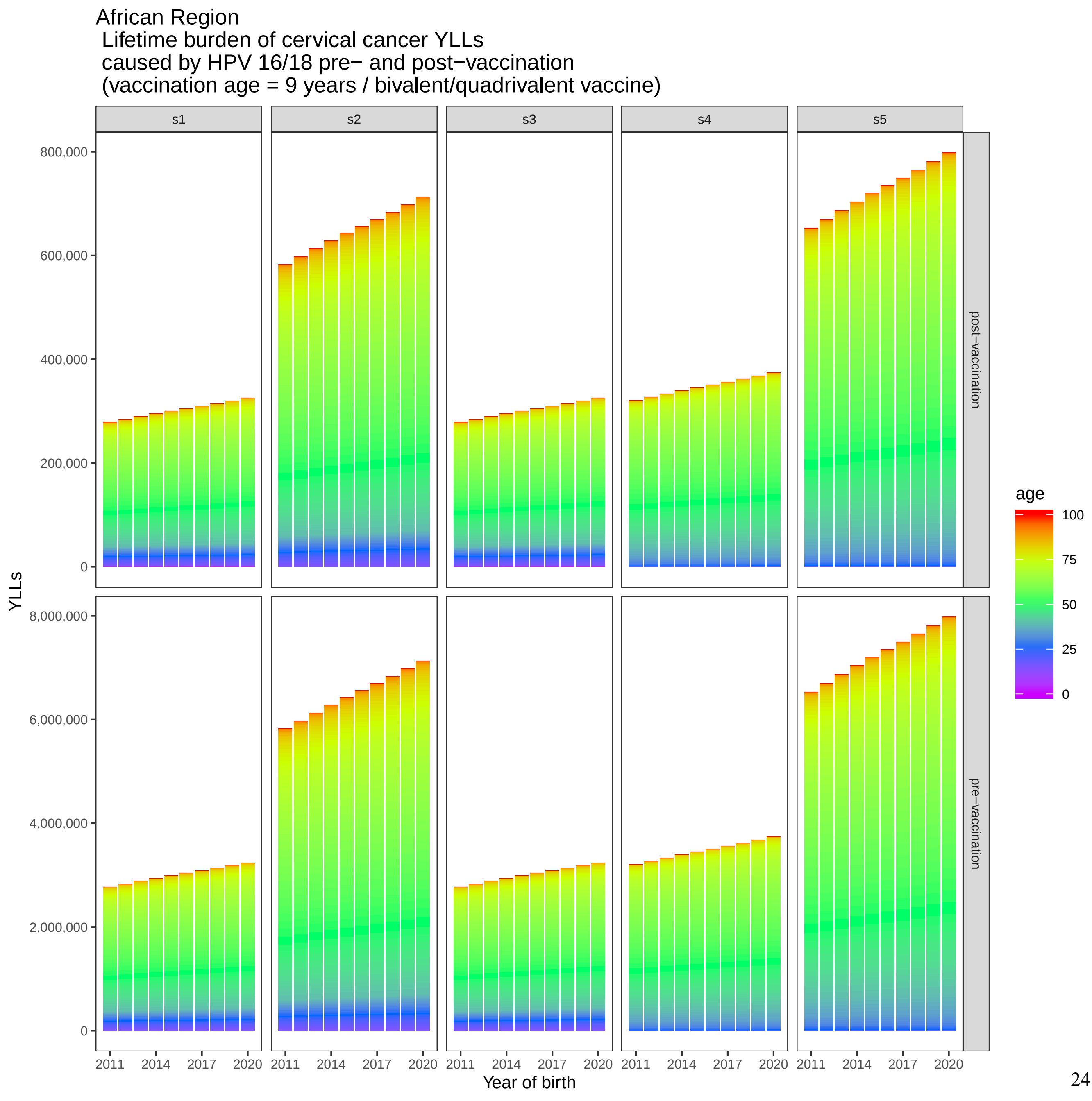
African Region
Lifetime burden of cervical cancer YLLs
caused by HPV 16/18 pre− and post−vaccination
(vaccination age = 9 years / bivalent/quadrivalent vaccine)
s1
s2
s3
s4
s5
post−vaccination
pre−vaccination
YLLs
800,000
600,000
400,000
200,000
0
8,000,000
6,000,000
4,000,000
2,000,000
0
2011
2014
2017
2020
Year of birth
age
100
75
50
25
0

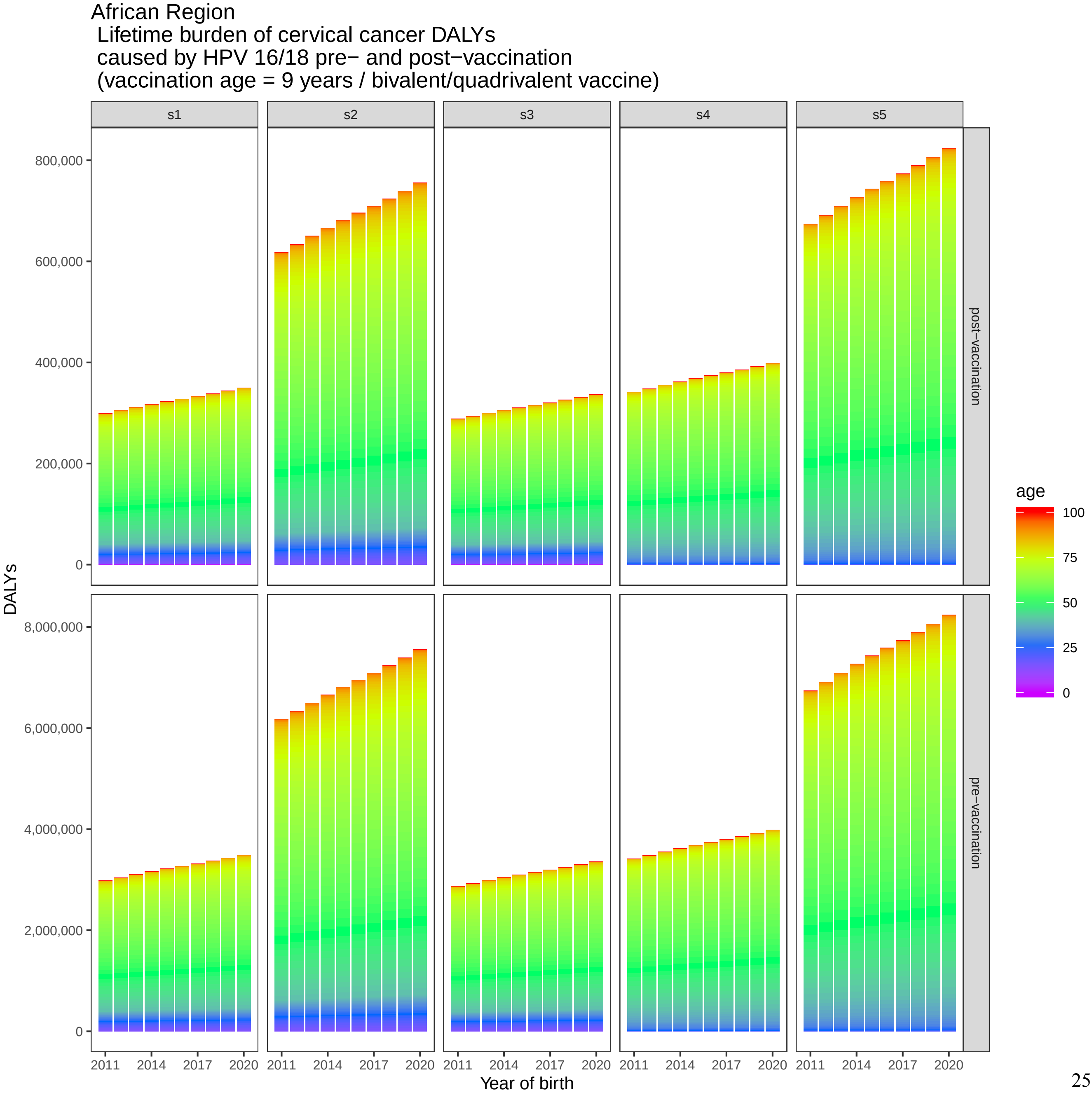
African Region
Lifetime burden of cervical cancer DALYs
caused by HPV 16/18 pre− and post−vaccination
(vaccination age = 9 years / bivalent/quadrivalent vaccine)
s1
s2
s3
s4
s5
post−vaccination
pre−vaccination
DALYs
800,000
600,000
400,000
200,000
0
8,000,000
6,000,000
4,000,000
2,000,000
0
2011
2014
2017
2020
Year of birth
age
100
75
50
25
0

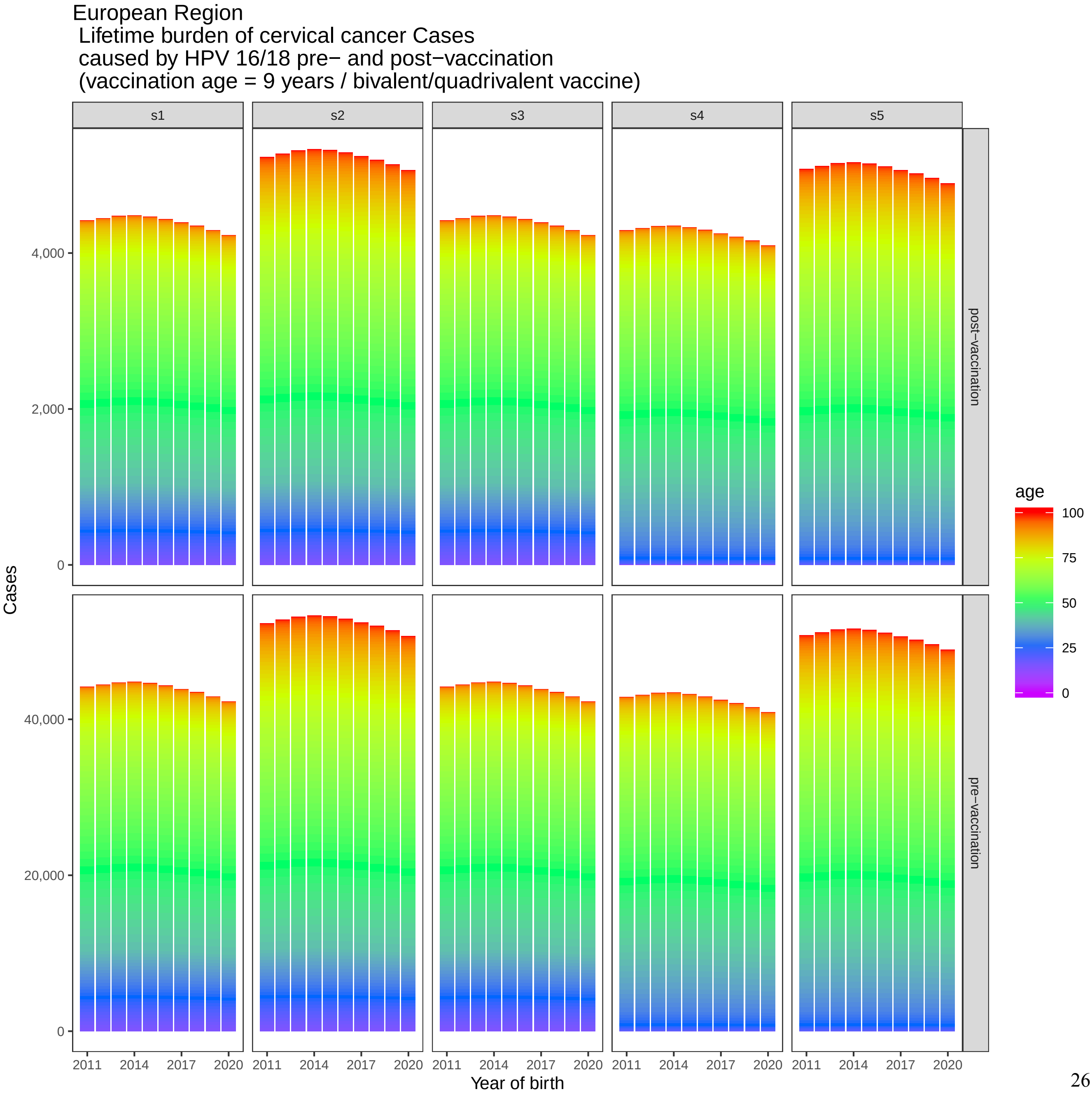

European Region
Lifetime burden of cervical cancer Cases
caused by HPV 16/18 pre− and post−vaccination
(vaccination age = 9 years / bivalent/quadrivalent vaccine)
s1
s2
s3
s4
s5
post−vaccination
pre−vaccination
Cases
4,000
2,000
0
40,000
20,000
0
2011
2014
2017
2020
Year of birth
age
100
75
50
25
0

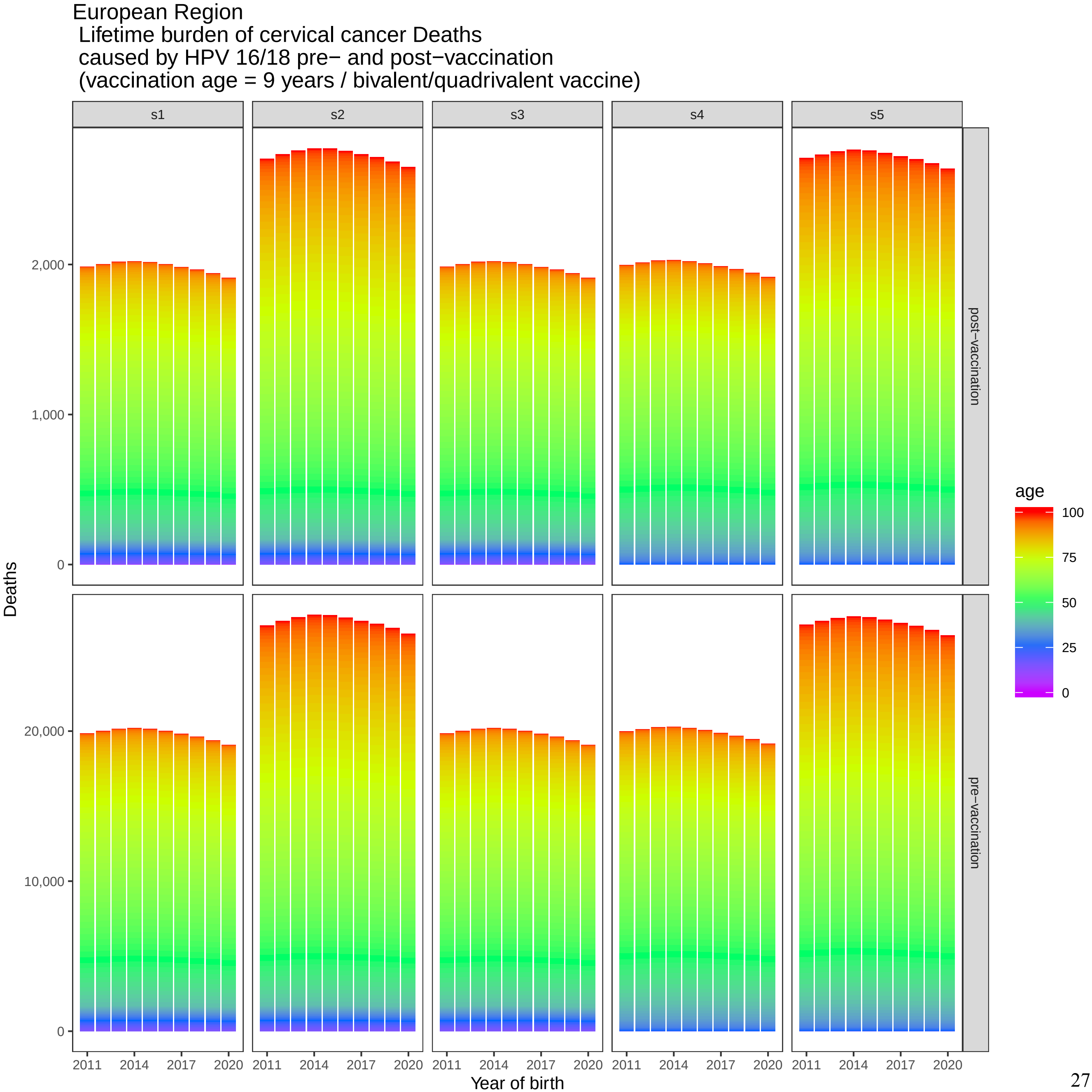
European Region
Lifetime burden of cervical cancer Deaths
caused by HPV 16/18 pre− and post−vaccination
(vaccination age = 9 years / bivalent/quadrivalent vaccine)
s1
s2
s3
s4
s5
post−vaccination
pre−vaccination
Deaths
0
1,000
2,000
10,000
20,000
2011
2014
2017
2020
Year of birth
age
0
25
50
75
100

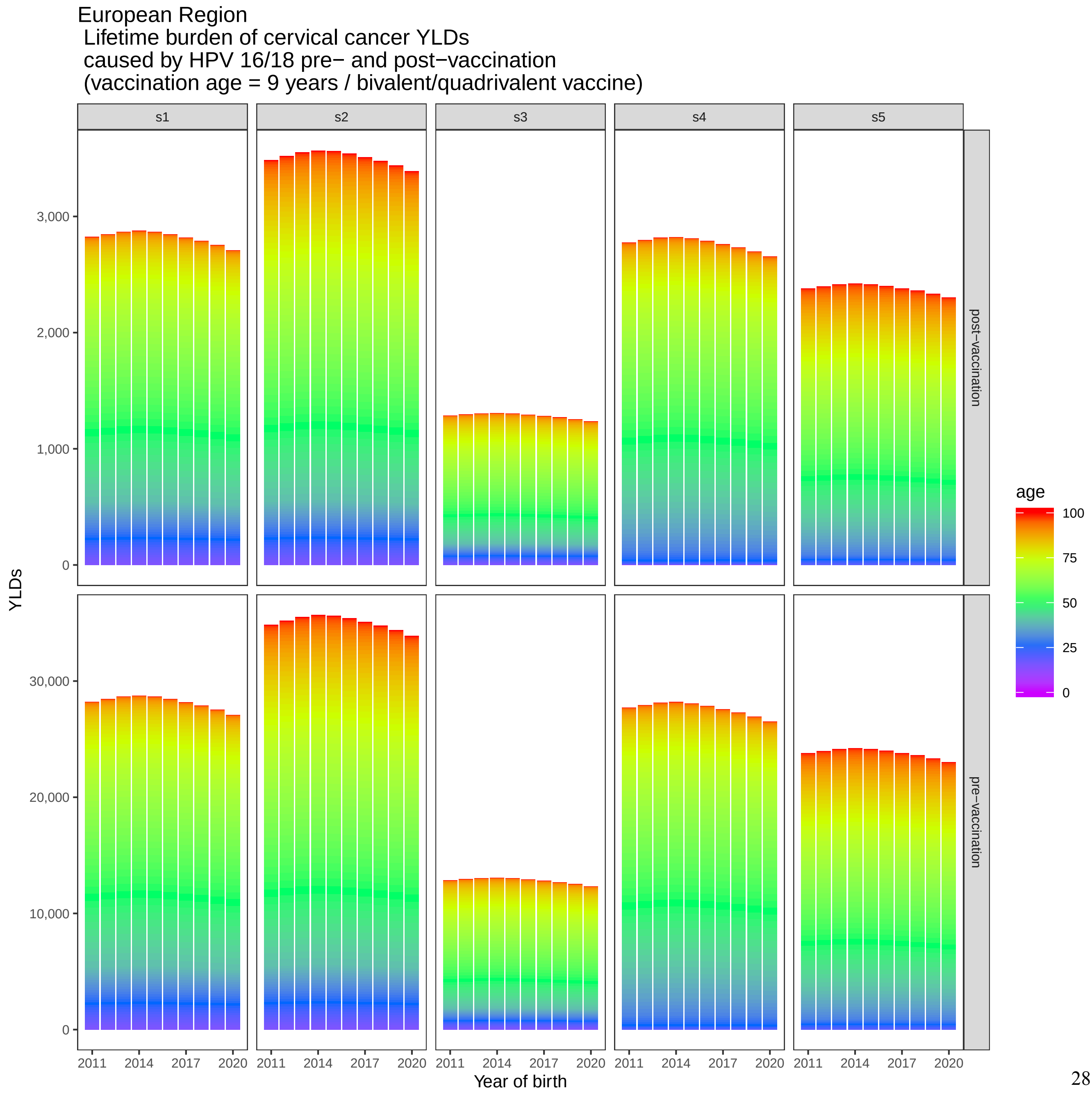
European Region
Lifetime burden of cervical cancer YLDs
caused by HPV 16/18 pre− and post−vaccination
(vaccination age = 9 years / bivalent/quadrivalent vaccine)
s1
s2
s3
s4
s5
post−vaccination
pre−vaccination
YLDs
Year of birth
0
1,000
2,000
3,000
10,000
20,000
30,000
2011
2014
2017
2020
age
100
75
50
25
0

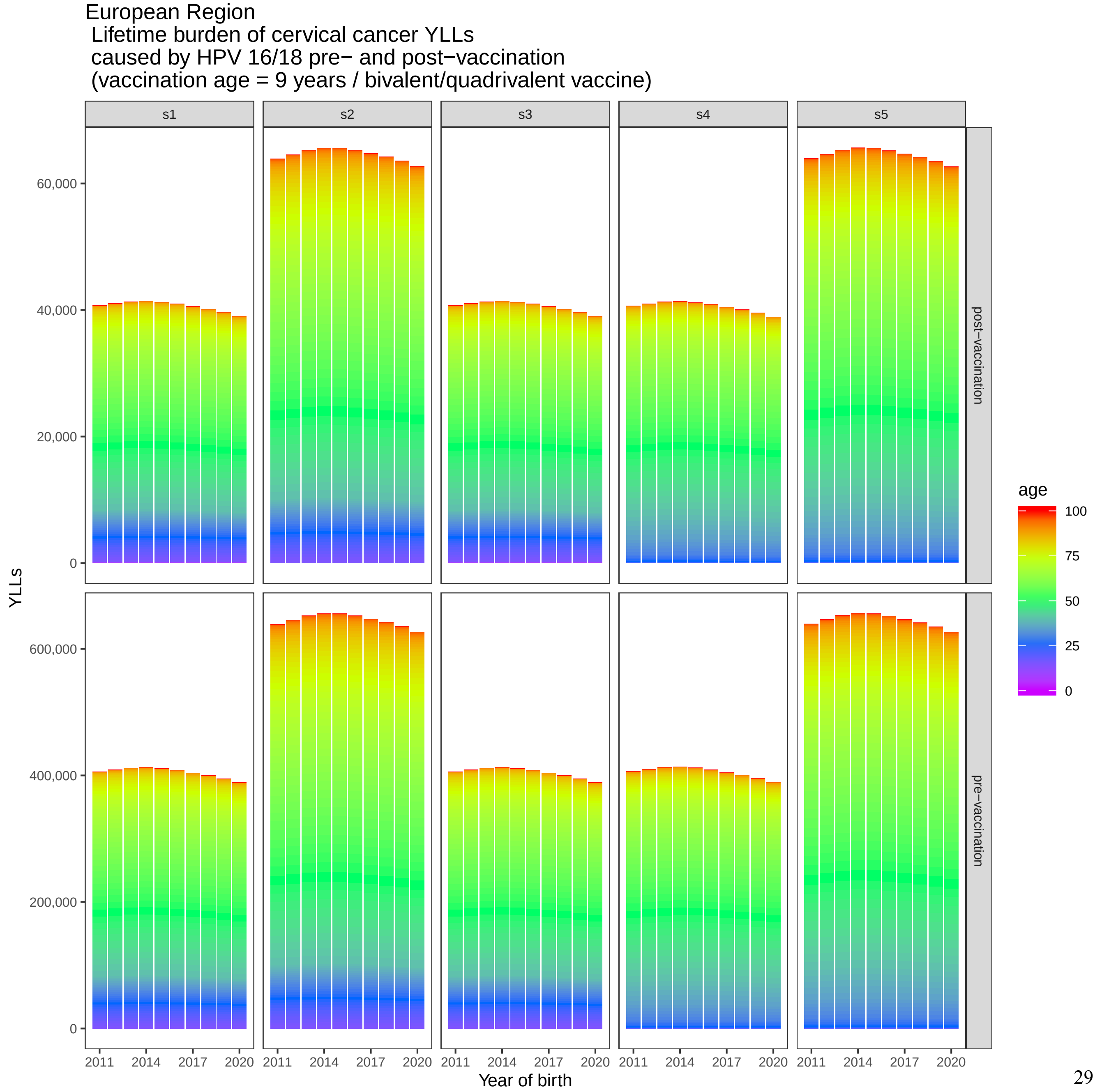
European Region
Lifetime burden of cervical cancer YLLs
caused by HPV 16/18 pre− and post−vaccination
(vaccination age = 9 years / bivalent/quadrivalent vaccine)
s1
s2
s3
s4
s5
post−vaccination
pre−vaccination
YLLs
60,000
40,000
20,000
0
600,000
400,000
200,000
0
2011
2014
2017
2020
Year of birth
age
100
75
50
25
0

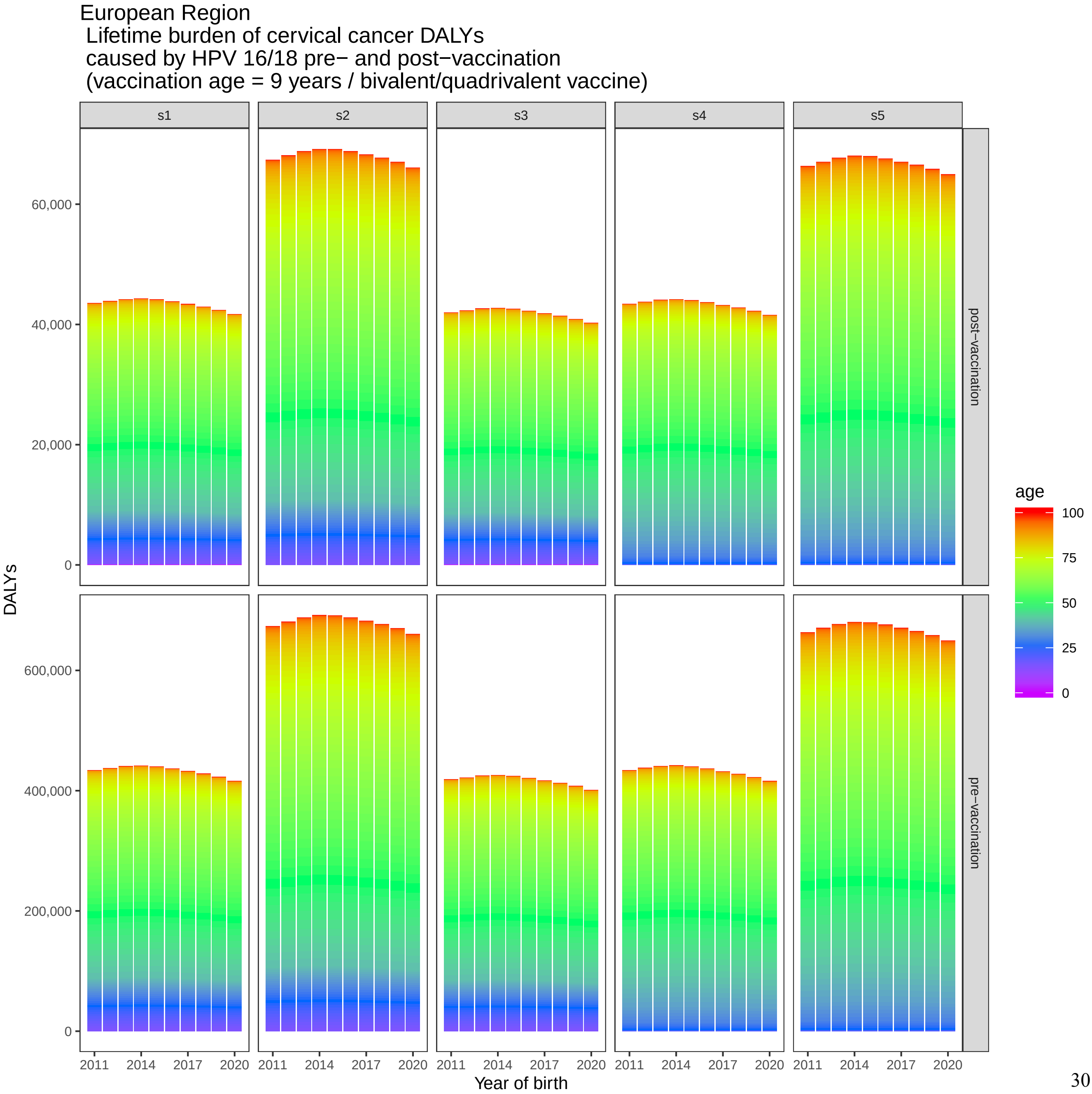

European Region
Lifetime burden of cervical cancer DALYs
caused by HPV 16/18 pre− and post−vaccination
(vaccination age = 9 years / bivalent/quadrivalent vaccine)
s1
s2
s3
s4
s5
post−vaccination
pre−vaccination
60,000
40,000
20,000
0
600,000
400,000
200,000
0
DALYs
2011
2014
2017
2020
Year of birth
age
100
75
50
25
0

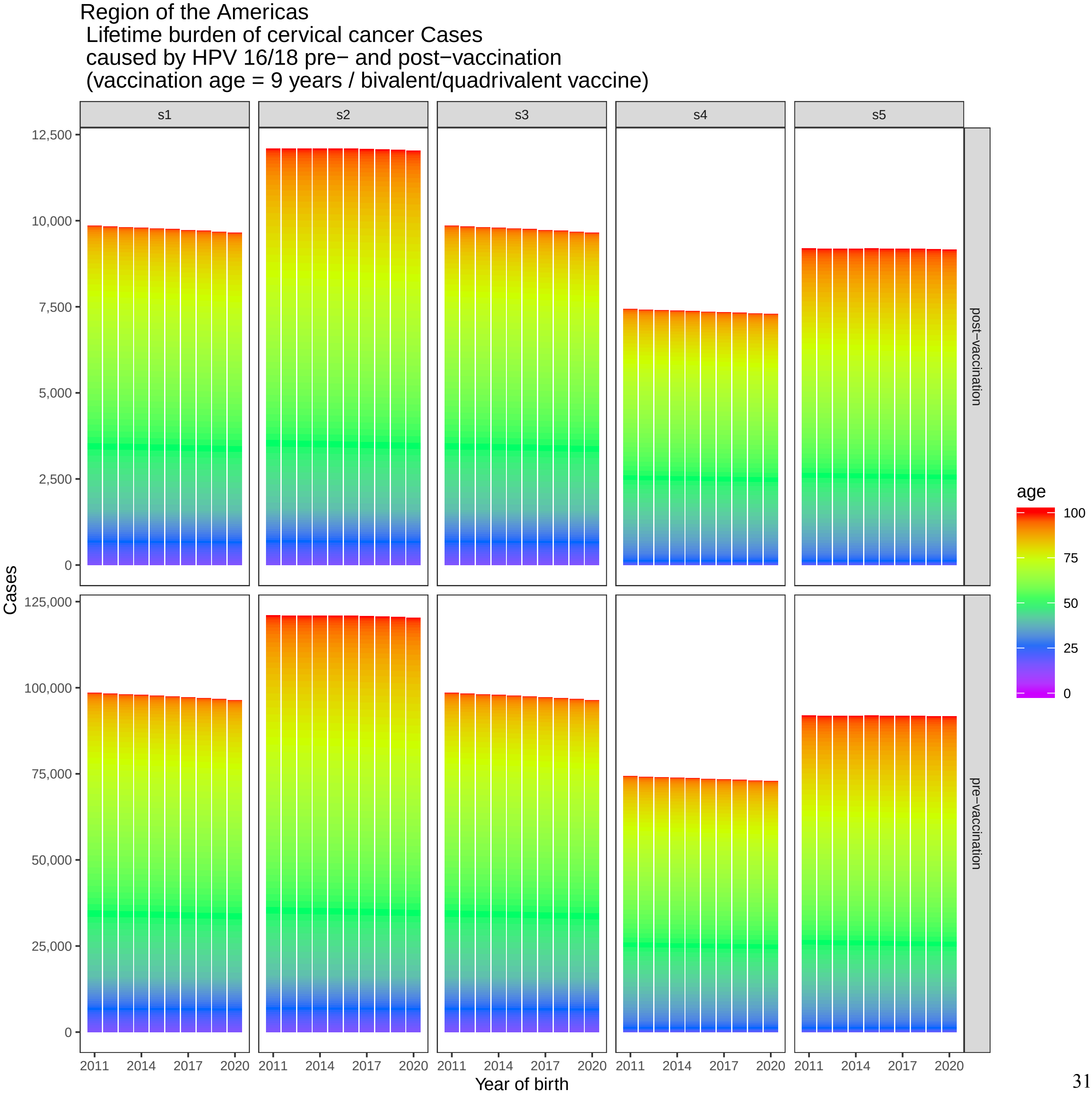

Region of the Americas
Lifetime burden of cervical cancer Cases
caused by HPV 16/18 pre− and post−vaccination
(vaccination age = 9 years / bivalent/quadrivalent vaccine)
s1
s2
s3
s4
s5
post−vaccination
pre−vaccination
Cases
0
2,500
5,000
7,500
10,000
12,500
25,000
50,000
75,000
100,000
125,000
2011
2014
2017
2020
Year of birth
age
0
25
50
75
100

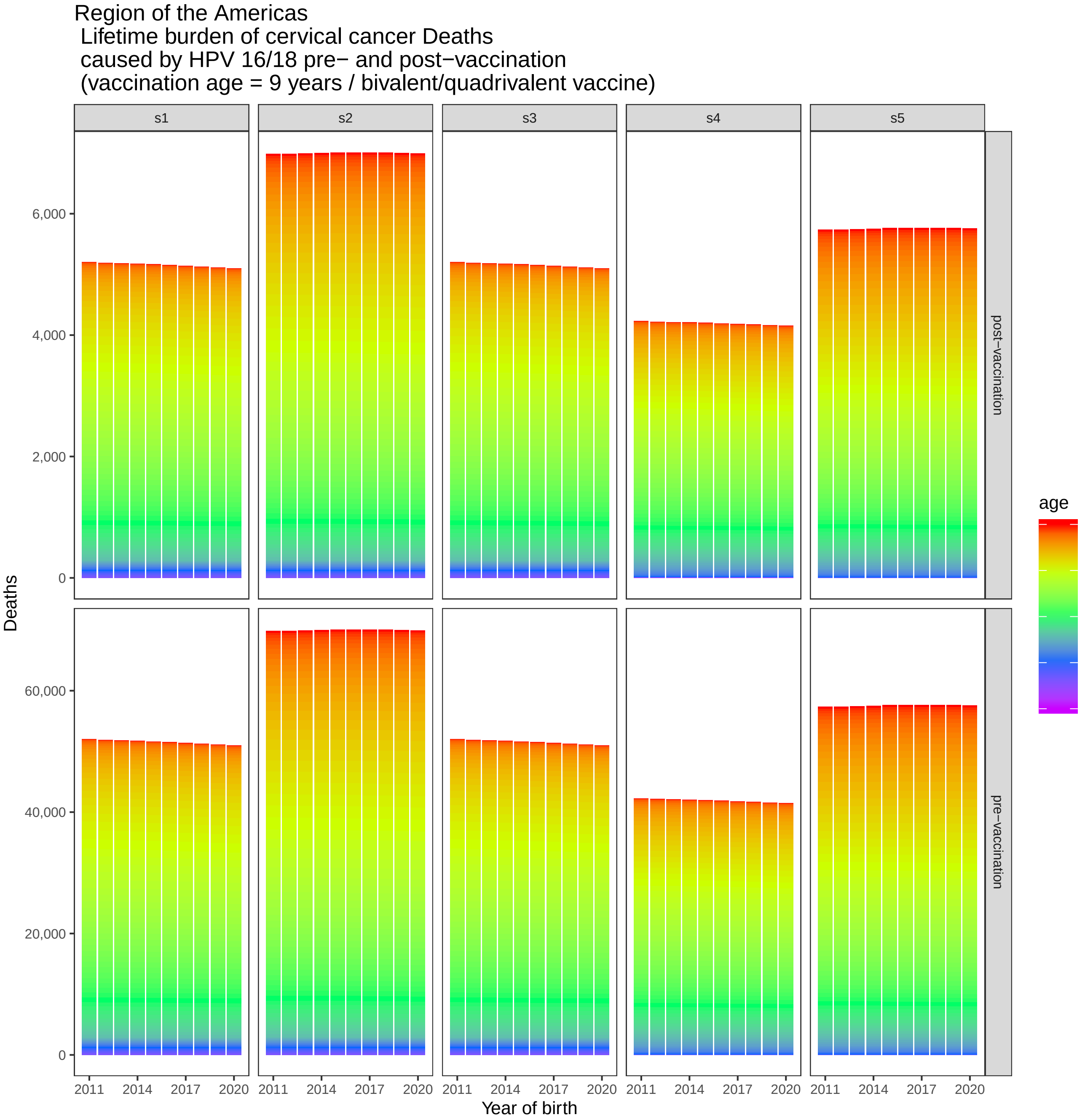
Region of the Americas
Lifetime burden of cervical cancer Deaths
caused by HPV 16/18 pre− and post−vaccination
(vaccination age = 9 years / bivalent/quadrivalent vaccine)
s1
s2
s3
s4
s5
post−vaccination
pre−vaccination
Deaths
6,000
4,000
2,000
0
60,000
40,000
20,000
0
2011
2014
2017
2020
Year of birth
age
100
75
50
25
0

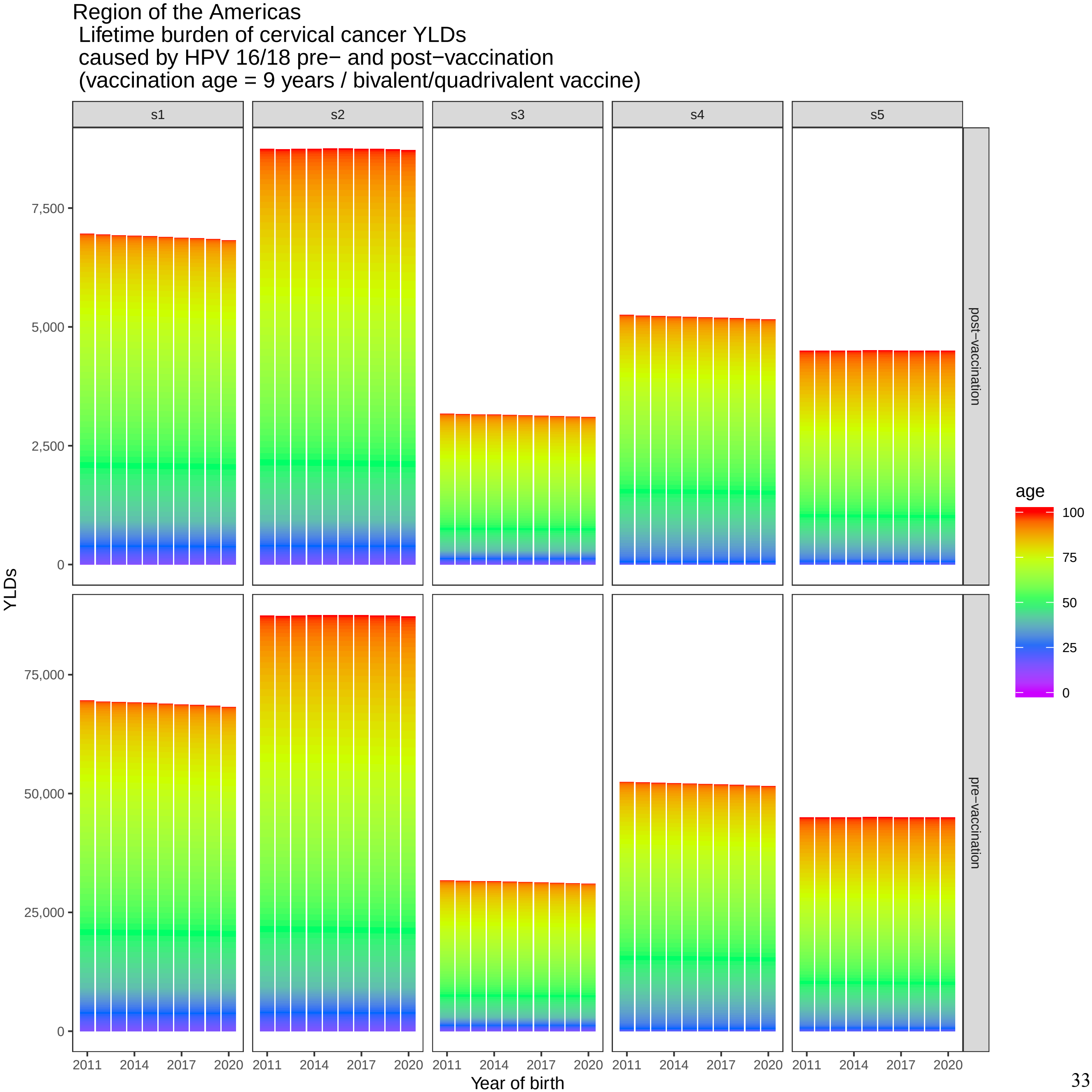

Region of the Americas
Lifetime burden of cervical cancer YLDs
caused by HPV 16/18 pre− and post−vaccination
(vaccination age = 9 years / bivalent/quadrivalent vaccine)
s1
s2
s3
s4
s5
post−vaccination
pre−vaccination
YLDs
0
2,500
5,000
7,500
25,000
50,000
75,000
2011
2014
2017
2020
Year of birth
age
100
75
50
25
0

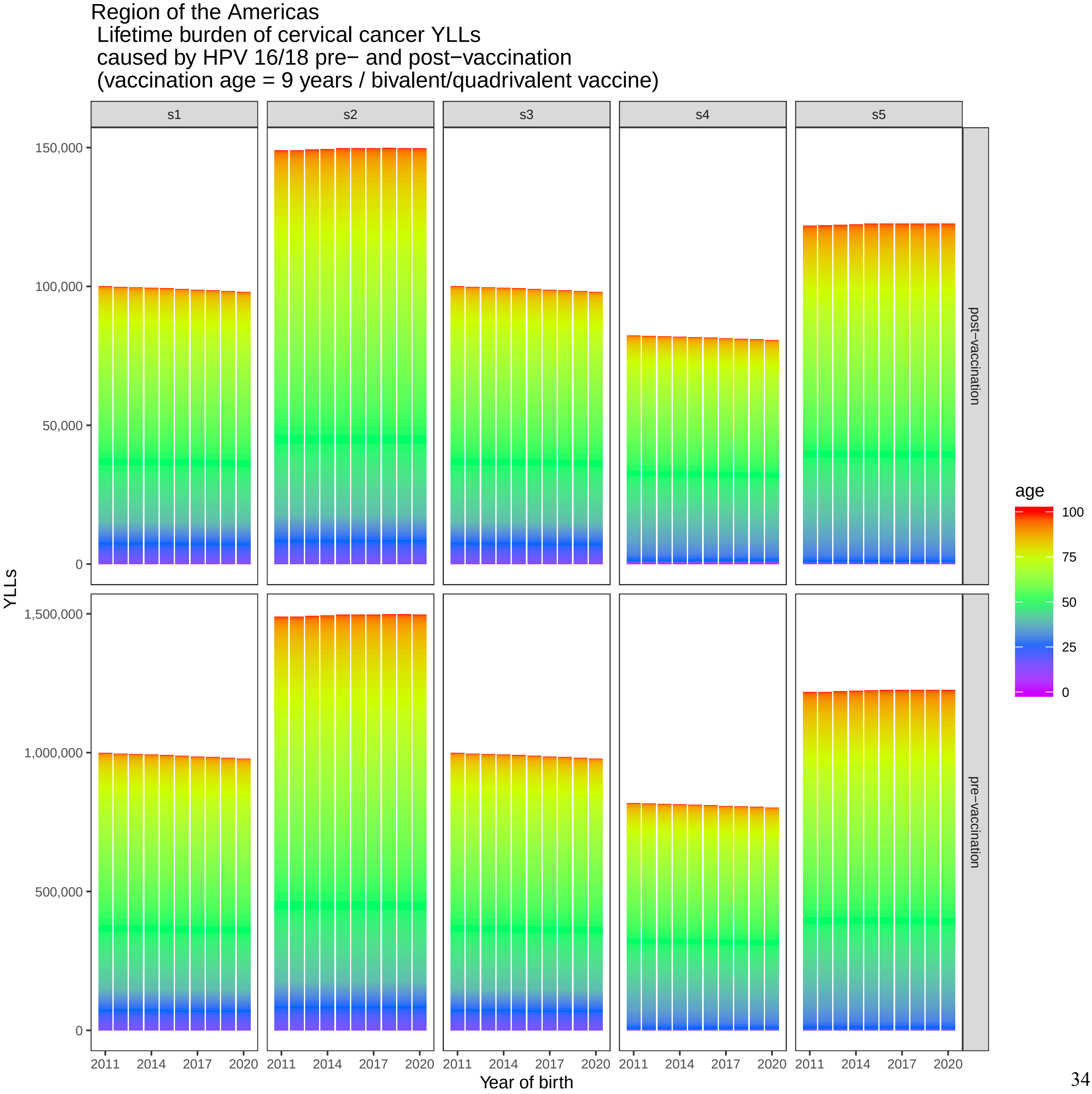
Region of the Americas
Lifetime burden of cervical cancer YLLs
caused by HPV 16/18 pre− and post−vaccination
(vaccination age = 9 years / bivalent/quadrivalent vaccine)
s1
s2
s3
s4
s5
post−vaccination
pre−vaccination
YLLs
150,000
100,000
50,000
0
1,500,000
1,000,000
500,000
2011
2014
2017
2020
Year of birth
age
100
75
50
25

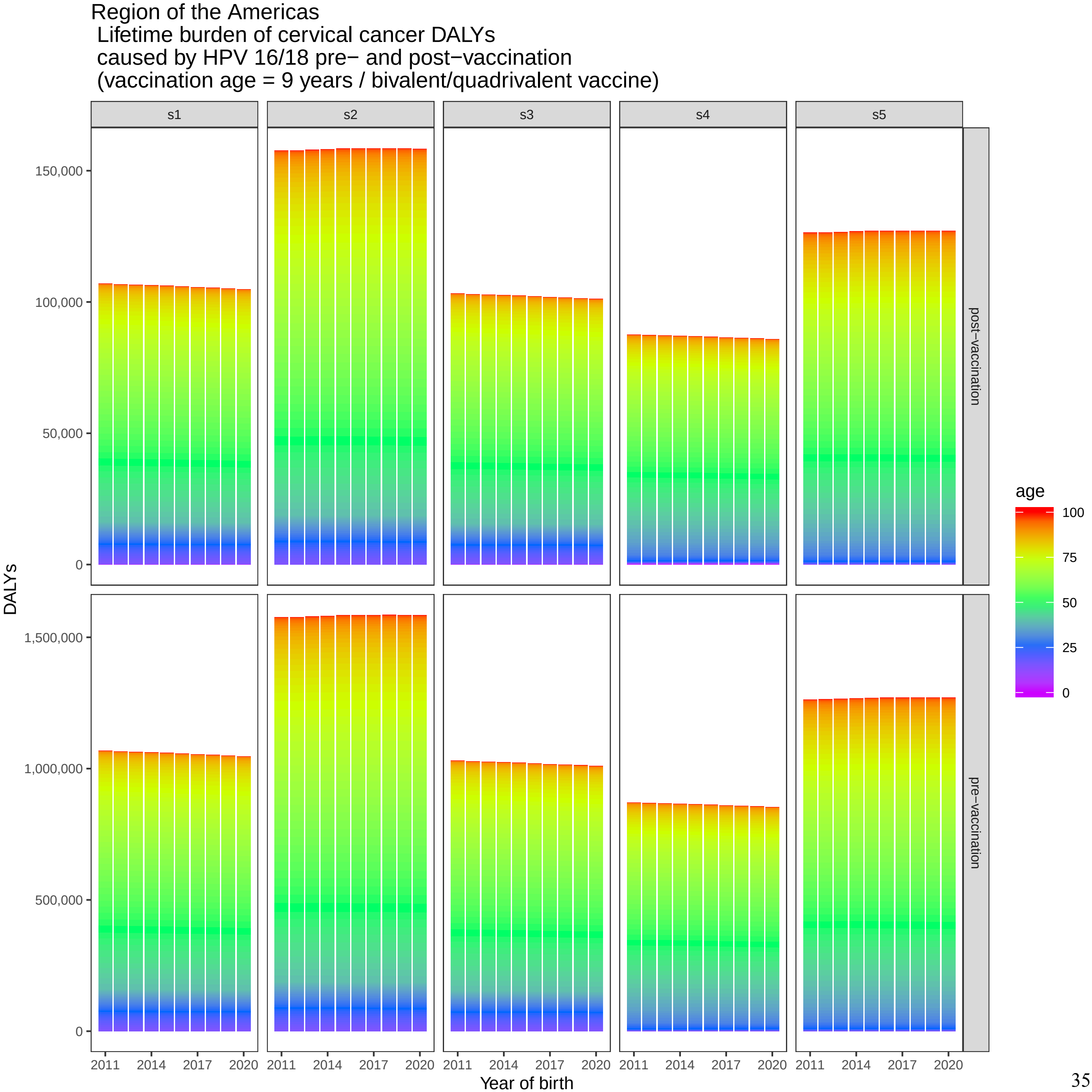
Region of the Americas
Lifetime burden of cervical cancer DALYs
caused by HPV 16/18 pre− and post−vaccination
(vaccination age = 9 years / bivalent/quadrivalent vaccine)
s1
s2
s3
s4
s5
post−vaccination
pre−vaccination
DALYs
150,000
100,000
50,000
0
1,500,000
1,000,000
500,000
0
2011
2014
2017
2020
Year of birth
age
100
75
50
25
0

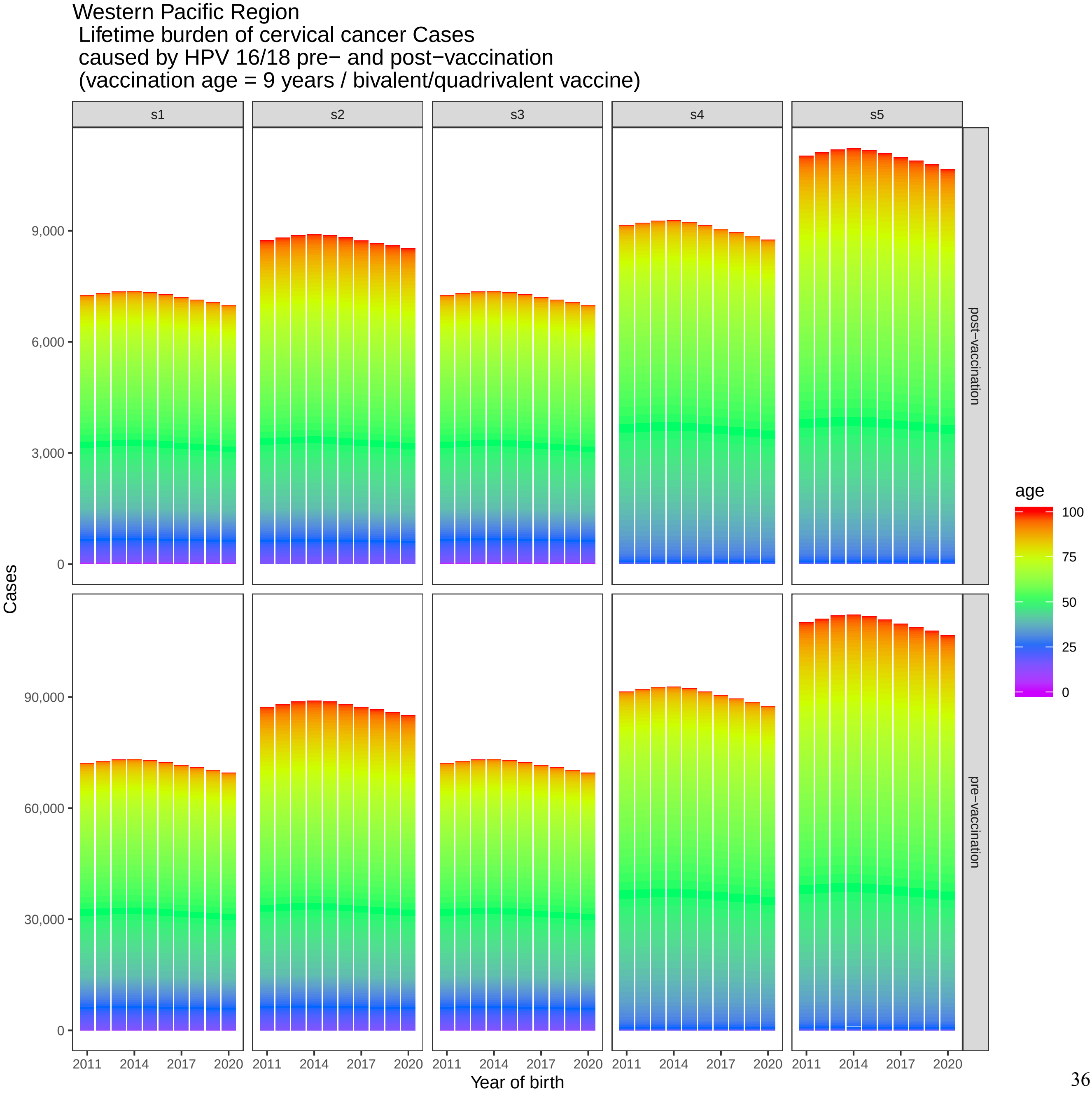

Western Pacific Region
Lifetime burden of cervical cancer Cases
caused by HPV 16/18 pre− and post−vaccination
(vaccination age = 9 years / bivalent/quadrivalent vaccine)
s1
s2
s3
s4
s5
post−vaccination
pre−vaccination
Cases
9,000
6,000
3,000
0
90,000
60,000
30,000
0
2011
2014
2017
2020
Year of birth
age
100
75
50
25
0

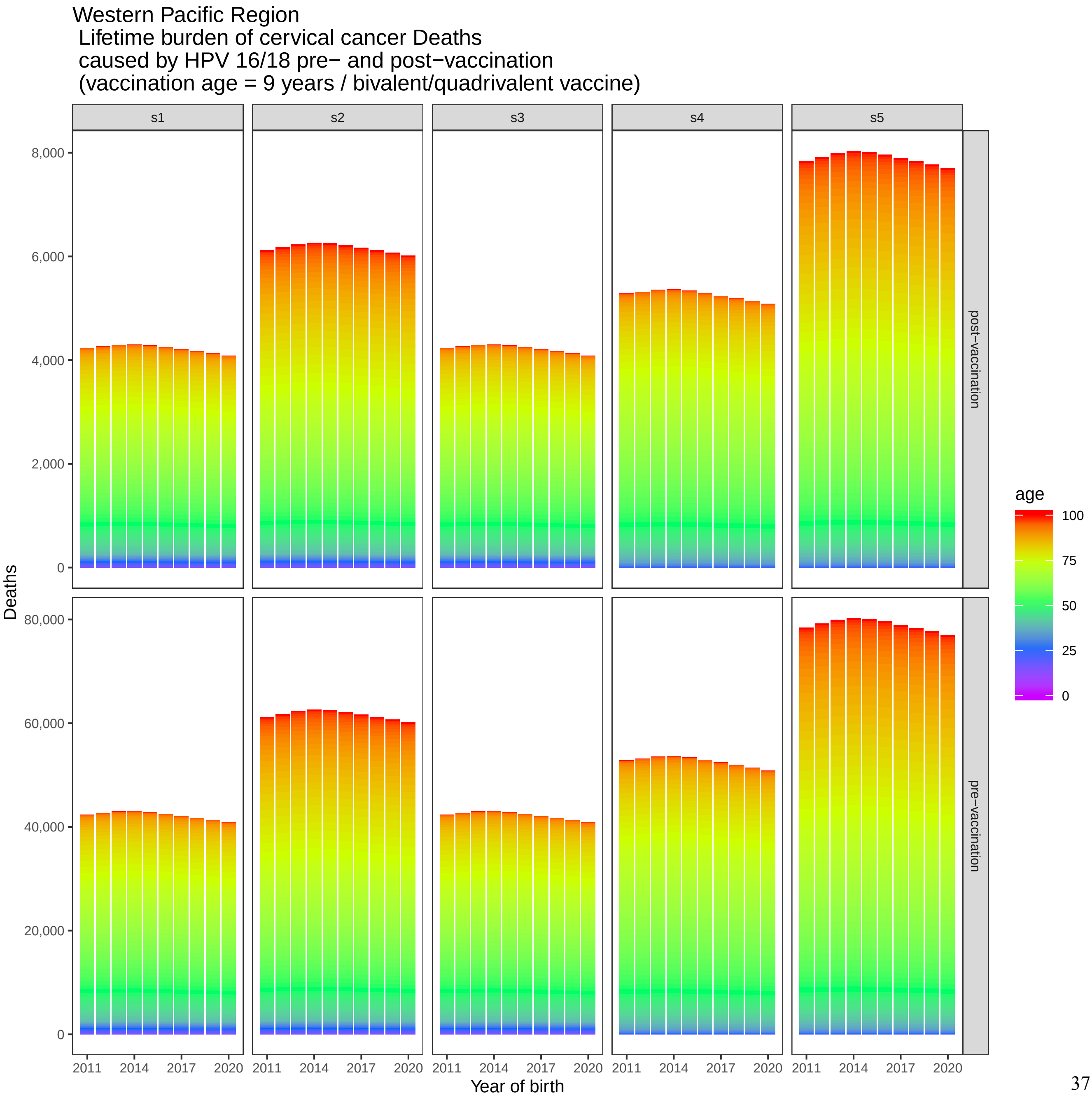
Western Pacific Region
Lifetime burden of cervical cancer Deaths
caused by HPV 16/18 pre− and post−vaccination
(vaccination age = 9 years / bivalent/quadrivalent vaccine)
s1
s2
s3
s4
s5
post−vaccination
pre−vaccination
Deaths
0
2,000
4,000
6,000
8,000
20,000
40,000
60,000
80,000
2011
2014
2017
2020
Year of birth
age
0
25
50
75
100

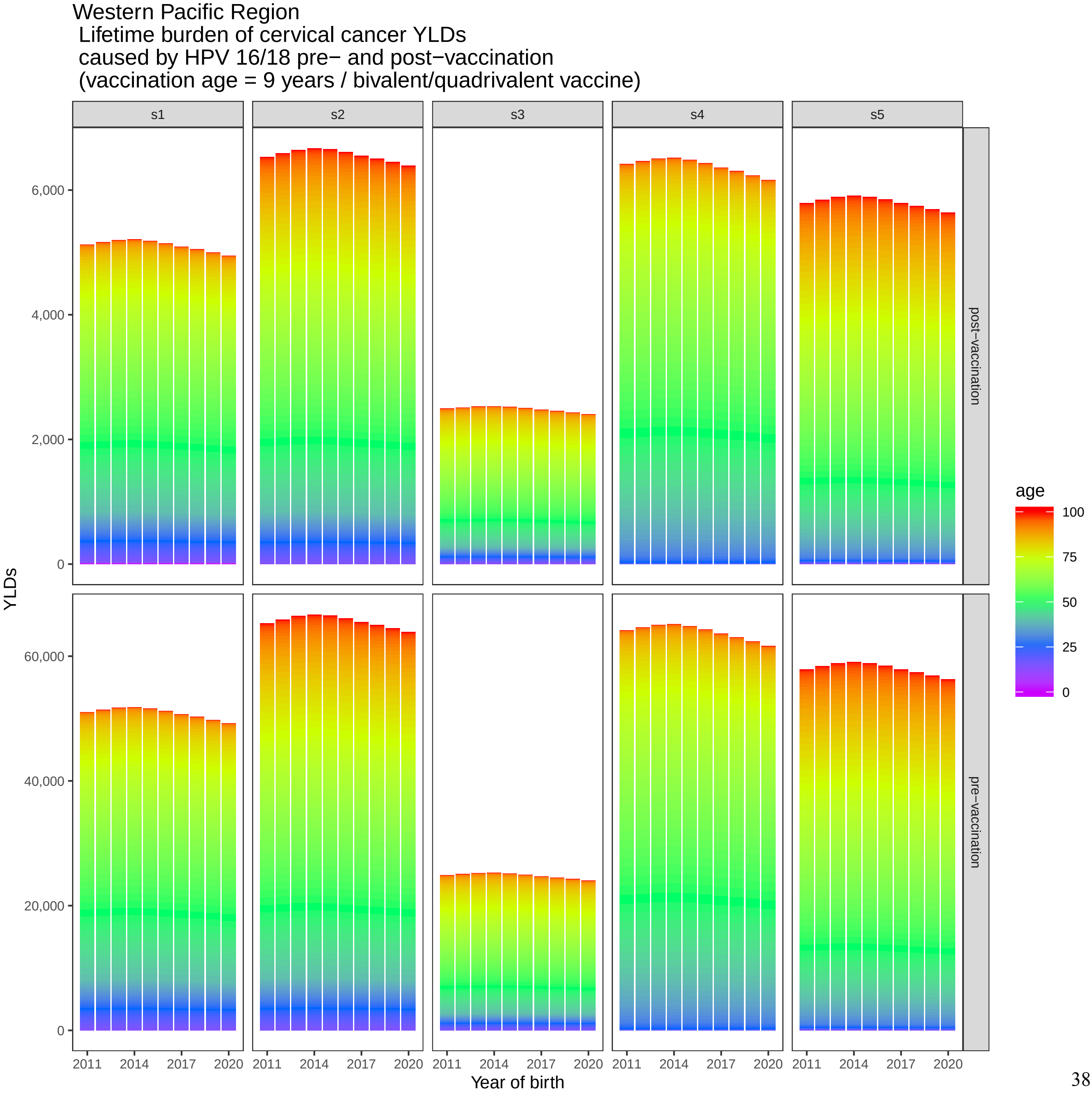
Western Pacific Region
Lifetime burden of cervical cancer YLDs
caused by HPV 16/18 pre− and post−vaccination
(vaccination age = 9 years / bivalent/quadrivalent vaccine)
s1
s2
s3
s4
s5
post−vaccination
pre−vaccination
YLDs
0
2,000
4,000
6,000
20,000
40,000
60,000
2011
2014
2017
2020
Year of birth
age
0
25
50
75
100

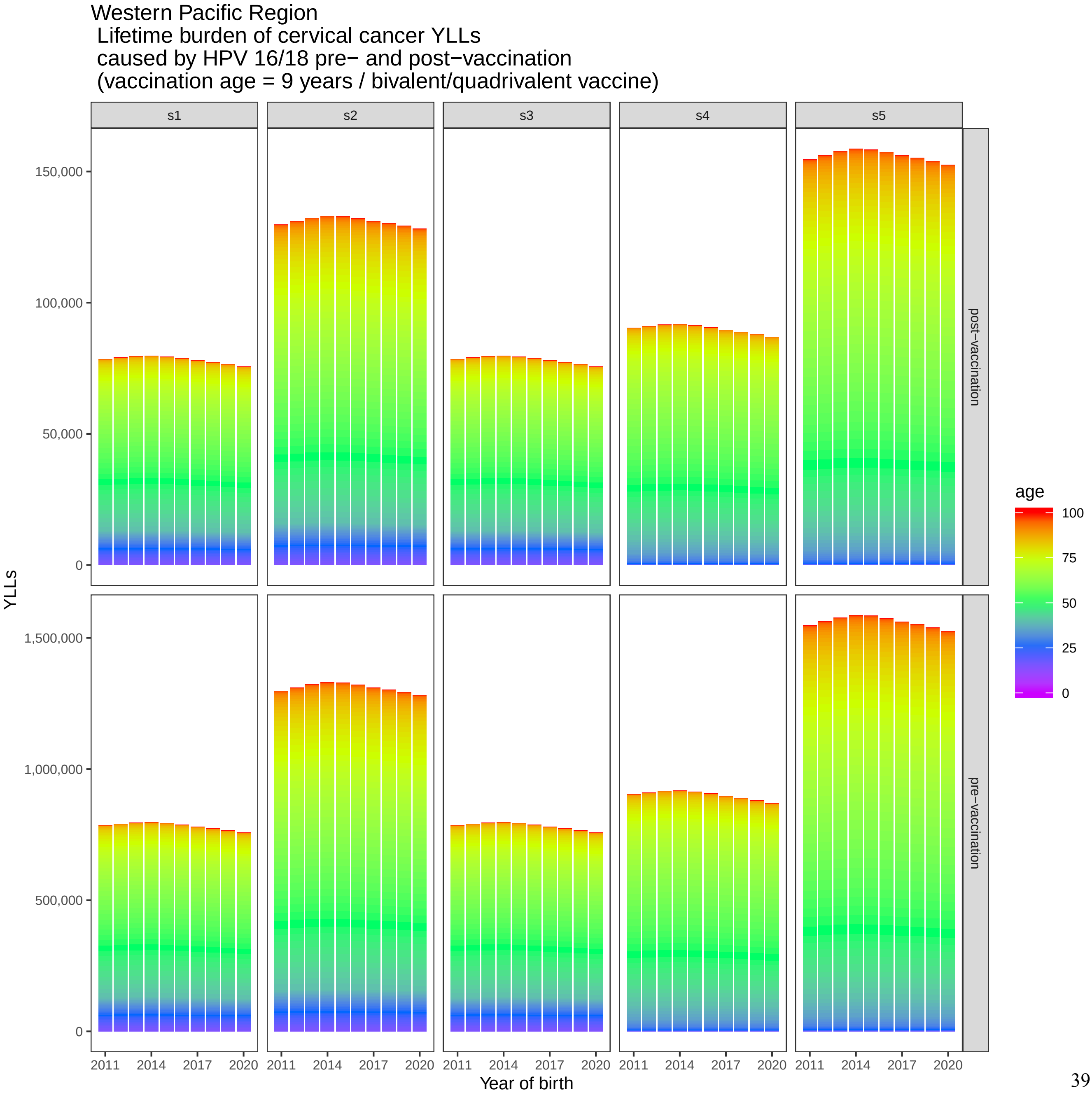

Western Pacific Region
Lifetime burden of cervical cancer YLLs
caused by HPV 16/18 pre− and post−vaccination
(vaccination age = 9 years / bivalent/quadrivalent vaccine)
s1
s2
s3
s4
s5
post−vaccination
pre−vaccination
150,000
100,000
50,000
0
1,500,000
1,000,000
500,000
0
YLLs
2011
2014
2017
2020
Year of birth
age
100
75
50
25
0

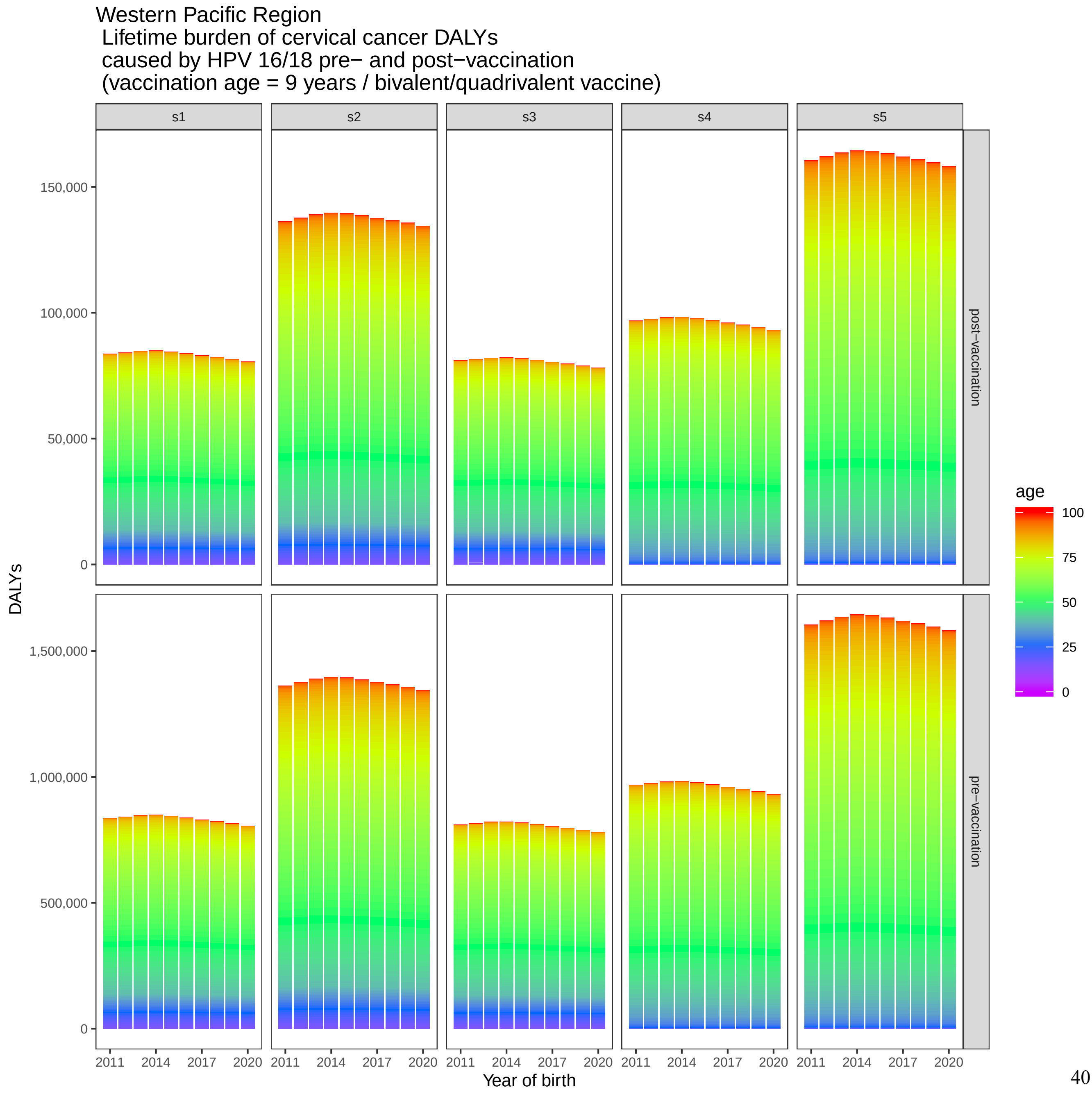

Western Pacific Region
Lifetime burden of cervical cancer DALYs
caused by HPV 16/18 pre− and post−vaccination
(vaccination age = 9 years / bivalent/quadrivalent vaccine)
s1
s2
s3
s4
s5
post−vaccination
pre−vaccination
DALYs
150,000
100,000
50,000
0
1,500,000
1,000,000
500,000
0
2011
2014
2017
2020
Year of birth
age
100
75
50
25
0

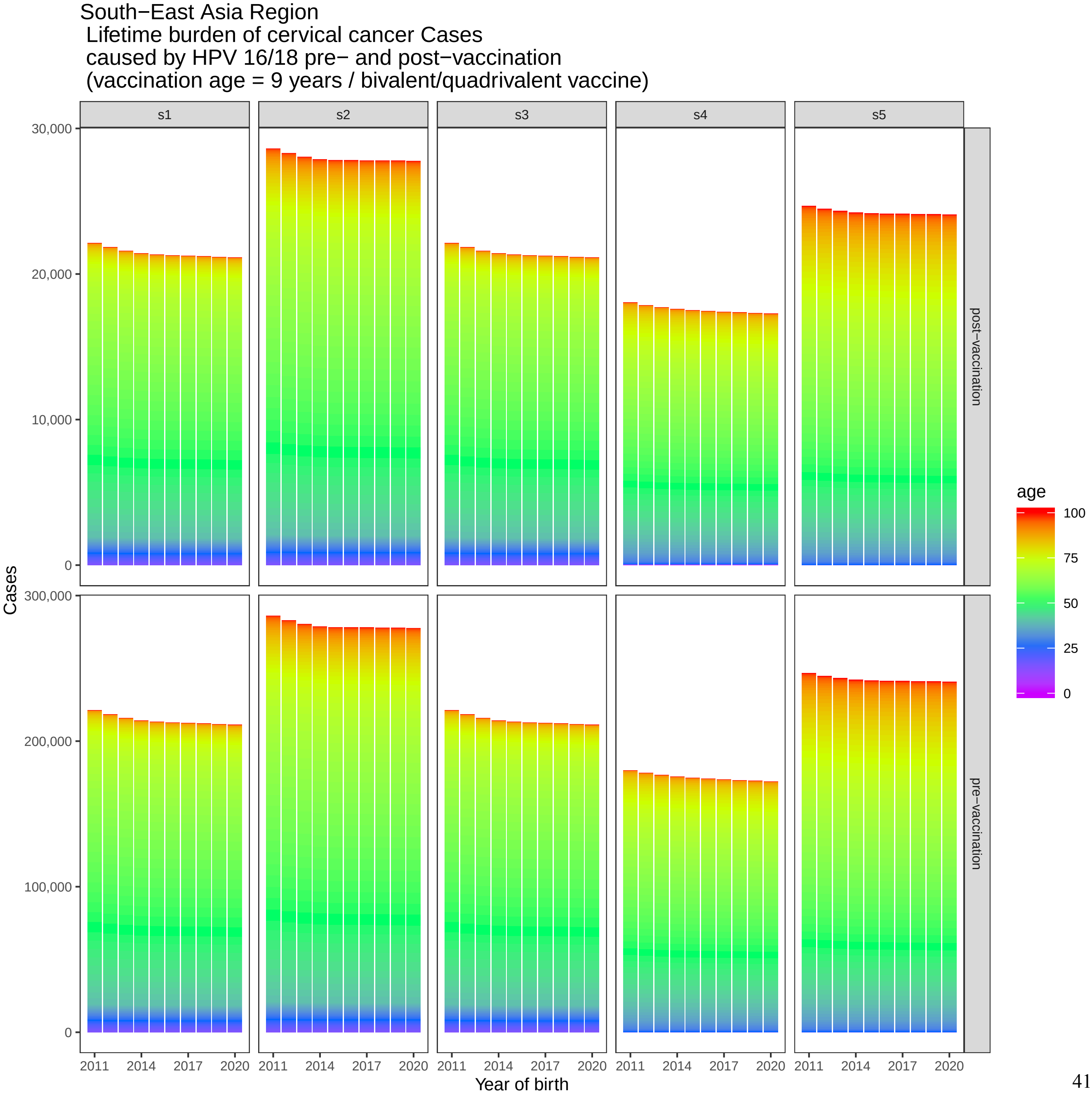
South−East Asia Region
Lifetime burden of cervical cancer Cases
caused by HPV 16/18 pre− and post−vaccination
(vaccination age = 9 years / bivalent/quadrivalent vaccine)
s1
s2
s3
s4
s5
post−vaccination
pre−vaccination
Cases
Year of birth
age
0
25
50
75
100
0
10,000
20,000
30,000
100,000
200,000
300,000
2011
2014
2017
2020

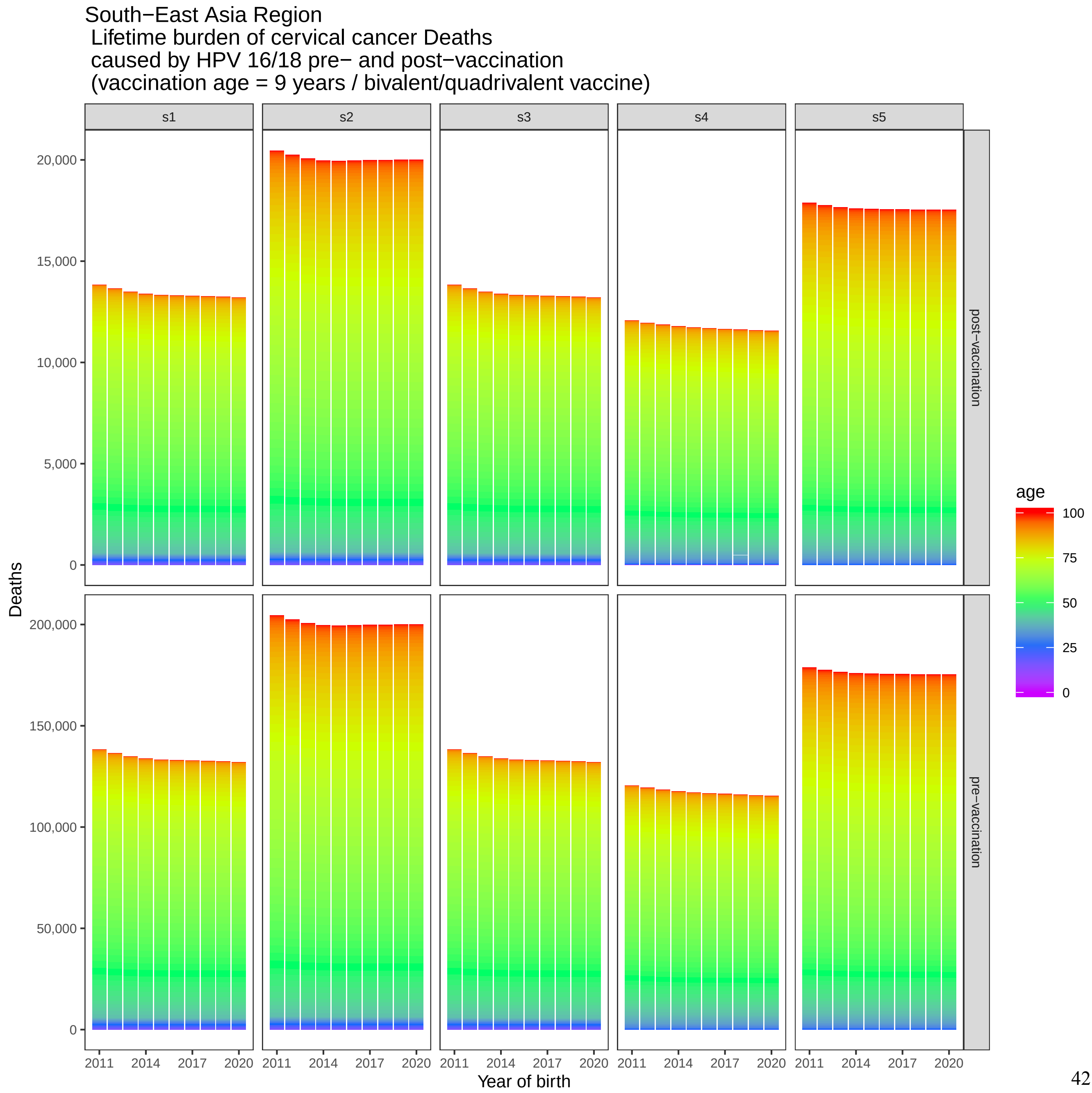
South−East Asia Region
Lifetime burden of cervical cancer Deaths
caused by HPV 16/18 pre− and post−vaccination
(vaccination age = 9 years / bivalent/quadrivalent vaccine)
s1
s2
s3
s4
s5
post−vaccination
pre−vaccination
Deaths
20,000
15,000
10,000
5,000
0
200,000
150,000
100,000
50,000
0
2011
2014
2017
2020
Year of birth
age
100
75
50
25
0

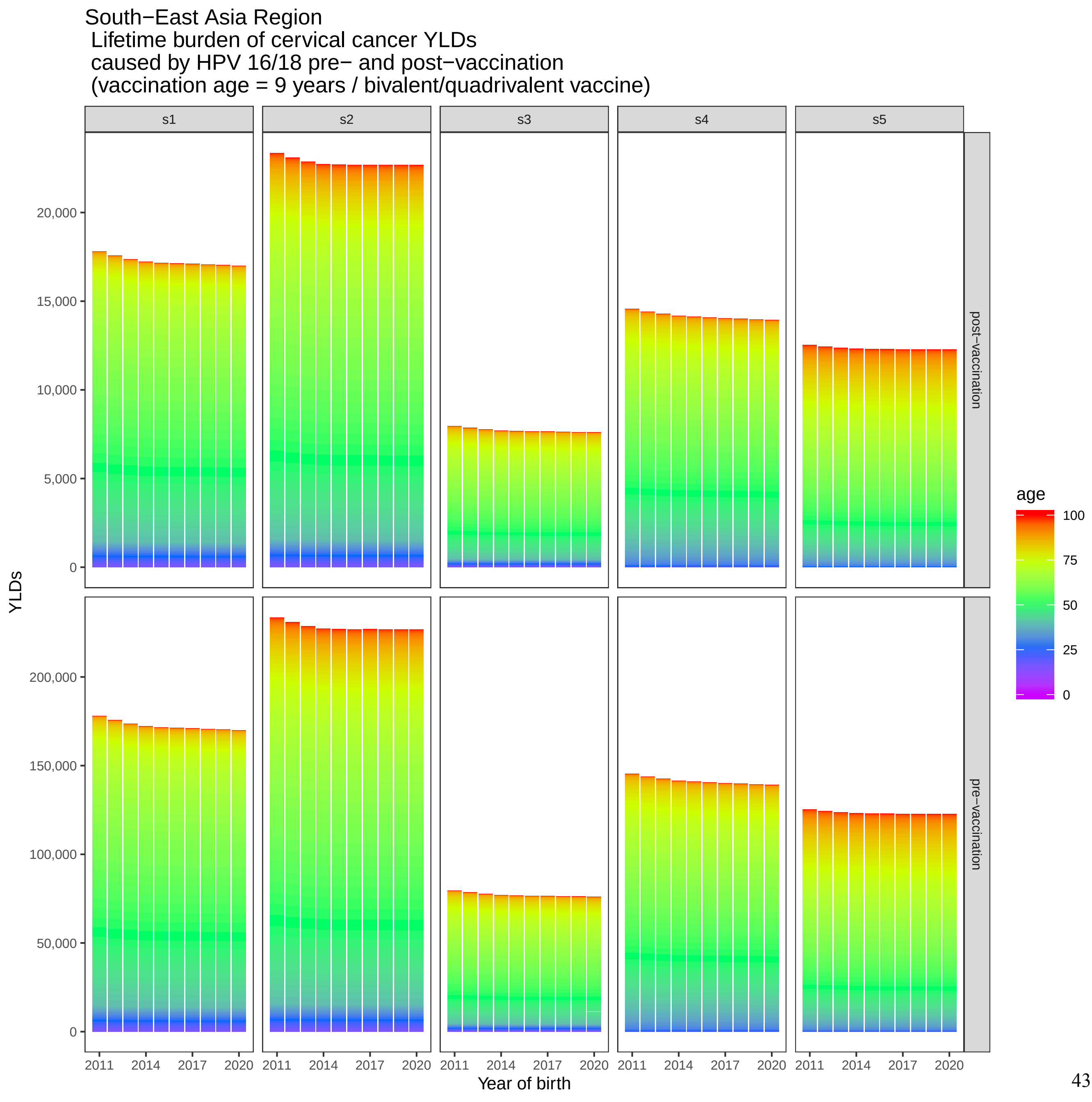
South−East Asia Region
Lifetime burden of cervical cancer YLDs
caused by HPV 16/18 pre− and post−vaccination
(vaccination age = 9 years / bivalent/quadrivalent vaccine)
s1
s2
s3
s4
s5
post−vaccination
pre−vaccination
YLDs
0
5,000
10,000
15,000
20,000
50,000
100,000
150,000
200,000
2011
2014
2017
2020
Year of birth
age
100
75
50
25
0

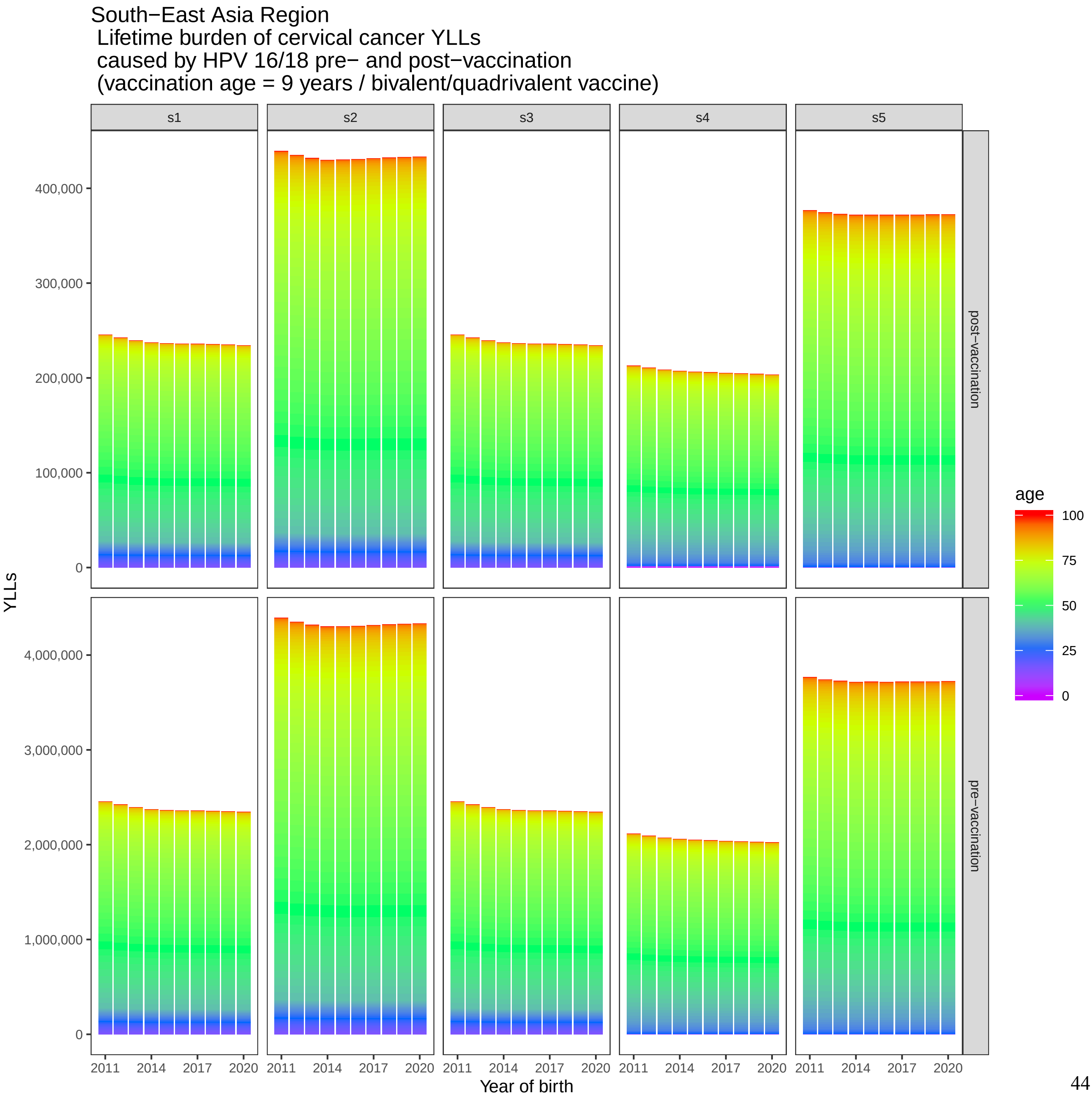
South−East Asia Region
Lifetime burden of cervical cancer YLLs
caused by HPV 16/18 pre− and post−vaccination
(vaccination age = 9 years / bivalent/quadrivalent vaccine)
s1
s2
s3
s4
s5
post−vaccination
pre−vaccination
YLLs
400,000
300,000
200,000
100,000
0
4,000,000
3,000,000
2,000,000
1,000,000
0
2011
2014
2017
2020
Year of birth
age
100
75
50
25
0

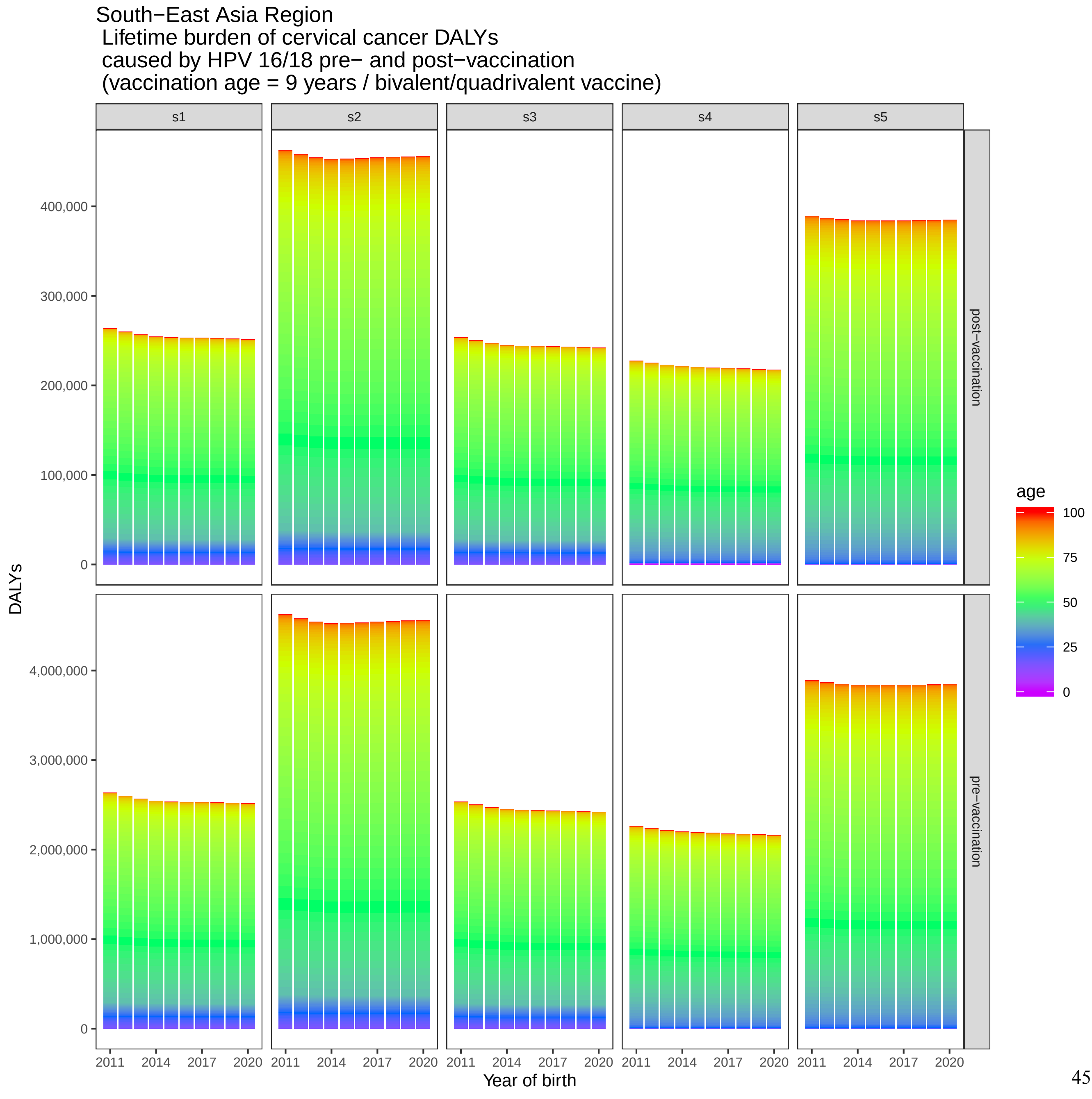
South−East Asia Region
Lifetime burden of cervical cancer DALYs
caused by HPV 16/18 pre− and post−vaccination
(vaccination age = 9 years / bivalent/quadrivalent vaccine)
s1
s2
s3
s4
s5
post−vaccination
pre−vaccination
DALYs
400,000
300,000
200,000
100,000
0
4,000,000
3,000,000
2,000,000
1,000,000
0
2011
2014
2017
2020
Year of birth
age
100
75
50
25
0

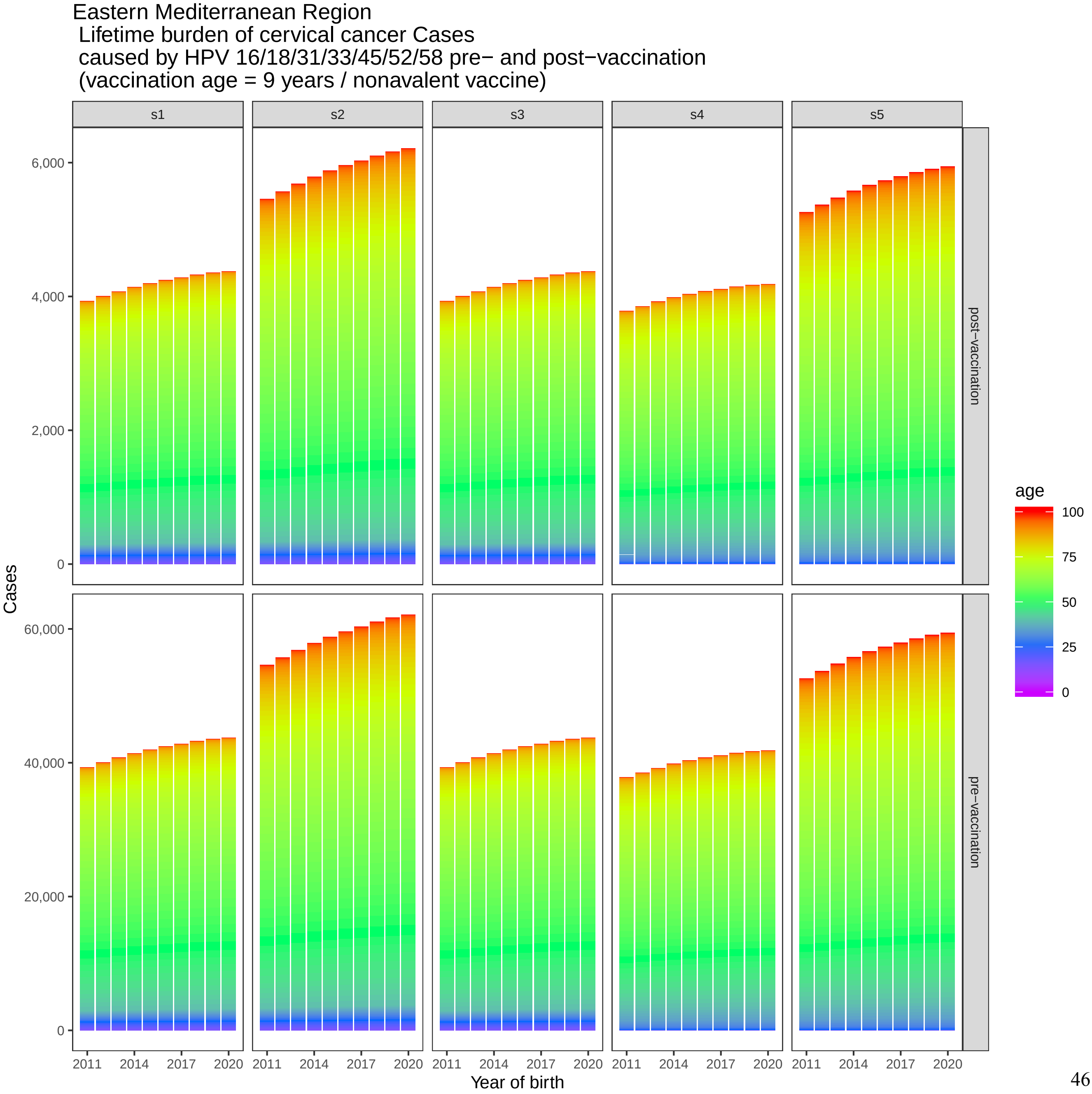
Eastern Mediterranean Region
Lifetime burden of cervical cancer Cases
caused by HPV 16/18/31/33/45/52/58 pre− and post−vaccination
(vaccination age = 9 years / nonavalent vaccine)
s1
s2
s3
s4
s5
post−vaccination
pre−vaccination
Cases
6,000
4,000
2,000
0
60,000
40,000
20,000
0
2011
2014
2017
2020
Year of birth
age
100
75
50
25
0

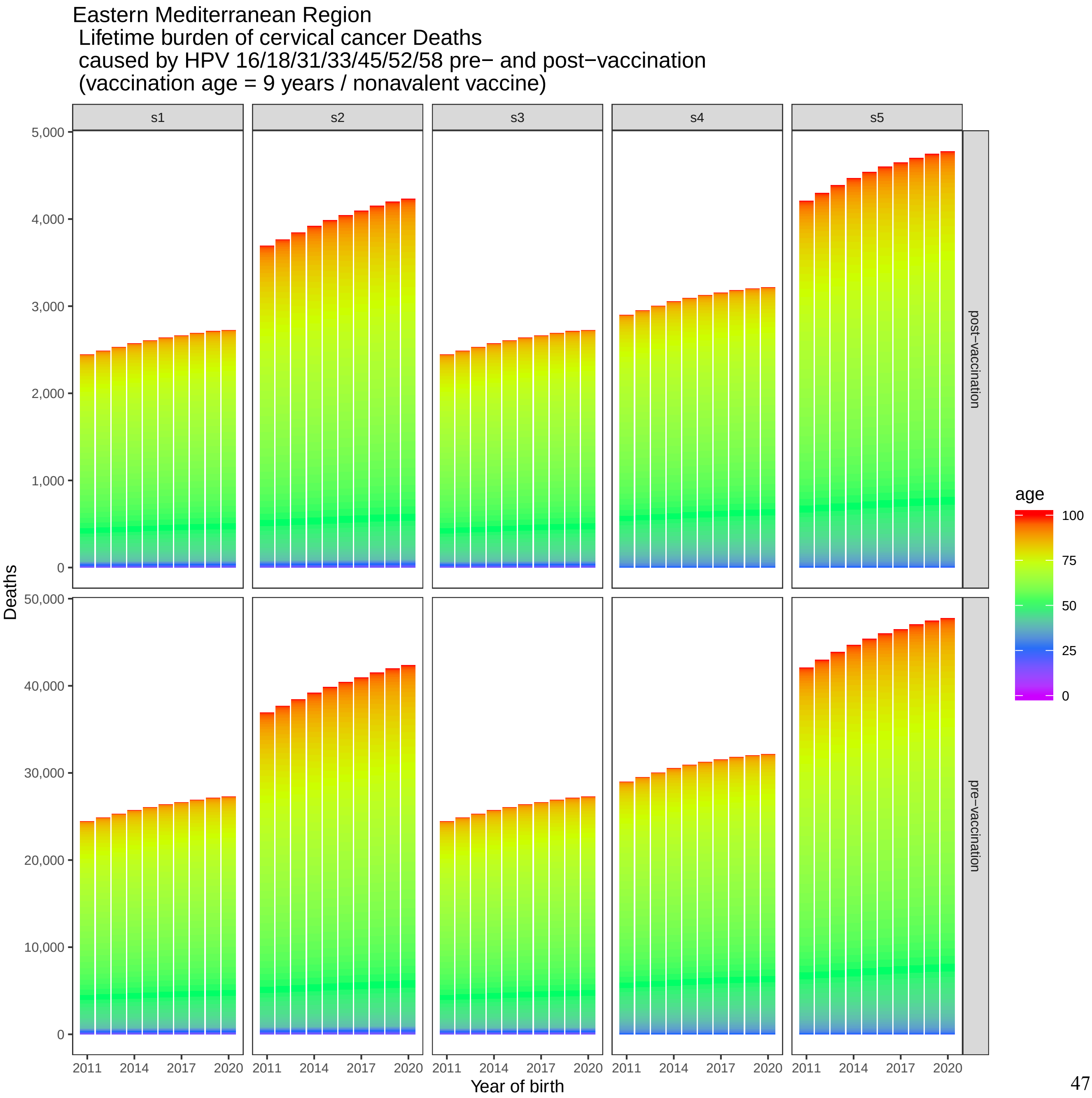
Eastern Mediterranean Region
Lifetime burden of cervical cancer Deaths
caused by HPV 16/18/31/33/45/52/58 pre− and post−vaccination
(vaccination age = 9 years / nonavalent vaccine)
s1
s2
s3
s4
s5
post−vaccination
pre−vaccination
Deaths
Year of birth
2011
2014
2017
2020
0
1,000
2,000
3,000
4,000
5,000
10,000
20,000
30,000
40,000
50,000
age
100
75
50
25
0

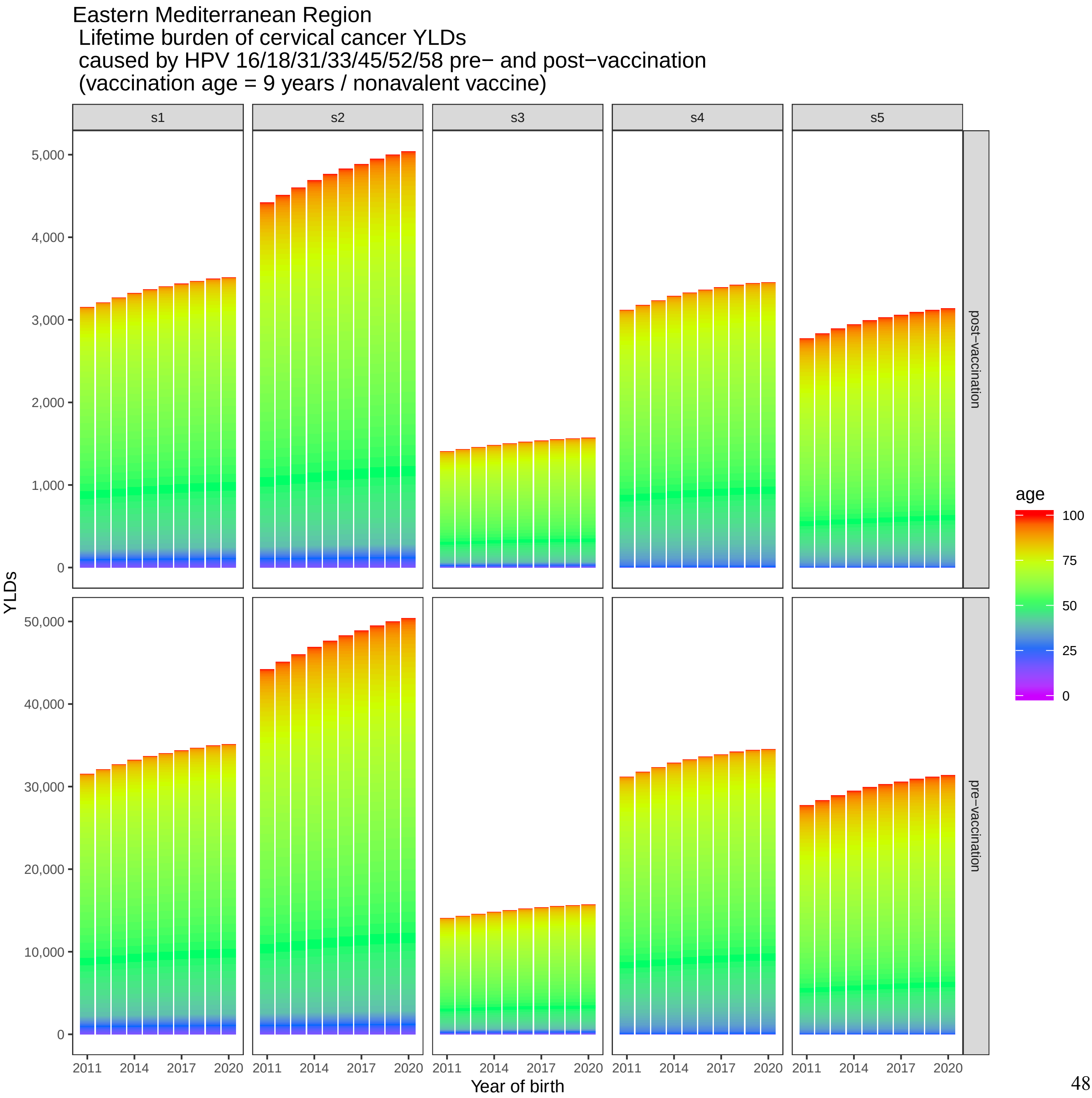

Eastern Mediterranean Region
Lifetime burden of cervical cancer YLDs
caused by HPV 16/18/31/33/45/52/58 pre− and post−vaccination
(vaccination age = 9 years / nonavalent vaccine)
s1
s2
s3
s4
s5
post−vaccination
pre−vaccination
YLDs
Year of birth
age
0
25
50
75
100
0
1,000
2,000
3,000
4,000
5,000
0
10,000
20,000
30,000
40,000
50,000
2011
2014
2017
2020

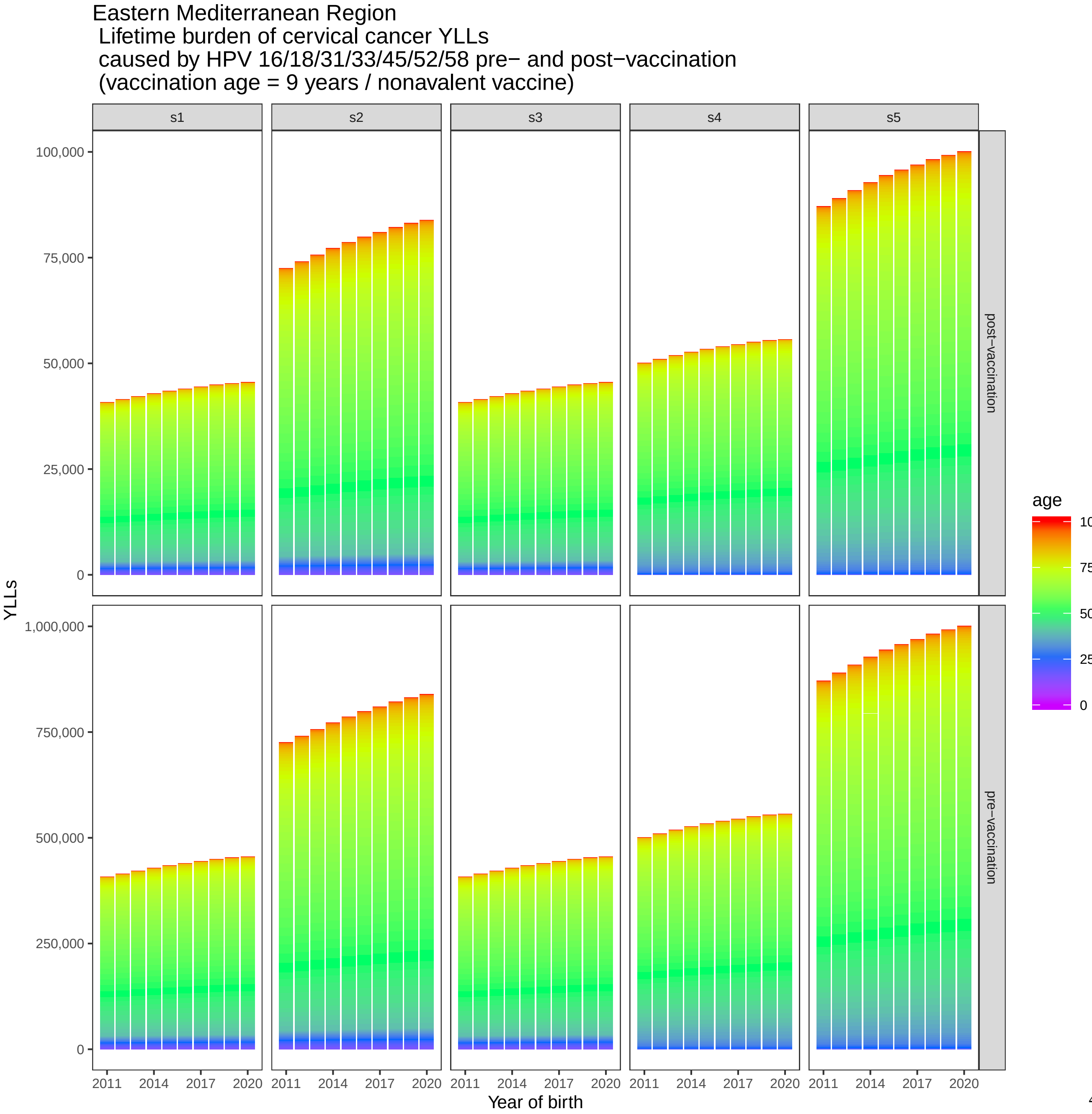
Eastern Mediterranean Region
Lifetime burden of cervical cancer YLLs
caused by HPV 16/18/31/33/45/52/58 pre− and post−vaccination
(vaccination age = 9 years / nonavalent vaccine)
s1
s2
s3
s4
s5
post−vaccination
pre−vaccination
YLLs
100,000
75,000
50,000
25,000
0
1,000,000
750,000
500,000
250,000
0
age
100
75
50
25
0
2011
2014
2017
2020
Year of birth

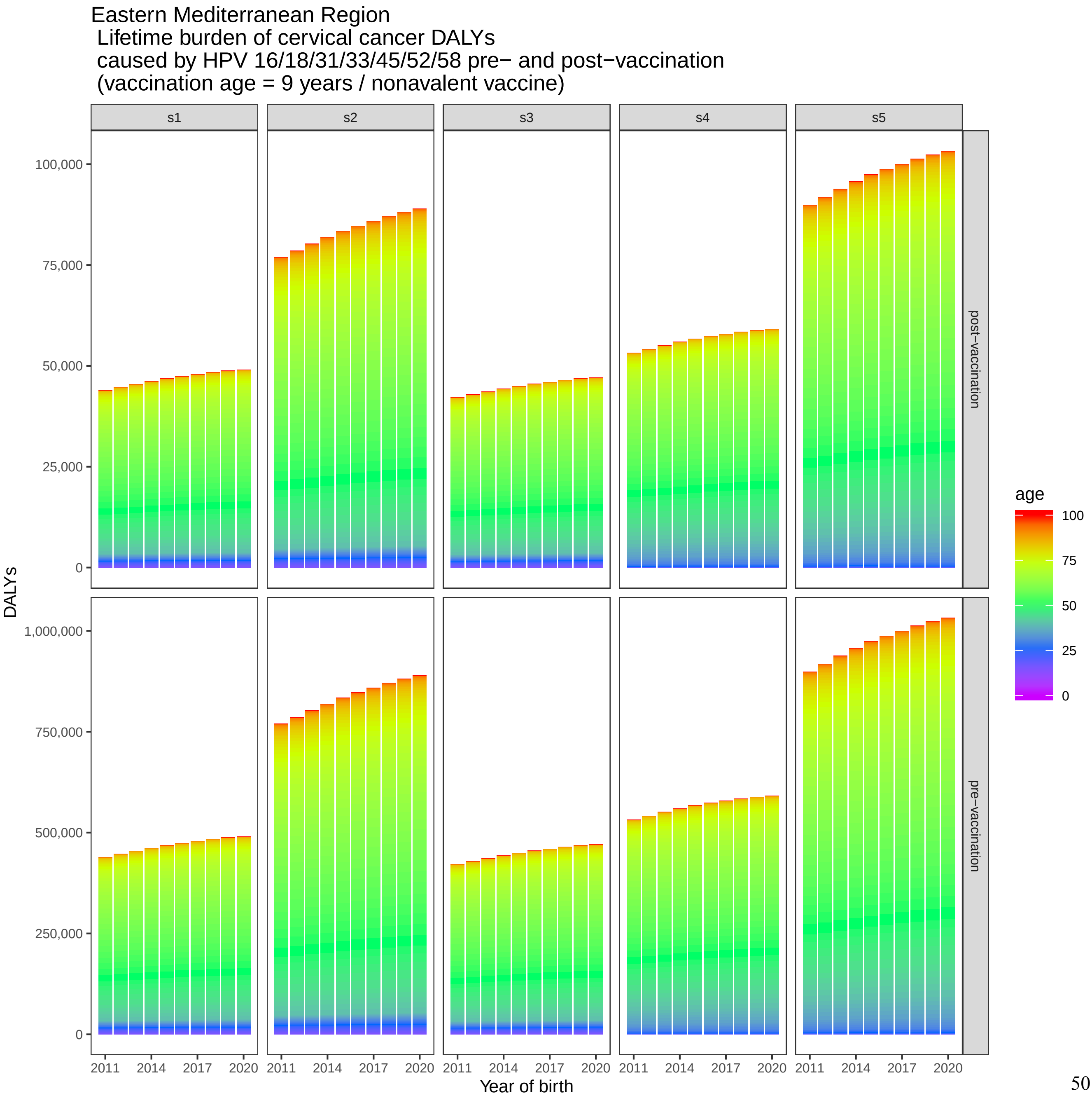
Eastern Mediterranean Region
Lifetime burden of cervical cancer DALYs
caused by HPV 16/18/31/33/45/52/58 pre− and post−vaccination
(vaccination age = 9 years / nonavalent vaccine)
s1
s2
s3
s4
s5
post−vaccination
pre−vaccination
DALYs
100,000
75,000
50,000
25,000
0
1,000,000
750,000
500,000
250,000
0
2011
2014
2017
2020
Year of birth
age
100
75
50
25
0

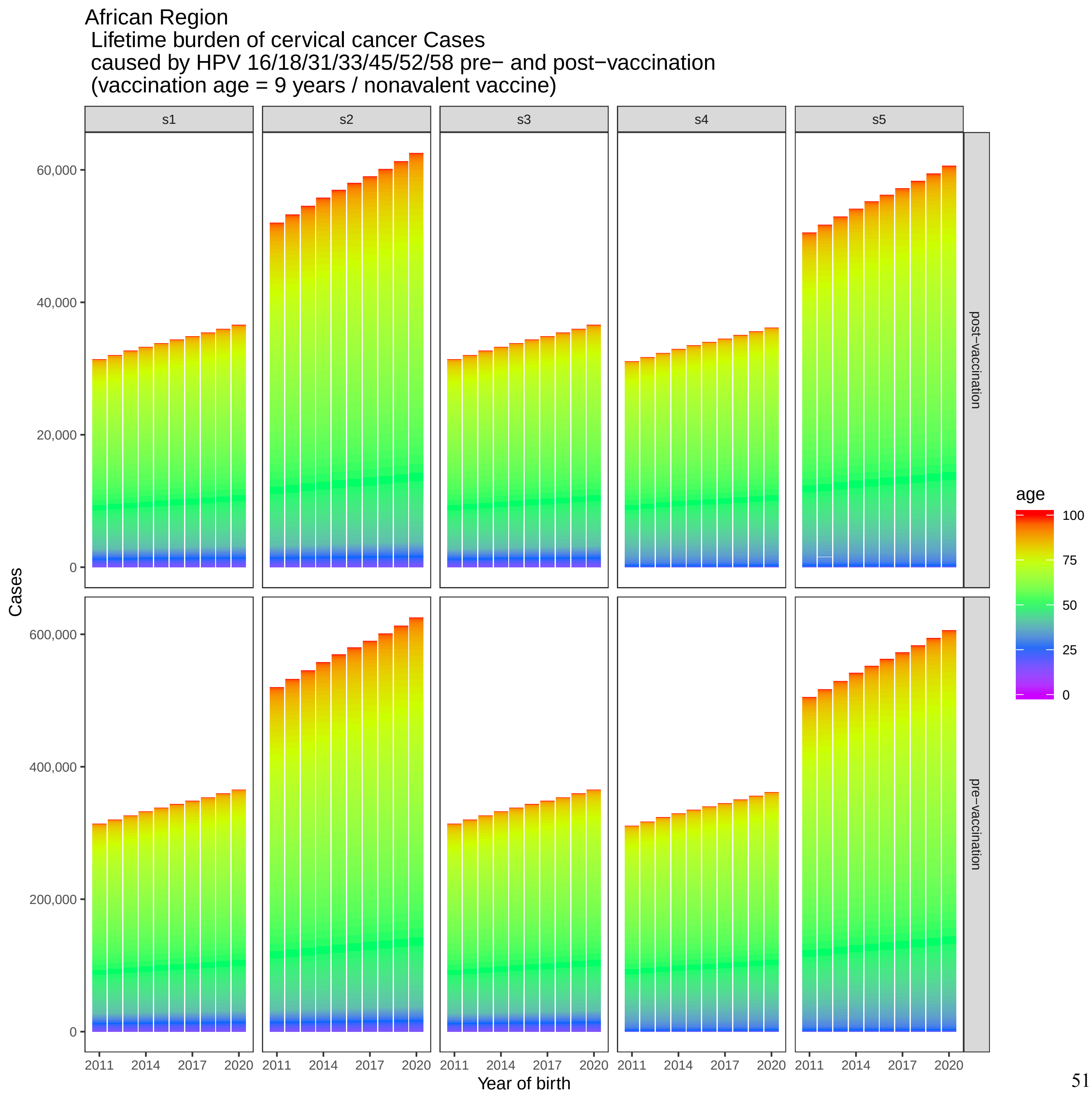

African Region
Lifetime burden of cervical cancer Cases
caused by HPV 16/18/31/33/45/52/58 pre− and post−vaccination
(vaccination age = 9 years / nonavalent vaccine)
s1
s2
s3
s4
s5
post−vaccination
pre−vaccination
Cases
0
20,000
40,000
60,000
0
200,000
400,000
600,000
2011
2014
2017
2020
Year of birth
age
100
75
50
25
0

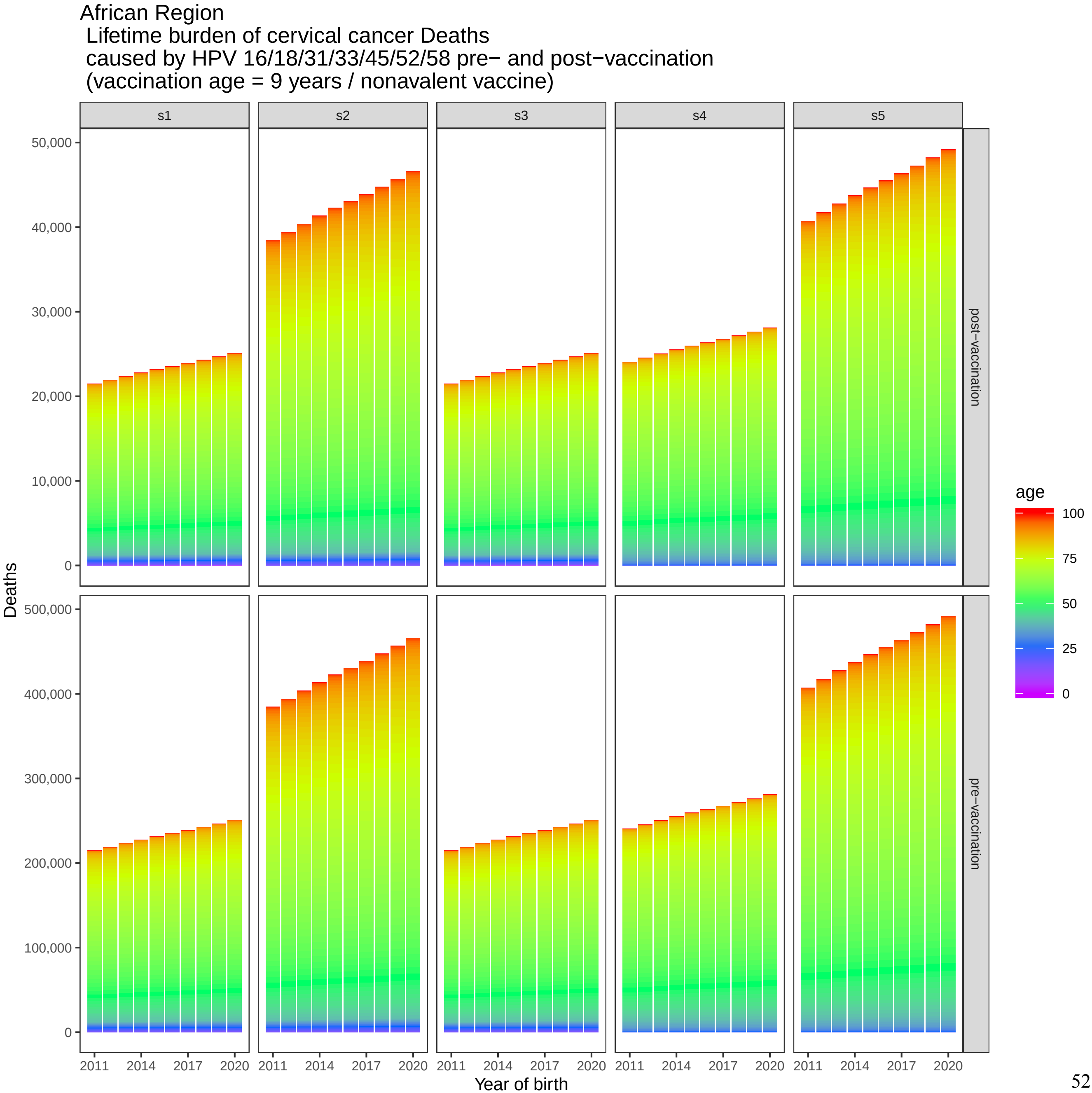
African Region
Lifetime burden of cervical cancer Deaths
caused by HPV 16/18/31/33/45/52/58 pre− and post−vaccination
(vaccination age = 9 years / nonavalent vaccine)
s1
s2
s3
s4
s5
post−vaccination
pre−vaccination
Deaths
Year of birth
age
50,000
40,000
30,000
20,000
10,000
0
500,000
400,000
300,000
200,000
100,000
0
2011
2014
2017
2020
100
75
50
25
0

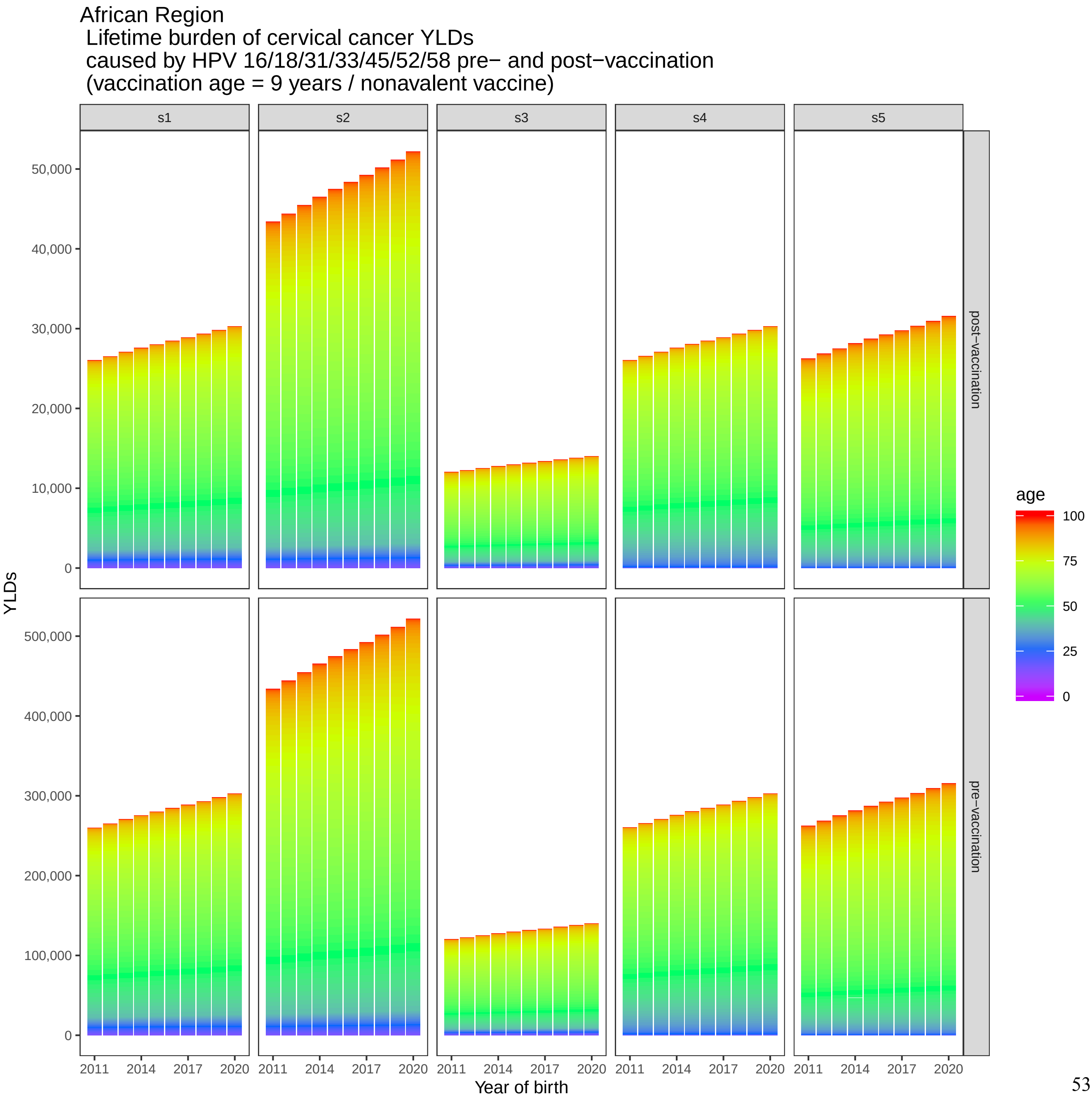
African Region
Lifetime burden of cervical cancer YLDs
caused by HPV 16/18/31/33/45/52/58 pre− and post−vaccination
(vaccination age = 9 years / nonavalent vaccine)
s1
s2
s3
s4
s5
post−vaccination
pre−vaccination
YLDs
0
10,000
20,000
30,000
40,000
50,000
100,000
200,000
300,000
400,000
500,000
2011
2014
2017
2020
Year of birth
age
100
75
50
25
0

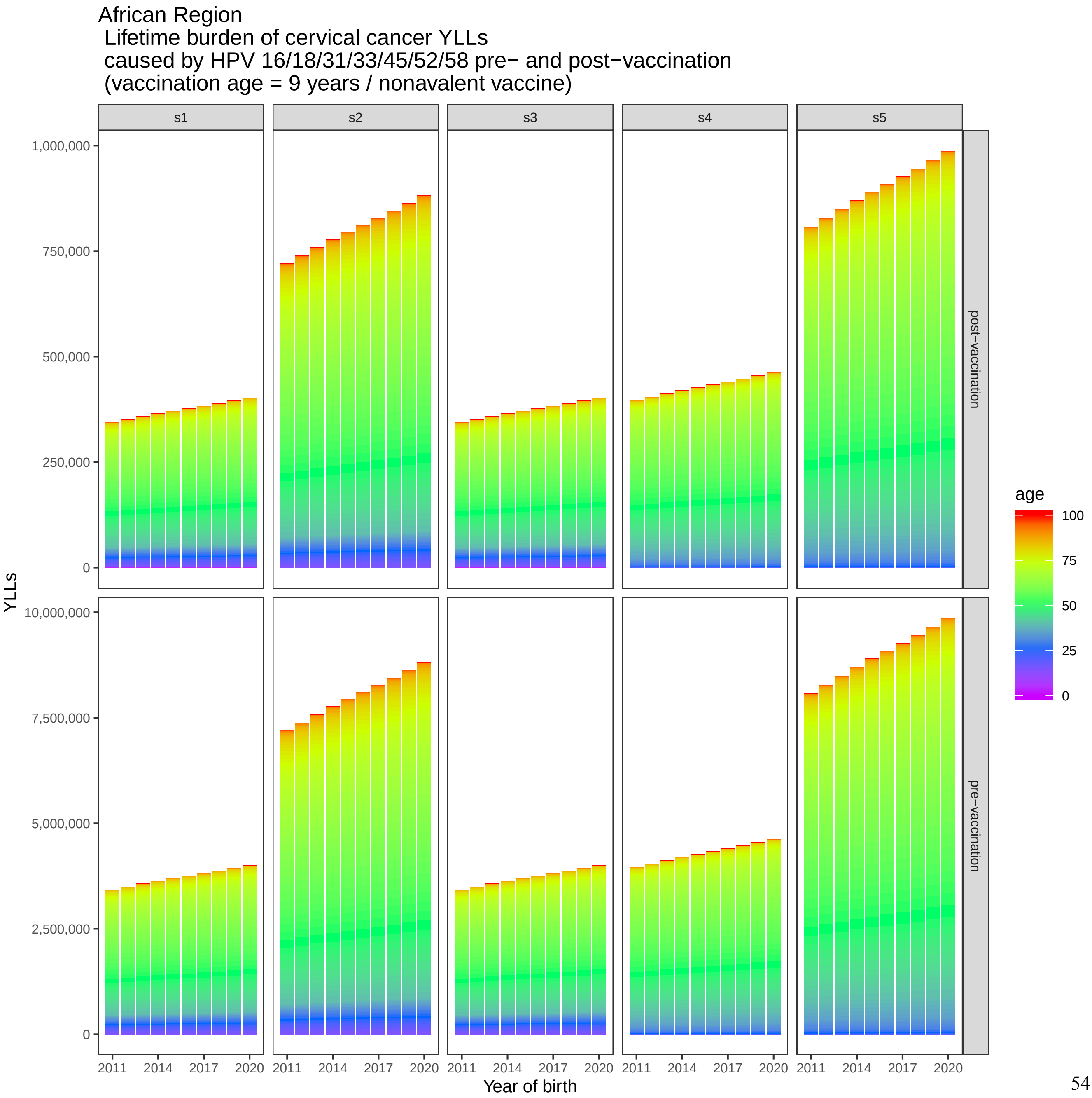
African Region
Lifetime burden of cervical cancer YLLs
caused by HPV 16/18/31/33/45/52/58 pre− and post−vaccination
(vaccination age = 9 years / nonavalent vaccine)
s1
s2
s3
s4
s5
post−vaccination
pre−vaccination
YLLs
1,000,000
750,000
500,000
250,000
0
10,000,000
7,500,000
5,000,000
2,500,000
0
2011
2014
2017
2020
Year of birth
age
100
75
50
25
0

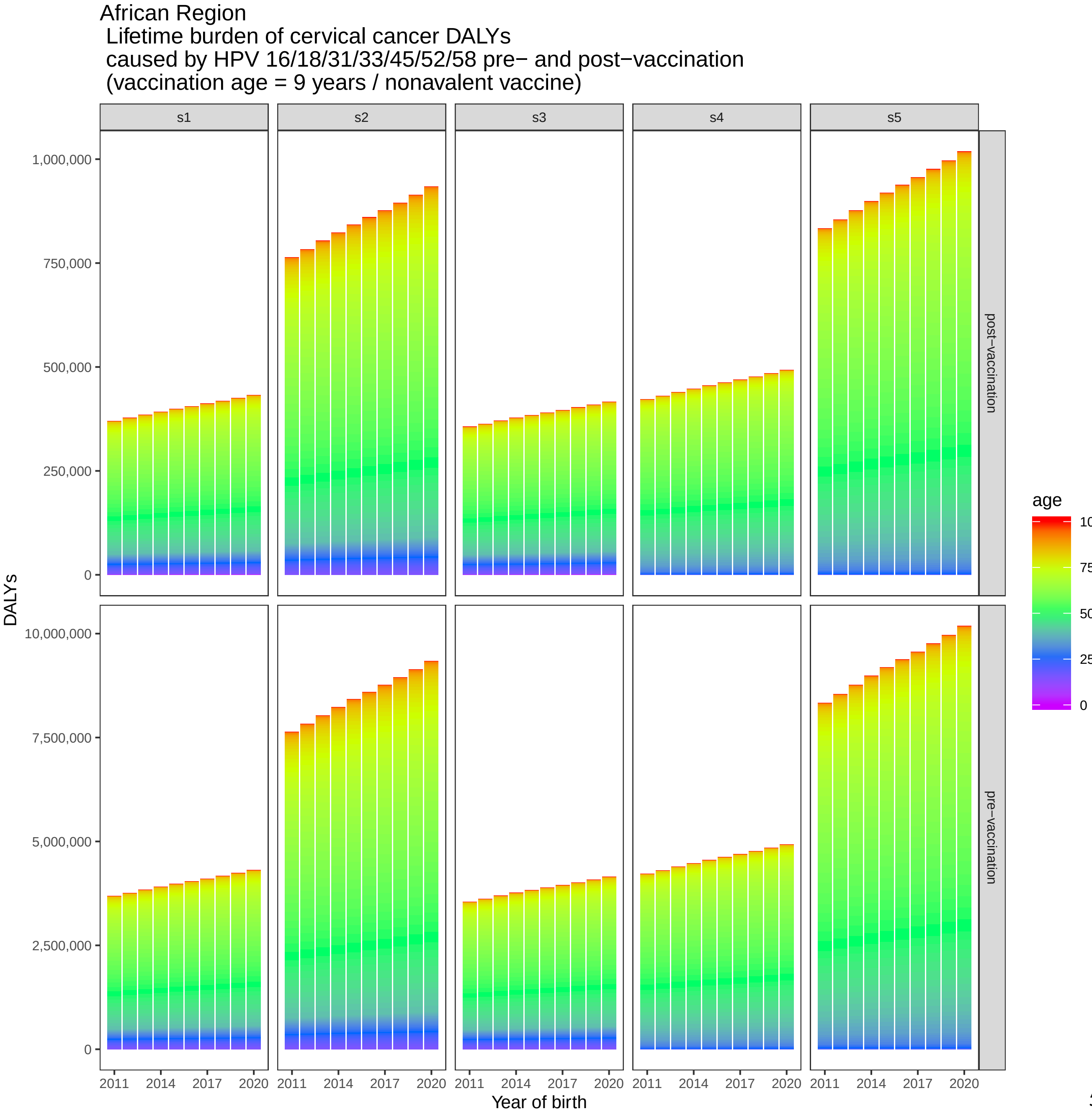
African Region
Lifetime burden of cervical cancer DALYs
caused by HPV 16/18/31/33/45/52/58 pre− and post−vaccination
(vaccination age = 9 years / nonavalent vaccine)
s1
s2
s3
s4
s5
post−vaccination
pre−vaccination
DALYs
1,000,000
750,000
500,000
250,000
0
10,000,000
7,500,000
5,000,000
2,500,000
0
2011
2014
2017
2020
Year of birth
age
100
75
50
25
0

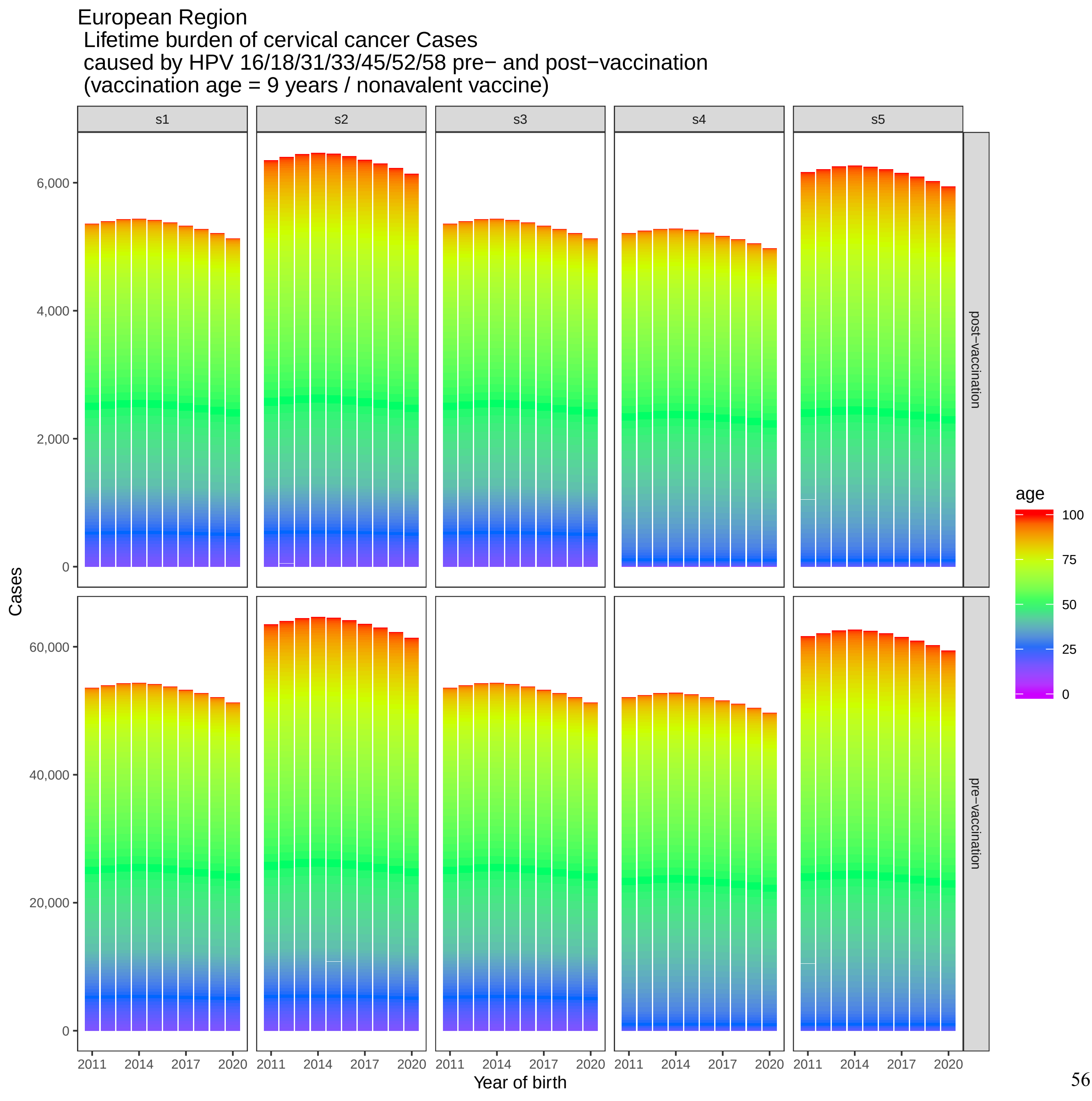
European Region
Lifetime burden of cervical cancer Cases
caused by HPV 16/18/31/33/45/52/58 pre− and post−vaccination
(vaccination age = 9 years / nonavalent vaccine)
s1
s2
s3
s4
s5
post−vaccination
pre−vaccination
Cases
Year of birth
2011
2014
2017
2020
6,000
4,000
2,000
0
60,000
40,000
20,000
age
100
75
50
25

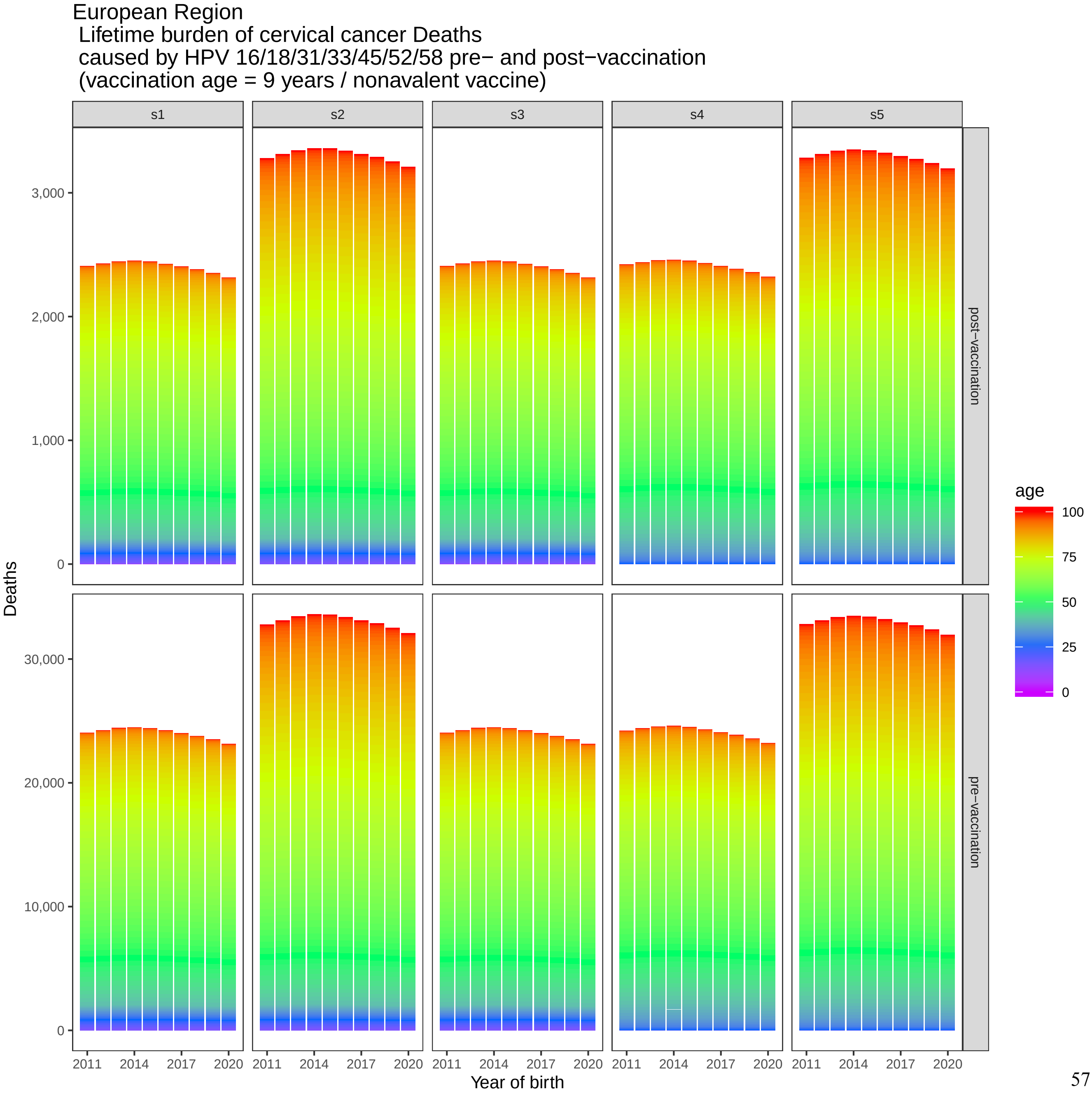
European Region
Lifetime burden of cervical cancer Deaths
caused by HPV 16/18/31/33/45/52/58 pre− and post−vaccination
(vaccination age = 9 years / nonavalent vaccine)
s1
s2
s3
s4
s5
post−vaccination
pre−vaccination
Deaths
Year of birth
age
100
75
50
25
0
3,000
2,000
1,000
30,000
20,000
10,000
2011
2014
2017
2020

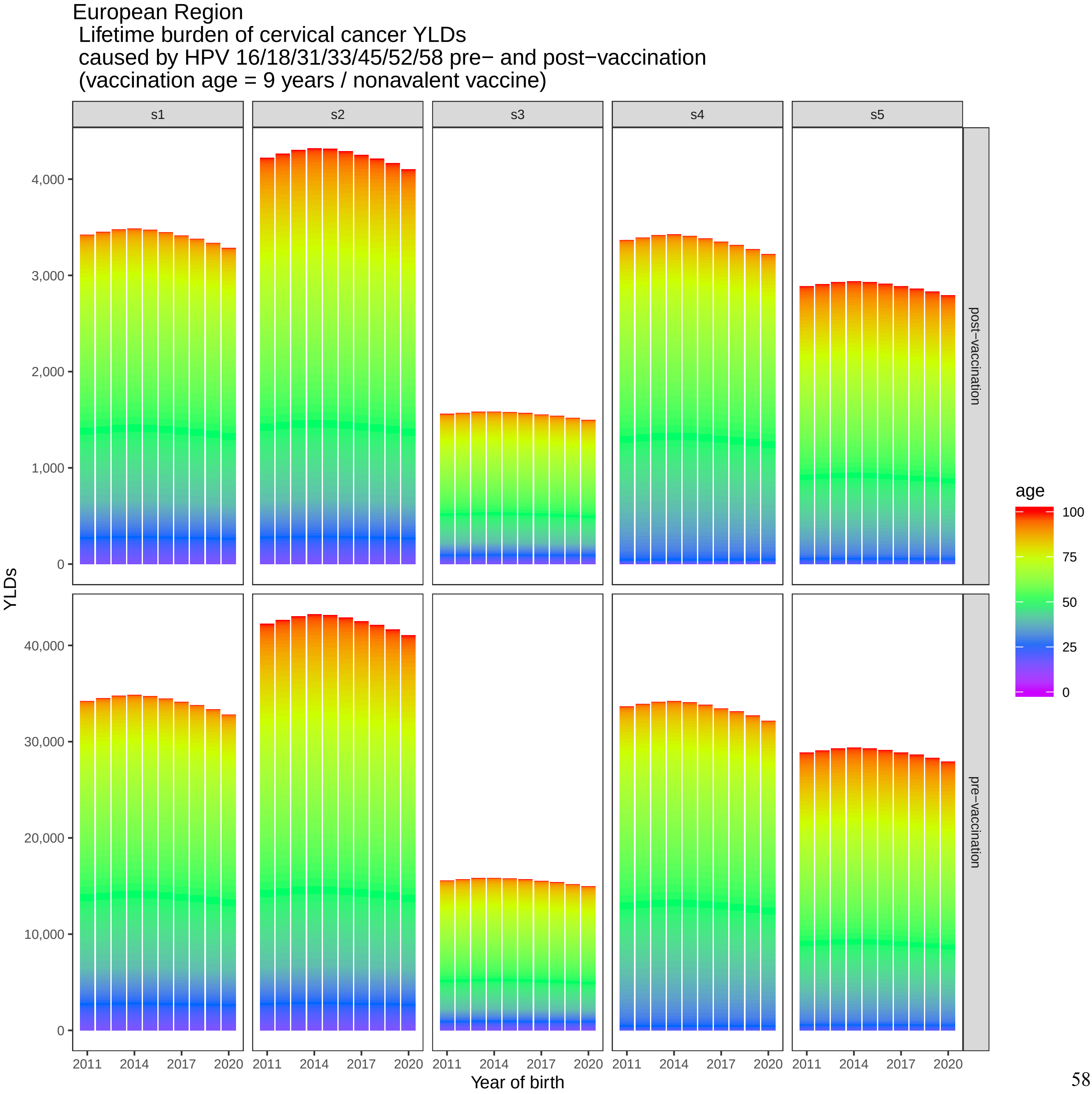
European Region
Lifetime burden of cervical cancer YLDs
caused by HPV 16/18/31/33/45/52/58 pre− and post−vaccination
(vaccination age = 9 years / nonavalent vaccine)
s1
s2
s3
s4
s5
post−vaccination
pre−vaccination
YLDs
0
1,000
2,000
3,000
4,000
10,000
20,000
30,000
40,000
2011
2014
2017
2020
Year of birth
age
0
25
50
75
100

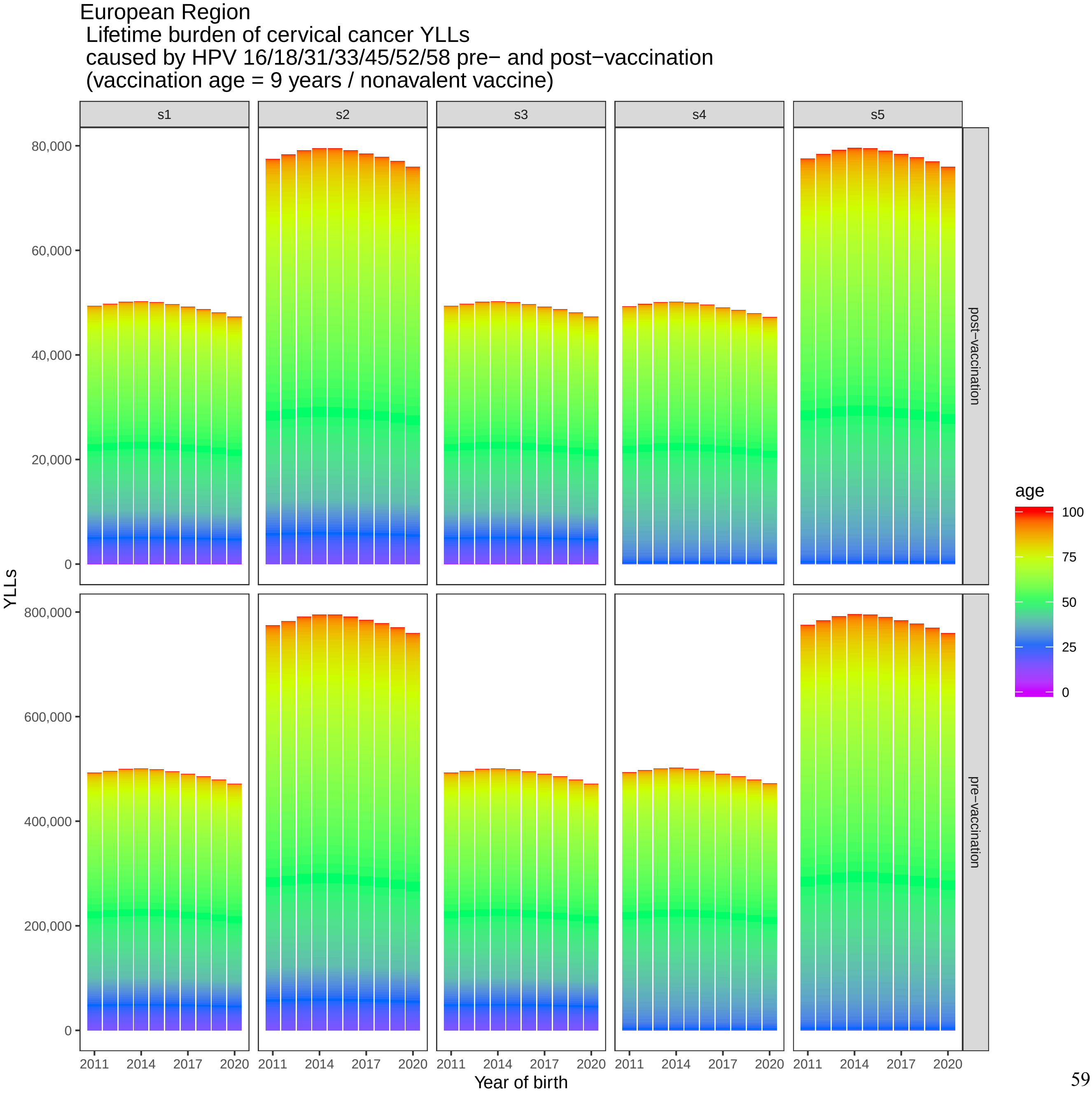

European Region
Lifetime burden of cervical cancer YLLs
caused by HPV 16/18/31/33/45/52/58 pre− and post−vaccination
(vaccination age = 9 years / nonavalent vaccine)
s1
s2
s3
s4
s5
post−vaccination
pre−vaccination
YLLs
Year of birth
age

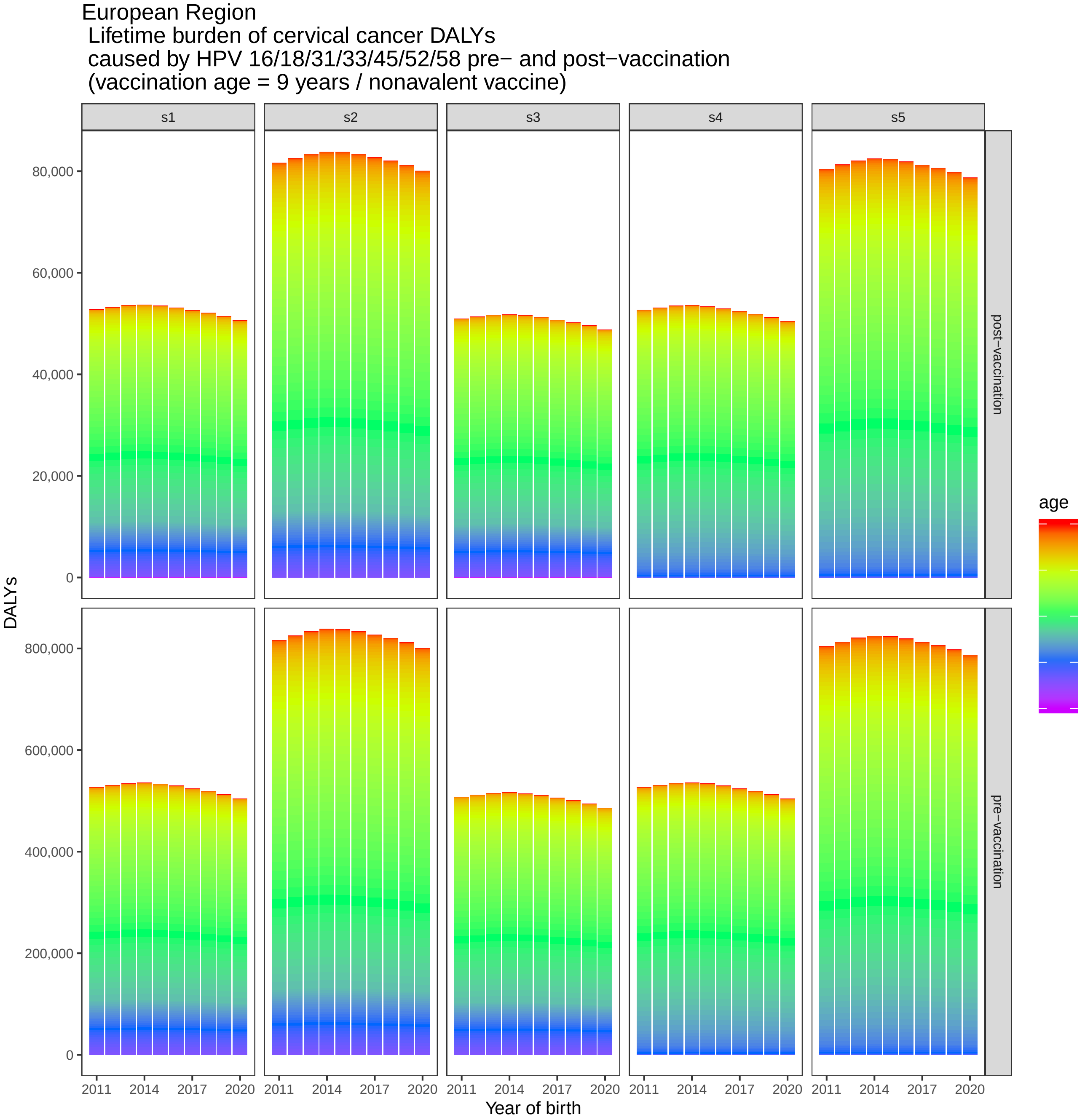
European Region
Lifetime burden of cervical cancer DALYs
caused by HPV 16/18/31/33/45/52/58 pre− and post−vaccination
(vaccination age = 9 years / nonavalent vaccine)
s1
s2
s3
s4
s5
post−vaccination
pre−vaccination
DALYs
0
20,000
40,000
60,000
80,000
200,000
400,000
600,000
800,000
age
100
75
50
25
2011
2014
2017
2020
Year of birth

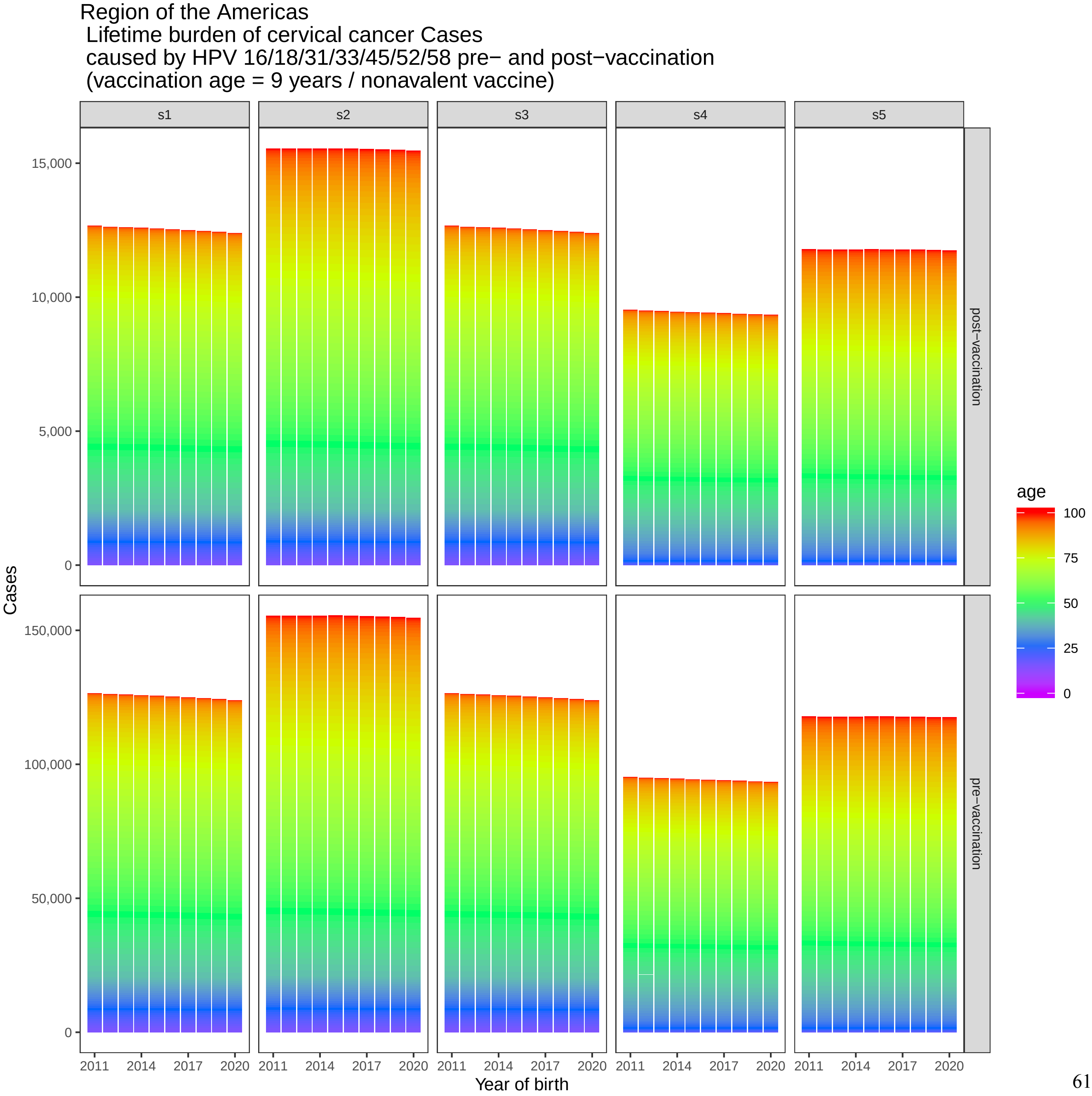

Region of the Americas
Lifetime burden of cervical cancer Cases
caused by HPV 16/18/31/33/45/52/58 pre− and post−vaccination
(vaccination age = 9 years / nonavalent vaccine)
s1
s2
s3
s4
s5
post−vaccination
pre−vaccination
Cases
Year of birth
age
15,000
10,000
5,000
0
150,000
100,000
50,000
2011
2014
2017
2020
100
75
50
25

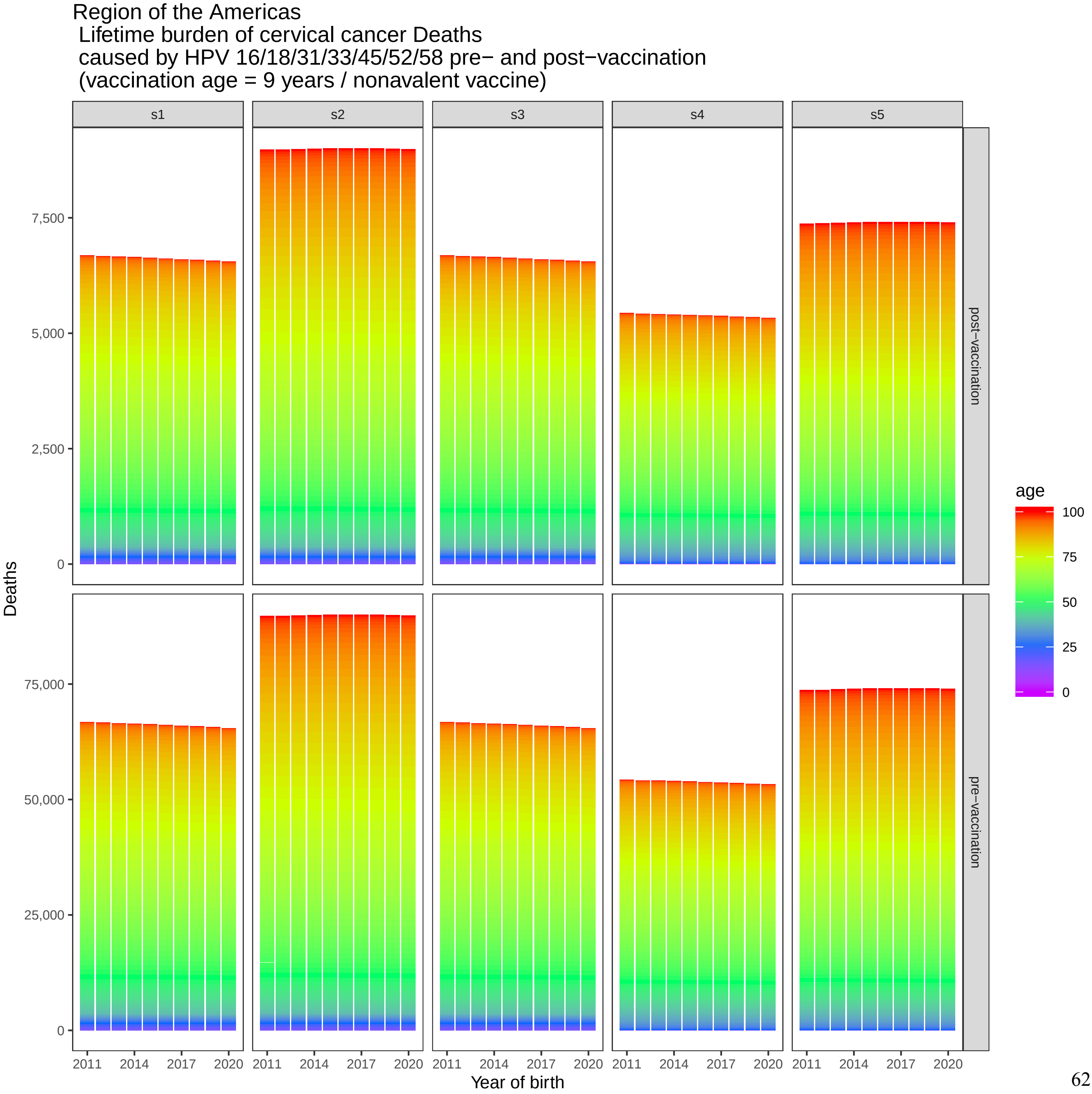

Region of the Americas
Lifetime burden of cervical cancer Deaths
caused by HPV 16/18/31/33/45/52/58 pre− and post−vaccination
(vaccination age = 9 years / nonavalent vaccine)
s1
s2
s3
s4
s5
post−vaccination
pre−vaccination
Deaths
7,500
5,000
2,500
0
75,000
50,000
25,000
0
2011
2014
2017
2020
Year of birth
age
100
75
50
25
0

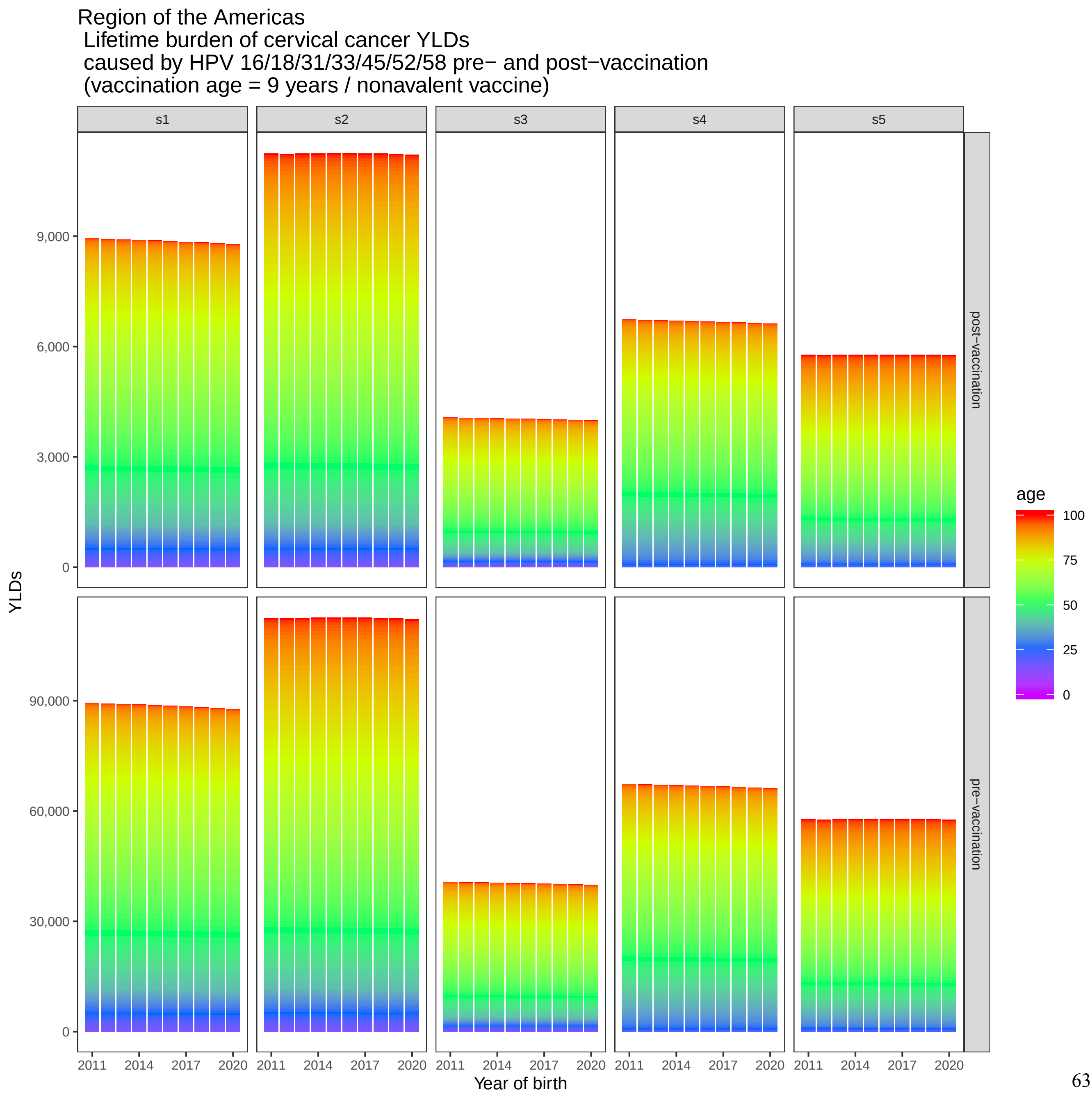
Region of the Americas
Lifetime burden of cervical cancer YLDs
caused by HPV 16/18/31/33/45/52/58 pre− and post−vaccination
(vaccination age = 9 years / nonavalent vaccine)
s1
s2
s3
s4
s5
post−vaccination
pre−vaccination
YLDs
9,000
6,000
3,000
0
90,000
60,000
30,000
0
2011
2014
2017
2020
Year of birth
age
100
75
50
25
0

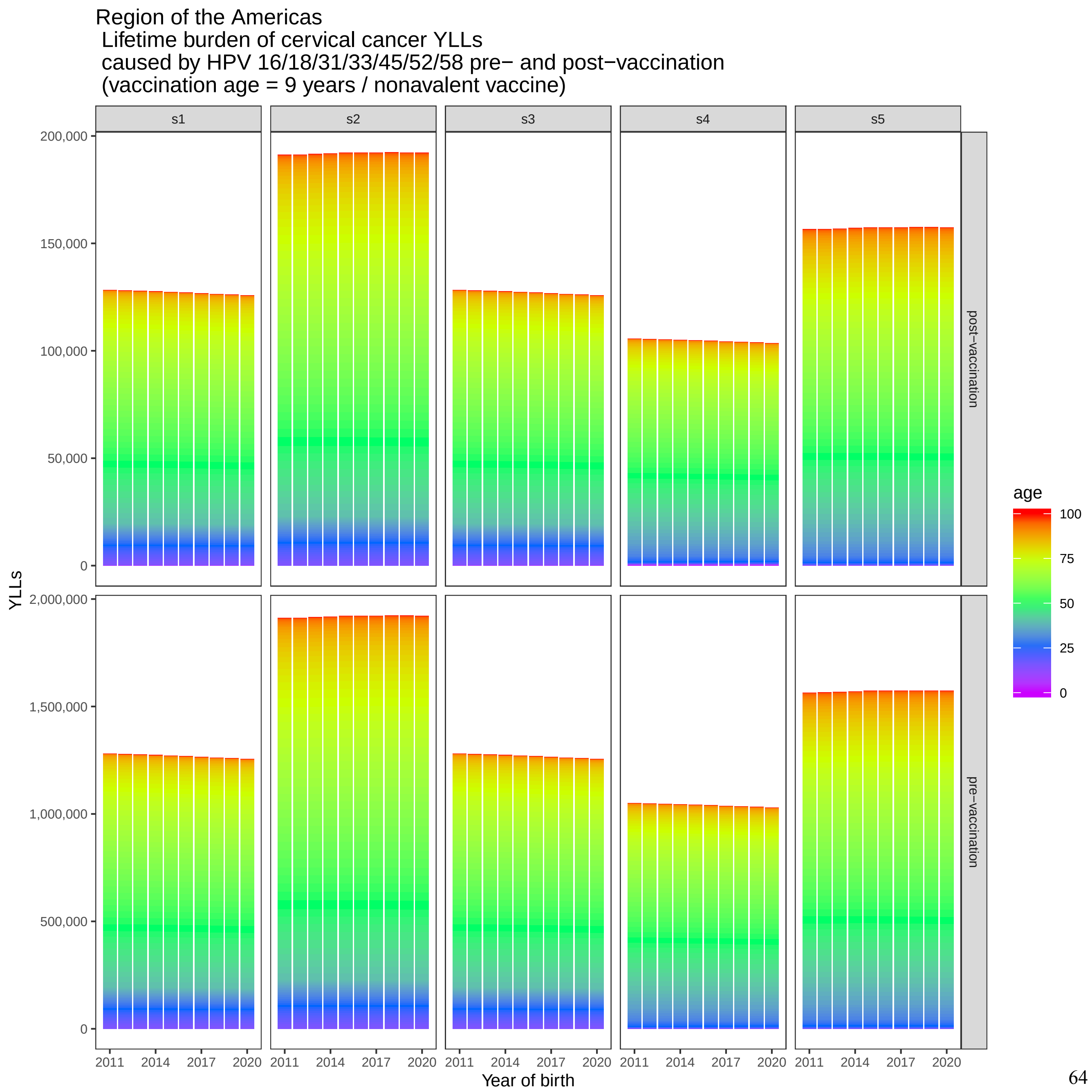

Region of the Americas
Lifetime burden of cervical cancer YLLs
caused by HPV 16/18/31/33/45/52/58 pre− and post−vaccination
(vaccination age = 9 years / nonavalent vaccine)
s1
s2
s3
s4
s5
post−vaccination
pre−vaccination
YLLs
Year of birth
age
0
25
50
75
100
200,000
150,000
100,000
50,000
2,000,000
1,500,000
1,000,000
500,000
2011
2014
2017
2020

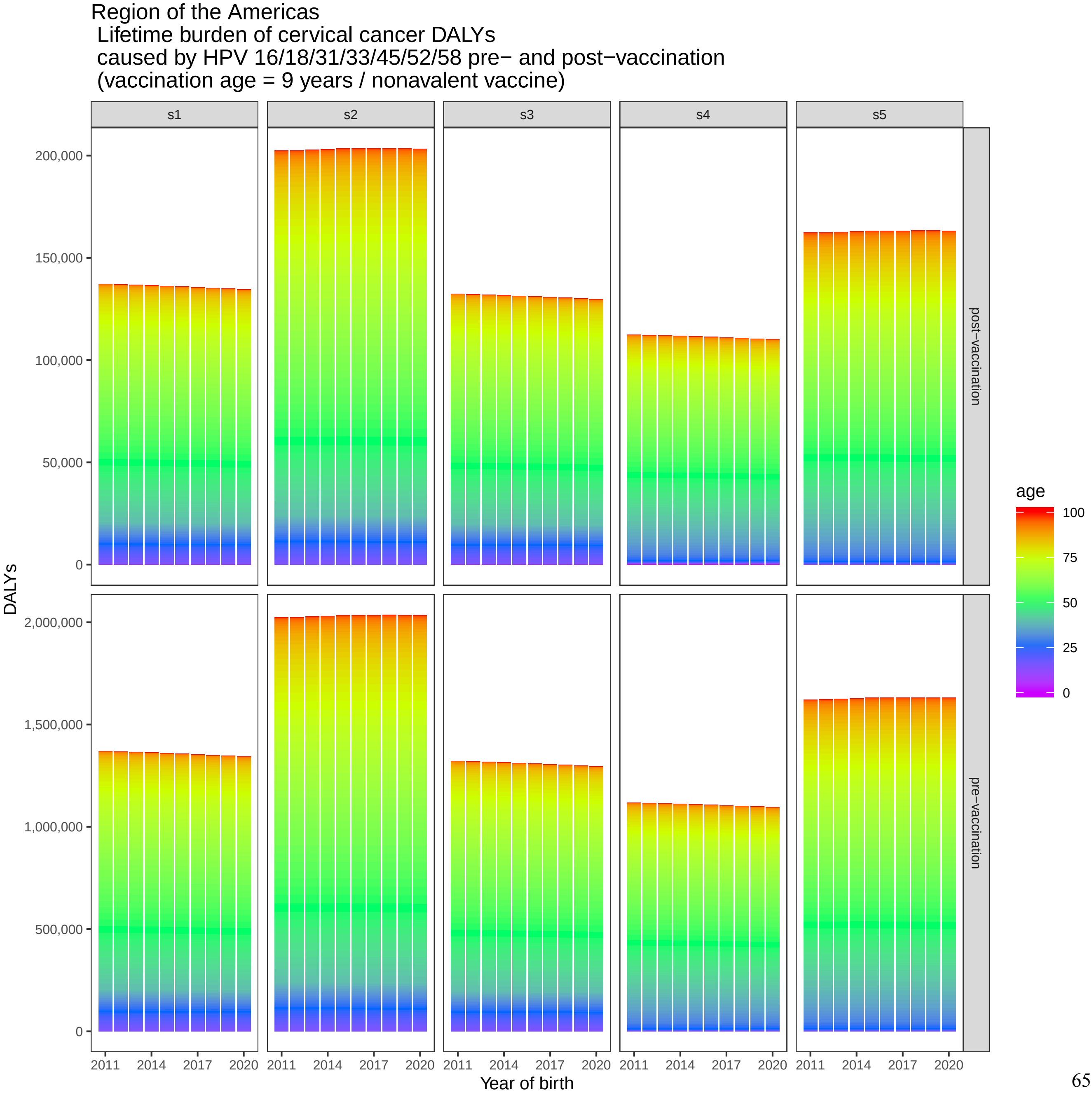
Region of the Americas
Lifetime burden of cervical cancer DALYs
caused by HPV 16/18/31/33/45/52/58 pre− and post−vaccination
(vaccination age = 9 years / nonavalent vaccine)
s1
s2
s3
s4
s5
post−vaccination
pre−vaccination
DALYs
Year of birth
age
0
25
50
75
100
200,000
150,000
100,000
50,000
2,000,000
1,500,000
1,000,000
500,000
2011
2014
2017
2020

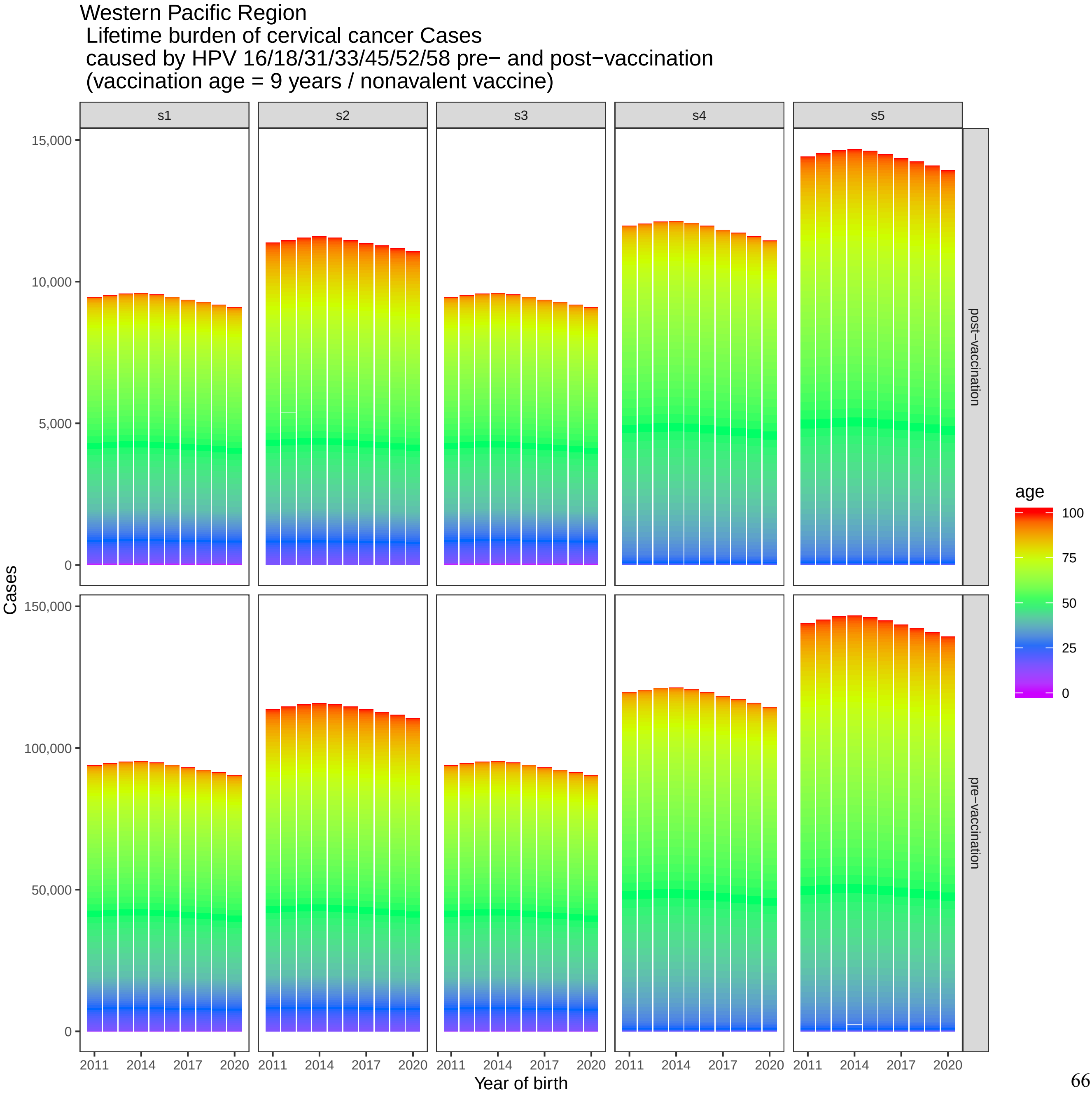

Western Pacific Region
Lifetime burden of cervical cancer Cases
caused by HPV 16/18/31/33/45/52/58 pre− and post−vaccination
(vaccination age = 9 years / nonavalent vaccine)
s1
s2
s3
s4
s5
post−vaccination
pre−vaccination
Cases
Year of birth
age
0
25
50
75
100
15,000
10,000
5,000
150,000
100,000
50,000
2011
2014
2017
2020

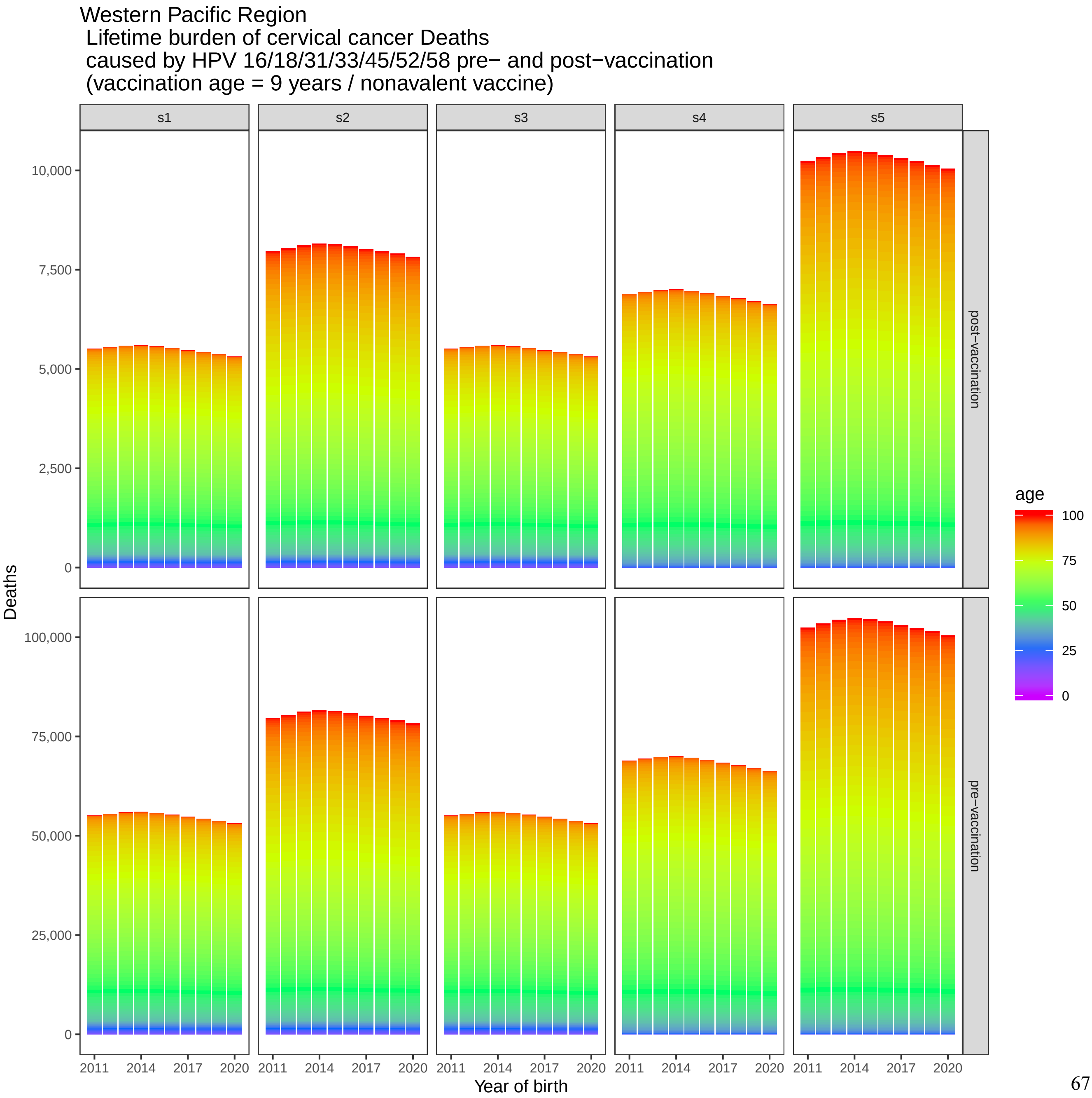

Western Pacific Region
Lifetime burden of cervical cancer Deaths
caused by HPV 16/18/31/33/45/52/58 pre− and post−vaccination
(vaccination age = 9 years / nonavalent vaccine)
s1
s2
s3
s4
s5
post−vaccination
pre−vaccination
Deaths
Year of birth
age
0
25
50
75
100
10,000
7,500
5,000
2,500
100,000
75,000
50,000
25,000
2011
2014
2017
2020

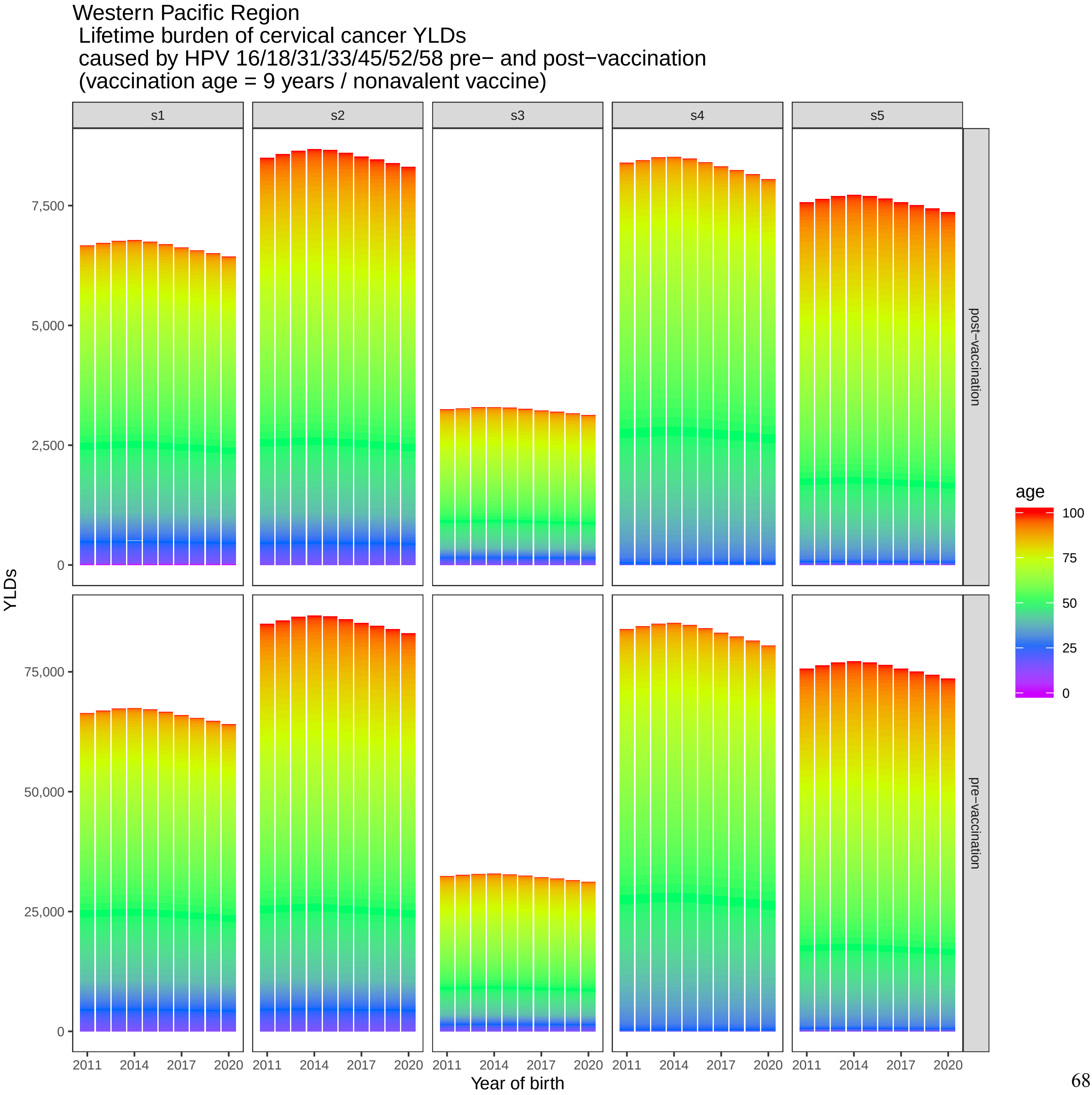
Western Pacific Region
Lifetime burden of cervical cancer YLDs
caused by HPV 16/18/31/33/45/52/58 pre− and post−vaccination
(vaccination age = 9 years / nonavalent vaccine)
s1
s2
s3
s4
s5
post−vaccination
pre−vaccination
YLDs
0
2,500
5,000
7,500
25,000
50,000
75,000
2011
2014
2017
2020
Year of birth
age
0
25
50
75
100

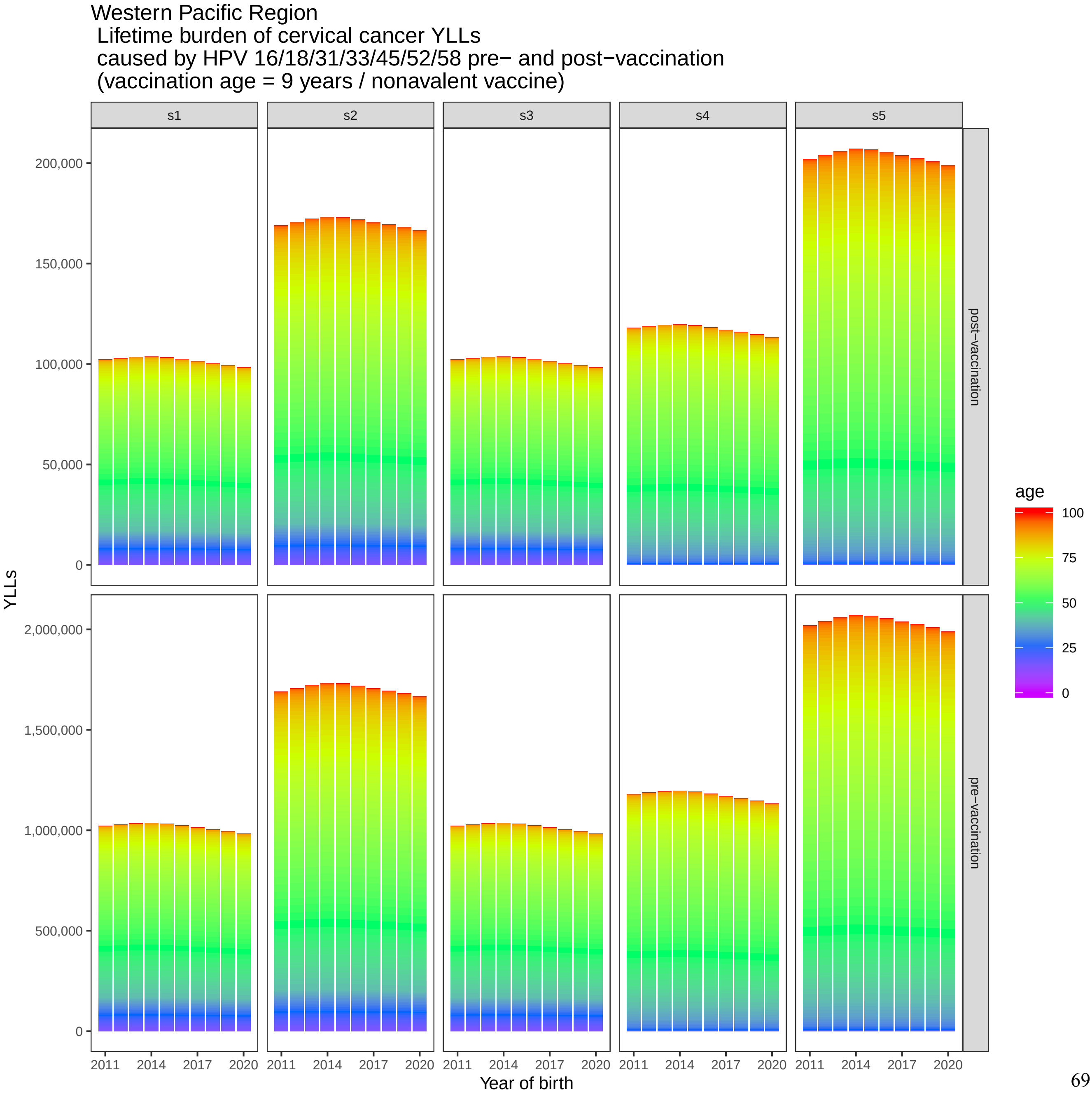
Western Pacific Region
Lifetime burden of cervical cancer YLLs
caused by HPV 16/18/31/33/45/52/58 pre− and post−vaccination
(vaccination age = 9 years / nonavalent vaccine)
s1
s2
s3
s4
s5
post−vaccination
pre−vaccination
YLLs
200,000
150,000
100,000
50,000
0
2,000,000
1,500,000
1,000,000
500,000
0
2011
2014
2017
2020
Year of birth
age
100
75
50
25
0

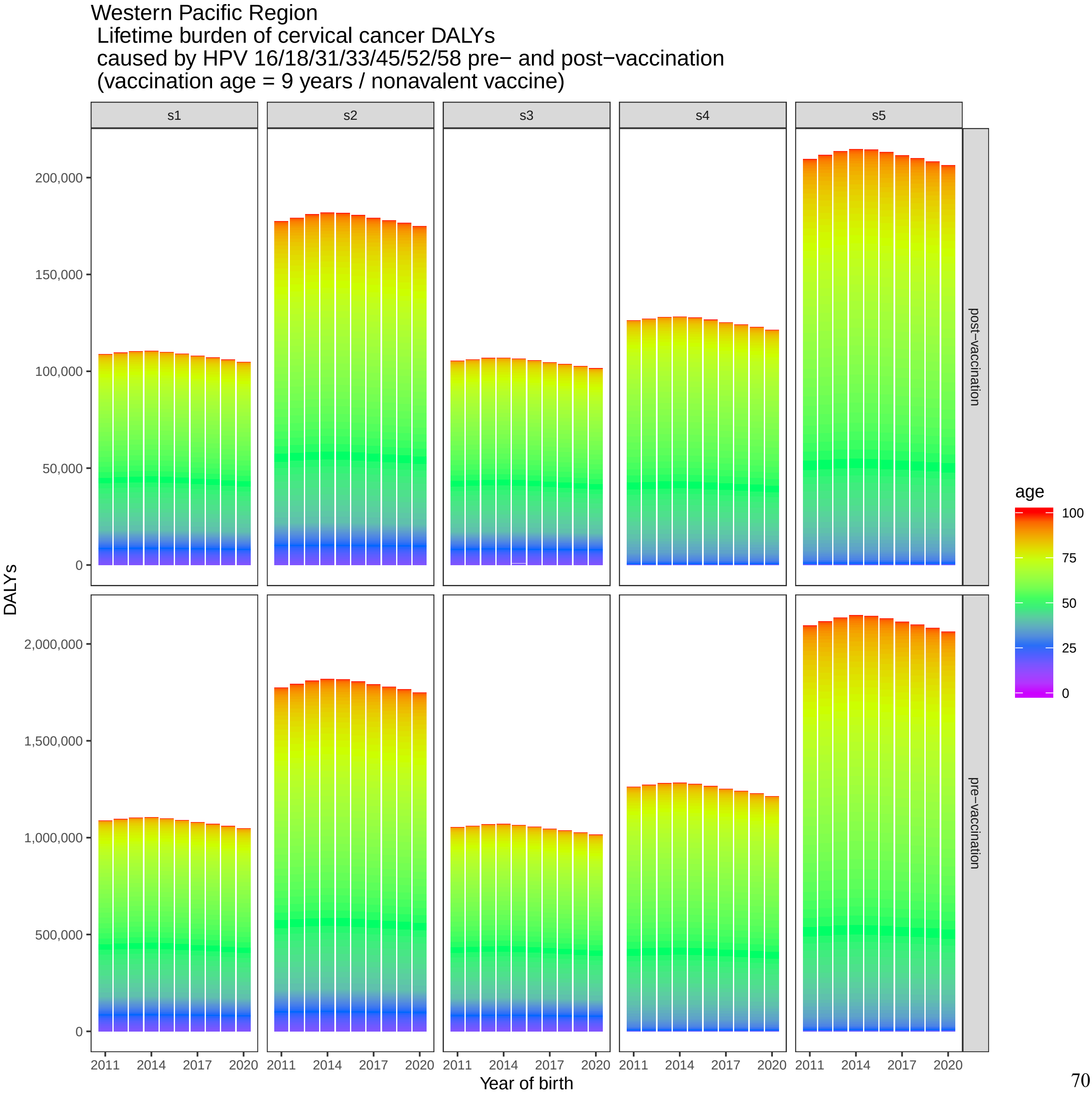

Western Pacific Region
Lifetime burden of cervical cancer DALYs
caused by HPV 16/18/31/33/45/52/58 pre− and post−vaccination
(vaccination age = 9 years / nonavalent vaccine)
s1
s2
s3
s4
s5
post−vaccination
pre−vaccination
DALYs
0
50,000
100,000
150,000
200,000
500,000
1,000,000
1,500,000
2,000,000
2011
2014
2017
2020
Year of birth
age
0
25
50
75
100

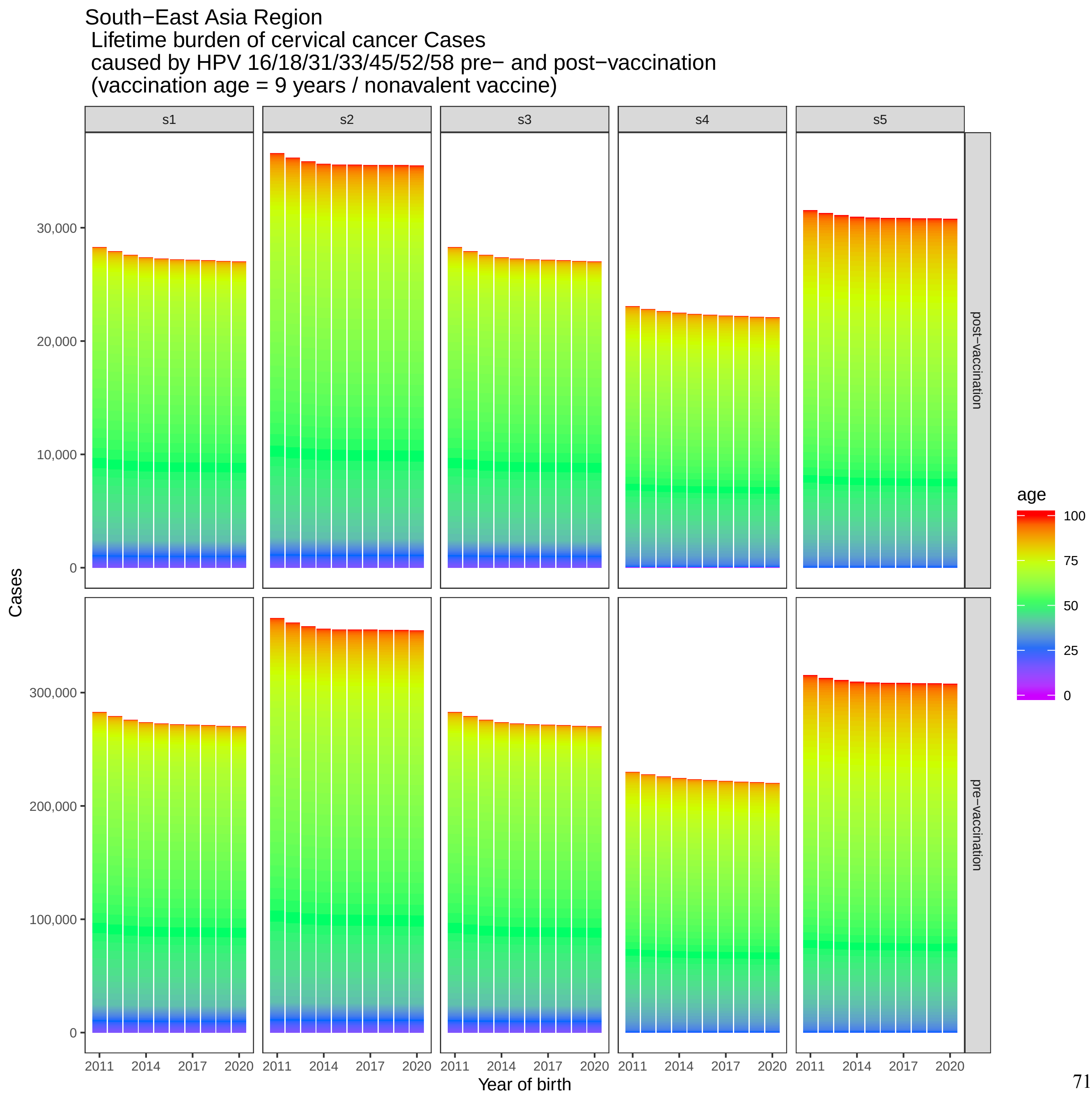

South−East Asia Region
Lifetime burden of cervical cancer Cases
caused by HPV 16/18/31/33/45/52/58 pre− and post−vaccination
(vaccination age = 9 years / nonavalent vaccine)
s1
s2
s3
s4
s5
post−vaccination
pre−vaccination
Cases
30,000
20,000
10,000
0
300,000
200,000
100,000
0
2011
2014
2017
2020
Year of birth
age
100
75
50
25
0

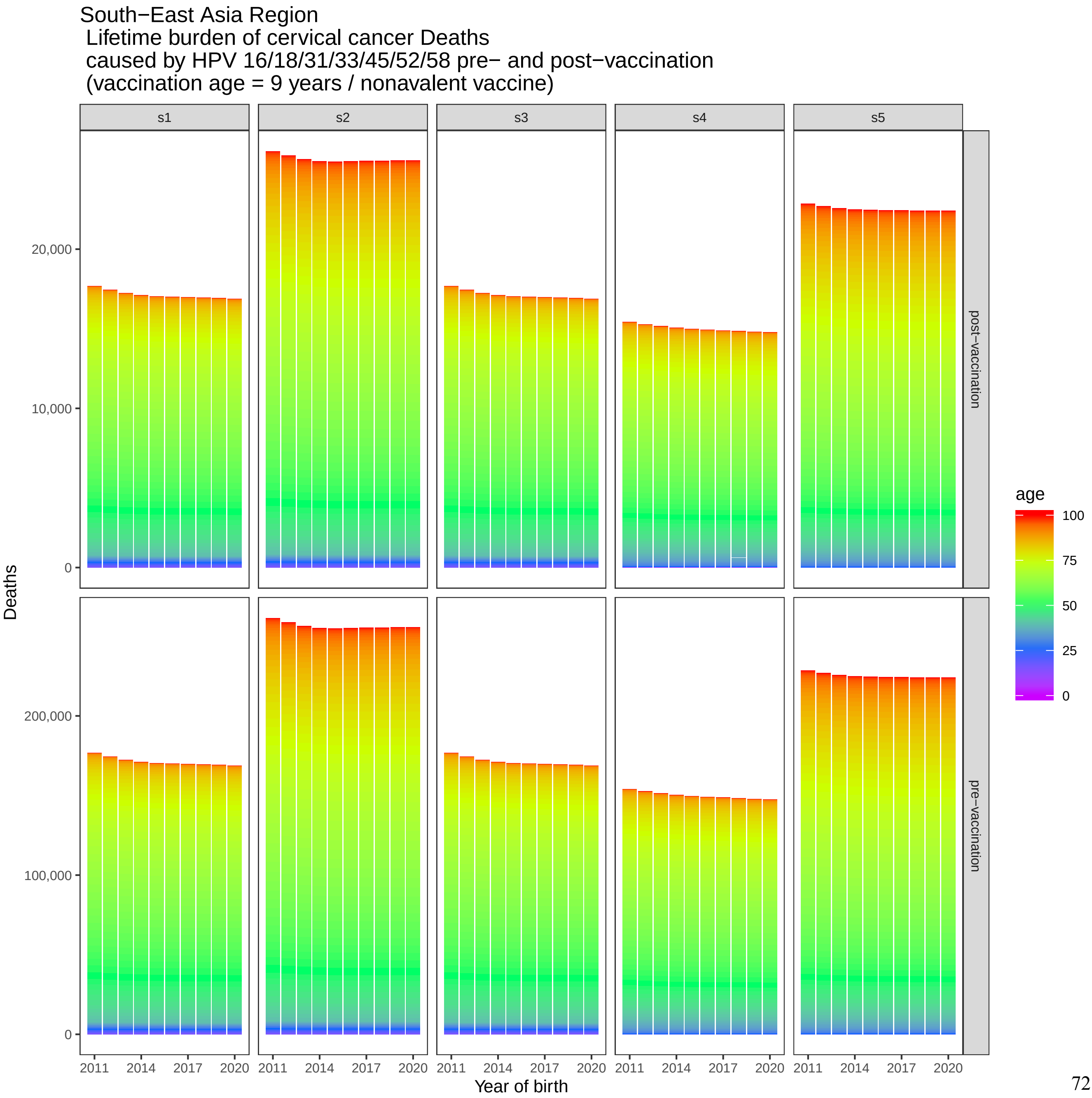

South−East Asia Region
Lifetime burden of cervical cancer Deaths
caused by HPV 16/18/31/33/45/52/58 pre− and post−vaccination
(vaccination age = 9 years / nonavalent vaccine)
s1
s2
s3
s4
s5
post−vaccination
pre−vaccination
Deaths
20,000
10,000
0
200,000
100,000
0
2011
2014
2017
2020
Year of birth
age
100
75
50
25
0

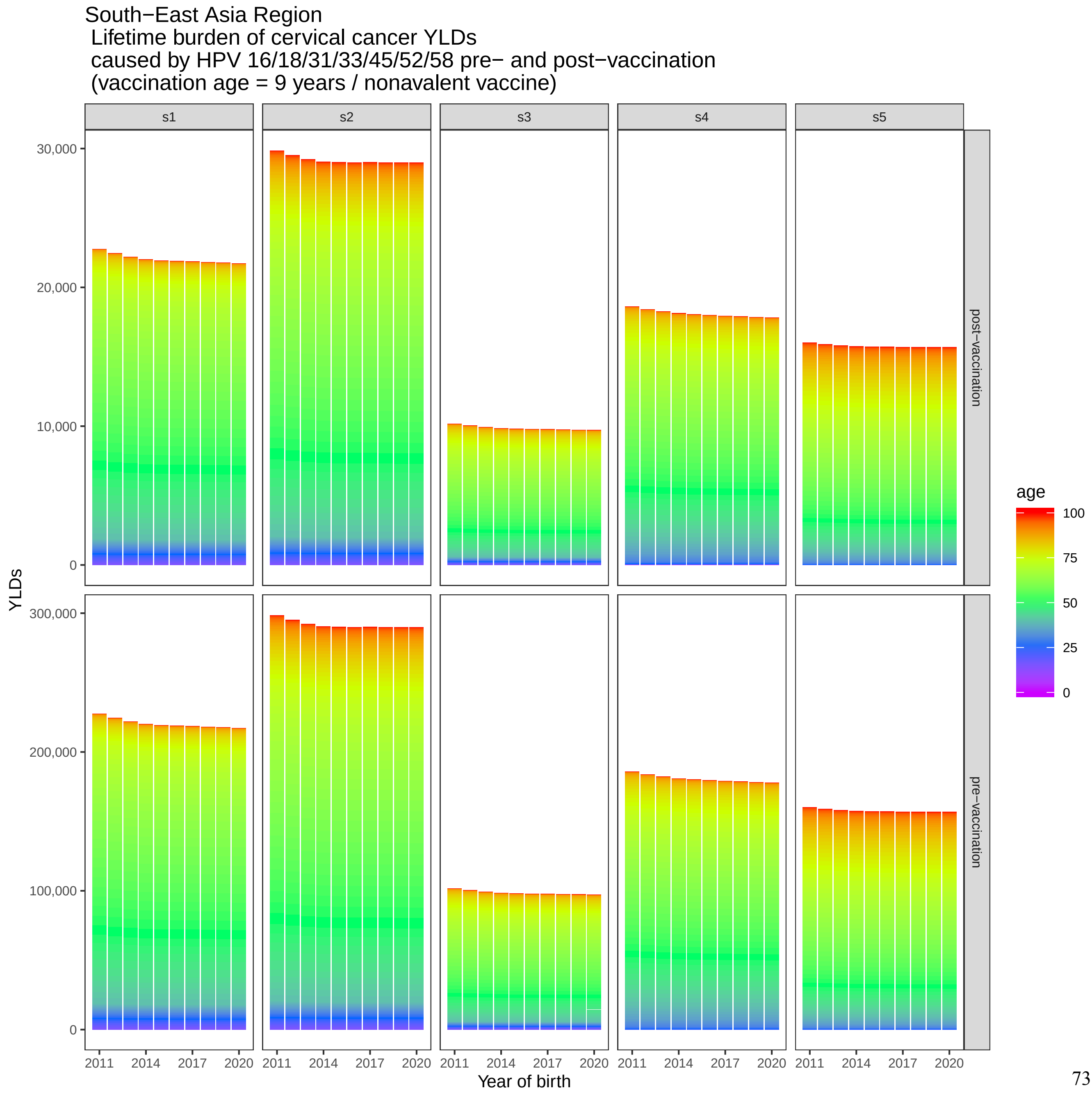

South−East Asia Region
Lifetime burden of cervical cancer YLDs
caused by HPV 16/18/31/33/45/52/58 pre− and post−vaccination
(vaccination age = 9 years / nonavalent vaccine)
s1
s2
s3
s4
s5
post−vaccination
pre−vaccination
YLDs
30,000
20,000
10,000
0
300,000
200,000
100,000
0
2011
2014
2017
2020
Year of birth
age
100
75
50
25
0

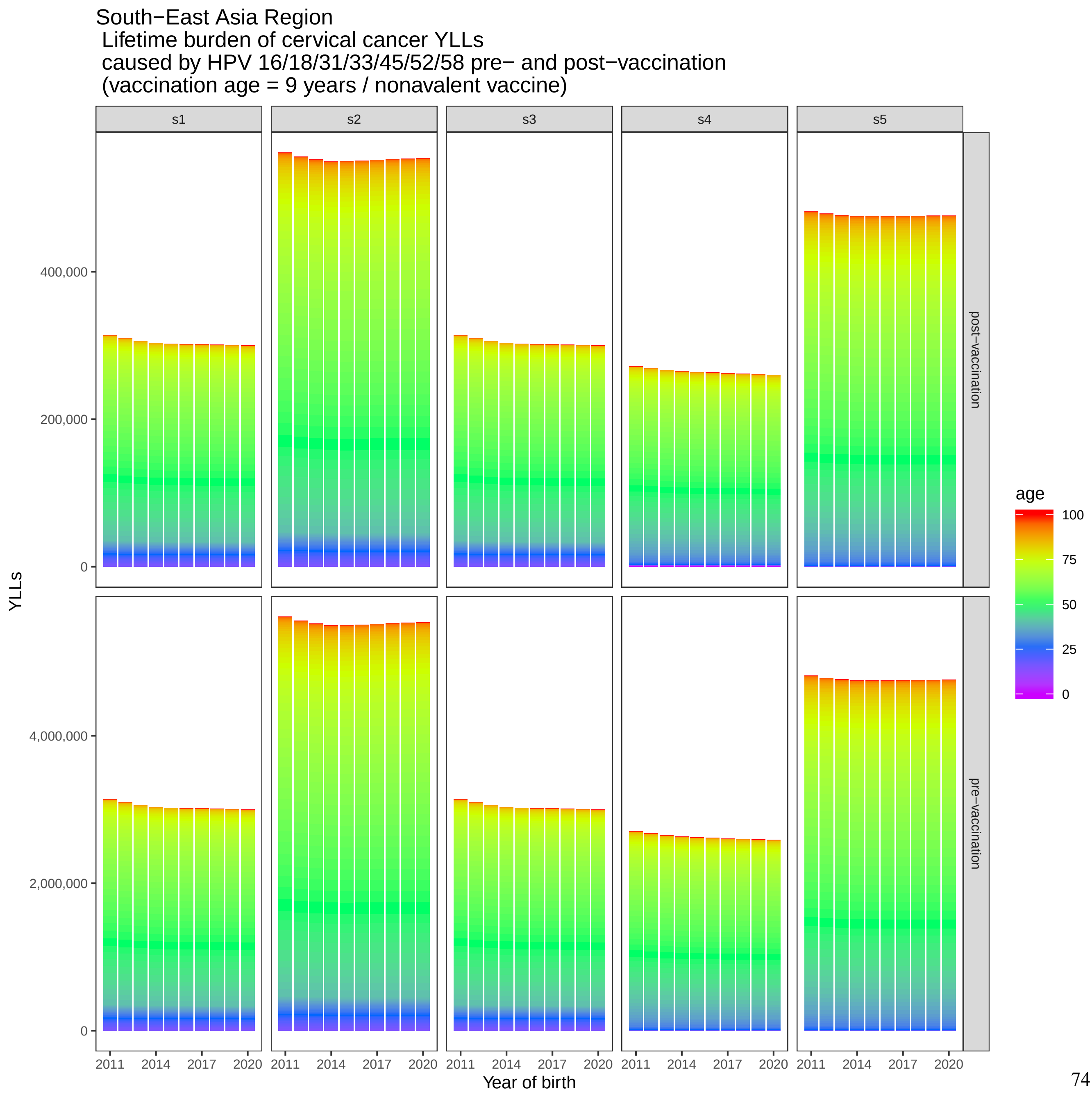
South−East Asia Region
Lifetime burden of cervical cancer YLLs
caused by HPV 16/18/31/33/45/52/58 pre− and post−vaccination
(vaccination age = 9 years / nonavalent vaccine)
s1
s2
s3
s4
s5
post−vaccination
pre−vaccination
YLLs
400,000
200,000
0
4,000,000
2,000,000
0
2011
2014
2017
2020
Year of birth
age
100
75
50
25
0

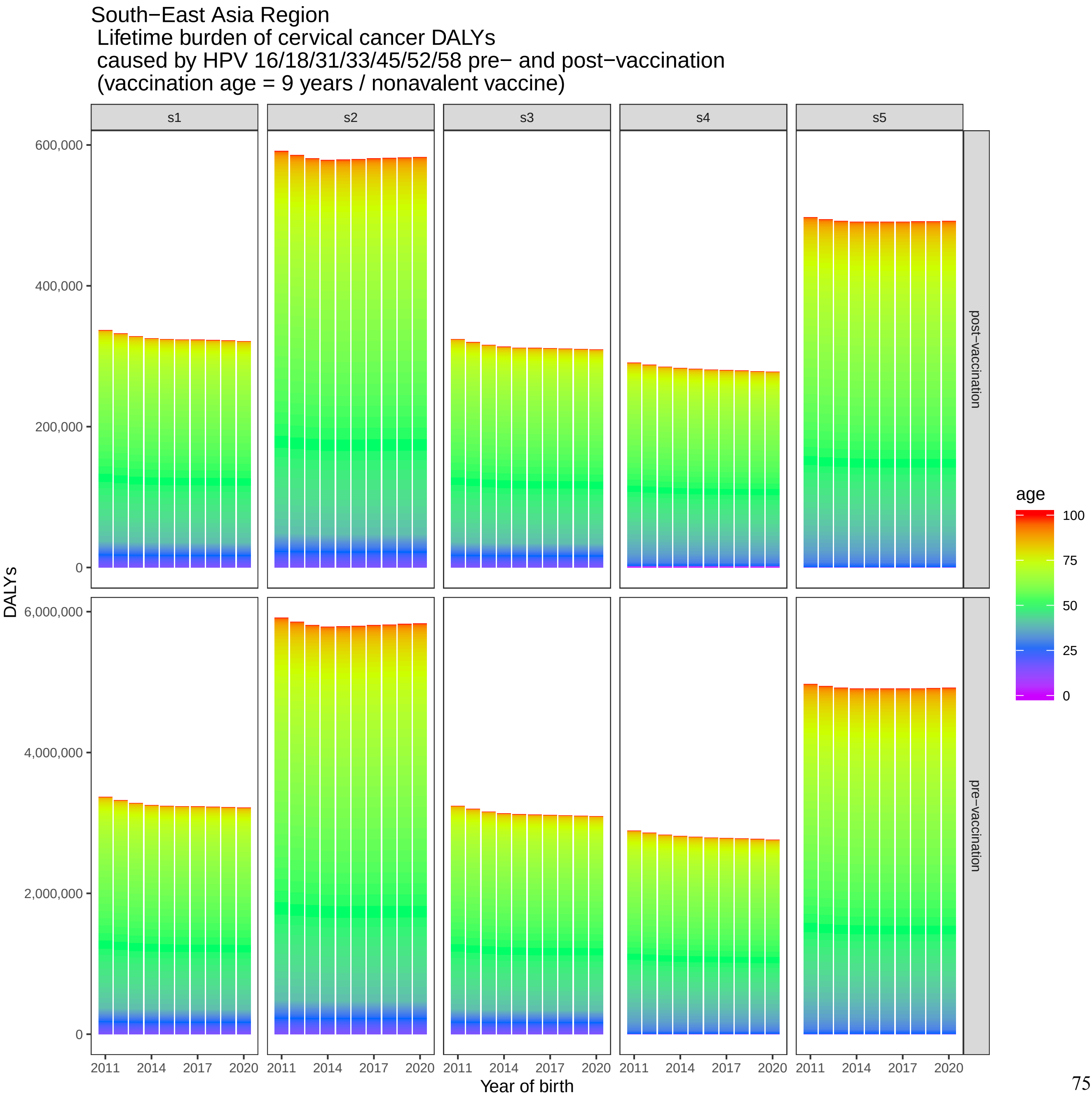

South−East Asia Region
Lifetime burden of cervical cancer DALYs
caused by HPV 16/18/31/33/45/52/58 pre− and post−vaccination
(vaccination age = 9 years / nonavalent vaccine)
s1
s2
s3
s4
s5
post−vaccination
pre−vaccination
DALYs
600,000
400,000
200,000
0
6,000,000
4,000,000
2,000,000
0
2011
2014
2017
2020
Year of birth
age
100
75
50
25
0

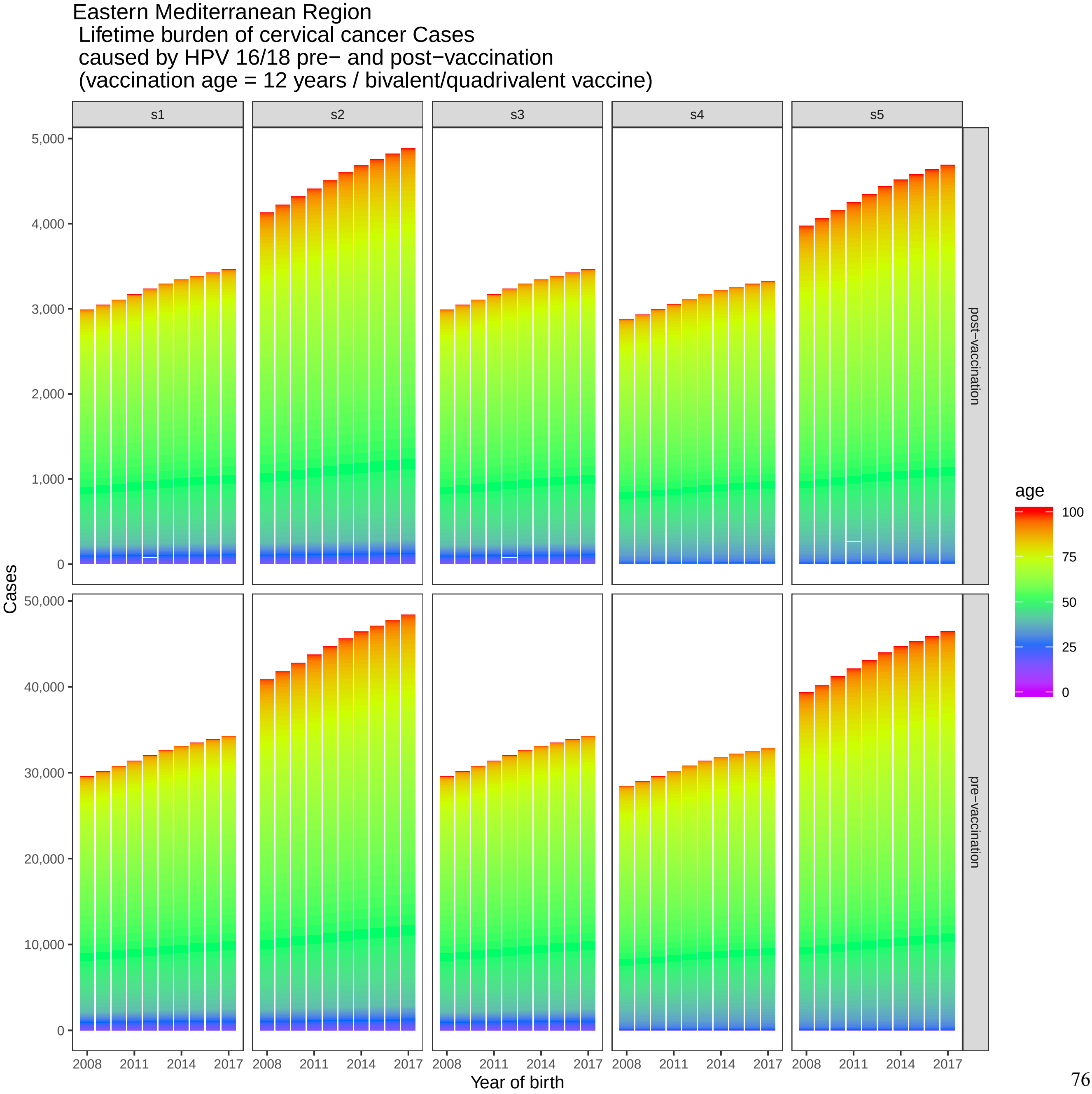
Eastern Mediterranean Region
Lifetime burden of cervical cancer Cases
caused by HPV 16/18 pre− and post−vaccination
(vaccination age = 12 years / bivalent/quadrivalent vaccine)
s1
s2
s3
s4
s5
post−vaccination
pre−vaccination
Cases
Year of birth
age
0
25
50
75
100
5,000
4,000
3,000
2,000
1,000
50,000
40,000
30,000
20,000
10,000
2008
2011
2014
2017

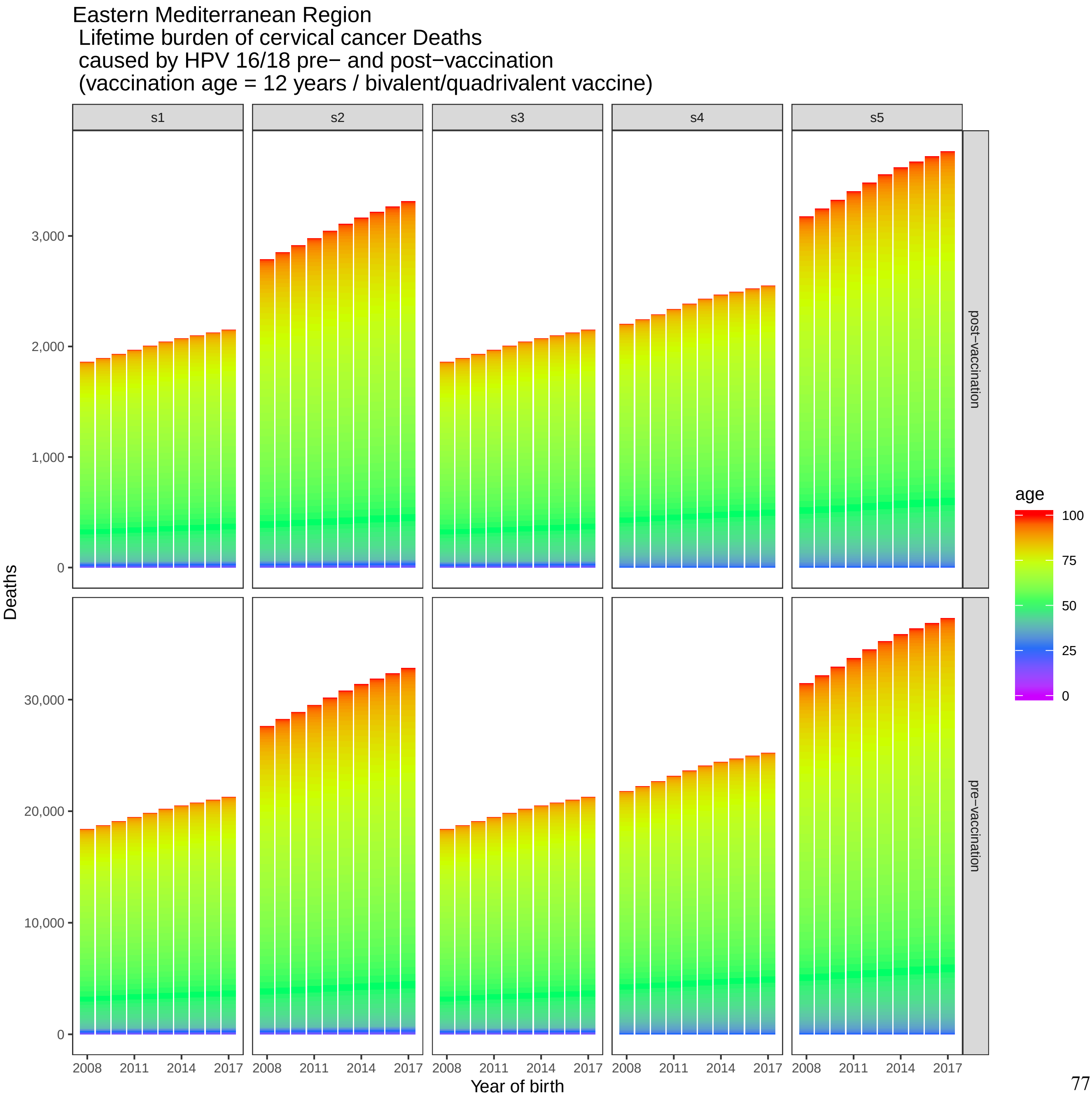
Eastern Mediterranean Region
Lifetime burden of cervical cancer Deaths
caused by HPV 16/18 pre− and post−vaccination
(vaccination age = 12 years / bivalent/quadrivalent vaccine)
s1
s2
s3
s4
s5
post−vaccination
pre−vaccination
Deaths
Year of birth
age
0
1,000
2,000
3,000
10,000
20,000
30,000
2008
2011
2014
2017
100
75
50
25
0

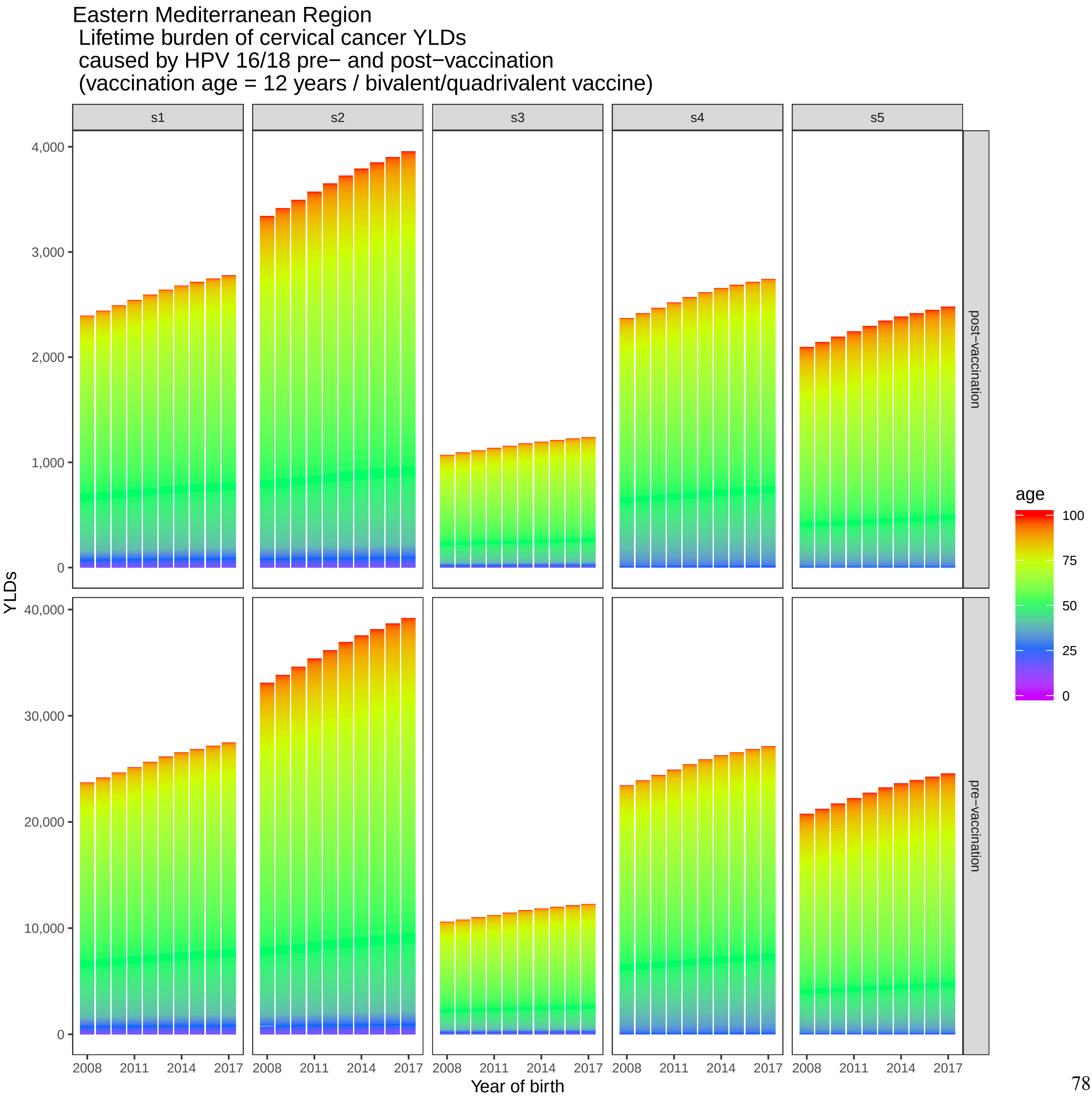
Eastern Mediterranean Region
Lifetime burden of cervical cancer YLDs
caused by HPV 16/18 pre− and post−vaccination
(vaccination age = 12 years / bivalent/quadrivalent vaccine)
s1
s2
s3
s4
s5
post−vaccination
pre−vaccination
YLDs
Year of birth
age
0
25
50
75
100
0
1,000
2,000
3,000
4,000
10,000
20,000
30,000
40,000
2008
2011
2014
2017

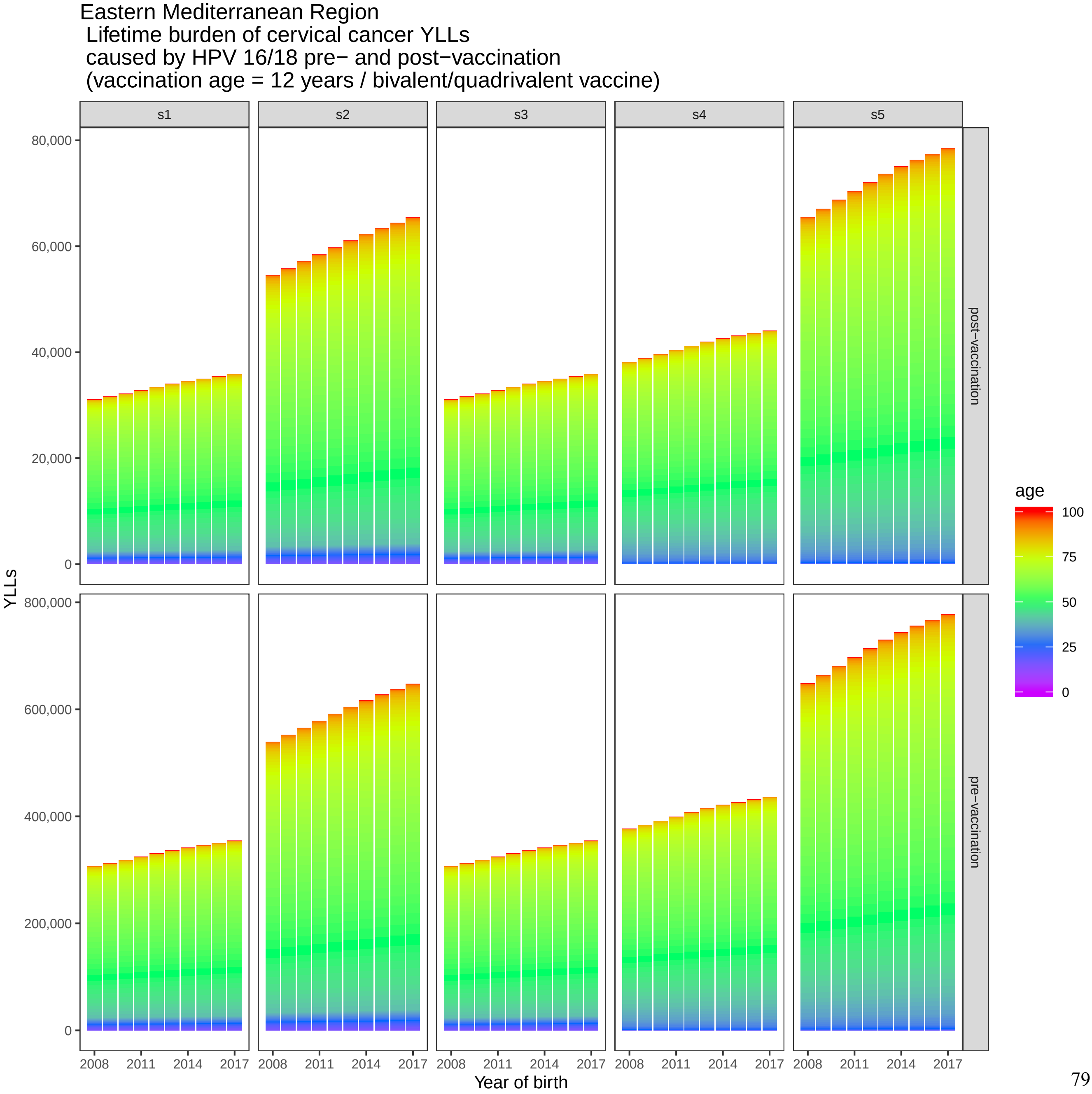
Eastern Mediterranean Region
Lifetime burden of cervical cancer YLLs
caused by HPV 16/18 pre− and post−vaccination
(vaccination age = 12 years / bivalent/quadrivalent vaccine)
s1
s2
s3
s4
s5
post−vaccination
pre−vaccination
YLLs
0
20,000
40,000
60,000
80,000
200,000
400,000
600,000
800,000
2008
2011
2014
2017
Year of birth
age
0
25
50
75
100

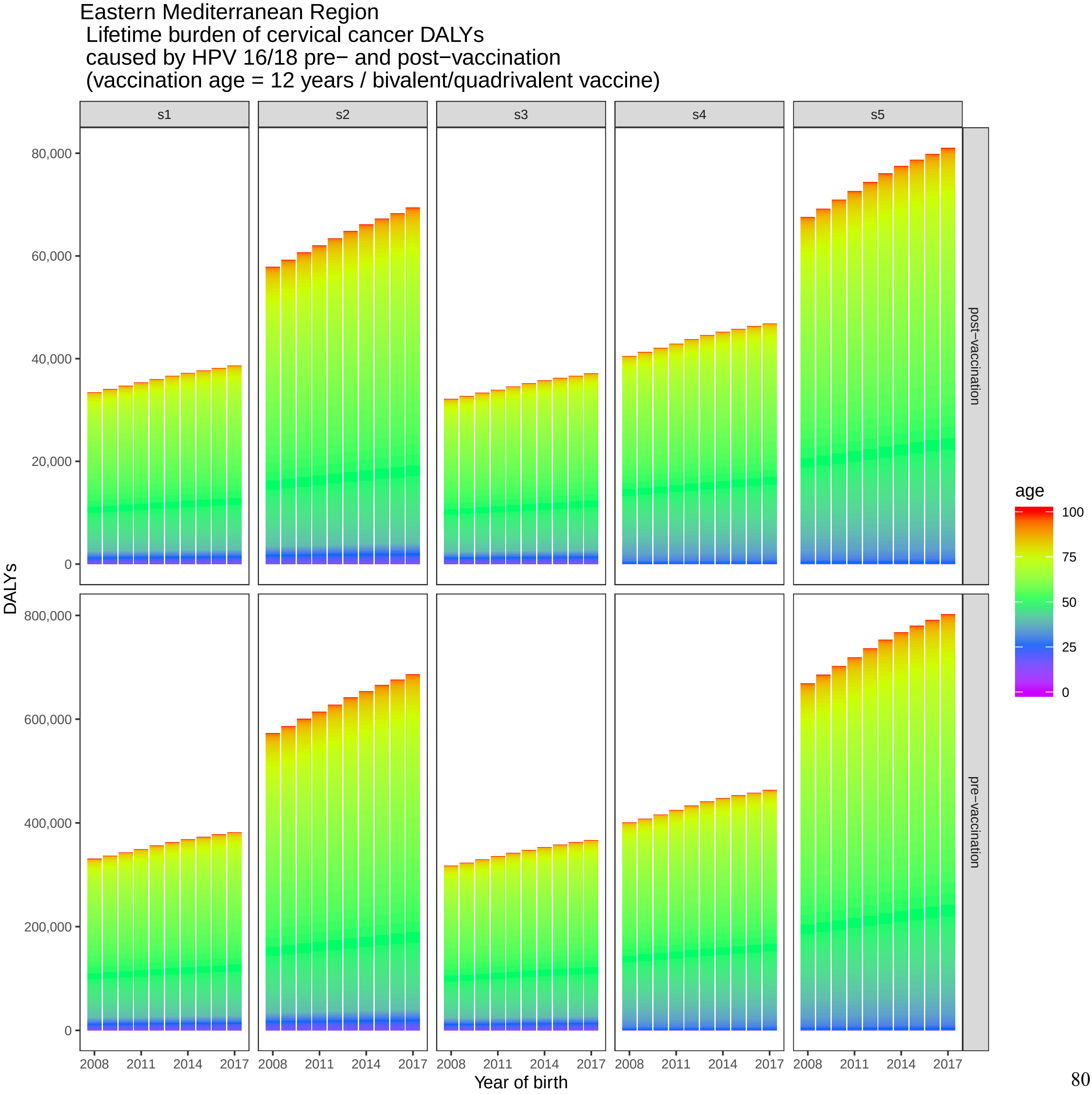
Eastern Mediterranean Region
Lifetime burden of cervical cancer DALYs
caused by HPV 16/18 pre− and post−vaccination
(vaccination age = 12 years / bivalent/quadrivalent vaccine)
s1
s2
s3
s4
s5
post−vaccination
pre−vaccination
DALYs
80,000
60,000
40,000
20,000
0
800,000
600,000
400,000
200,000
0
2008
2011
2014
2017
Year of birth
age
100
75
50
25
0

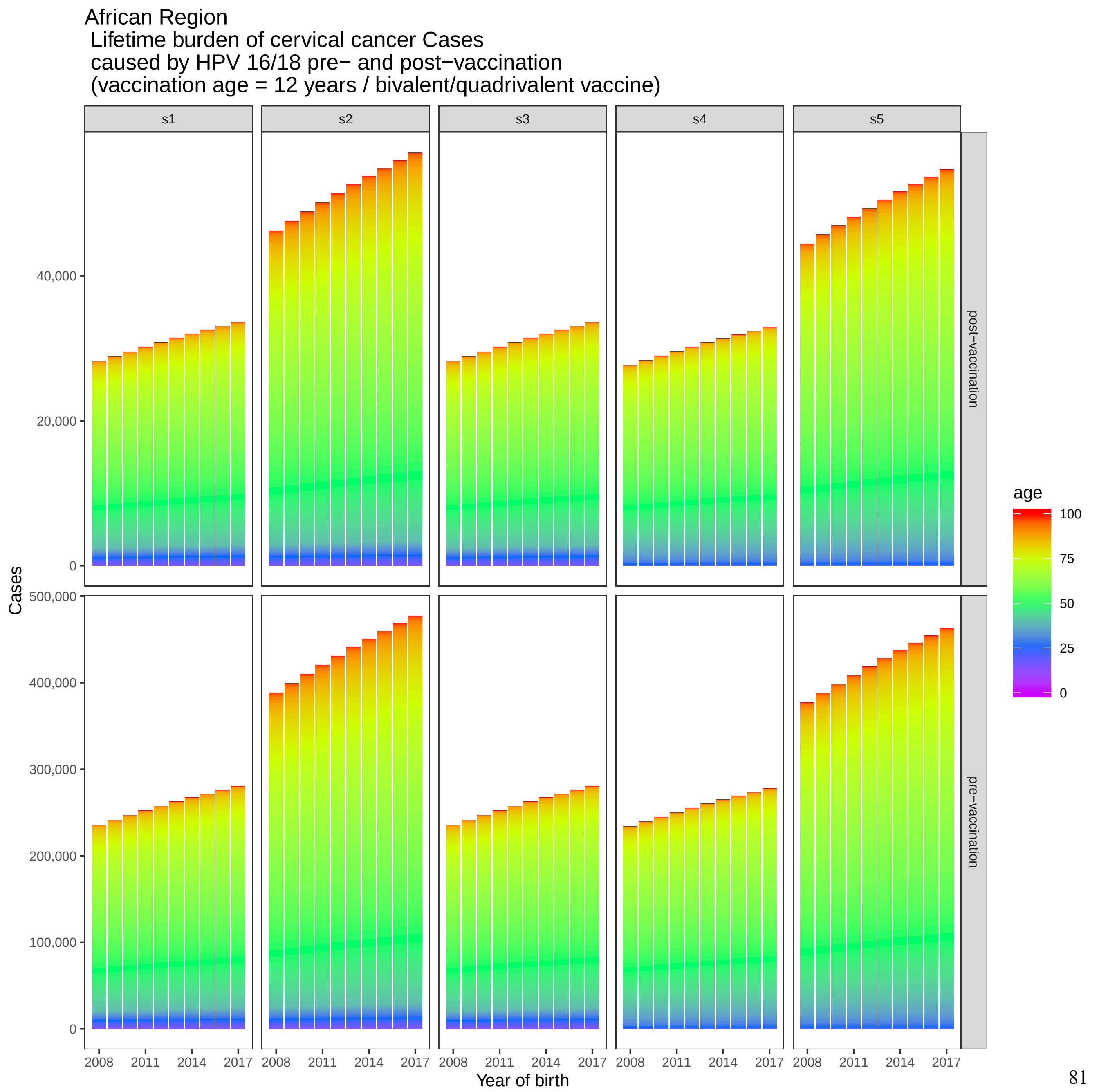

African Region
Lifetime burden of cervical cancer Cases
caused by HPV 16/18 pre− and post−vaccination
(vaccination age = 12 years / bivalent/quadrivalent vaccine)
s1
s2
s3
s4
s5
post−vaccination
pre−vaccination
Cases
0
20,000
40,000
100,000
200,000
300,000
400,000
500,000
2008
2011
2014
2017
Year of birth
age
0
25
50
75
100

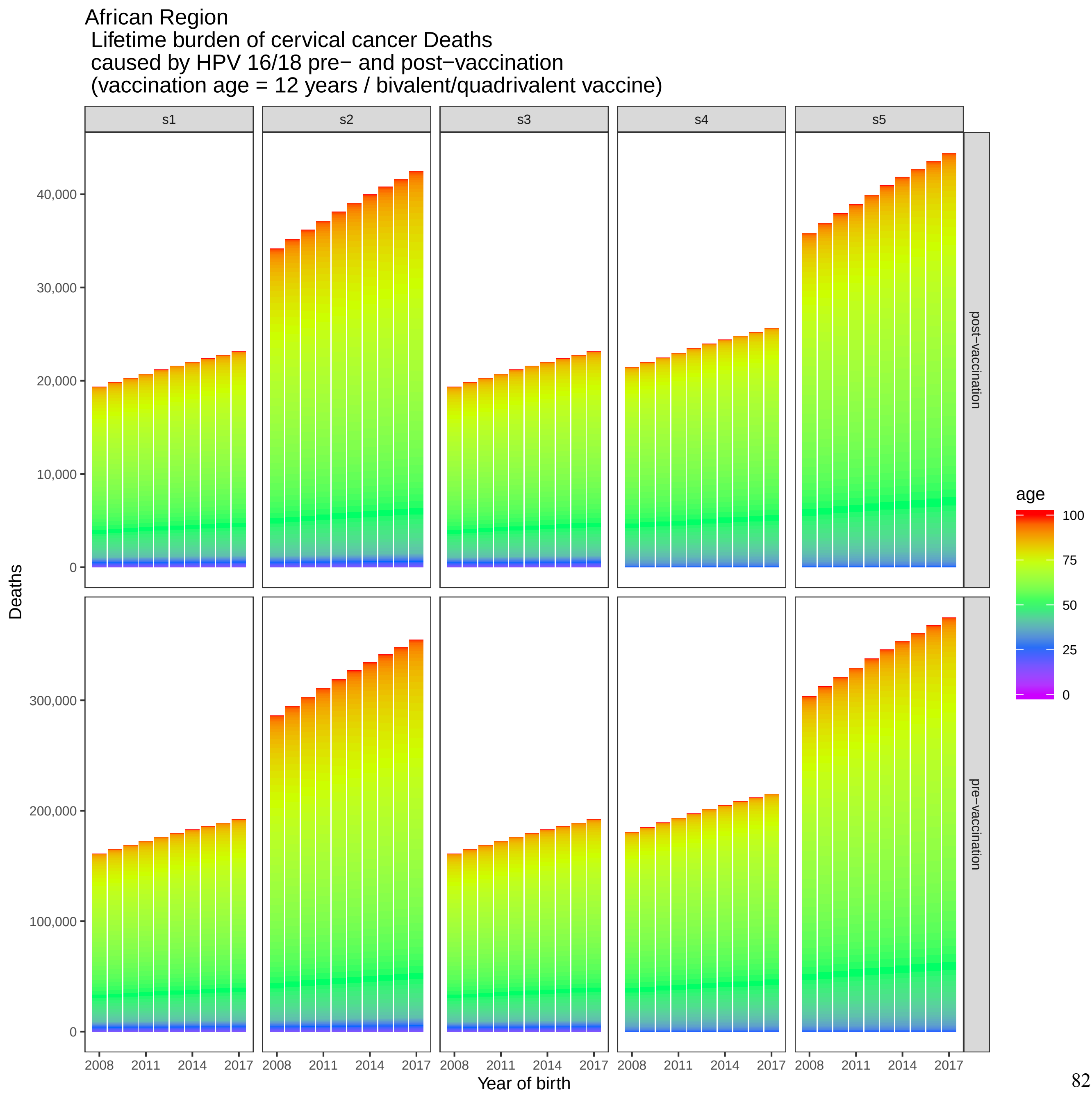
African Region
Lifetime burden of cervical cancer Deaths
caused by HPV 16/18 pre− and post−vaccination
(vaccination age = 12 years / bivalent/quadrivalent vaccine)
s1
s2
s3
s4
s5
post−vaccination
pre−vaccination
Deaths
0
10,000
20,000
30,000
40,000
100,000
200,000
300,000
Year of birth
2008
2011
2014
2017
age
0
25
50
75
100

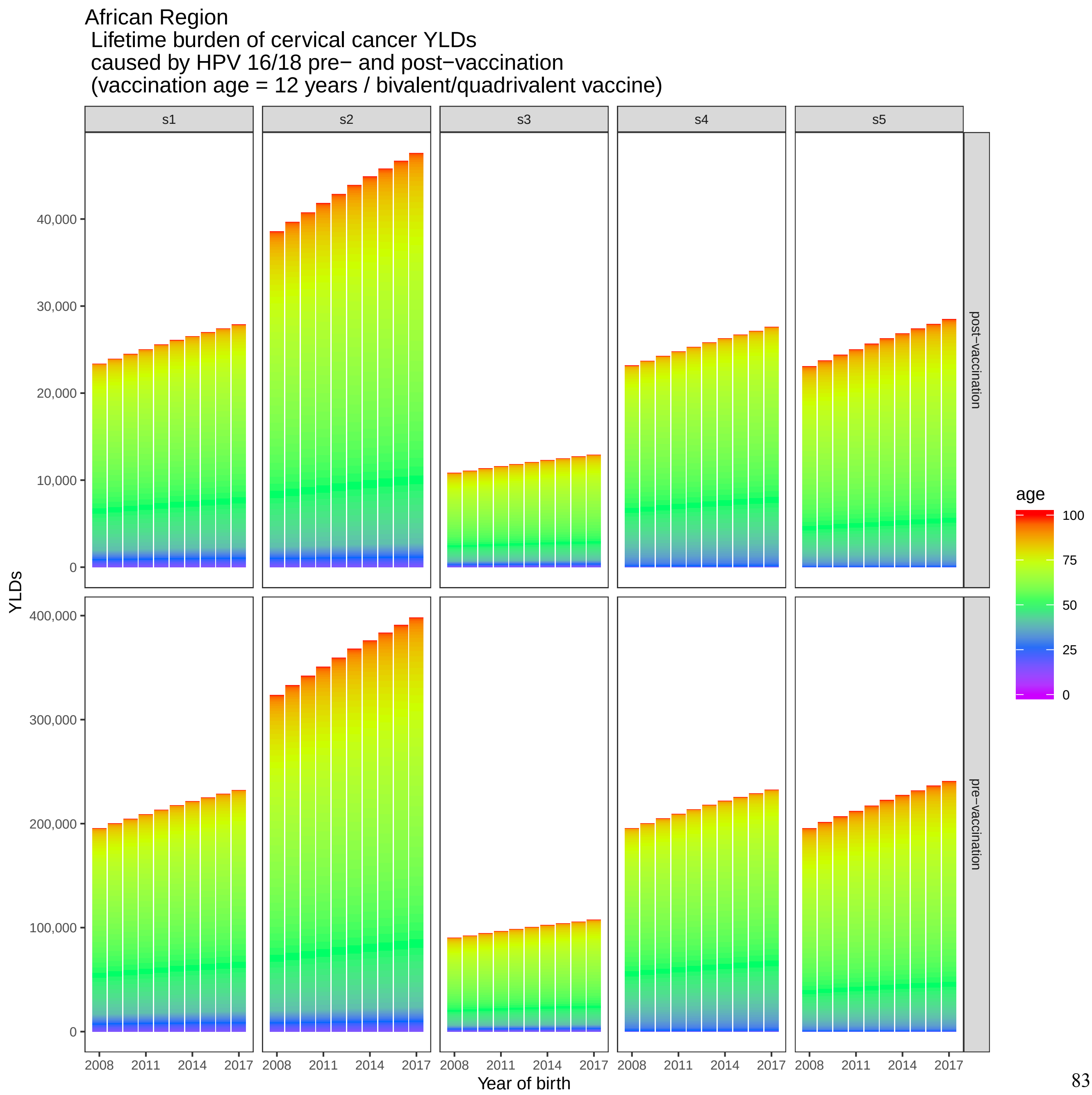

African Region
Lifetime burden of cervical cancer YLDs
caused by HPV 16/18 pre− and post−vaccination
(vaccination age = 12 years / bivalent/quadrivalent vaccine)
s1
s2
s3
s4
s5
post−vaccination
pre−vaccination
YLDs
40,000
30,000
20,000
10,000
0
400,000
300,000
200,000
100,000
0
2008
2011
2014
2017
Year of birth
age
100
75
50
25
0

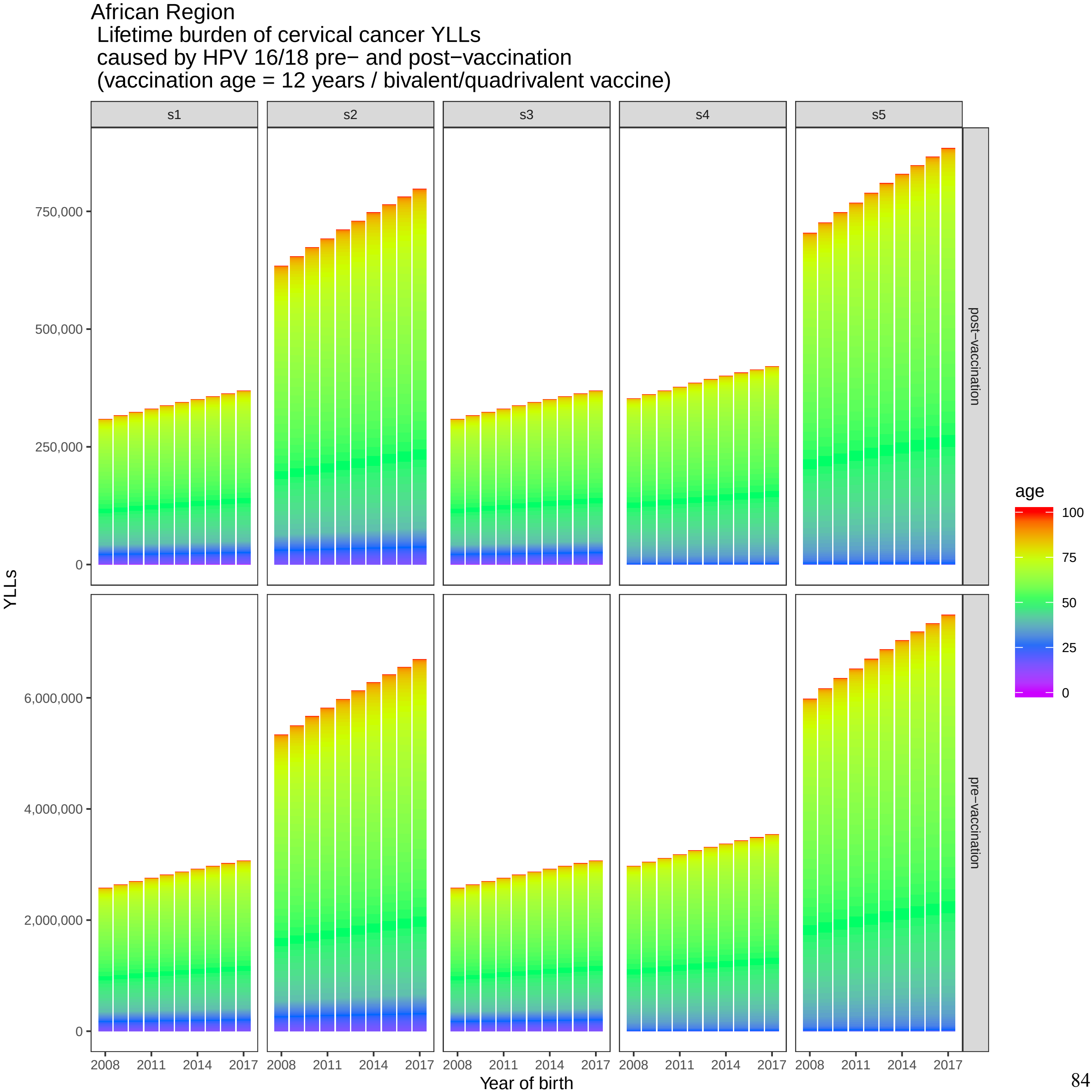

African Region
Lifetime burden of cervical cancer YLLs
caused by HPV 16/18 pre− and post−vaccination
(vaccination age = 12 years / bivalent/quadrivalent vaccine)
s1
s2
s3
s4
s5
post−vaccination
pre−vaccination
750,000
500,000
250,000
0
6,000,000
4,000,000
2,000,000
0
YLLs
2008
2011
2014
2017
Year of birth
age
100
75
50
25
0

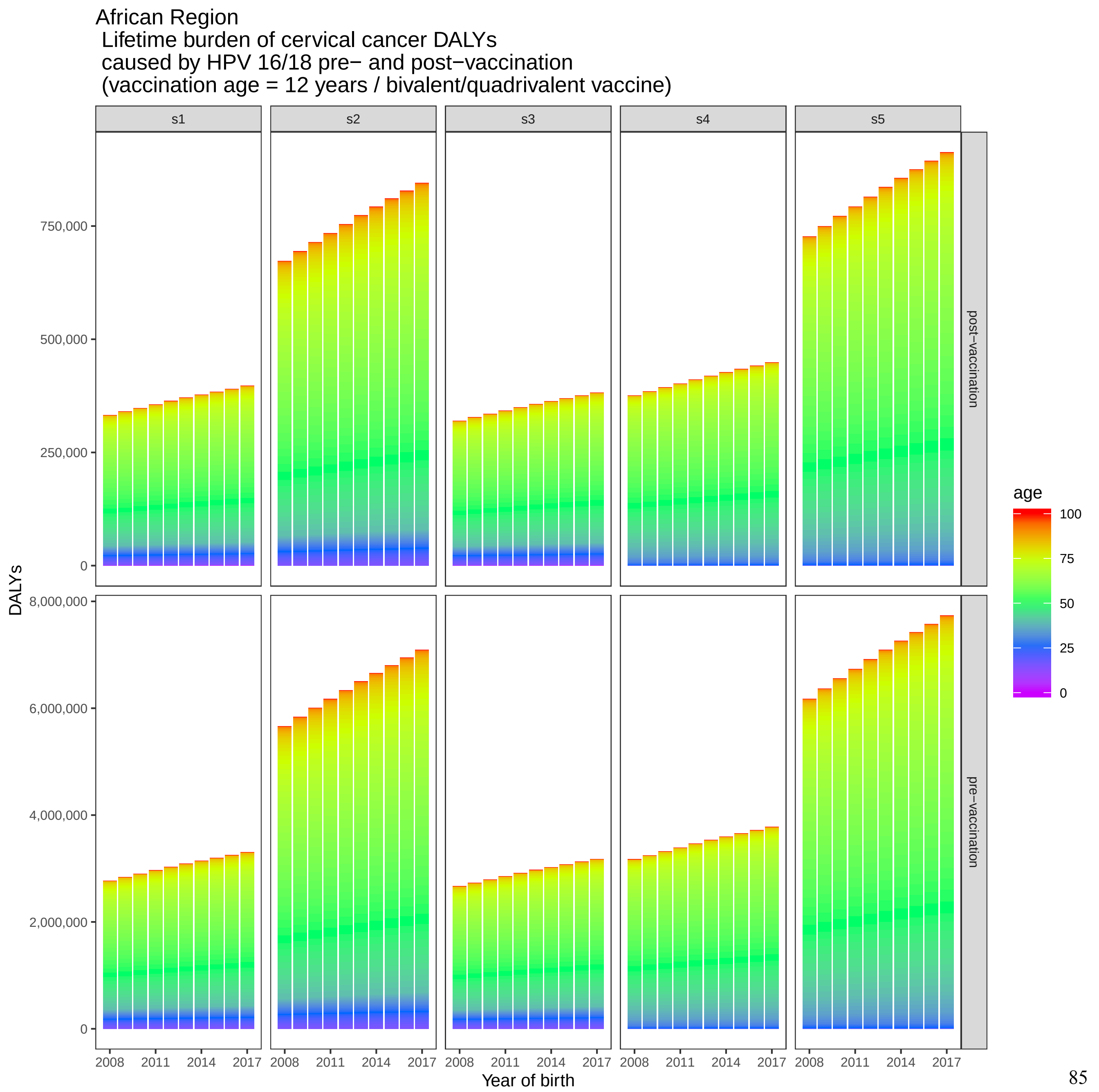
African Region
Lifetime burden of cervical cancer DALYs
caused by HPV 16/18 pre− and post−vaccination
(vaccination age = 12 years / bivalent/quadrivalent vaccine)
s1
s2
s3
s4
s5
post−vaccination
pre−vaccination
DALYs
750,000
500,000
250,000
0
8,000,000
6,000,000
4,000,000
2,000,000
0
2008
2011
2014
2017
Year of birth
age
100
75
50
25
0

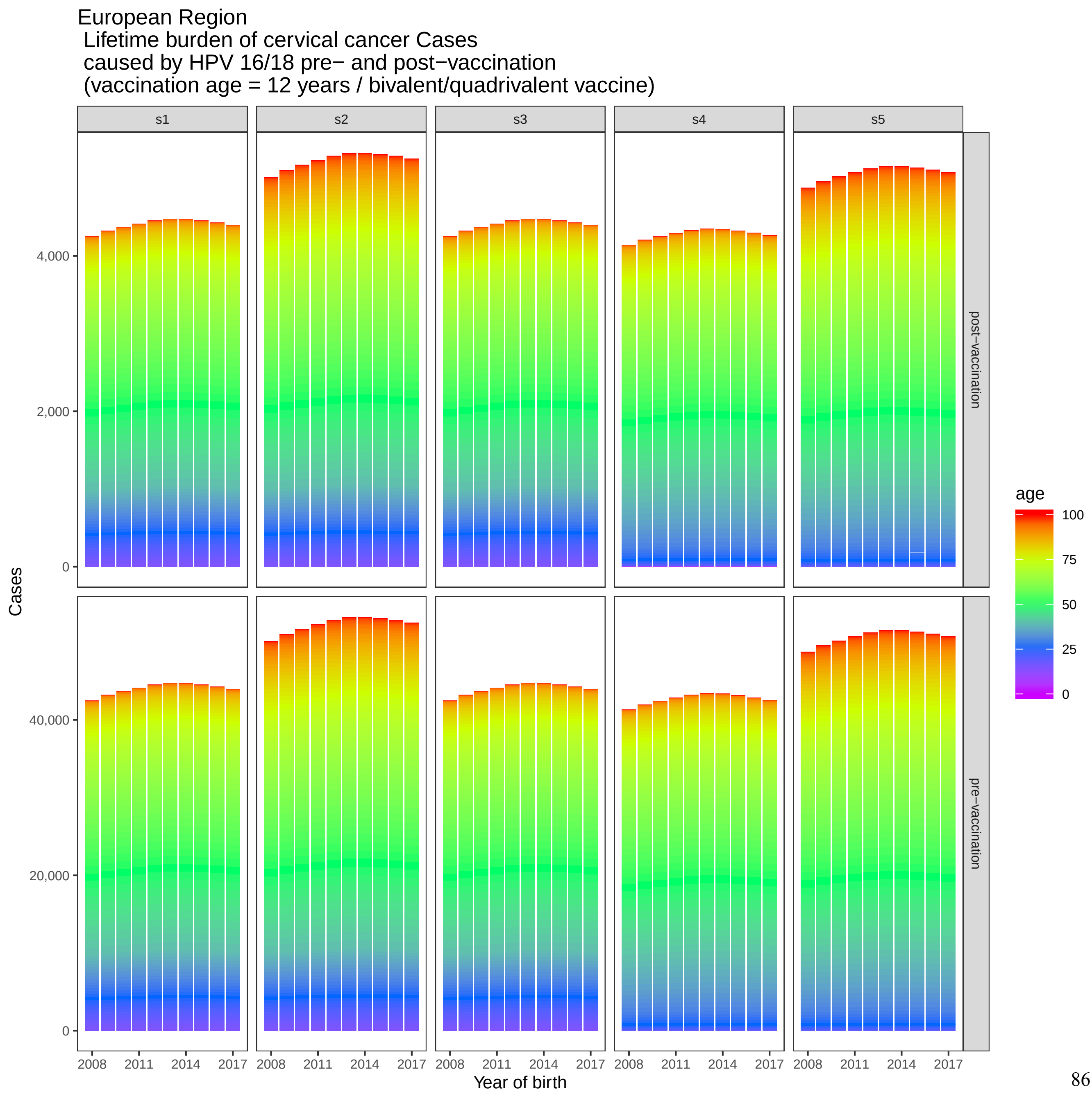

European Region
Lifetime burden of cervical cancer Cases
caused by HPV 16/18 pre− and post−vaccination
(vaccination age = 12 years / bivalent/quadrivalent vaccine)
s1
s2
s3
s4
s5
post−vaccination
pre−vaccination
Cases
4,000
2,000
0
40,000
20,000
0
Year of birth
2008
2011
2014
2017
age
100
75
50
25
0

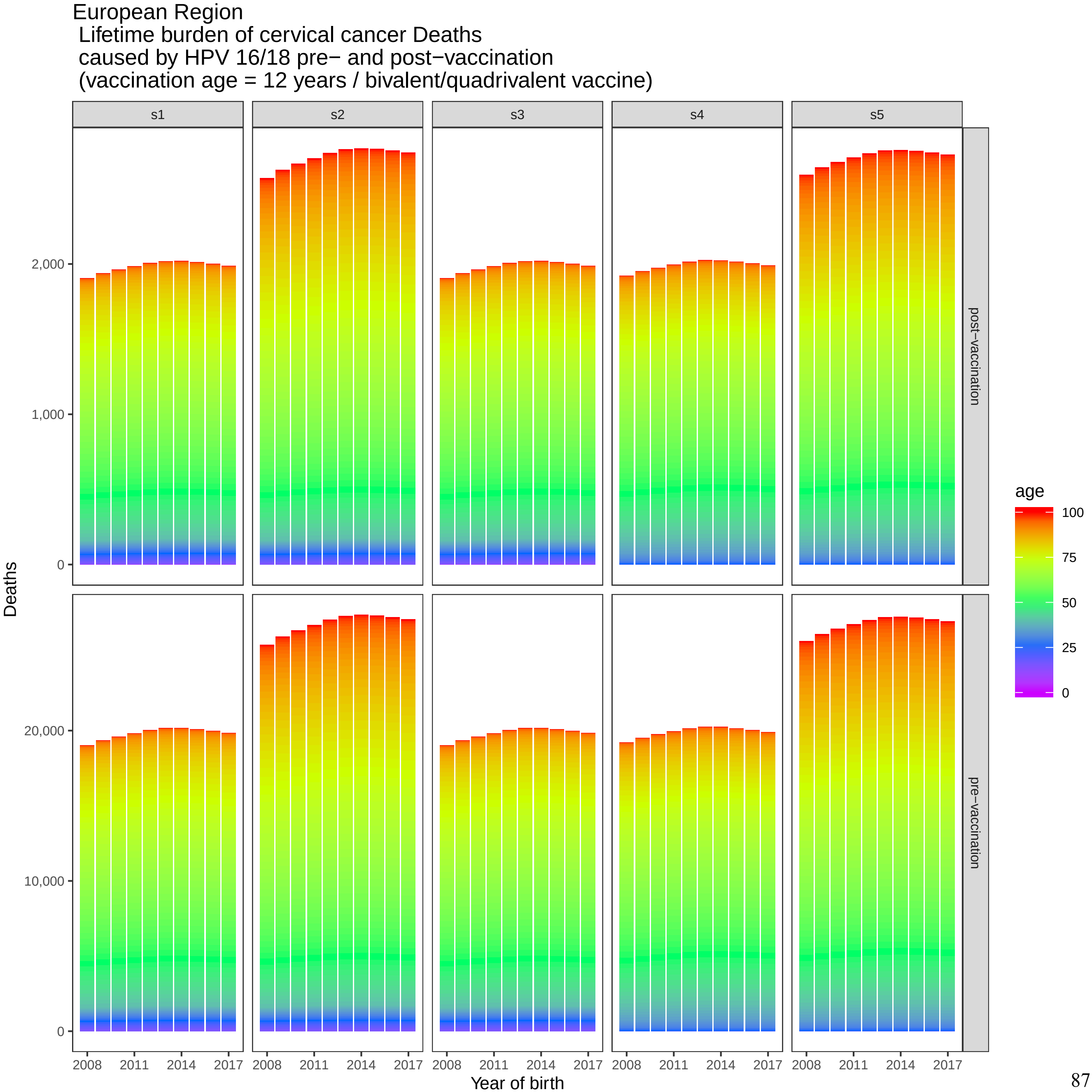
European Region
Lifetime burden of cervical cancer Deaths
caused by HPV 16/18 pre− and post−vaccination
(vaccination age = 12 years / bivalent/quadrivalent vaccine)
s1
s2
s3
s4
s5
post−vaccination
pre−vaccination
Deaths
2,000
1,000
0
20,000
10,000
0
2008
2011
2014
2017
Year of birth
age
100
75
50
25
0

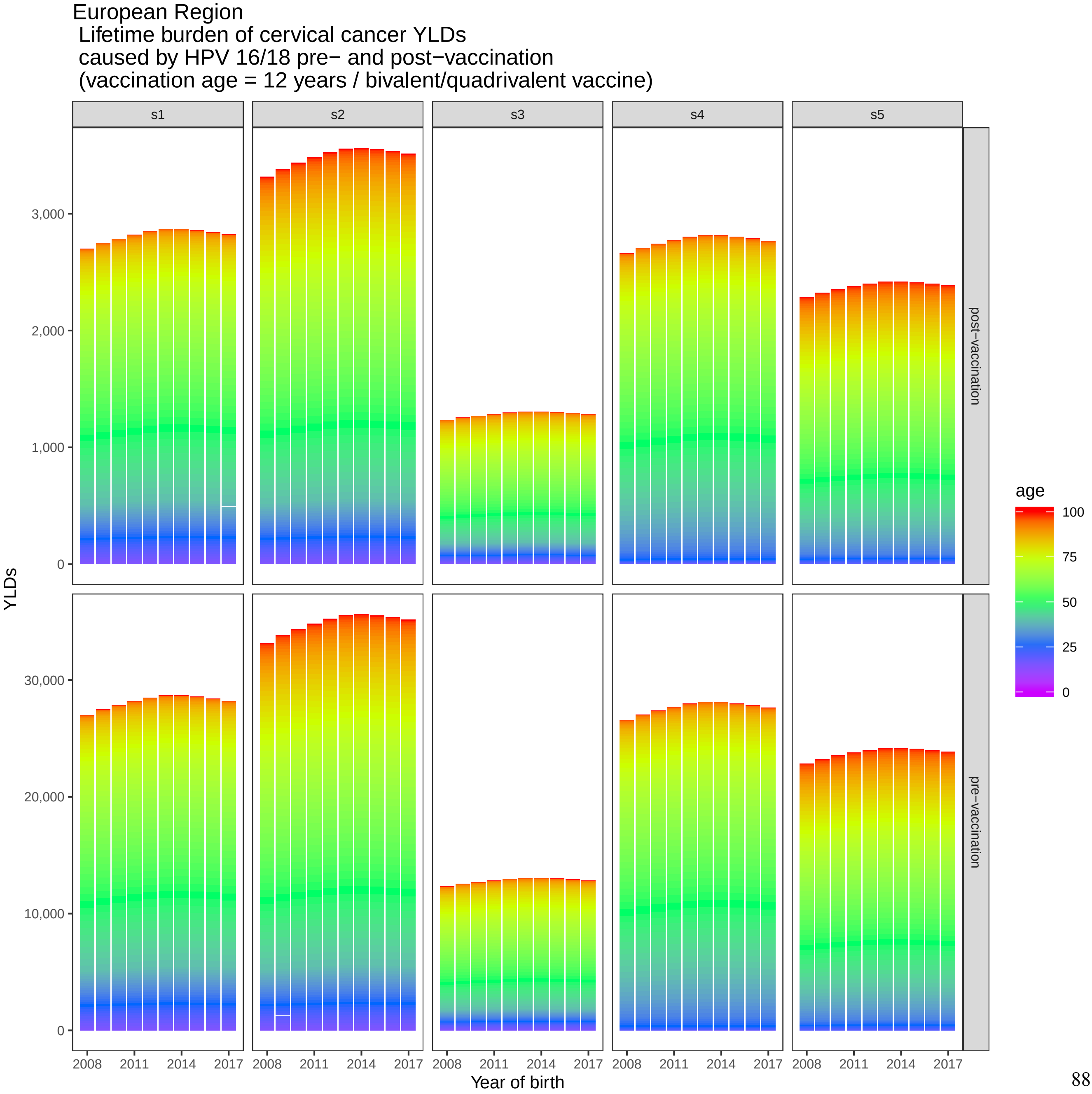
European Region
Lifetime burden of cervical cancer YLDs
caused by HPV 16/18 pre− and post−vaccination
(vaccination age = 12 years / bivalent/quadrivalent vaccine)
s1
s2
s3
s4
s5
post−vaccination
pre−vaccination
YLDs
Year of birth
age
0
25
50
75
100
0
1,000
2,000
3,000
10,000
20,000
30,000
2008
2011
2014
2017

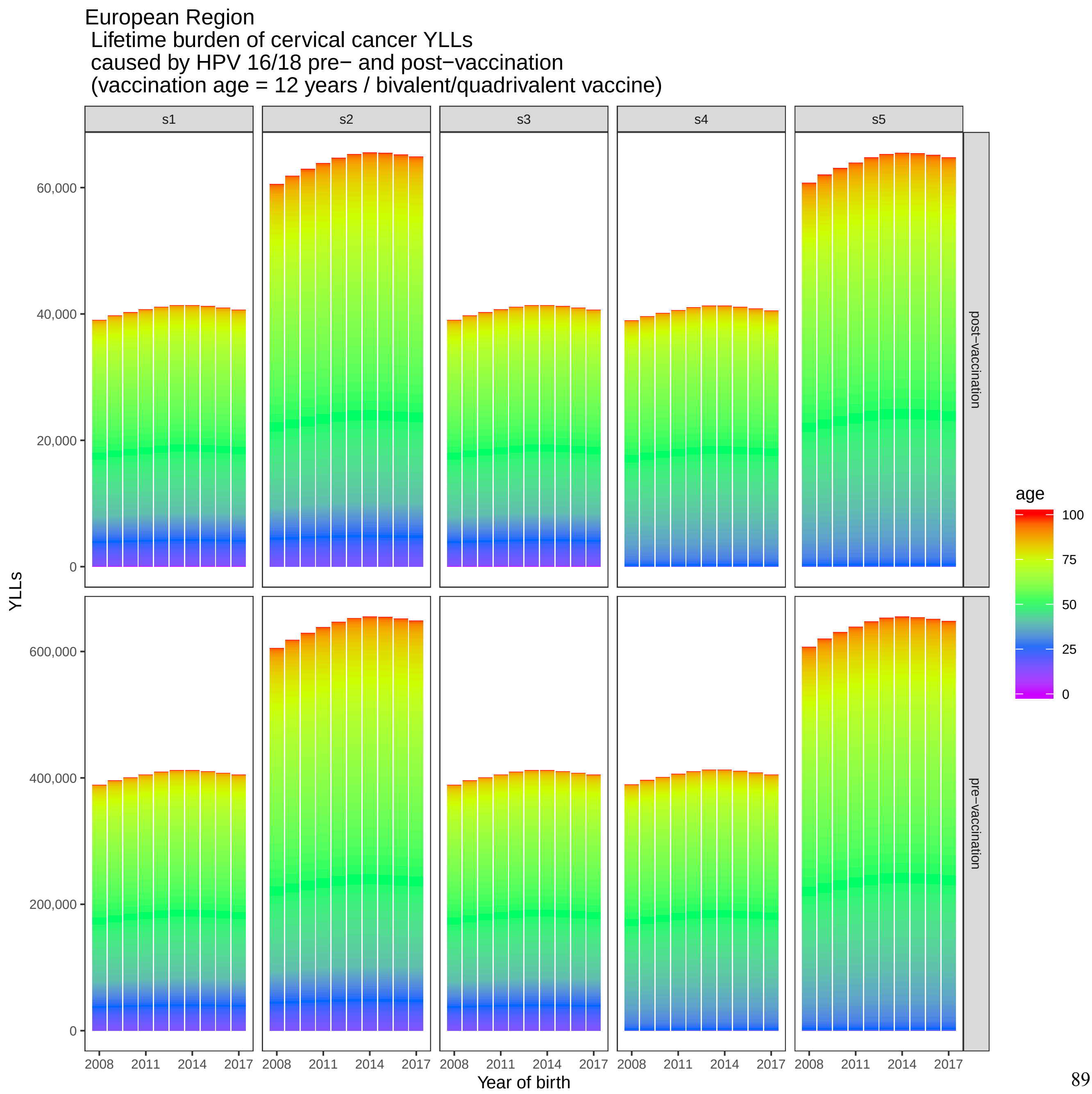

European Region
Lifetime burden of cervical cancer YLLs
caused by HPV 16/18 pre− and post−vaccination
(vaccination age = 12 years / bivalent/quadrivalent vaccine)
s1
s2
s3
s4
s5
post−vaccination
pre−vaccination
YLLs
60,000
40,000
20,000
0
600,000
400,000
200,000
0
2008
2011
2014
2017
Year of birth
age
100
75
50
25
0

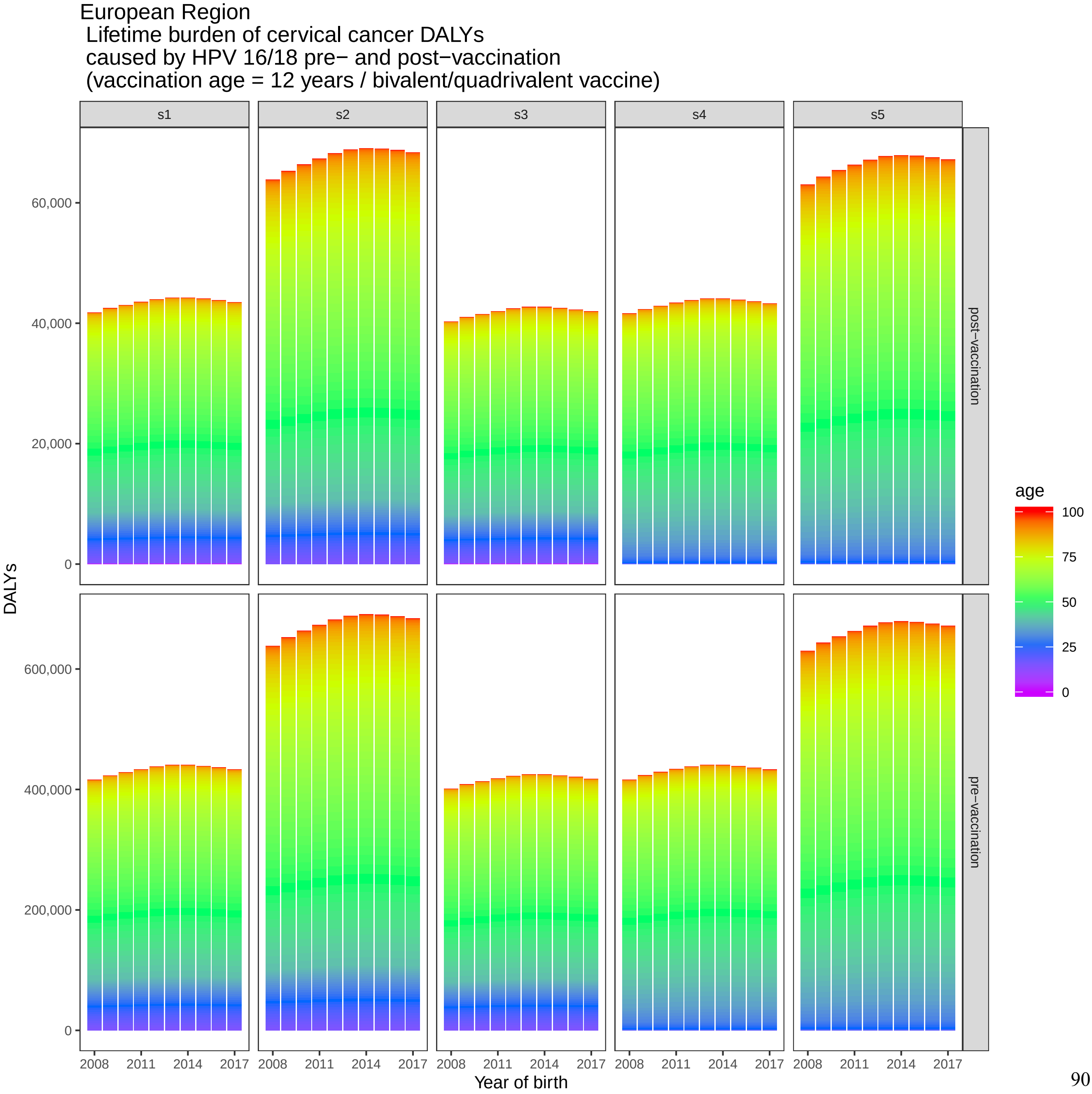
European Region
Lifetime burden of cervical cancer DALYs
caused by HPV 16/18 pre− and post−vaccination
(vaccination age = 12 years / bivalent/quadrivalent vaccine)
s1
s2
s3
s4
s5
post−vaccination
pre−vaccination
DALYs
0
20,000
40,000
60,000
200,000
400,000
600,000
2008
2011
2014
2017
Year of birth
age
0
25
50
75
100

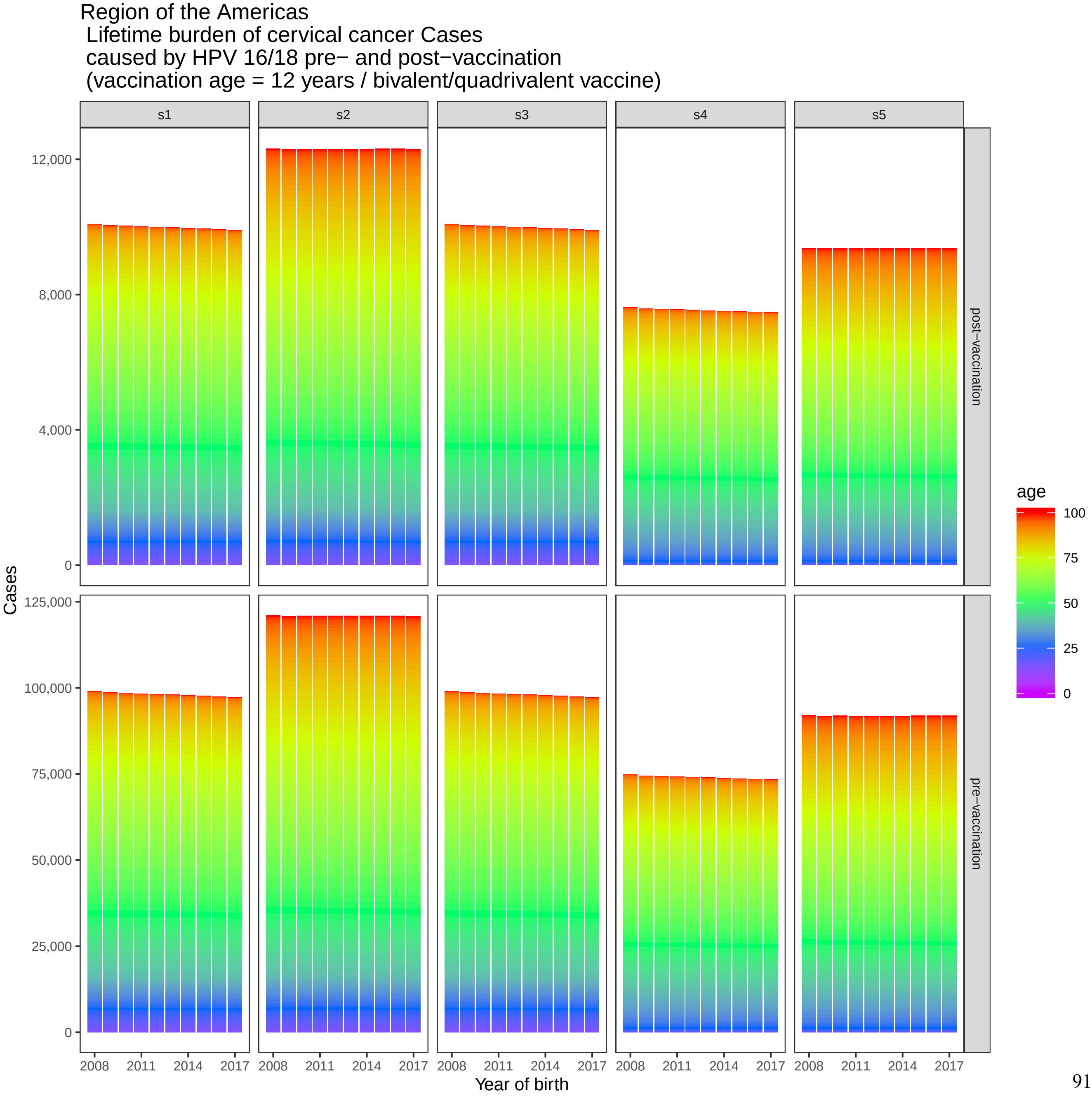

Region of the Americas
Lifetime burden of cervical cancer Cases
caused by HPV 16/18 pre− and post−vaccination
(vaccination age = 12 years / bivalent/quadrivalent vaccine)
s1
s2
s3
s4
s5
post−vaccination
pre−vaccination
Cases
12,000
8,000
4,000
0
125,000
100,000
75,000
50,000
25,000
0
2008
2011
2014
2017
Year of birth
age
100
75
50
25
0

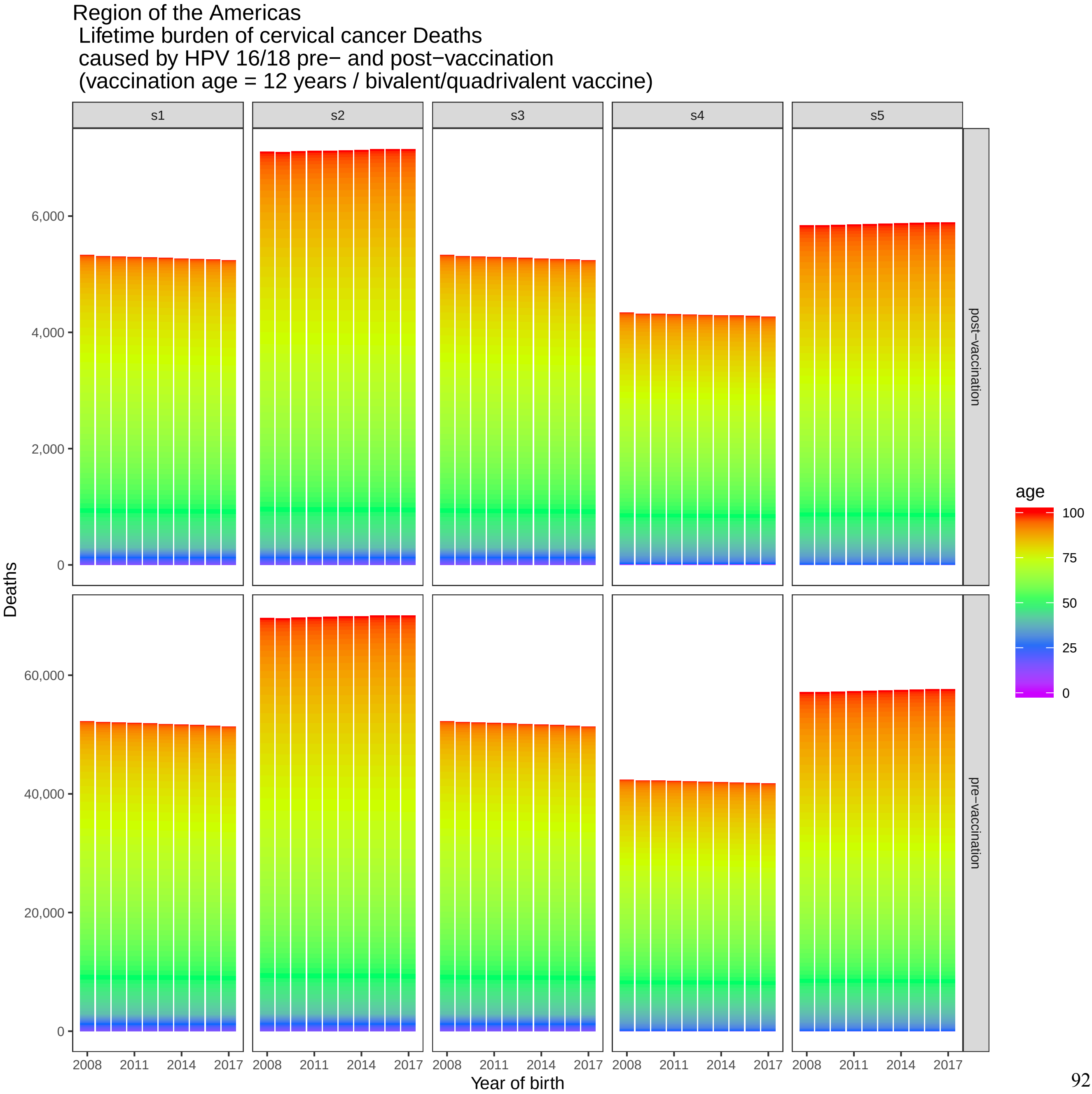

Region of the Americas
Lifetime burden of cervical cancer Deaths
caused by HPV 16/18 pre− and post−vaccination
(vaccination age = 12 years / bivalent/quadrivalent vaccine)
s1
s2
s3
s4
s5
post−vaccination
pre−vaccination
Deaths
6,000
4,000
2,000
0
60,000
40,000
20,000
0
2008
2011
2014
2017
Year of birth
age
100
75
50
25
0

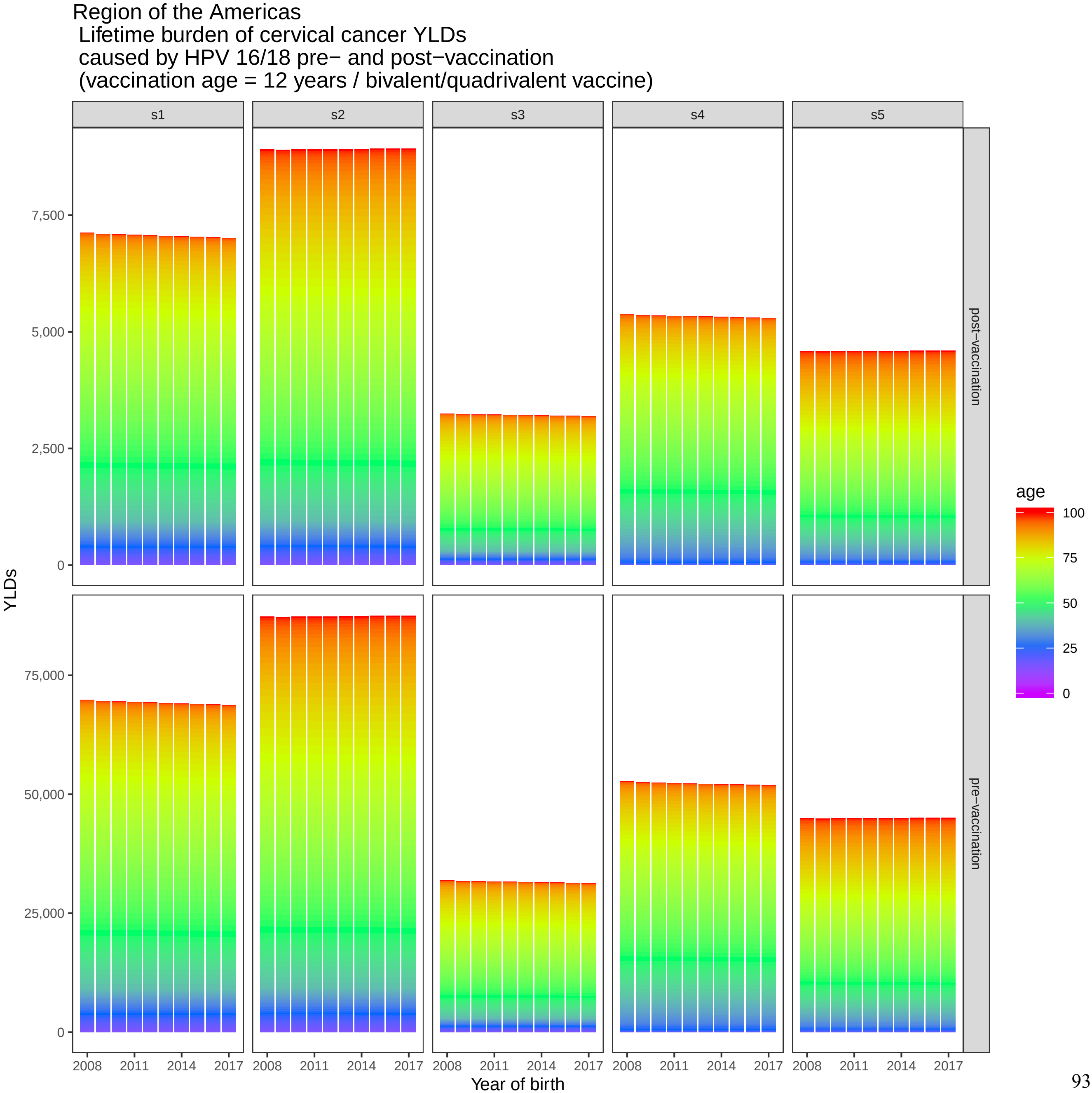
Region of the Americas
Lifetime burden of cervical cancer YLDs
caused by HPV 16/18 pre− and post−vaccination
(vaccination age = 12 years / bivalent/quadrivalent vaccine)
s1
s2
s3
s4
s5
post−vaccination
pre−vaccination
YLDs
0
2,500
5,000
7,500
0
25,000
50,000
75,000
2008
2011
2014
2017
Year of birth
age
0
25
50
75
100

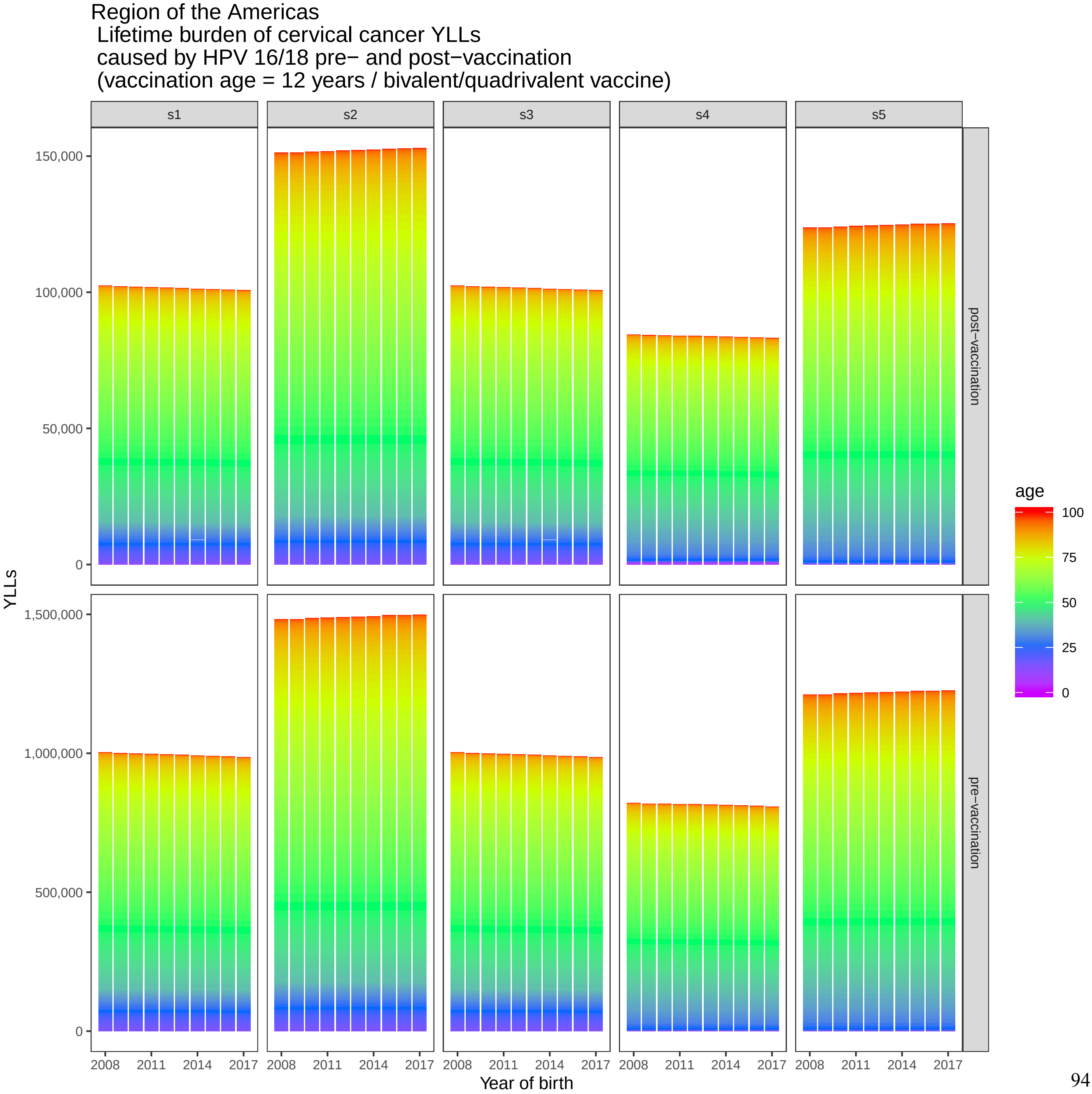
Region of the Americas
Lifetime burden of cervical cancer YLLs
caused by HPV 16/18 pre− and post−vaccination
(vaccination age = 12 years / bivalent/quadrivalent vaccine)
s1
s2
s3
s4
s5
post−vaccination
pre−vaccination
YLLs
150,000
100,000
50,000
0
1,500,000
1,000,000
500,000
0
2008
2011
2014
2017
Year of birth
age
100
75
50
25
0

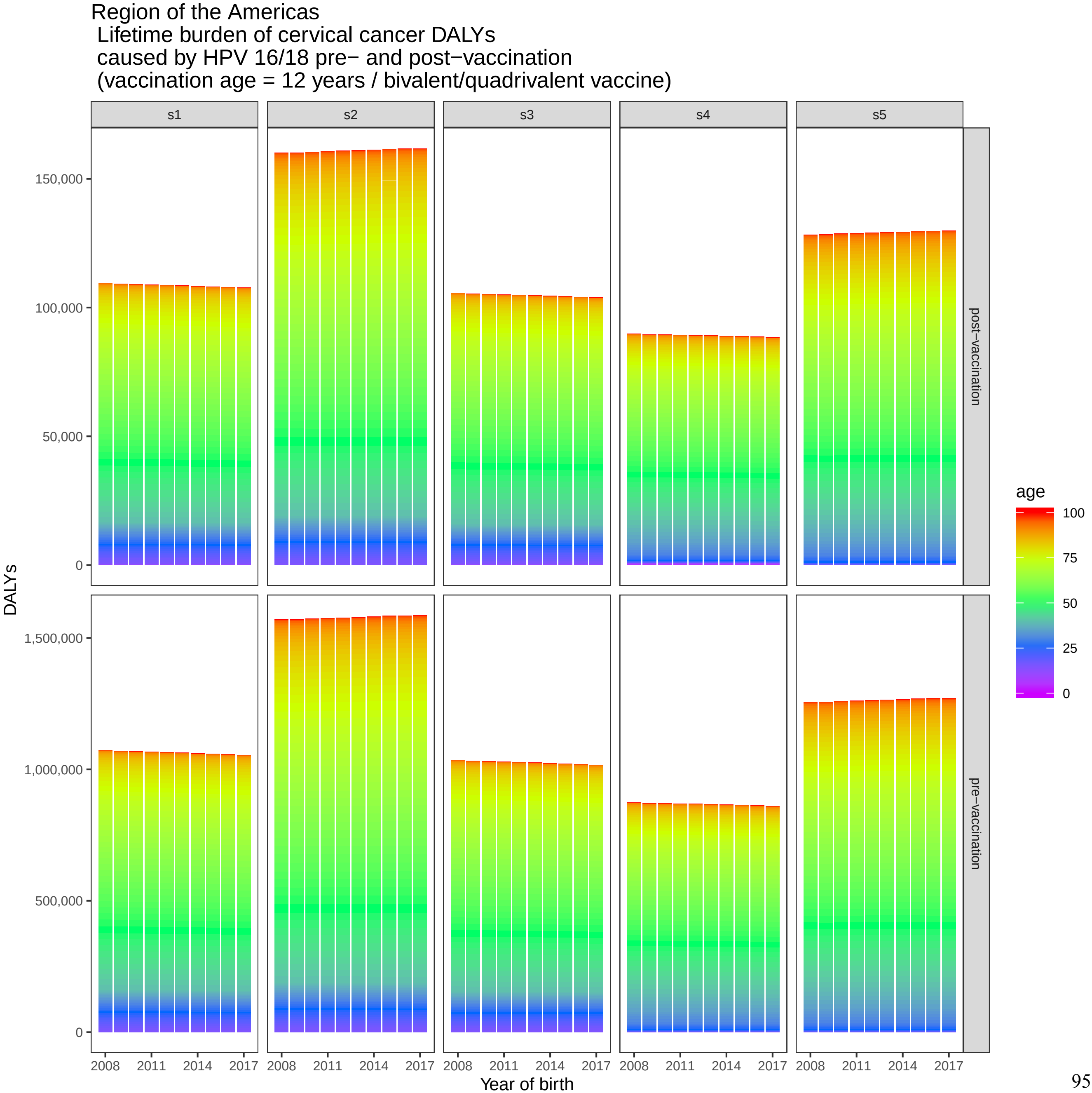
Region of the Americas
Lifetime burden of cervical cancer DALYs
caused by HPV 16/18 pre− and post−vaccination
(vaccination age = 12 years / bivalent/quadrivalent vaccine)
s1
s2
s3
s4
s5
post−vaccination
pre−vaccination
DALYs
150,000
100,000
50,000
0
1,500,000
1,000,000
500,000
0
2008
2011
2014
2017
Year of birth
age
100
75
50
25
0

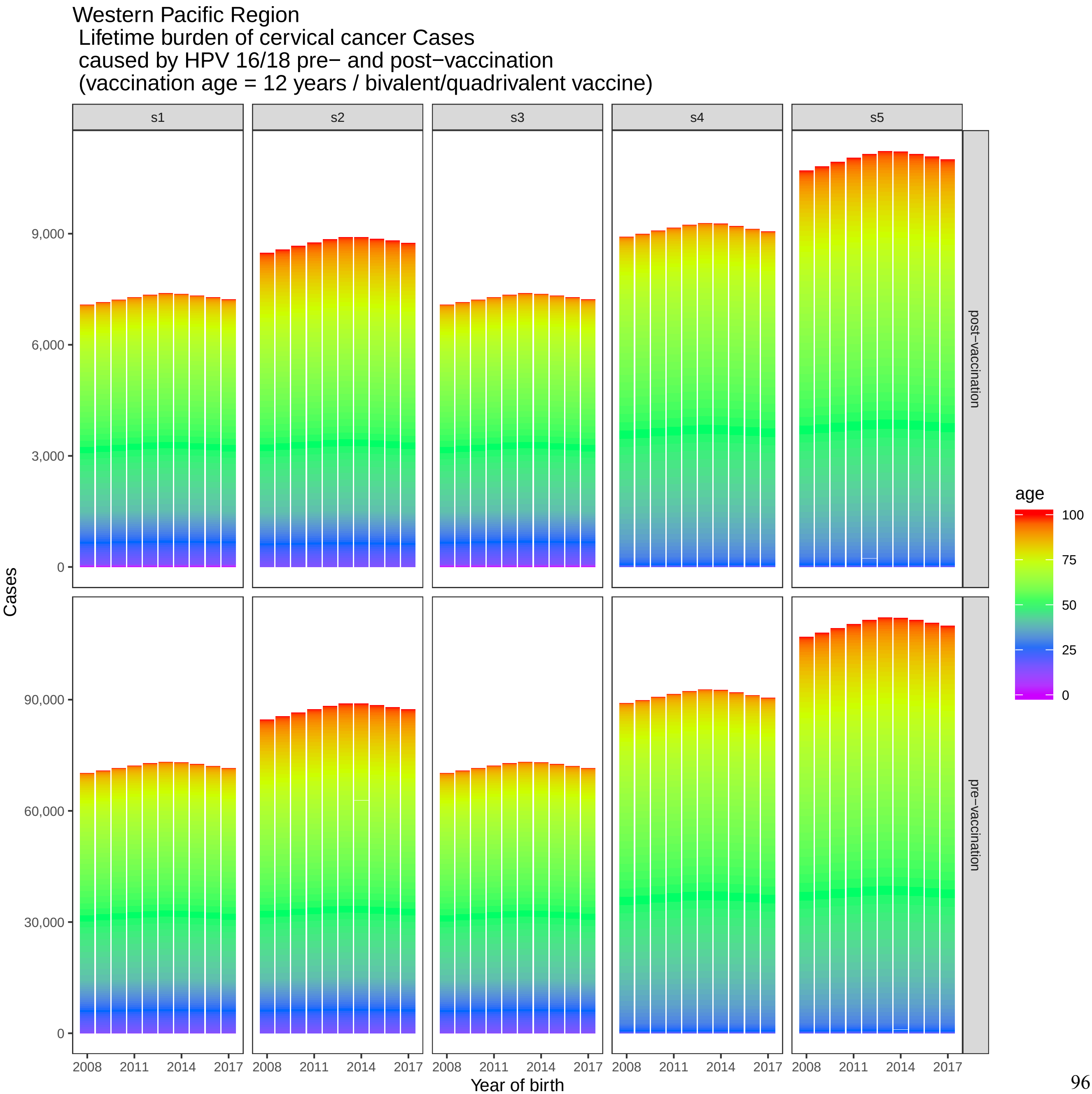
Western Pacific Region
Lifetime burden of cervical cancer Cases
caused by HPV 16/18 pre− and post−vaccination
(vaccination age = 12 years / bivalent/quadrivalent vaccine)
s1
s2
s3
s4
s5
post−vaccination
pre−vaccination
Cases
9,000
6,000
3,000
0
90,000
60,000
30,000
0
2008
2011
2014
2017
Year of birth
age
100
75
50
25
0

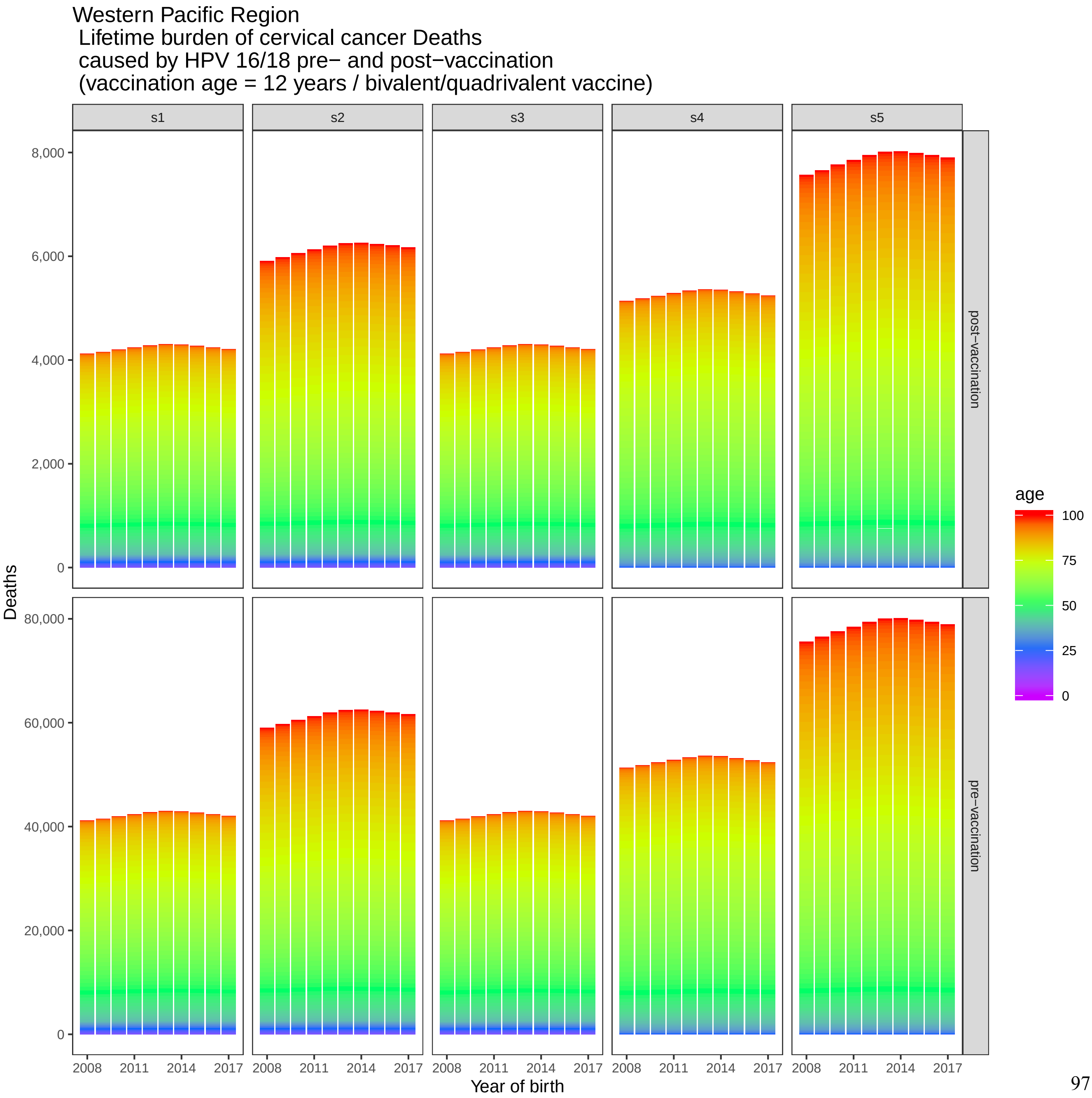
Western Pacific Region
Lifetime burden of cervical cancer Deaths
caused by HPV 16/18 pre− and post−vaccination
(vaccination age = 12 years / bivalent/quadrivalent vaccine)
s1
s2
s3
s4
s5
post−vaccination
pre−vaccination
Deaths
0
2,000
4,000
6,000
8,000
20,000
40,000
60,000
80,000
2008
2011
2014
2017
Year of birth
age
100
75
50
25
0

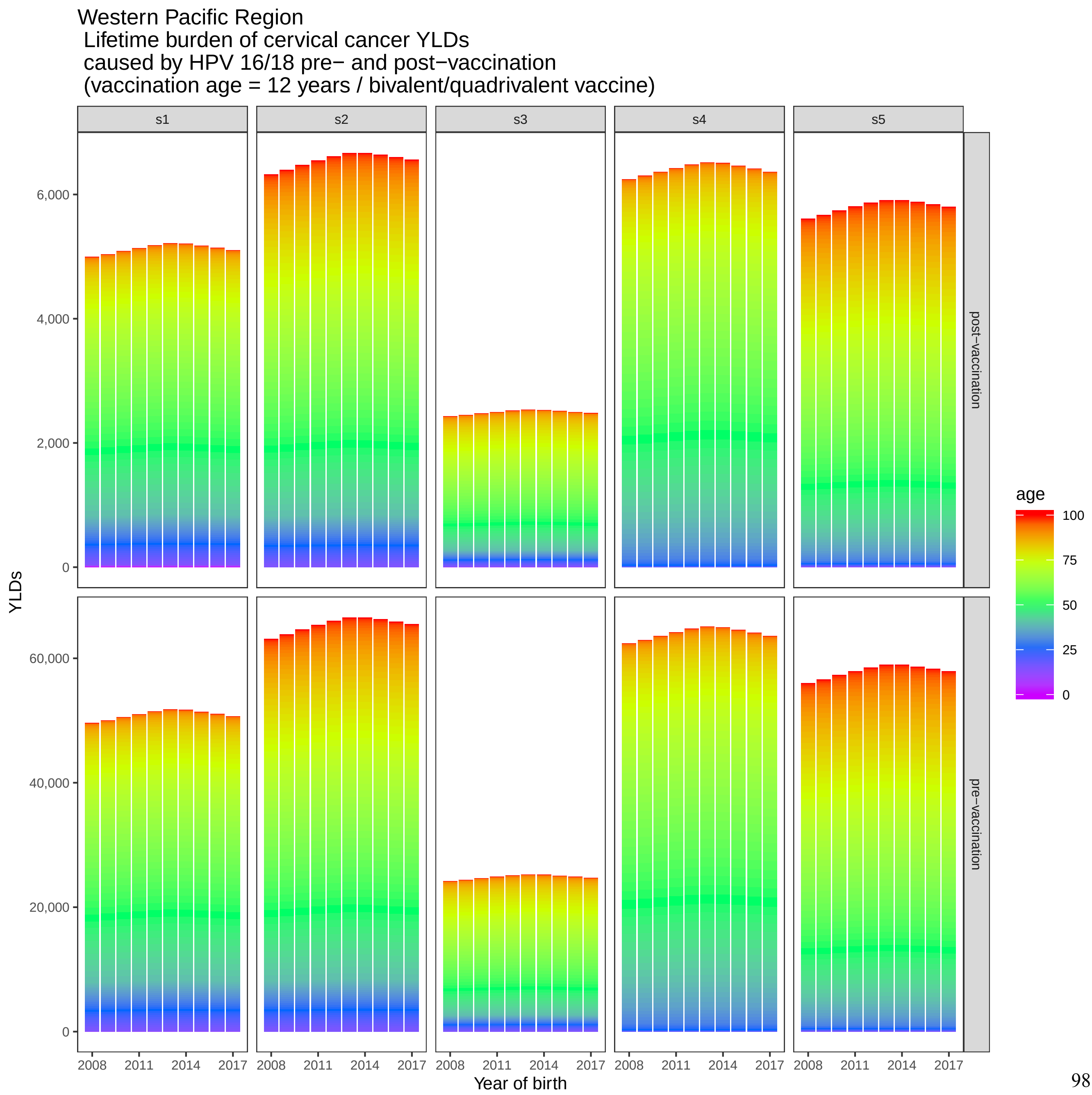
Western Pacific Region
Lifetime burden of cervical cancer YLDs
caused by HPV 16/18 pre− and post−vaccination
(vaccination age = 12 years / bivalent/quadrivalent vaccine)
s1
s2
s3
s4
s5
post−vaccination
pre−vaccination
YLDs
6,000
4,000
2,000
0
60,000
40,000
20,000
0
2008
2011
2014
2017
Year of birth
age
100
75
50
25
0

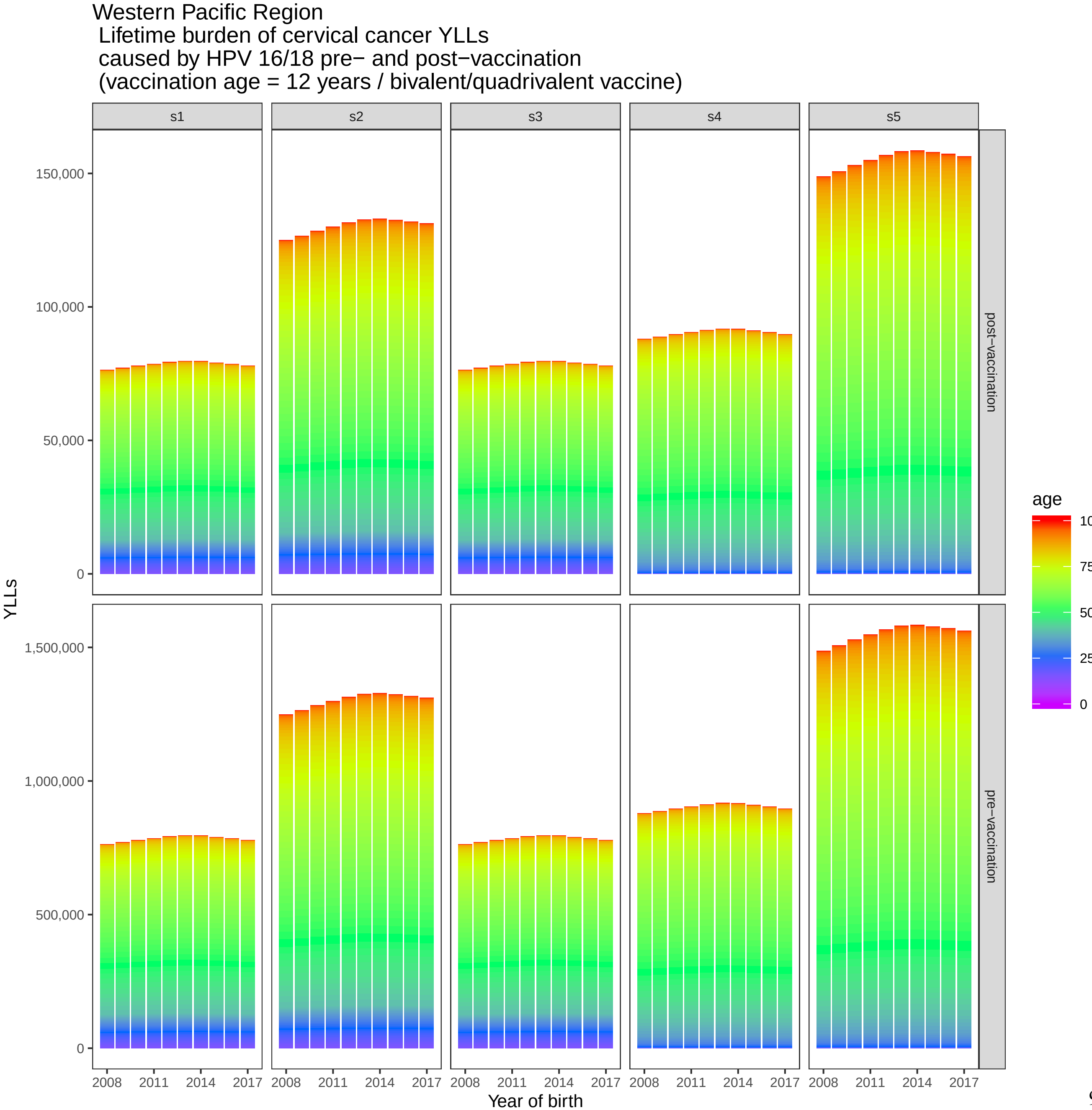

Western Pacific Region
Lifetime burden of cervical cancer YLLs
caused by HPV 16/18 pre− and post−vaccination
(vaccination age = 12 years / bivalent/quadrivalent vaccine)
s1
s2
s3
s4
s5
post−vaccination
pre−vaccination
YLLs
150,000
100,000
50,000
0
1,500,000
1,000,000
500,000
0
2008
2011
2014
2017
Year of birth
age
100
75
50
25
0

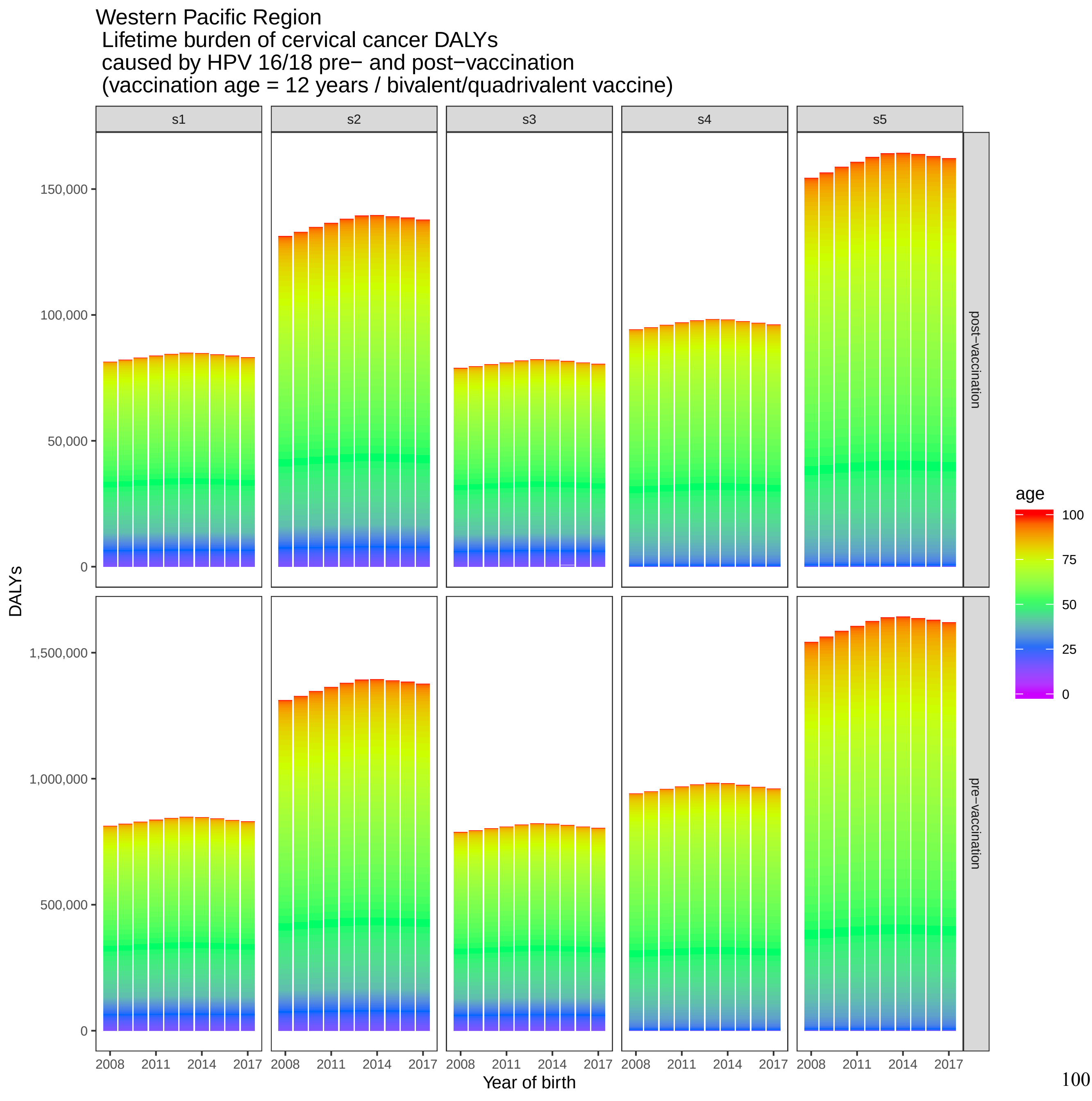

Western Pacific Region
Lifetime burden of cervical cancer DALYs
caused by HPV 16/18 pre− and post−vaccination
(vaccination age = 12 years / bivalent/quadrivalent vaccine)
s1
s2
s3
s4
s5
post−vaccination
pre−vaccination
150,000
100,000
50,000
0
1,500,000
1,000,000
500,000
0
DALYs
2008
2011
2014
2017
Year of birth
age
100
75
50
25
0

South−East Asia Region
Lifetime burden of cervical cancer Cases
caused by HPV 16/18 pre− and post−vaccination
(vaccination age = 12 years / bivalent/quadrivalent vaccine)

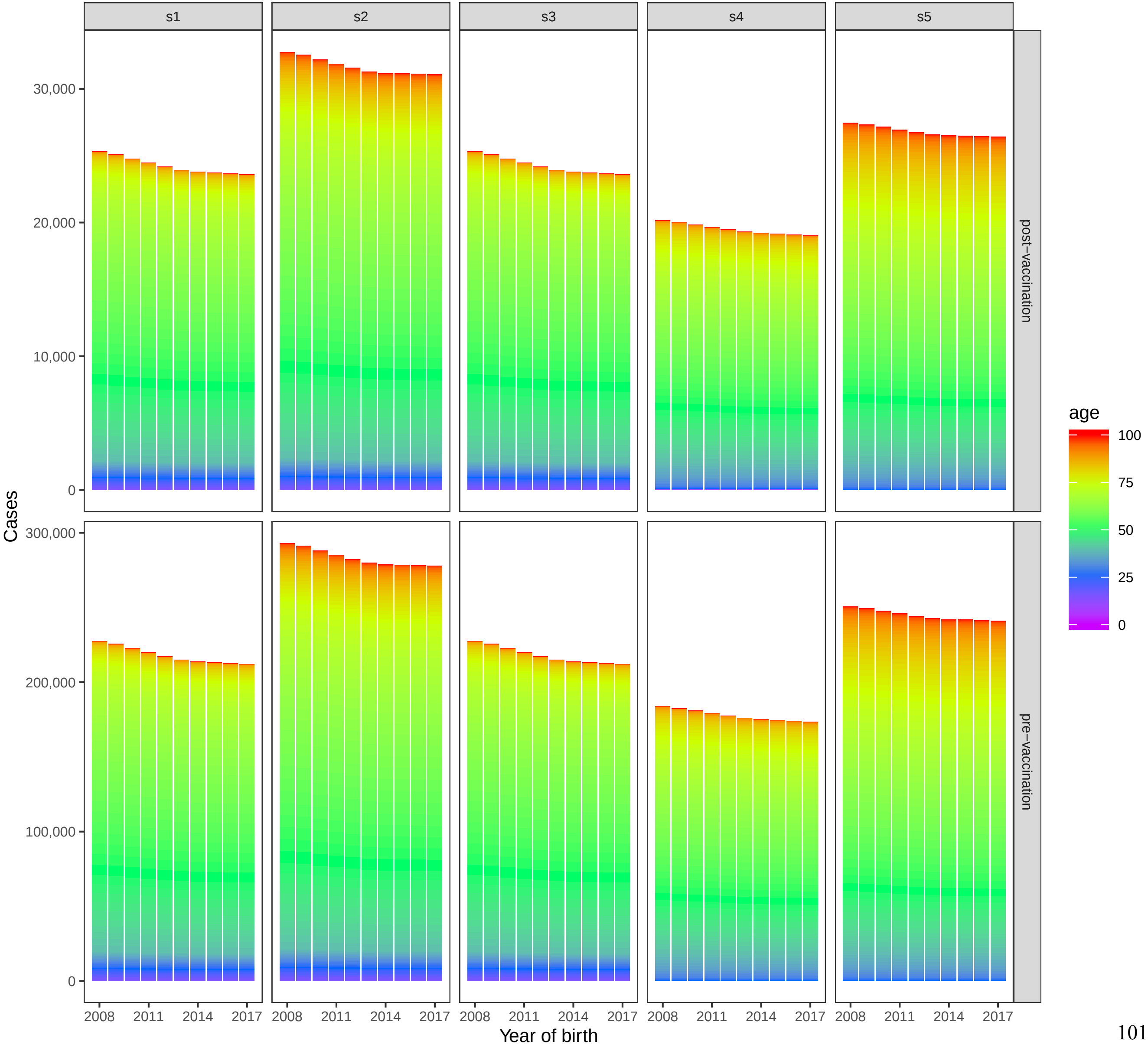

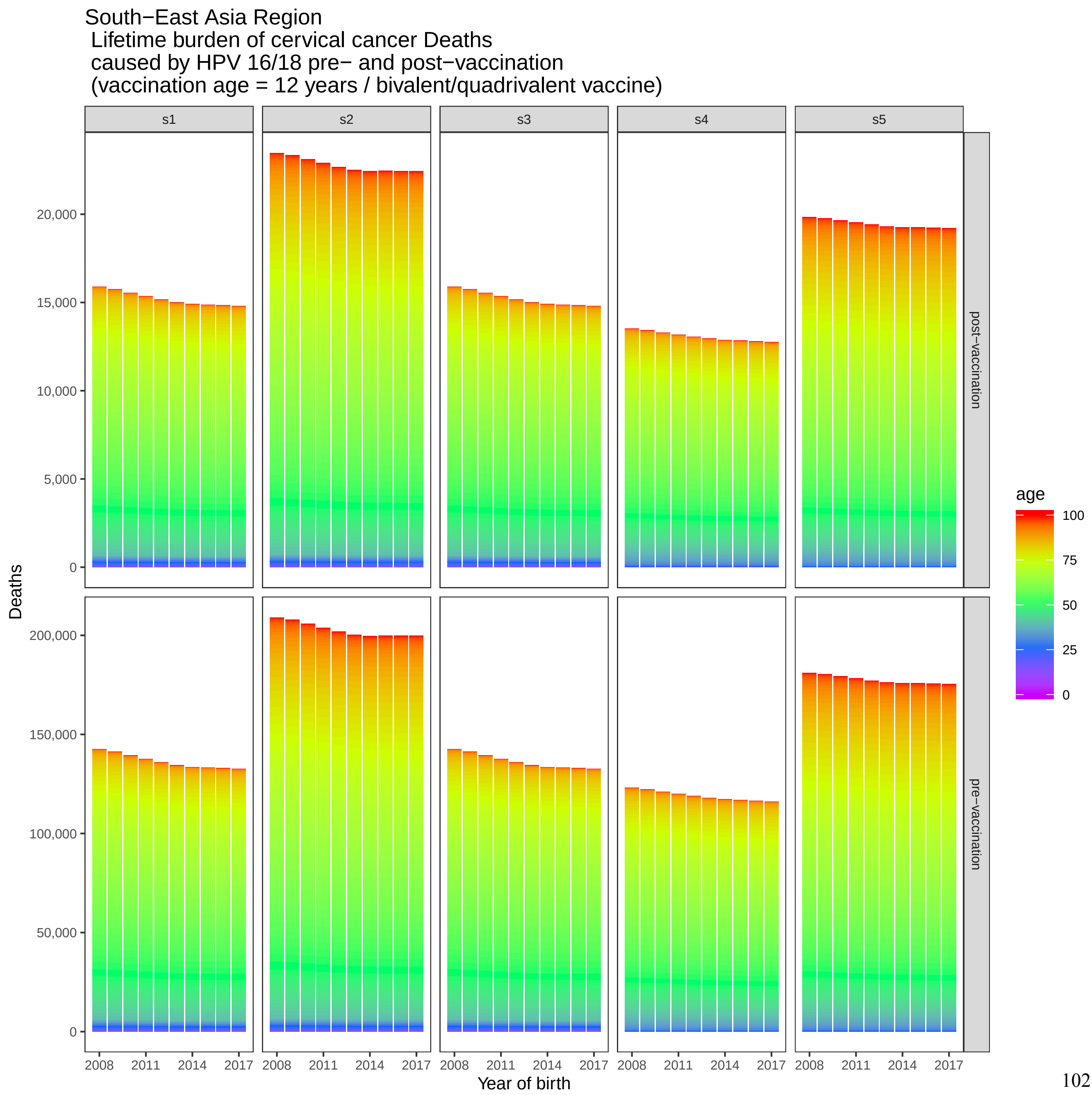
South−East Asia Region
Lifetime burden of cervical cancer Deaths
caused by HPV 16/18 pre− and post−vaccination
(vaccination age = 12 years / bivalent/quadrivalent vaccine)
s1
s2
s3
s4
s5
post−vaccination
pre−vaccination
Deaths
Year of birth
age
0
25
50
75
100
20,000
15,000
10,000
5,000
0
200,000
150,000
100,000
50,000
2008
2011
2014
2017

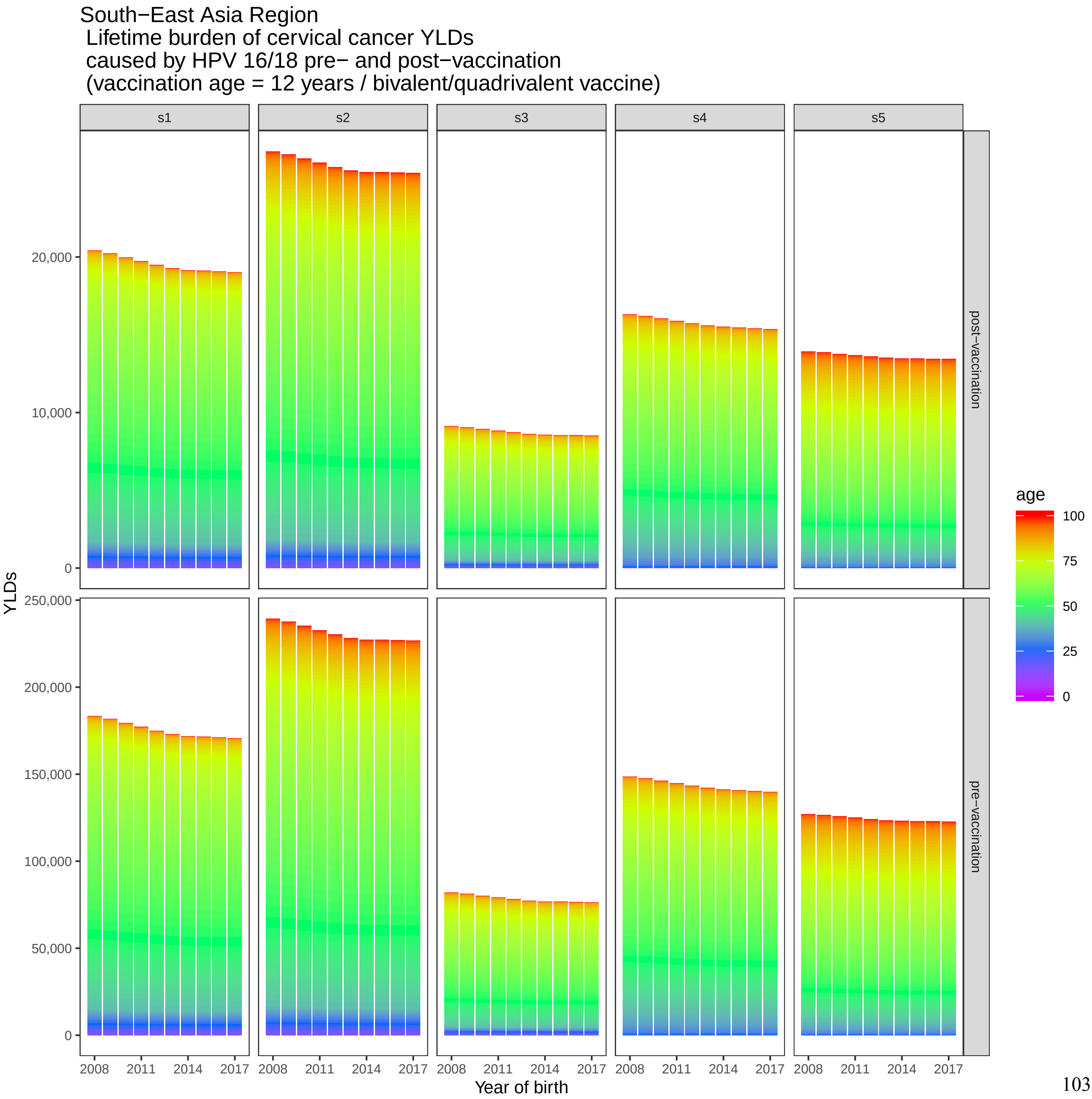
South−East Asia Region
Lifetime burden of cervical cancer YLDs
caused by HPV 16/18 pre− and post−vaccination
(vaccination age = 12 years / bivalent/quadrivalent vaccine)
s1
s2
s3
s4
s5
post−vaccination
pre−vaccination
YLDs
0
10,000
20,000
0
50,000
100,000
150,000
200,000
250,000
2008
2011
2014
2017
Year of birth
age
100
75
50
25
0

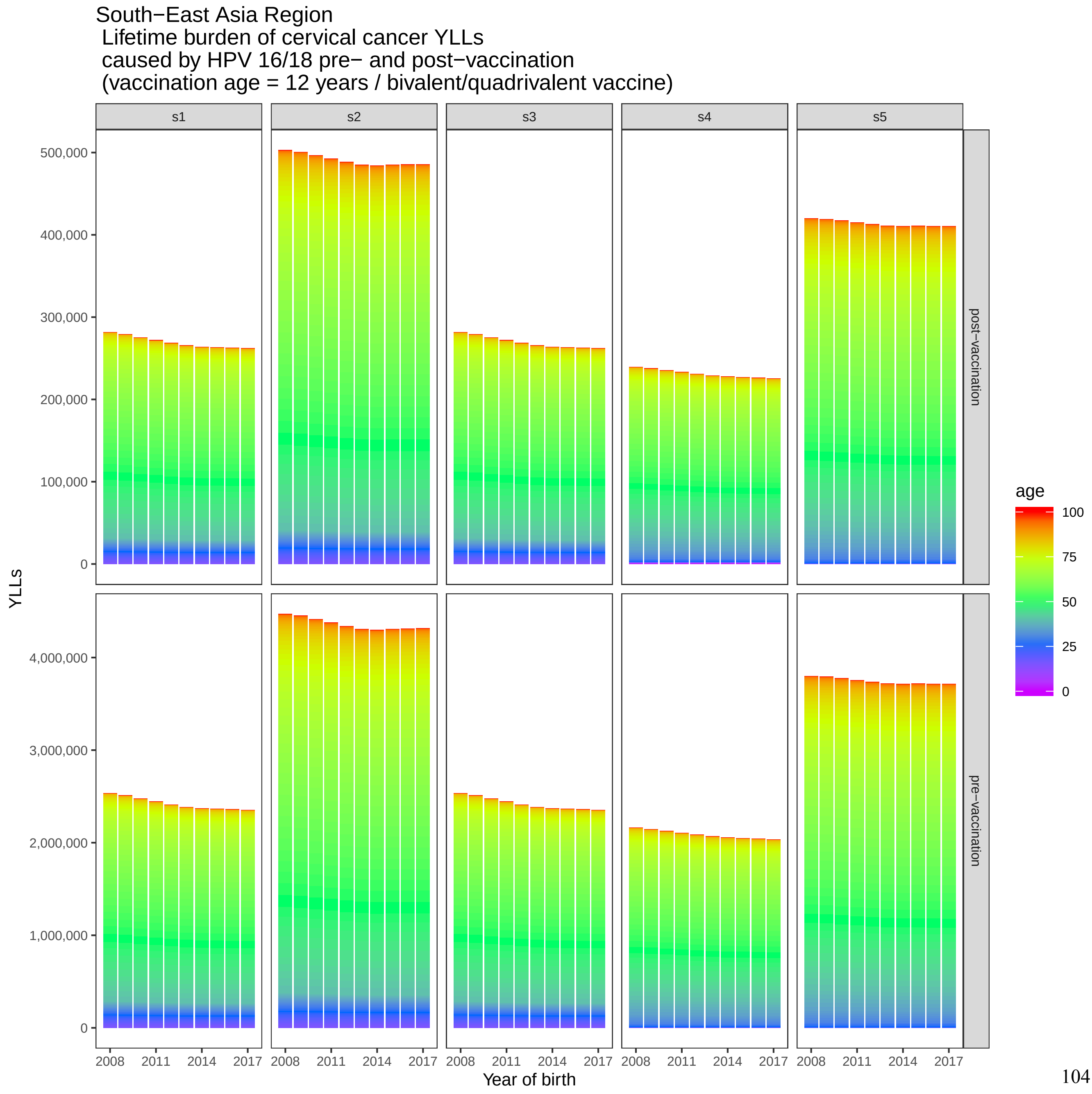
South−East Asia Region
Lifetime burden of cervical cancer YLLs
caused by HPV 16/18 pre− and post−vaccination
(vaccination age = 12 years / bivalent/quadrivalent vaccine)
s1
s2
s3
s4
s5
post−vaccination
pre−vaccination
YLLs
500,000
400,000
300,000
200,000
100,000
0
4,000,000
3,000,000
2,000,000
1,000,000
0
2008
2011
2014
2017
Year of birth
age
100
75
50
25
0

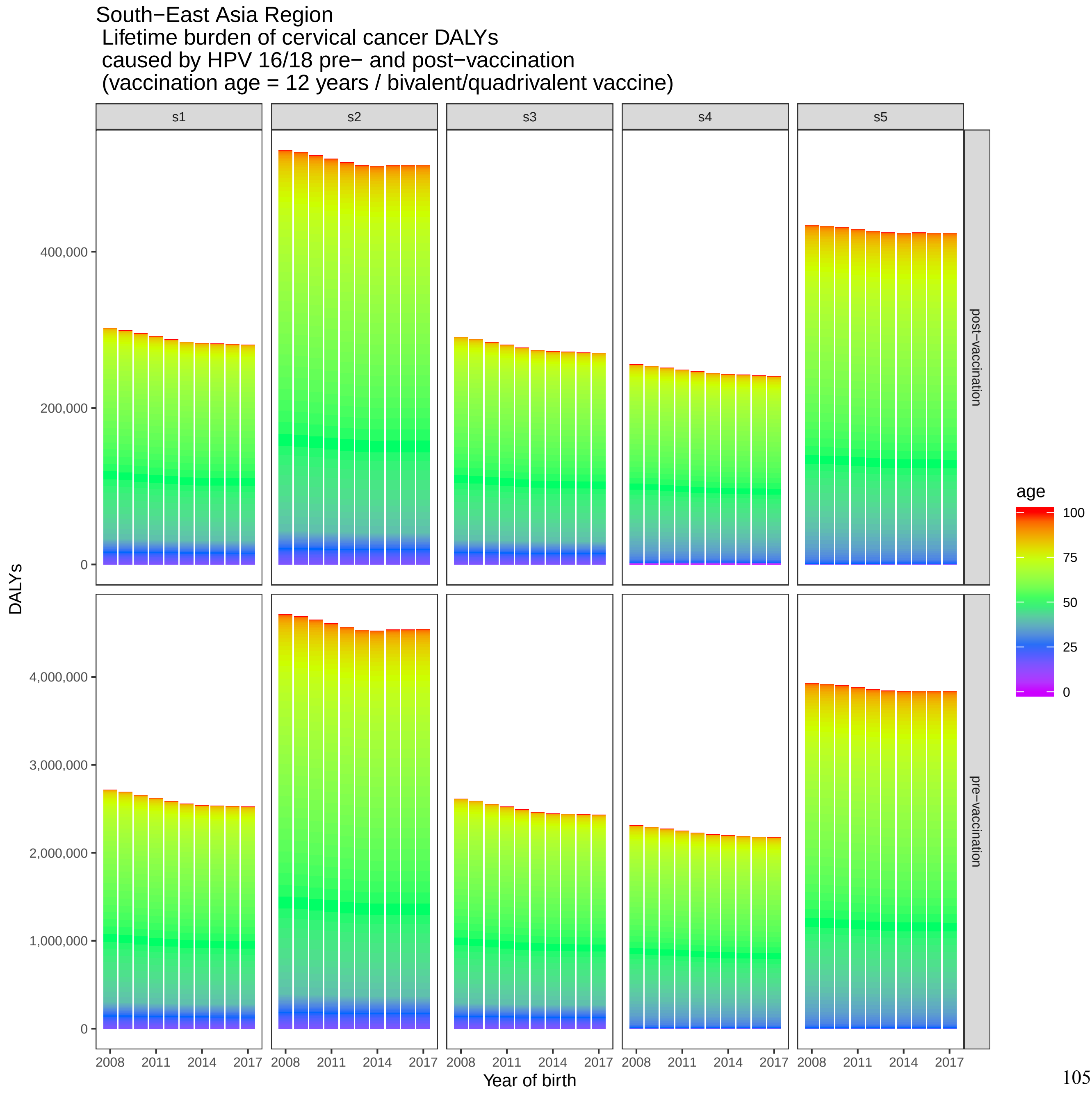

South−East Asia Region
Lifetime burden of cervical cancer DALYs
caused by HPV 16/18 pre− and post−vaccination
(vaccination age = 12 years / bivalent/quadrivalent vaccine)
s1
s2
s3
s4
s5
post−vaccination
pre−vaccination
DALYs
400,000
200,000
0
4,000,000
3,000,000
2,000,000
1,000,000
0
2008
2011
2014
2017
Year of birth
age
100
75
50
25
0

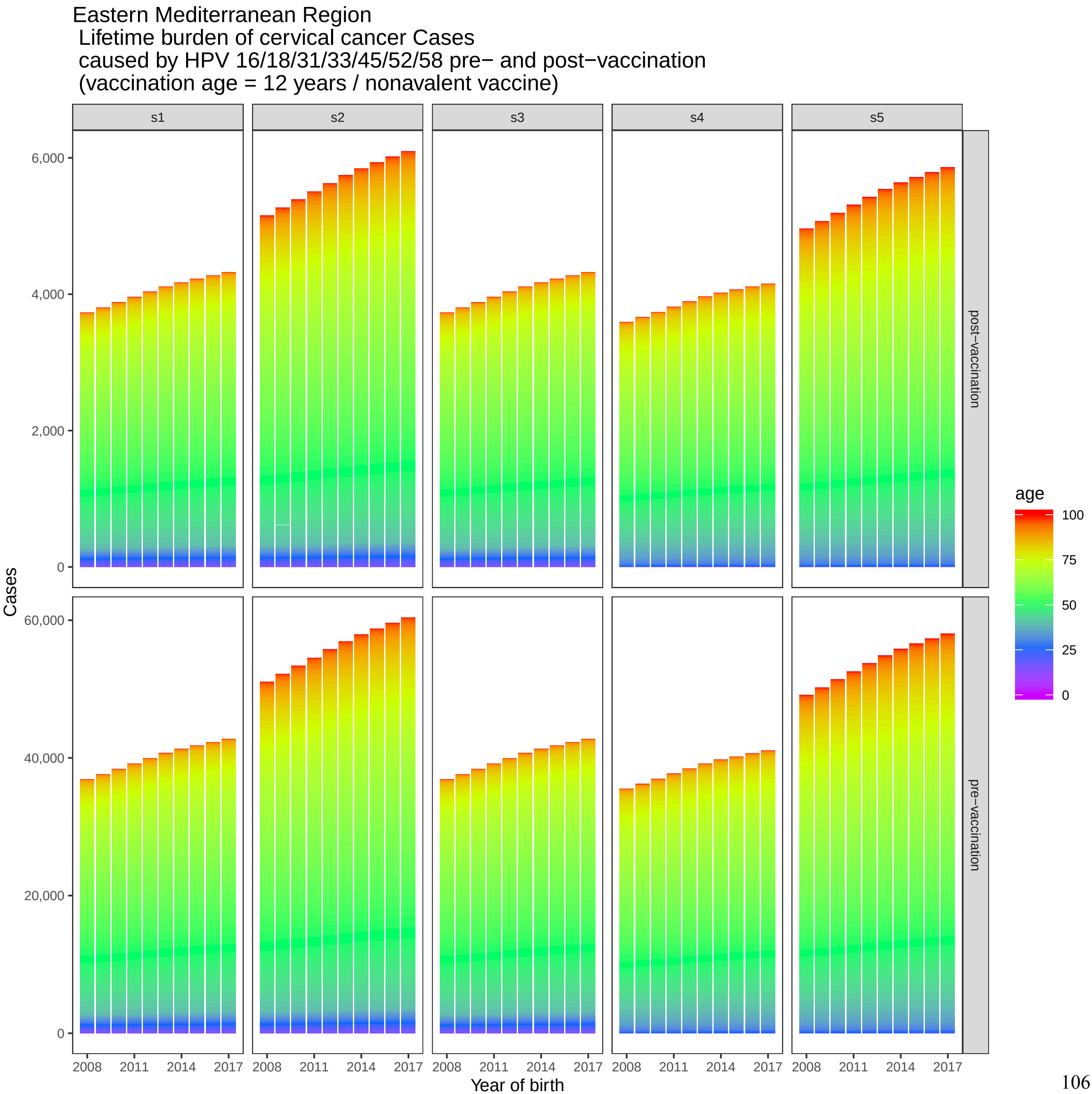

Eastern Mediterranean Region
Lifetime burden of cervical cancer Cases
caused by HPV 16/18/31/33/45/52/58 pre− and post−vaccination
(vaccination age = 12 years / nonavalent vaccine)
s1
s2
s3
s4
s5
post−vaccination
pre−vaccination
Cases
6,000
4,000
2,000
0
60,000
40,000
20,000
0
2008
2011
2014
2017
Year of birth
age
100
75
50
25
0

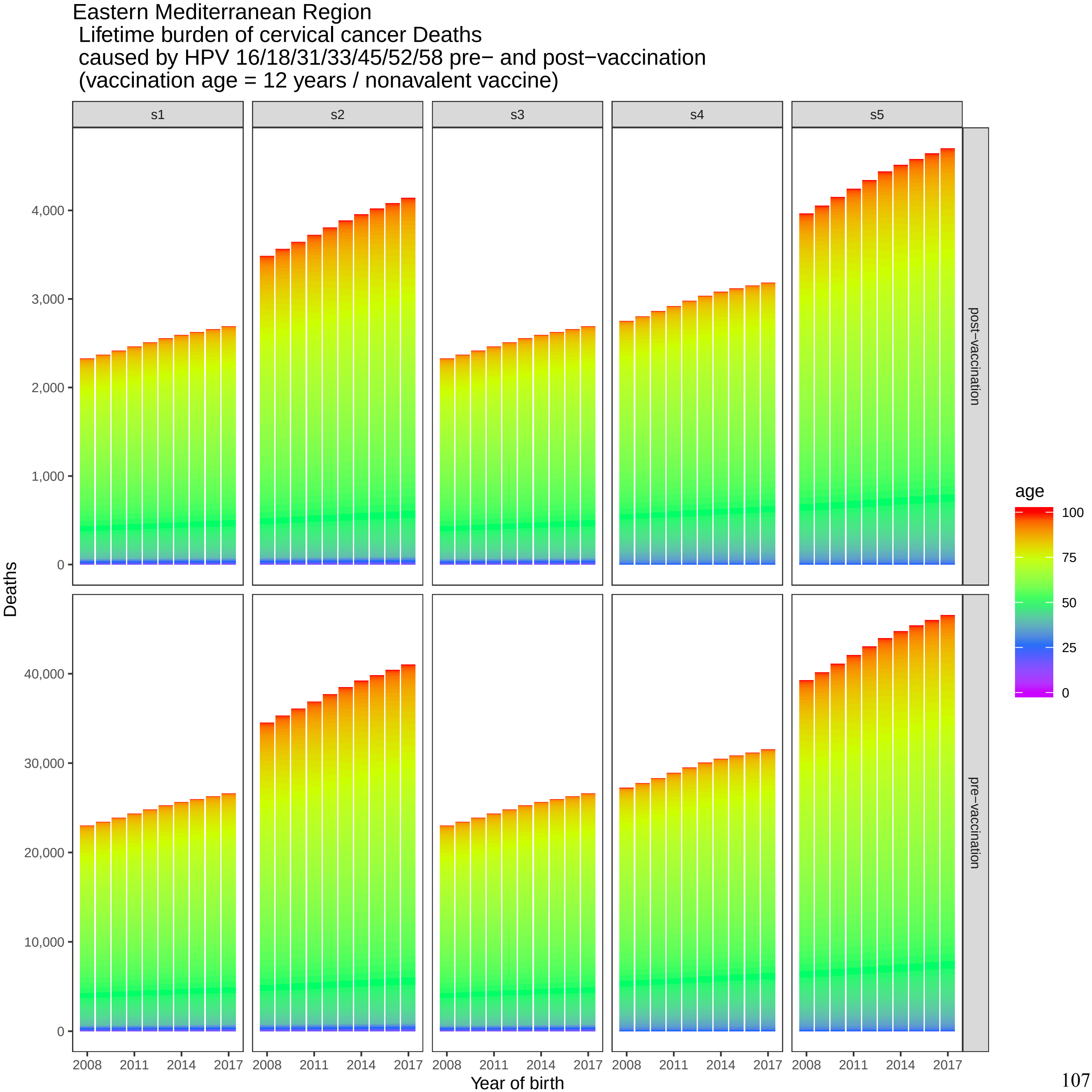
Eastern Mediterranean Region
Lifetime burden of cervical cancer Deaths
caused by HPV 16/18/31/33/45/52/58 pre− and post−vaccination
(vaccination age = 12 years / nonavalent vaccine)
s1
s2
s3
s4
s5
post−vaccination
pre−vaccination
Deaths
0
1,000
2,000
3,000
4,000
10,000
20,000
30,000
40,000
Year of birth
2008
2011
2014
2017
age
0
25
50
75
100

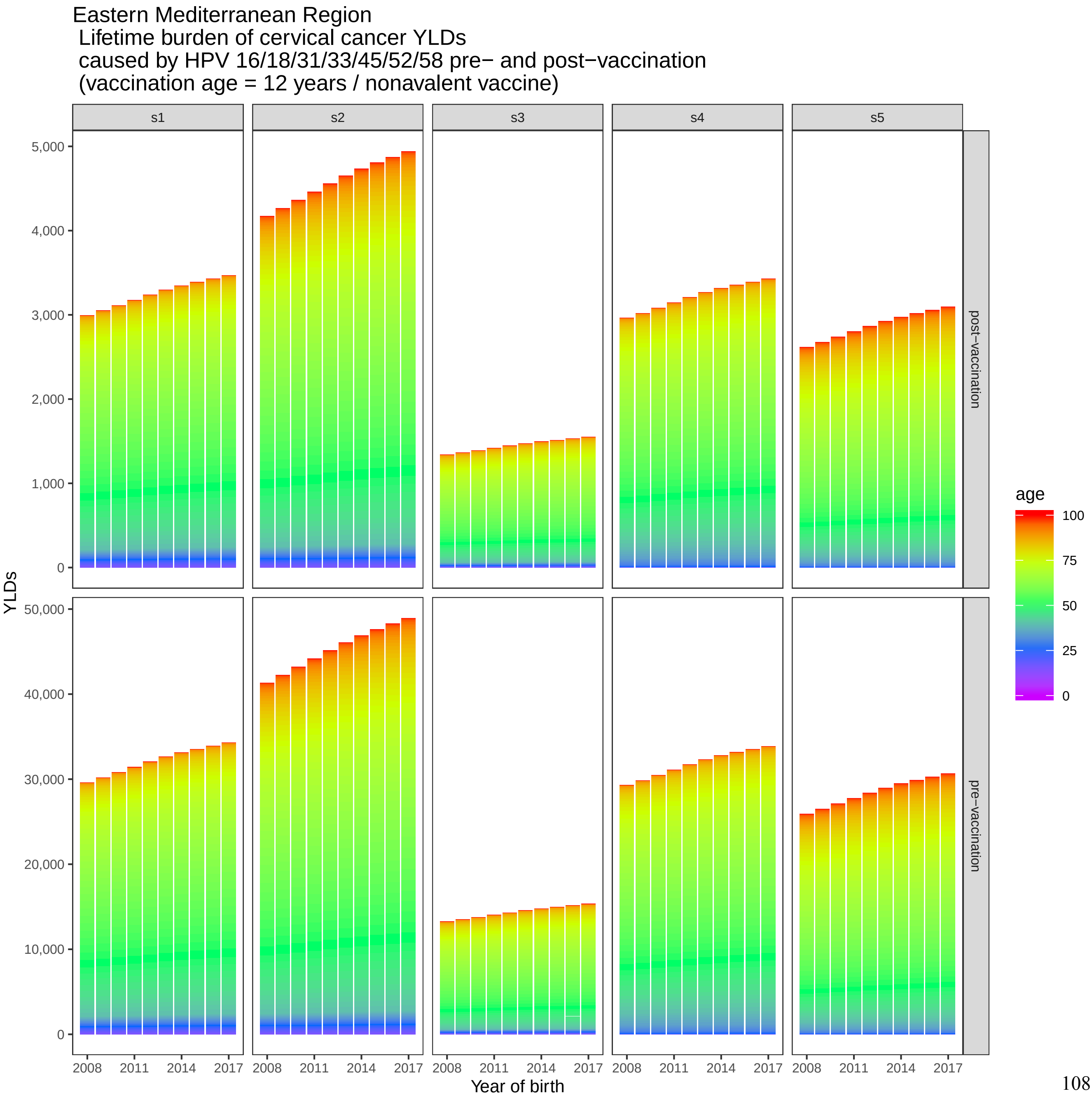

Eastern Mediterranean Region
Lifetime burden of cervical cancer YLDs
caused by HPV 16/18/31/33/45/52/58 pre− and post−vaccination
(vaccination age = 12 years / nonavalent vaccine)
s1
s2
s3
s4
s5
post−vaccination
pre−vaccination
YLDs
Year of birth
age

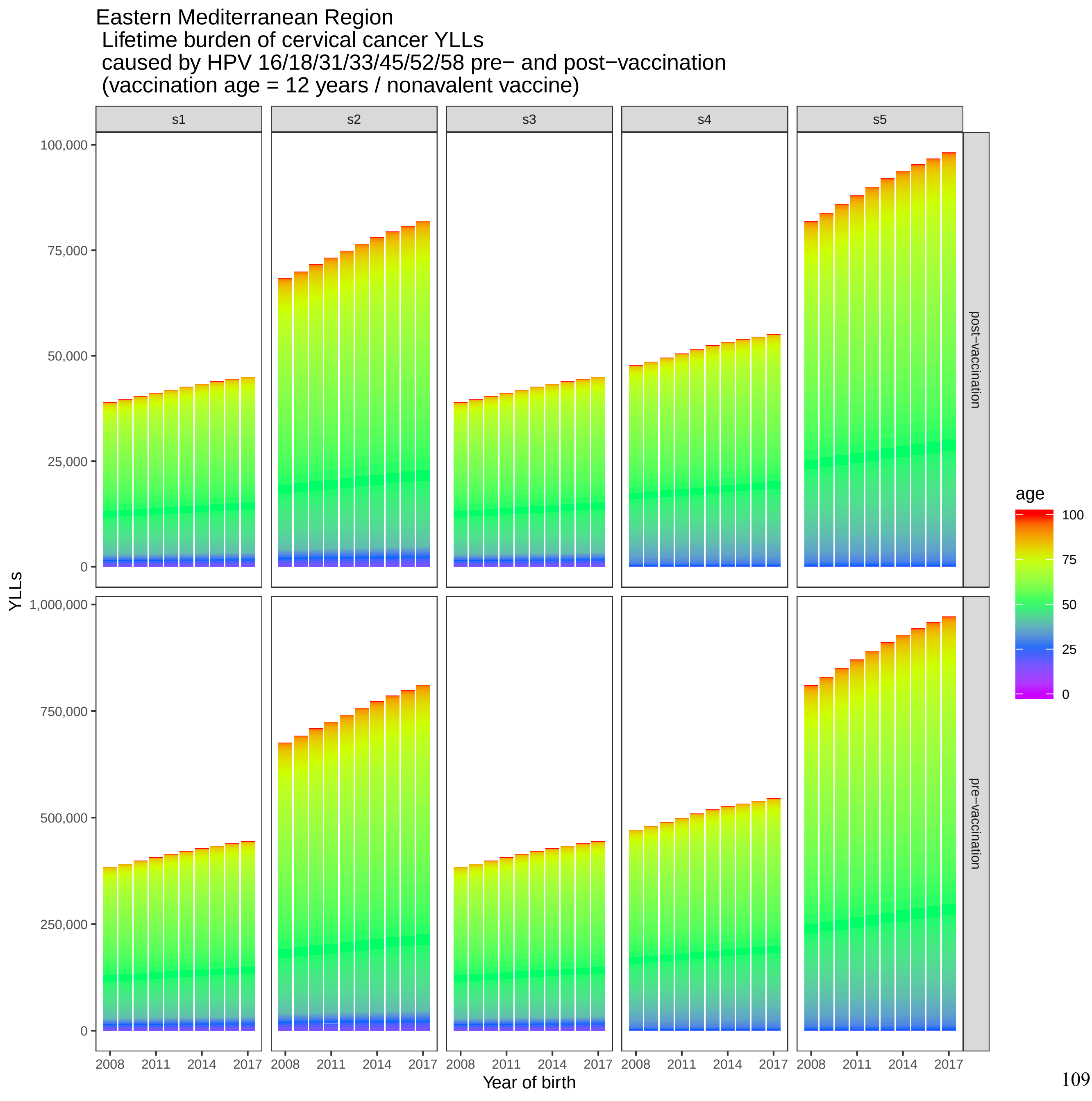
Eastern Mediterranean Region
Lifetime burden of cervical cancer YLLs
caused by HPV 16/18/31/33/45/52/58 pre− and post−vaccination
(vaccination age = 12 years / nonavalent vaccine)
s1
s2
s3
s4
s5
post−vaccination
pre−vaccination
YLLs
Year of birth
age
0
25
50
75
100
2008
2011
2014
2017
0
25,000
50,000
75,000
100,000
250,000
500,000
750,000
1,000,000

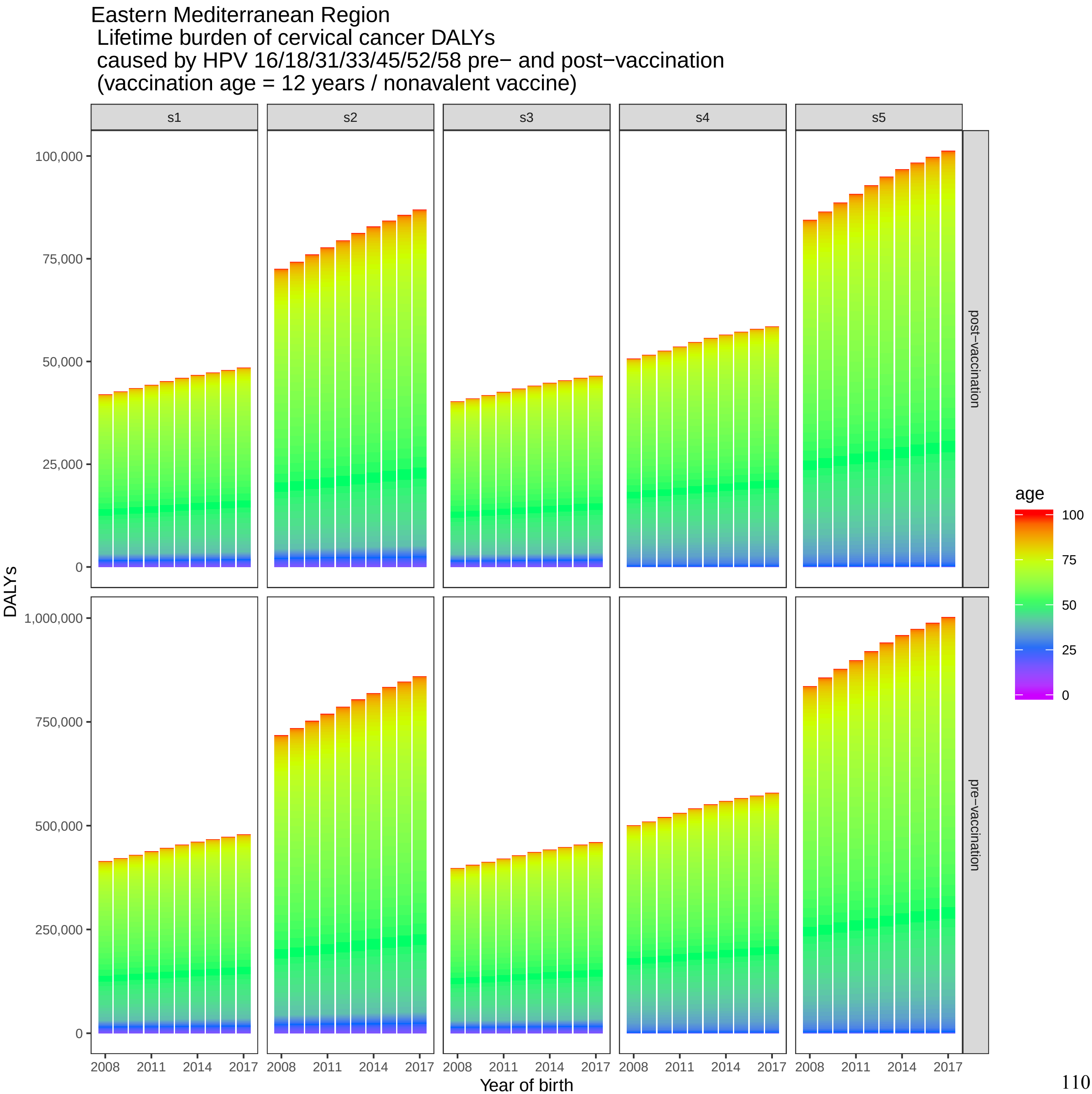
Eastern Mediterranean Region
Lifetime burden of cervical cancer DALYs
caused by HPV 16/18/31/33/45/52/58 pre− and post−vaccination
(vaccination age = 12 years / nonavalent vaccine)
s1
s2
s3
s4
s5
post−vaccination
pre−vaccination
DALYs
100,000
75,000
50,000
25,000
0
1,000,000
750,000
500,000
250,000
0
Year of birth
2008
2011
2014
2017
age
100
75
50
25
0

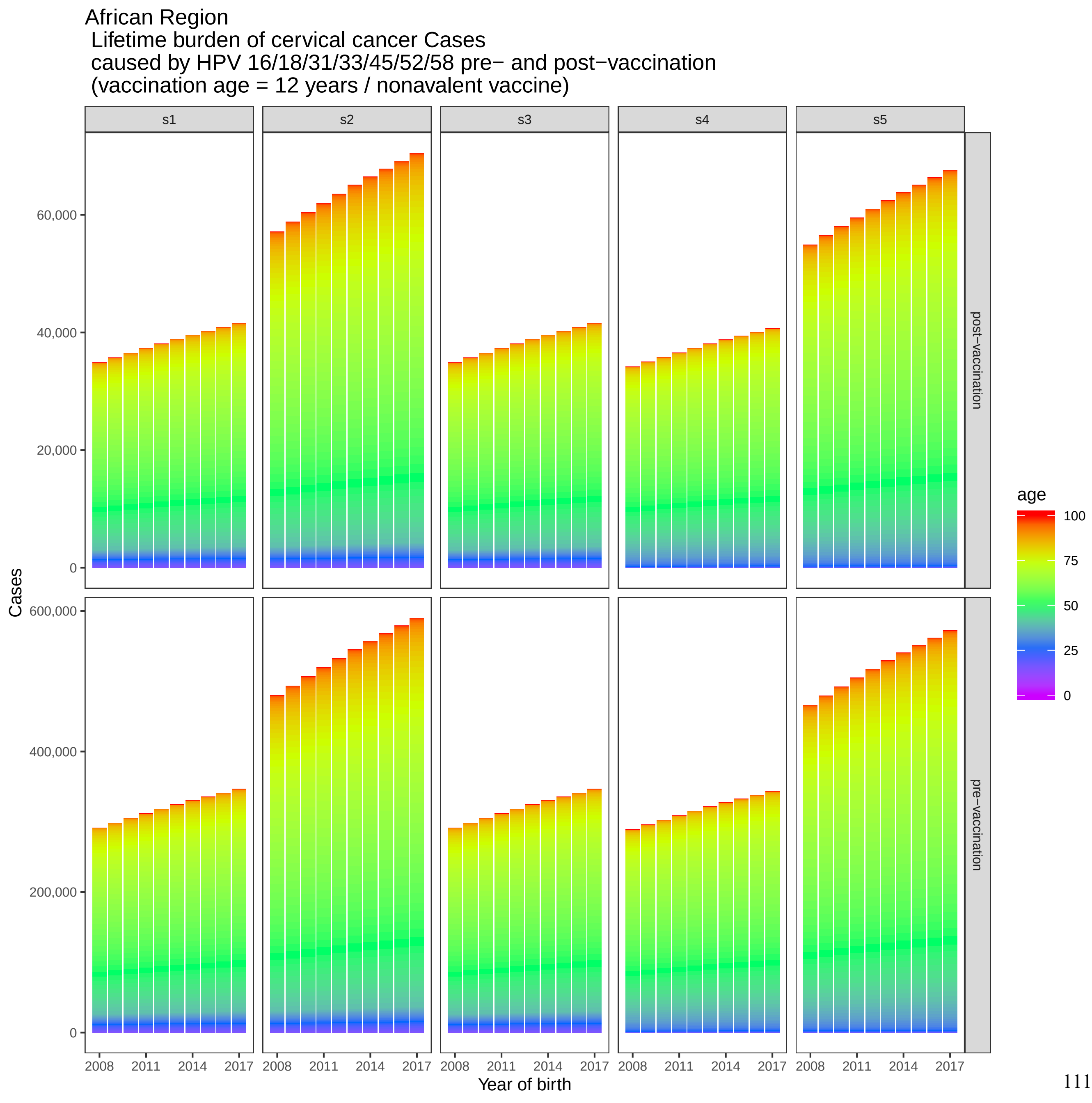
African Region
Lifetime burden of cervical cancer Cases
caused by HPV 16/18/31/33/45/52/58 pre− and post−vaccination
(vaccination age = 12 years / nonavalent vaccine)
s1
s2
s3
s4
s5
post−vaccination
pre−vaccination
Cases
Year of birth
age
2008
2011
2014
2017
0
20,000
40,000
60,000
200,000
400,000
600,000
100
75
50
25

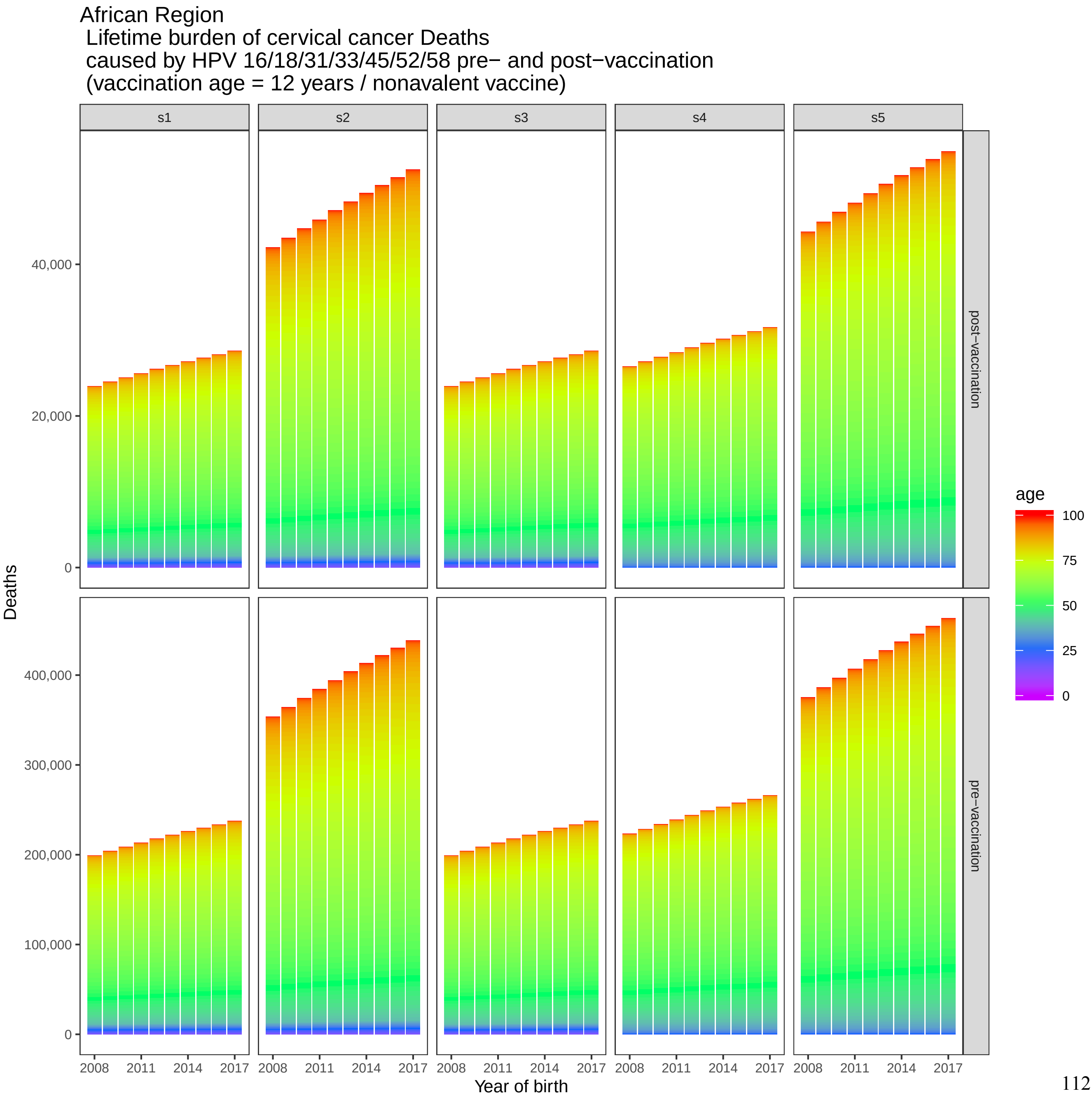

African Region
Lifetime burden of cervical cancer Deaths
caused by HPV 16/18/31/33/45/52/58 pre− and post−vaccination
(vaccination age = 12 years / nonavalent vaccine)
s1
s2
s3
s4
s5
post−vaccination
pre−vaccination
Deaths
0
20,000
40,000
100,000
200,000
300,000
400,000
Year of birth
2008
2011
2014
2017
age
0
25
50
75
100

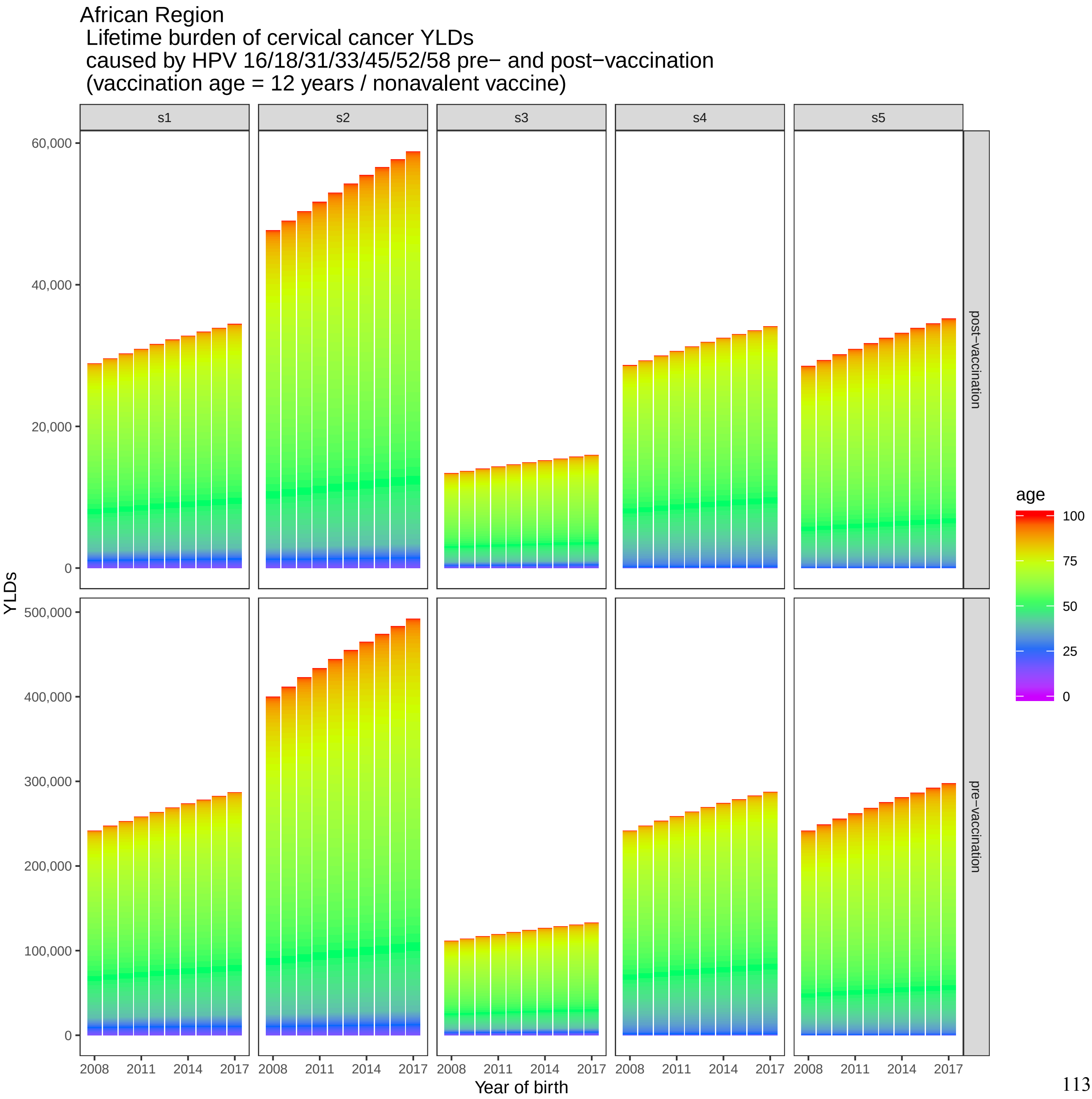
African Region
Lifetime burden of cervical cancer YLDs
caused by HPV 16/18/31/33/45/52/58 pre− and post−vaccination
(vaccination age = 12 years / nonavalent vaccine)
s1
s2
s3
s4
s5
post−vaccination
pre−vaccination
YLDs
0
20,000
40,000
60,000
0
100,000
200,000
300,000
400,000
500,000
2008
2011
2014
2017
Year of birth
age
0
25
50
75
100

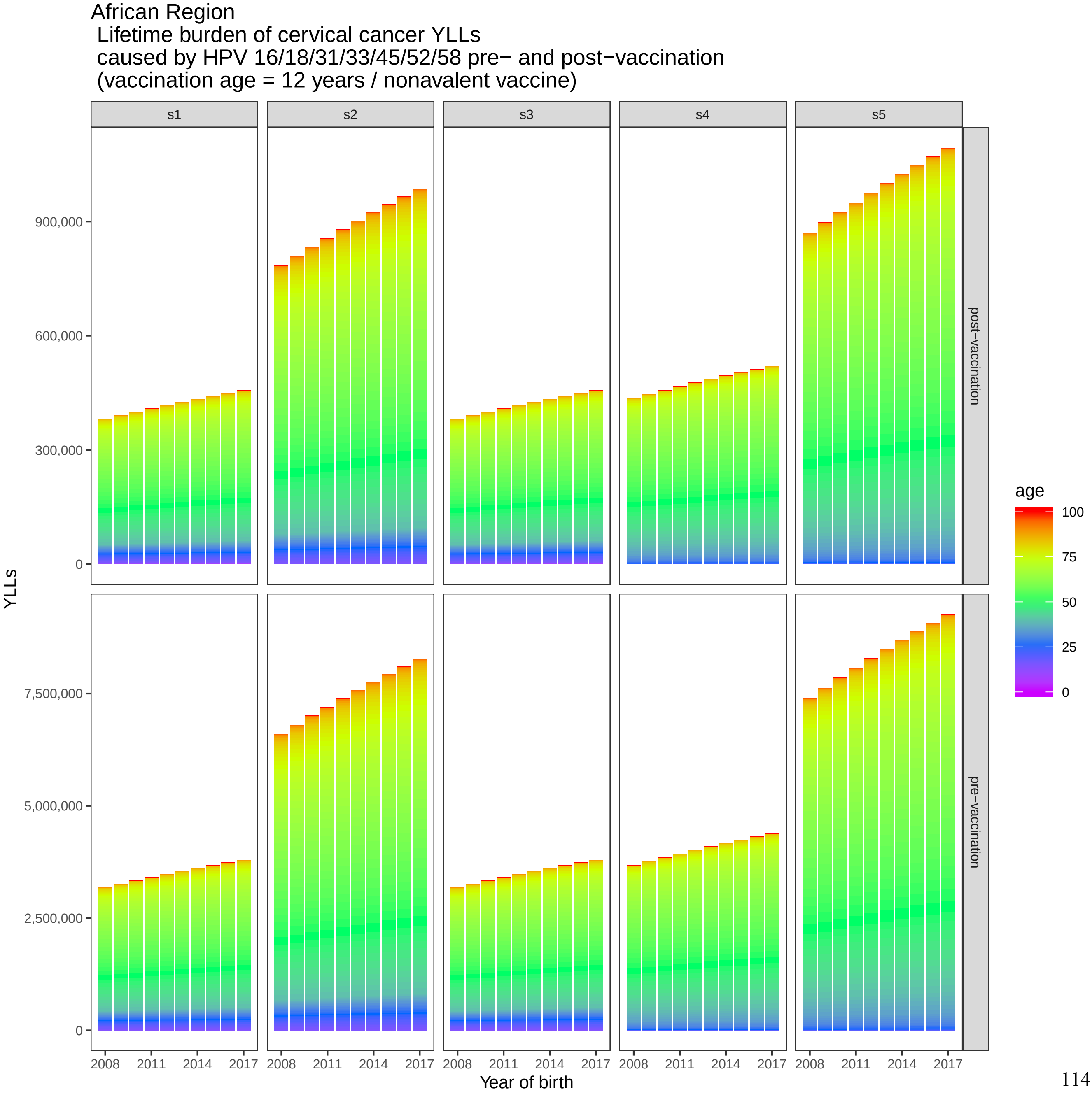
African Region
Lifetime burden of cervical cancer YLLs
caused by HPV 16/18/31/33/45/52/58 pre− and post−vaccination
(vaccination age = 12 years / nonavalent vaccine)
s1
s2
s3
s4
s5
post−vaccination
pre−vaccination
YLLs
900,000
600,000
300,000
0
7,500,000
5,000,000
2,500,000
0
2008
2011
2014
2017
Year of birth
age
100
75
50
25
0

African Region
Lifetime burden of cervical cancer DALYs
caused by HPV 16/18/31/33/45/52/58 pre− and post−vaccination
(vaccination age = 12 years / nonavalent vaccine)

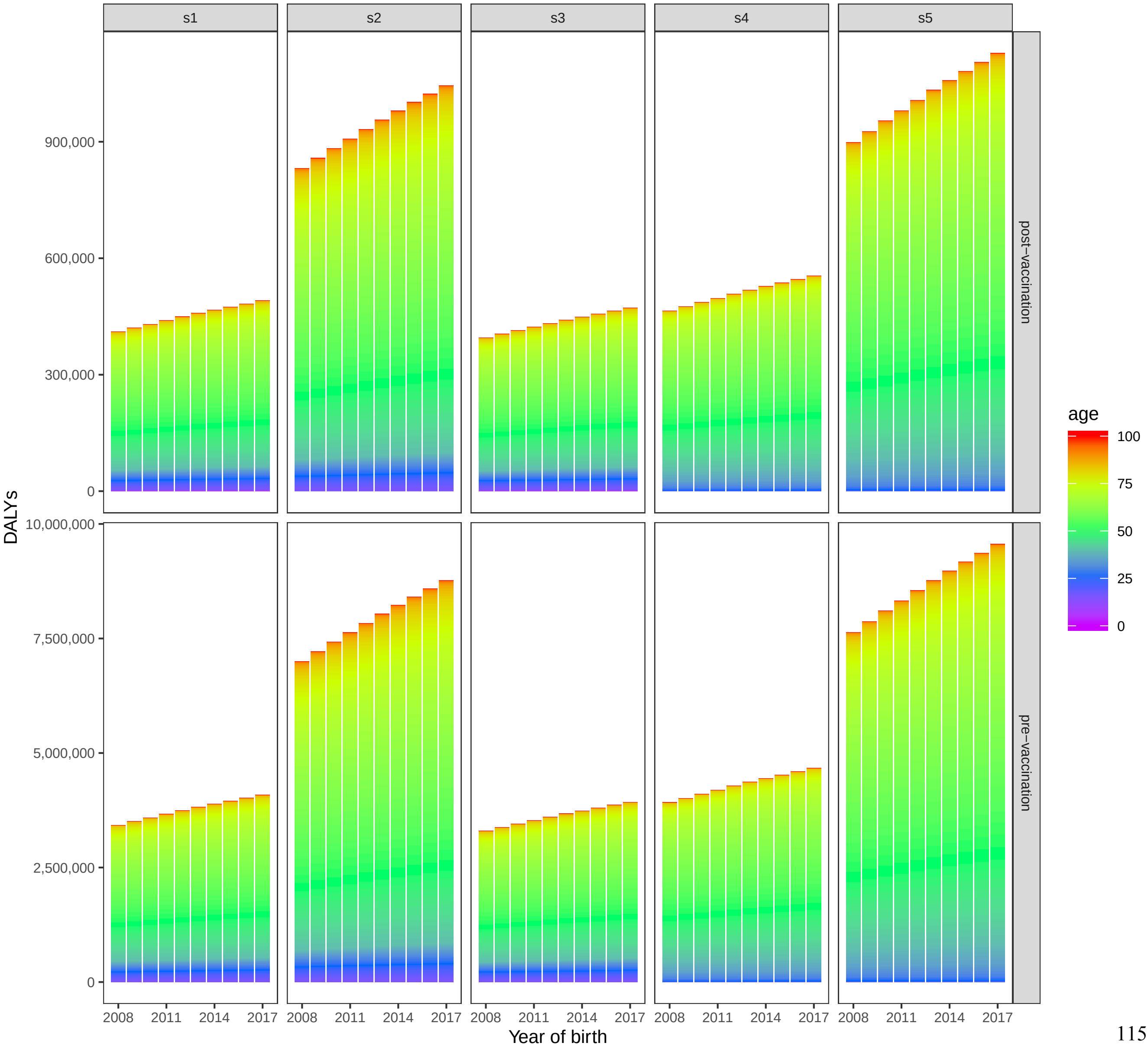

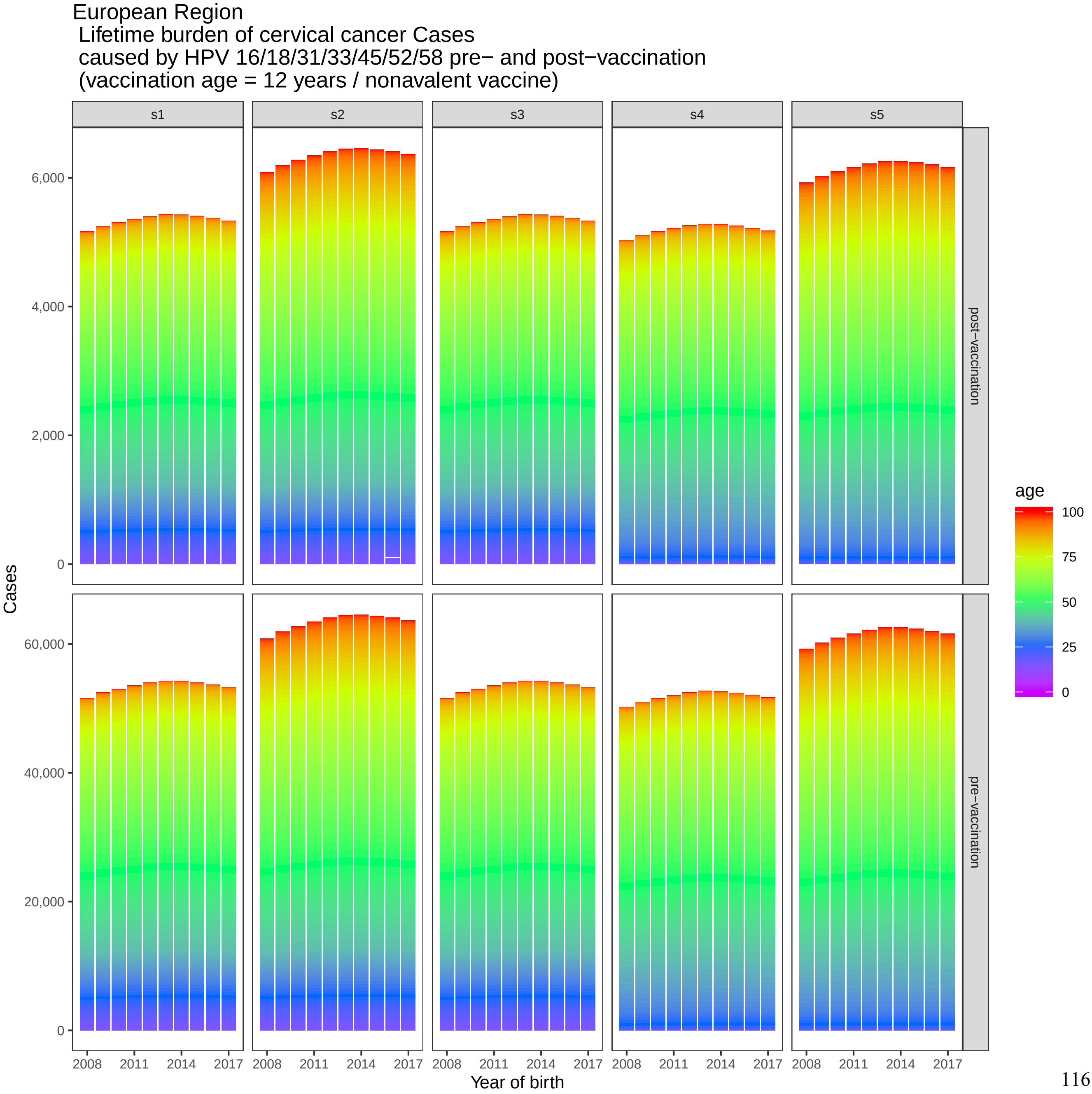
European Region
Lifetime burden of cervical cancer Cases
caused by HPV 16/18/31/33/45/52/58 pre− and post−vaccination
(vaccination age = 12 years / nonavalent vaccine)
s1
s2
s3
s4
s5
post−vaccination
pre−vaccination
Cases
Year of birth
2008
2011
2014
2017
0
2,000
4,000
6,000
20,000
40,000
60,000
age
0
25
50
75
100

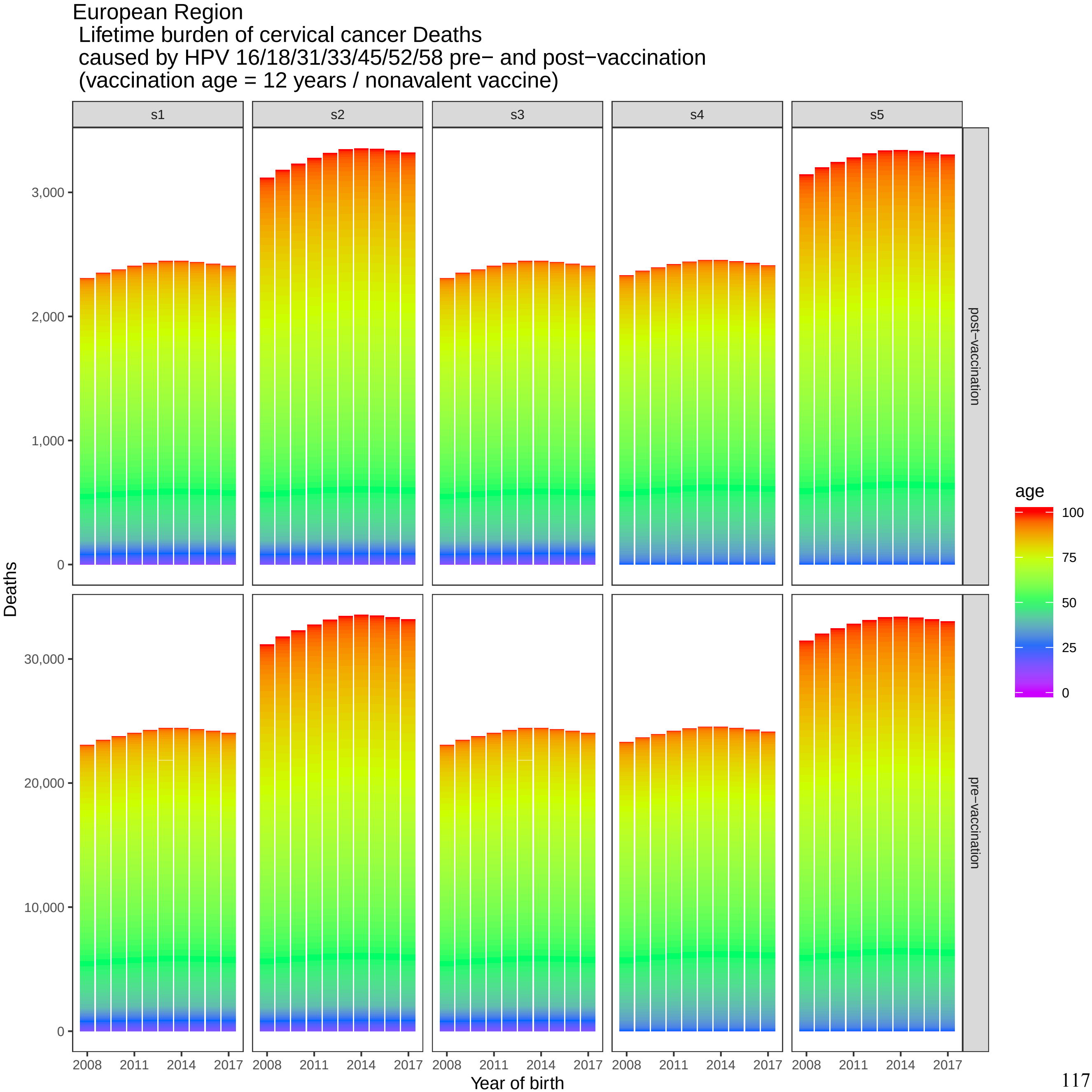

European Region
Lifetime burden of cervical cancer Deaths
caused by HPV 16/18/31/33/45/52/58 pre− and post−vaccination
(vaccination age = 12 years / nonavalent vaccine)
s1
s2
s3
s4
s5
post−vaccination
pre−vaccination
Deaths
3,000
2,000
1,000
0
30,000
20,000
10,000
0
2008
2011
2014
2017
Year of birth
age
100
75
50
25
0

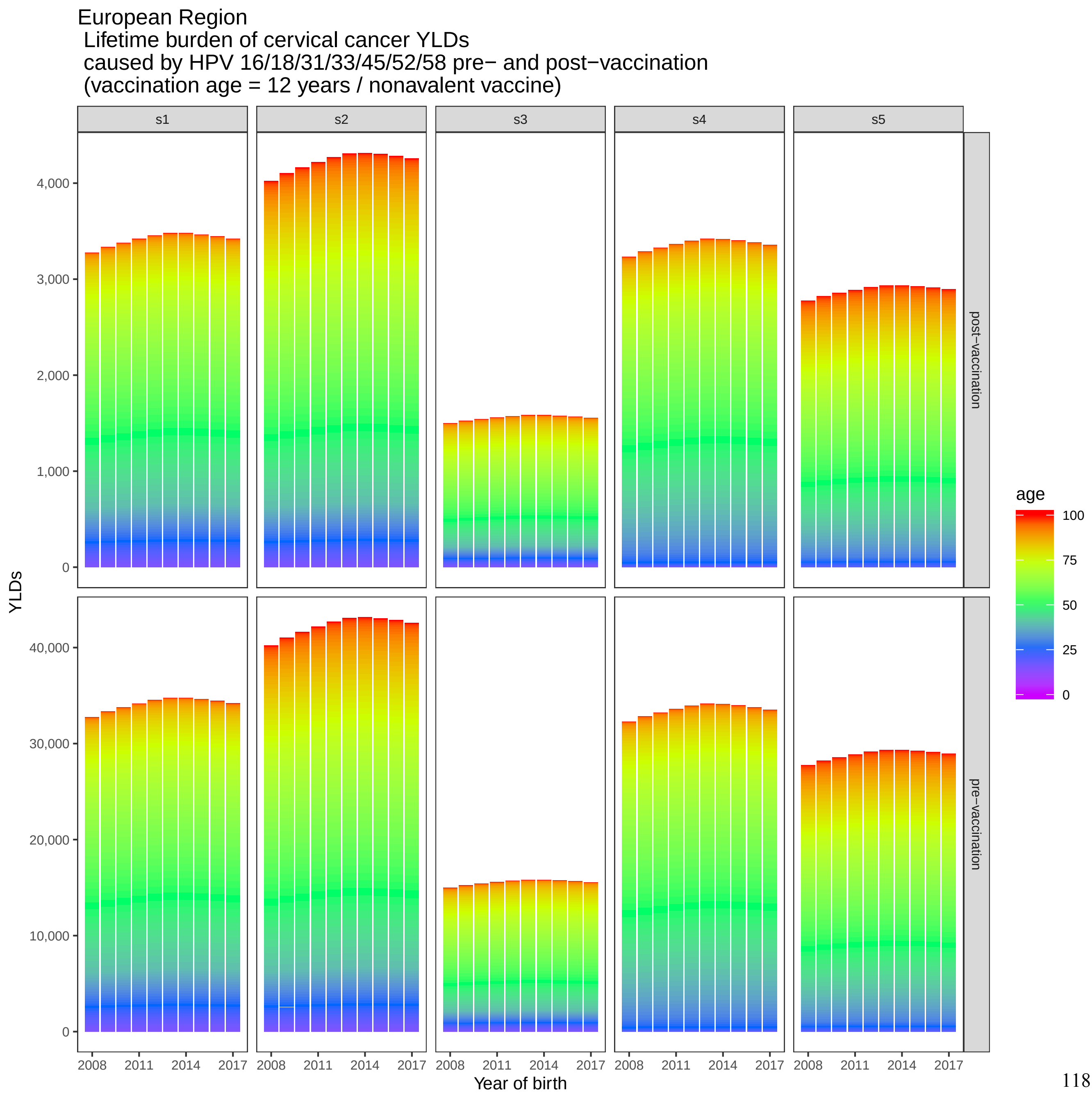
European Region
Lifetime burden of cervical cancer YLDs
caused by HPV 16/18/31/33/45/52/58 pre− and post−vaccination
(vaccination age = 12 years / nonavalent vaccine)
s1
s2
s3
s4
s5
post−vaccination
pre−vaccination
YLDs
Year of birth
age
0
25
50
75
100
0
1,000
2,000
3,000
4,000
10,000
20,000
30,000
40,000
2008
2011
2014
2017

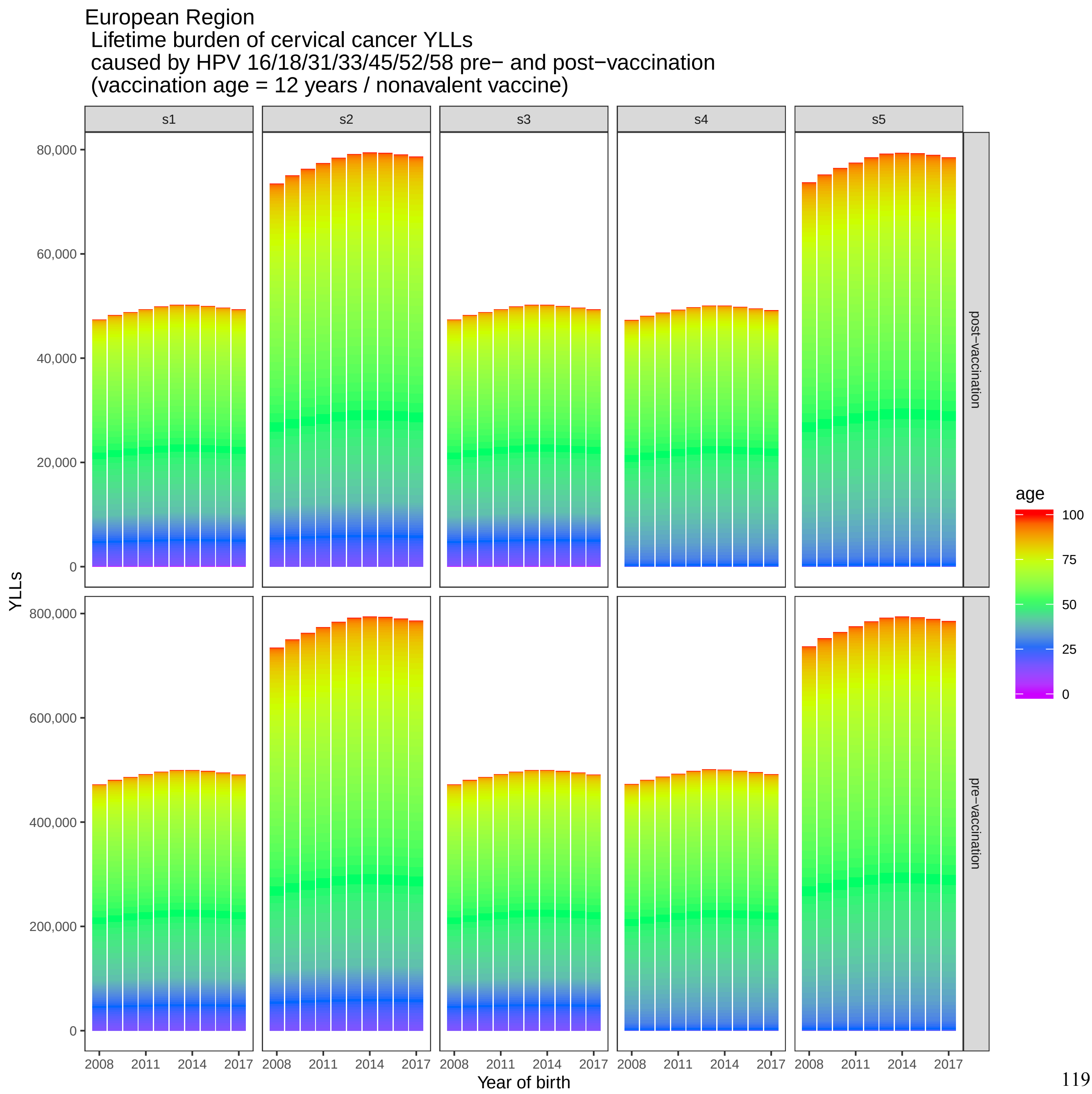

European Region
Lifetime burden of cervical cancer YLLs
caused by HPV 16/18/31/33/45/52/58 pre− and post−vaccination
(vaccination age = 12 years / nonavalent vaccine)
s1
s2
s3
s4
s5
post−vaccination
pre−vaccination
YLLs
80,000
60,000
40,000
20,000
0
800,000
600,000
400,000
200,000
0
2008
2011
2014
2017
Year of birth
age
100
75
50
25
0

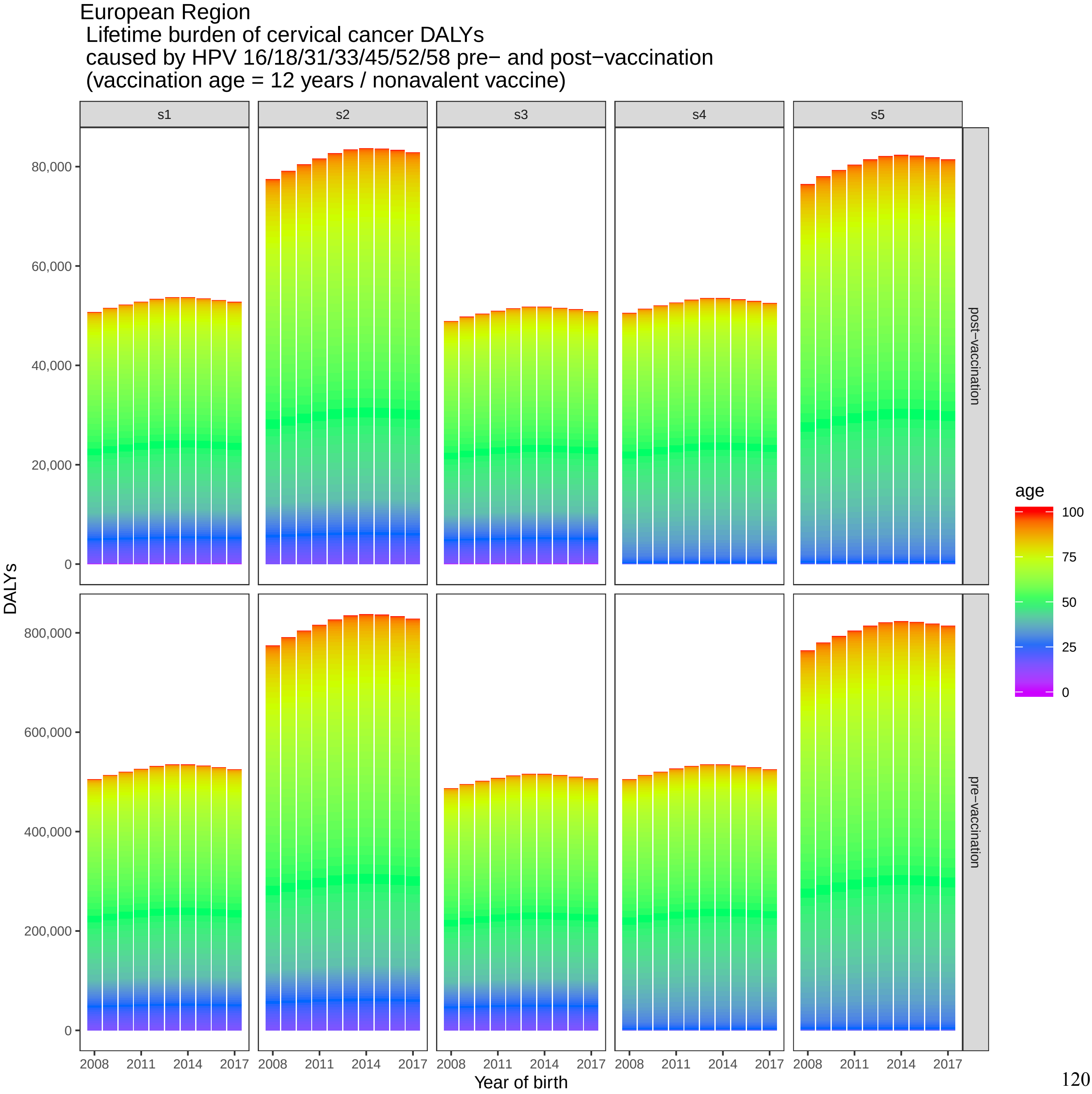

European Region
Lifetime burden of cervical cancer DALYs
caused by HPV 16/18/31/33/45/52/58 pre− and post−vaccination
(vaccination age = 12 years / nonavalent vaccine)
s1
s2
s3
s4
s5
post−vaccination
pre−vaccination
DALYs
0
20,000
40,000
60,000
80,000
0
200,000
400,000
600,000
800,000
2008
2011
2014
2017
Year of birth
age
100
75
50
25
0

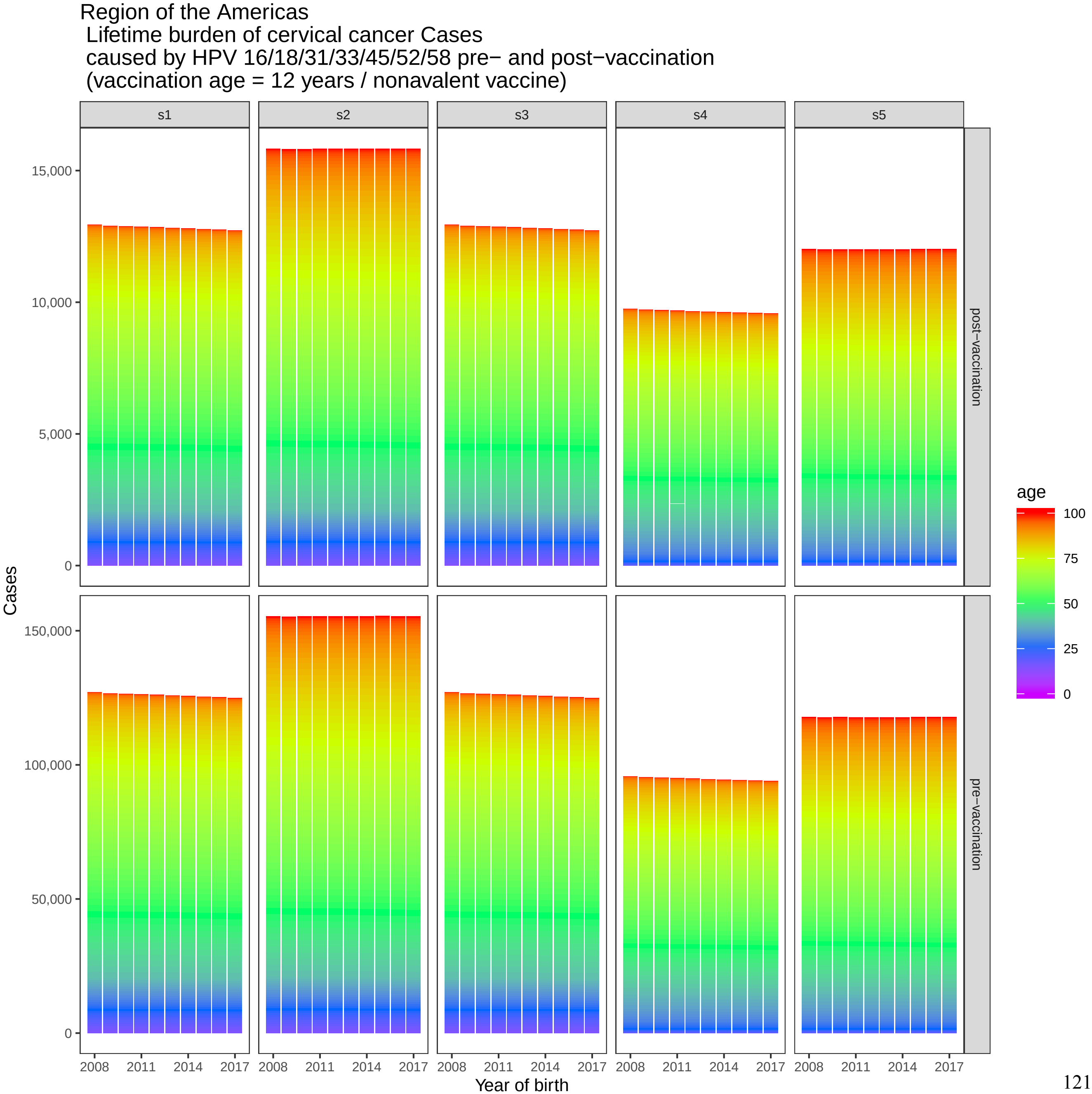
Region of the Americas
Lifetime burden of cervical cancer Cases
caused by HPV 16/18/31/33/45/52/58 pre− and post−vaccination
(vaccination age = 12 years / nonavalent vaccine)
s1
s2
s3
s4
s5
post−vaccination
pre−vaccination
Cases
15,000
10,000
5,000
0
150,000
100,000
50,000
0
2008
2011
2014
2017
Year of birth
age
100
75
50
25
0

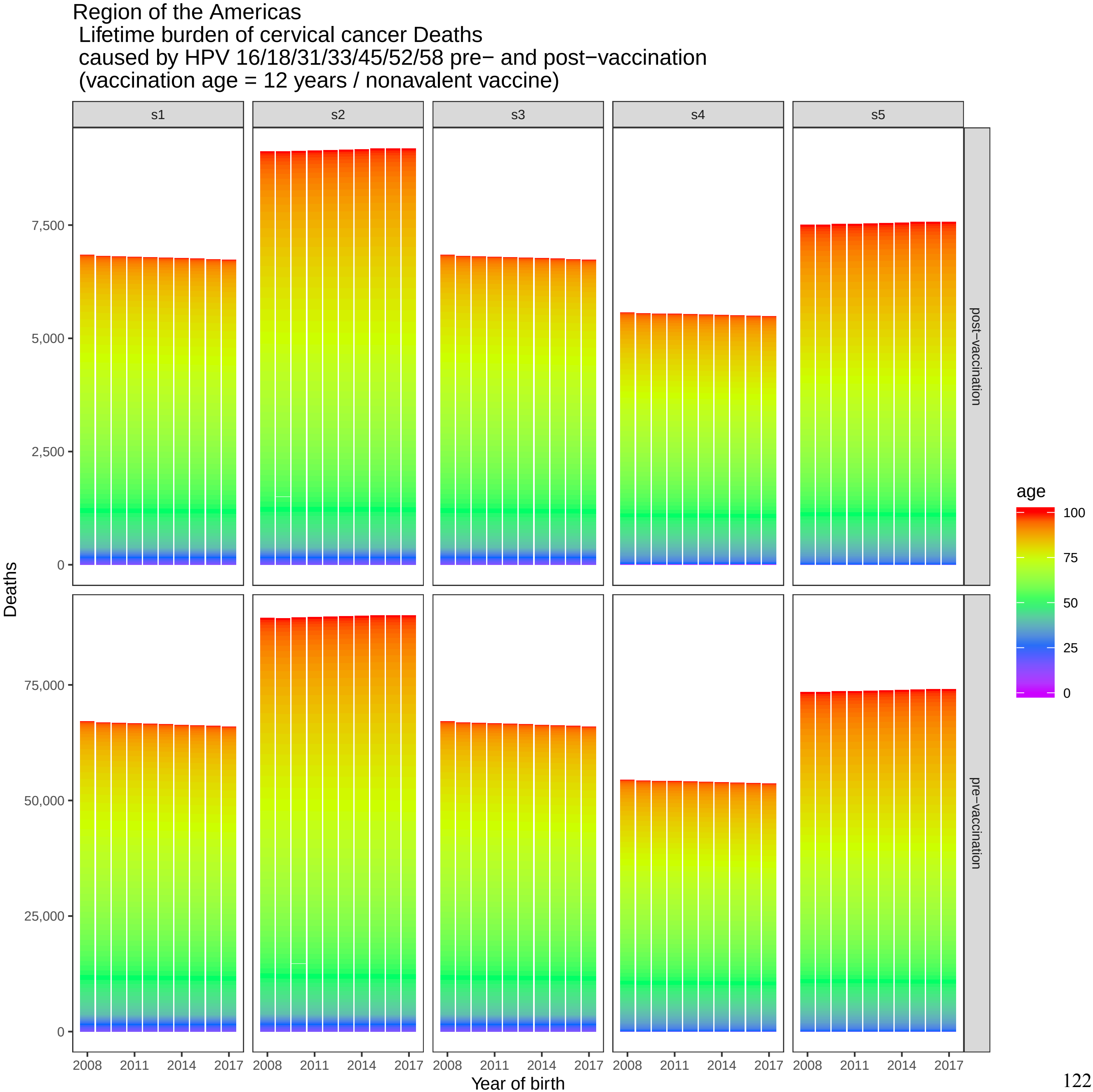
Region of the Americas
Lifetime burden of cervical cancer Deaths
caused by HPV 16/18/31/33/45/52/58 pre− and post−vaccination
(vaccination age = 12 years / nonavalent vaccine)
s1
s2
s3
s4
s5
post−vaccination
pre−vaccination
Deaths
7,500
5,000
2,500
0
75,000
50,000
25,000
0
Year of birth
2008
2011
2014
2017
age
100
75
50
25
0

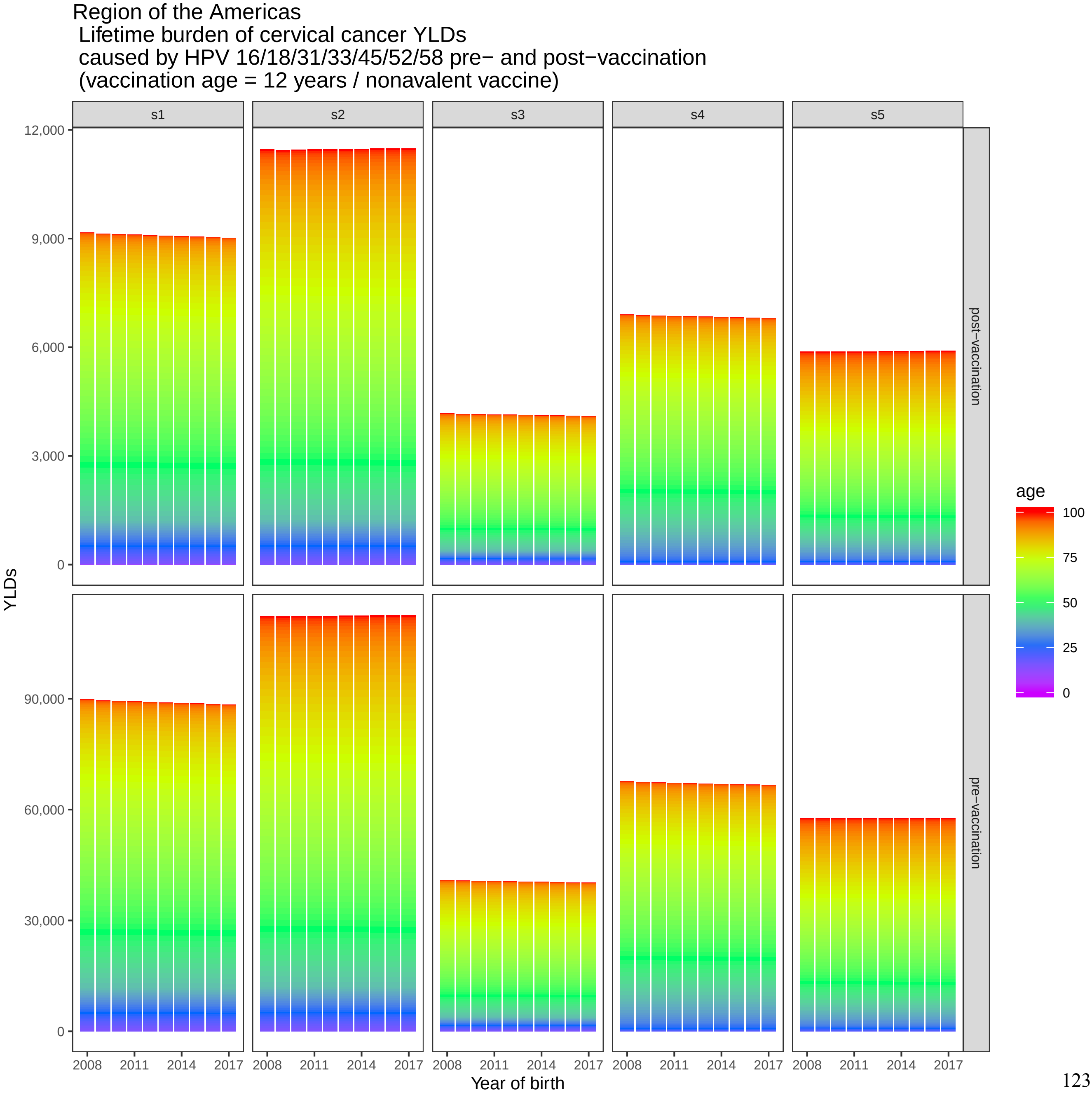
Region of the Americas
Lifetime burden of cervical cancer YLDs
caused by HPV 16/18/31/33/45/52/58 pre− and post−vaccination
(vaccination age = 12 years / nonavalent vaccine)
s1
s2
s3
s4
s5
post−vaccination
pre−vaccination
YLDs
Year of birth
age
0
25
50
75
100
12,000
9,000
6,000
3,000
90,000
60,000
30,000
2008
2011
2014
2017

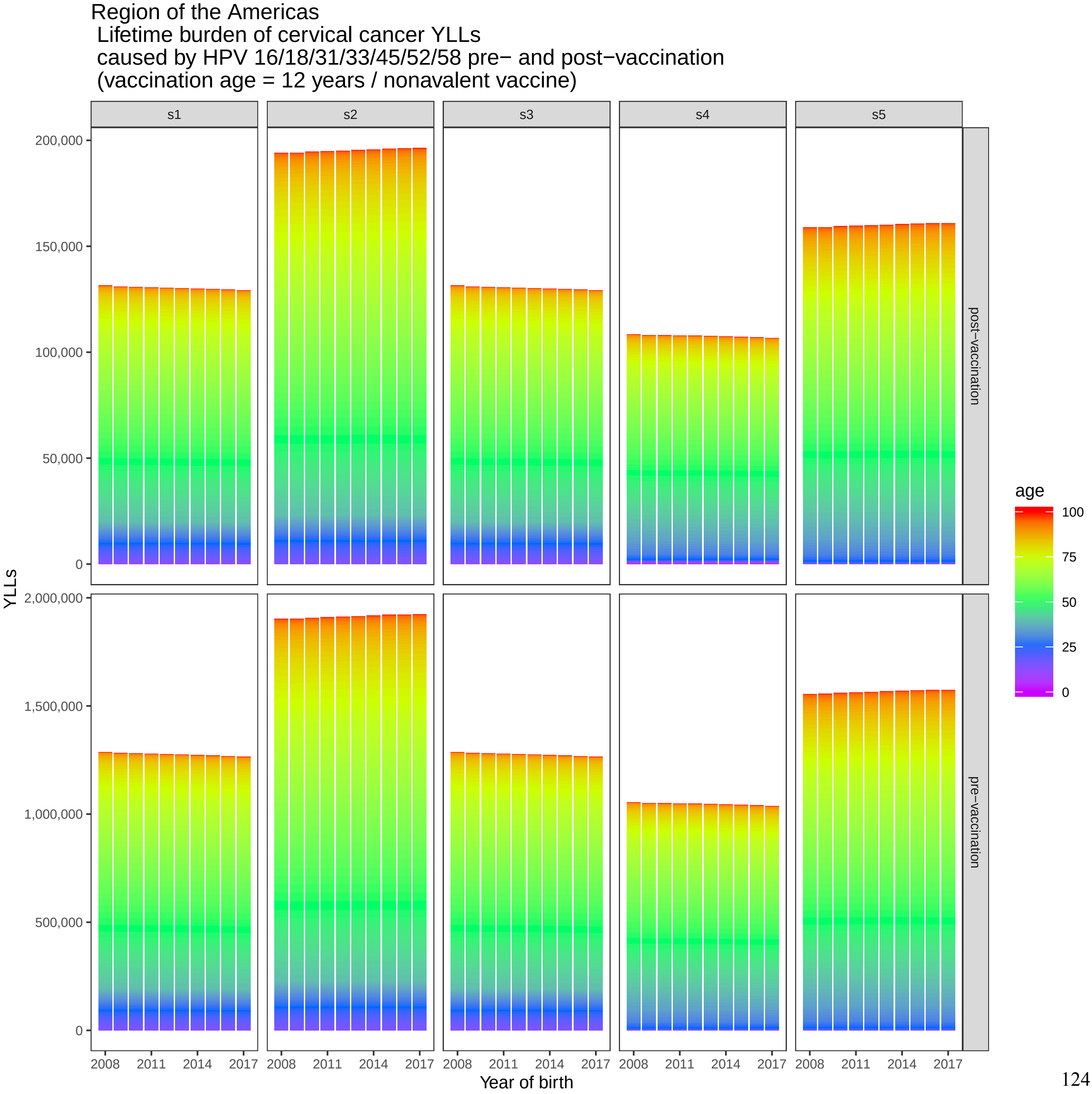

Region of the Americas
Lifetime burden of cervical cancer YLLs
caused by HPV 16/18/31/33/45/52/58 pre− and post−vaccination
(vaccination age = 12 years / nonavalent vaccine)
s1
s2
s3
s4
s5
post−vaccination
pre−vaccination
YLLs
0
50,000
100,000
150,000
200,000
500,000
1,000,000
1,500,000
2,000,000
2008
2011
2014
2017
Year of birth
age
0
25
50
75
100

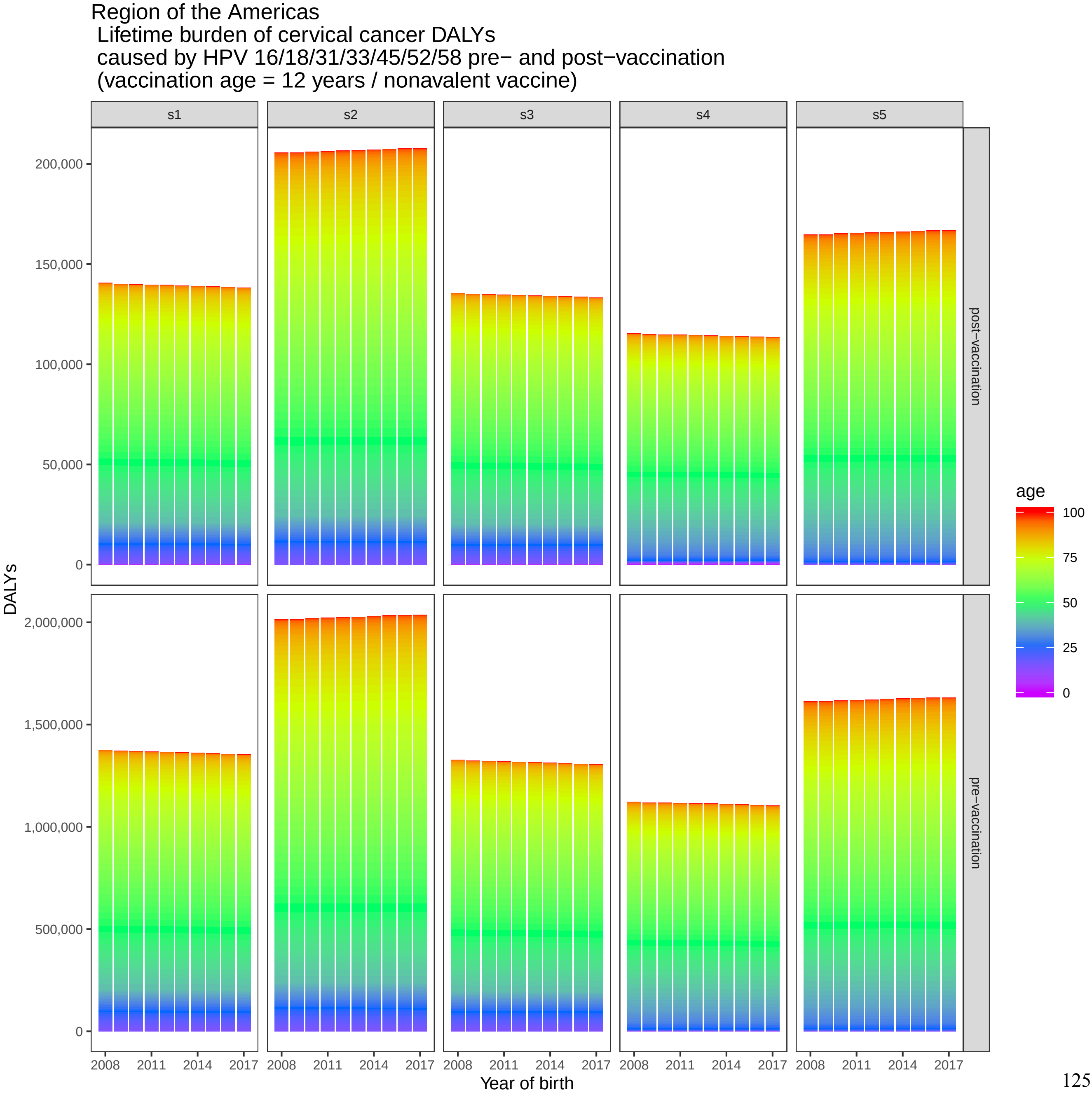
Region of the Americas
Lifetime burden of cervical cancer DALYs
caused by HPV 16/18/31/33/45/52/58 pre− and post−vaccination
(vaccination age = 12 years / nonavalent vaccine)
s1
s2
s3
s4
s5
post−vaccination
pre−vaccination
DALYs
Year of birth
age
200,000
150,000
100,000
50,000
0
2,000,000
1,500,000
1,000,000
500,000
2008
2011
2014
2017
100
75
50
25

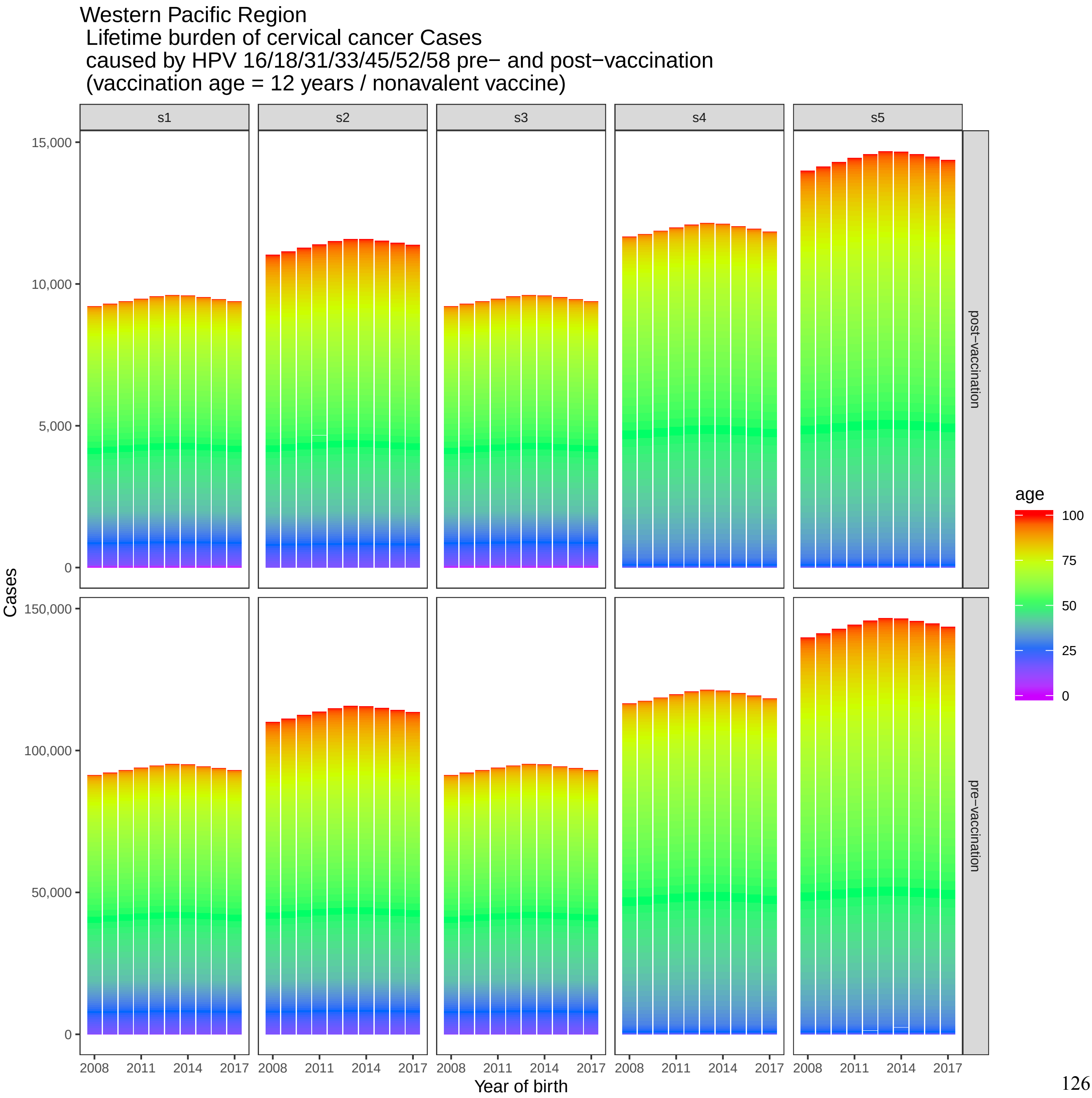
Western Pacific Region
Lifetime burden of cervical cancer Cases
caused by HPV 16/18/31/33/45/52/58 pre− and post−vaccination
(vaccination age = 12 years / nonavalent vaccine)
s1
s2
s3
s4
s5
post−vaccination
pre−vaccination
Cases
Year of birth
age
100
75
50
25
0
15,000
10,000
5,000
0
150,000
100,000
50,000
2008
2011
2014
2017

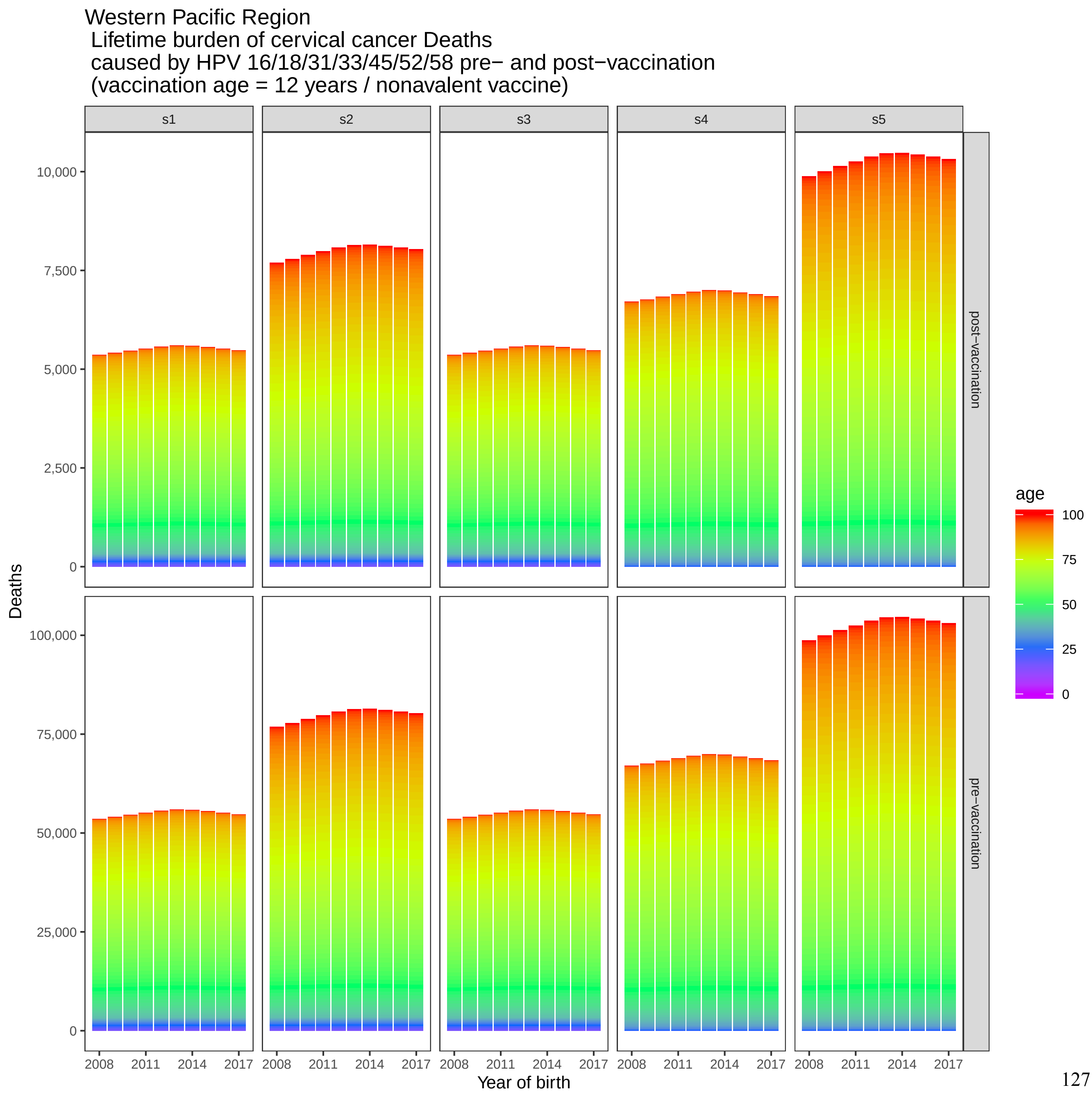
Western Pacific Region
Lifetime burden of cervical cancer Deaths
caused by HPV 16/18/31/33/45/52/58 pre− and post−vaccination
(vaccination age = 12 years / nonavalent vaccine)
s1
s2
s3
s4
s5
post−vaccination
pre−vaccination
Deaths
Year of birth
age
0
25
50
75
100
0
2,500
5,000
7,500
10,000
0
25,000
50,000
75,000
100,000
2008
2011
2014
2017

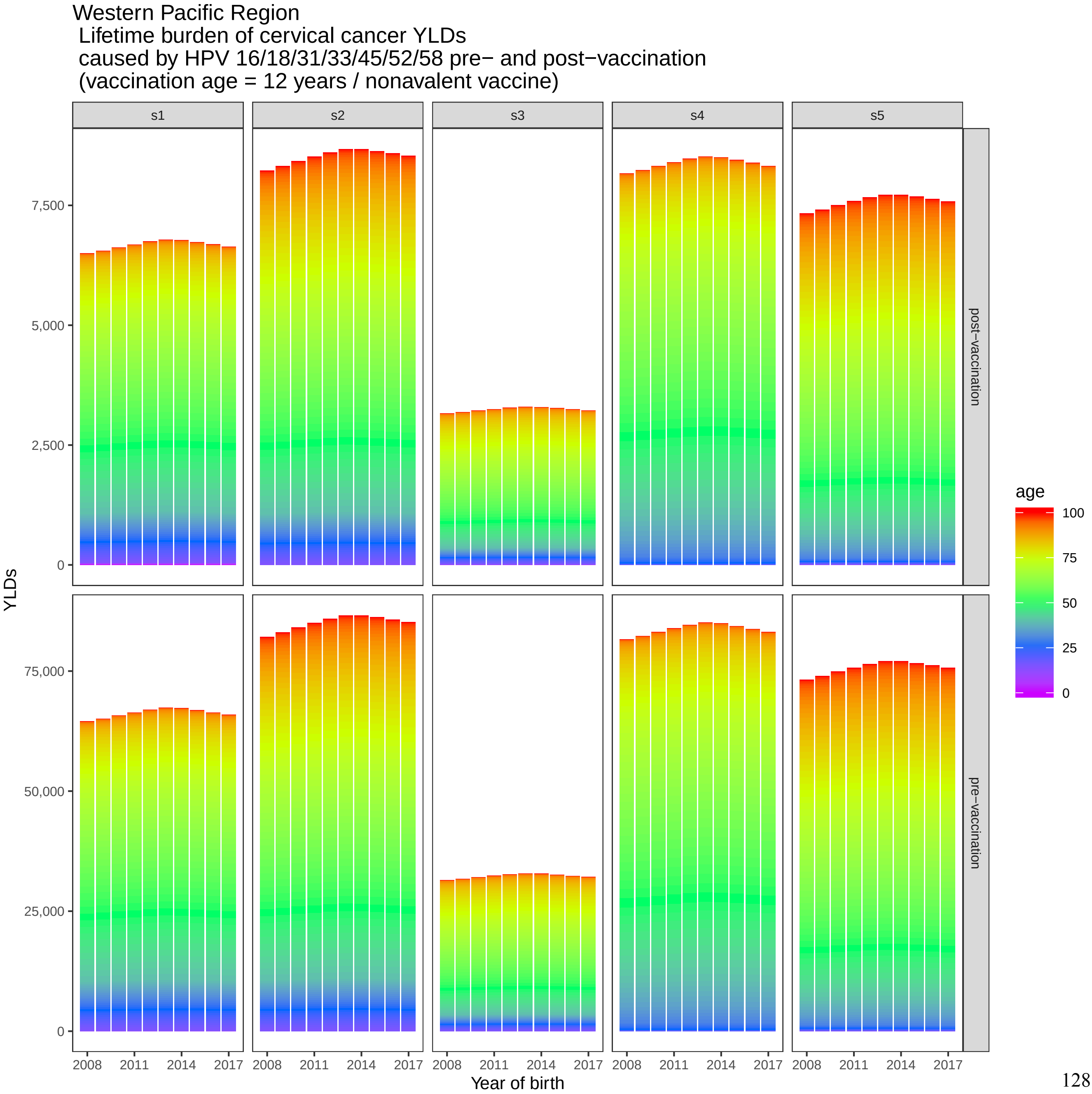

Western Pacific Region
Lifetime burden of cervical cancer YLDs
caused by HPV 16/18/31/33/45/52/58 pre− and post−vaccination
(vaccination age = 12 years / nonavalent vaccine)
s1
s2
s3
s4
s5
post−vaccination
pre−vaccination
YLDs
0
2,500
5,000
7,500
25,000
50,000
75,000
age
100
75
50
25
0
2008
2011
2014
2017
Year of birth

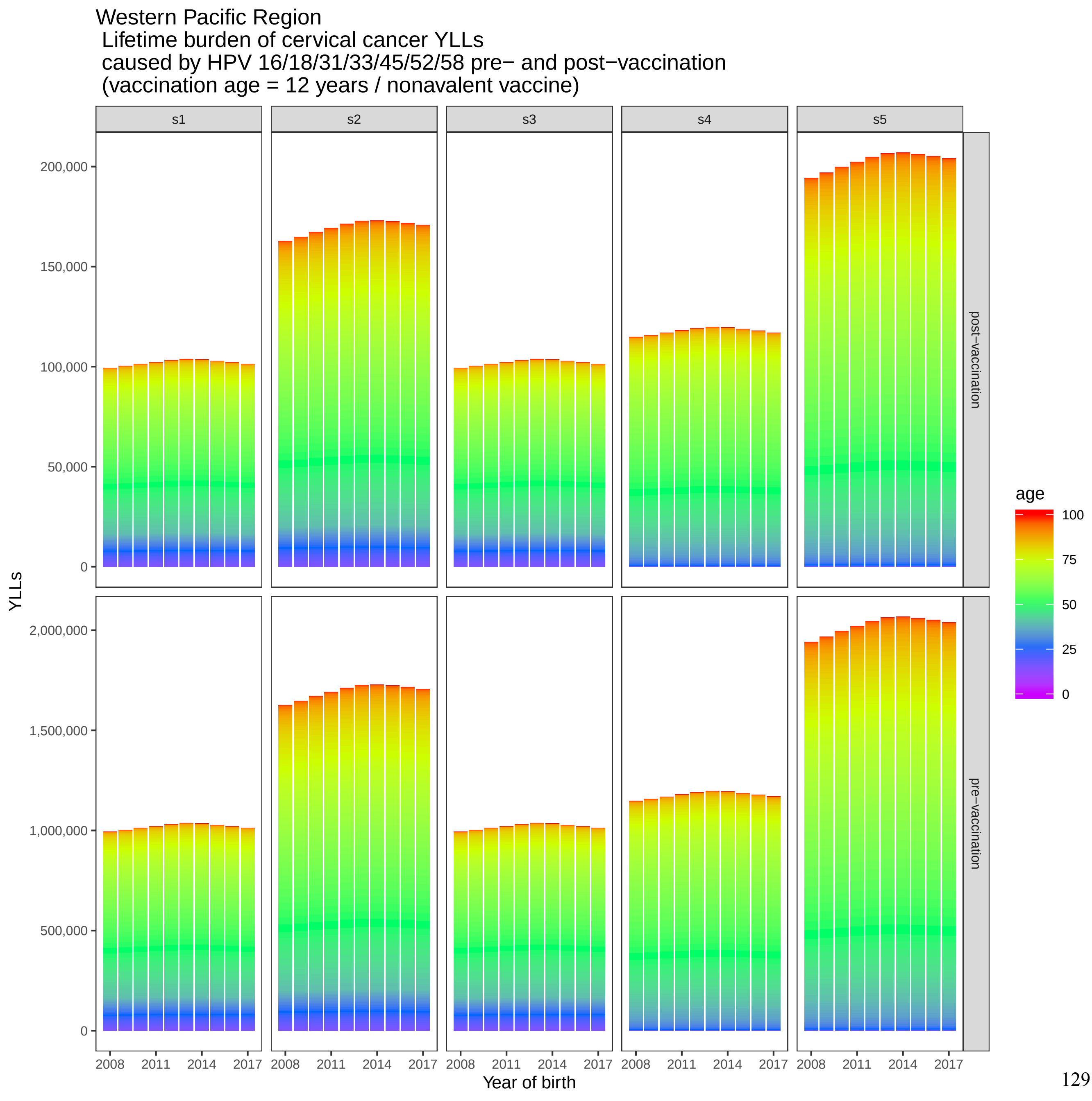
Western Pacific Region
Lifetime burden of cervical cancer YLLs
caused by HPV 16/18/31/33/45/52/58 pre− and post−vaccination
(vaccination age = 12 years / nonavalent vaccine)
s1
s2
s3
s4
s5
post−vaccination
pre−vaccination
YLLs
Year of birth
age
0
25
50
75
100
200,000
150,000
100,000
50,000
2,000,000
1,500,000
1,000,000
500,000
2008
2011
2014
2017

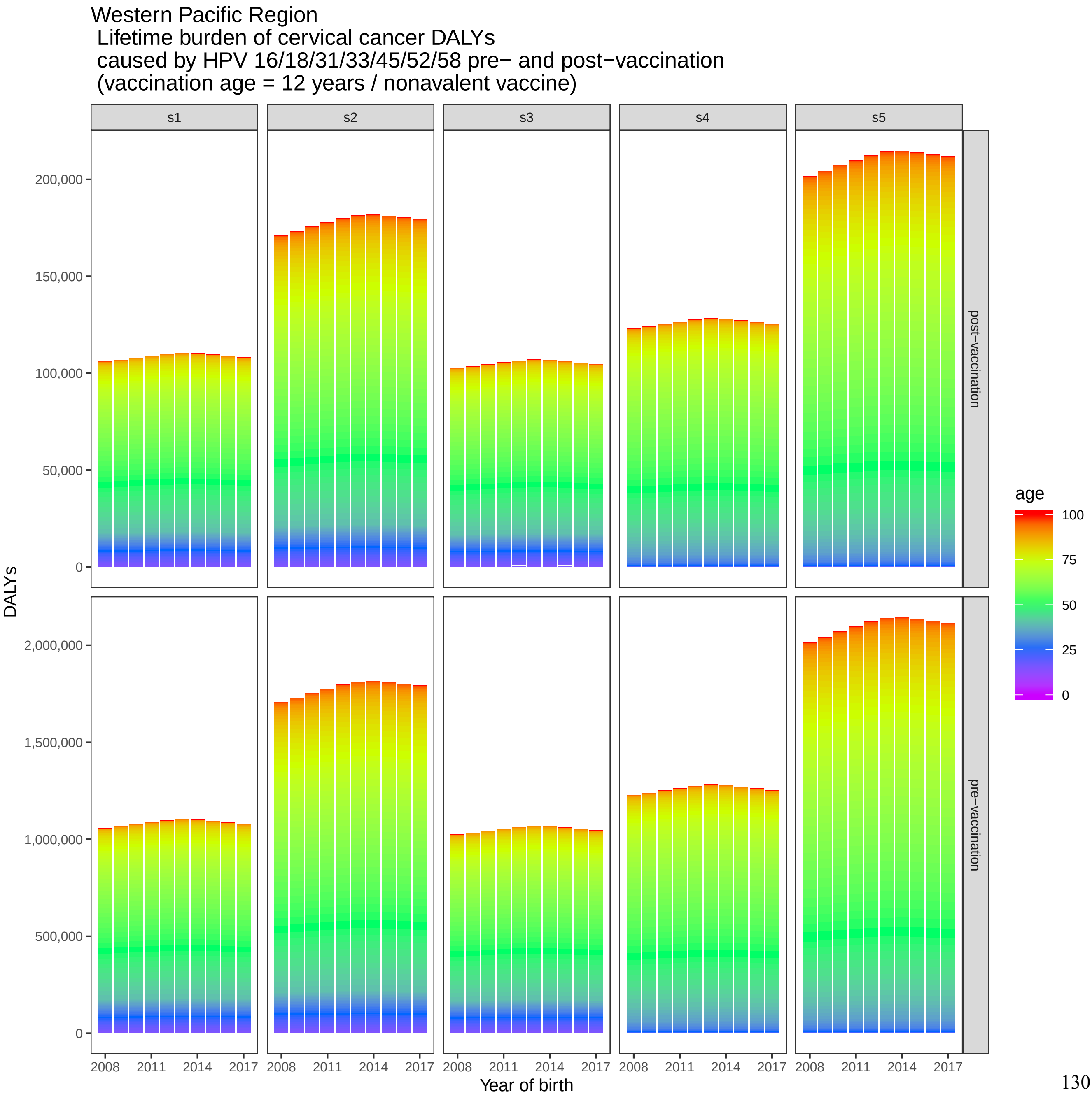
Western Pacific Region
Lifetime burden of cervical cancer DALYs
caused by HPV 16/18/31/33/45/52/58 pre− and post−vaccination
(vaccination age = 12 years / nonavalent vaccine)
s1
s2
s3
s4
s5
post−vaccination
pre−vaccination
DALYs
200,000
150,000
100,000
50,000
0
2,000,000
1,500,000
1,000,000
500,000
0
2008
2011
2014
2017
Year of birth
age
100
75
50
25
0

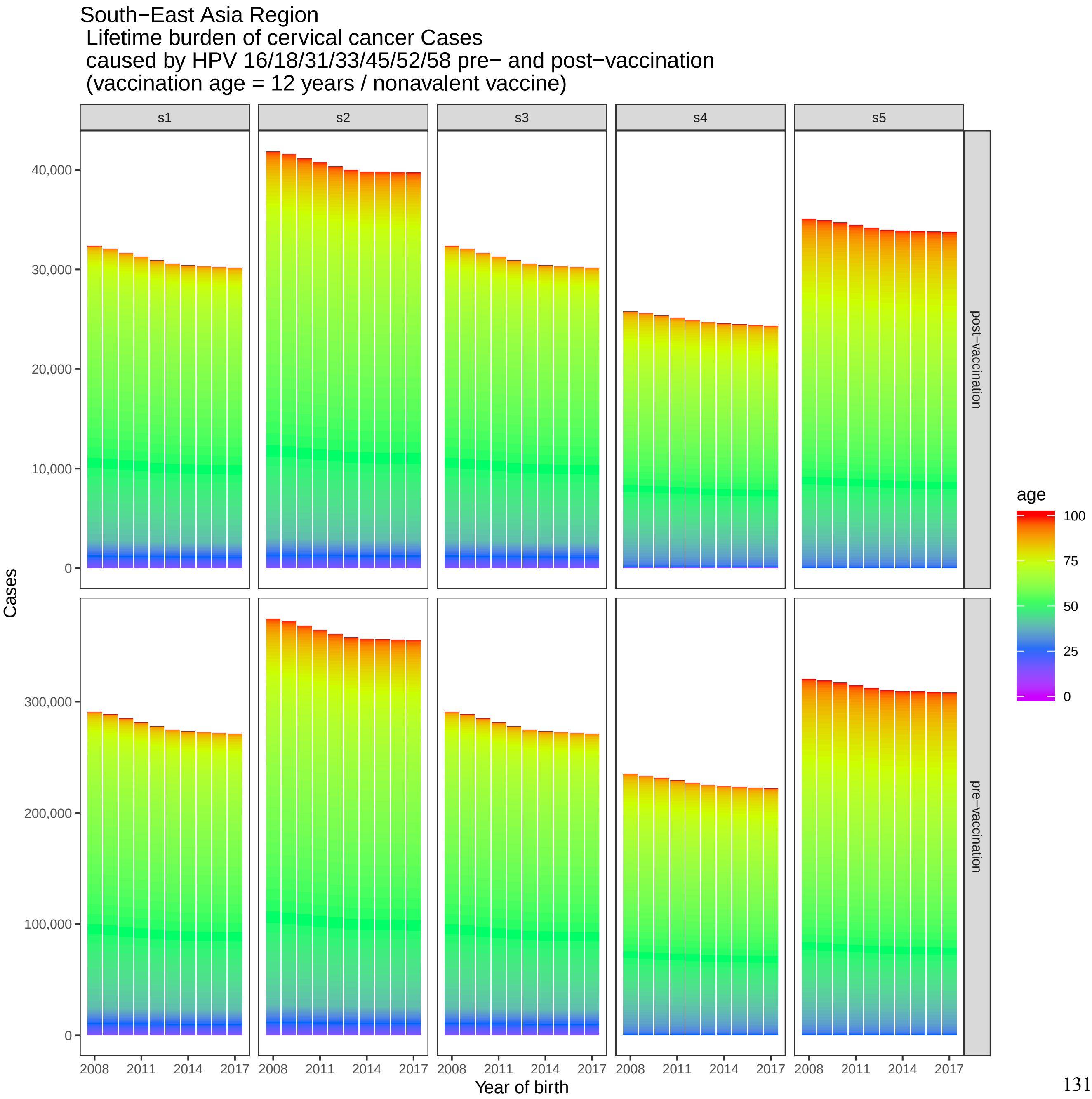

South−East Asia Region
Lifetime burden of cervical cancer Cases
caused by HPV 16/18/31/33/45/52/58 pre− and post−vaccination
(vaccination age = 12 years / nonavalent vaccine)
s1
s2
s3
s4
s5
post−vaccination
pre−vaccination
Cases
Year of birth
age
0
25
50
75
100
0
10,000
20,000
30,000
40,000
100,000
200,000
300,000
2008
2011
2014
2017

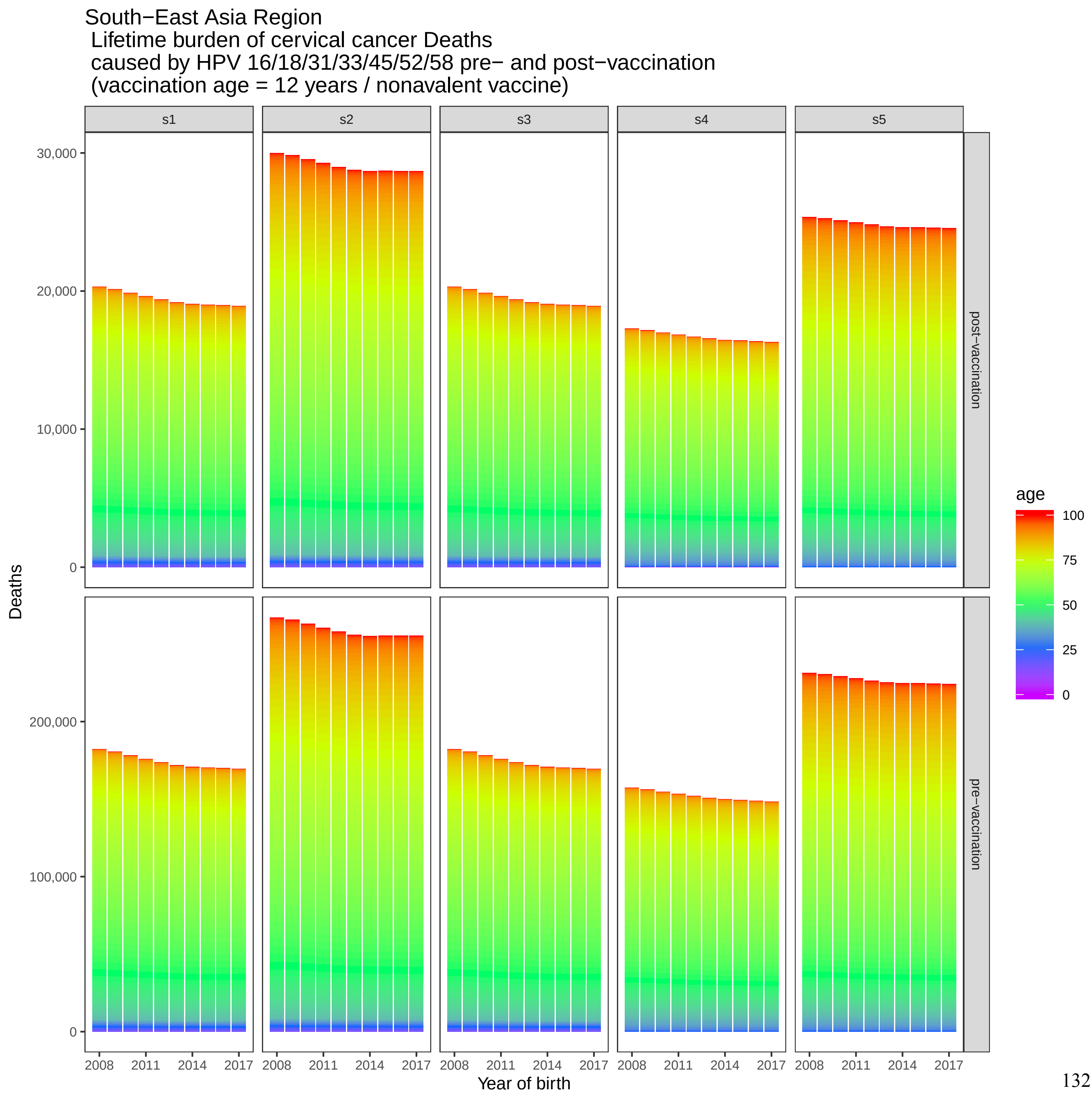
South−East Asia Region
Lifetime burden of cervical cancer Deaths
caused by HPV 16/18/31/33/45/52/58 pre− and post−vaccination
(vaccination age = 12 years / nonavalent vaccine)
s1
s2
s3
s4
s5
post−vaccination
pre−vaccination
Deaths
30,000
20,000
10,000
0
200,000
100,000
0
Year of birth
2008
2011
2014
2017
age
100
75
50
25
0

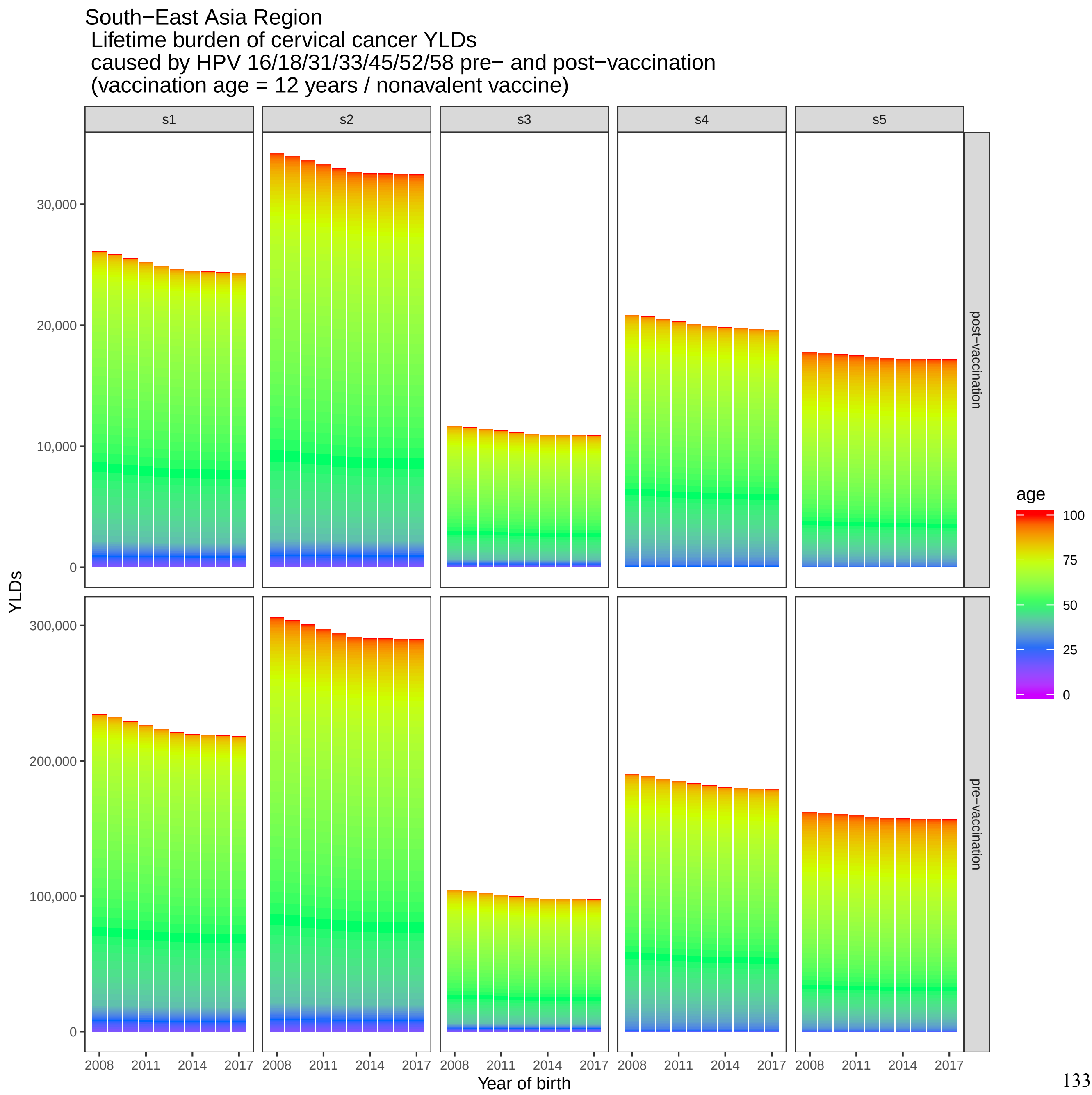
South−East Asia Region
Lifetime burden of cervical cancer YLDs
caused by HPV 16/18/31/33/45/52/58 pre− and post−vaccination
(vaccination age = 12 years / nonavalent vaccine)
s1
s2
s3
s4
s5
post−vaccination
pre−vaccination
YLDs
0
10,000
20,000
30,000
100,000
200,000
300,000
2008
2011
2014
2017
Year of birth
age
0
25
50
75
100

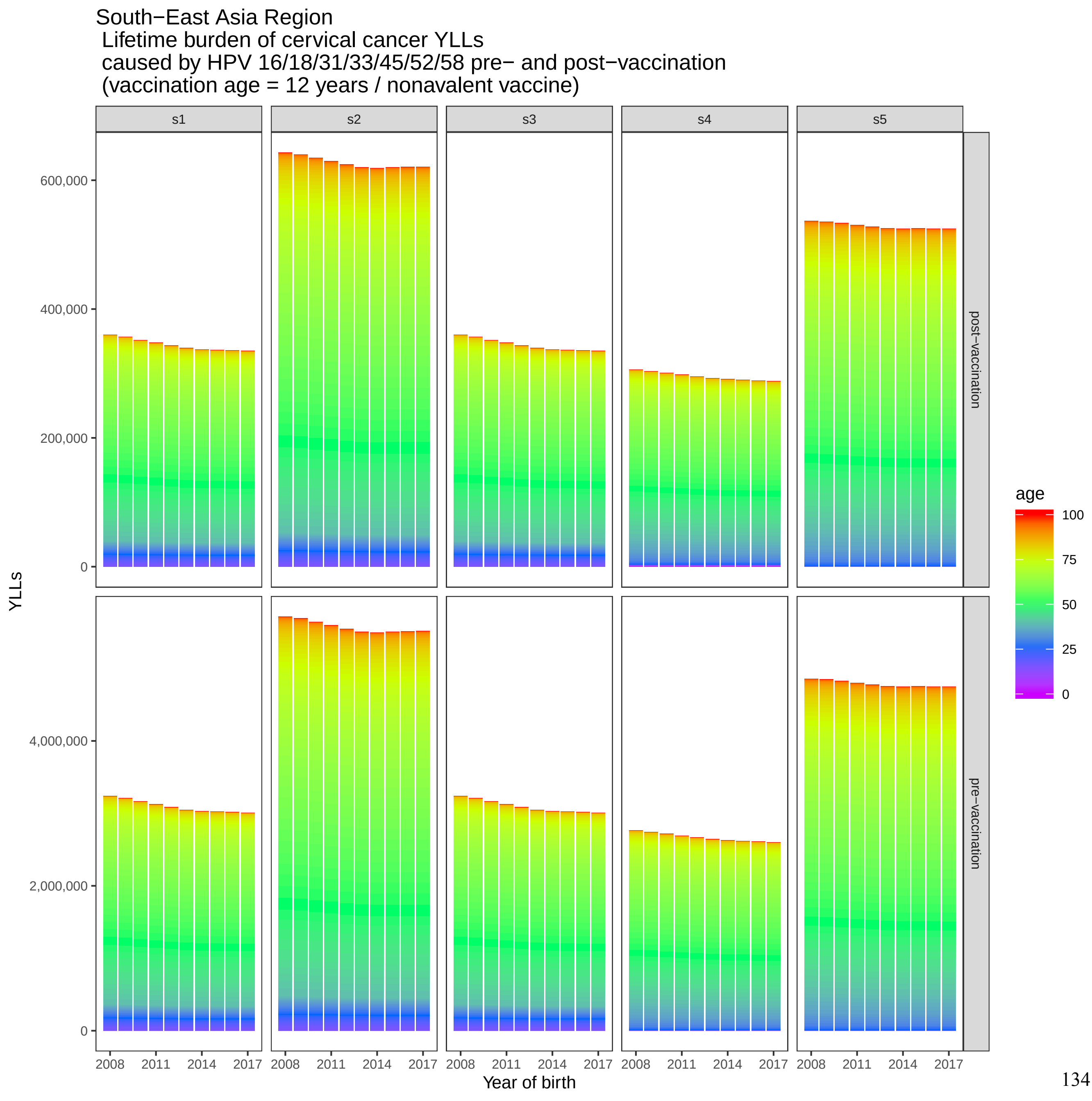
South−East Asia Region
Lifetime burden of cervical cancer YLLs
caused by HPV 16/18/31/33/45/52/58 pre− and post−vaccination
(vaccination age = 12 years / nonavalent vaccine)
s1
s2
s3
s4
s5
post−vaccination
pre−vaccination
YLLs
600,000
400,000
200,000
0
4,000,000
2,000,000
0
2008
2011
2014
2017
Year of birth
age
100
75
50
25
0

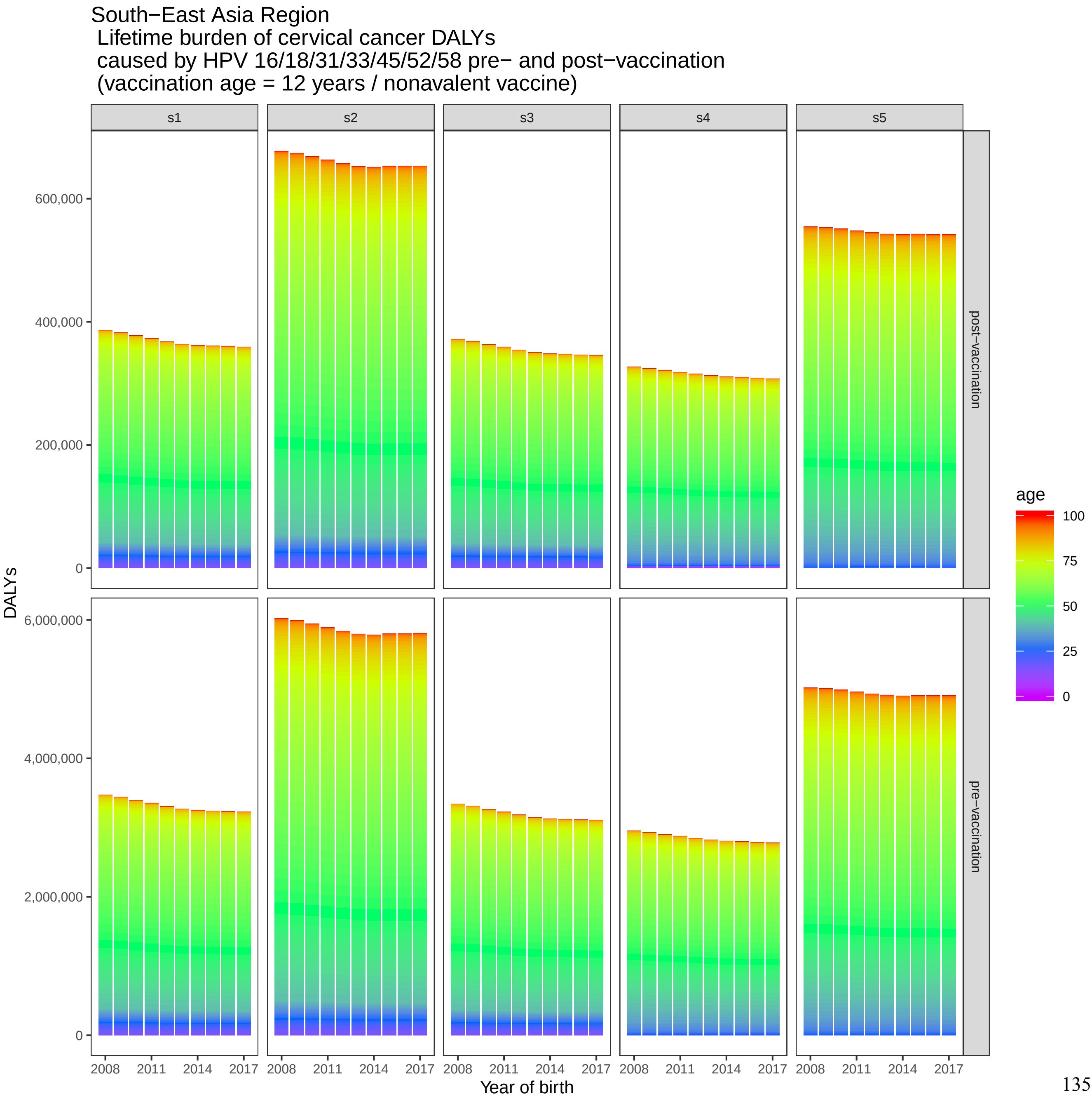
South−East Asia Region
Lifetime burden of cervical cancer DALYs
caused by HPV 16/18/31/33/45/52/58 pre− and post−vaccination
(vaccination age = 12 years / nonavalent vaccine)
s1
s2
s3
s4
s5
post−vaccination
pre−vaccination
DALYs
600,000
400,000
200,000
0
6,000,000
4,000,000
2,000,000
0
2008
2011
2014
2017
Year of birth
age
100
75
50
25
0

A7. Cervical cancer burden pre- and post-vaccination at the national level

The lifetime burden of cervical cancer in terms of cases, deaths, YLDs, YLLs and DALYs pre- and post-vaccination at 90% coverage during 2020-2029 in 177 countries for bivalent/quadrivalent and nonavalent vaccination of 9-year-old and 12-year-old girls. Cervical cancer burden pre- and post-vaccination refers to cervical cancer caused by HPV 16/18 and HPV 16/18/31/33/45/52/58 for bivalent/quadrivalent and nonavalent vaccination respectively.

See appendix 2 for data on the lifetime burden of cervical cancer.

A8. Vaccination impact per 1000 vaccinated girls at the global level

The lifetime impact of vaccination on cases, deaths, YLLs, YLDs and DALYs averted per 1000 vaccinated girls at the global level (in 177 countries) for bivalent/quadrivalent and nonavalent vaccination of 9-year-old and 12-year-old girls.

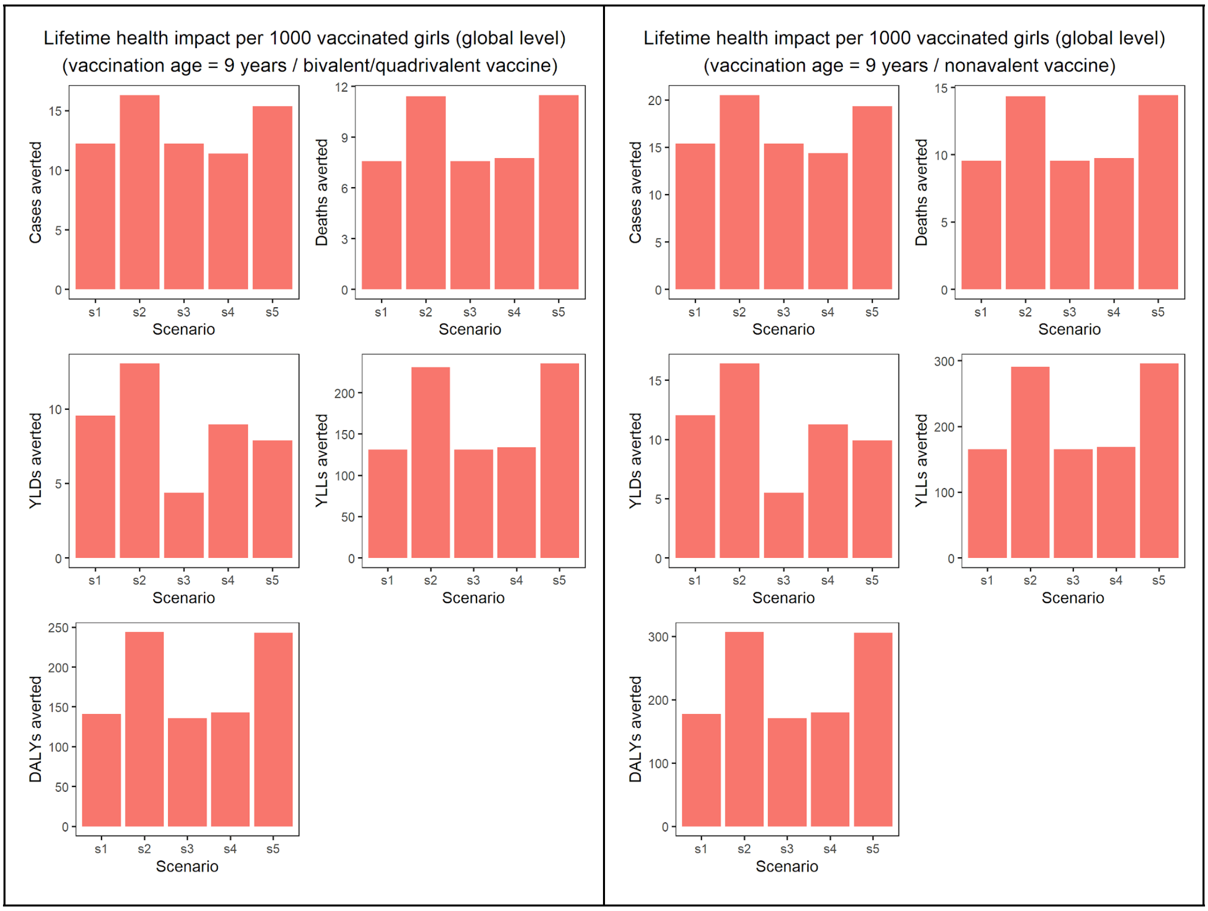

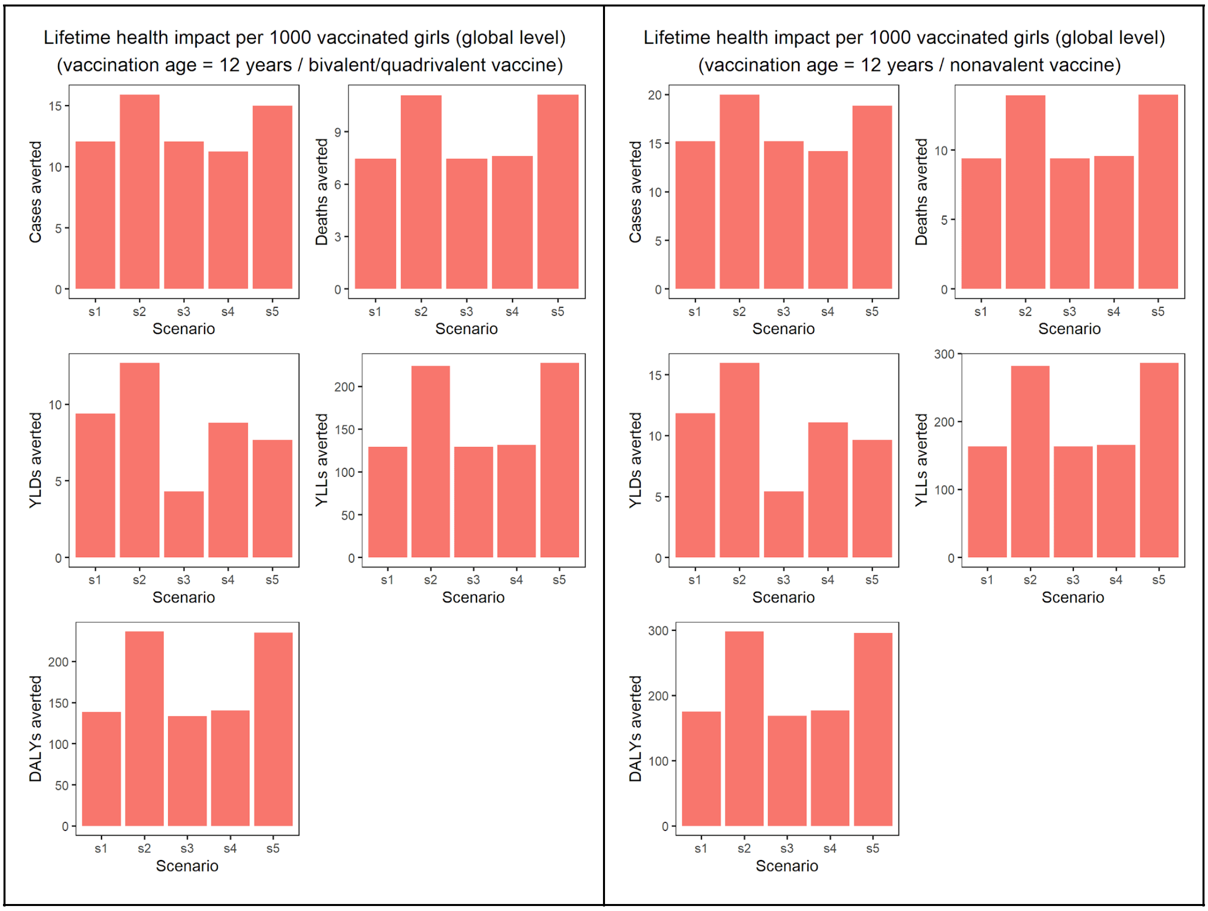

s1 - WHO 2009 demography, GBD 2001 disability weights, GLOBOCAN 2012 cervical cancer burden
s2 - UNWPP 2019 demography, GBD 2001 disability weights, GLOBOCAN 2012 cervical cancer burden
s3 - WHO 2009 demography, GBD 2017 disability weights, GLOBOCAN 2012 cervical cancer burden
s4 - WHO 2009 demography, GBD 2001 disability weights, GLOBOCAN 2018 cervical cancer burden
s5 - UNWPP 2019 demography, GBD 2017 disability weights, GLOBOCAN 2018 cervical cancer burden

A9. Global estimates of HPV vaccination impact of 12-year-old girls

Health impact of bivalent/quadrivalent and nonavalent HPV vaccination of 12-year-old girls at 90% coverage during 2020-2029 on the averted lifetime burden of cervical cancer in 177 countries for the 5 comparative scenarios.

Bivalent/quadrivalent HPV vaccination impact on cervical cancer caused by HPV 16/18					
Scenario	Cases	Deaths	YLDs	YLLs	DALYs
	averted per 1000 vaccinated girls				
s1	12.1	7.5	9.4	130	139
s2	15.9	11.1	12.7	224	237
s3	12.1	7.5	4.3	130	134
s4	11.2	7.6	8.8	132	140
s5	15	11.1	7.7	227	235
**Nonavalent HPV vaccination impact on cervical cancer caused by HPV 16/18/31/33/45/52/58**					
**Scenario**	**Cases**	**Deaths**	**YLDs**	**YLLs**	**DALYs**
	**averted per 1000 vaccinated girls**				
s1	15.2	9.4	11.9	163	175
s2	20	13.9	16	282	298
s3	15.2	9.4	5.4	163	169
s4	14.2	9.6	11.1	166	177
s5	18.9	14	9.7	286	296

s1 - WHO 2009 demography, GBD 2001 disability weights, GLOBOCAN 2012 cervical cancer burden
s2 - UNWPP 2019 demography, GBD 2001 disability weights, GLOBOCAN 2012 cervical cancer burden
s3 - WHO 2009 demography, GBD 2017 disability weights, GLOBOCAN 2012 cervical cancer burden
s4 - WHO 2009 demography, GBD 2001 disability weights, GLOBOCAN 2018 cervical cancer burden
s5 - UNWPP 2019 demography, GBD 2017 disability weights, GLOBOCAN 2018 cervical cancer burden

A10. Number of 12-year-old girls needed to be vaccinated to prevent cervical cancer

The number of 12-year-old girls to be vaccinated to prevent 1 case, 1 death, 1 year loss due to morbidity (YLD), 1 year loss due to premature mortality (YLL), or 1 year loss of healthy life (DALY) from cervical cancer for the 5 comparative scenarios (bivalent/quadrivalent and nonavalent HPV vaccination).

**Bivalent/quadrivalent HPV vaccine**					
**Scenario**	**Number of girls needed to be vaccinated to prevent cervical cancer caused by HPV 16/18**				
	**1 case**	**1 death**	**1 YLD**	**1 YLL**	**1 DALY**
s1	83	134	106	7.7	7.2
s2	63	90	79	4.5	4.2
s3	83	134	232	7.7	7.5
s4	89	132	114	7.6	7.1
s5	67	90	130	4.4	4.3
**Nonavalent HPV vaccine**					
**Scenario**	**Number of girls needed to be vaccinated to prevent cervical cancer caused by HPV 16/18/31/33/45/52/58**				
	**1 case**	**1 death**	**1 YLD**	**1 YLL**	**1 DALY**
s1	66	107	84	6.1	5.7
s2	50	72	63	3.5	3.4
s3	66	107	184	6.1	5.9
s4	71	105	90	6	5.7
s5	53	72	104	3.5	3.4

s1 - WHO 2009 demography, GBD 2001 disability weights, GLOBOCAN 2012 cervical cancer burden
s2 - UNWPP 2019 demography, GBD 2001 disability weights, GLOBOCAN 2012 cervical cancer burden
s3 - WHO 2009 demography, GBD 2017 disability weights, GLOBOCAN 2012 cervical cancer burden
s4 - WHO 2009 demography, GBD 2001 disability weights, GLOBOCAN 2018 cervical cancer burden
s5 - UNWPP 2019 demography, GBD 2017 disability weights, GLOBOCAN 2018 cervical cancer burden

A11. Vaccination impact per 1000 vaccinated 12-year-old girls at the regional level

Lifetime health impact of bivalent/quadrivalent and nonavalent HPV vaccination of 12-year-old girls during 2020-2029 on cases, deaths, YLDs, YLLs and DALYs averted per 1000 vaccinated girls in the 6 WHO regions (estimates after the combined PRIME updates for demography, disability weights and cervical cancer burden).

Bivalent/quadrivalent HPV vaccine					
WHO region	Cases	Deaths	YLDs	YLLs	DALYs
	averted per 1000 vaccinated girls				
African Region	28	22	14	441	456
South-East Asia Region	15	11	7	223	231
Region of the Americas	13	8	6	168	174
Western Pacific Region	10	7	5	136	141
European Region	9	5	4	115	120
Eastern Mediterranean Region	6	4	3	92	95
**Nonavalent HPV vaccine**					
WHO region	Cases	Deaths	YLDs	YLLs	DALYs
	averted per 1000 vaccinated girls				
African Region	34	27	18	546	563
South-East Asia Region	19	14	9	285	295
Region of the Americas	16	10	8	215	223
Western Pacific Region	13	9	7	178	184
European Region	11	6	5	140	145
Eastern Mediterranean Region	7	6	4	115	119

A12. Vaccination impact per 1000 vaccinated 12-year-old girls at the regional level

Lifetime health impact of bivalent/quadrivalent and nonavalent HPV vaccination of 12-year-old girls during 2020-2029 on cases, deaths and DALYs averted per 1000 vaccinated girls in the 6 WHO regions (estimates after the combined PRIME updates for demography, disability weights and cervical cancer burden).

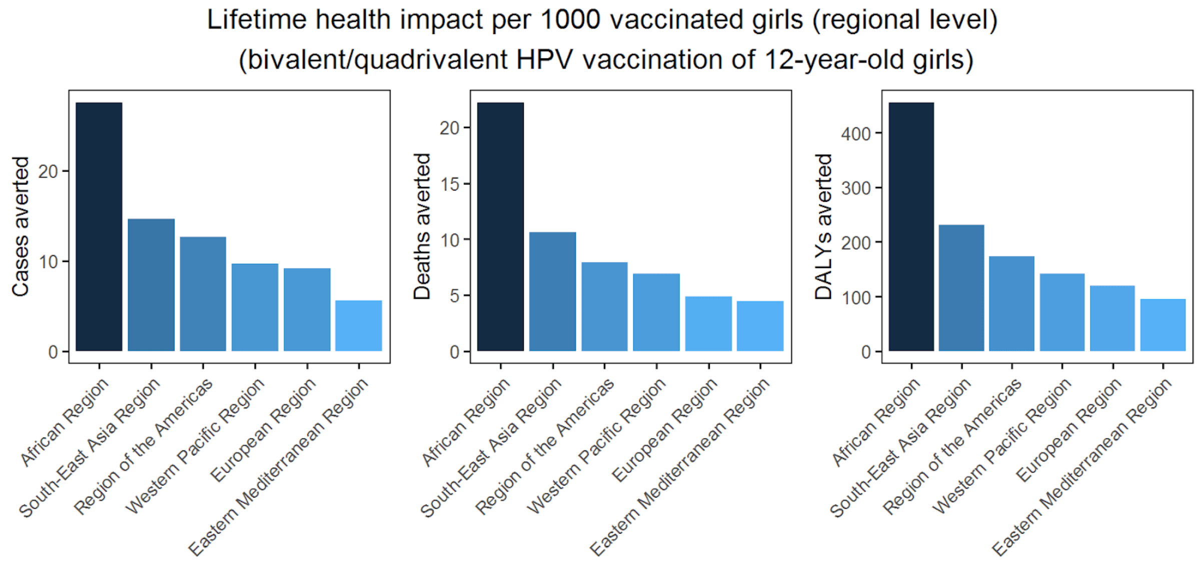

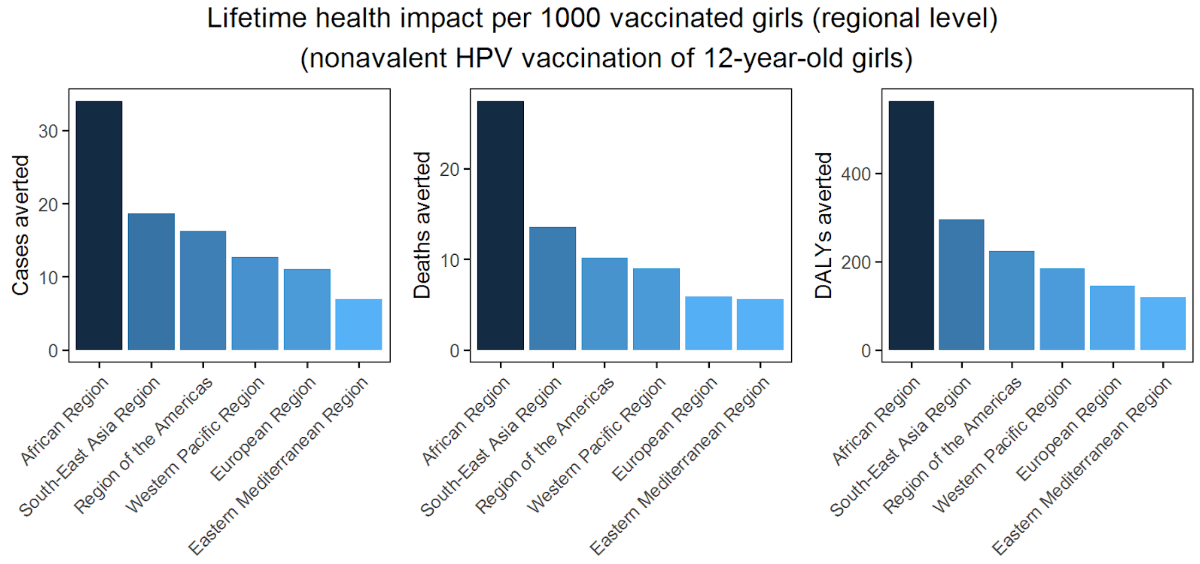

A13. Vaccination impact per 1000 vaccinated girls at the regional level

Lifetime health impact of HPV vaccination during 2020-2029 on cases, deaths, YLLs, YLDs and DALYs averted per 1000 vaccinated girls in the 6 WHO regions for bivalent/quadrivalent and nonavalent vaccination of 9-year-old and 12-year-old girls.

Lifetime health impact per 1000 vaccinated girls − African Region
(vaccination age = 9 years / bivalent/quadrivalent vaccine)

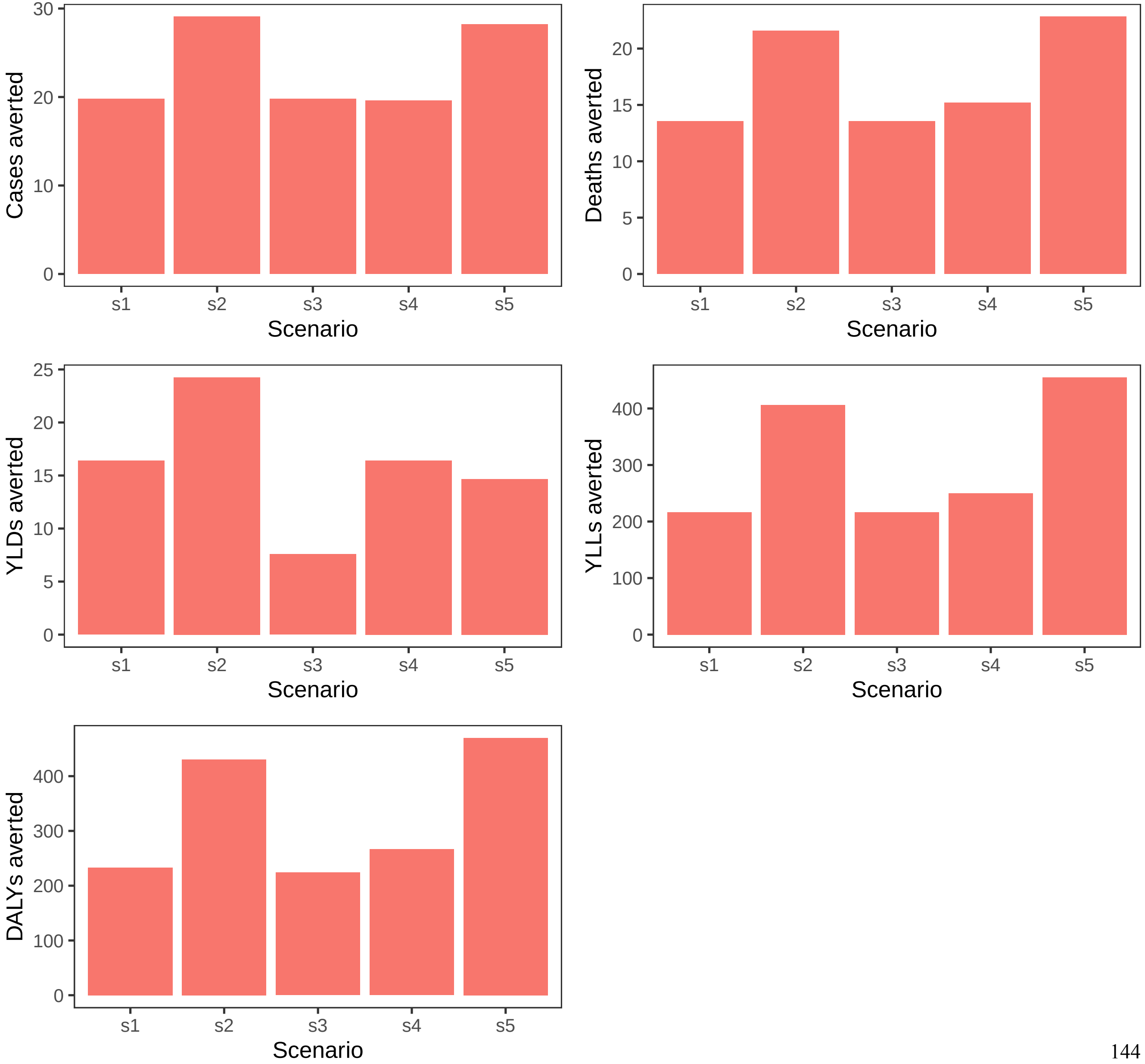

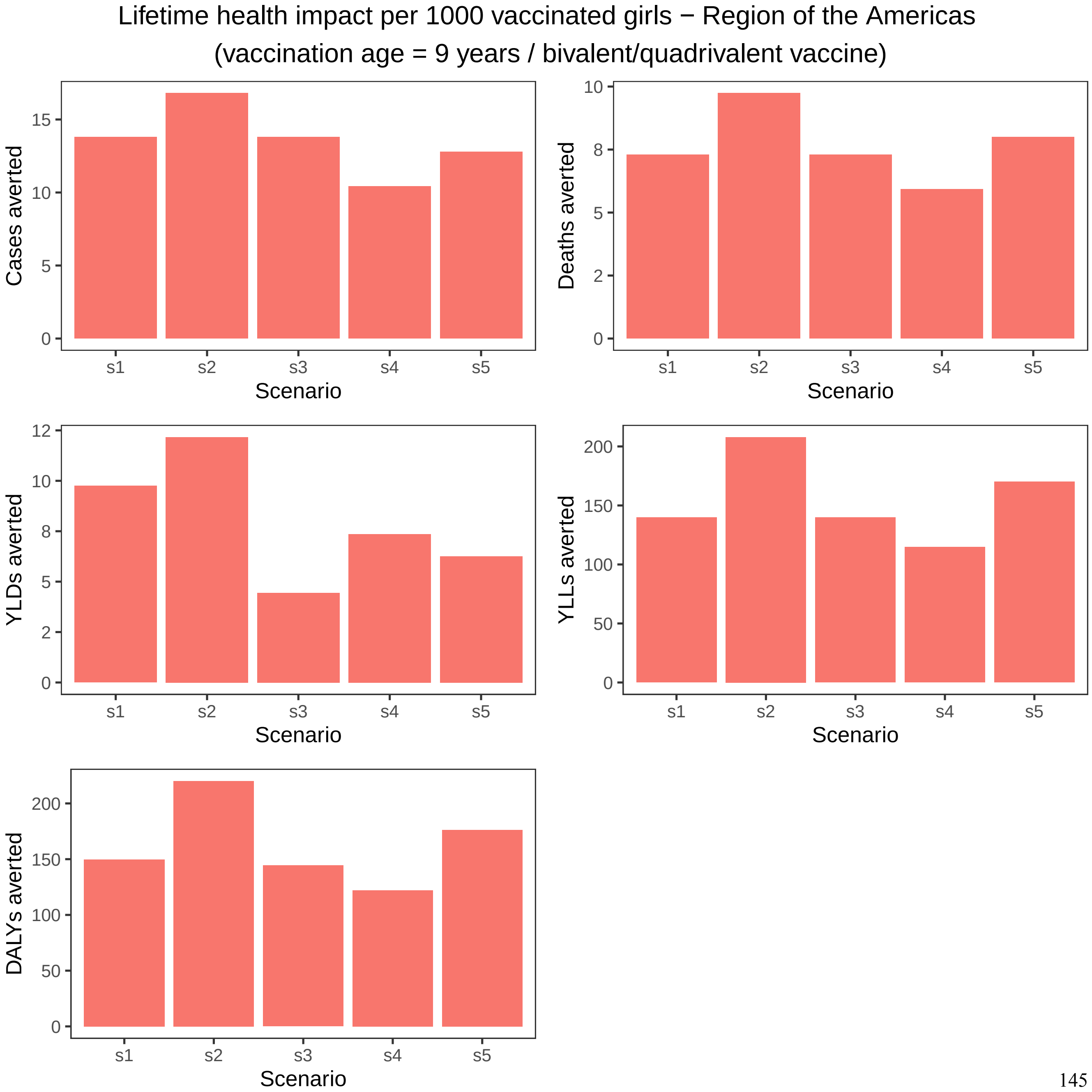
Lifetime health impact per 1000 vaccinated girls − Region of the Americas
(vaccination age = 9 years / bivalent/quadrivalent vaccine)
Cases averted
Deaths averted
YLDs averted
YLLs averted
DALYs averted
Scenario
s1
s2
s3
s4
s5

Lifetime health impact per 1000 vaccinated girls − Eastern Mediterranean Region
(vaccination age = 9 years / bivalent/quadrivalent vaccine)

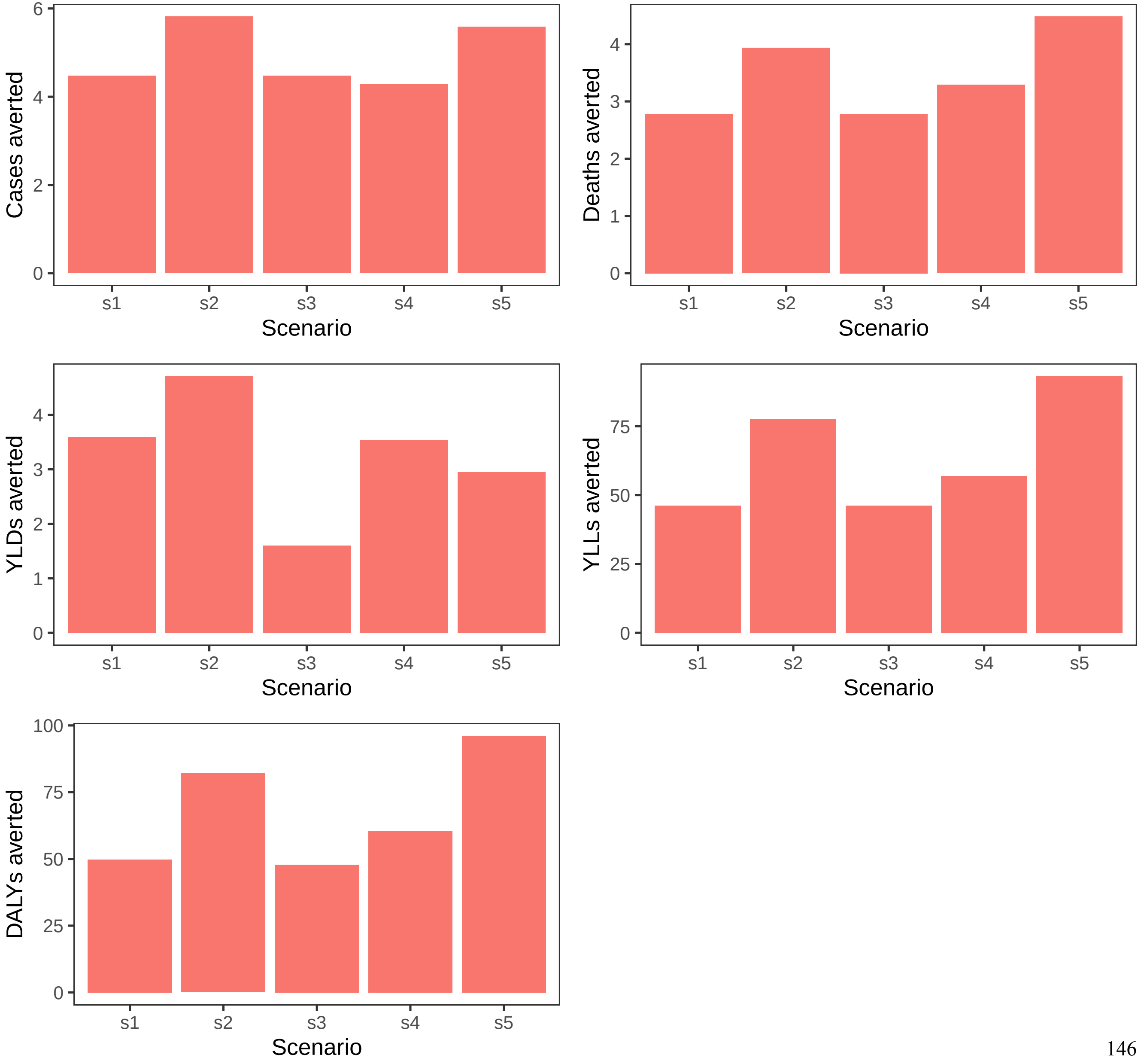

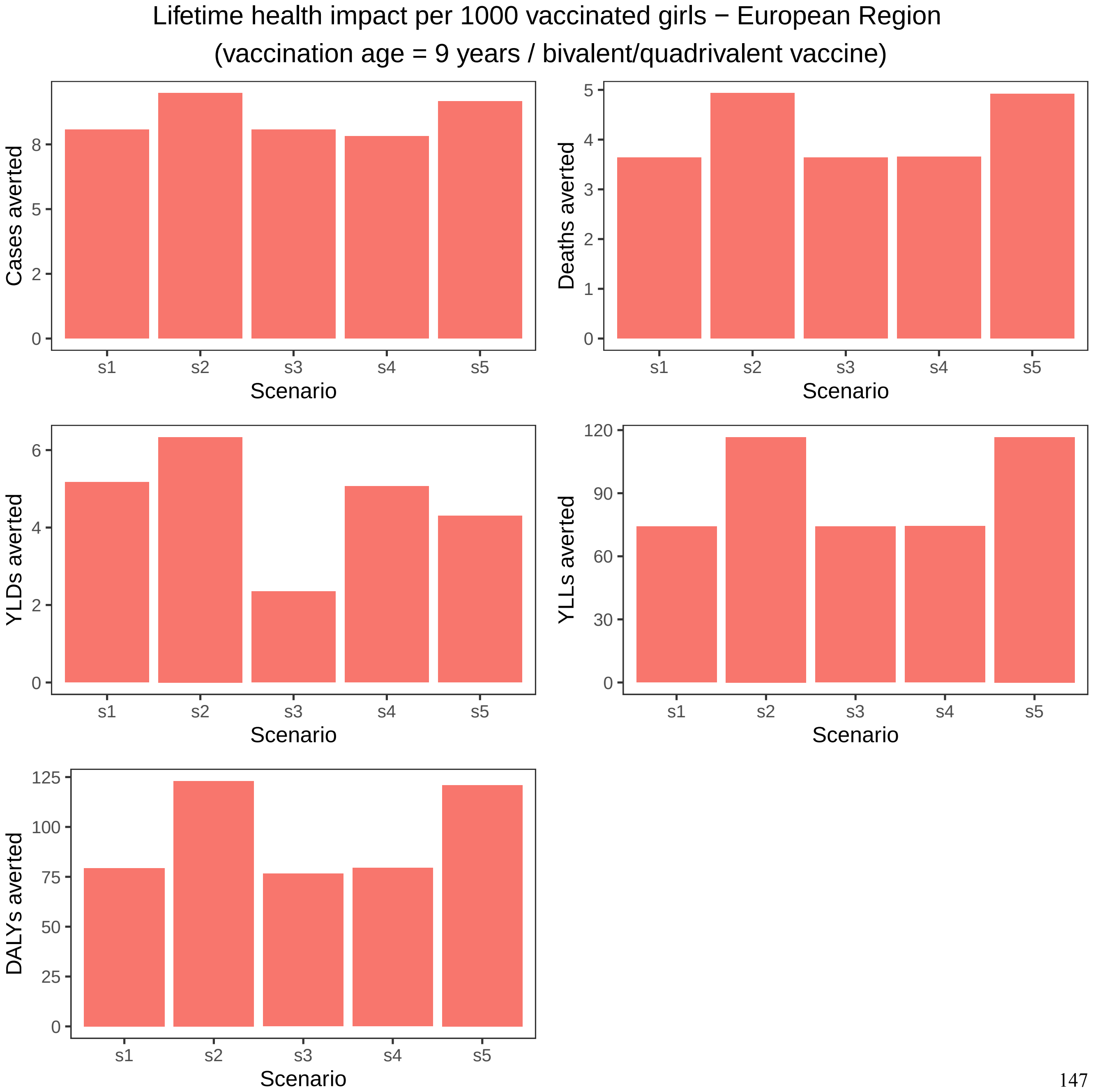
Lifetime health impact per 1000 vaccinated girls − European Region
(vaccination age = 9 years / bivalent/quadrivalent vaccine)
Cases averted
Deaths averted
YLDs averted
YLLs averted
DALYs averted
Scenario
s1
s2
s3
s4
s5

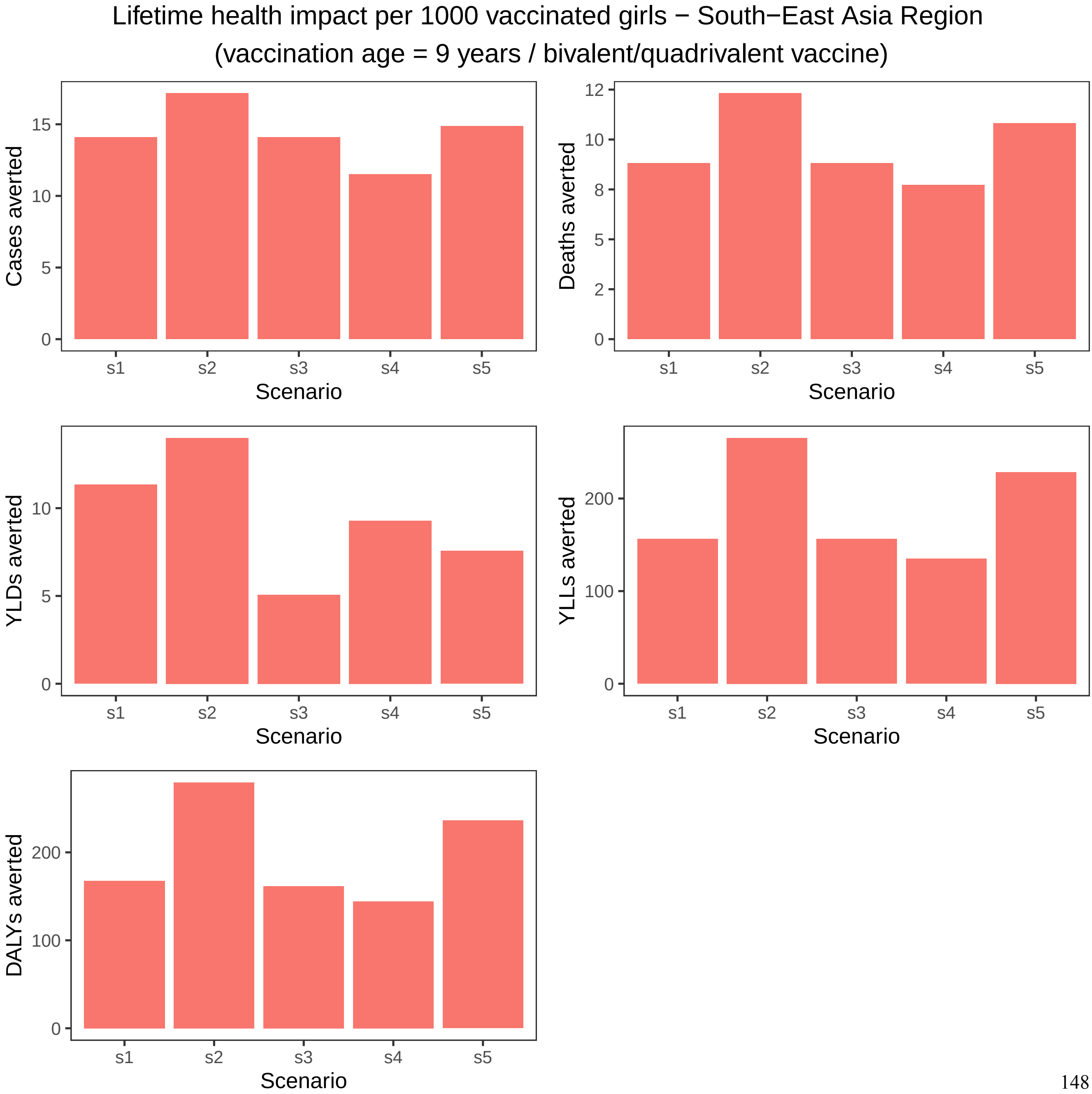
Lifetime health impact per 1000 vaccinated girls − South−East Asia Region
(vaccination age = 9 years / bivalent/quadrivalent vaccine)
Cases averted
0
5
10
15
Deaths averted
0
2
5
8
10
12
YLDs averted
0
5
10
YLLs averted
0
100
200
DALYs averted
0
100
200
s1
s2
s3
s4
s5
Scenario

Lifetime health impact per 1000 vaccinated girls − Western Pacific Region
(vaccination age = 9 years / bivalent/quadrivalent vaccine)

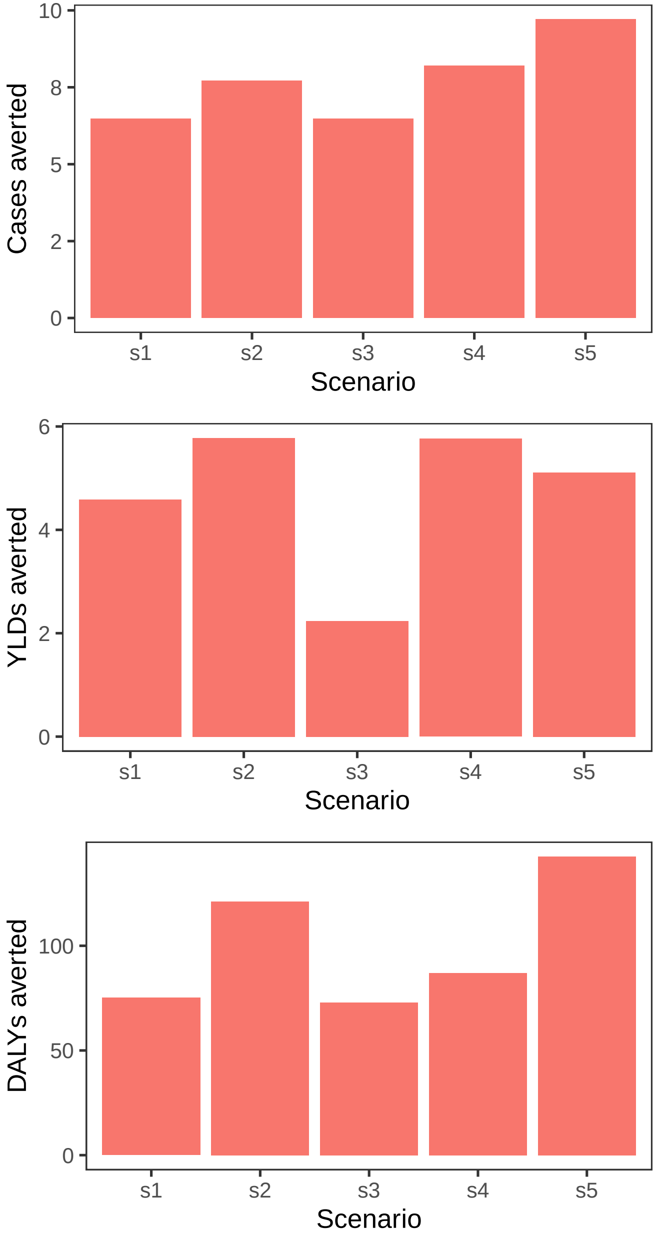

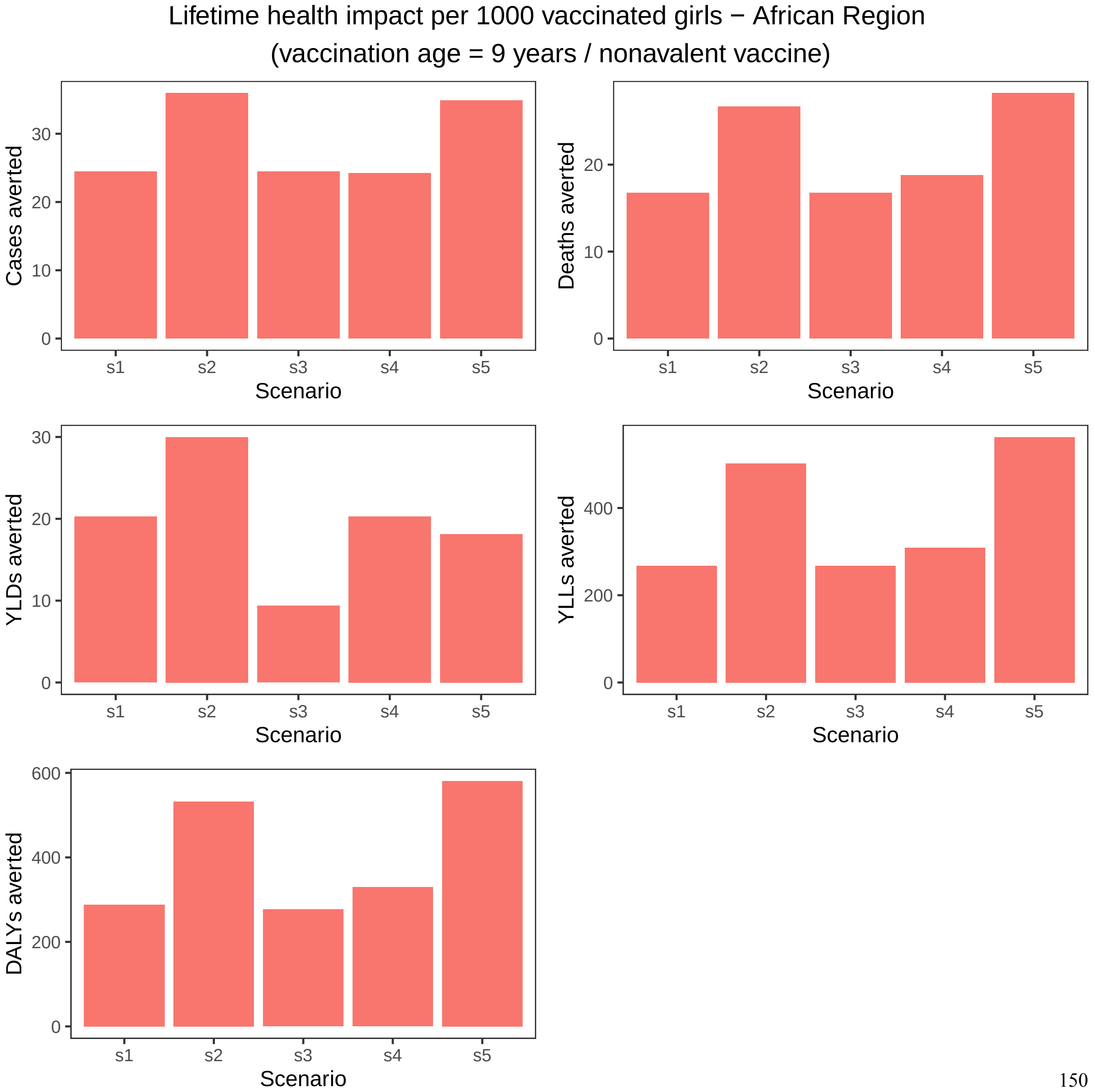

Lifetime health impact per 1000 vaccinated girls − African Region
(vaccination age = 9 years / nonavalent vaccine)
Cases averted
Deaths averted
YLDs averted
YLLs averted
DALYs averted
Scenario
s1
s2
s3
s4
s5
0
10
20
30
200
400
600

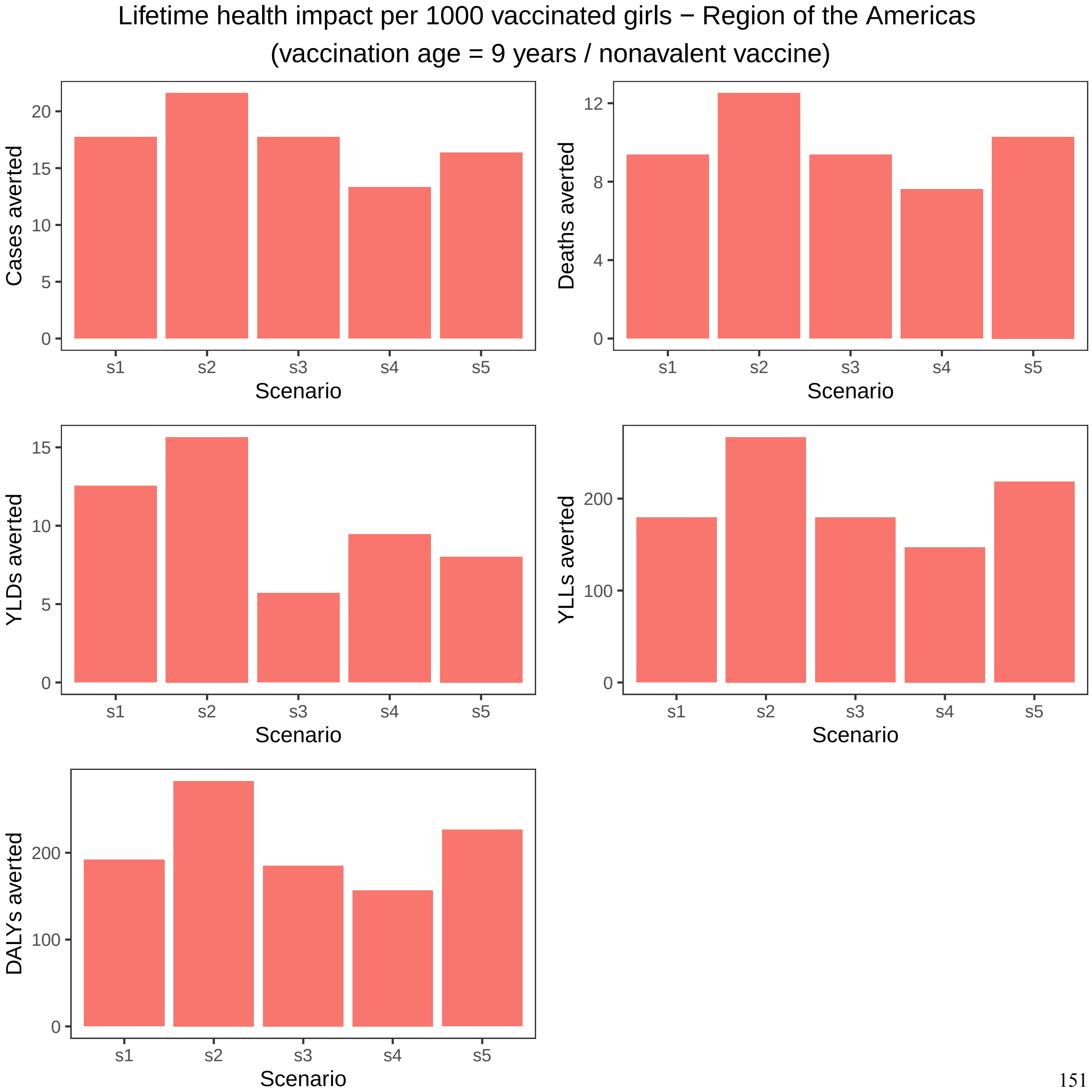
Lifetime health impact per 1000 vaccinated girls − Region of the Americas
(vaccination age = 9 years / nonavalent vaccine)
Cases averted
Deaths averted
YLDs averted
YLLs averted
DALYs averted
Scenario
s1
s2
s3
s4
s5
0
5
10
15
20
4
8
12
100
200

Lifetime health impact per 1000 vaccinated girls − Eastern Mediterranean Region
(vaccination age = 9 years / nonavalent vaccine)

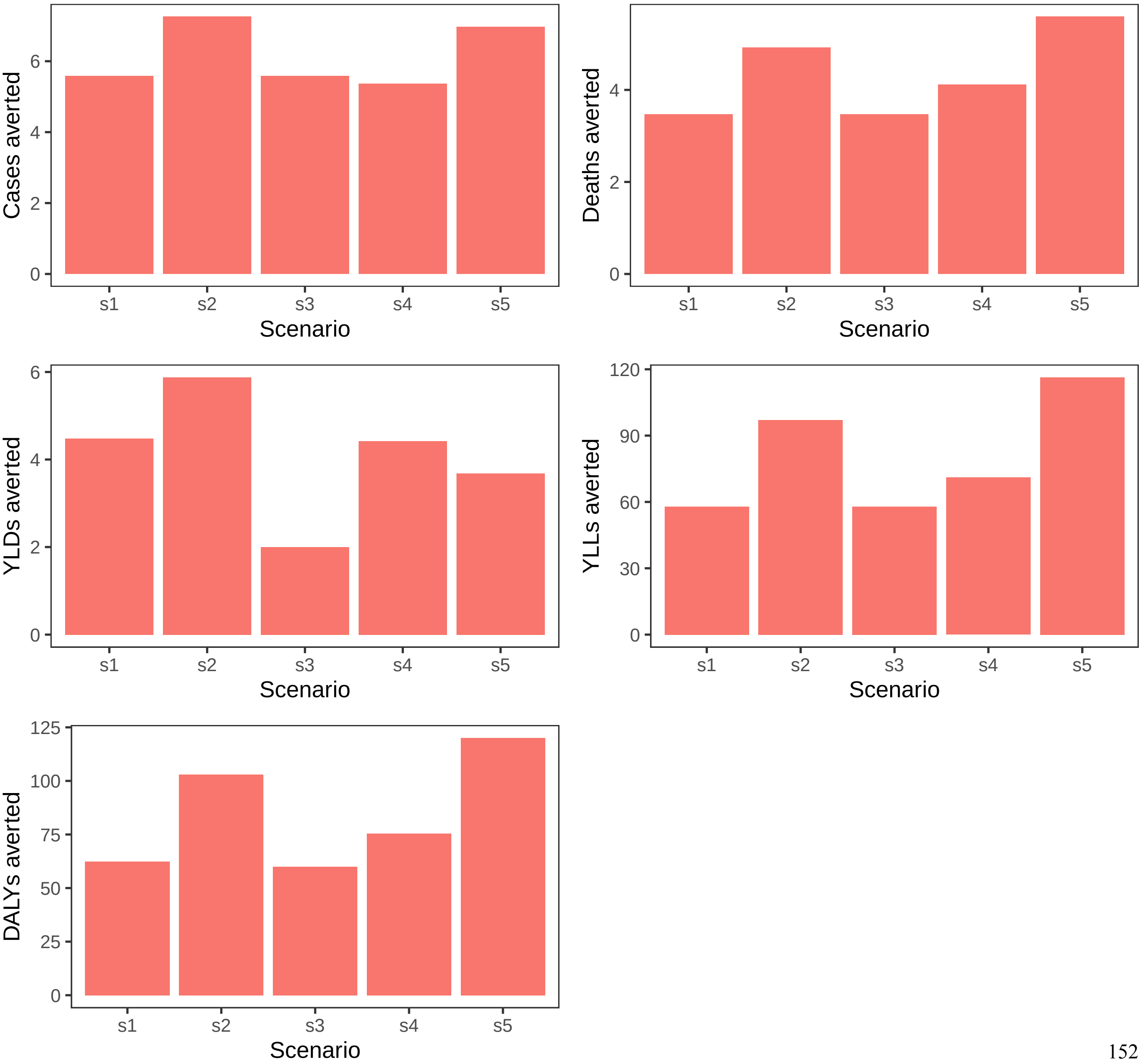

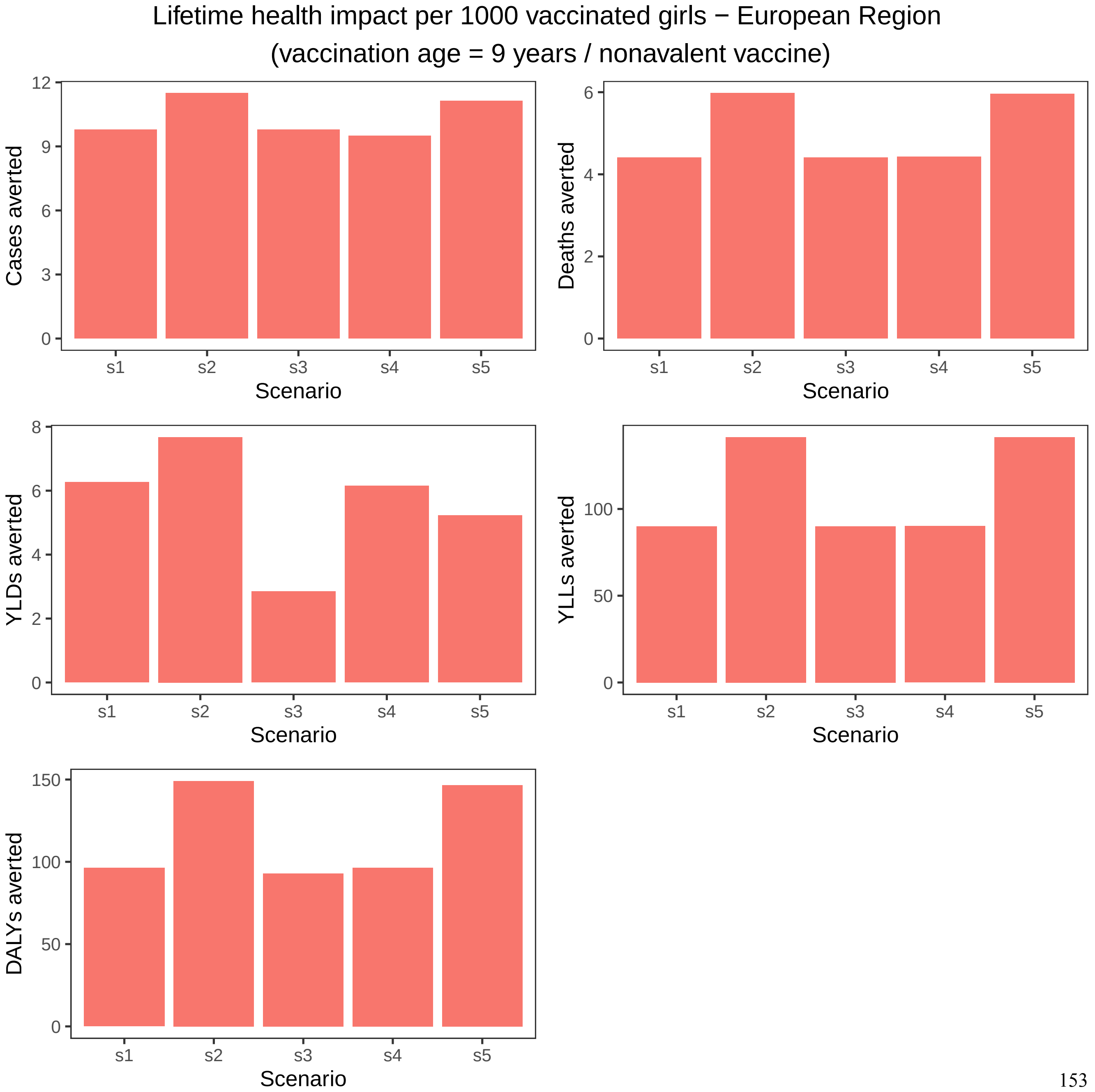

Lifetime health impact per 1000 vaccinated girls − European Region
(vaccination age = 9 years / nonavalent vaccine)
Cases averted
Deaths averted
YLDs averted
YLLs averted
DALYs averted
Scenario
s1
s2
s3
s4
s5

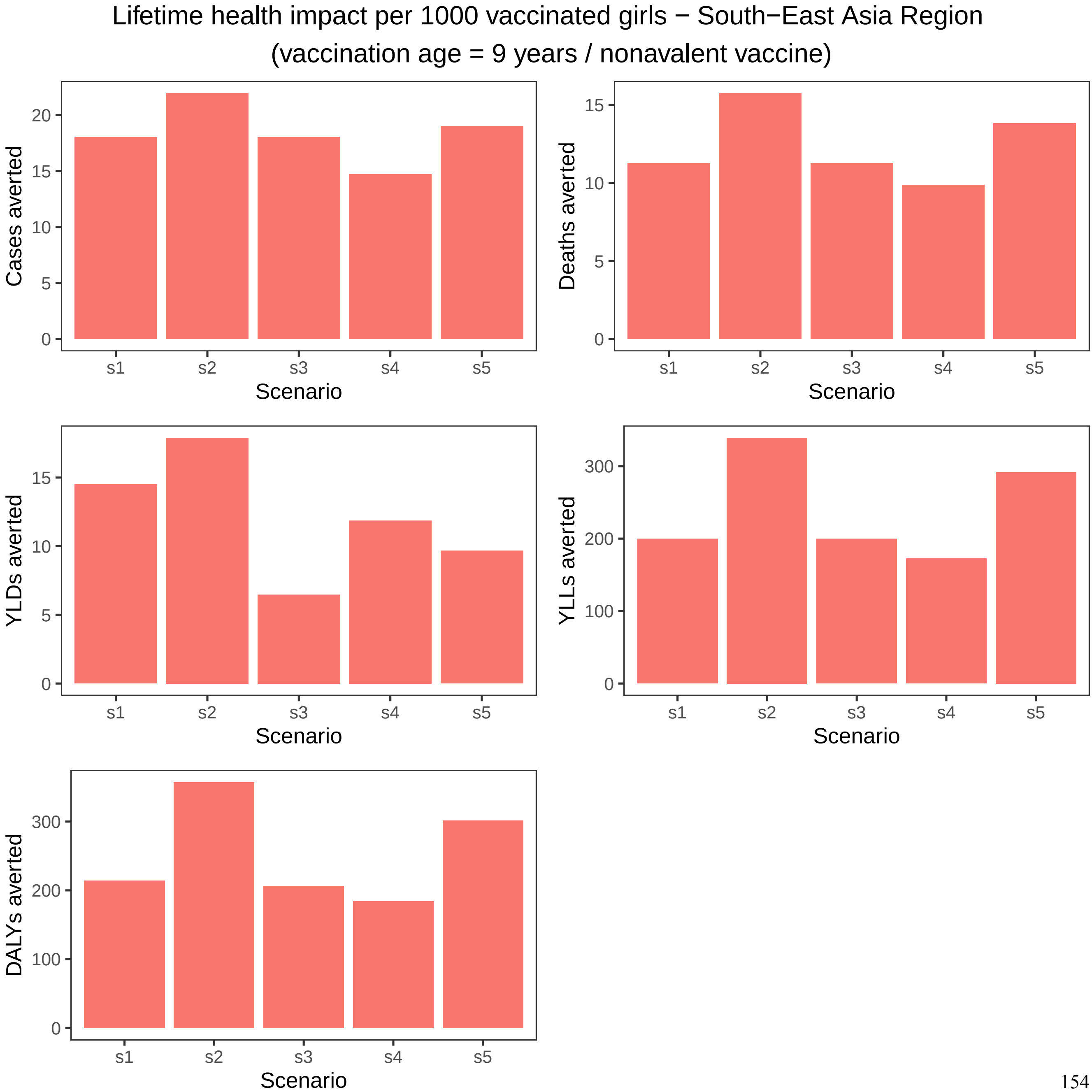

Lifetime health impact per 1000 vaccinated girls − South−East Asia Region
(vaccination age = 9 years / nonavalent vaccine)
Cases averted
Deaths averted
YLDs averted
YLLs averted
DALYs averted
Scenario
s1
s2
s3
s4
s5

Lifetime health impact per 1000 vaccinated girls − Western Pacific Region
(vaccination age = 9 years / nonavalent vaccine)

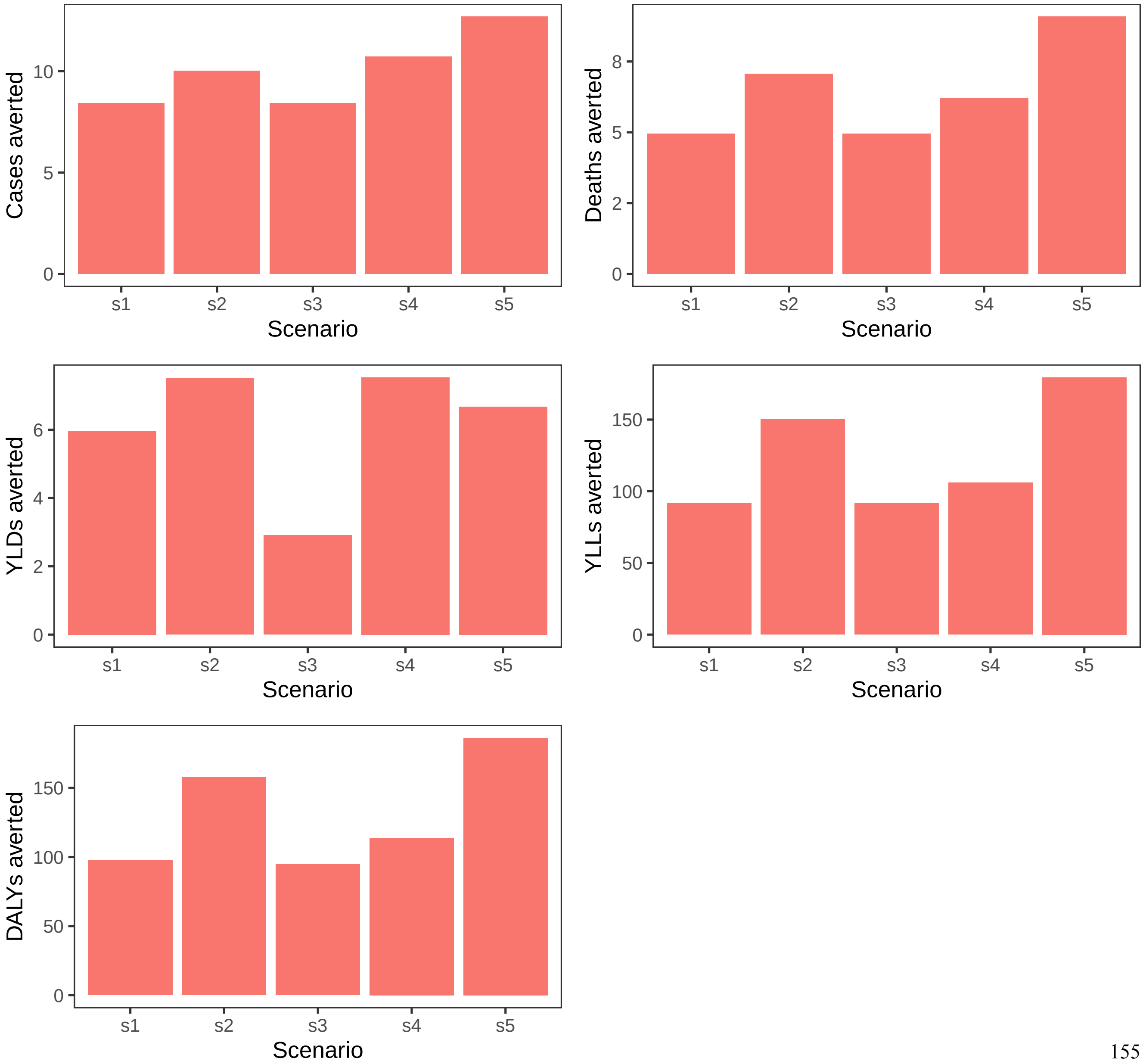

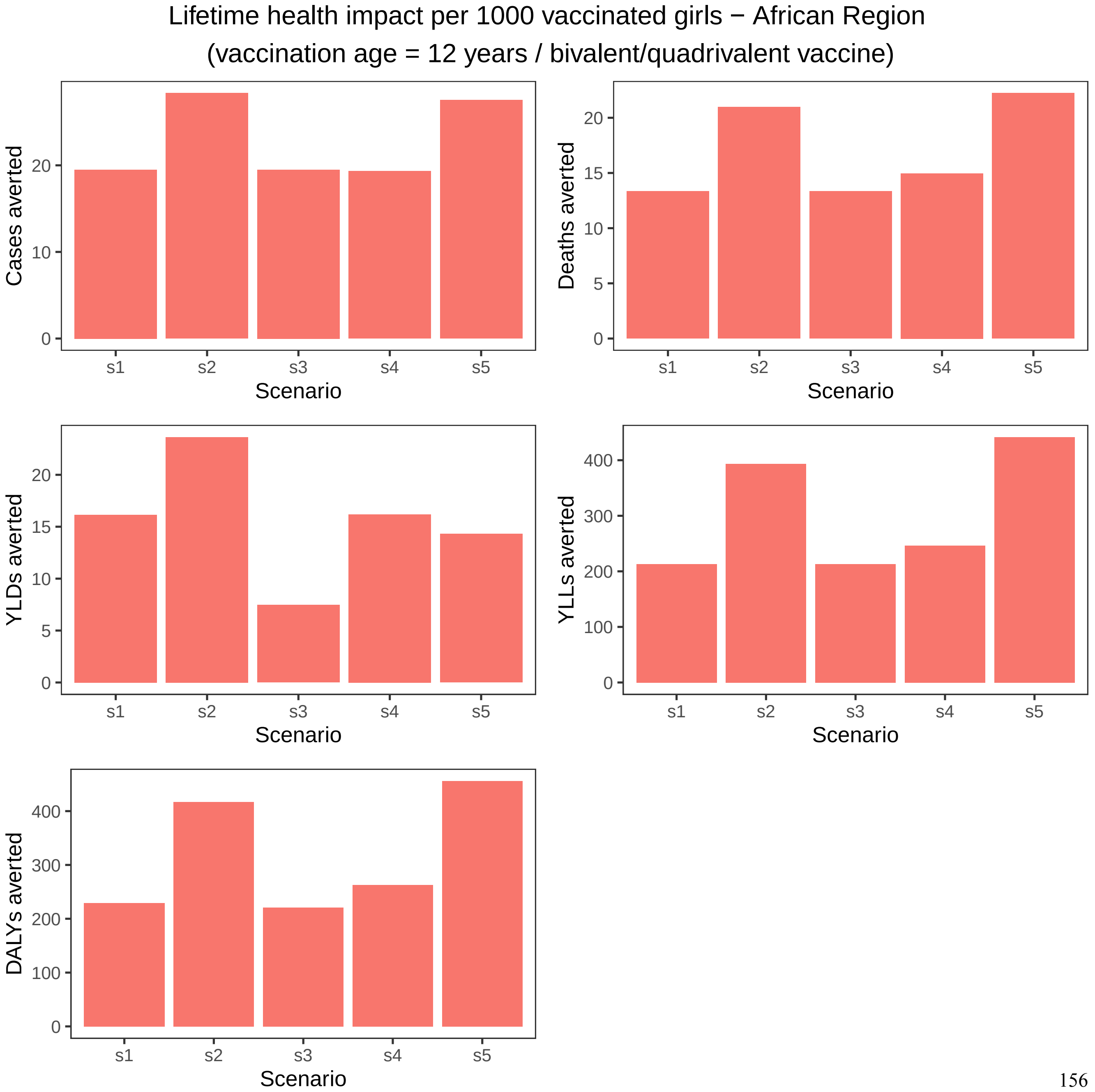

Lifetime health impact per 1000 vaccinated girls − African Region
(vaccination age = 12 years / bivalent/quadrivalent vaccine)
Cases averted
0
10
20
s1
s2
s3
s4
s5
Scenario
Deaths averted
0
5
10
15
20
s1
s2
s3
s4
s5
Scenario
YLDs averted
0
5
10
15
20
s1
s2
s3
s4
s5
Scenario
YLLs averted
0
100
200
300
400
s1
s2
s3
s4
s5
Scenario
DALYs averted
0
100
200
300
400
s1
s2
s3
s4
s5
Scenario

Lifetime health impact per 1000 vaccinated girls − Region of the Americas
(vaccination age = 12 years / bivalent/quadrivalent vaccine)

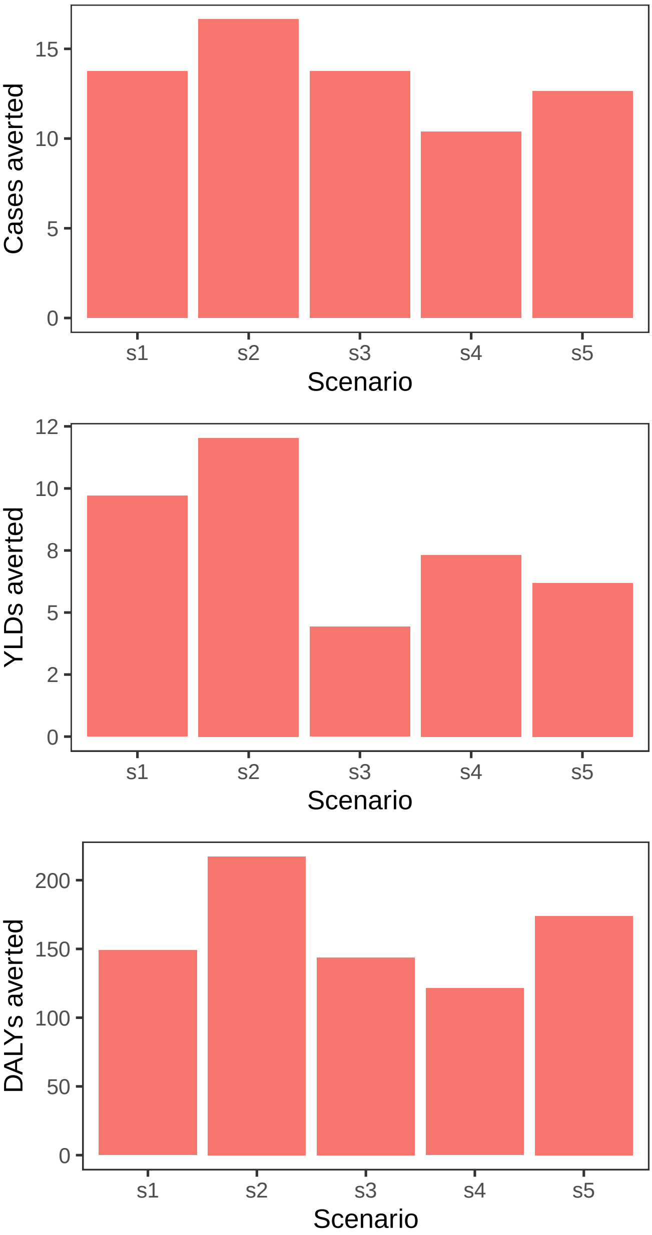

Lifetime health impact per 1000 vaccinated girls − Eastern Mediterranean Region
(vaccination age = 12 years / bivalent/quadrivalent vaccine)

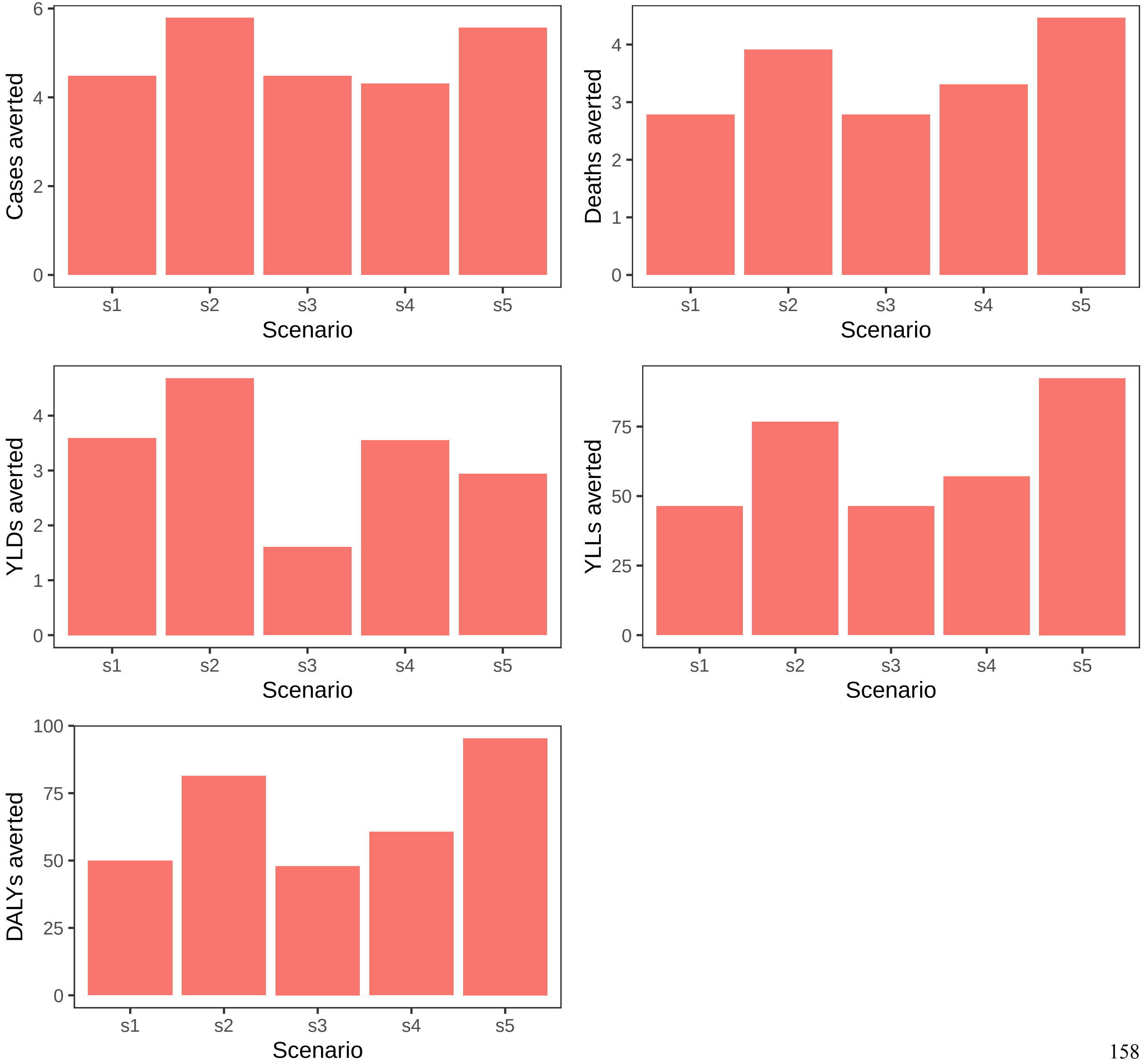

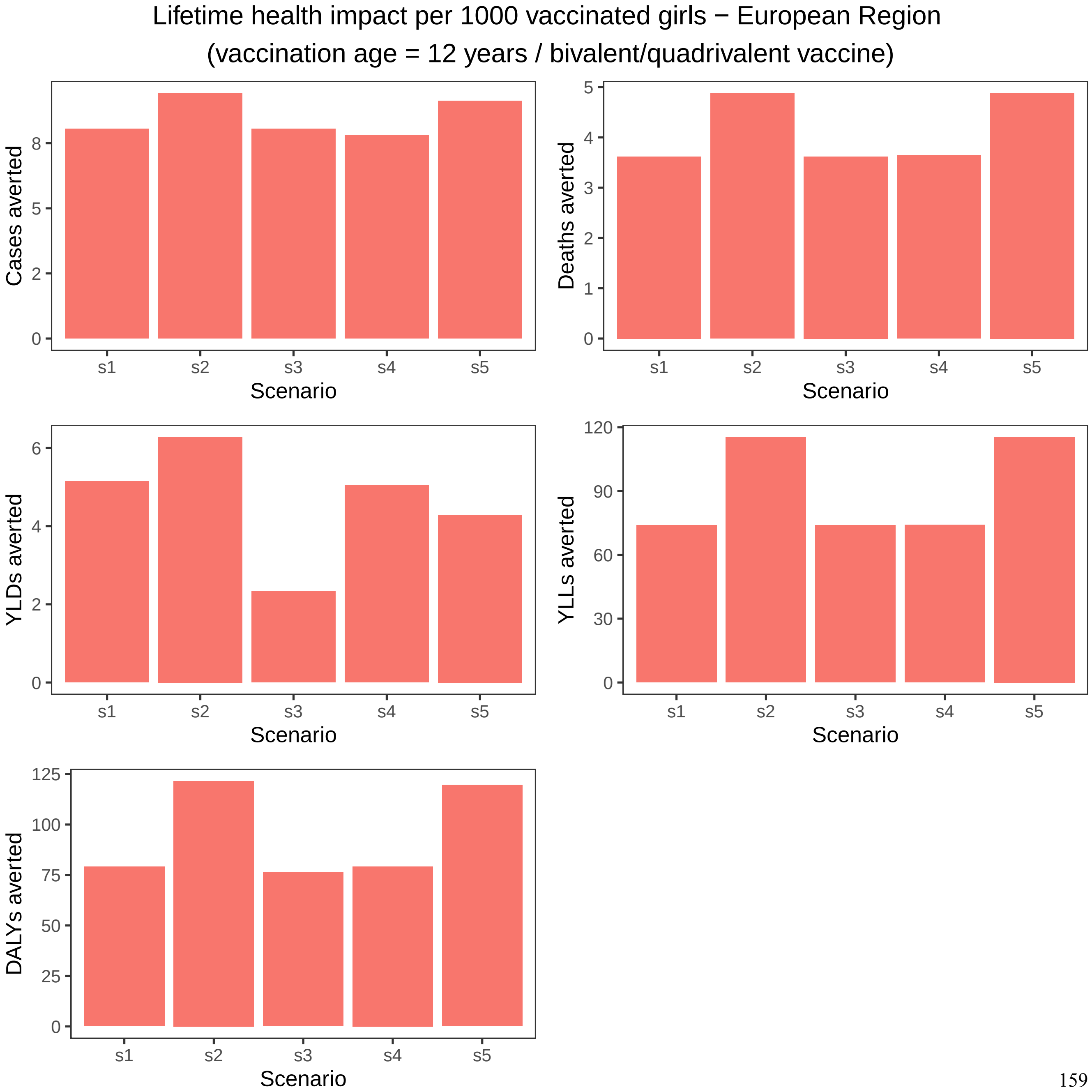

Lifetime health impact per 1000 vaccinated girls − European Region
(vaccination age = 12 years / bivalent/quadrivalent vaccine)
Cases averted
Deaths averted
YLDs averted
YLLs averted
DALYs averted
Scenario
s1
s2
s3
s4
s5

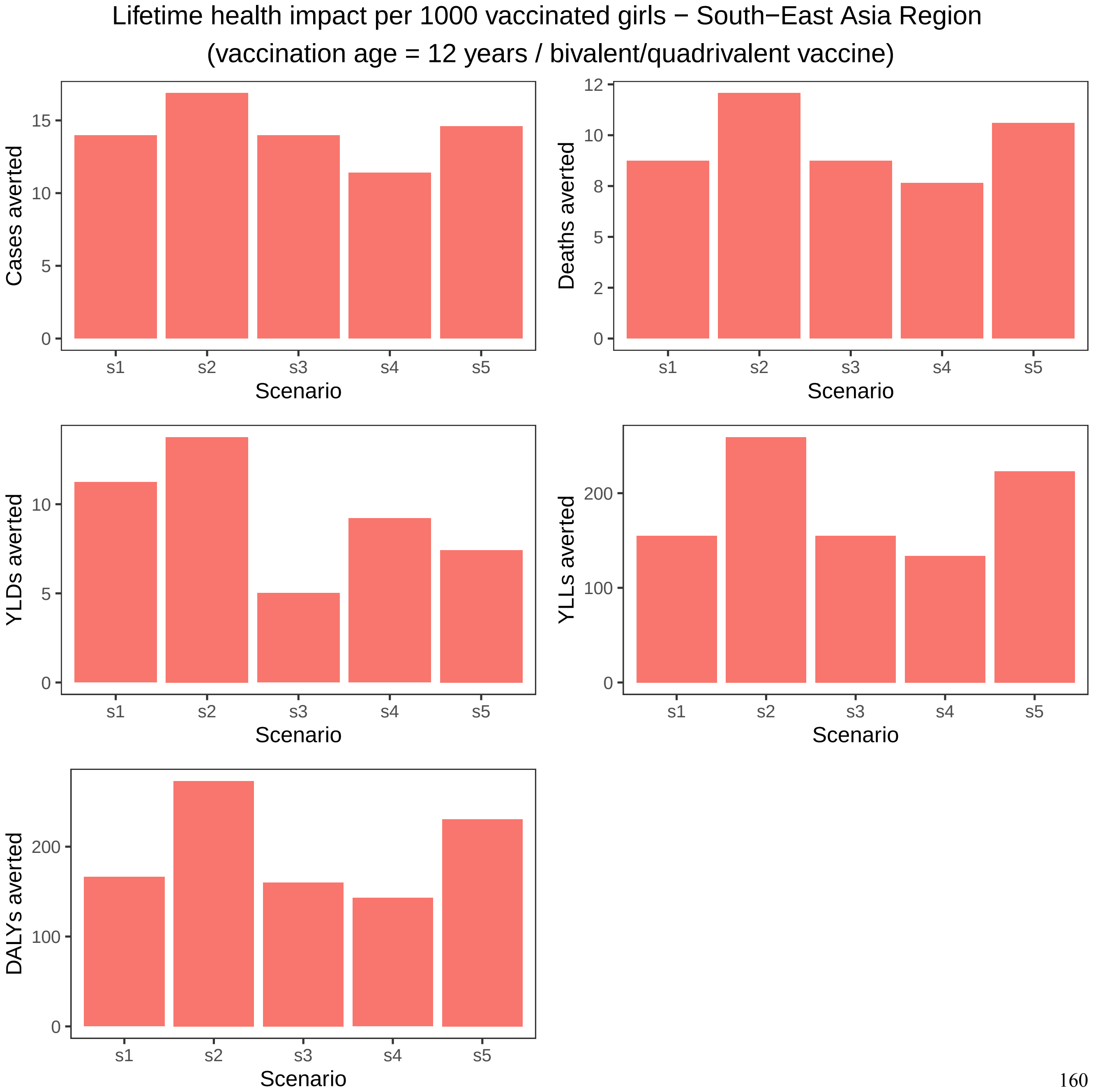
Lifetime health impact per 1000 vaccinated girls − South−East Asia Region
(vaccination age = 12 years / bivalent/quadrivalent vaccine)
Cases averted
Deaths averted
YLDs averted
YLLs averted
DALYs averted
Scenario
s1
s2
s3
s4
s5
0
5
10
15
2
8
12
100
200

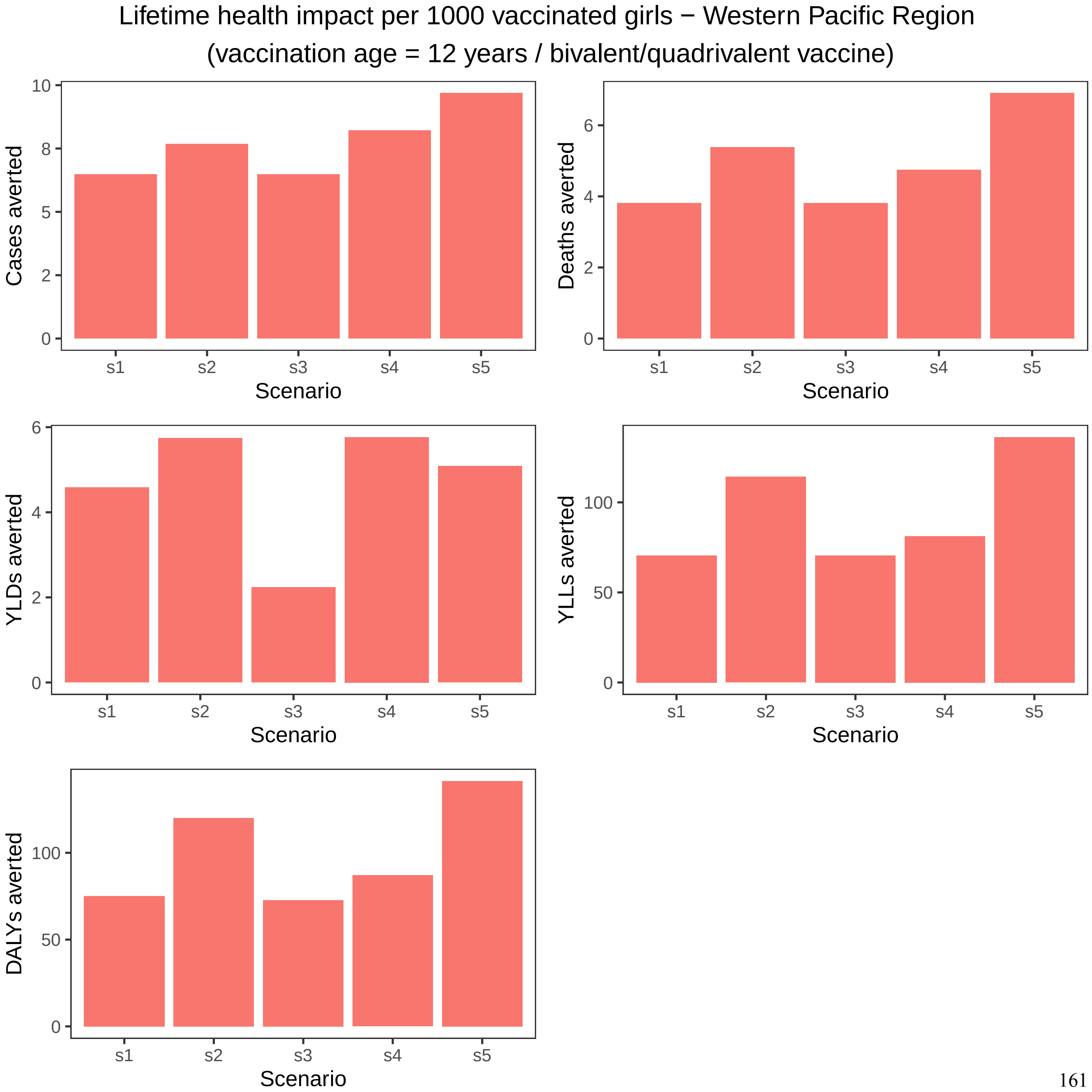

Lifetime health impact per 1000 vaccinated girls − Western Pacific Region
(vaccination age = 12 years / bivalent/quadrivalent vaccine)
Cases averted
Deaths averted
YLDs averted
YLLs averted
DALYs averted
Scenario
s1
s2
s3
s4
s5
0
2
4
5
6
8
10
50
100

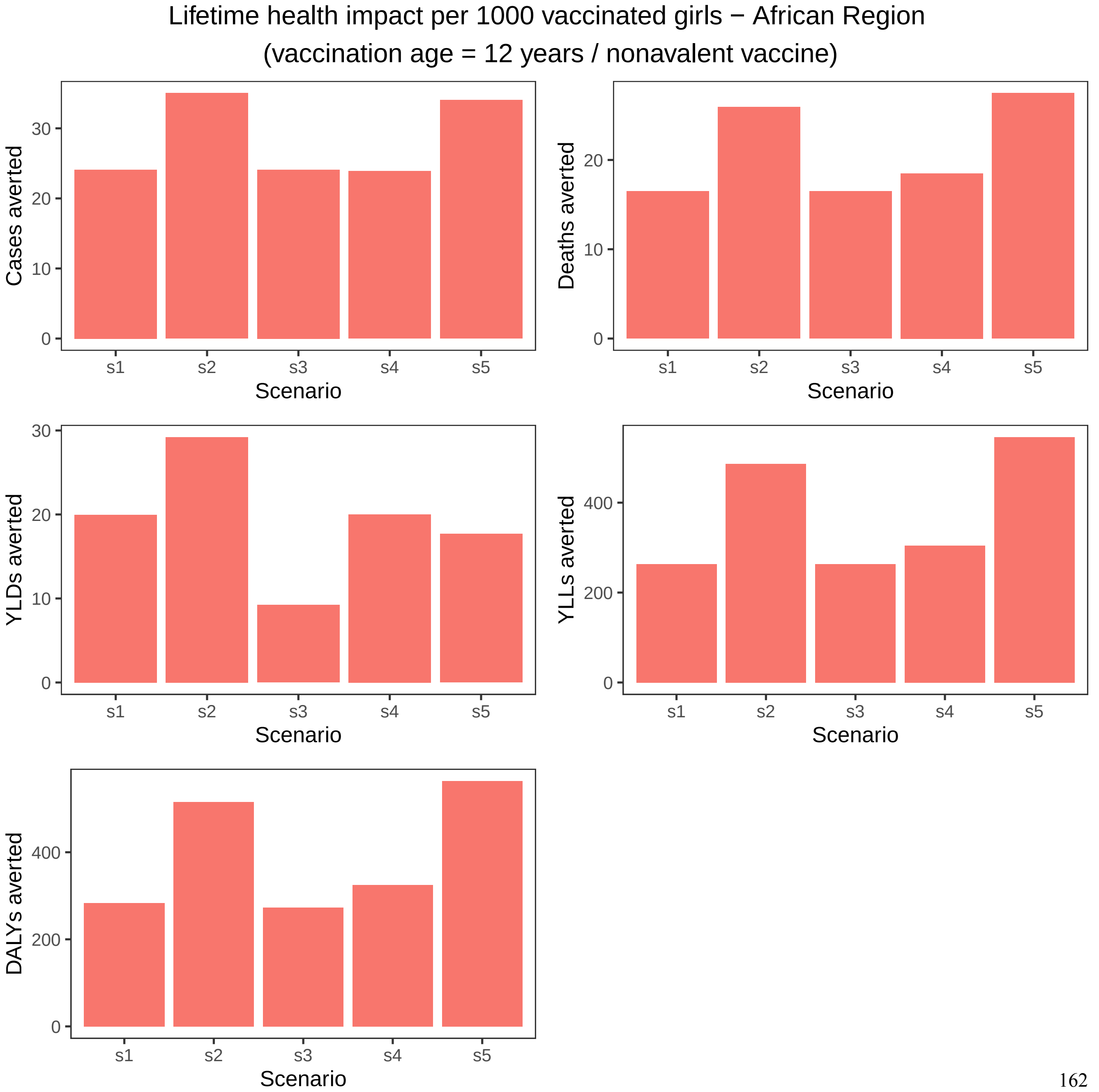
Lifetime health impact per 1000 vaccinated girls − African Region
(vaccination age = 12 years / nonavalent vaccine)
Cases averted
Deaths averted
YLDs averted
YLLs averted
DALYs averted
Scenario
s1
s2
s3
s4
s5
0
10
20
30
200
400

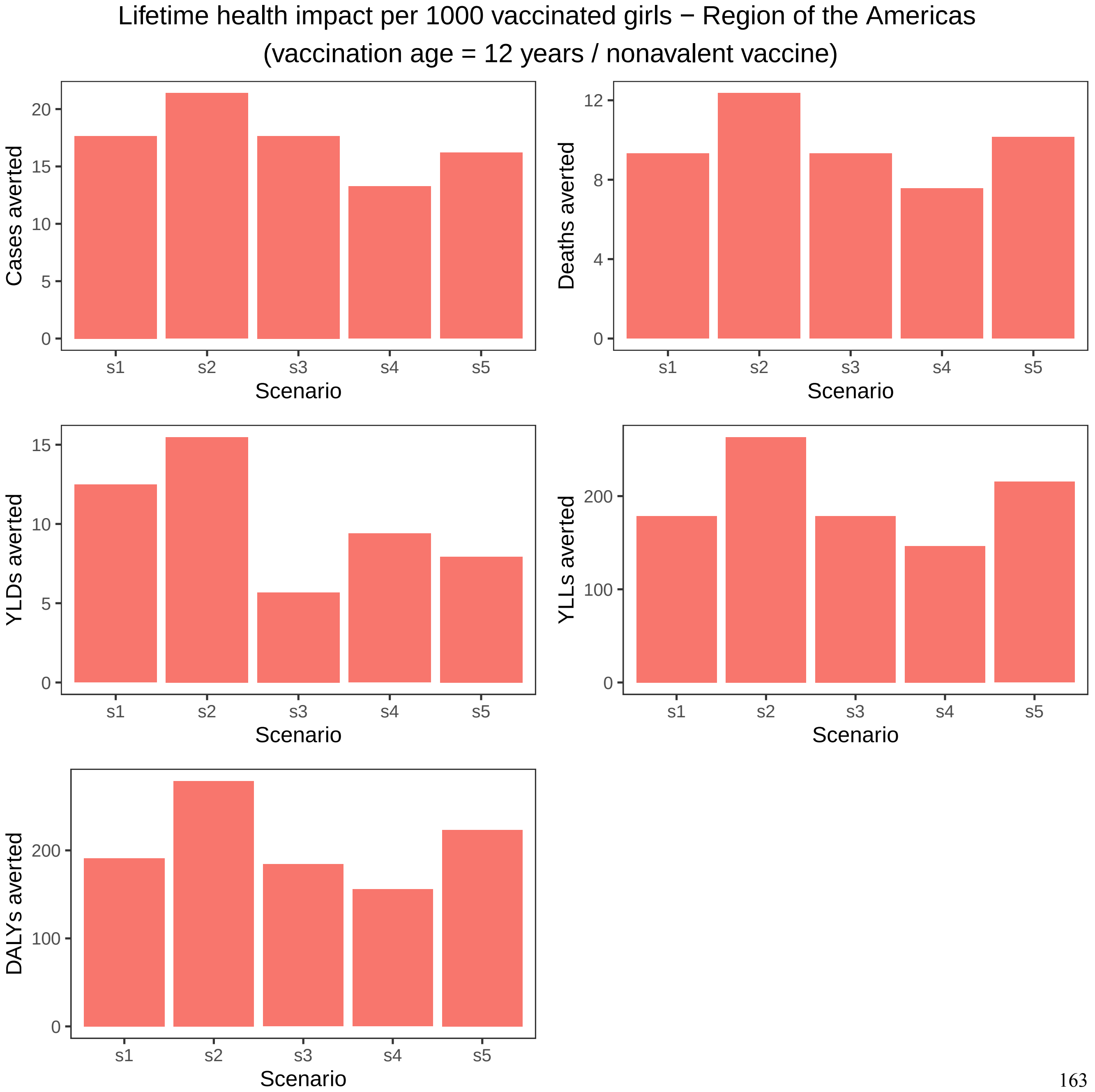
Lifetime health impact per 1000 vaccinated girls − Region of the Americas
(vaccination age = 12 years / nonavalent vaccine)
Cases averted
0
5
10
15
20
Deaths averted
0
4
8
12
YLDs averted
0
5
10
15
YLLs averted
0
100
200
DALYs averted
0
100
200
s1
s2
s3
s4
s5
Scenario

Lifetime health impact per 1000 vaccinated girls − Eastern Mediterranean Region
(vaccination age = 12 years / nonavalent vaccine)

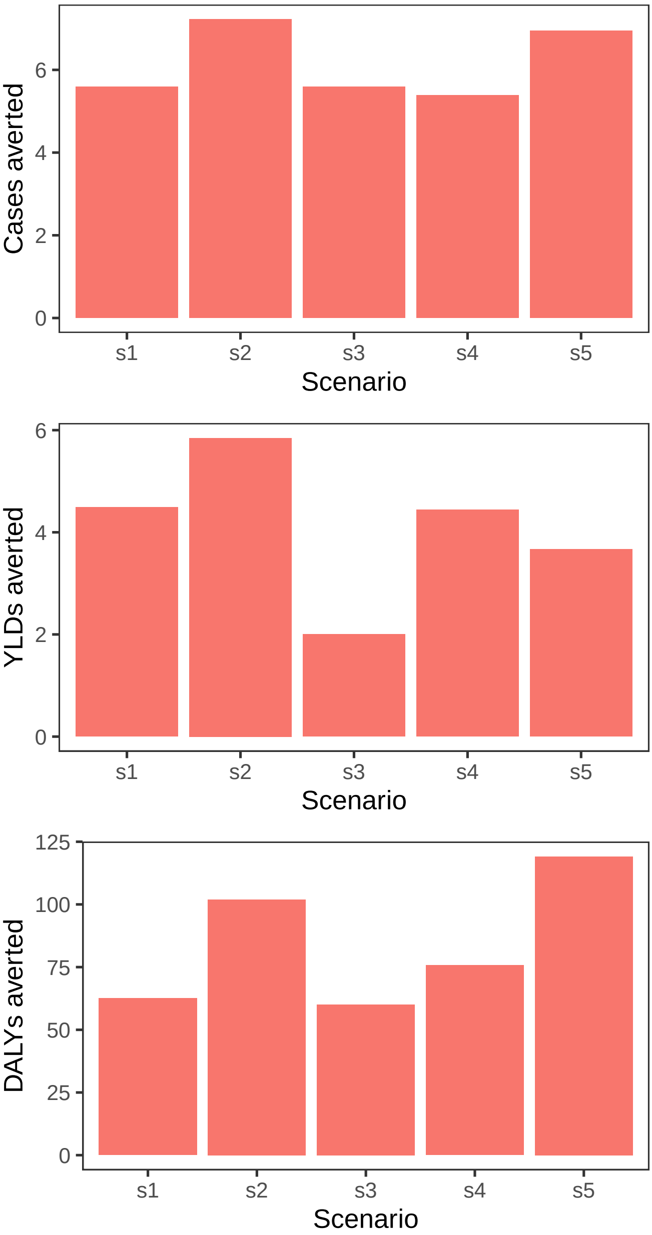

Lifetime health impact per 1000 vaccinated girls − European Region
(vaccination age = 12 years / nonavalent vaccine)

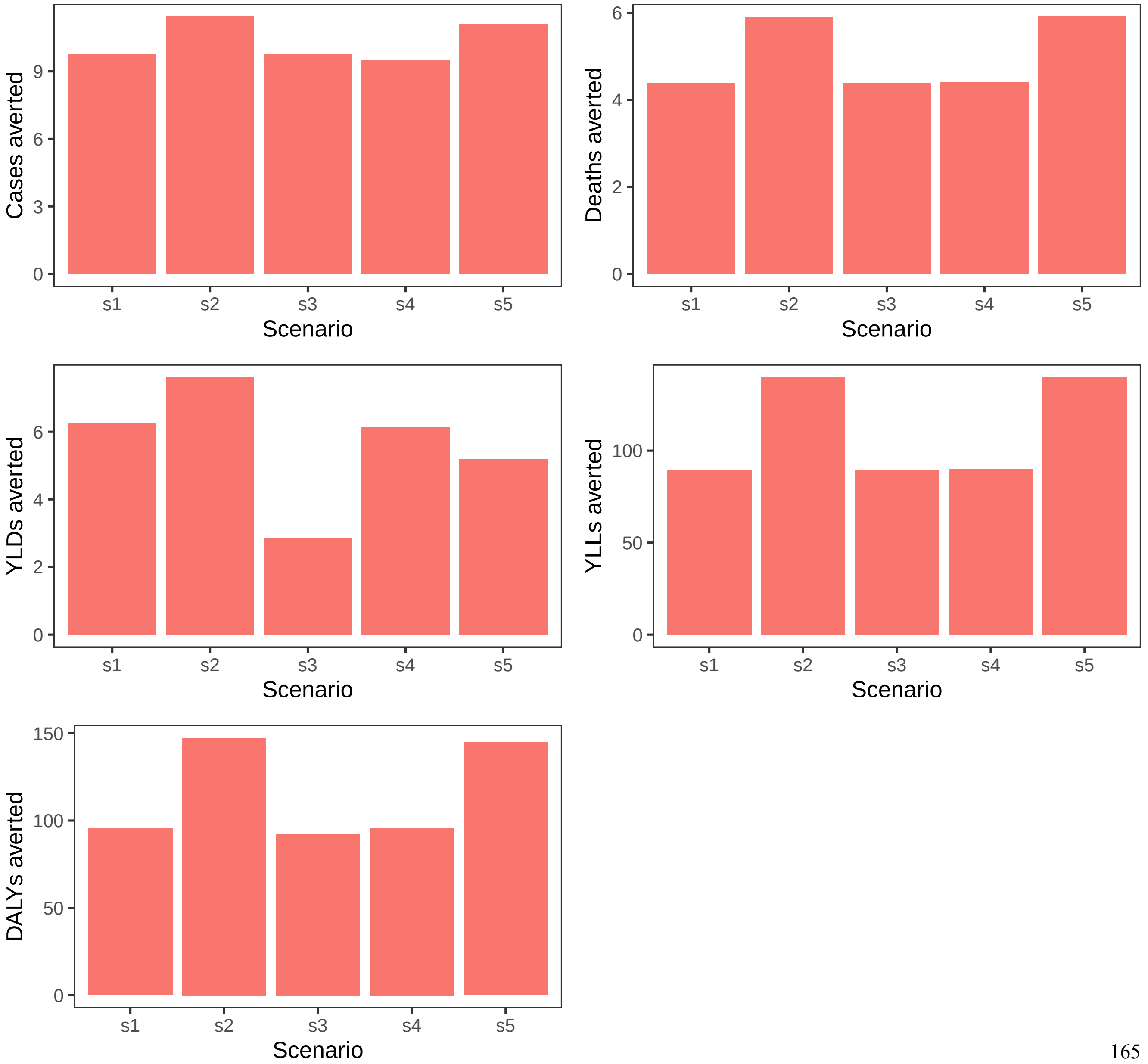

Lifetime health impact per 1000 vaccinated girls − South−East Asia Region
(vaccination age = 12 years / nonavalent vaccine)

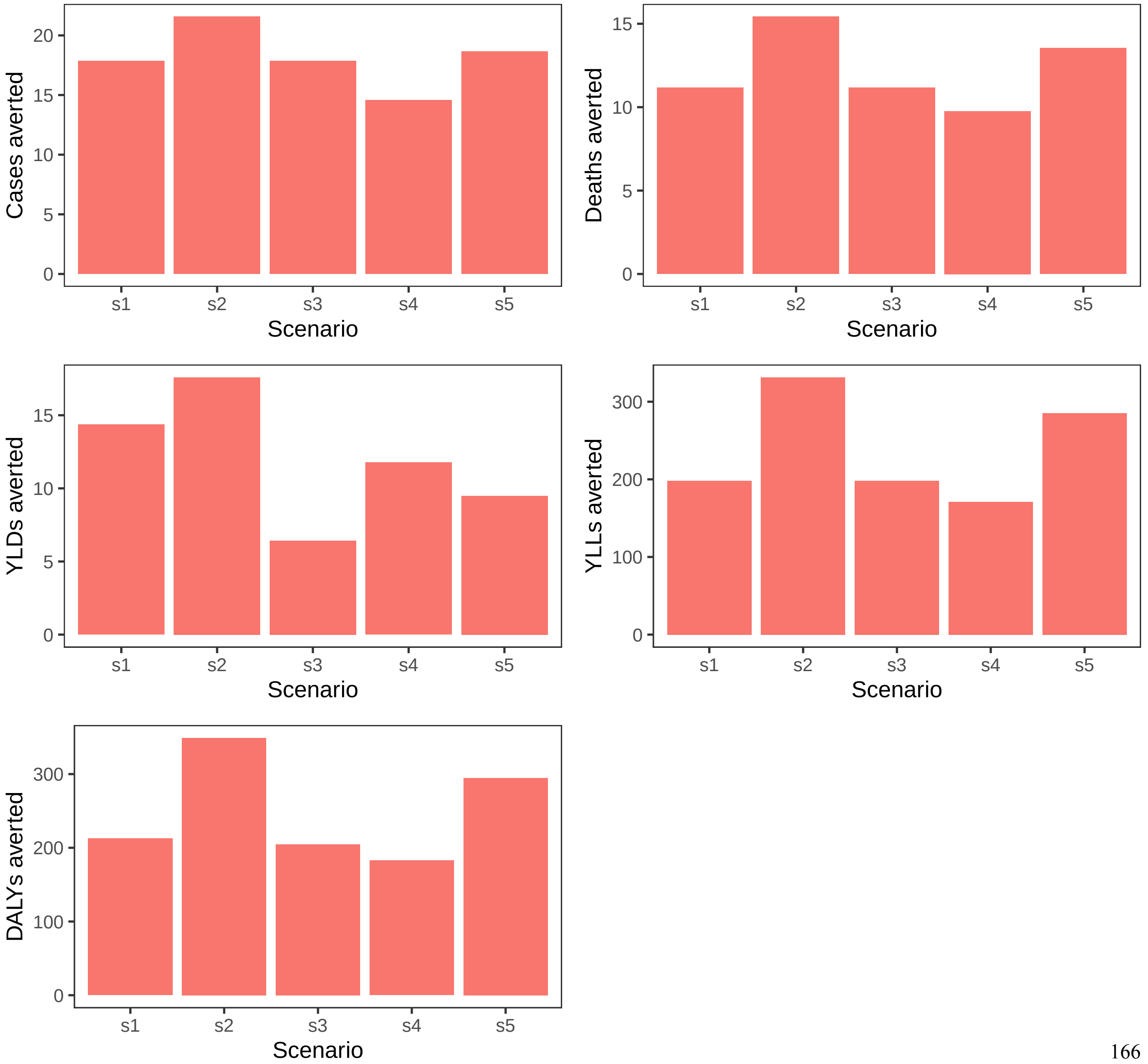

Lifetime health impact per 1000 vaccinated girls − Western Pacific Region
(vaccination age = 12 years / nonavalent vaccine)

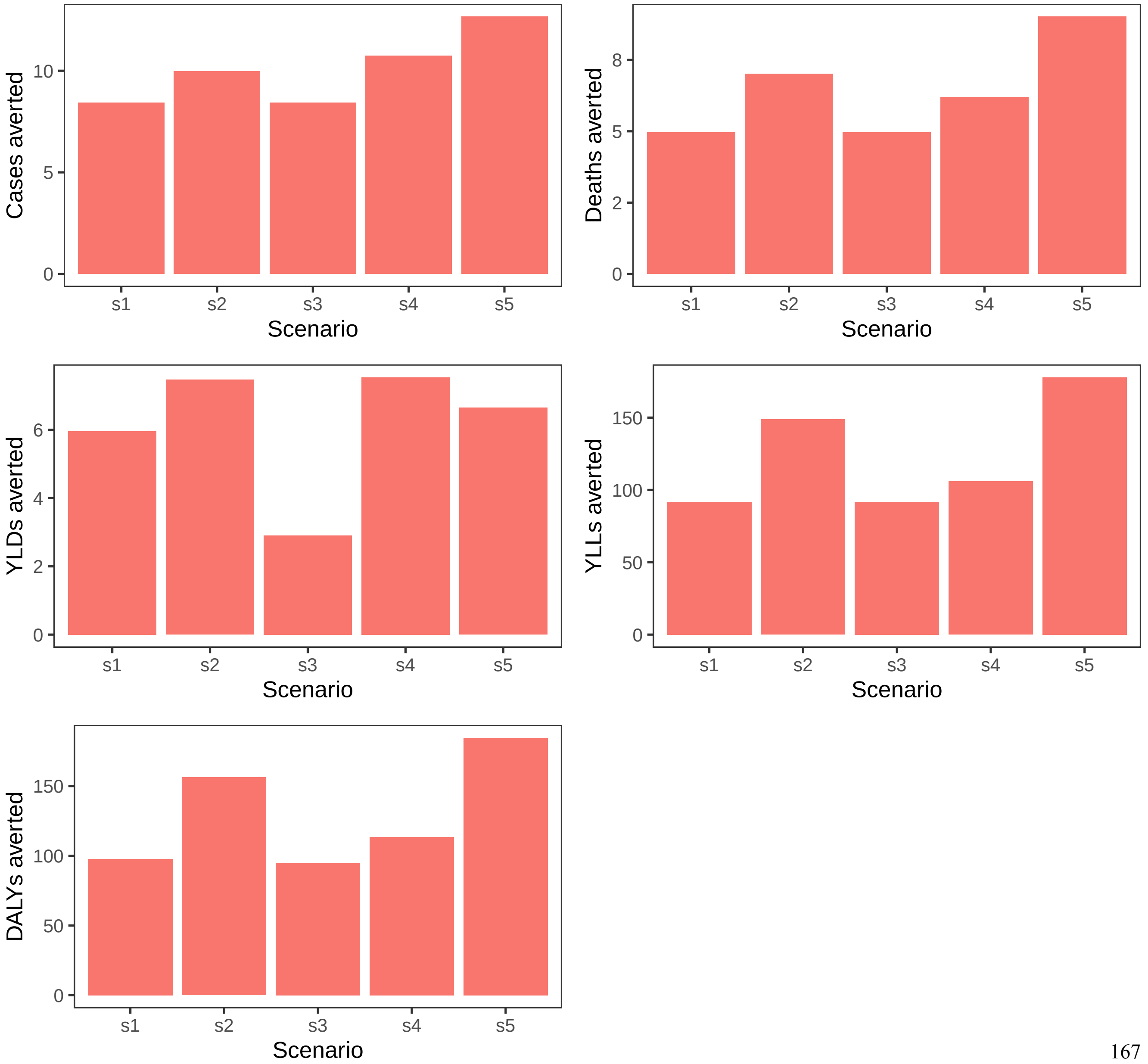

A14. HPV vaccination impact of 12-year-old girls at the national level

Lifetime health impact of bivalent/quadrivalent and nonavalent HPV vaccination of 12-year-old girls during 2020-2029 on cases, deaths and DALYs averted per 1000 vaccinated girls in 177 countries (estimates after the combined PRIME updates for demography, disability weights and cervical cancer burden).

Lifetime health impact of bivalent/quadrivalent HPV vaccination of 12-year-old girls

Cases averted per 1000 vaccinated girls

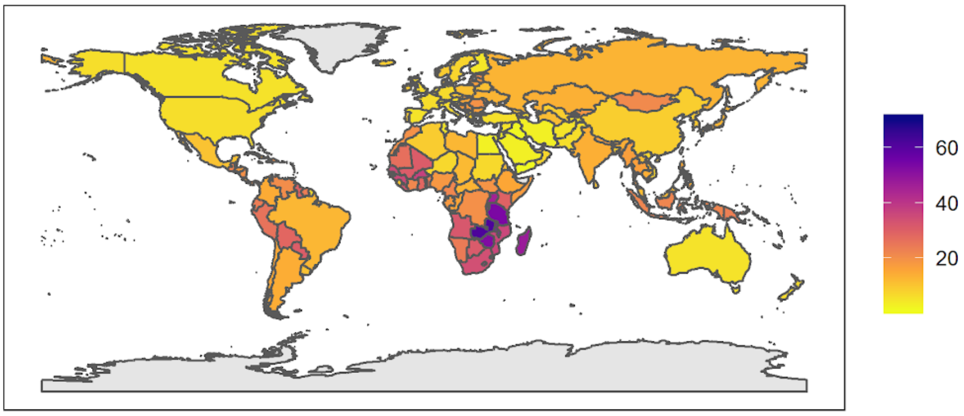

Deaths averted per 1000 vaccinated girls

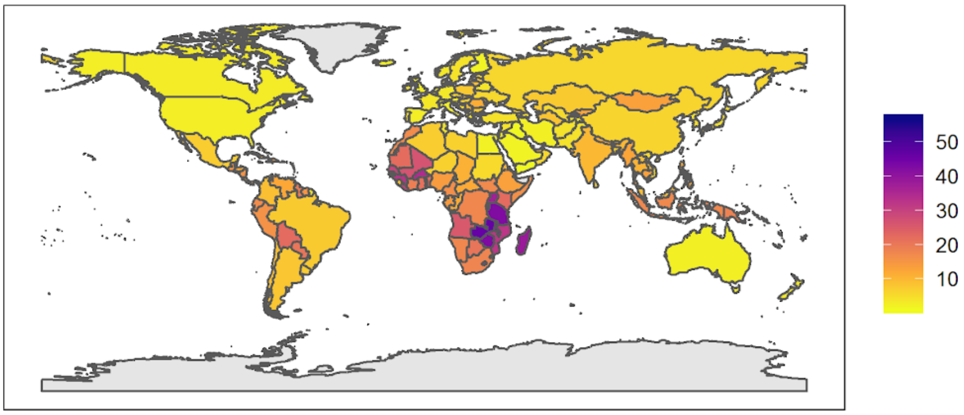

DALYs averted per 1000 vaccinated girls

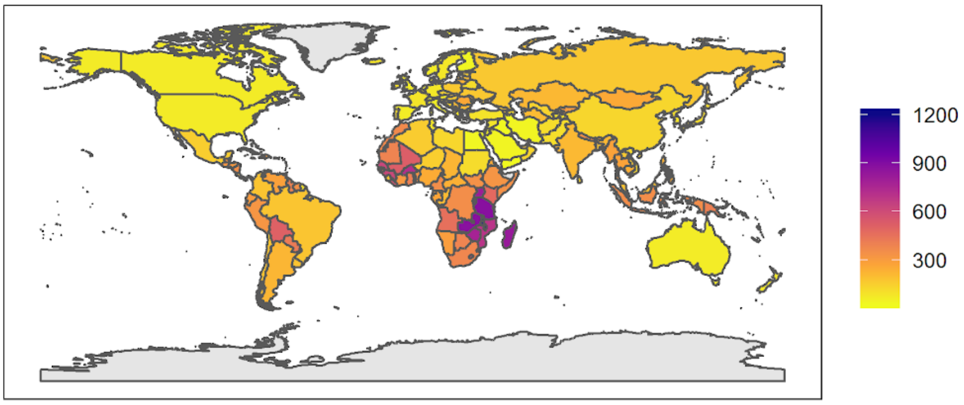

Lifetime health impact of nonavalent HPV vaccination of 12-year-old girls

Cases averted per 1000 vaccinated girls

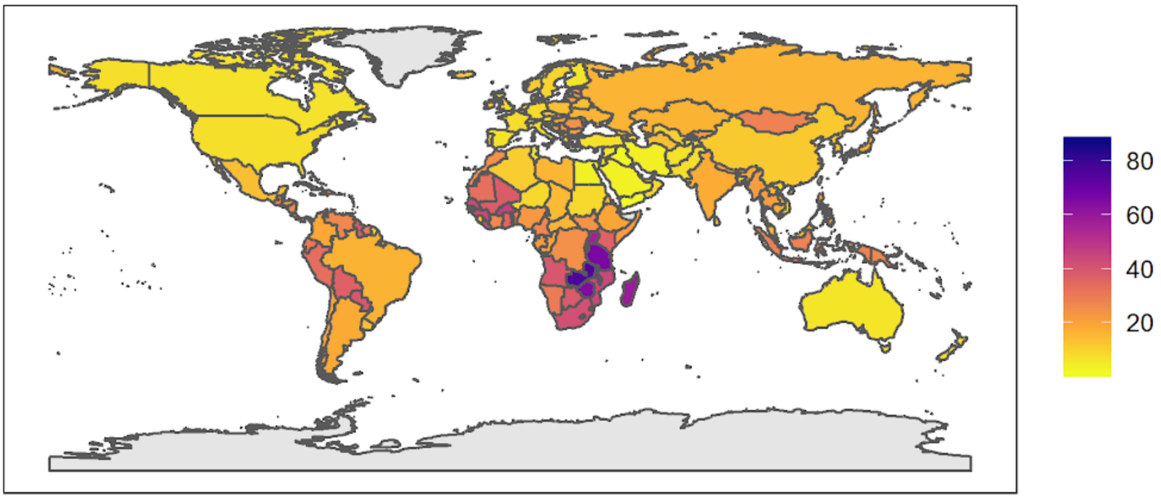

Deaths averted per 1000 vaccinated girls

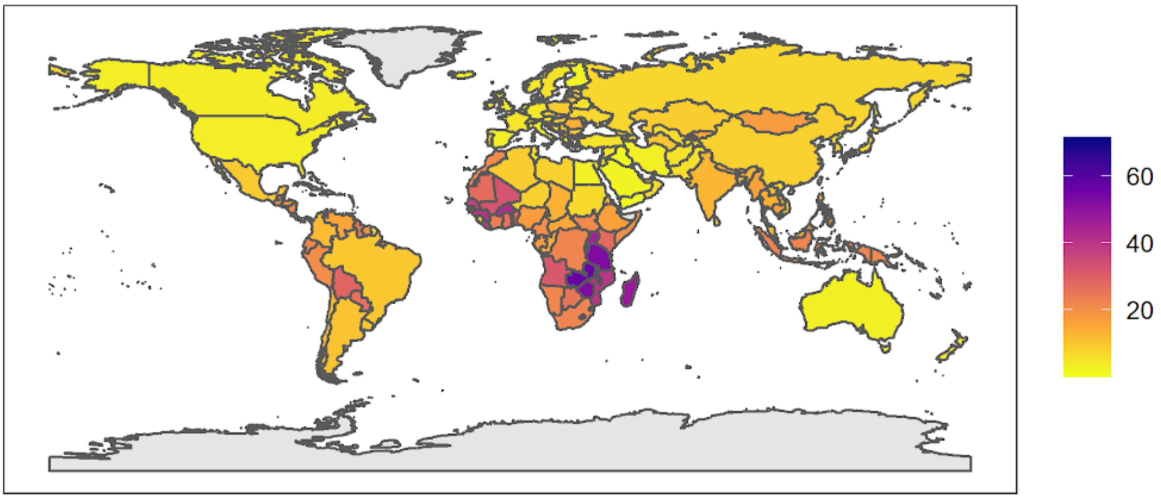

DALYs averted per 1000 vaccinated girls

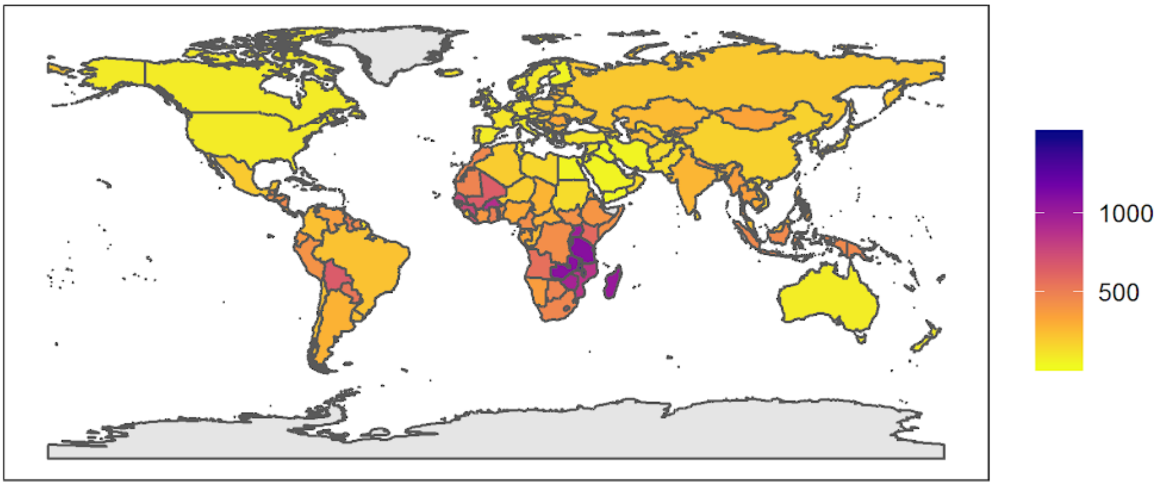

A15. National estimates of HPV vaccination impact for comparative scenarios

Lifetime health impact of HPV vaccination on cases, deaths, YLDs, YLLs and DALYs averted per 1000 vaccinated girls in 177 countries for the 5 comparative scenarios for bivalent/quadrivalent and nonavalent vaccination of 9-year-old and 12-year-old girls.

scenario s1 / vaccination age = 9 years / bivalent/quadrivalent vaccine

Cases averted per 1000 vaccinated girls

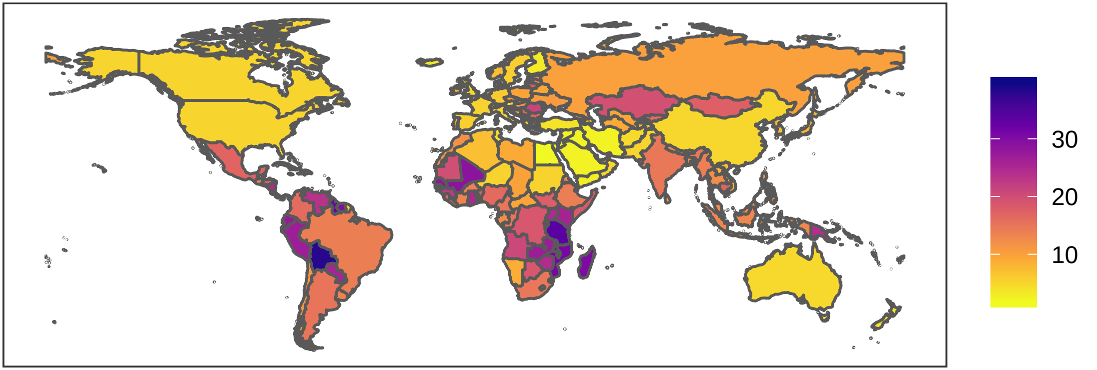

Deaths averted per 1000 vaccinated girls

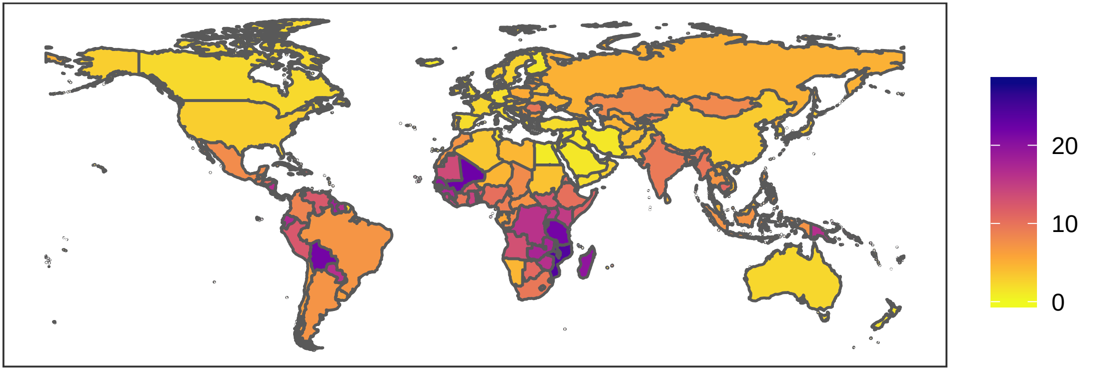

DALYs averted per 1000 vaccinated girls

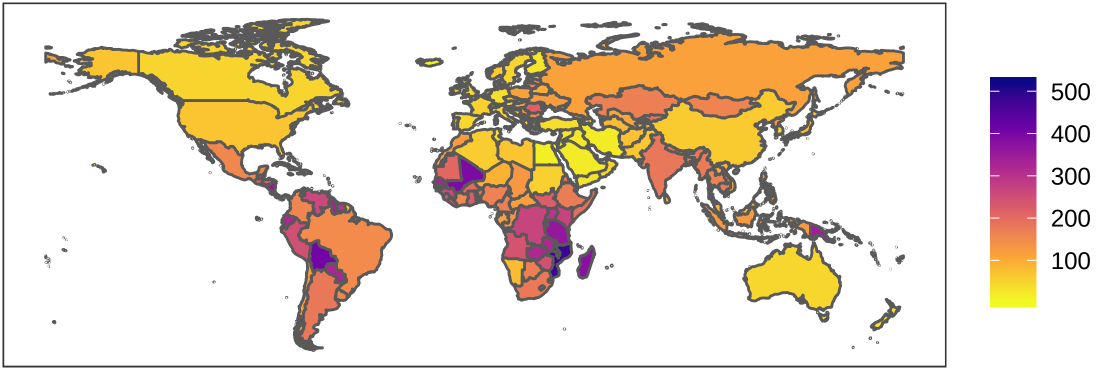

scenario s1 / vaccination age = 9 years / bivalent/quadrivalent vaccine

YLDs averted per 1000 vaccinated girls

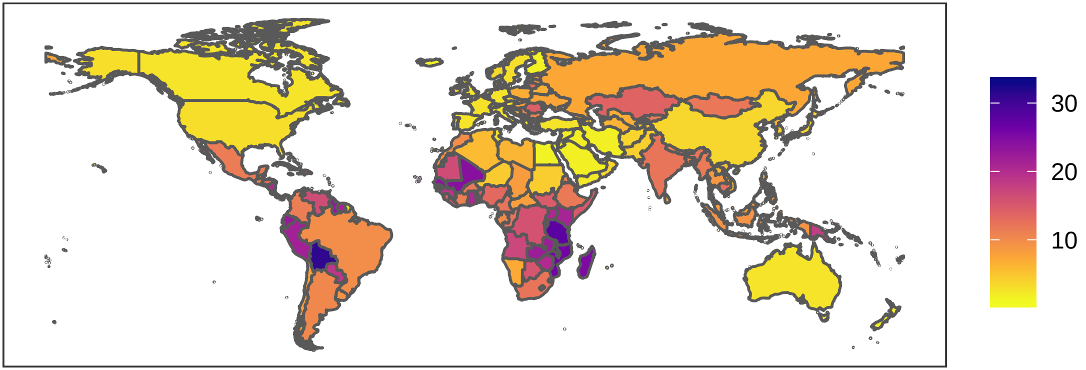

YLLs averted per 1000 vaccinated girls

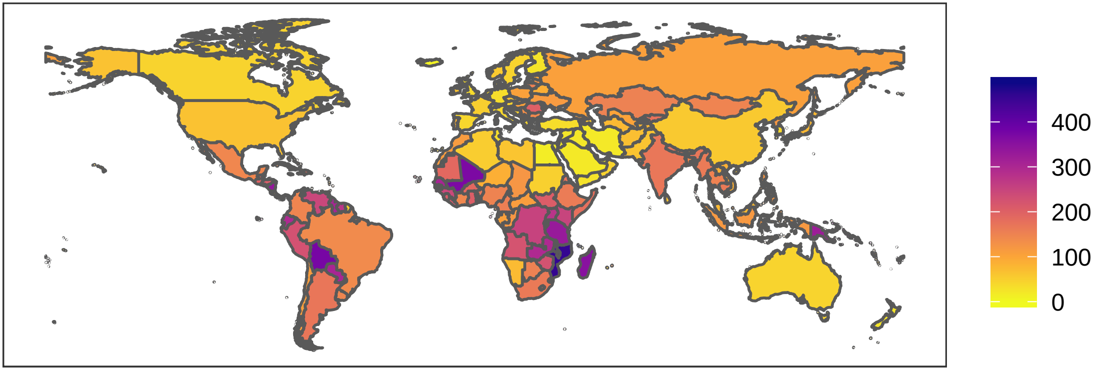

DALYs averted per 1000 vaccinated girls

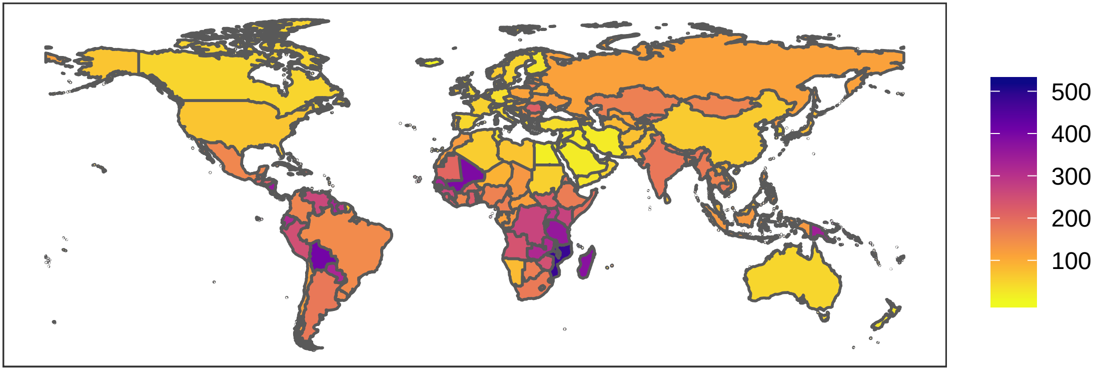

scenario s2 / vaccination age = 9 years / bivalent/quadrivalent vaccine

Cases averted per 1000 vaccinated girls

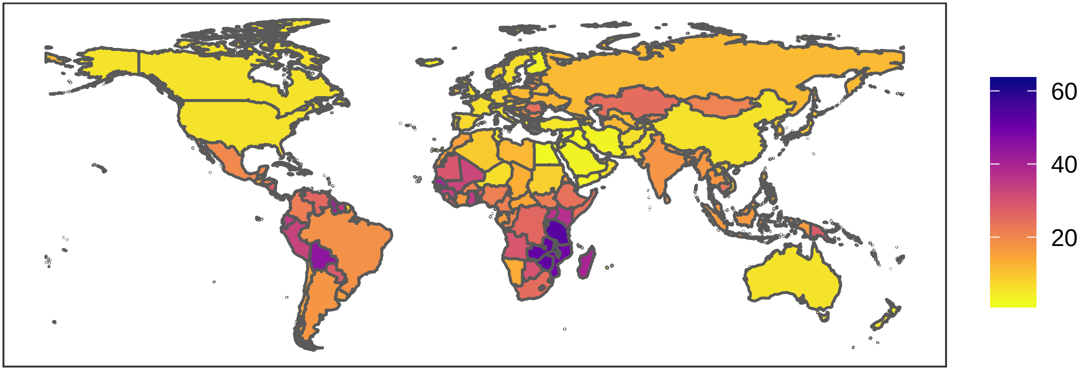

Deaths averted per 1000 vaccinated girls

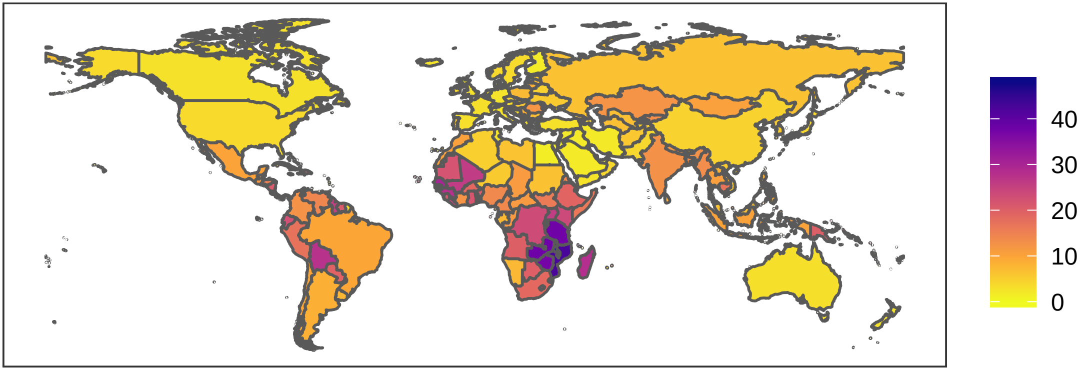

DALYs averted per 1000 vaccinated girls

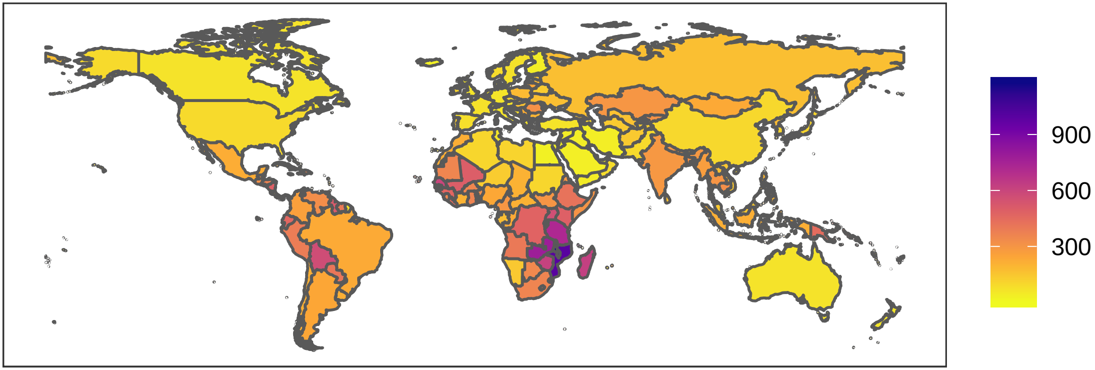

scenario s2 / vaccination age = 9 years / bivalent/quadrivalent vaccine

YLDs averted per 1000 vaccinated girls

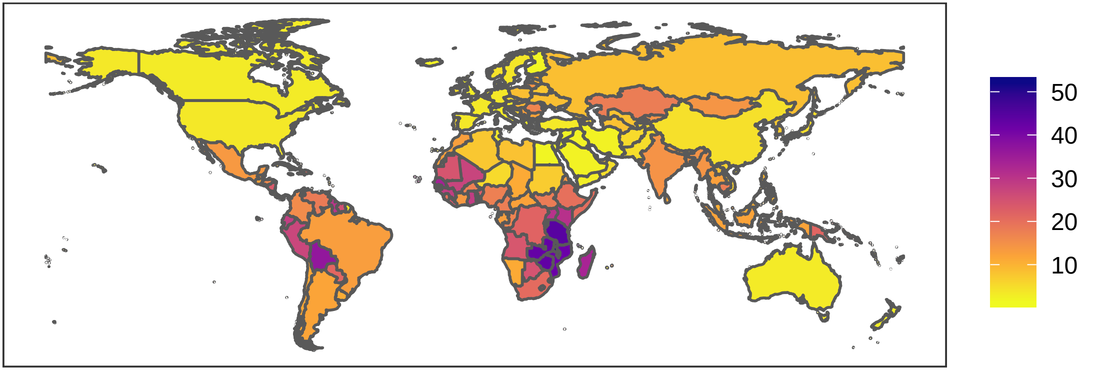

YLLs averted per 1000 vaccinated girls

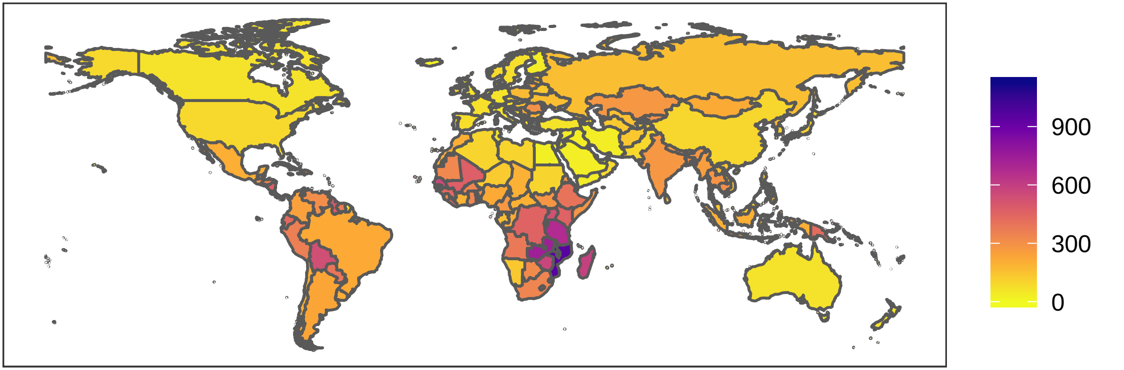

DALYs averted per 1000 vaccinated girls

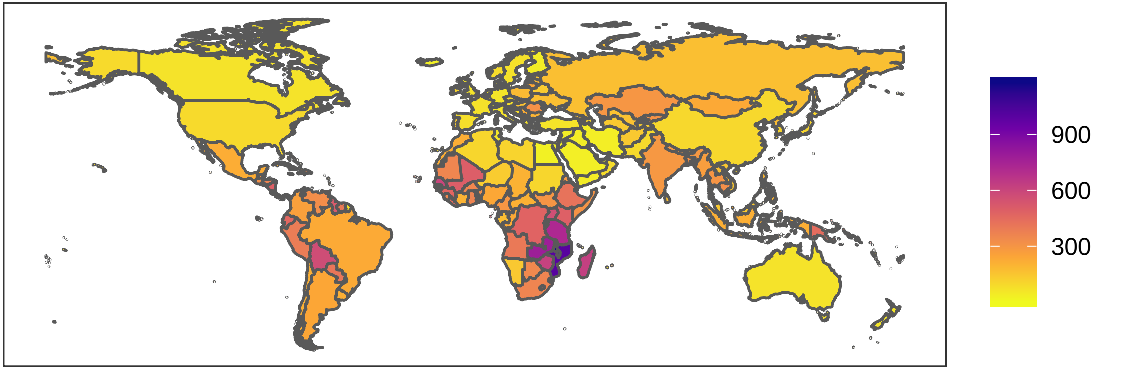

scenario s3 / vaccination age = 9 years / bivalent/quadrivalent vaccine

Cases averted per 1000 vaccinated girls

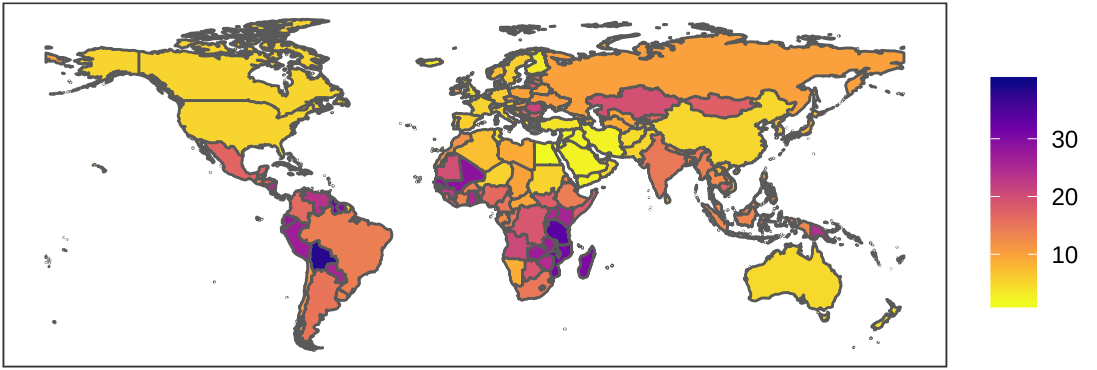

Deaths averted per 1000 vaccinated girls

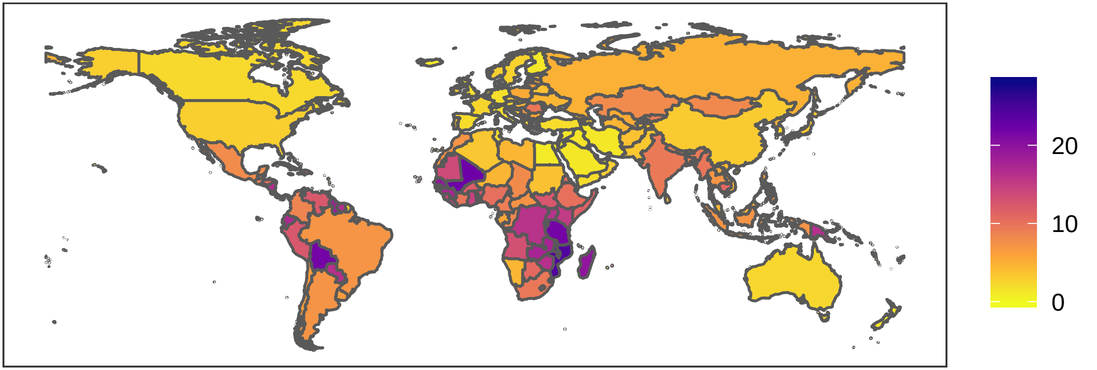

DALYs averted per 1000 vaccinated girls

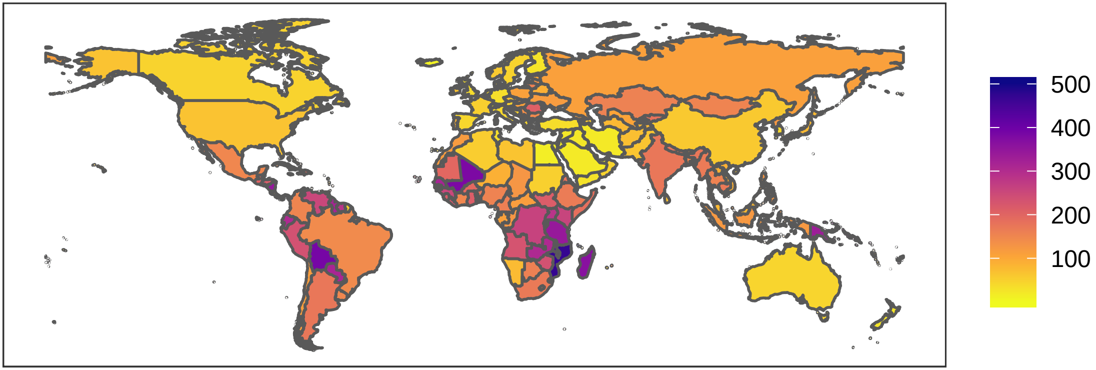

scenario s3 / vaccination age = 9 years / bivalent/quadrivalent vaccine

YLDs averted per 1000 vaccinated girls

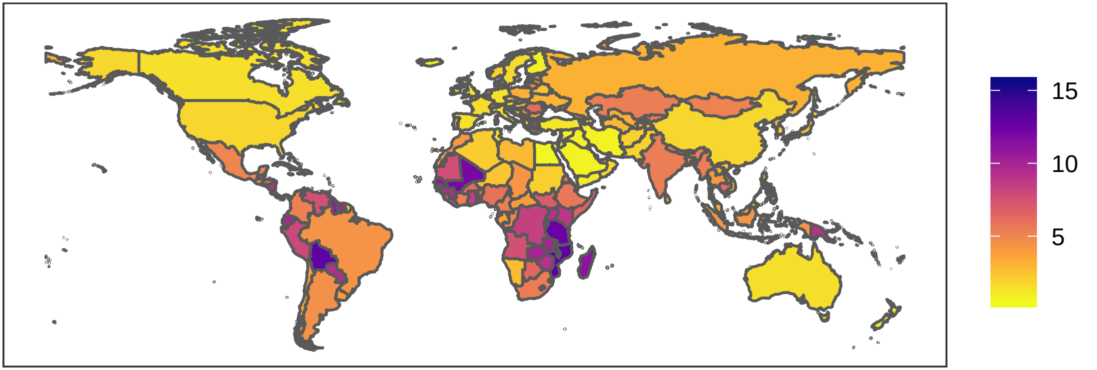

YLLs averted per 1000 vaccinated girls

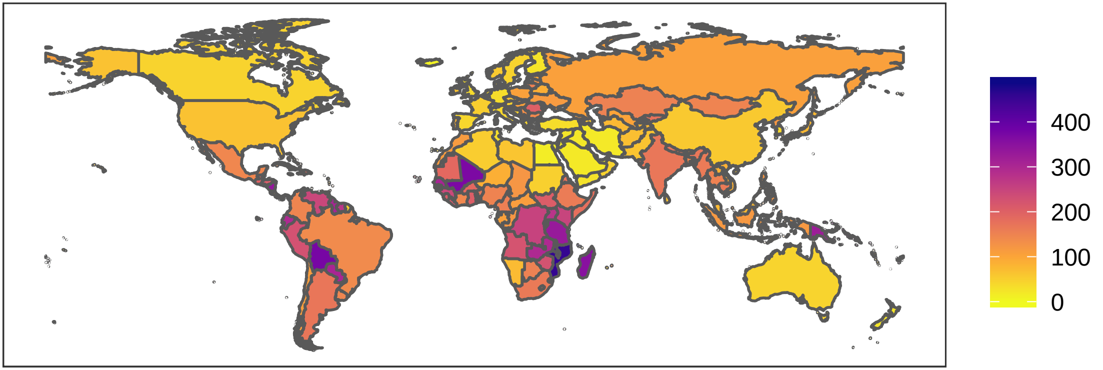

DALYs averted per 1000 vaccinated girls

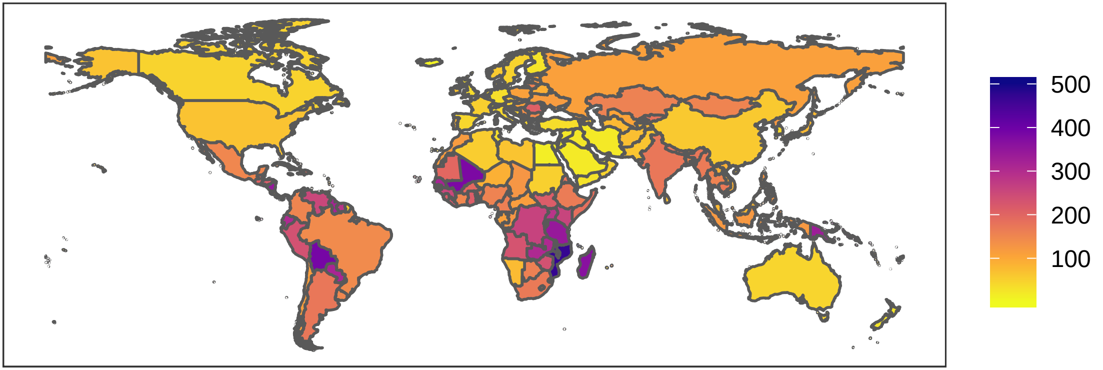

scenario s4 / vaccination age = 9 years / bivalent/quadrivalent vaccine

Cases averted per 1000 vaccinated girls

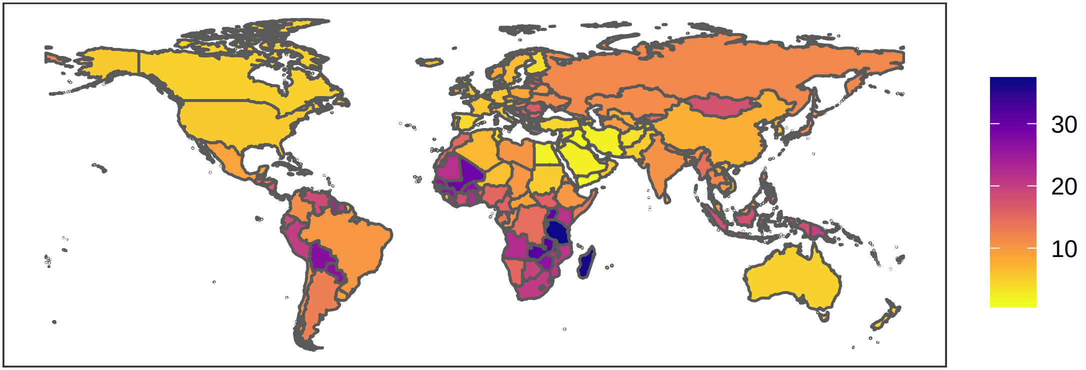

Deaths averted per 1000 vaccinated girls

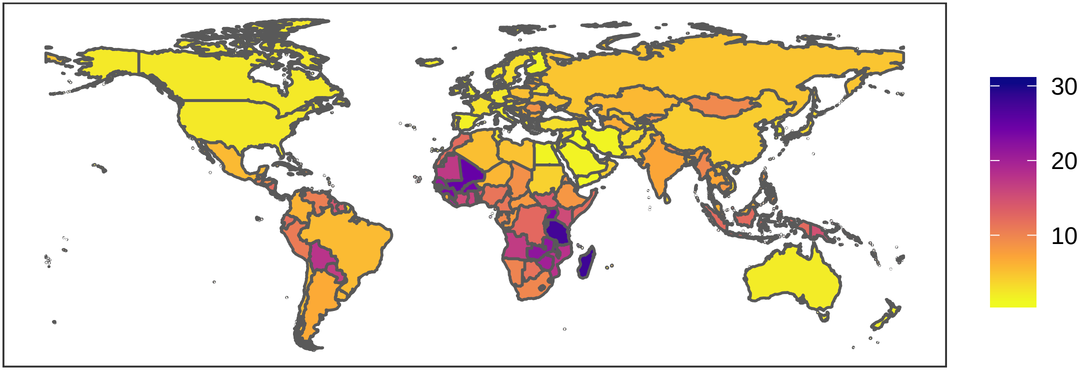

DALYs averted per 1000 vaccinated girls

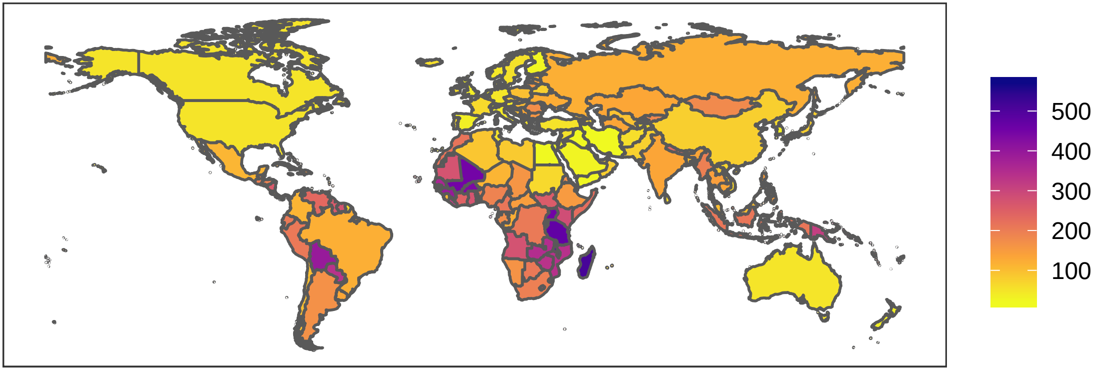

scenario s4 / vaccination age = 9 years / bivalent/quadrivalent vaccine

YLDs averted per 1000 vaccinated girls

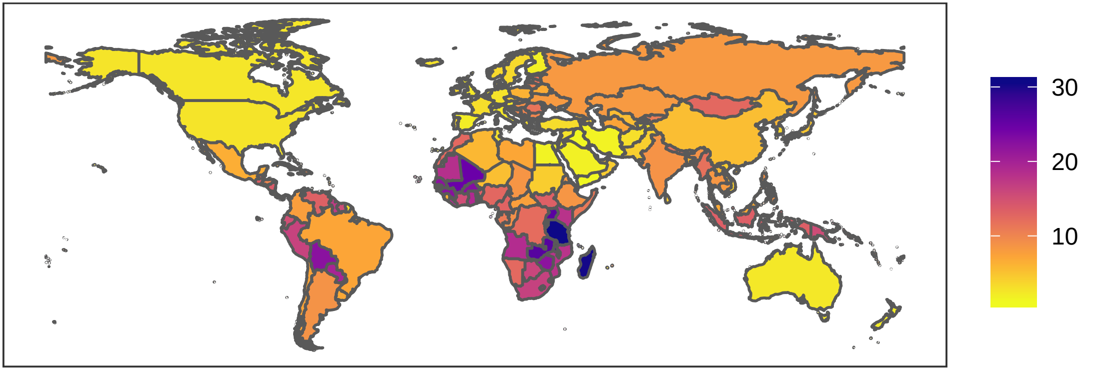

YLLs averted per 1000 vaccinated girls

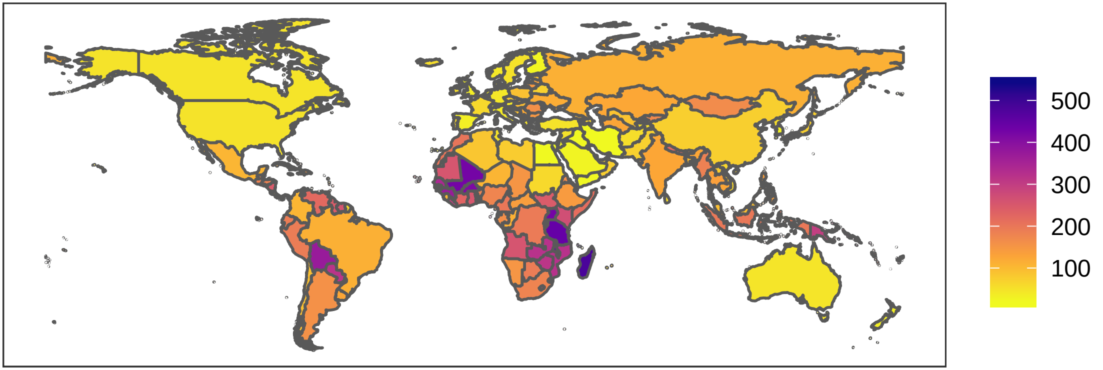

DALYs averted per 1000 vaccinated girls

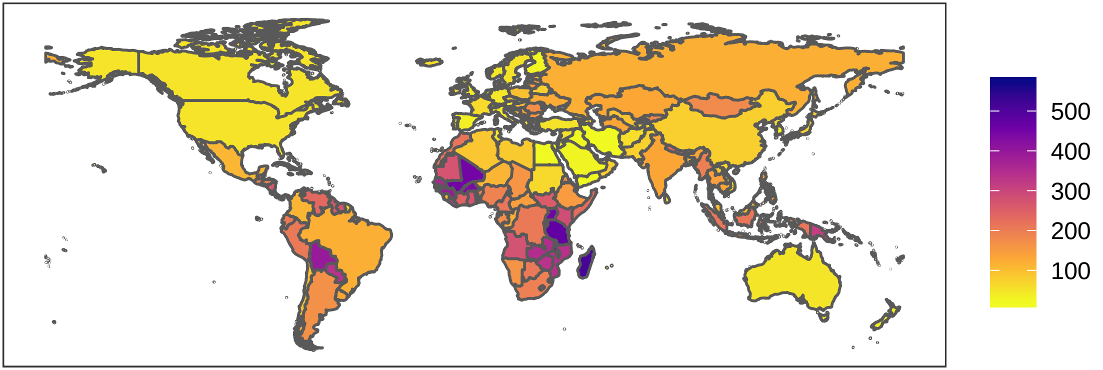

scenario s5 / vaccination age = 9 years / bivalent/quadrivalent vaccine

Cases averted per 1000 vaccinated girls

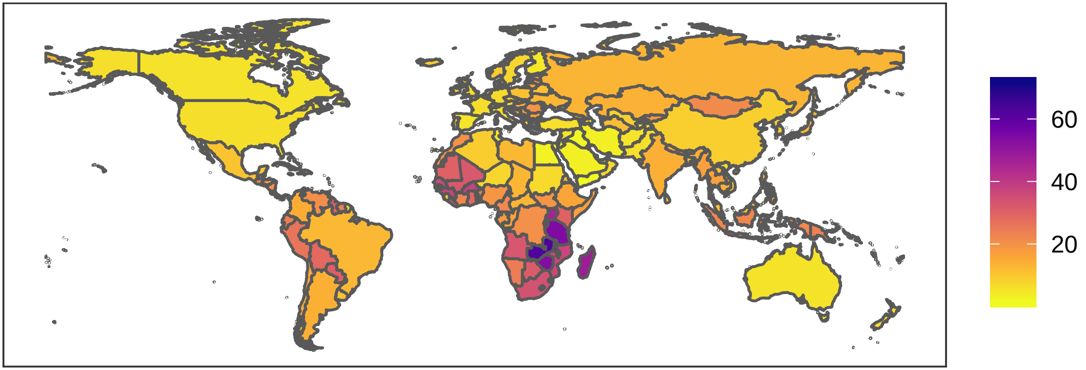

Deaths averted per 1000 vaccinated girls

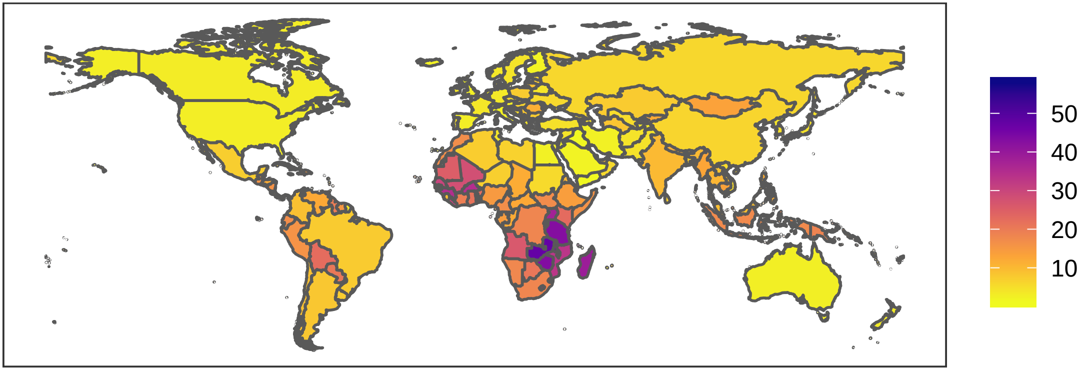

DALYs averted per 1000 vaccinated girls

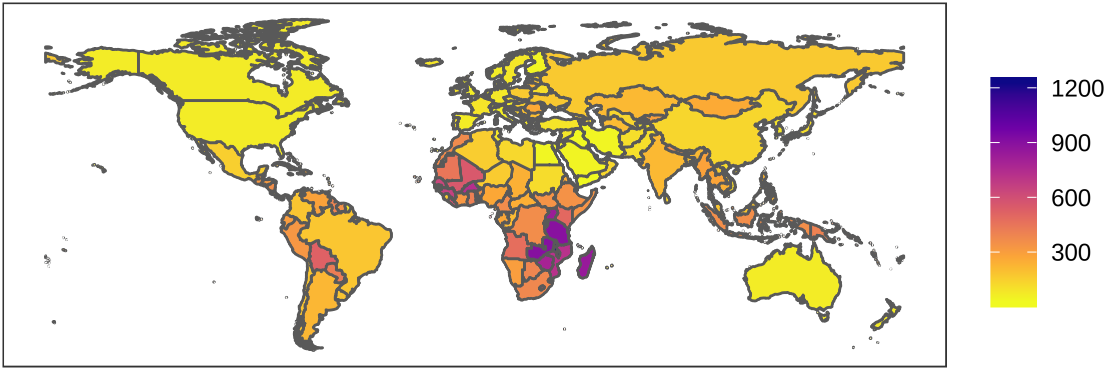

scenario s5 / vaccination age = 9 years / bivalent/quadrivalent vaccine

YLDs averted per 1000 vaccinated girls

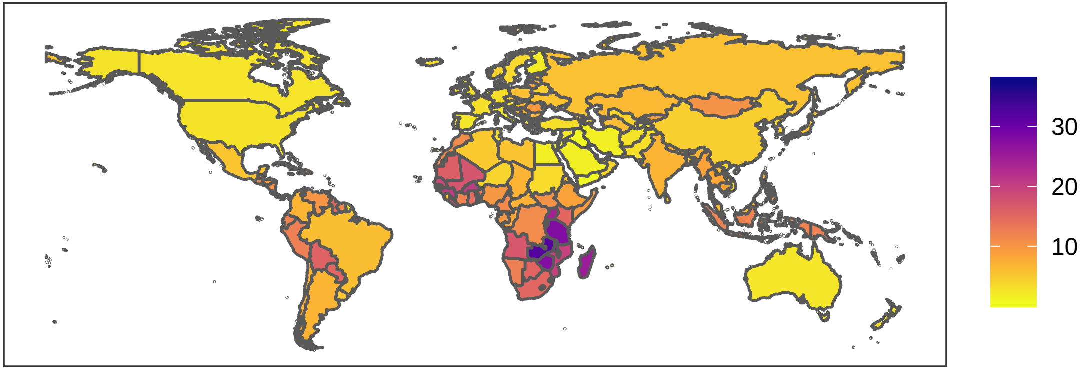

YLLs averted per 1000 vaccinated girls

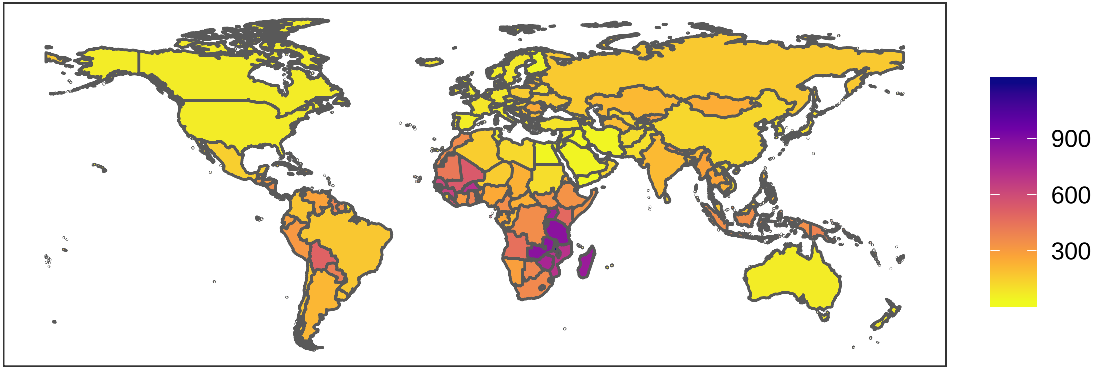

DALYs averted per 1000 vaccinated girls

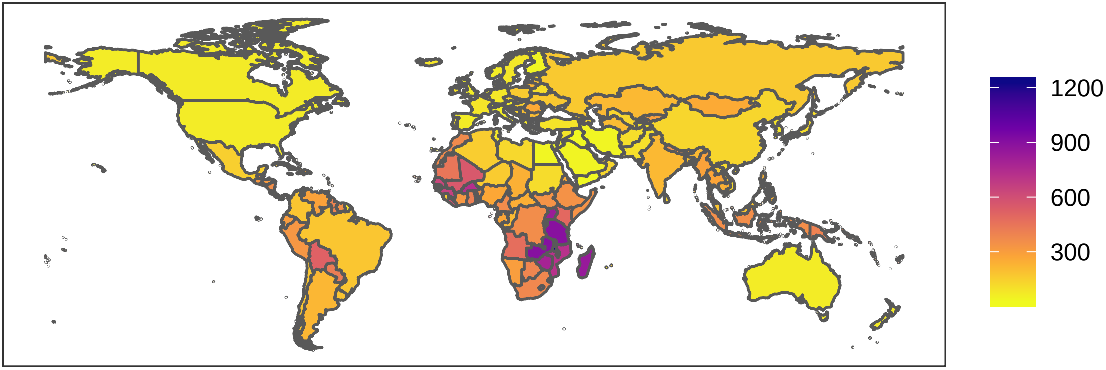

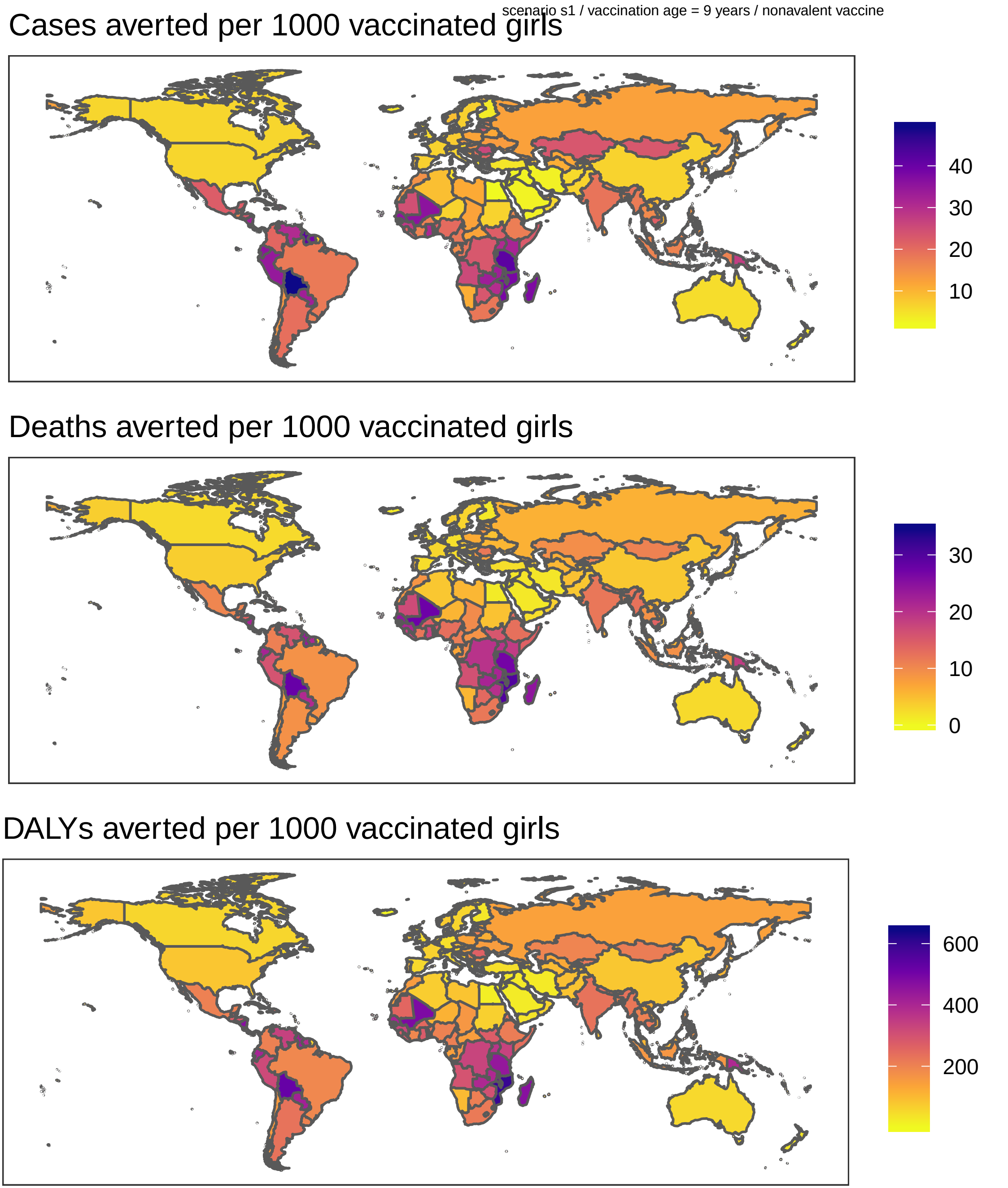
scenario s1 / vaccination age = 9 years / nonavalent vaccine
Cases averted per 1000 vaccinated girls
40
30
20
10
Deaths averted per 1000 vaccinated girls
30
20
10
0
DALYs averted per 1000 vaccinated girls
600
400
200

scenario s1 / vaccination age = 9 years / nonavalent vaccine

YLDs averted per 1000 vaccinated girls

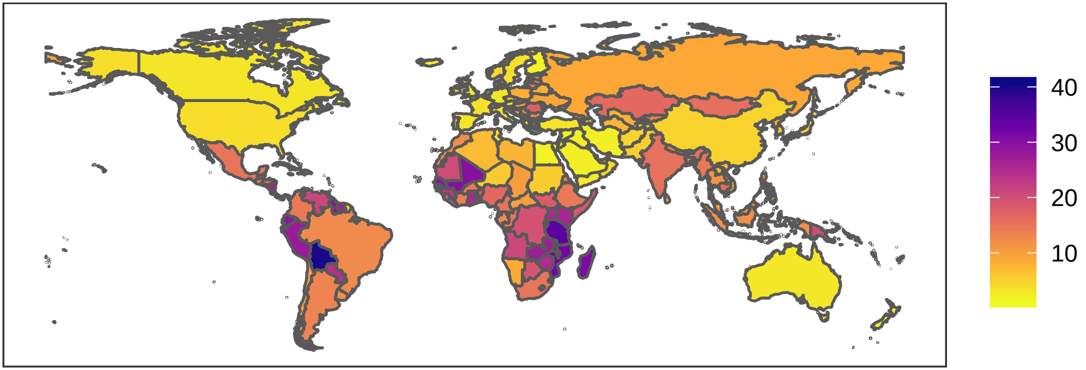

YLLs averted per 1000 vaccinated girls

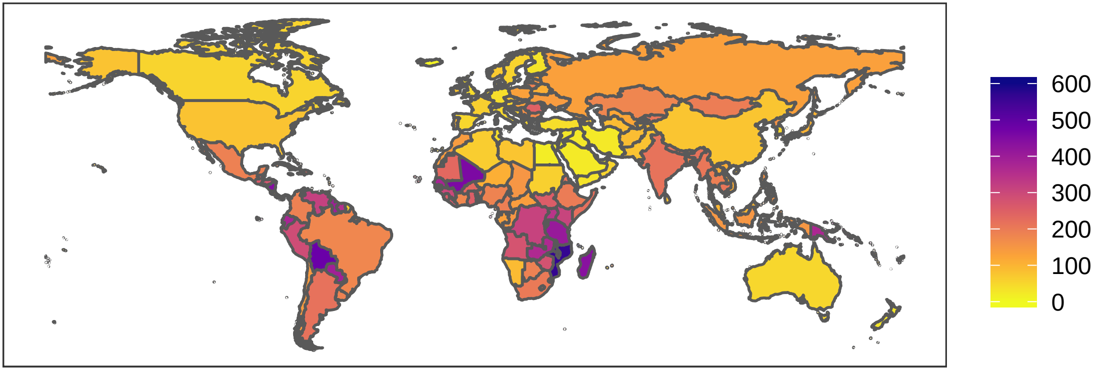

DALYs averted per 1000 vaccinated girls

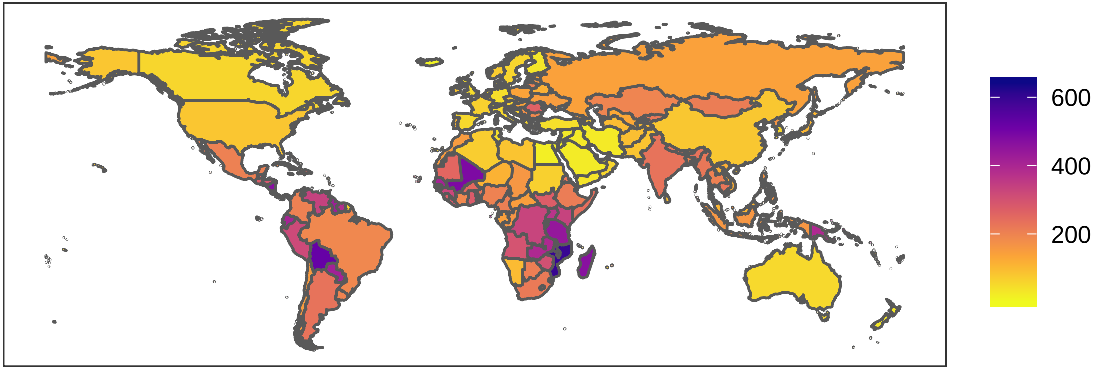

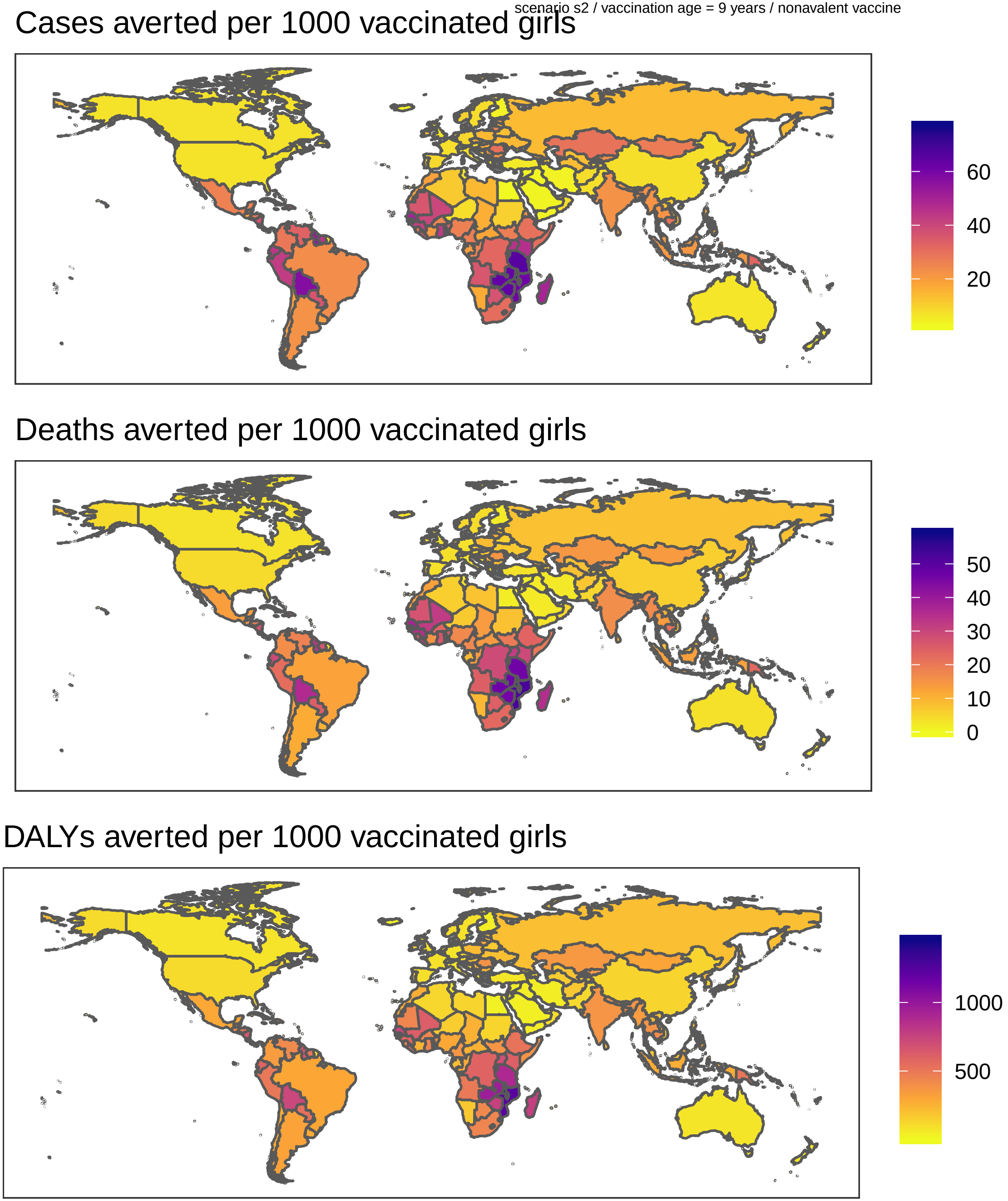
scenario s2 / vaccination age = 9 years / nonavalent vaccine
Cases averted per 1000 vaccinated girls
60
40
20
Deaths averted per 1000 vaccinated girls
50
40
30
20
10
0
DALYs averted per 1000 vaccinated girls
1000
500

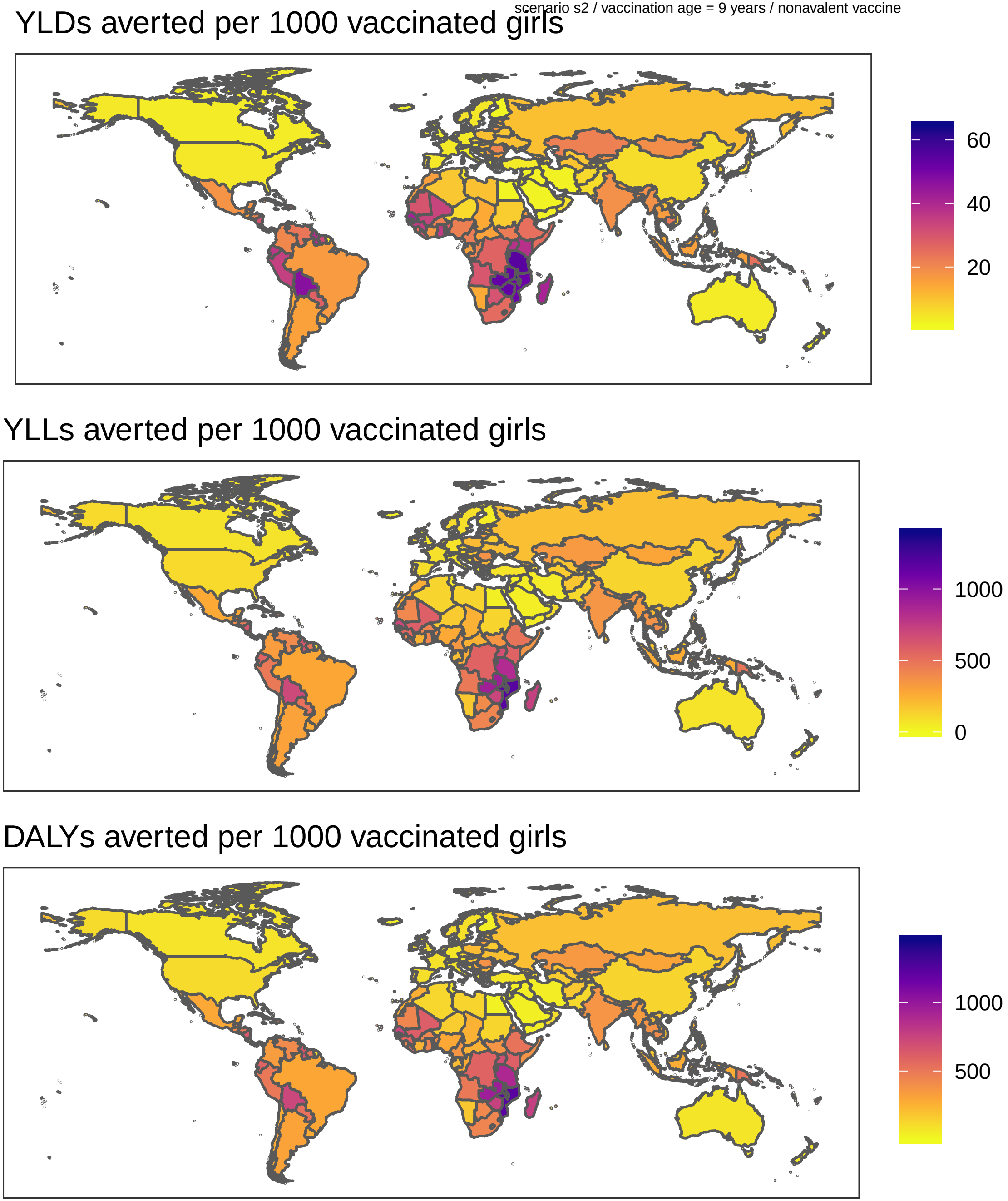
scenario s2 / vaccination age = 9 years / nonavalent vaccine
YLDs averted per 1000 vaccinated girls
60
40
20
YLLs averted per 1000 vaccinated girls
1000
500
0
DALYs averted per 1000 vaccinated girls
1000
500

scenario s3 / vaccination age = 9 years / nonavalent vaccine

Cases averted per 1000 vaccinated girls

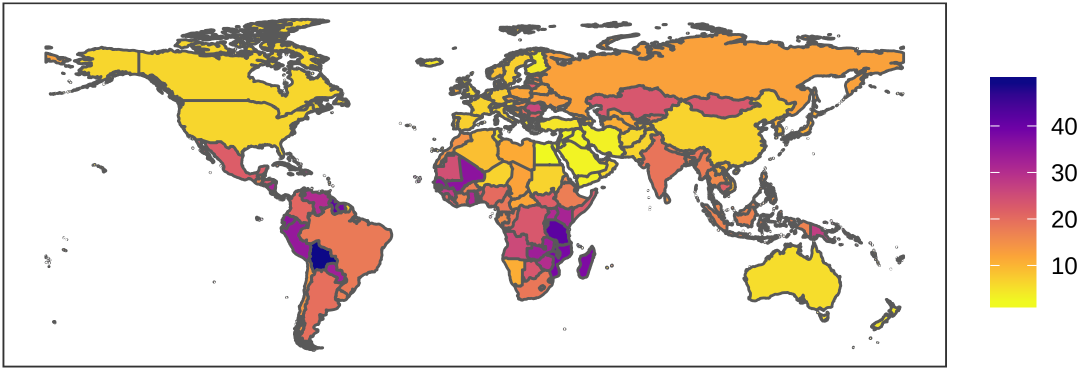

Deaths averted per 1000 vaccinated girls

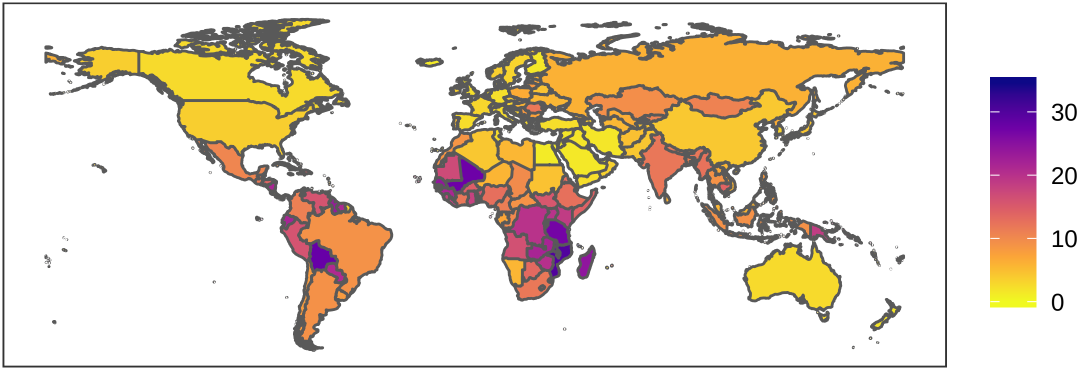

DALYs averted per 1000 vaccinated girls

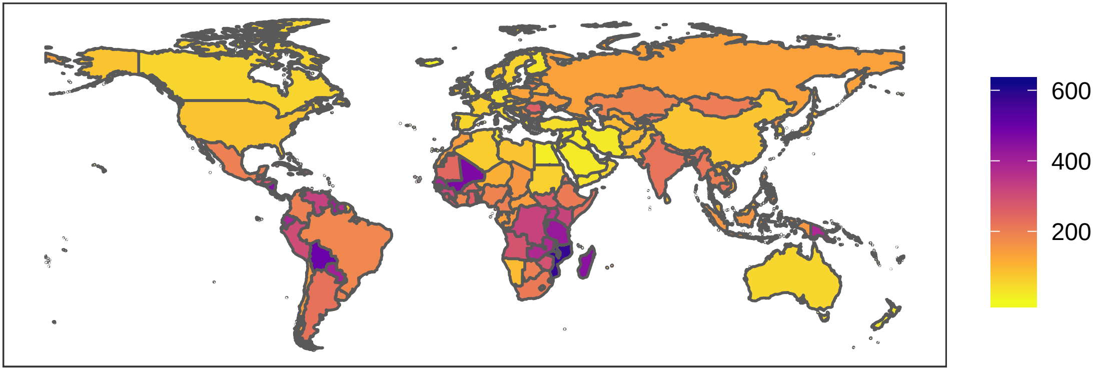

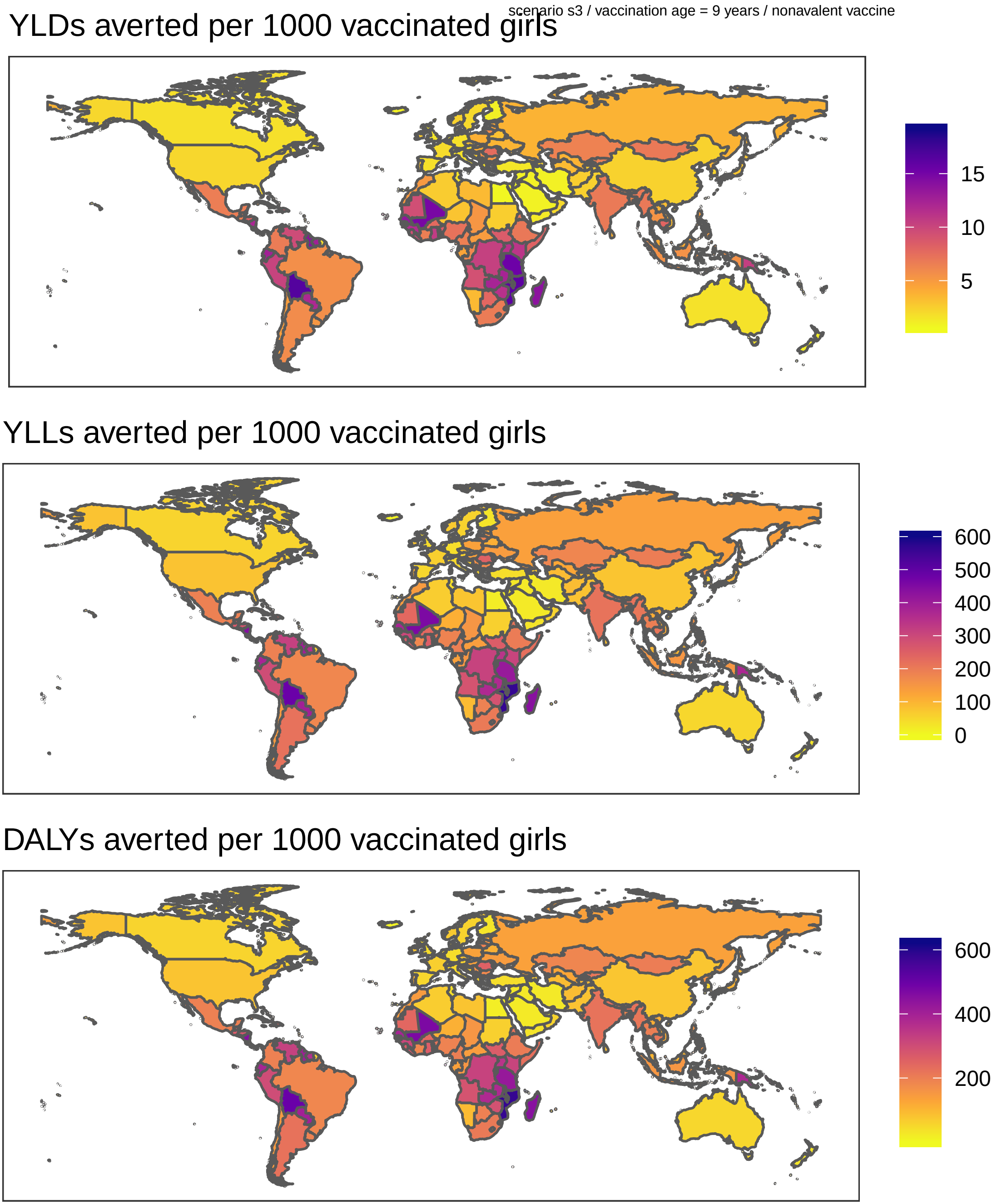
scenario s3 / vaccination age = 9 years / nonavalent vaccine
YLDs averted per 1000 vaccinated girls
15
10
5
YLLs averted per 1000 vaccinated girls
600
500
400
300
200
100
0
DALYs averted per 1000 vaccinated girls
600
400
200

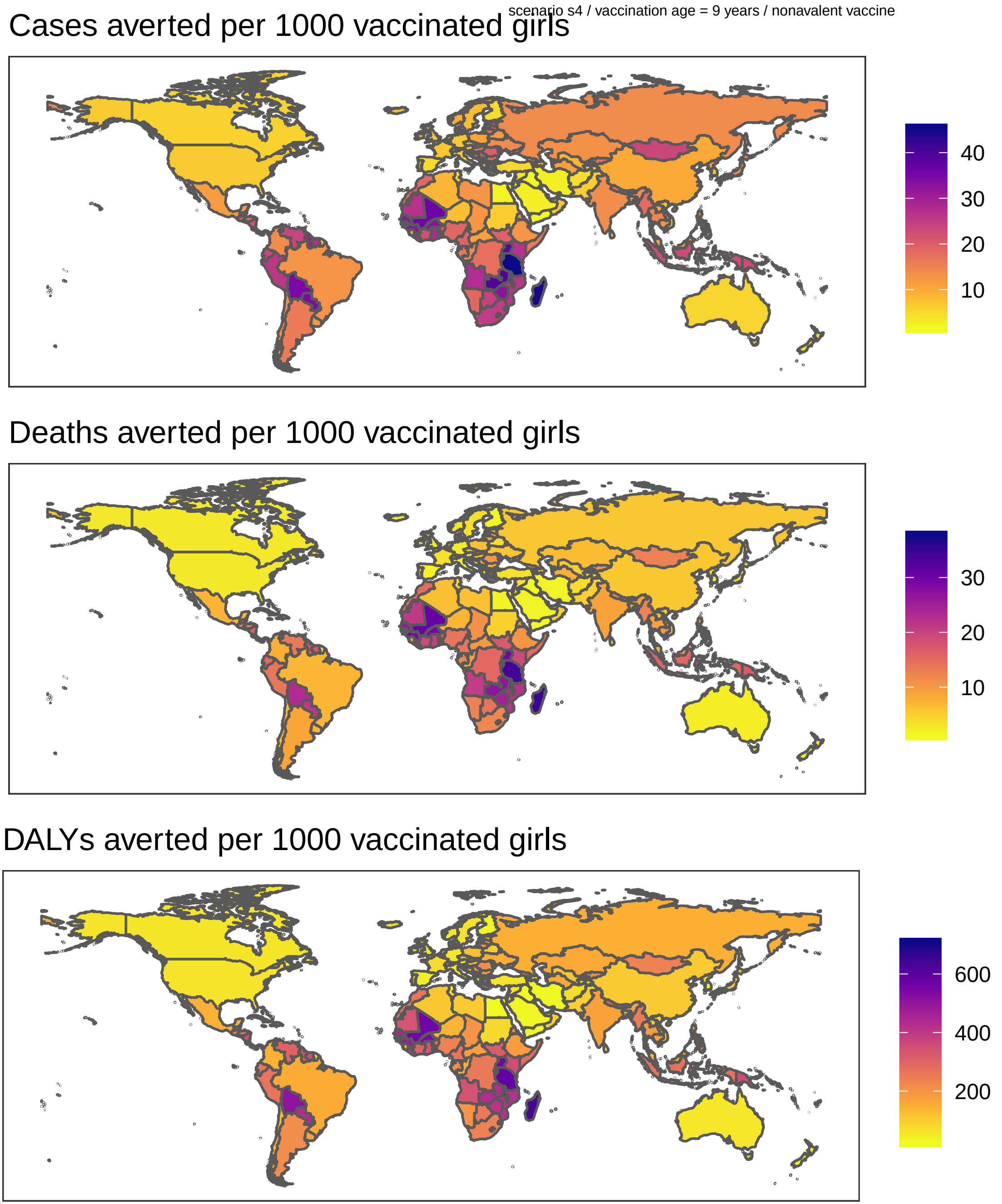

scenario s4 / vaccination age = 9 years / nonavalent vaccine
Cases averted per 1000 vaccinated girls
40
30
20
10
Deaths averted per 1000 vaccinated girls
30
20
10
DALYs averted per 1000 vaccinated girls
600
400
200

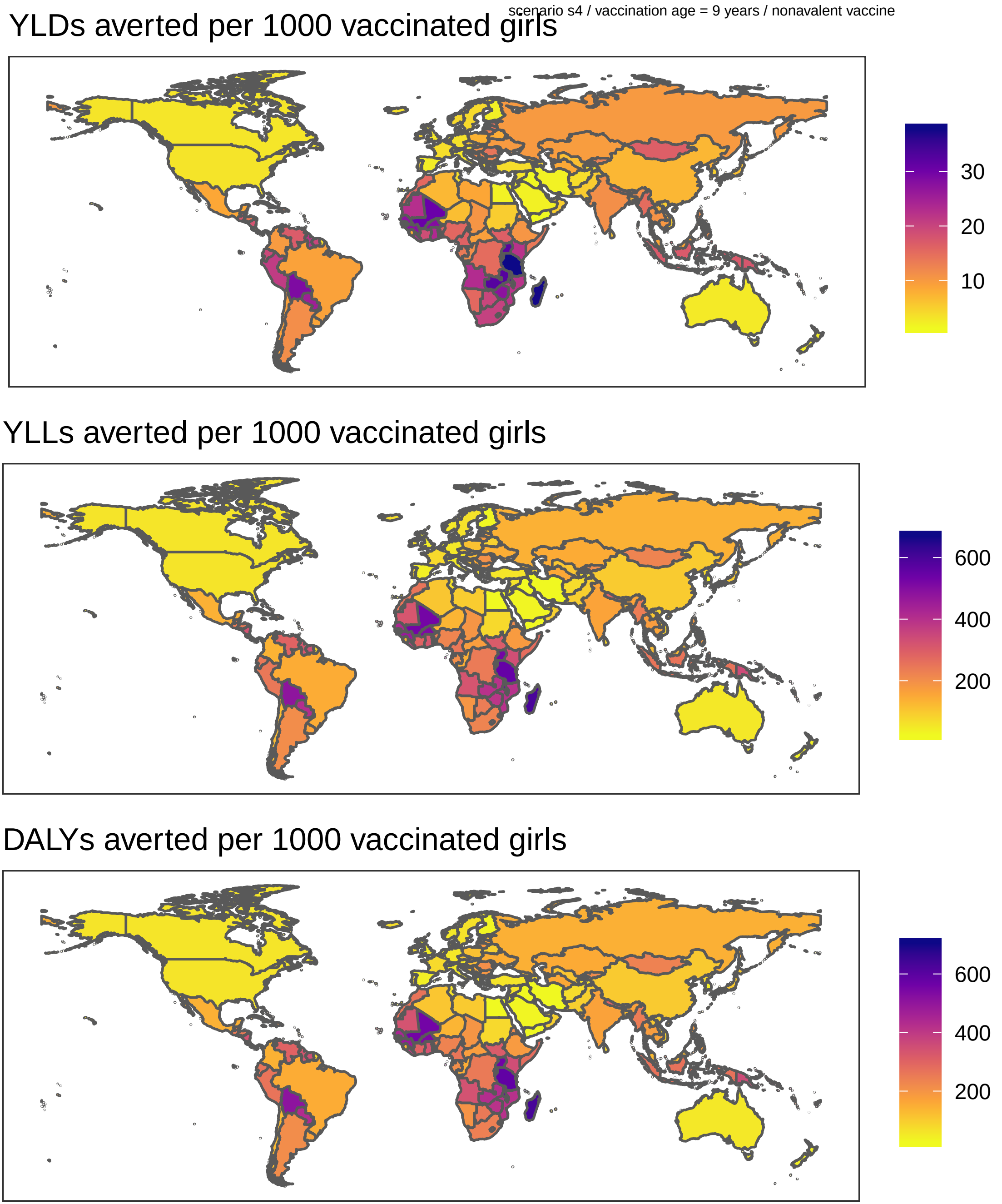
scenario s4 / vaccination age = 9 years / nonavalent vaccine
YLDs averted per 1000 vaccinated girls
30
20
10
YLLs averted per 1000 vaccinated girls
600
400
200
DALYs averted per 1000 vaccinated girls
600
400
200

scenario s5 / vaccination age = 9 years / nonavalent vaccine

Cases averted per 1000 vaccinated girls

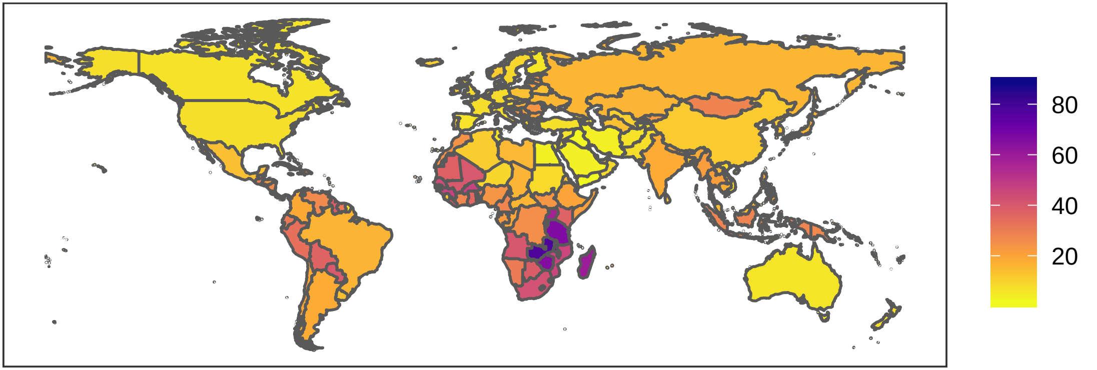

Deaths averted per 1000 vaccinated girls

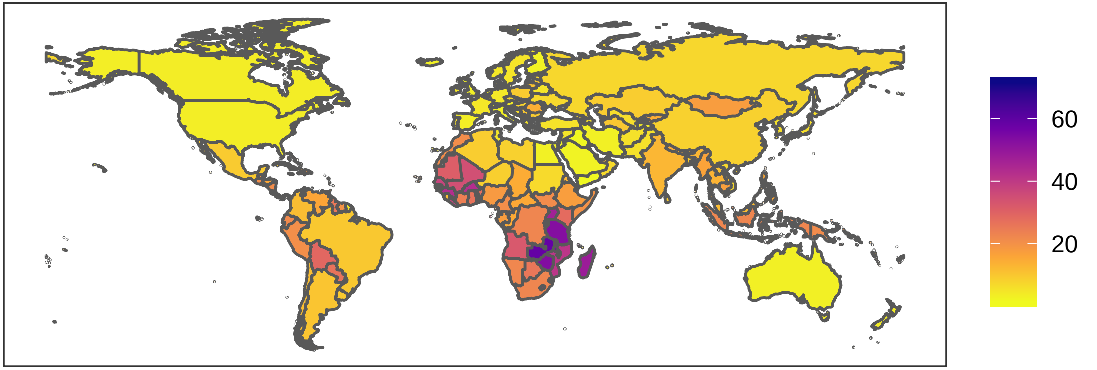

DALYs averted per 1000 vaccinated girls

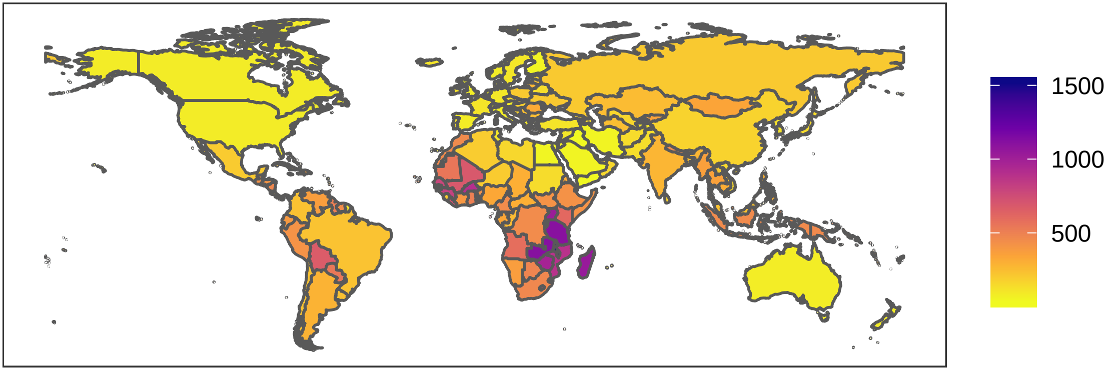

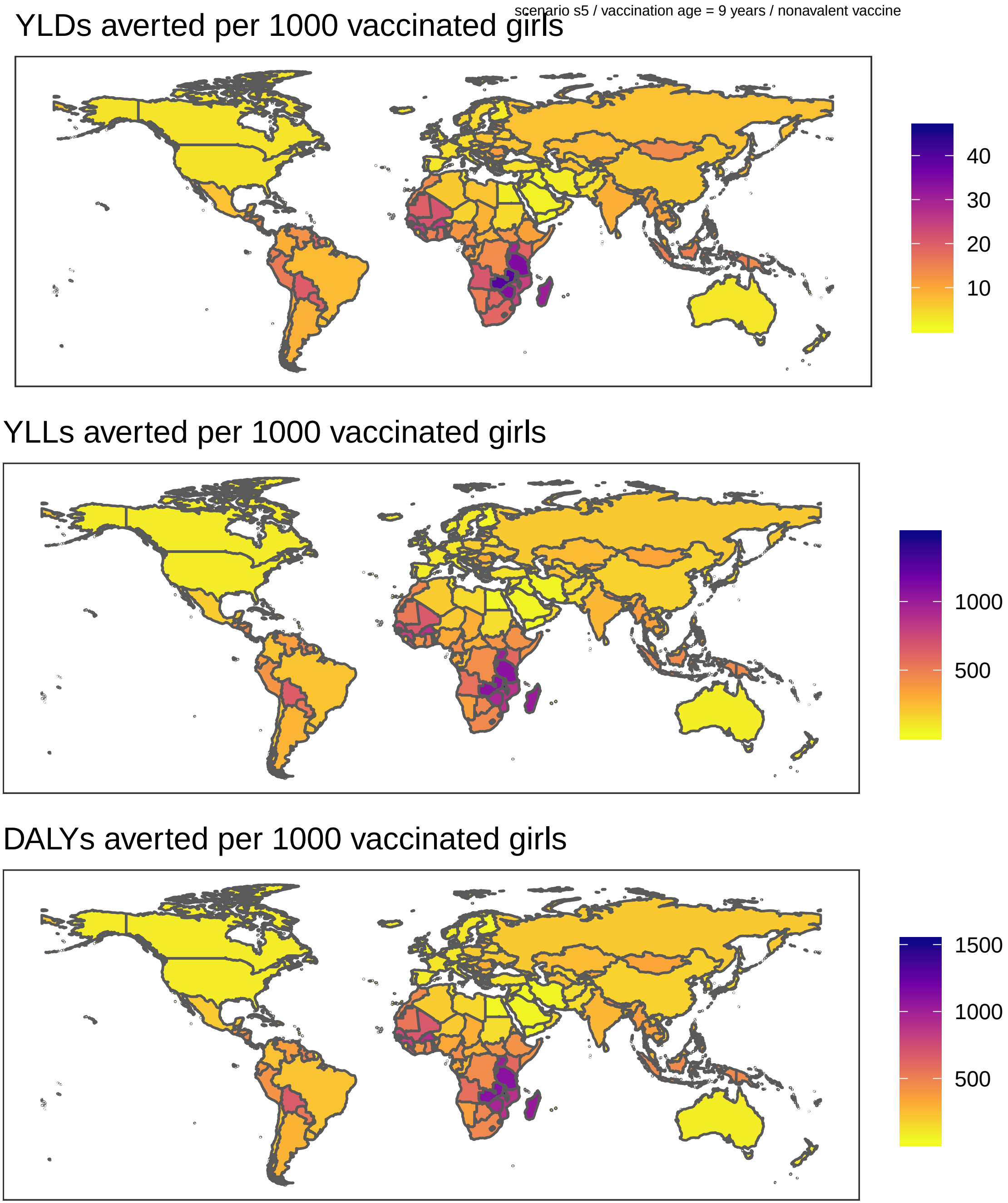
scenario s5 / vaccination age = 9 years / nonavalent vaccine
YLDs averted per 1000 vaccinated girls
40
30
20
10
YLLs averted per 1000 vaccinated girls
1000
500
DALYs averted per 1000 vaccinated girls
1500
1000
500

scenario s1 / vaccination age = 12 years / bivalent/quadrivalent vaccine

Cases averted per 1000 vaccinated girls

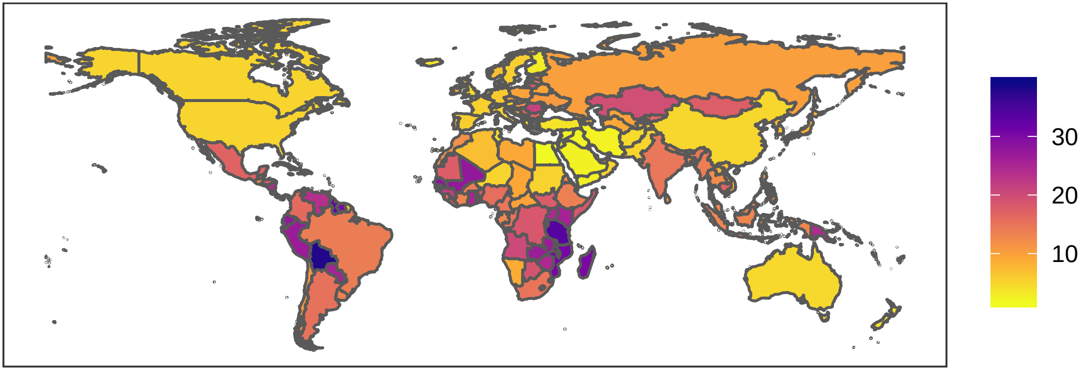

Deaths averted per 1000 vaccinated girls

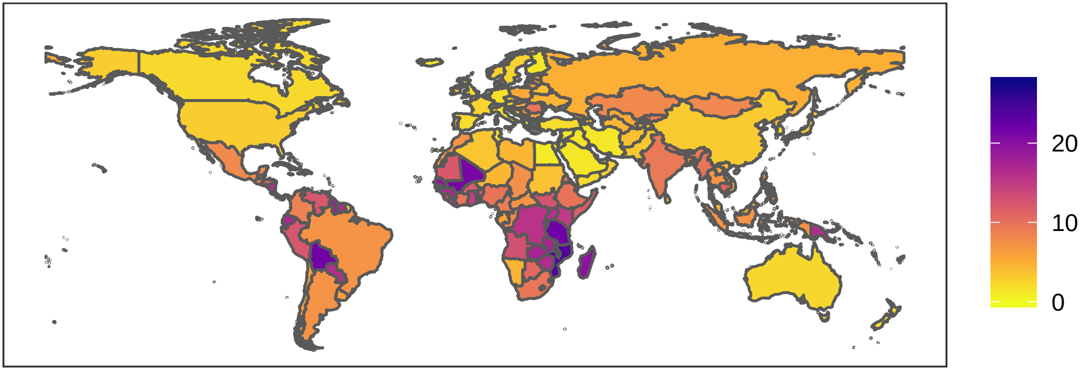

DALYs averted per 1000 vaccinated girls

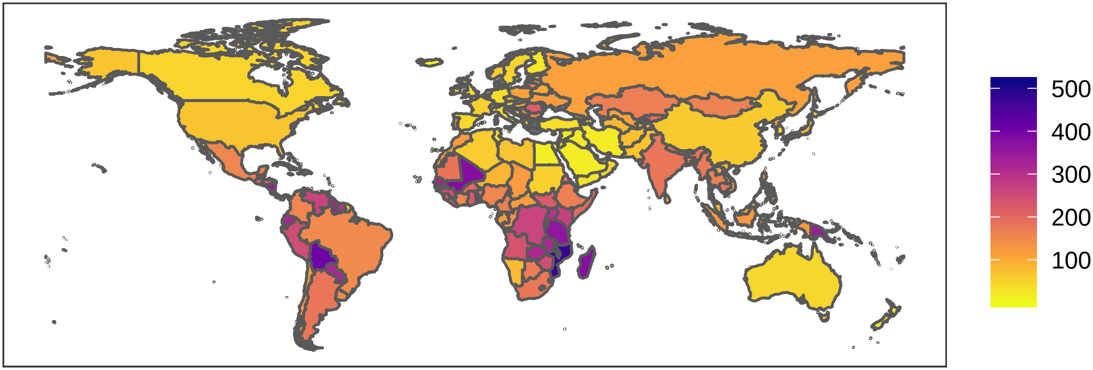

scenario s1 / vaccination age = 12 years / bivalent/quadrivalent vaccine

YLDs averted per 1000 vaccinated girls

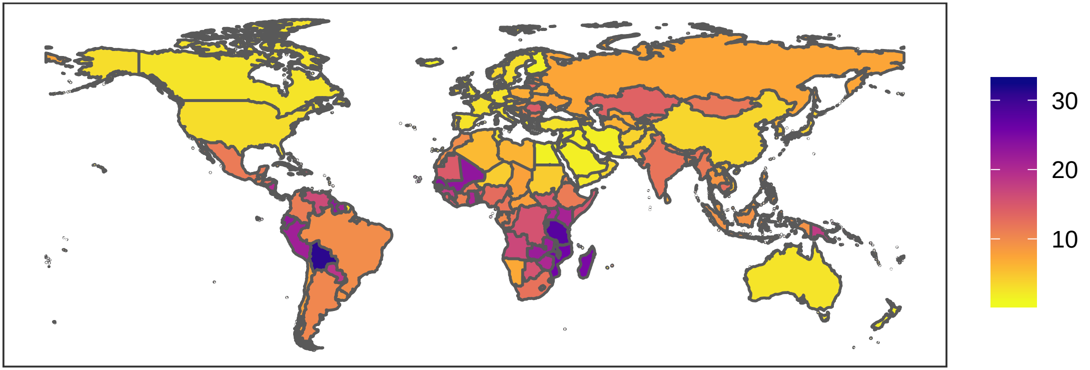

YLLs averted per 1000 vaccinated girls

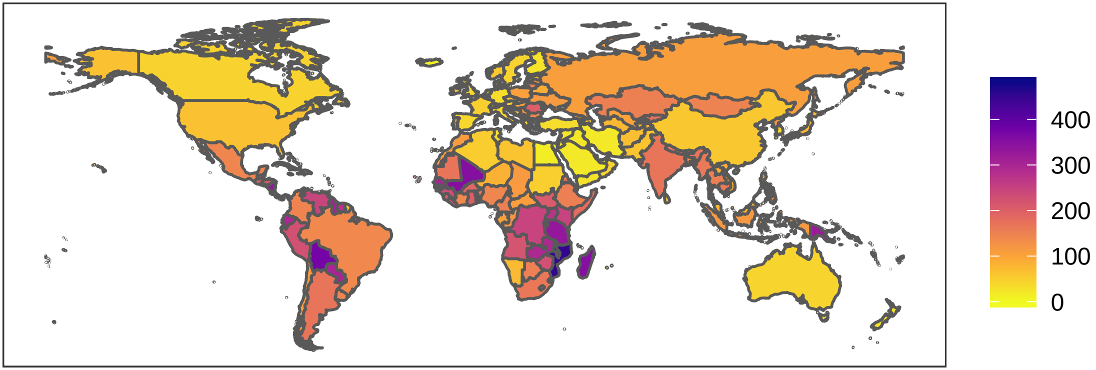

DALYs averted per 1000 vaccinated girls

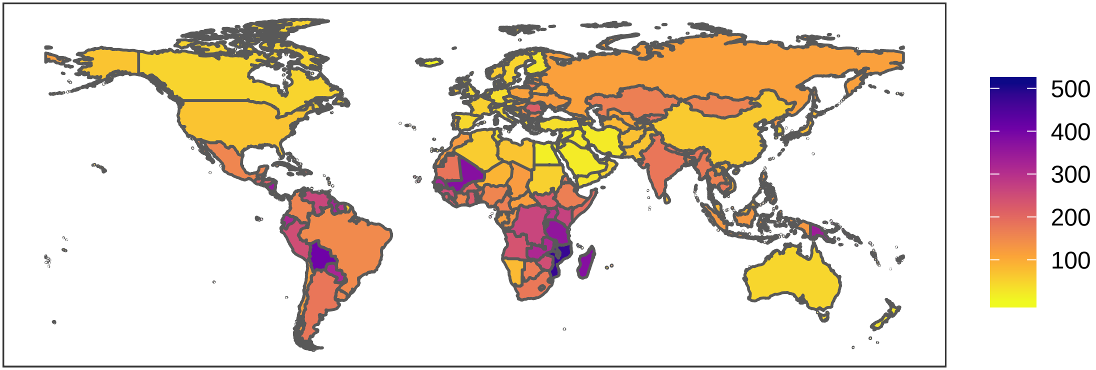

scenario s2 / vaccination age = 12 years / bivalent/quadrivalent vaccine

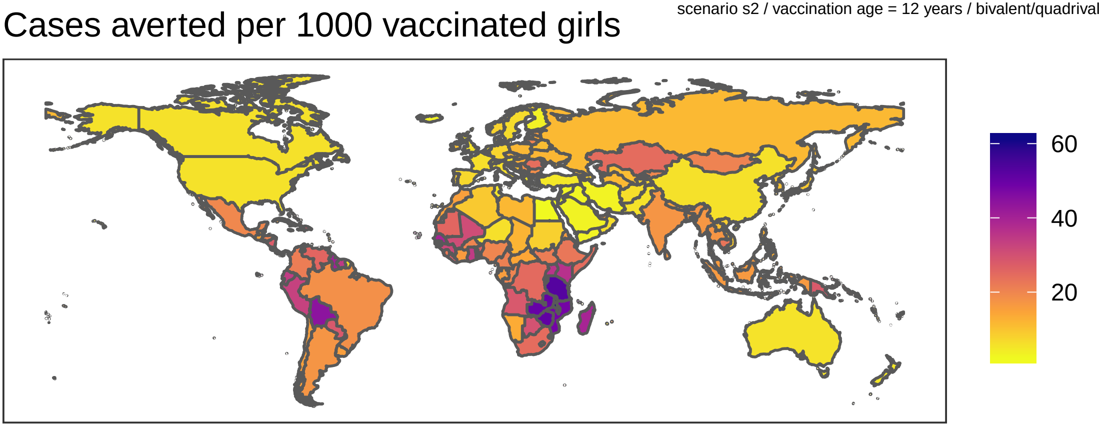

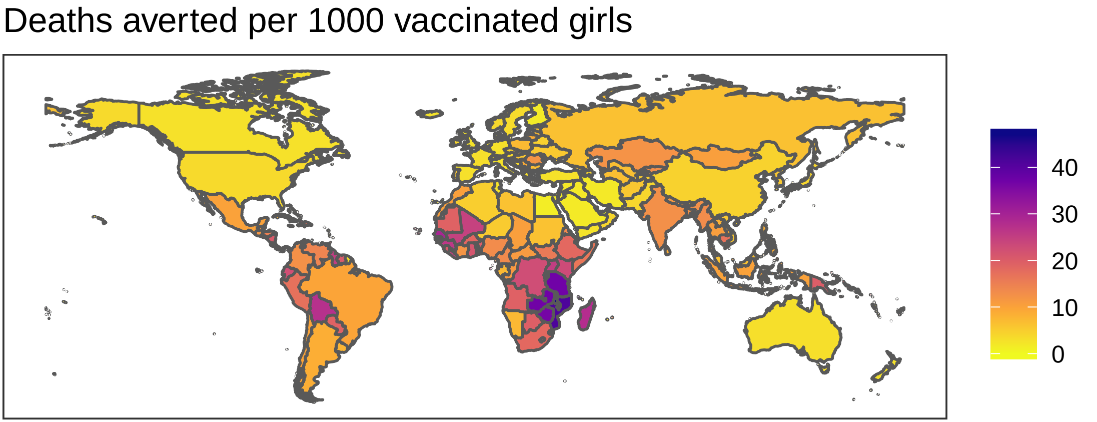

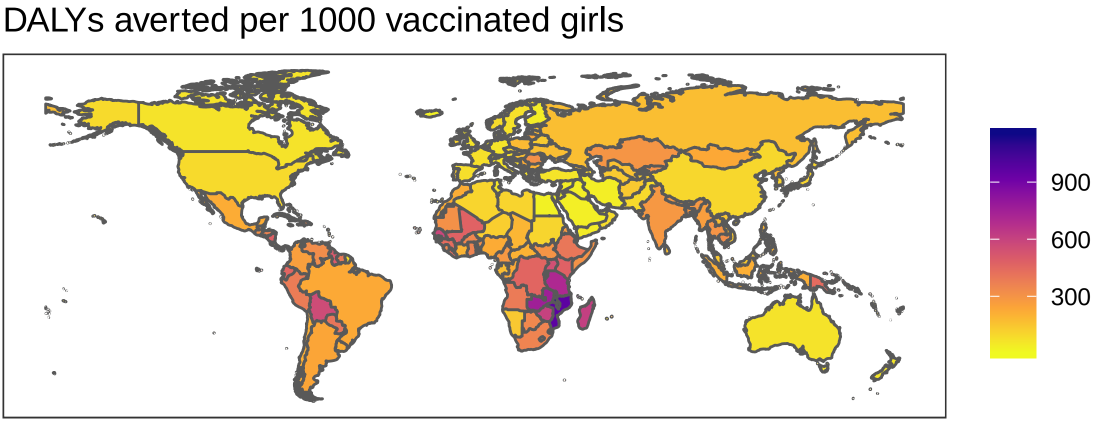

scenario s2 / vaccination age = 12 years / bivalent/quadrivalent vaccine

YLDs averted per 1000 vaccinated girls

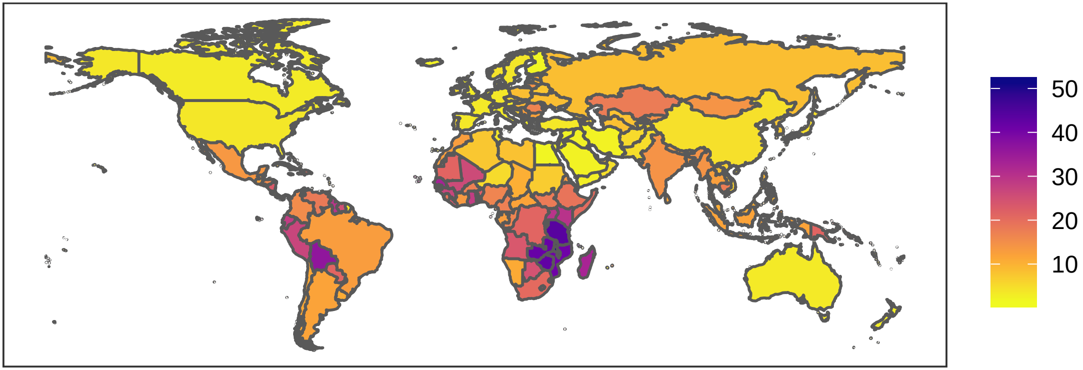

YLLs averted per 1000 vaccinated girls

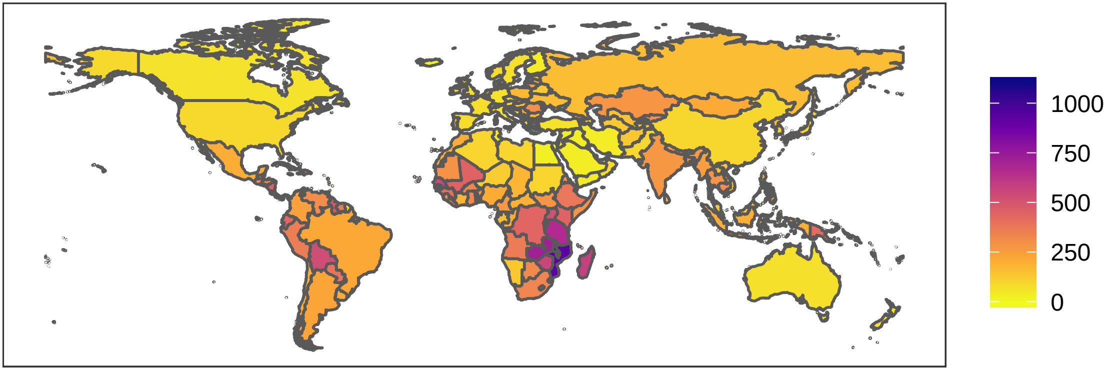

DALYs averted per 1000 vaccinated girls

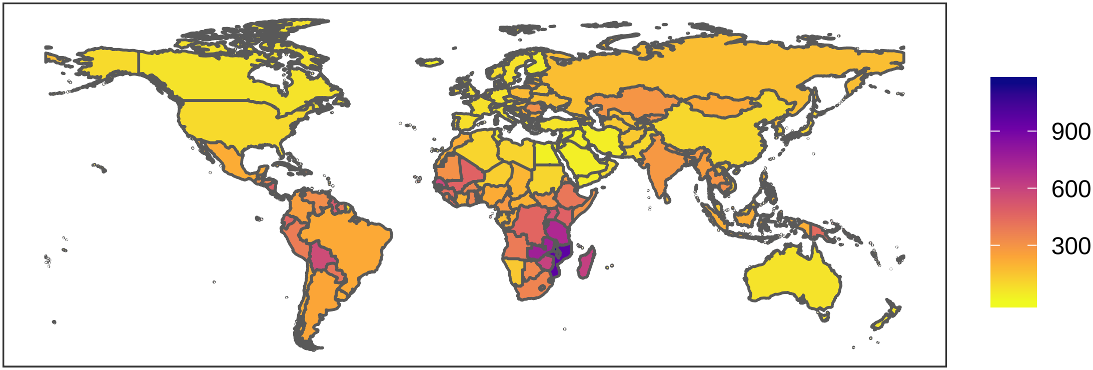

scenario s3 / vaccination age = 12 years / bivalent/quadrivalent vaccine

Cases averted per 1000 vaccinated girls

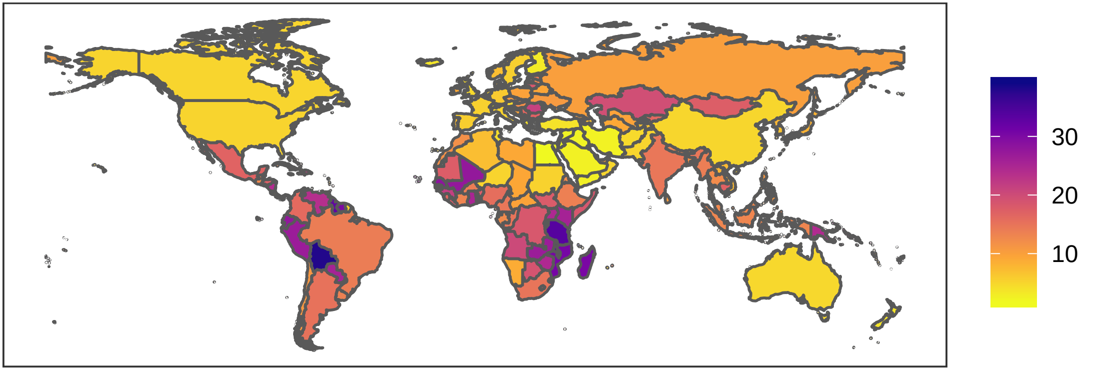

Deaths averted per 1000 vaccinated girls

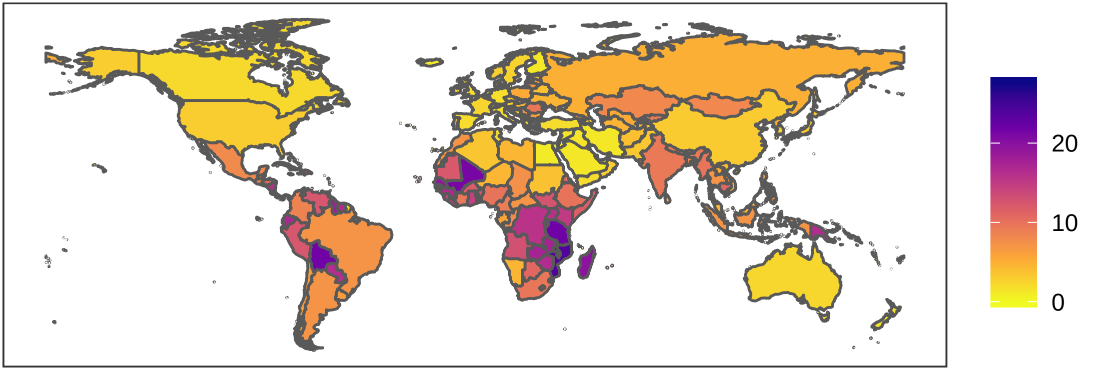

DALYs averted per 1000 vaccinated girls

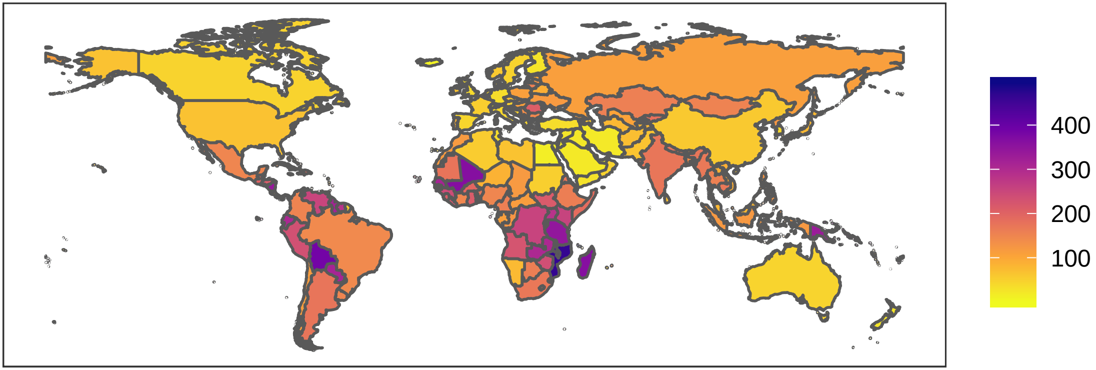

YLDs averted per 1000 vaccinated girls

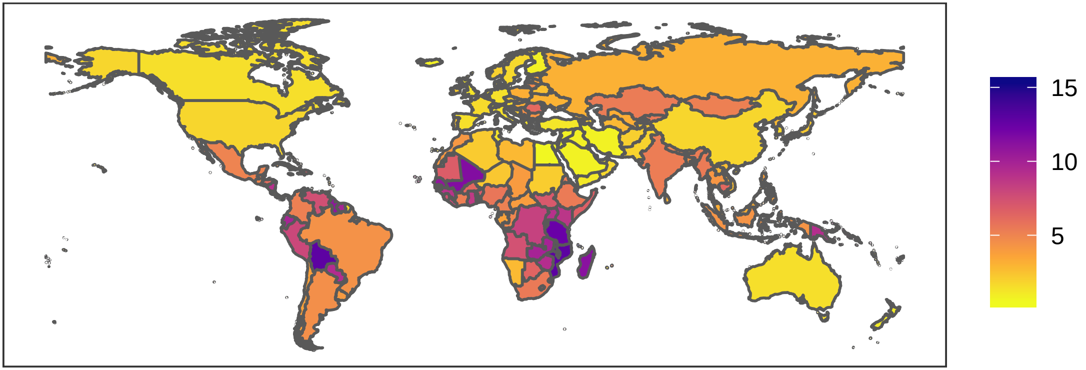

YLLs averted per 1000 vaccinated girls

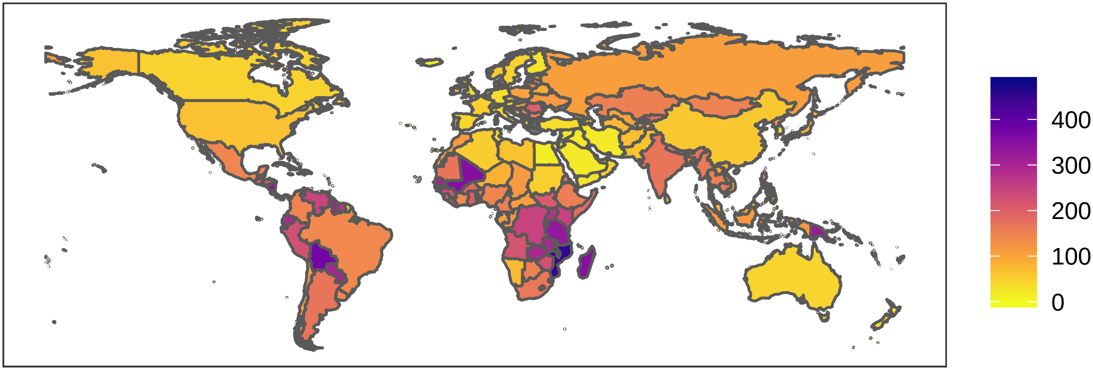

DALYs averted per 1000 vaccinated girls

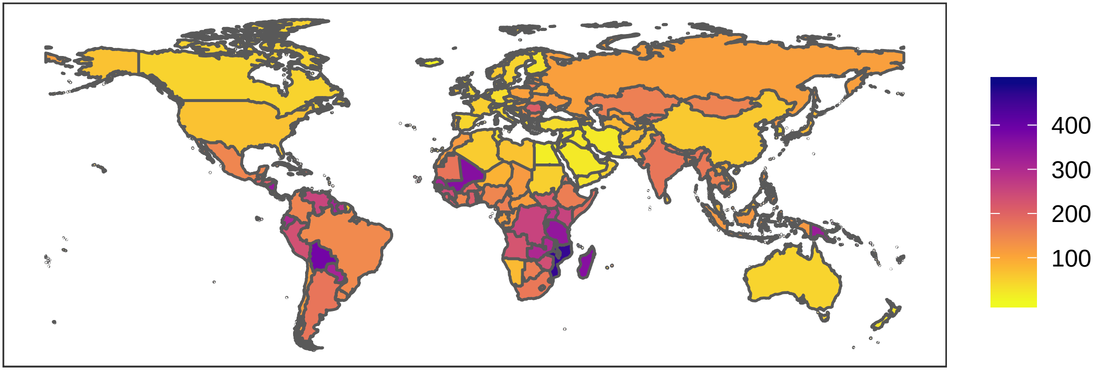

scenario s4 / vaccination age = 12 years / bivalent/quadrivalent vaccine

Cases averted per 1000 vaccinated girls

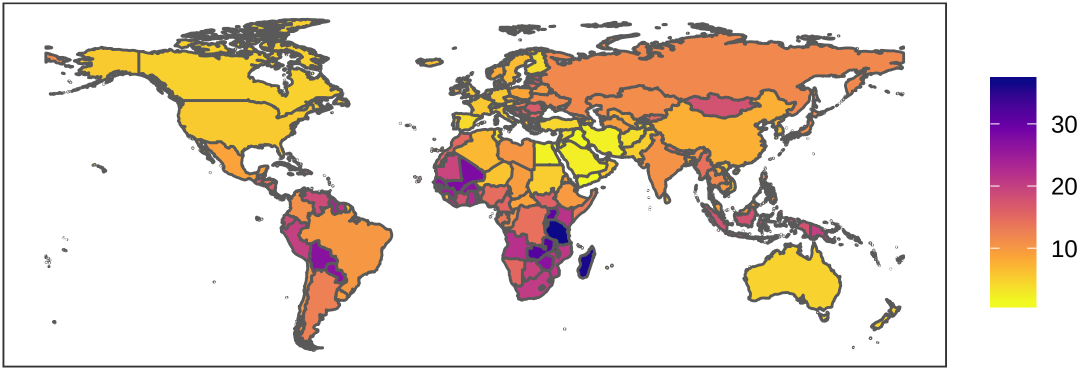

Deaths averted per 1000 vaccinated girls

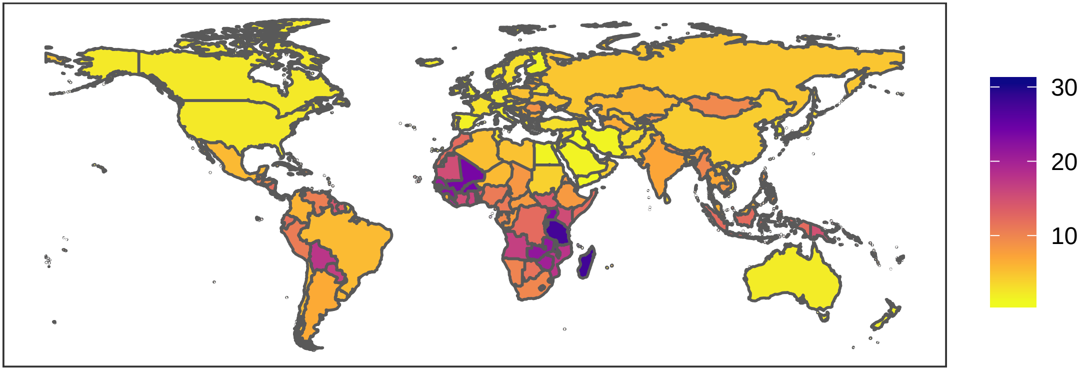

DALYs averted per 1000 vaccinated girls

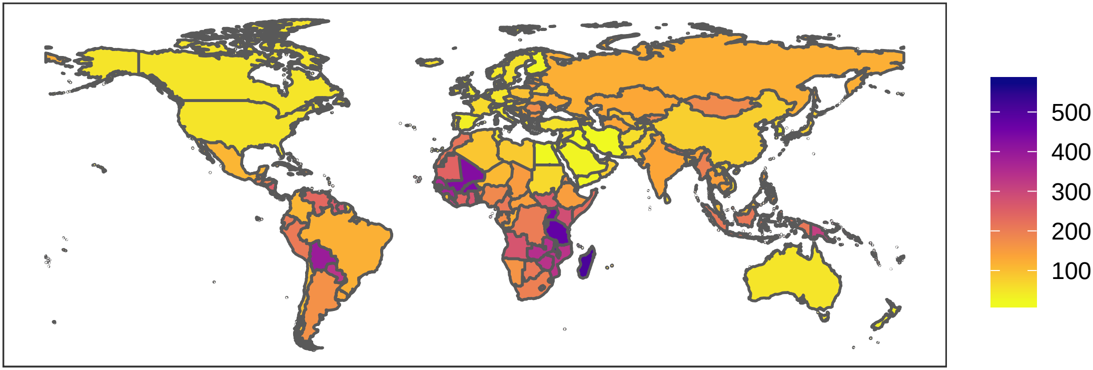

scenario s4 / vaccination age = 12 years / bivalent/quadrivalent vaccine

YLDs averted per 1000 vaccinated girls

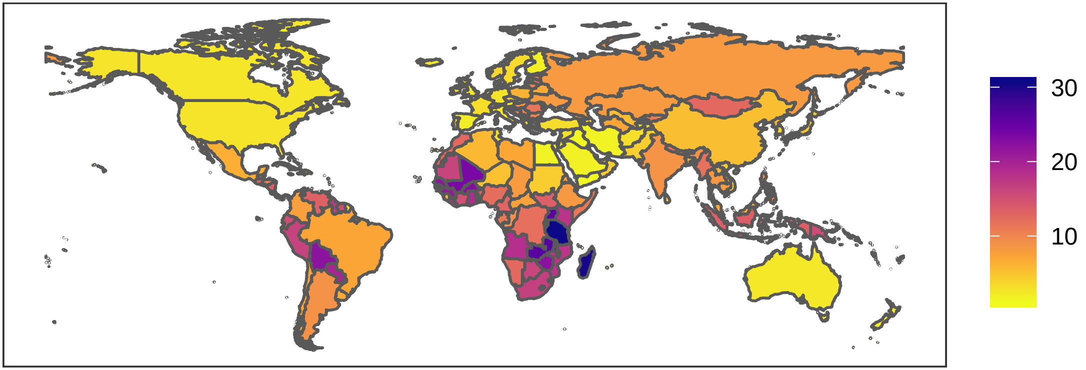

YLLs averted per 1000 vaccinated girls

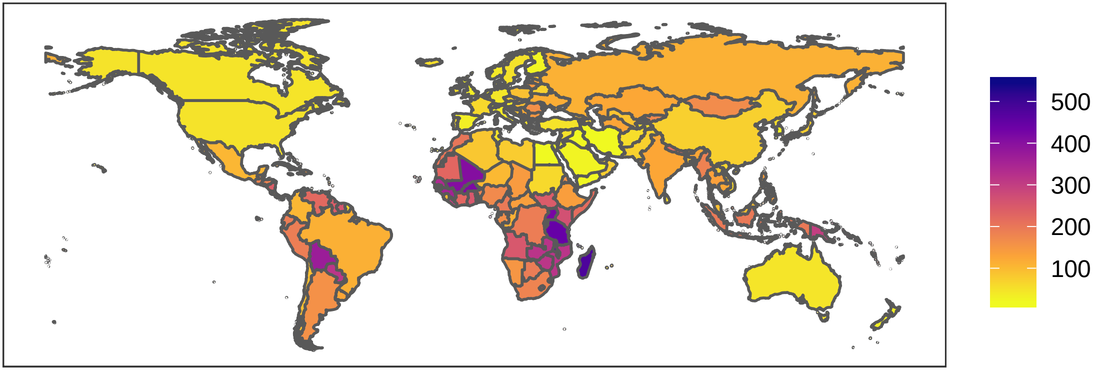

DALYs averted per 1000 vaccinated girls

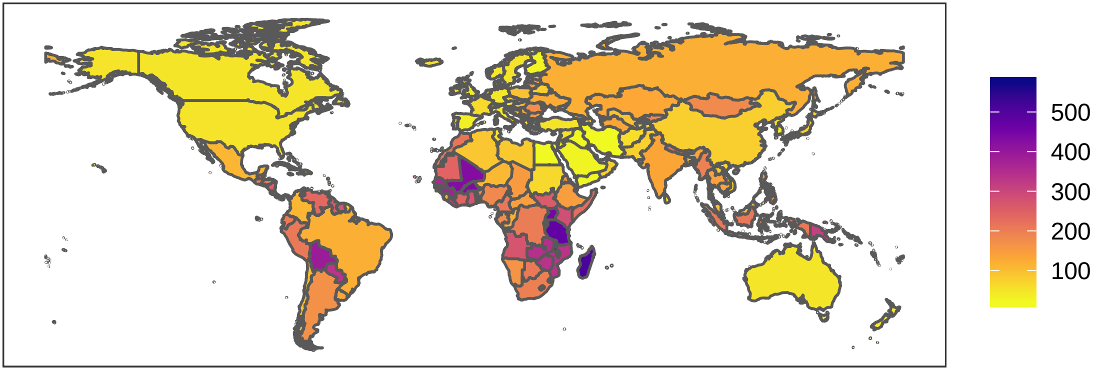

scenario s5 / vaccination age = 12 years / bivalent/quadrivalent vaccine

Cases averted per 1000 vaccinated girls

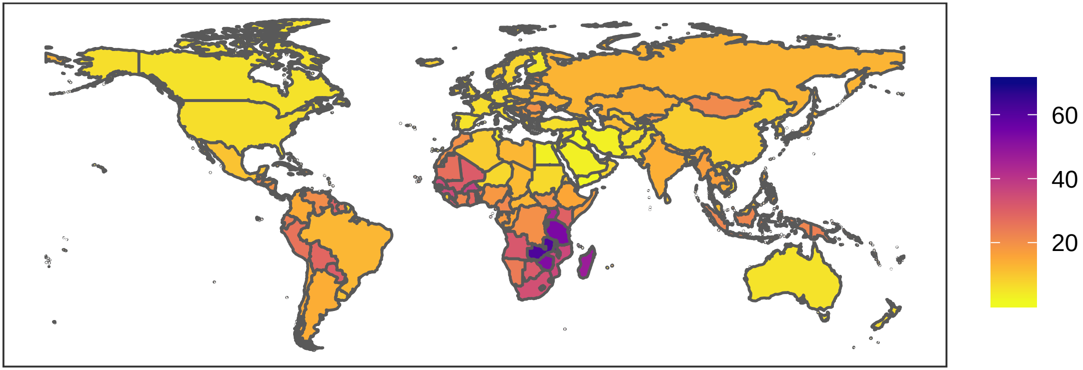

Deaths averted per 1000 vaccinated girls

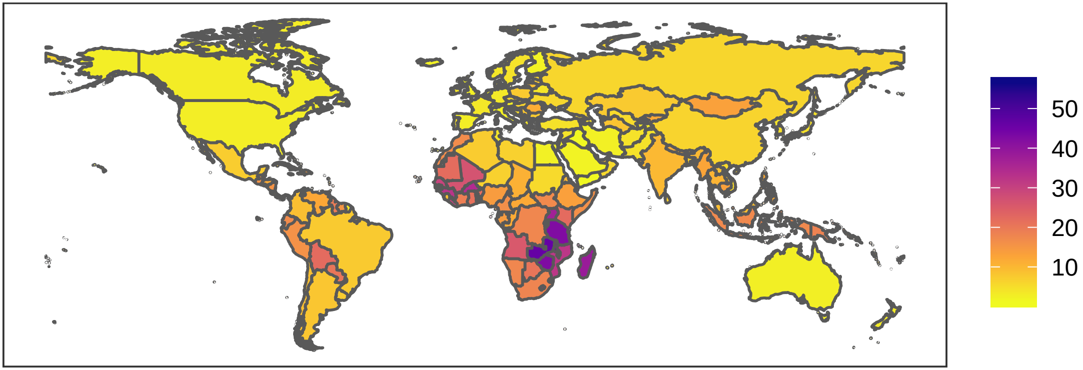

DALYs averted per 1000 vaccinated girls

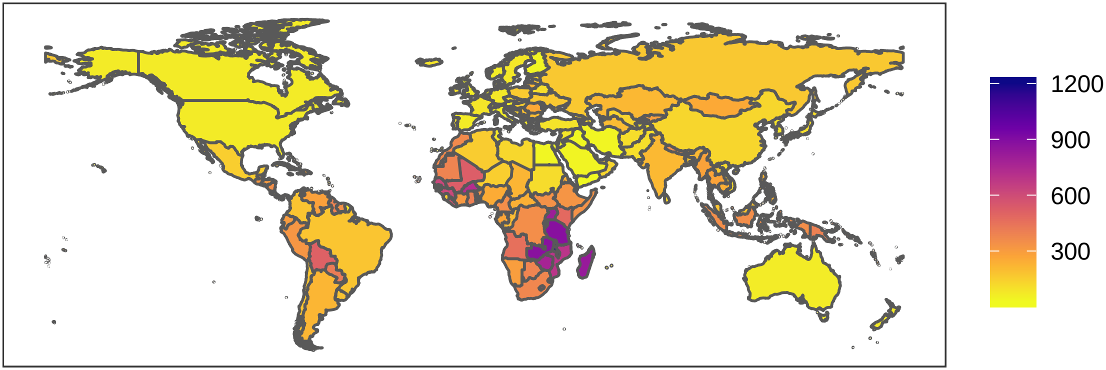

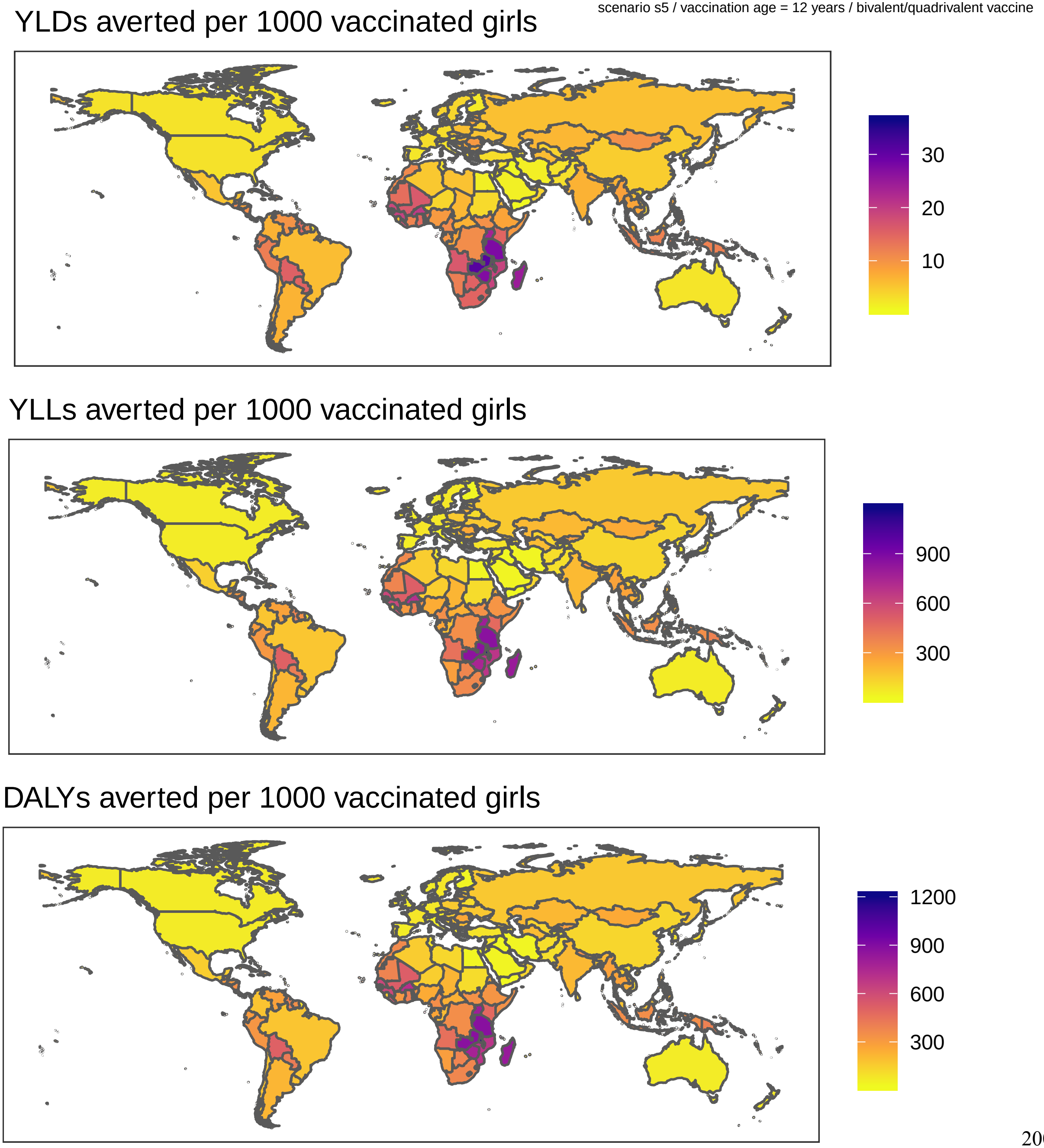
scenario s5 / vaccination age = 12 years / bivalent/quadrivalent vaccine
YLDs averted per 1000 vaccinated girls
30
20
10
YLLs averted per 1000 vaccinated girls
900
600
300
DALYs averted per 1000 vaccinated girls
1200
900
600
300

scenario s1 / vaccination age = 12 years / nonavalent vaccine

Cases averted per 1000 vaccinated girls

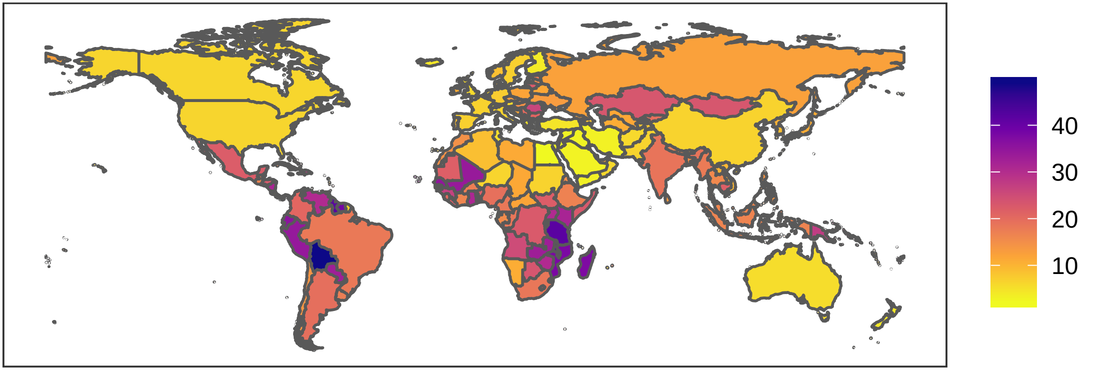

Deaths averted per 1000 vaccinated girls

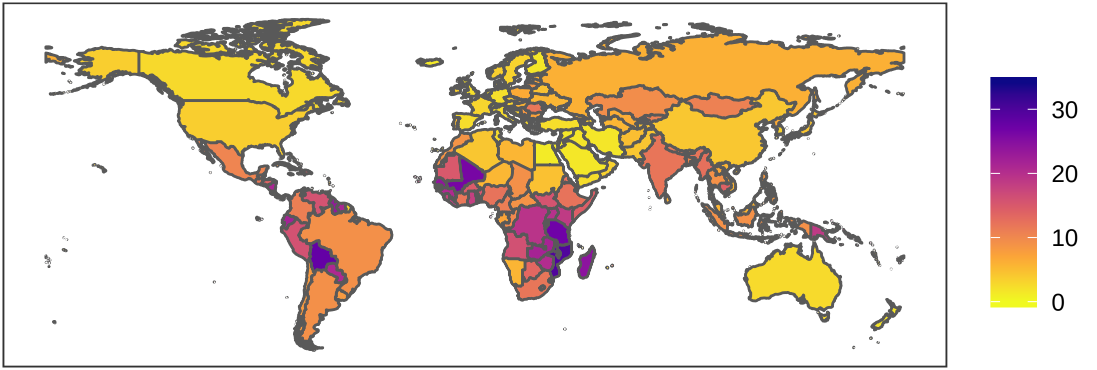

DALYs averted per 1000 vaccinated girls

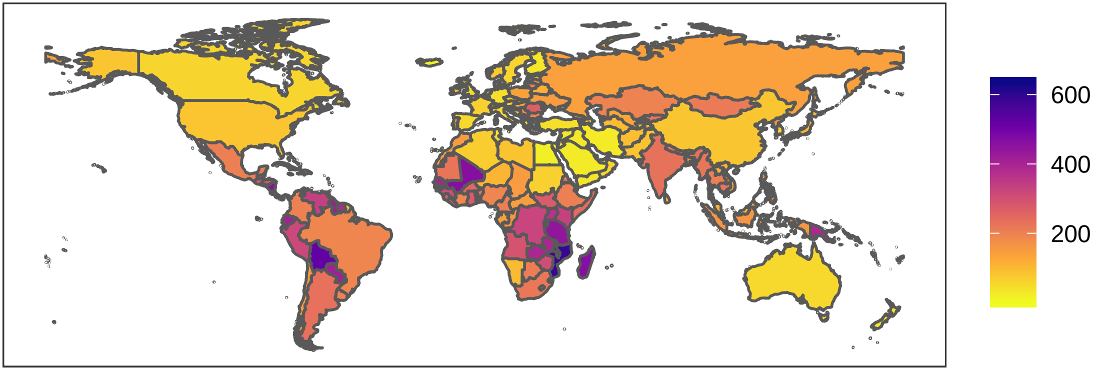

scenario s1 / vaccination age = 12 years / nonavalent vaccine

YLDs averted per 1000 vaccinated girls

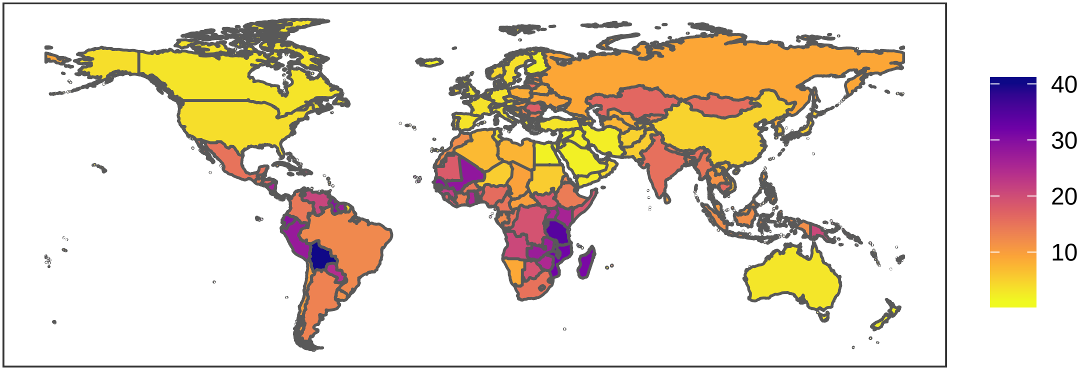

YLLs averted per 1000 vaccinated girls

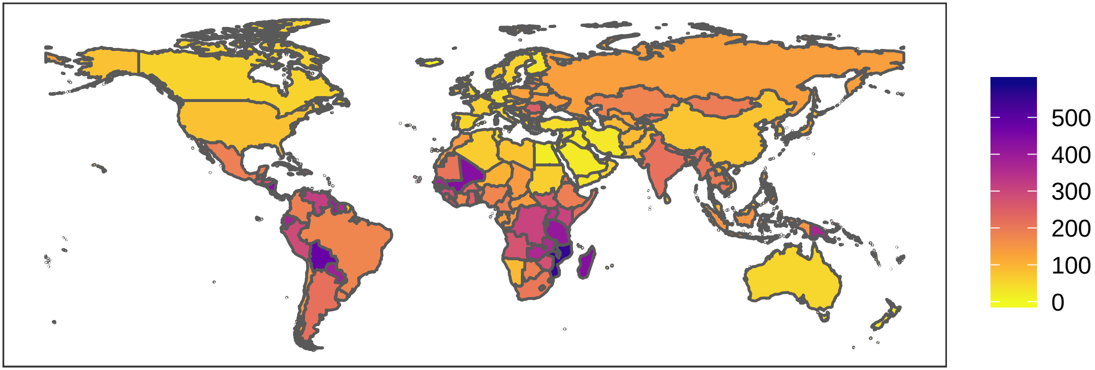

DALYs averted per 1000 vaccinated girls

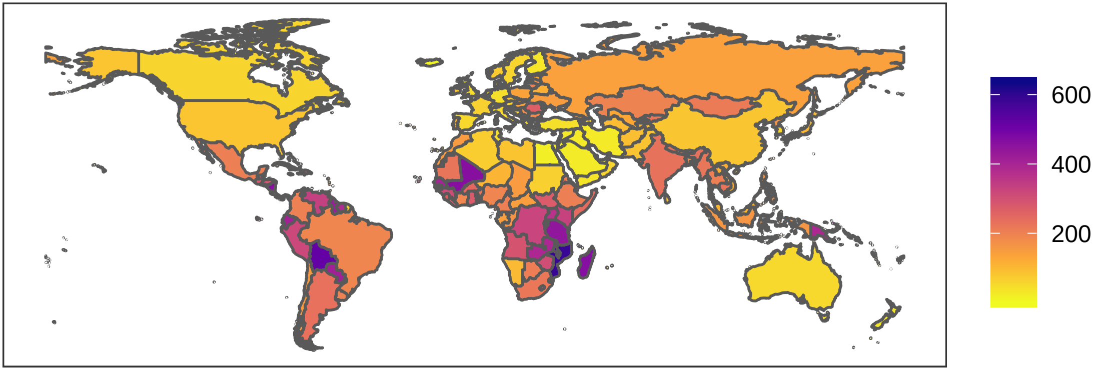

scenario s2 / vaccination age = 12 years / nonavalent vaccine

Cases averted per 1000 vaccinated girls

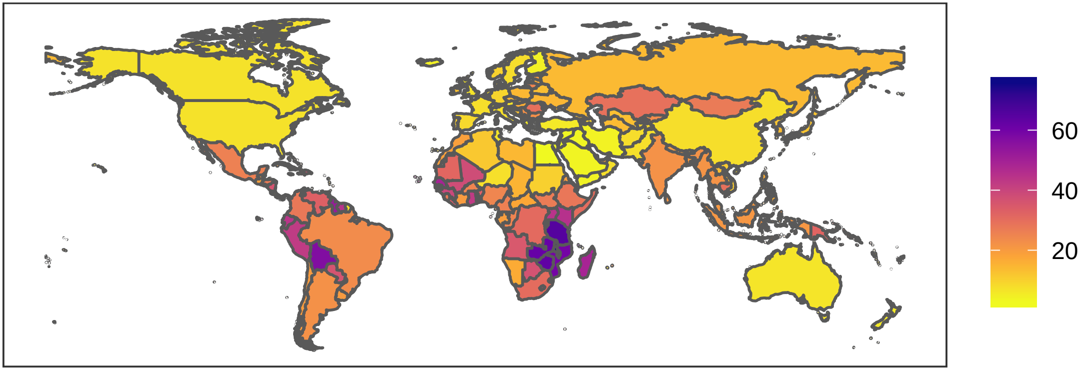

Deaths averted per 1000 vaccinated girls

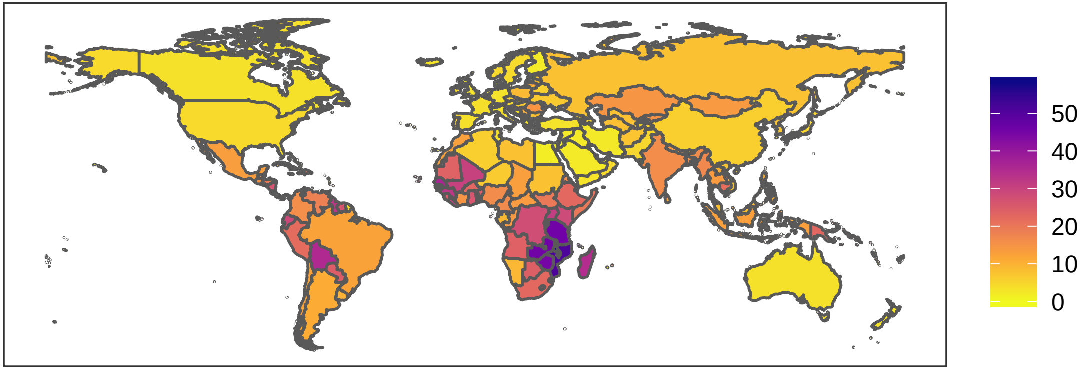

DALYs averted per 1000 vaccinated girls

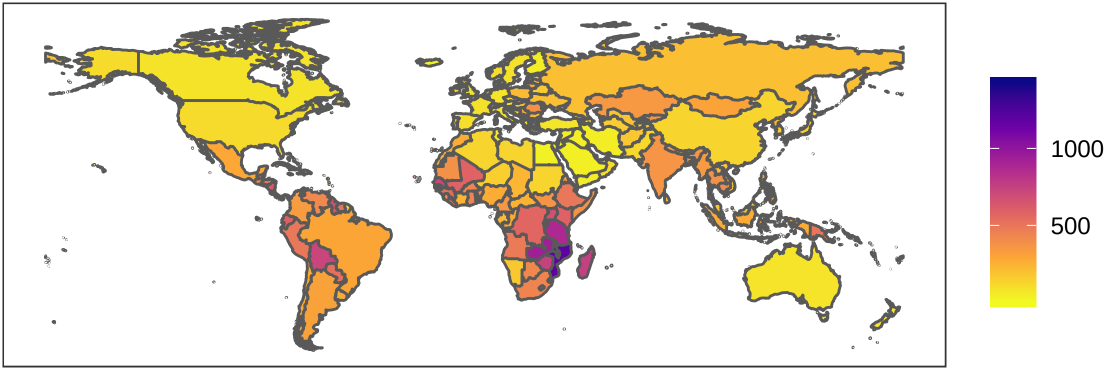

scenario s2 / vaccination age = 12 years / nonavalent vaccine

YLDs averted per 1000 vaccinated girls

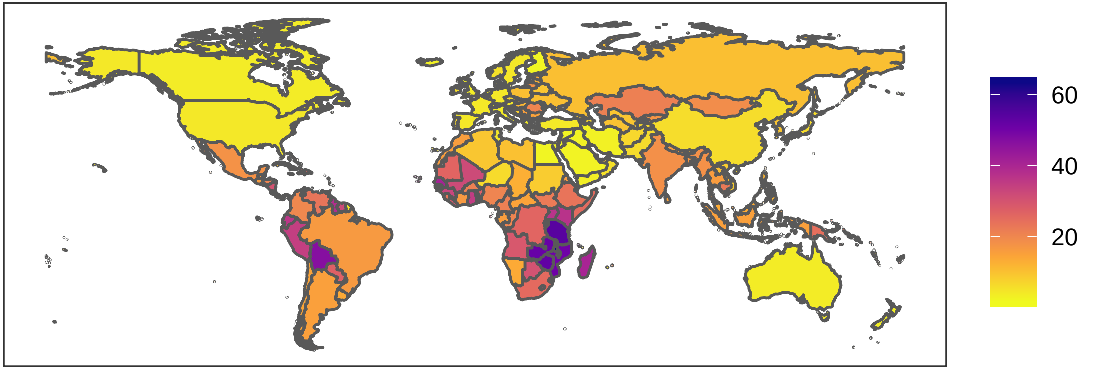

YLLs averted per 1000 vaccinated girls

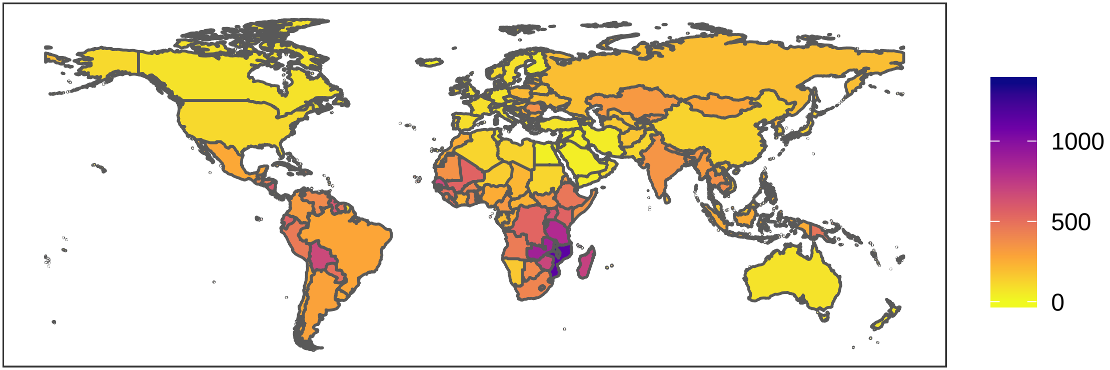

DALYs averted per 1000 vaccinated girls

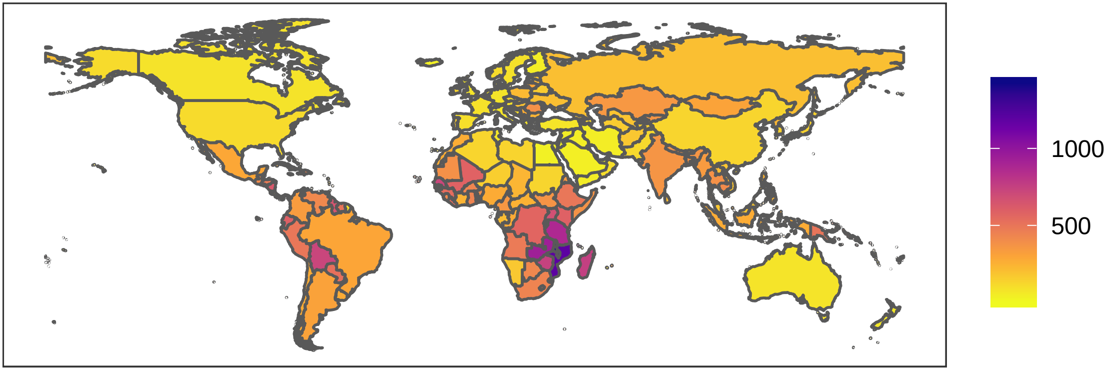

scenario s3 / vaccination age = 12 years / nonavalent vaccine

Cases averted per 1000 vaccinated girls

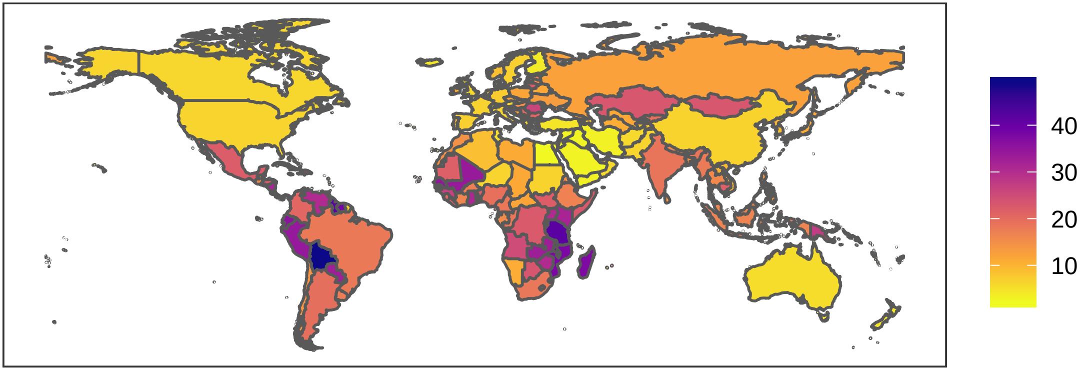

Deaths averted per 1000 vaccinated girls

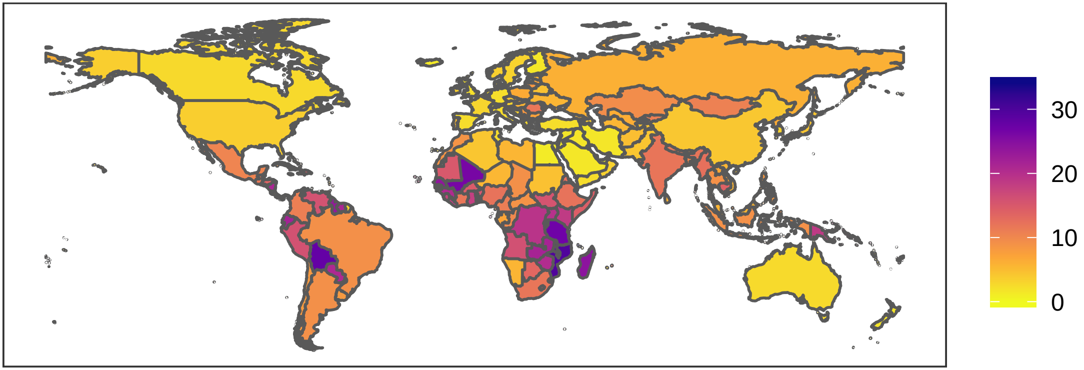

DALYs averted per 1000 vaccinated girls

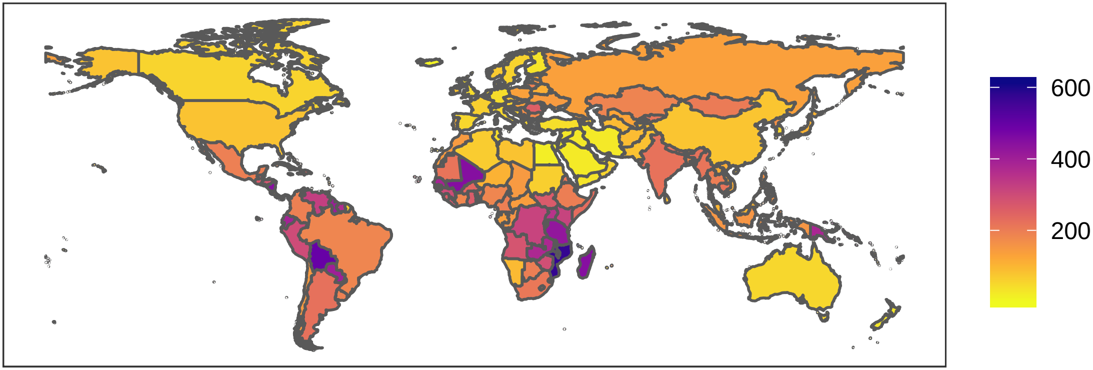

scenario s3 / vaccination age = 12 years / nonavalent vaccine

YLDs averted per 1000 vaccinated girls

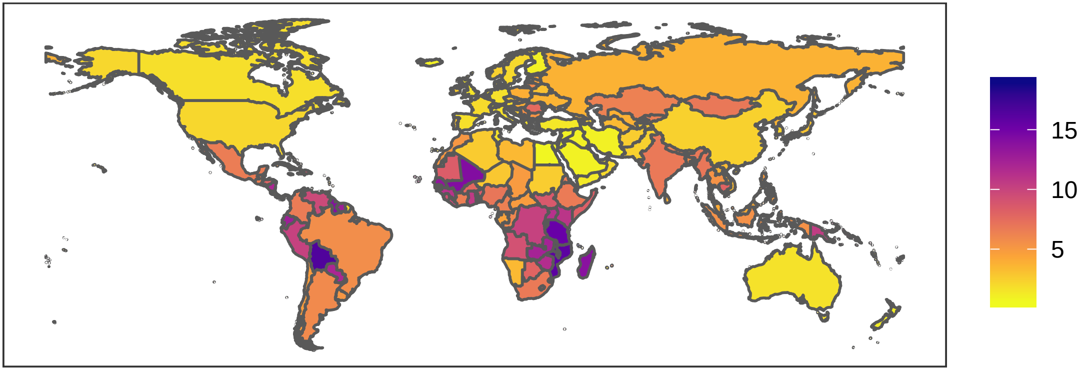

YLLs averted per 1000 vaccinated girls

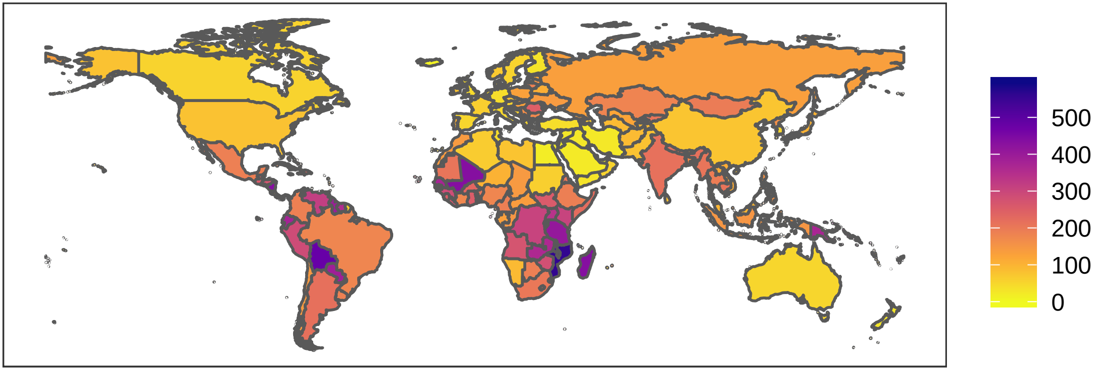

DALYs averted per 1000 vaccinated girls

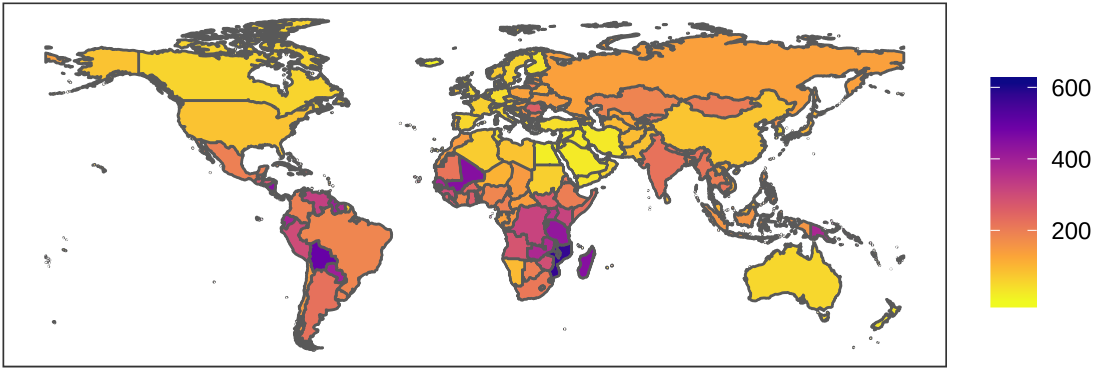

scenario s4 / vaccination age = 12 years / nonavalent vaccine

Cases averted per 1000 vaccinated girls

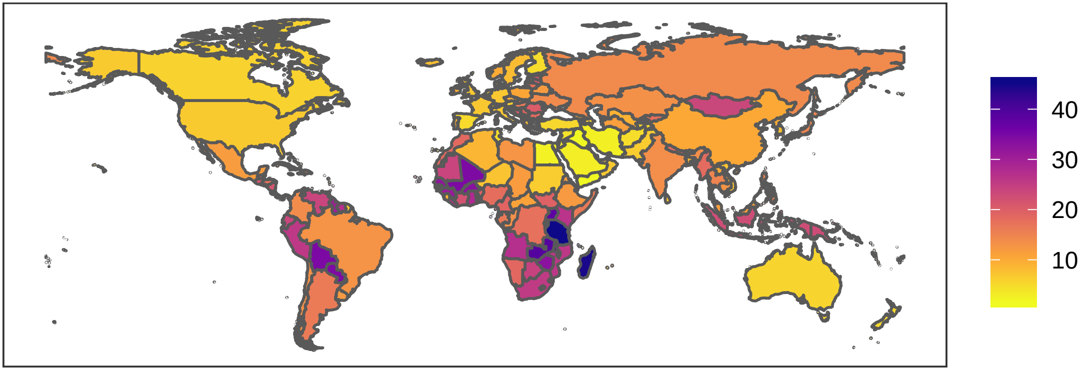

Deaths averted per 1000 vaccinated girls

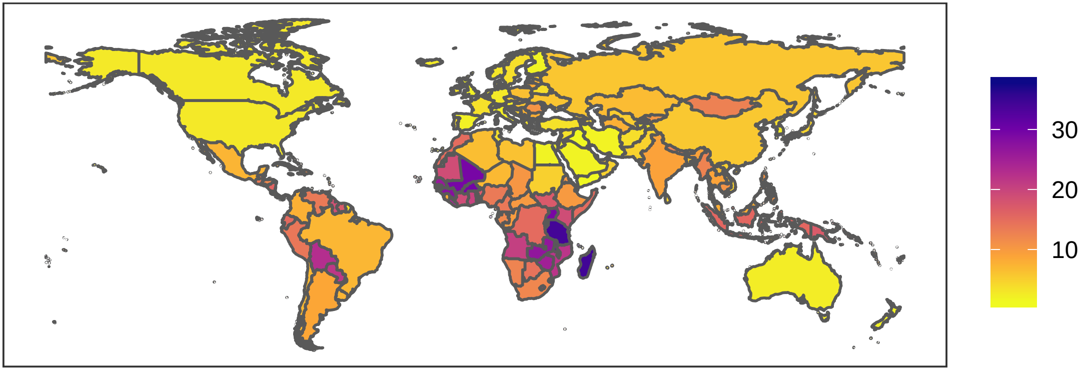

DALYs averted per 1000 vaccinated girls

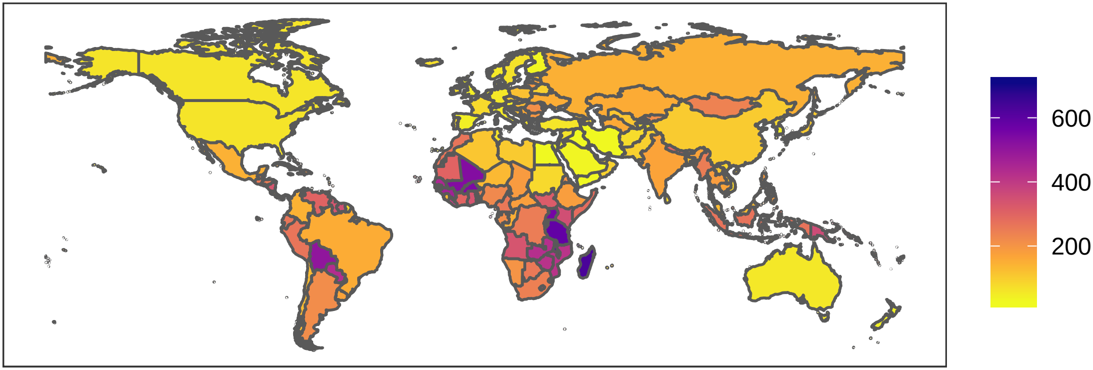

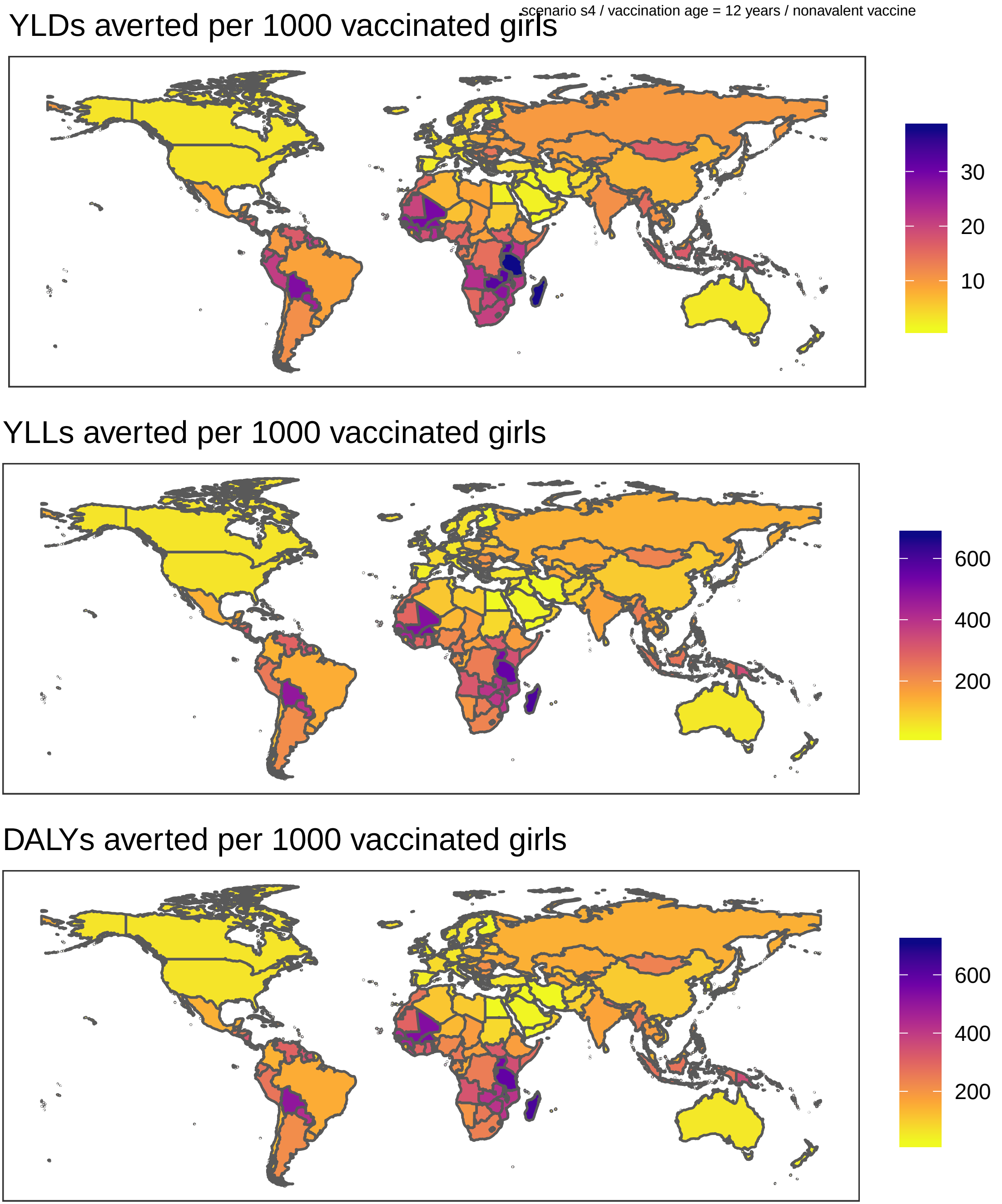

scenario s4 / vaccination age = 12 years / nonavalent vaccine
YLDs averted per 1000 vaccinated girls
30
20
10
YLLs averted per 1000 vaccinated girls
600
400
200
DALYs averted per 1000 vaccinated girls
600
400
200

scenario s5 / vaccination age = 12 years / nonavalent vaccine

Cases averted per 1000 vaccinated girls

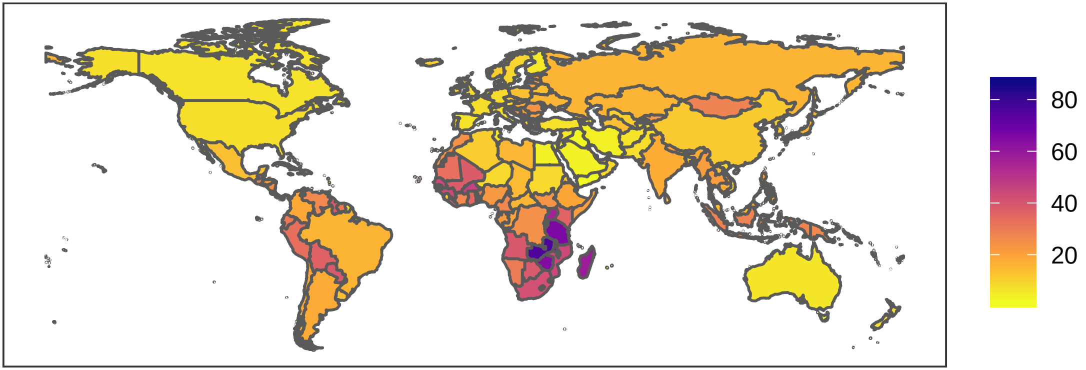

Deaths averted per 1000 vaccinated girls

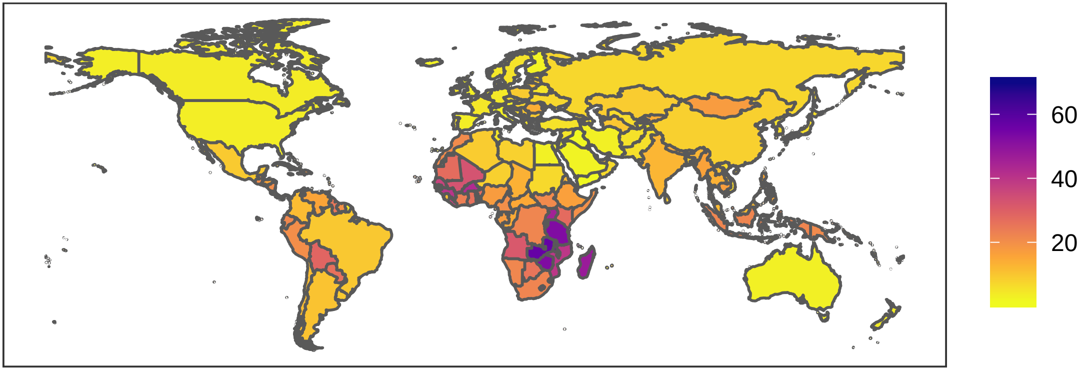

DALYs averted per 1000 vaccinated girls

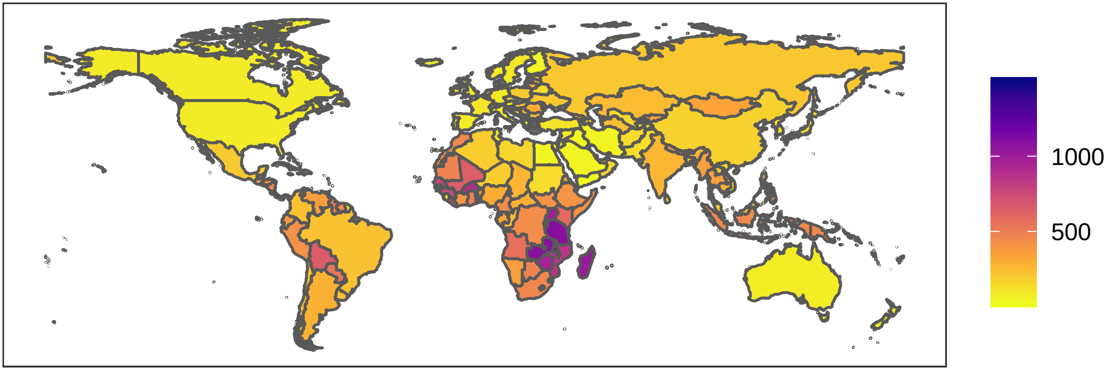

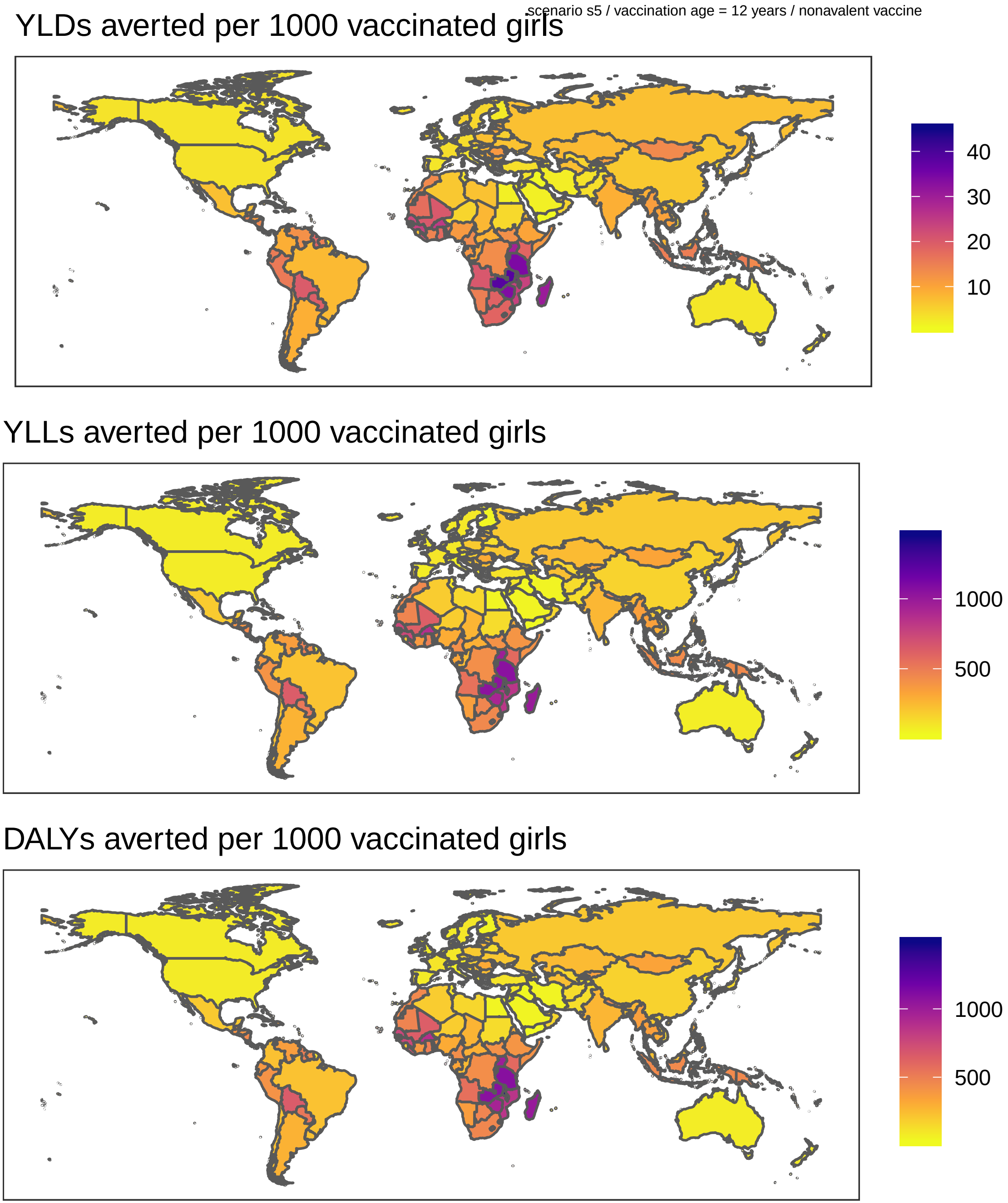
scenario s5 / vaccination age = 12 years / nonavalent vaccine
YLDs averted per 1000 vaccinated girls
40
30
20
10
YLLs averted per 1000 vaccinated girls
1000
500
DALYs averted per 1000 vaccinated girls
1000
500

A16. Vaccination impact per 1000 vaccinated girls at the national level

Lifetime health impact of HPV vaccination on cases, deaths, YLLs, YLDs and DALYs averted per 1000 vaccinated girls in 177 countries for the 5 comparative scenarios for bivalent/quadrivalent and nonavalent vaccination of 9-year-old and 12-year-old girls.

See appendix 2 for data on the lifetime health impact of HPV vaccination.

A17. Vaccination impact per 1000 vaccinated girls at the national level – updated PRIME estimates

Lifetime health impact of HPV vaccination on cases, deaths, YLLs, YLDs and DALYs averted per 1000 vaccinated girls in 177 countries for bivalent/quadrivalent and nonavalent vaccination of 9-year-old and 12-year-old girls (estimates after the combined PRIME updates for demography, disability weights and cervical cancer burden).

Cases averted per 1000 vaccinated girls (vaccination age = 9 years / bivalent/quadrivalent vaccine)

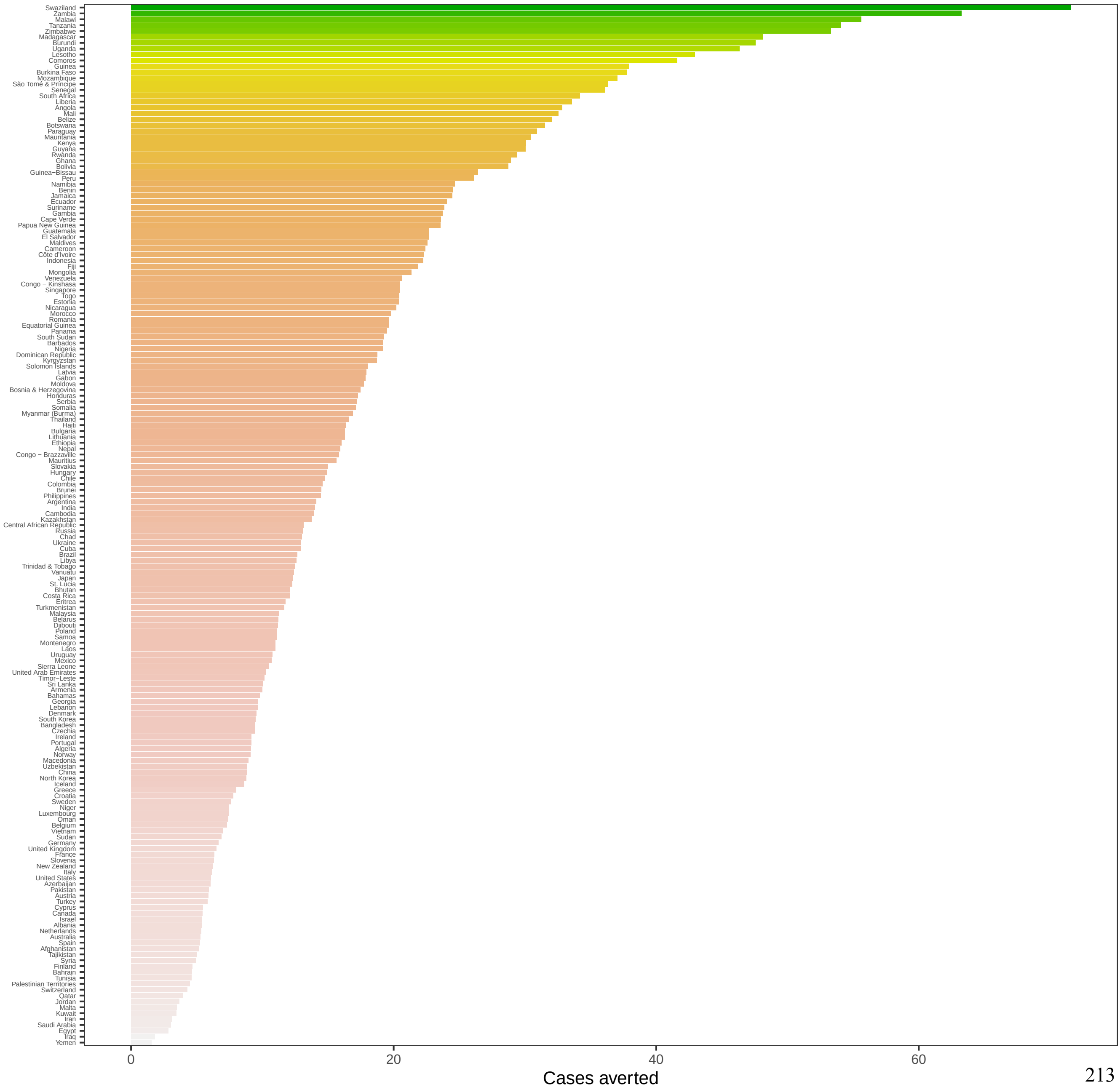

Deaths averted per 1000 vaccinated girls (vaccination age = 9 years / bivalent/quadrivalent vaccine)

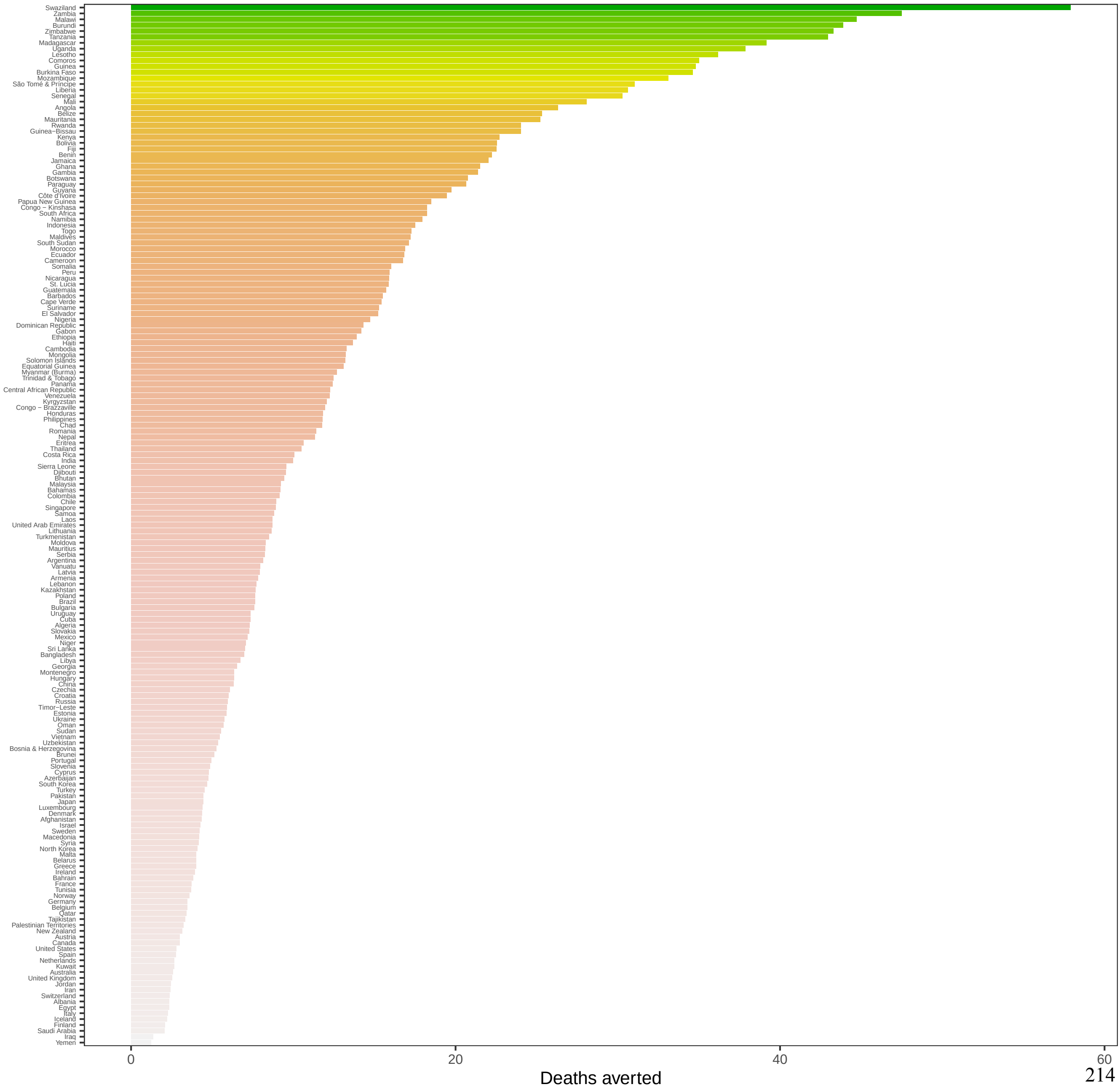

YLDs averted per 1000 vaccinated girls (vaccination age = 9 years / bivalent/quadrivalent vaccine)

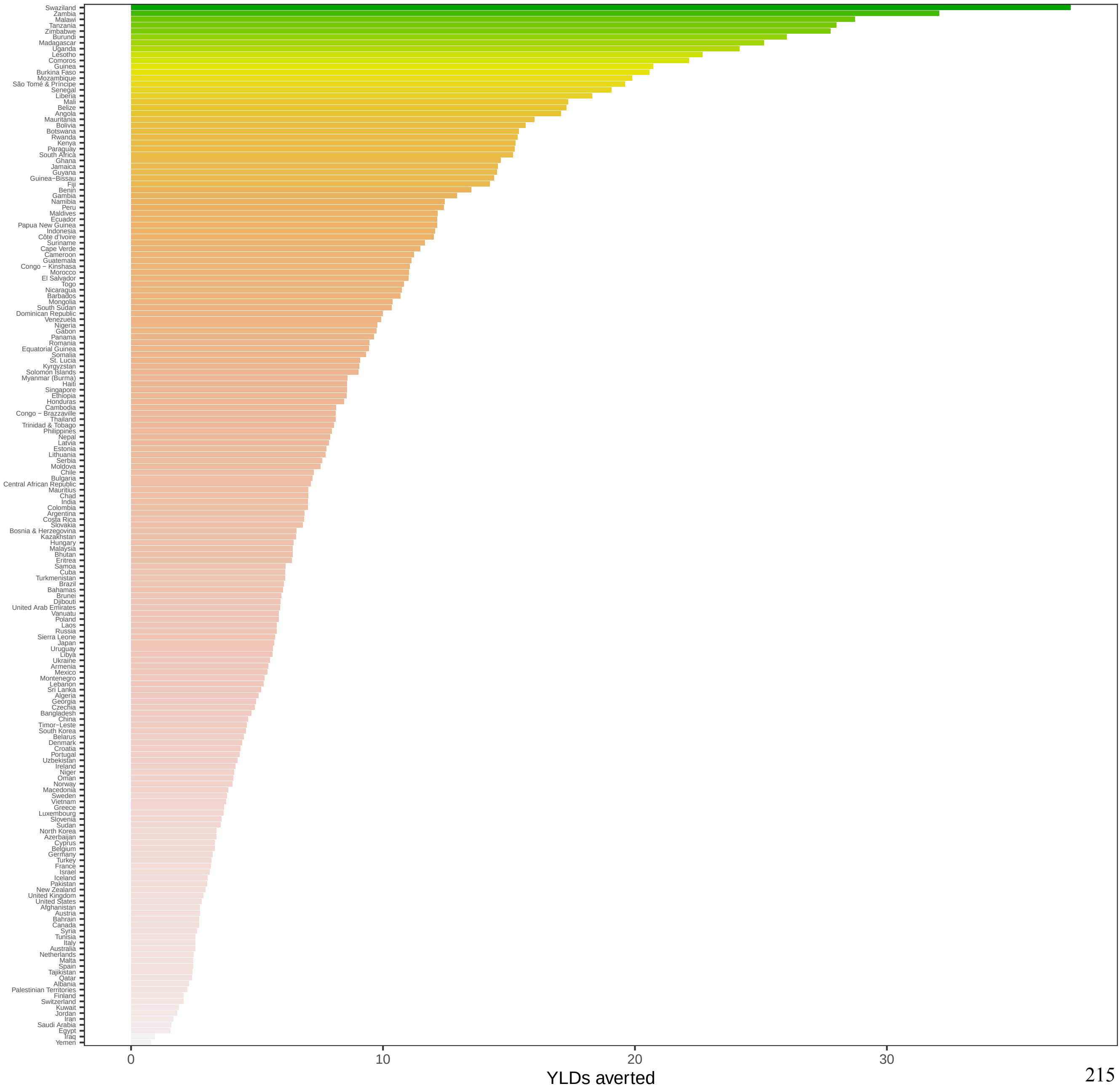

YLLs averted per 1000 vaccinated girls (vaccination age = 9 years / bivalent/quadrivalent vaccine)

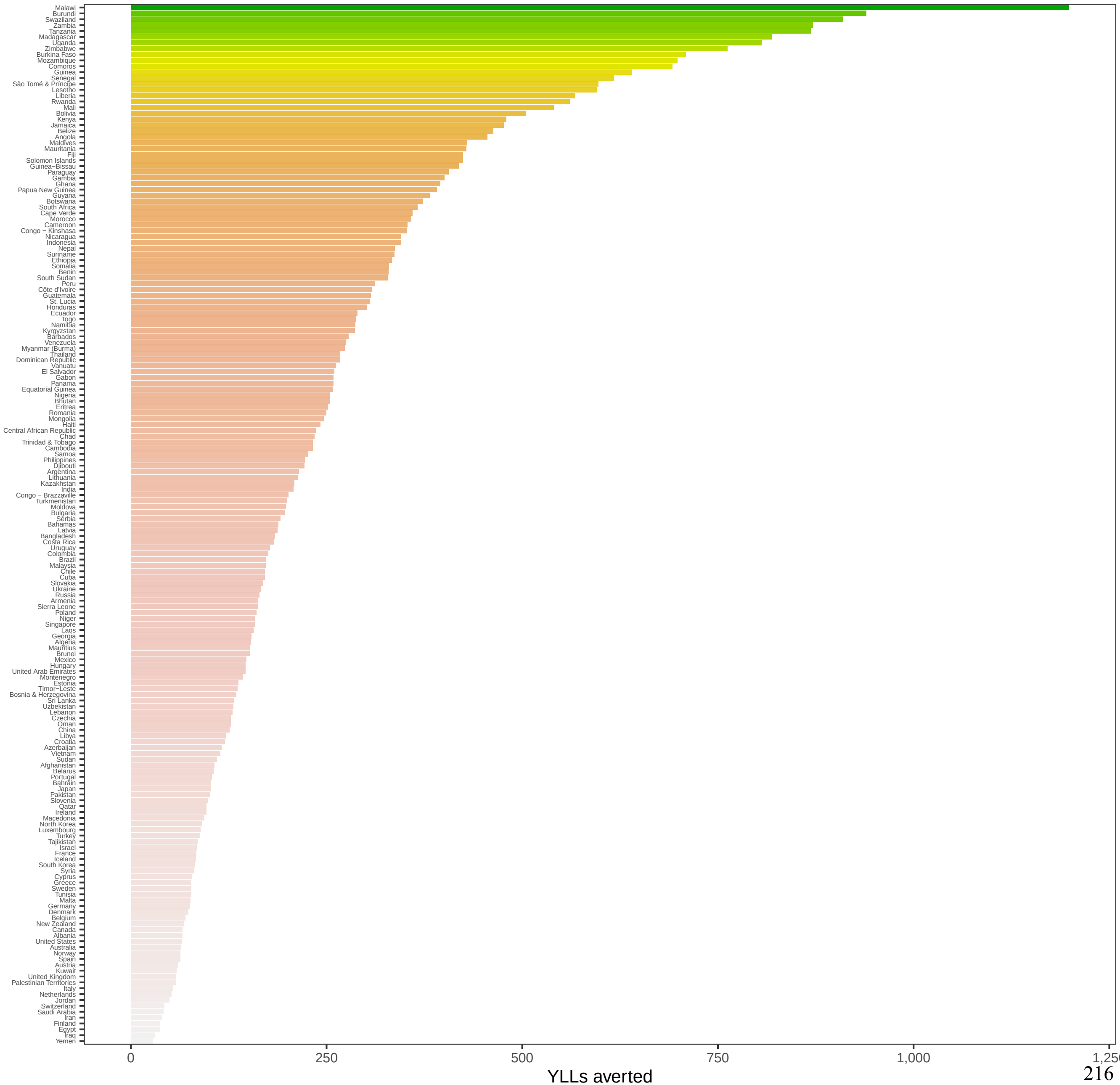

DALYs averted per 1000 vaccinated girls (vaccination age = 9 years / bivalent/quadrivalent vaccine)

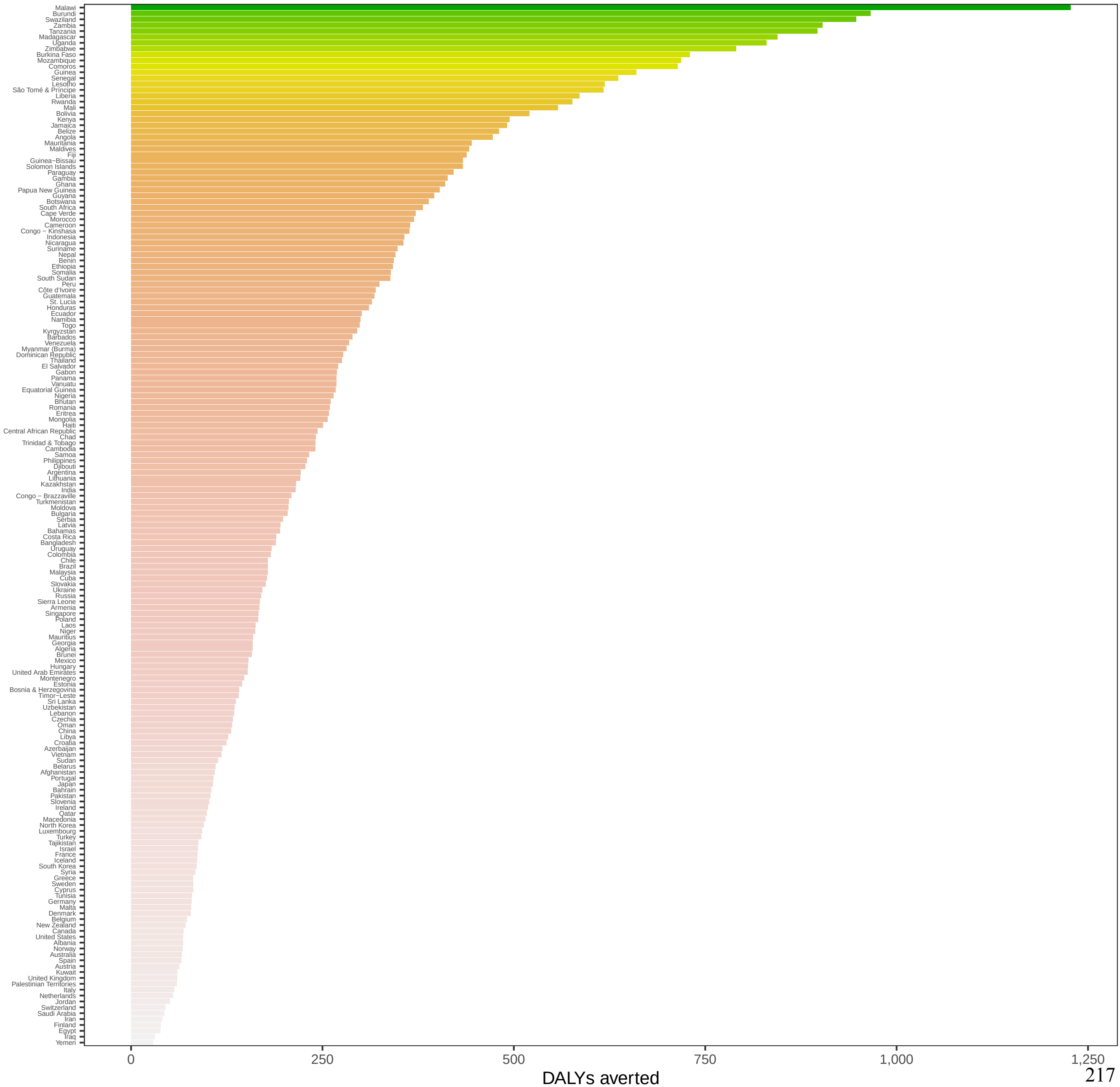

Cases averted per 1000 vaccinated girls (vaccination age = 9 years / nonavalent vaccine)

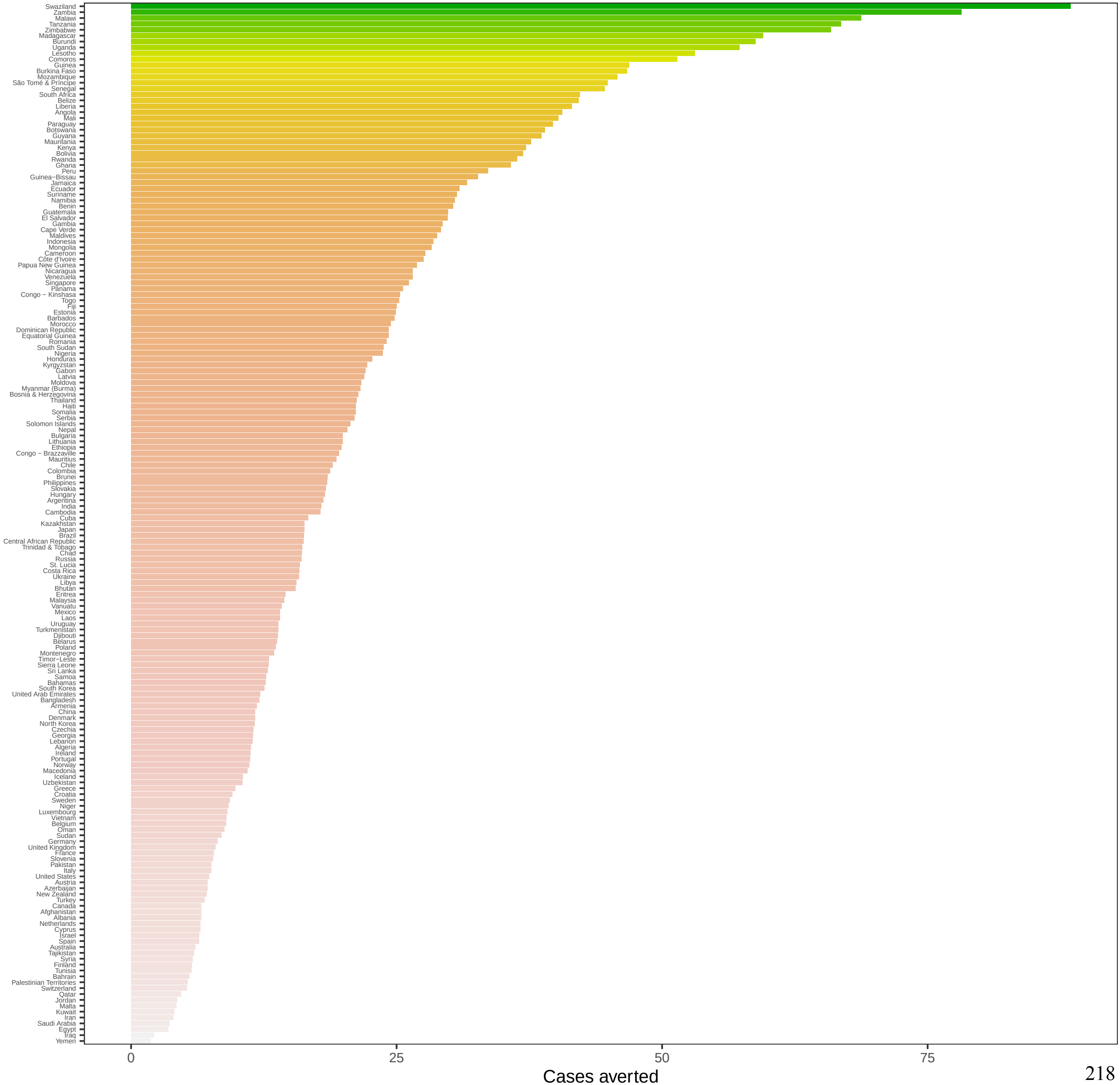

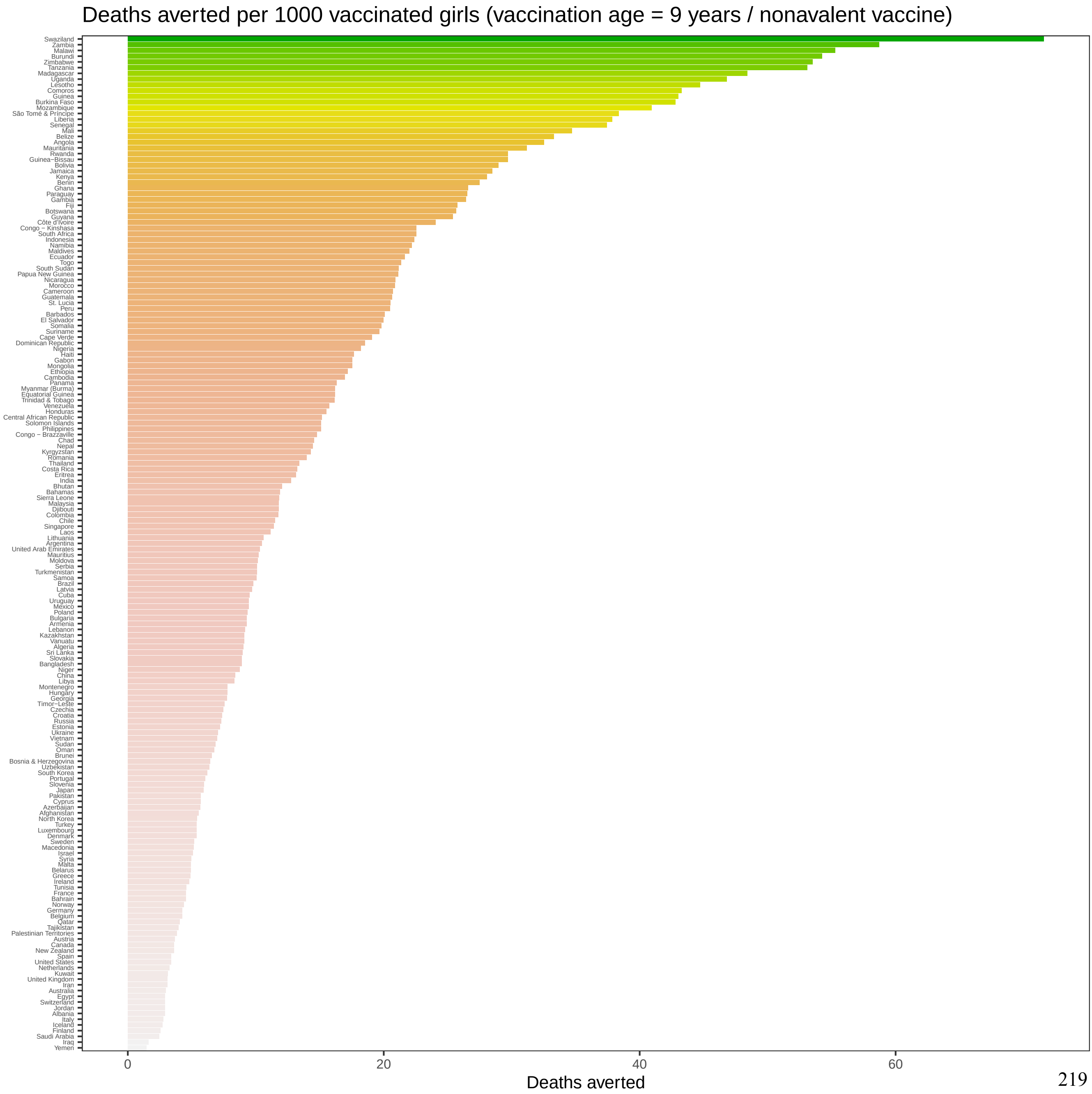

Deaths averted per 1000 vaccinated girls (vaccination age = 9 years / nonavalent vaccine)
Swaziland
Zambia
Malawi
Burundi
Zimbabwe
Tanzania
Madagascar
Uganda
Lesotho
Comoros
Guinea
Burkina Faso
Mozambique
São Tomé & Príncipe
Liberia
Senegal
Mali
Belize
Angola
Mauritania
Rwanda
Guinea−Bissau
Bolivia
Jamaica
Kenya
Benin
Ghana
Paraguay
Gambia
Fiji
Botswana
Guyana
Côte d'Ivoire
Congo − Kinshasa
South Africa
Indonesia
Namibia
Maldives
Ecuador
Togo
South Sudan
Papua New Guinea
Nicaragua
Morocco
Cameroon
Guatemala
St. Lucia
Peru
Barbados
El Salvador
Somalia
Suriname
Cape Verde
Dominican Republic
Nigeria
Haiti
Gabon
Mongolia
Ethiopia
Cambodia
Panama
Myanmar (Burma)
Equatorial Guinea
Trinidad & Tobago
Venezuela
Honduras
Central African Republic
Solomon Islands
Philippines
Congo − Brazzaville
Chad
Nepal
Kyrgyzstan
Romania
Thailand
Costa Rica
Eritrea
India
Bhutan
Bahamas
Sierra Leone
Malaysia
Djibouti
Colombia
Chile
Singapore
Laos
Lithuania
Argentina
United Arab Emirates
Mauritius
Moldova
Serbia
Turkmenistan
Samoa
Brazil
Latvia
Cuba
Uruguay
Mexico
Poland
Bulgaria
Armenia
Lebanon
Kazakhstan
Vanuatu
Algeria
Sri Lanka
Slovakia
Bangladesh
Niger
China
Libya
Montenegro
Hungary
Georgia
Timor−Leste
Czechia
Croatia
Russia
Estonia
Ukraine
Vietnam
Sudan
Oman
Brunei
Bosnia & Herzegovina
Uzbekistan
South Korea
Portugal
Slovenia
Japan
Pakistan
Cyprus
Azerbaijan
Afghanistan
North Korea
Turkey
Luxembourg
Denmark
Sweden
Macedonia
Israel
Syria
Malta
Belarus
Greece
Ireland
Tunisia
France
Bahrain
Norway
Germany
Belgium
Qatar
Tajikistan
Palestinian Territories
Austria
Canada
New Zealand
Spain
United States
Netherlands
Kuwait
United Kingdom
Iran
Australia
Egypt
Switzerland
Jordan
Albania
Italy
Iceland
Finland
Saudi Arabia
Iraq
Yemen
0
20
40
60
Deaths averted

YLDs averted per 1000 vaccinated girls (vaccination age = 9 years / nonavalent vaccine)

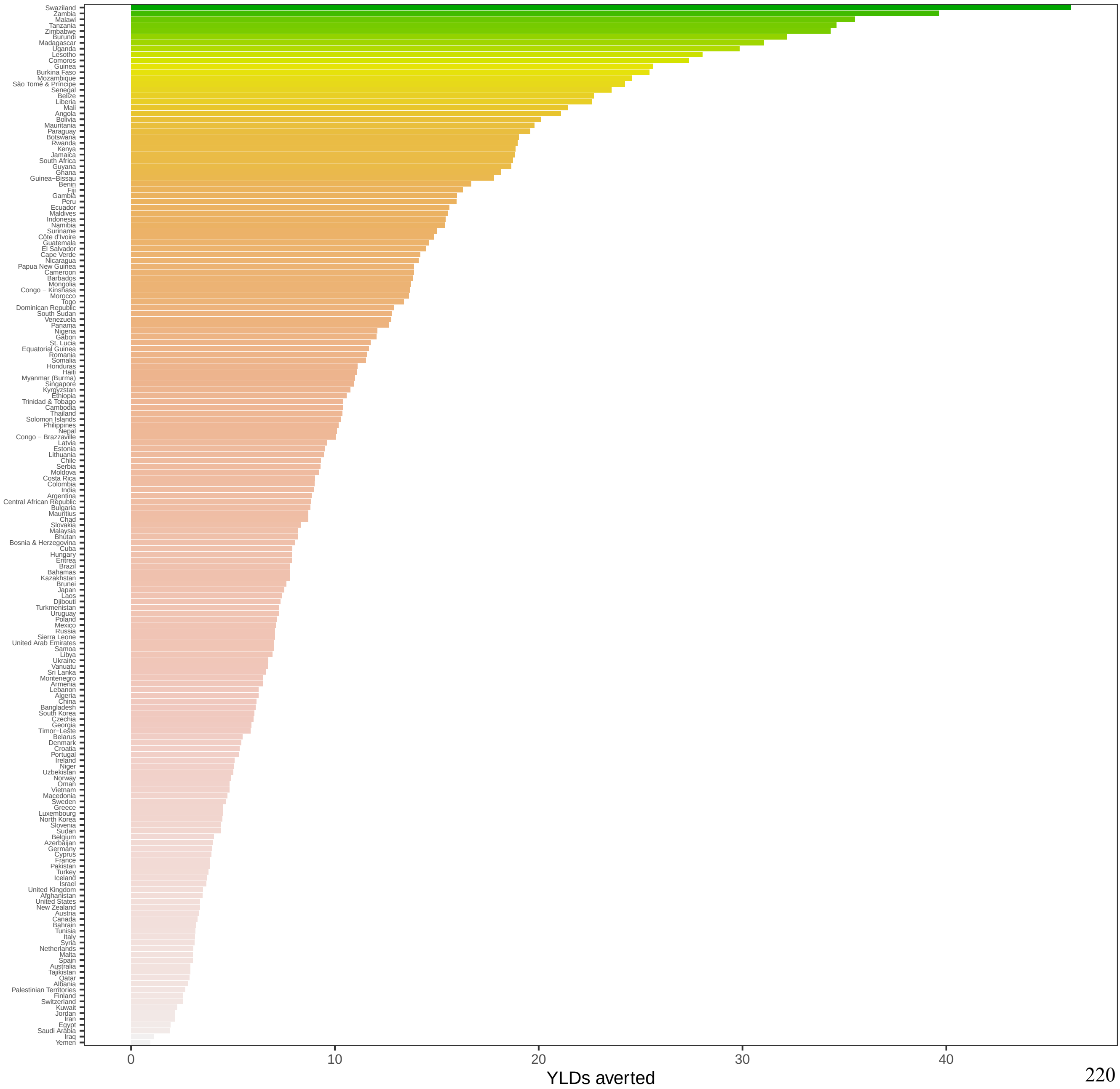

YLLs averted per 1000 vaccinated girls (vaccination age = 9 years / nonavalent vaccine)

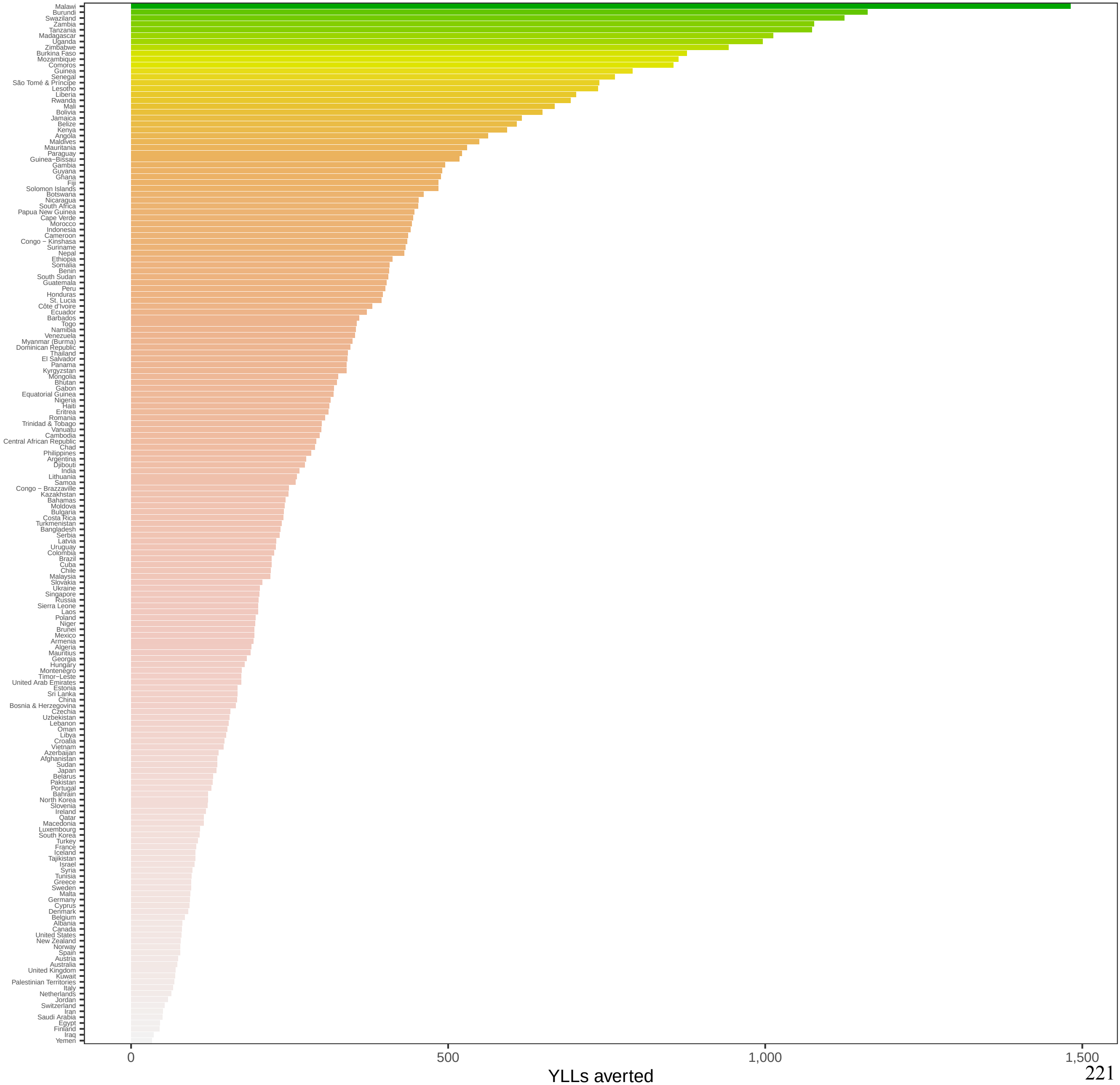

DALYs averted per 1000 vaccinated girls (vaccination age = 9 years / nonavalent vaccine)

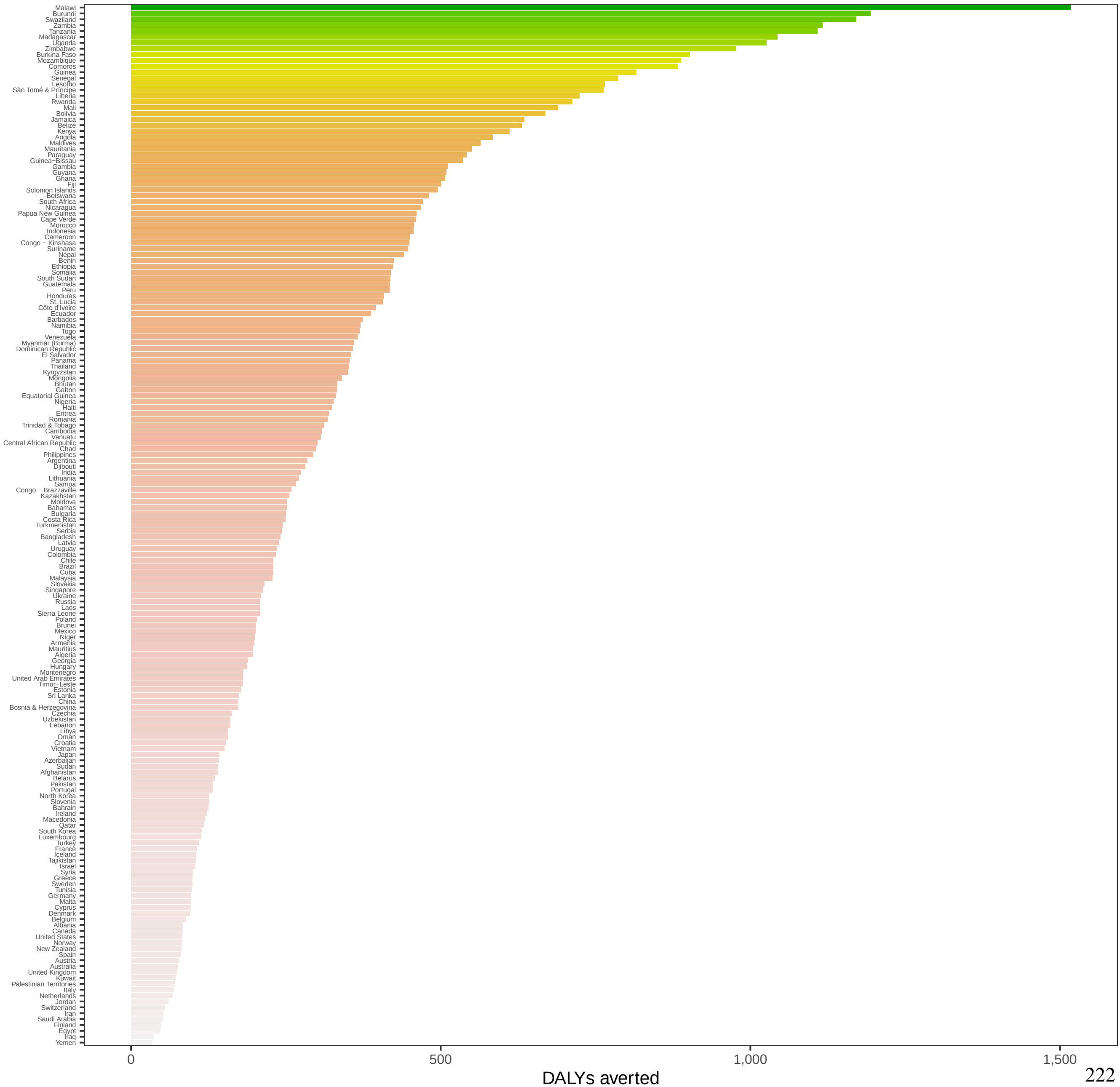

Cases averted per 1000 vaccinated girls (vaccination age = 12 years / bivalent/quadrivalent vaccine)

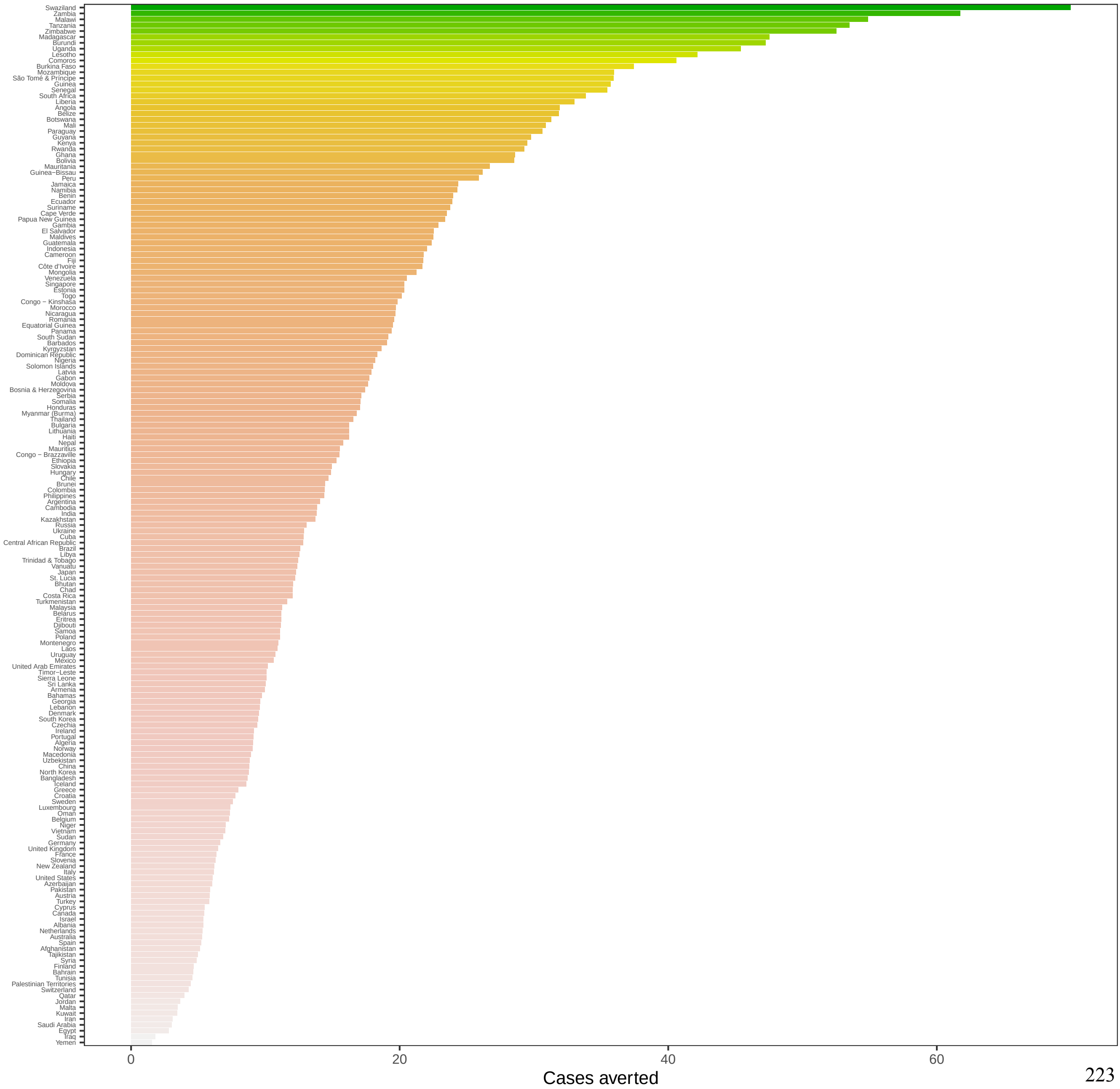

Deaths averted per 1000 vaccinated girls (vaccination age = 12 years / bivalent/quadrivalent vaccine)

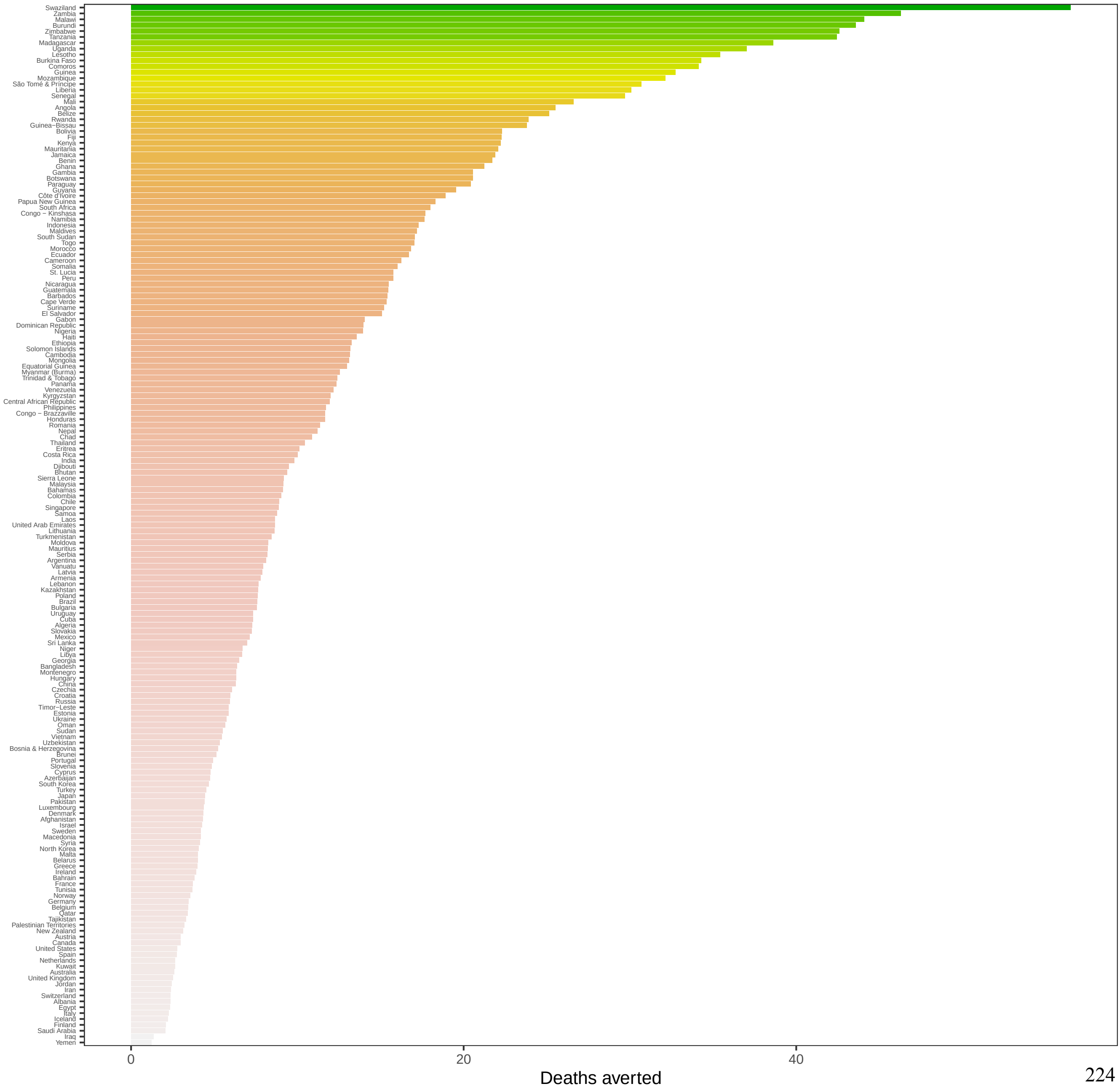

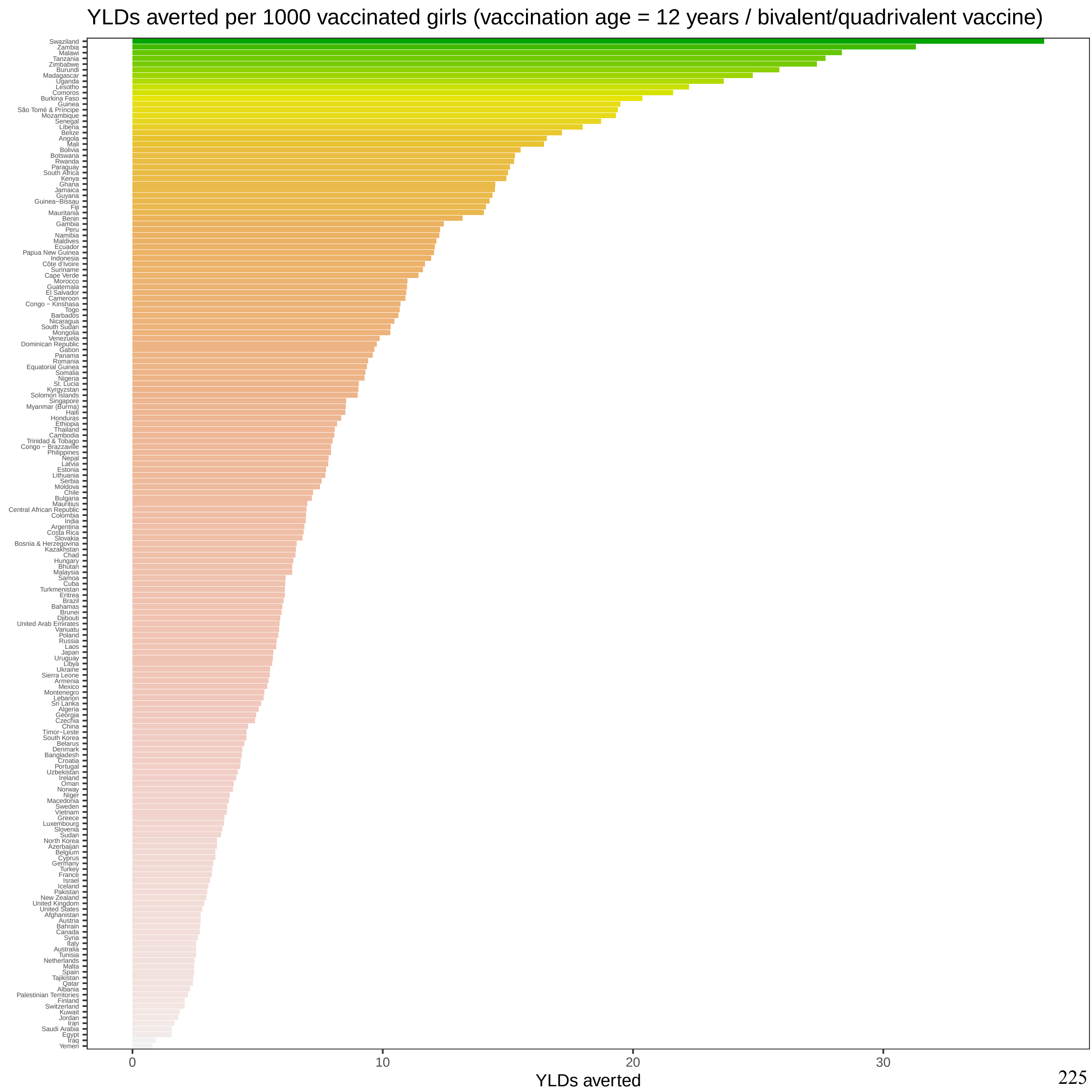
YLDs averted per 1000 vaccinated girls (vaccination age = 12 years / bivalent/quadrivalent vaccine)
YLDs averted
0
10
20
30

YLLs averted per 1000 vaccinated girls (vaccination age = 12 years / bivalent/quadrivalent vaccine)

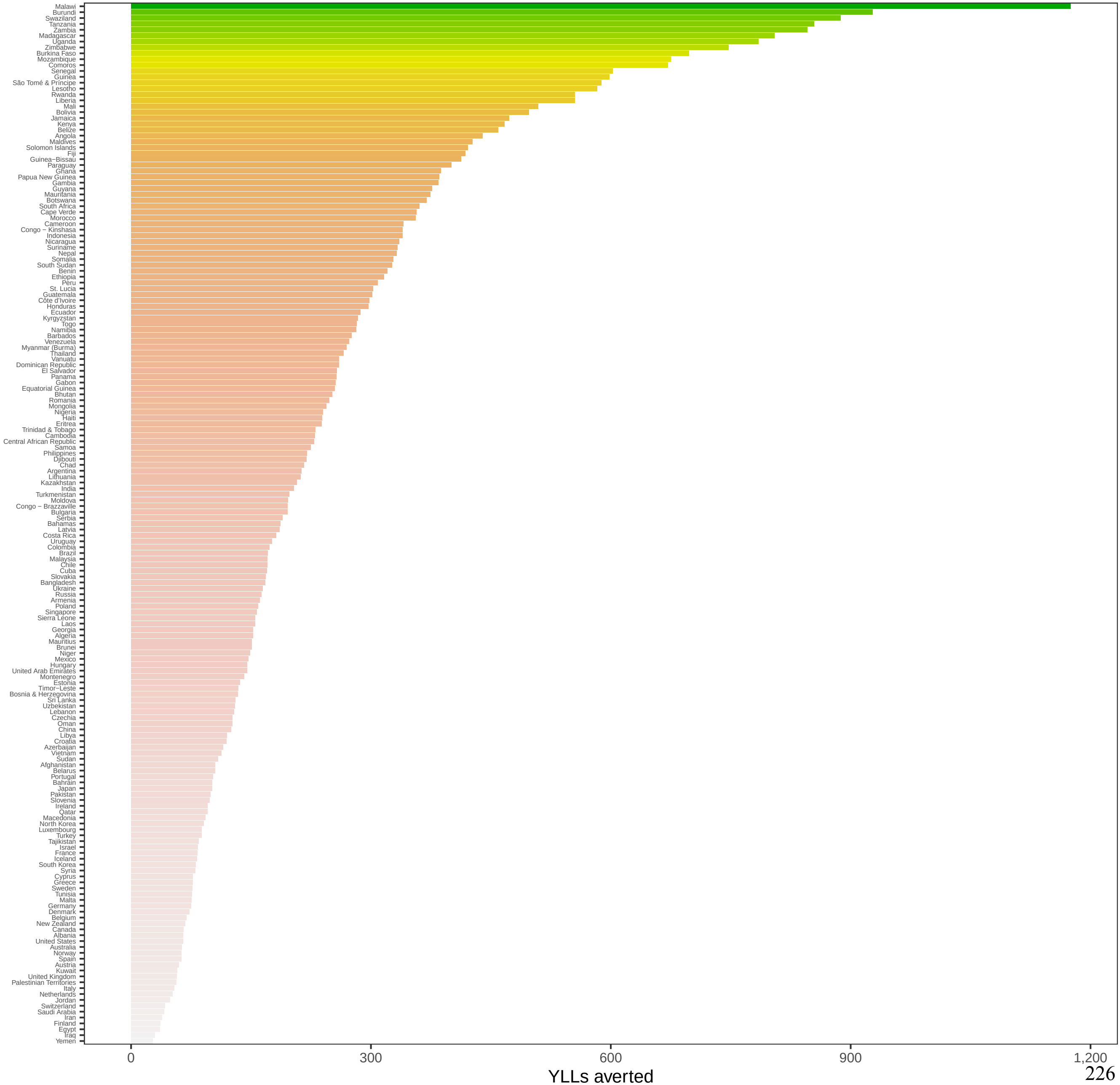

DALYs averted per 1000 vaccinated girls (vaccination age = 12 years / bivalent/quadrivalent vaccine)

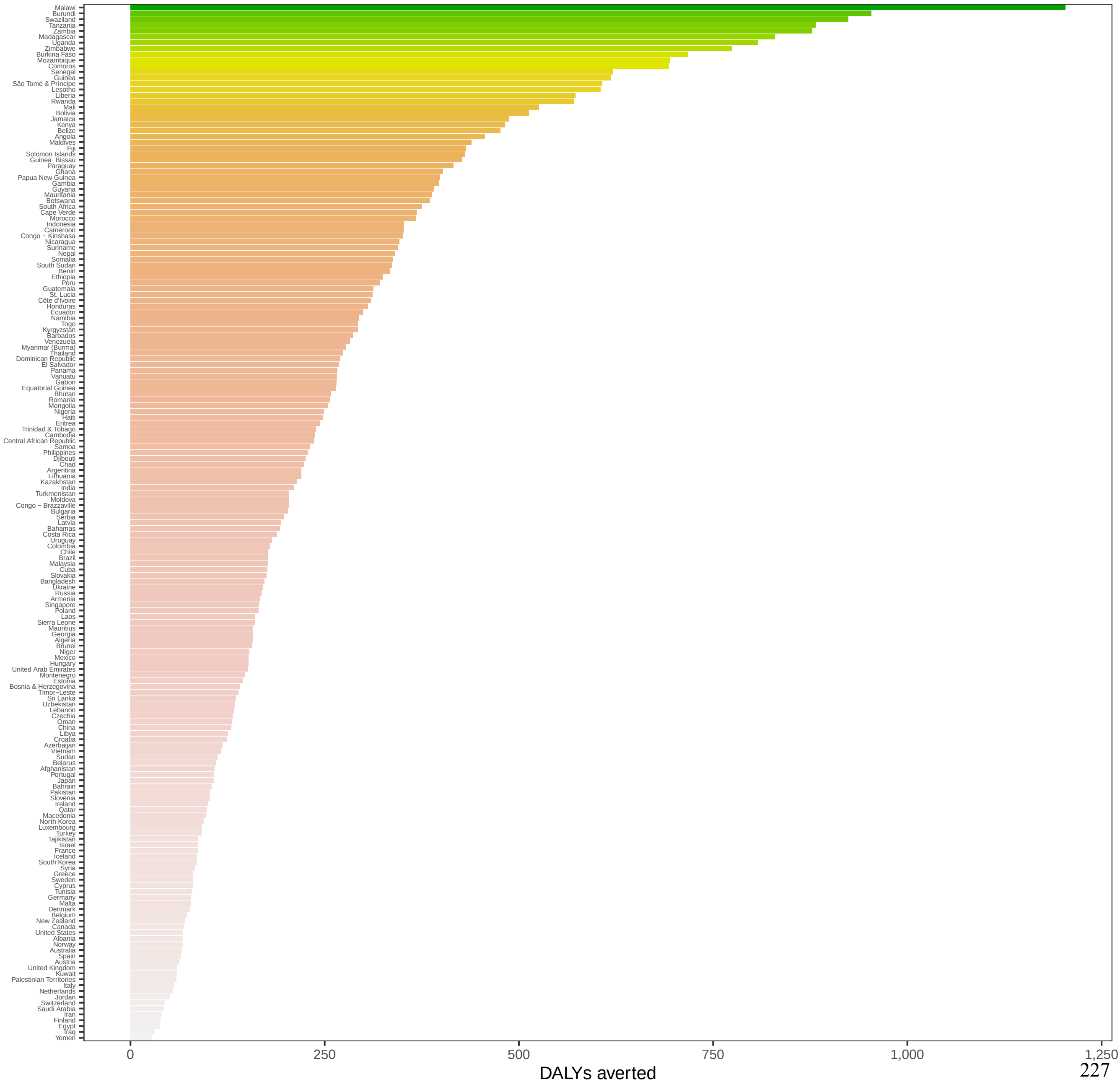

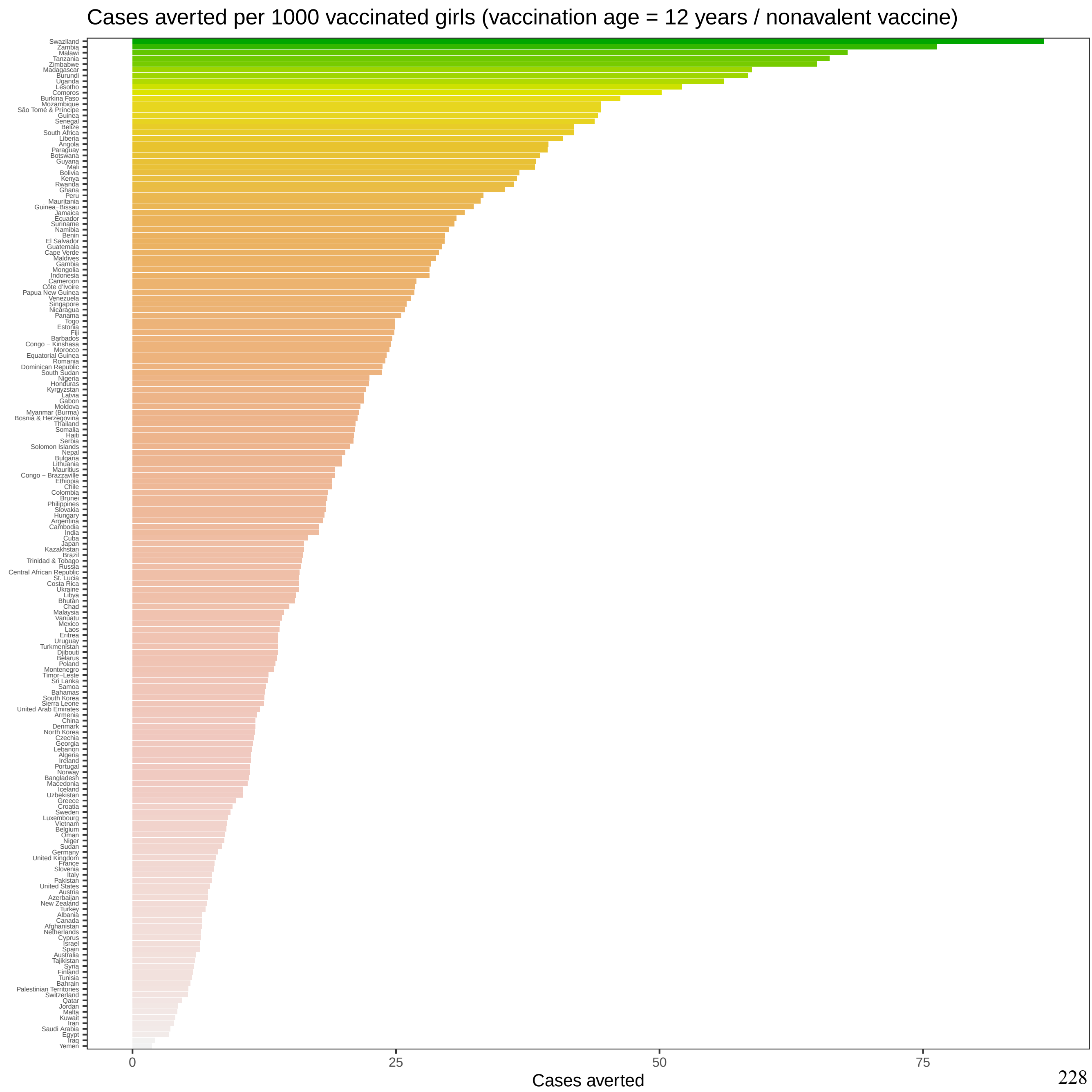
Cases averted per 1000 vaccinated girls (vaccination age = 12 years / nonavalent vaccine)
0
25
50
75
Cases averted

Deaths averted per 1000 vaccinated girls (vaccination age = 12 years / nonavalent vaccine)

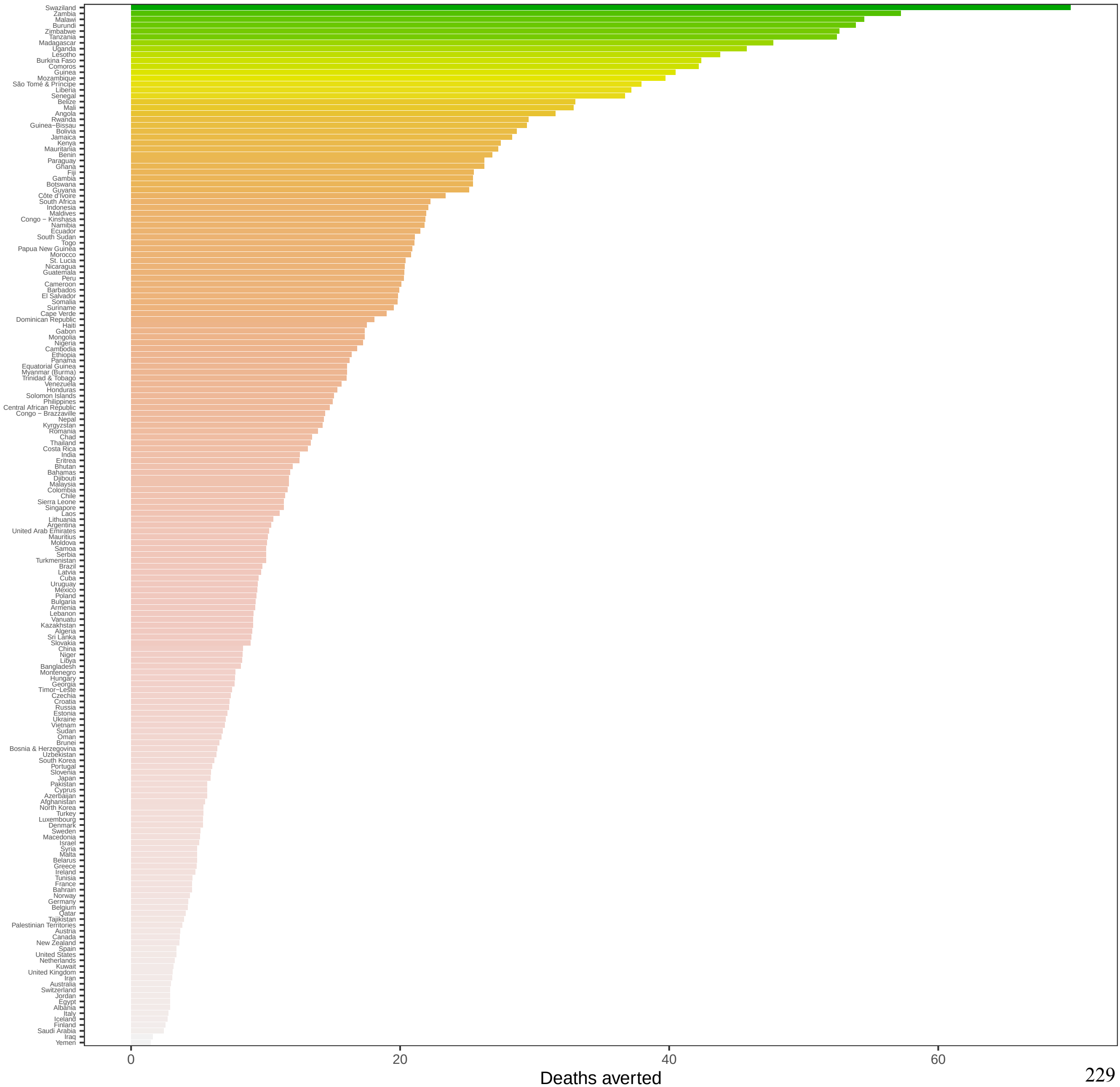

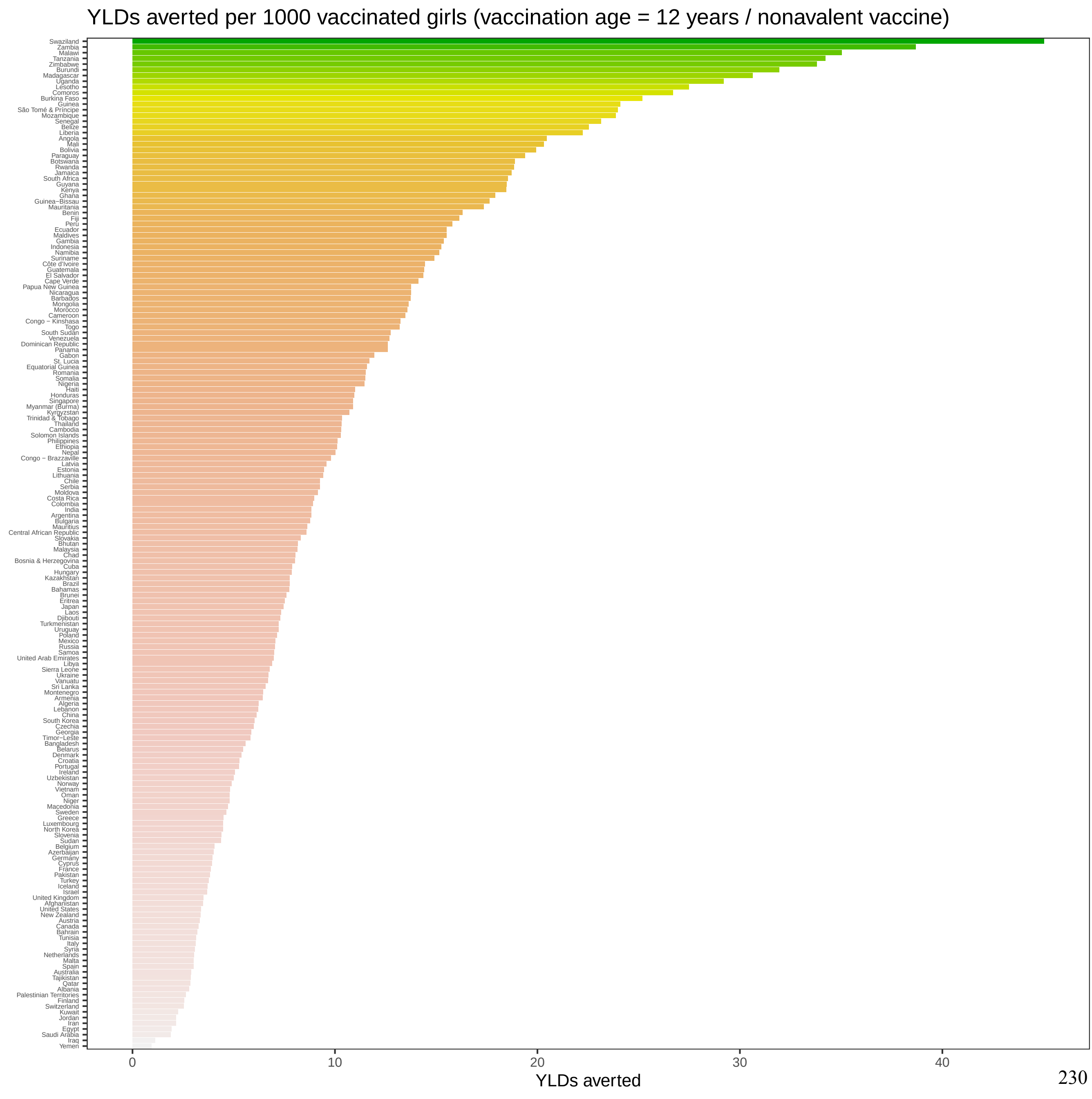
YLDs averted per 1000 vaccinated girls (vaccination age = 12 years / nonavalent vaccine)
Swaziland
Zambia
Malawi
Tanzania
Zimbabwe
Burundi
Madagascar
Uganda
Lesotho
Comoros
Burkina Faso
Guinea
São Tomé & Príncipe
Mozambique
Senegal
Belize
Liberia
Angola
Mali
Bolivia
Paraguay
Botswana
Rwanda
Jamaica
South Africa
Guyana
Kenya
Ghana
Guinea−Bissau
Mauritania
Benin
Fiji
Peru
Ecuador
Maldives
Gambia
Indonesia
Namibia
Suriname
Côte d'Ivoire
Guatemala
El Salvador
Cape Verde
Papua New Guinea
Nicaragua
Barbados
Mongolia
Morocco
Cameroon
Congo − Kinshasa
Togo
South Sudan
Venezuela
Dominican Republic
Panama
Gabon
St. Lucia
Equatorial Guinea
Romania
Somalia
Nigeria
Haiti
Honduras
Singapore
Myanmar (Burma)
Kyrgyzstan
Trinidad & Tobago
Thailand
Cambodia
Solomon Islands
Philippines
Ethiopia
Nepal
Congo − Brazzaville
Latvia
Estonia
Lithuania
Chile
Serbia
Moldova
Costa Rica
Colombia
India
Argentina
Bulgaria
Mauritius
Central African Republic
Slovakia
Bhutan
Malaysia
Chad
Bosnia & Herzegovina
Cuba
Hungary
Kazakhstan
Brazil
Bahamas
Brunei
Eritrea
Japan
Laos
Djibouti
Turkmenistan
Uruguay
Poland
Mexico
Russia
Samoa
United Arab Emirates
Libya
Sierra Leone
Ukraine
Vanuatu
Sri Lanka
Montenegro
Armenia
Algeria
Lebanon
China
South Korea
Czechia
Georgia
Timor−Leste
Bangladesh
Belarus
Denmark
Croatia
Portugal
Ireland
Uzbekistan
Norway
Vietnam
Oman
Niger
Macedonia
Sweden
Greece
Luxembourg
North Korea
Slovenia
Sudan
Belgium
Azerbaijan
Germany
Cyprus
France
Pakistan
Turkey
Iceland
Israel
United Kingdom
Afghanistan
United States
New Zealand
Austria
Canada
Bahrain
Tunisia
Italy
Syria
Netherlands
Malta
Spain
Australia
Tajikistan
Qatar
Albania
Palestinian Territories
Finland
Switzerland
Kuwait
Jordan
Iran
Egypt
Saudi Arabia
Iraq
Yemen
0
10
20
30
40
YLDs averted

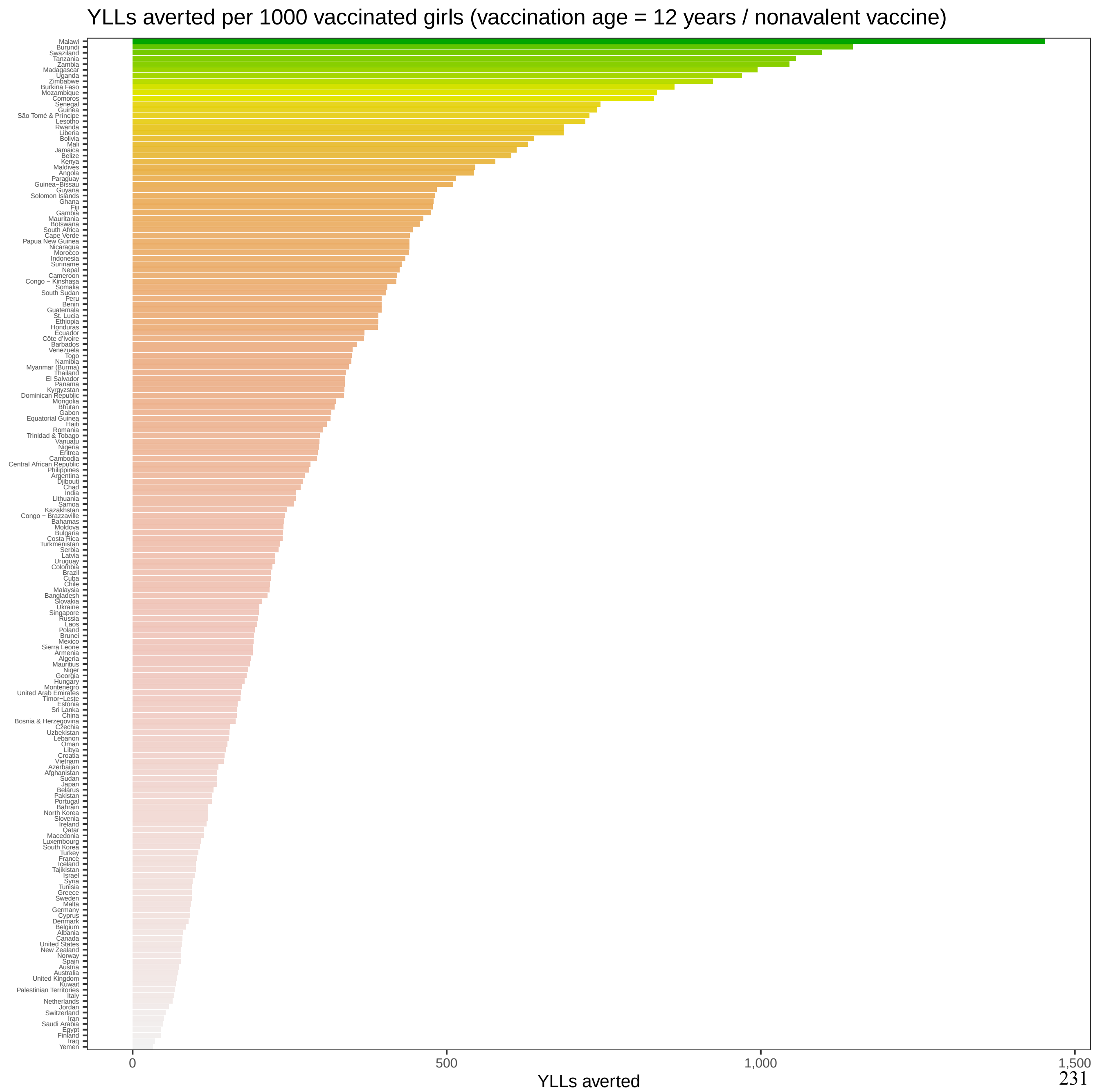
YLLs averted per 1000 vaccinated girls (vaccination age = 12 years / nonavalent vaccine)
YLLs averted
0
500
1,000
1,500

DALYs averted per 1000 vaccinated girls (vaccination age = 12 years / nonavalent vaccine)

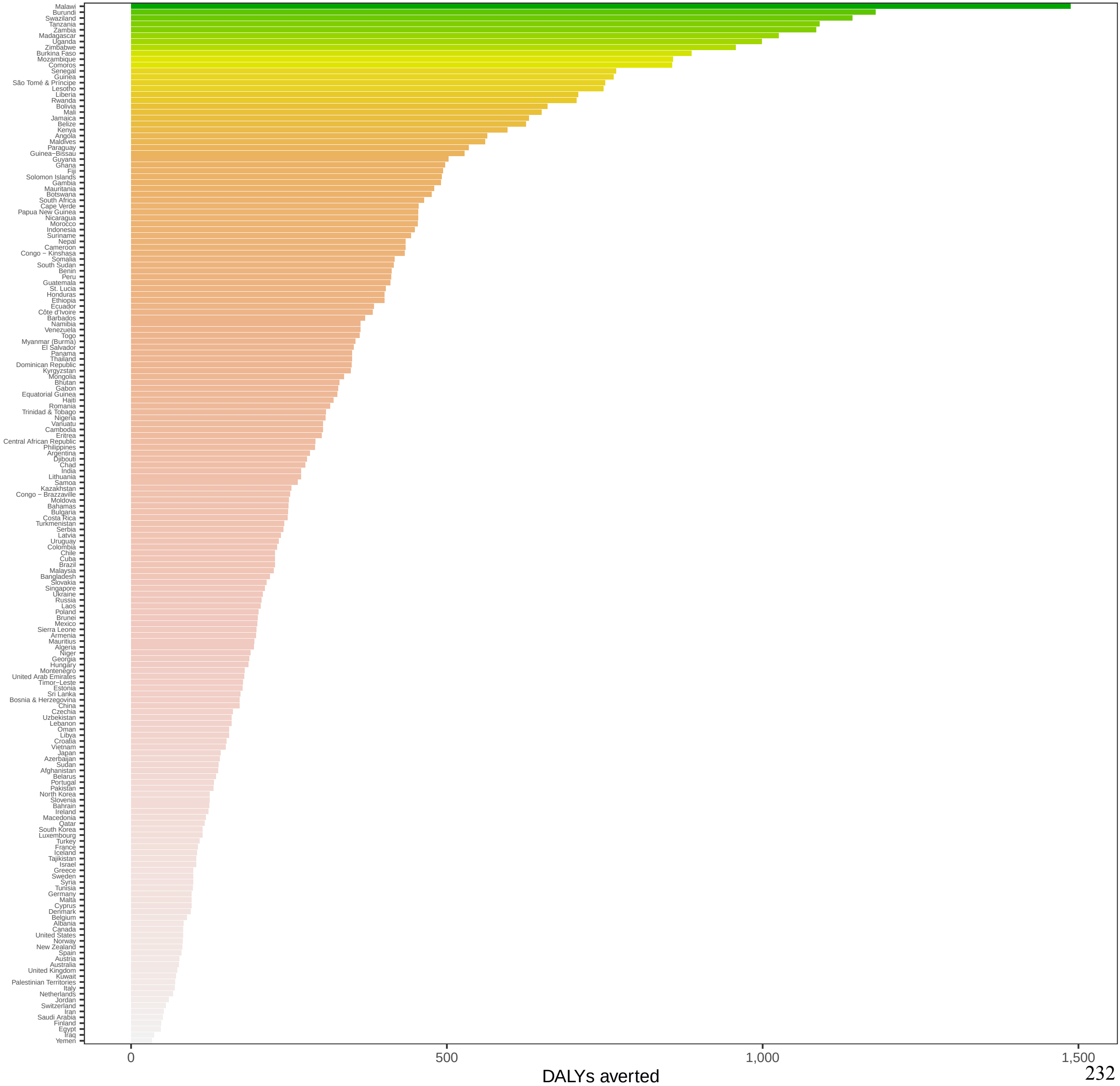

A18. National estimates of HPV vaccination impact

Lifetime health impact of HPV vaccination at 90% coverage during 2020-2029 on cases, deaths, YLLs, YLDs and DALYs averted per 1000 vaccinated girls in 177 countries for bivalent/quadrivalent and nonavalent vaccination of 9-year-old and 12-year-old girls (estimates after the combined PRIME updates for demography, disability weights and cervical cancer burden).

	Cervical cancer burden averted per 1000 vaccinated girls																			
	HPV vaccination of 9-year-old girls										HPV vaccination of 12-year-old girls									
	Bivalent / quadrivalent vaccine					Nonavalent vaccine					Bivalent / quadrivalent vaccine					Nonavalent vaccine				
	Cases	Deaths	YLDs	YLLs	DALYs	Cases	Deaths	YLDs	YLLs	DALYs	Cases	Deaths	YLDs	YLLs	DALYs	Cases	Deaths	YLDs	YLLs	DALYs
**Country**	**averted per 1000 vaccinated girls**																			
Afghanistan	5	4	3	106	109	7	6	3	136	140	5	4	3	105	108	7	6	3	135	138
Albania	5	2	2	66	68	7	3	3	80	83	5	2	2	65	68	7	3	3	80	83
Algeria	9	7	5	154	159	11	9	6	190	196	9	7	5	152	157	11	9	6	188	195
Angola	33	26	17	455	473	41	33	21	563	584	32	26	17	440	456	39	32	20	544	564
Argentina	14	8	7	214	221	18	10	9	276	285	14	8	7	213	220	18	10	9	274	283
Armenia	10	8	5	162	168	12	9	6	193	199	10	8	5	161	166	12	9	6	191	198
Australia	5	3	3	64	66	6	3	3	73	76	5	3	3	64	66	6	3	3	73	76
Austria	6	3	3	60	63	7	4	3	74	77	6	3	3	60	63	7	4	3	73	77
Azerbaijan	6	5	3	116	119	7	6	4	138	142	6	5	3	115	118	7	6	4	137	141
Bahamas	10	9	6	188	194	13	12	8	244	251	10	9	6	186	192	13	12	8	241	249
Bahrain	5	4	3	102	105	6	5	3	121	125	5	4	3	101	104	6	5	3	120	124
Bangladesh	9	7	5	184	189	12	9	6	235	241	9	6	4	168	172	11	8	6	214	220
Barbados	19	16	11	278	289	25	20	14	360	374	19	15	11	276	287	25	20	14	357	371
Belarus	11	4	4	106	110	14	5	5	130	135	11	4	4	105	110	14	5	5	129	134
Belgium	7	3	3	69	73	9	4	4	85	89	7	3	3	69	72	9	4	4	84	89
Belize	32	25	17	463	480	42	33	23	608	631	32	25	17	459	476	42	33	23	603	625

Benin	25	22	13	329	343	30	28	17	407	424	24	22	13	320	334	30	27	16	396	412
Bhutan	12	9	6	254	260	15	12	8	325	333	12	9	6	252	258	15	12	8	322	330
Bolivia	29	23	16	505	520	37	29	20	649	669	29	22	16	497	513	37	29	20	639	659
Bosnia & Herzegovina	17	5	7	135	141	21	6	8	165	173	17	5	7	134	140	21	6	8	164	172
Botswana	32	21	15	373	389	39	26	19	462	481	31	21	15	369	385	39	25	19	457	476
Brazil	13	8	6	172	178	16	10	8	222	229	13	8	6	171	177	16	10	8	220	228
Brunei	14	5	6	152	158	18	7	8	194	202	14	5	6	151	157	18	7	8	193	200
Bulgaria	16	8	7	197	204	20	9	9	241	250	16	8	7	196	203	20	9	9	239	248
Burkina Faso	38	35	21	709	730	47	43	25	877	902	37	34	20	698	718	46	42	25	862	888
Burundi	48	44	26	940	966	59	54	32	1161	1194	47	44	26	928	953	58	54	32	1147	1179
Cambodia	14	13	8	232	241	18	17	10	297	307	14	13	8	230	238	18	17	10	294	304
Cameroon	22	17	11	353	364	28	21	14	437	450	22	16	11	340	351	27	20	13	421	434
Canada	5	3	3	66	69	7	4	3	80	83	5	3	3	66	68	7	4	3	79	83
Cape Verde	24	15	11	360	371	29	19	14	445	459	23	15	11	357	368	29	19	14	441	455
Central African Republic	13	12	7	236	243	16	15	9	292	301	13	12	7	229	236	16	15	9	283	292
Chad	13	12	7	234	242	16	15	9	290	299	12	11	7	216	223	15	13	8	267	275
Chile	15	9	7	171	179	19	12	9	220	229	15	9	7	170	177	19	11	9	219	228
China	9	6	5	126	130	12	8	6	167	173	9	6	5	125	129	12	8	6	165	172
Colombia	15	9	7	175	182	19	12	9	225	234	14	9	7	173	180	19	12	9	222	231
Comoros	42	35	22	692	714	51	43	27	855	882	41	34	22	672	693	50	42	27	830	857
Congo - Brazzaville	16	12	8	201	210	20	15	10	249	259	15	12	8	196	204	19	14	10	242	252
Congo - Kinshasa	20	18	11	352	363	25	23	14	436	449	20	18	11	340	350	25	22	13	420	433
Costa Rica	12	10	7	183	190	16	13	9	240	249	12	10	7	182	188	16	13	9	239	248
Croatia	8	6	4	120	125	10	7	5	147	152	8	6	4	119	124	9	7	5	146	151
Cuba	13	7	6	171	177	17	10	8	221	229	13	7	6	170	176	17	9	8	220	228
Cyprus	5	5	3	78	81	7	6	4	92	96	5	5	3	77	80	6	6	4	92	95
Czechia	9	6	5	128	133	12	7	6	156	162	9	6	5	127	132	11	7	6	155	161

Cote d'Ivoire	22	19	12	308	320	28	24	15	380	395	22	19	12	298	309	27	23	14	368	382
Denmark	10	4	4	73	78	12	5	5	90	95	10	4	4	73	77	12	5	5	89	94
Djibouti	11	10	6	222	227	14	12	7	274	281	11	9	6	219	225	14	12	7	271	279
Dominican Republic	19	14	10	267	277	24	19	13	345	358	18	14	10	260	270	24	18	13	336	349
Ecuador	24	17	12	289	301	31	22	16	372	387	24	17	12	287	299	31	21	16	369	384
Egypt	3	2	2	37	38	4	3	2	45	47	3	2	2	36	38	3	3	2	45	47
El Salvador	23	15	11	260	271	30	20	14	341	356	23	15	11	257	268	30	20	14	338	352
Equatorial Guinea	20	13	9	258	267	24	16	12	319	330	19	13	9	255	264	24	16	12	315	327
Eritrea	12	11	6	252	258	15	13	8	311	319	11	10	6	238	244	14	13	8	294	302
Estonia	20	6	8	137	145	25	7	9	168	177	20	6	8	136	144	25	7	9	167	176
Ethiopia	16	14	9	333	342	20	17	11	412	423	15	13	8	316	325	19	16	10	391	401
Fiji	22	23	14	424	438	25	26	16	485	501	22	22	14	418	432	25	25	16	478	494
Finland	5	2	2	37	39	6	3	3	45	48	5	2	2	37	39	6	3	3	45	47
France	6	4	3	84	87	8	5	4	102	106	6	4	3	83	86	8	5	4	102	106
Gabon	18	14	10	259	269	22	18	12	320	332	18	14	10	256	265	22	17	12	316	328
Gambia	24	21	13	400	413	29	26	16	495	511	23	21	12	384	397	28	25	15	475	490
Georgia	10	7	5	154	159	11	8	6	183	188	10	6	5	153	158	11	8	6	181	187
Germany	7	3	3	75	79	8	4	4	92	96	7	3	3	75	78	8	4	4	92	96
Ghana	29	22	15	395	410	36	27	18	489	507	29	21	14	388	402	35	26	18	479	497
Greece	8	4	4	77	81	10	5	5	95	99	8	4	4	77	81	10	5	4	94	99
Guatemala	23	16	11	307	318	30	21	15	403	418	22	15	11	301	312	29	20	14	396	410
Guinea	38	35	21	639	660	47	43	26	791	816	36	33	19	598	618	44	40	24	740	764
Guinea-Bissau	26	24	14	419	433	33	30	18	518	536	26	24	14	413	427	32	29	18	510	528
Guyana	30	20	15	382	396	39	25	19	490	509	30	20	14	377	391	38	25	18	484	503
Haiti	16	14	9	242	250	21	18	11	313	324	16	14	9	239	248	21	18	11	309	320
Honduras	17	12	8	302	310	23	16	11	397	408	17	12	8	297	305	22	15	11	390	401
Hungary	15	6	6	146	153	18	8	8	179	187	15	6	6	145	152	18	8	8	178	186

Iceland	9	2	3	83	86	11	3	4	101	105	9	2	3	82	85	10	3	4	101	105
India	14	10	7	208	215	18	13	9	265	274	14	10	7	204	211	18	13	9	260	269
Indonesia	22	18	12	345	357	28	22	15	441	456	22	17	12	340	351	28	22	15	434	449
Iran	3	2	2	39	41	4	3	2	50	52	3	2	2	39	41	4	3	2	50	52
Iraq	2	1	1	30	31	2	2	1	36	37	2	1	1	30	31	2	2	1	36	37
Ireland	9	4	4	96	101	11	5	5	118	123	9	4	4	96	100	11	5	5	117	122
Israel	5	4	3	84	87	6	5	4	100	104	5	4	3	84	87	6	5	4	99	103
Italy	6	2	3	54	57	8	3	3	66	69	6	2	3	54	56	8	3	3	66	69
Jamaica	24	22	15	477	491	32	28	19	616	635	24	22	14	473	487	31	28	19	611	630
Japan	12	4	6	102	107	16	6	8	135	142	12	4	6	101	107	16	6	7	134	142
Jordan	4	2	2	49	51	4	3	2	58	60	4	2	2	49	50	4	3	2	58	60
Kazakhstan	14	8	7	209	215	16	9	8	248	256	14	8	7	207	214	16	9	8	246	254
Kenya	30	23	15	479	495	37	28	19	593	611	29	22	15	467	482	36	27	18	577	596
Kuwait	3	3	2	58	60	4	3	2	69	72	3	3	2	58	60	4	3	2	69	71
Kyrgyzstan	19	12	9	286	295	22	14	11	340	350	19	12	9	284	293	22	14	11	337	347
Laos	11	9	6	157	163	14	11	7	200	208	11	9	6	155	161	14	11	7	198	205
Latvia	18	8	8	187	195	22	10	10	229	238	18	8	8	186	193	22	10	10	227	237
Lebanon	10	8	5	130	135	11	9	6	154	160	10	8	5	129	134	11	9	6	153	159
Lesotho	43	36	23	596	619	53	45	28	737	765	42	35	22	583	605	52	44	27	720	748
Liberia	34	31	18	567	586	42	38	23	701	724	33	30	18	555	573	41	37	22	686	708
Libya	13	7	6	121	127	16	8	7	150	157	13	7	6	120	125	15	8	7	148	155
Lithuania	16	9	8	213	221	20	11	9	261	271	16	9	8	212	220	20	11	9	260	269
Luxembourg	7	4	4	89	93	9	5	4	109	113	7	4	4	88	92	9	5	4	108	113
Macedonia	9	4	4	94	97	11	5	5	114	119	9	4	4	93	97	11	5	5	114	118
Madagascar	48	39	25	819	844	60	48	31	1013	1044	48	39	25	805	829	59	48	31	995	1025
Malawi	56	45	29	1199	1228	69	55	36	1482	1517	55	44	28	1175	1204	68	55	35	1453	1488
Malaysia	11	9	6	172	178	14	12	8	220	228	11	9	6	170	177	14	12	8	218	226

Maldives	23	17	12	429	442	29	22	16	549	564	23	17	12	427	439	29	22	16	545	561
Mali	33	28	17	540	557	40	35	21	668	689	31	27	16	509	526	38	33	20	629	650
Malta	3	4	2	76	79	4	5	3	93	96	3	4	2	76	78	4	5	3	93	96
Mauritania	30	25	16	429	445	38	31	20	530	550	27	22	14	374	388	33	27	17	462	480
Mauritius	16	8	7	152	159	19	10	9	188	197	16	8	7	151	158	19	10	9	187	195
Mexico	11	7	5	148	153	14	9	7	194	201	11	7	5	146	152	14	9	7	192	199
Moldova	18	8	8	198	206	22	10	9	242	252	18	8	7	196	204	22	10	9	240	249
Mongolia	21	13	10	247	257	28	18	14	327	340	21	13	10	244	254	28	17	14	323	337
Montenegro	11	6	5	143	148	13	8	6	175	181	11	6	5	142	147	13	8	6	173	180
Morocco	20	17	11	358	369	24	21	14	443	457	20	17	11	356	367	24	21	14	440	454
Mozambique	37	33	20	698	718	46	41	25	863	888	36	32	19	675	694	44	40	24	834	858
Myanmar	17	13	9	273	282	22	16	11	349	360	17	13	9	269	278	21	16	11	344	355
Namibia	25	18	12	287	299	30	22	15	355	370	24	18	12	281	294	30	22	15	348	363
Nepal	16	11	8	337	345	20	14	10	431	441	16	11	8	332	340	20	14	10	425	435
Netherlands	5	3	2	52	54	7	3	3	64	67	5	3	2	52	54	7	3	3	63	66
New Zealand	6	3	3	68	71	7	4	3	78	81	6	3	3	68	71	7	4	3	77	81
Nicaragua	20	16	11	345	356	27	21	14	453	467	20	15	10	335	346	26	20	14	441	454
Niger	7	7	4	158	162	9	9	5	196	201	7	7	4	149	153	9	8	5	184	189
Nigeria	19	15	10	255	264	24	18	12	315	327	18	14	9	240	249	22	17	11	297	308
North Korea	9	4	3	91	95	12	5	4	121	125	9	4	3	91	94	12	5	4	120	125
Norway	9	4	4	63	67	11	4	5	78	82	9	4	4	63	67	11	4	5	77	82
Oman	7	6	4	128	132	9	7	5	152	156	7	6	4	127	131	9	7	5	150	155
Pakistan	6	4	3	101	104	8	6	4	128	132	6	4	3	99	102	7	6	4	127	131
Palestinian Territories	4	3	2	57	60	5	4	3	68	71	4	3	2	57	59	5	4	3	67	70
Panama	20	12	10	259	268	26	16	13	340	352	19	12	10	257	267	26	16	13	338	350
Papua New Guinea	24	18	12	391	403	27	21	14	447	461	23	18	12	386	398	27	21	14	441	455
Paraguay	31	21	15	406	421	40	27	20	522	541	31	20	15	401	416	39	26	19	515	534

Peru	26	16	12	312	324	34	20	16	401	417	26	16	12	308	321	33	20	16	396	412
Philippines	14	12	8	222	230	18	15	10	284	294	14	12	8	220	228	18	15	10	281	291
Poland	11	8	6	160	166	14	9	7	196	203	11	8	6	159	165	14	9	7	195	202
Portugal	9	5	4	103	108	11	6	5	126	132	9	5	4	103	107	11	6	5	126	131
Qatar	4	3	2	96	99	5	4	3	114	117	4	3	2	96	98	5	4	3	114	117
Romania	20	11	9	250	259	24	14	12	306	317	20	11	9	248	257	24	14	12	303	315
Russia	13	6	6	164	170	16	7	7	201	208	13	6	6	163	169	16	7	7	200	207
Rwanda	29	24	15	561	576	36	30	19	693	712	29	24	15	555	570	36	30	19	686	705
Samoa	11	9	6	227	233	13	10	7	259	266	11	9	6	225	231	13	10	7	257	264
Saudi Arabia	3	2	2	41	43	4	2	2	49	51	3	2	2	41	43	4	2	2	49	51
Senegal	36	30	19	617	636	45	37	24	763	786	35	30	19	603	621	44	37	23	745	768
Serbia	17	8	8	191	199	21	10	9	234	243	17	8	8	189	197	21	10	9	232	241
Sierra Leone	10	10	6	162	168	13	12	7	200	207	10	9	5	155	161	12	11	7	192	198
Singapore	20	9	9	158	167	26	11	11	202	213	20	9	9	157	166	26	11	11	201	212
Slovakia	15	7	7	169	176	18	9	8	207	215	15	7	7	168	175	18	9	8	206	214
Slovenia	6	5	4	99	102	8	6	4	121	125	6	5	4	98	102	8	6	4	120	124
Solomon Islands	18	13	9	424	433	21	15	10	485	495	18	13	9	421	430	21	15	10	481	492
Somalia	17	16	9	330	339	21	20	12	408	419	17	16	9	328	337	21	20	11	405	417
South Africa	34	18	15	366	381	42	23	19	452	471	34	18	15	360	375	42	22	19	446	464
South Korea	9	5	5	81	86	13	6	6	108	114	9	5	5	81	85	13	6	6	107	113
South Sudan	19	17	10	328	339	24	21	13	406	419	19	17	10	326	337	24	21	13	403	416
Spain	5	3	2	63	66	6	3	3	77	80	5	3	2	63	65	6	3	3	77	80
Sri Lanka	10	7	5	131	136	13	9	7	168	174	10	7	5	130	135	13	9	7	167	173
St. Lucia	12	16	9	305	314	16	21	12	395	407	12	16	9	303	312	16	20	12	391	403
Sudan	7	6	4	110	113	9	7	4	136	140	7	6	4	109	112	8	7	4	134	139
Suriname	24	15	12	336	348	31	20	15	432	447	24	15	12	333	345	31	20	15	428	443
Swaziland	72	58	37	910	947	88	72	46	1125	1171	70	57	36	888	924	87	70	45	1097	1142

Sweden	8	4	4	77	81	9	5	5	94	99	8	4	4	77	80	9	5	5	94	98
Switzerland	4	2	2	43	45	5	3	3	53	55	4	2	2	43	45	5	3	3	52	55
Syria	5	4	3	81	84	6	5	3	96	99	5	4	3	80	83	6	5	3	95	98
São Tomé and Príncipe	36	31	20	597	617	45	38	24	738	763	36	31	19	588	607	44	38	24	727	751
Tajikistan	5	3	2	85	88	6	4	3	101	104	5	3	2	85	87	6	4	3	100	103
Tanzania	54	43	28	869	897	67	53	35	1074	1108	53	42	28	854	882	66	52	34	1056	1090
Thailand	17	10	8	267	276	21	13	10	342	352	17	10	8	266	274	21	13	10	340	350
Timor-Leste	10	6	5	136	141	13	8	6	174	180	10	6	5	134	139	13	8	6	171	177
Togo	20	17	11	288	299	25	21	13	356	369	20	17	11	282	293	25	21	13	349	362
Trinidad & Tobago	12	12	8	233	241	16	16	10	301	311	12	12	8	231	239	16	16	10	298	308
Tunisia	5	4	3	77	79	6	5	3	95	98	5	4	3	76	79	6	5	3	94	97
Turkey	6	5	3	89	92	7	5	4	105	109	6	5	3	88	91	7	5	4	105	108
Turkmenistan	12	9	6	200	206	14	10	7	237	244	12	8	6	198	204	14	10	7	235	242
Uganda	46	38	24	806	830	57	47	30	996	1026	45	37	24	785	808	56	46	29	970	999
Ukraine	13	6	6	166	171	16	7	7	203	209	13	6	5	165	170	16	7	7	201	208
United Arab Emirates	10	9	6	146	152	12	10	7	174	181	10	9	6	145	151	12	10	7	172	179
United Kingdom	6	3	3	57	60	8	3	4	70	74	6	3	3	57	60	8	3	4	70	73
United States	6	3	3	65	68	7	3	3	79	83	6	3	3	65	68	7	3	3	79	82
Uruguay	11	7	6	178	183	14	9	7	228	236	11	7	6	176	182	14	9	7	227	234
Uzbekistan	9	5	4	131	135	11	6	5	155	160	9	5	4	130	134	10	6	5	154	159
Vanuatu	12	8	6	262	268	14	9	7	300	306	12	8	6	260	266	14	9	7	297	304
Venezuela	21	12	10	275	285	26	16	13	353	366	21	12	10	273	282	26	16	13	350	363
Vietnam	7	5	4	114	118	9	7	5	146	151	7	5	4	113	117	9	7	5	145	150
Yemen	2	1	1	27	28	2	1	1	33	34	2	1	1	27	28	2	1	1	32	33
Zambia	63	47	32	871	903	78	59	40	1077	1117	62	46	31	846	877	76	57	39	1046	1084
Zimbabwe	53	43	28	762	790	66	54	34	943	977	53	43	27	747	775	65	53	34	924	957

A19. Vaccination impact in comparative scenarios

Percentage changes in the lifetime impact of HPV vaccination on cases, deaths, YLLs, YLDs and DALYs averted per 1000 vaccinated girls in each of the 177 countries for the different comparative scenarios (s2, s3, s4, s5) in comparison to the base scenario (s1) for bivalent/quadrivalent and nonavalent vaccination of 9-year-old and 12-year-old girls.

Percentage change in cases averted per 1000 vaccinated girls
(vaccination age = 9 years / bivalent/quadrivalent vaccine)

Comparsion of scenario s2 in comparison to scenario s1

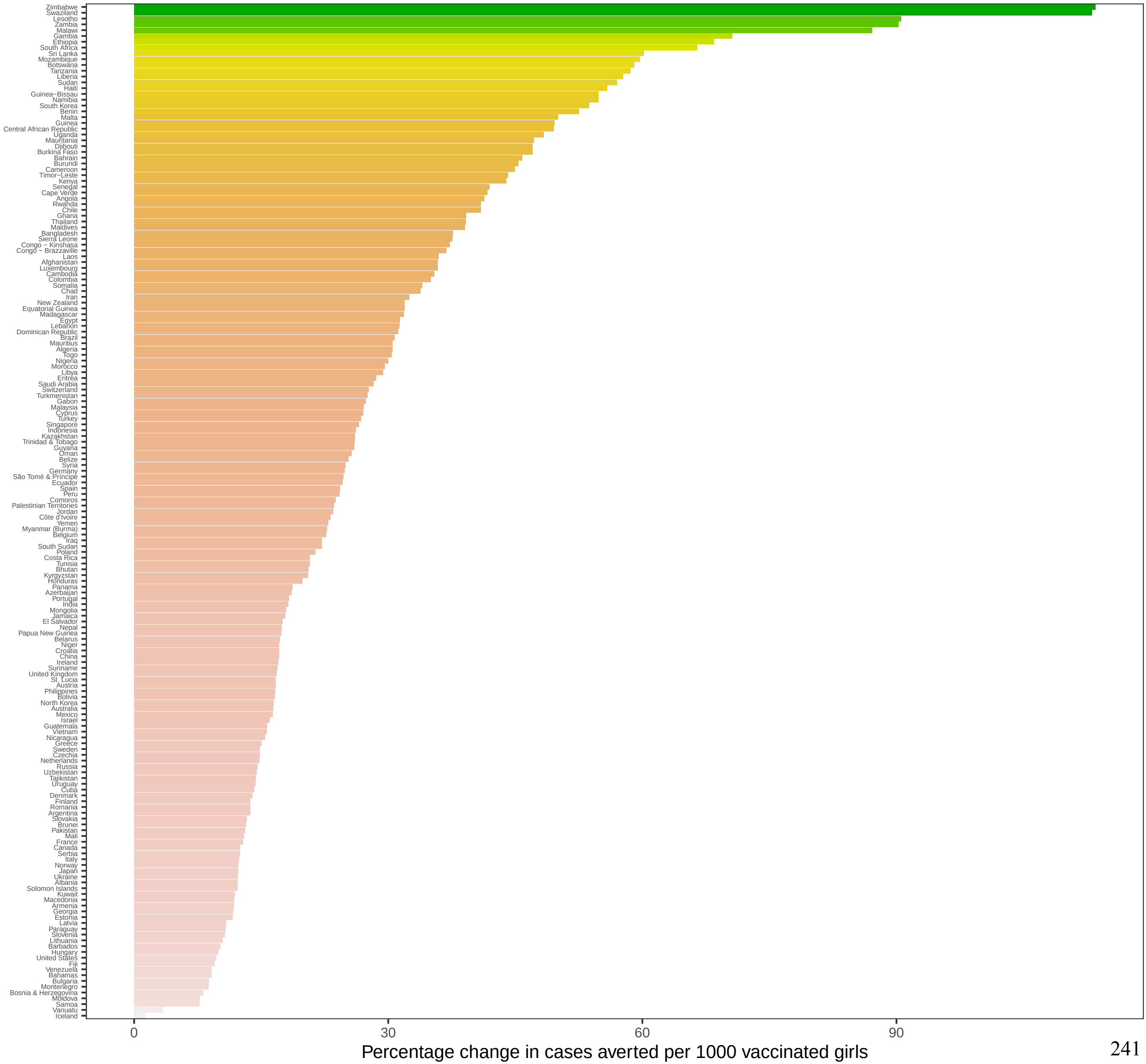

Percentage change in deaths averted per 1000 vaccinated girls (vaccination age = 9 years / bivalent/quadrivalent vaccine)

Comparsion of scenario s2 in comparison to scenario s1

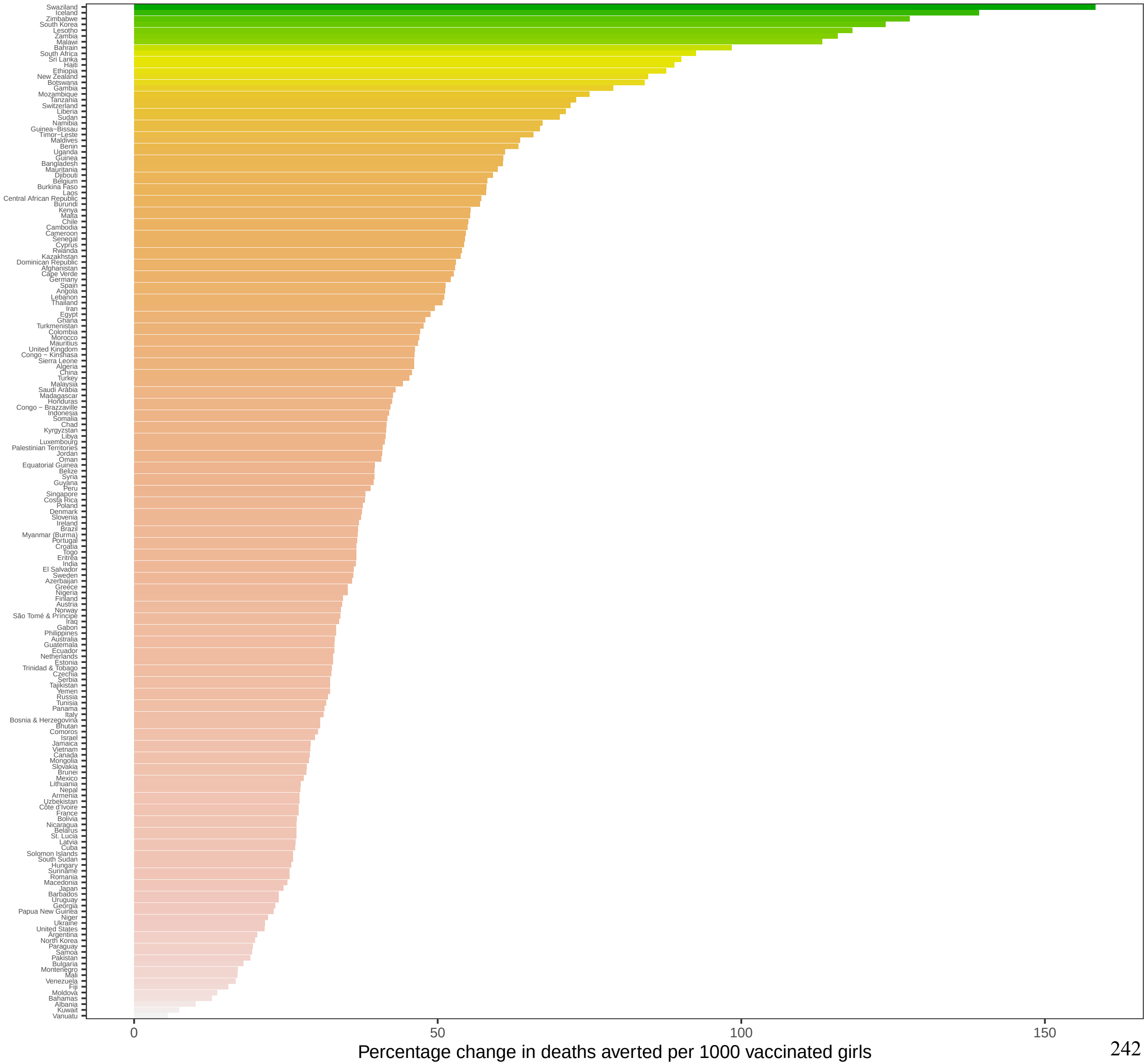

Percentage change in YLDs averted per 1000 vaccinated girls
(vaccination age = 9 years / bivalent/quadrivalent vaccine)

Comparsion of scenario s2 in comparison to scenario s1

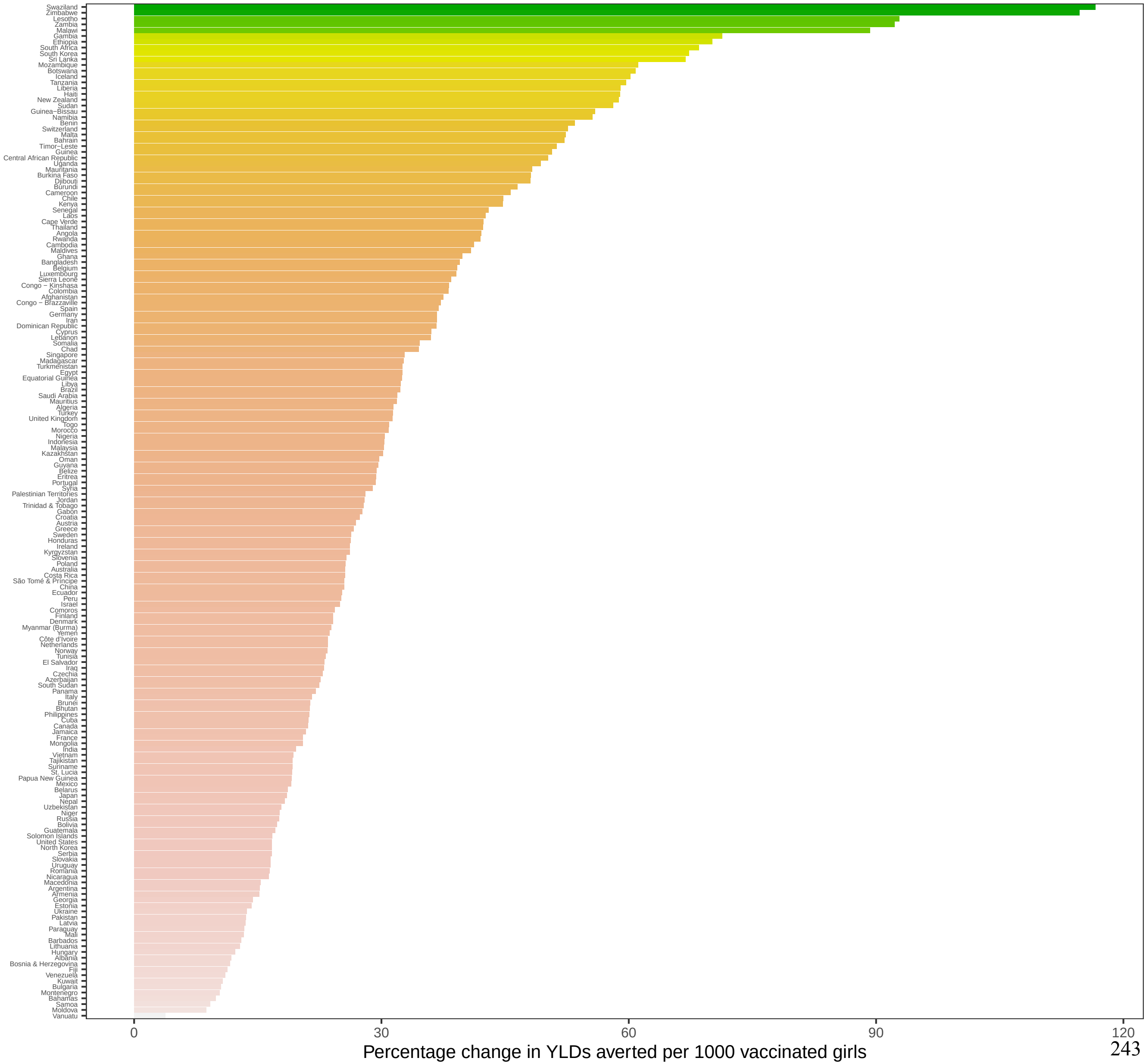

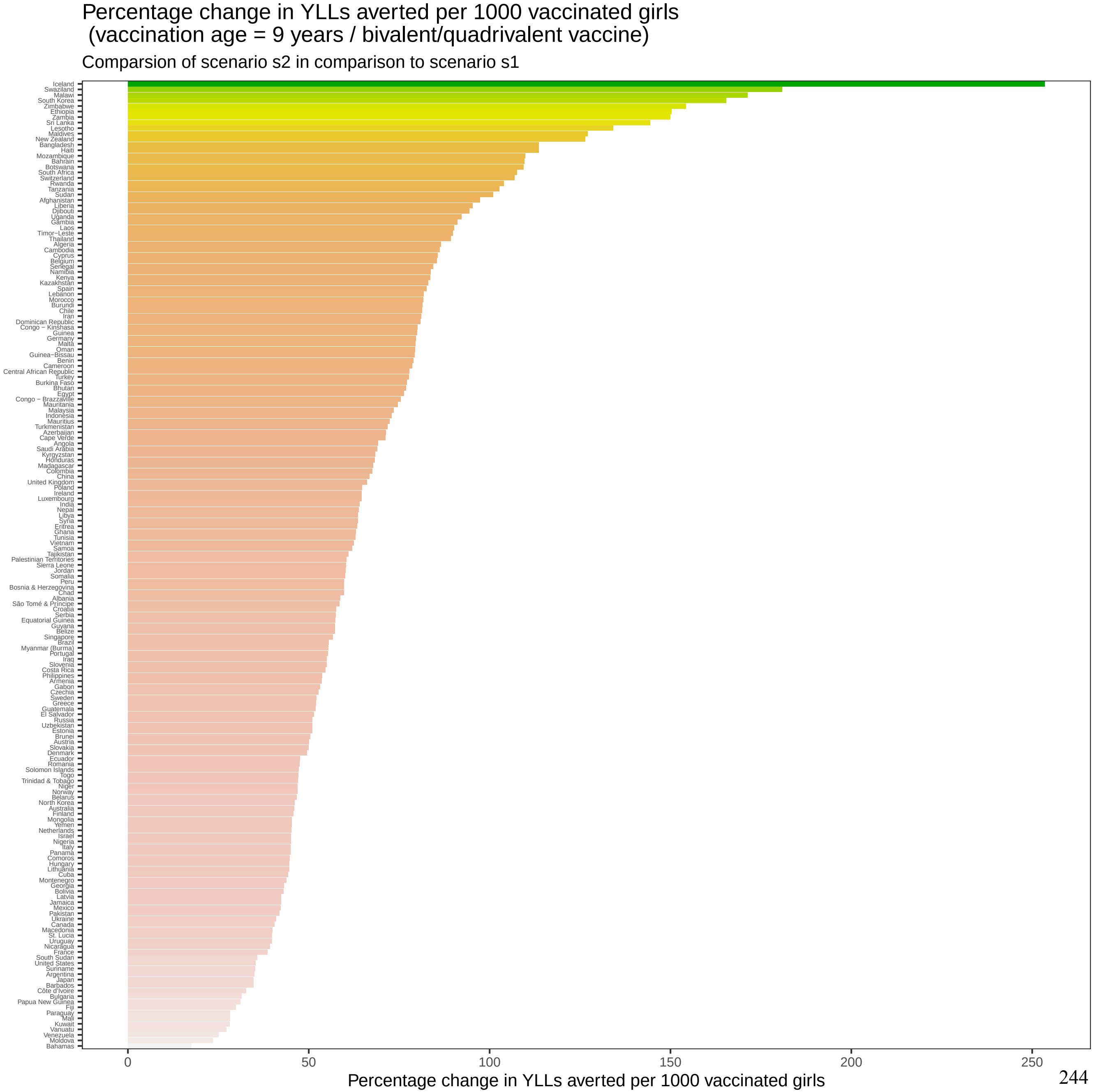
Percentage change in YLLs averted per 1000 vaccinated girls
(vaccination age = 9 years / bivalent/quadrivalent vaccine)
Comparsion of scenario s2 in comparison to scenario s1
Iceland
Swaziland
Malawi
South Korea
Zimbabwe
Ethiopia
Zambia
Sri Lanka
Lesotho
Maldives
New Zealand
Bangladesh
Haiti
Mozambique
Bahrain
Botswana
South Africa
Switzerland
Rwanda
Tanzania
Sudan
Afghanistan
Liberia
Djibouti
Uganda
Gambia
Laos
Timor−Leste
Thailand
Algeria
Cambodia
Cyprus
Belgium
Senegal
Namibia
Kenya
Kazakhstan
Spain
Lebanon
Morocco
Burundi
Chile
Iran
Dominican Republic
Congo − Kinshasa
Guinea
Germany
Malta
Oman
Guinea−Bissau
Benin
Cameroon
Central African Republic
Turkey
Burkina Faso
Bhutan
Egypt
Congo − Brazzaville
Mauritania
Malaysia
Indonesia
Mauritius
Turkmenistan
Azerbaijan
Cape Verde
Angola
Saudi Arabia
Kyrgyzstan
Honduras
Madagascar
Colombia
China
United Kingdom
Poland
Ireland
Luxembourg
India
Nepal
Libya
Syria
Eritrea
Ghana
Tunisia
Vietnam
Samoa
Tajikistan
Palestinian Territories
Sierra Leone
Jordan
Somalia
Peru
Bosnia & Herzegovina
Chad
Albania
São Tomé & Príncipe
Croatia
Serbia
Equatorial Guinea
Guyana
Belize
Singapore
Brazil
Myanmar (Burma)
Portugal
Iraq
Slovenia
Costa Rica
Philippines
Armenia
Gabon
Czechia
Sweden
Greece
Guatemala
El Salvador
Russia
Uzbekistan
Estonia
Brunei
Austria
Slovakia
Denmark
Ecuador
Romania
Solomon Islands
Togo
Trinidad & Tobago
Niger
Norway
Belarus
North Korea
Australia
Finland
Mongolia
Yemen
Netherlands
Israel
Nigeria
Italy
Panama
Comoros
Hungary
Lithuania
Cuba
Montenegro
Georgia
Bolivia
Latvia
Jamaica
Mexico
Pakistan
Ukraine
Canada
Macedonia
St. Lucia
Uruguay
Nicaragua
France
South Sudan
United States
Suriname
Argentina
Japan
Barbados
Côte d'Ivoire
Bulgaria
Papua New Guinea
Fiji
Paraguay
Mali
Kuwait
Vanuatu
Venezuela
Moldova
Bahamas
0
50
100
150
200
250
Percentage change in YLLs averted per 1000 vaccinated girls

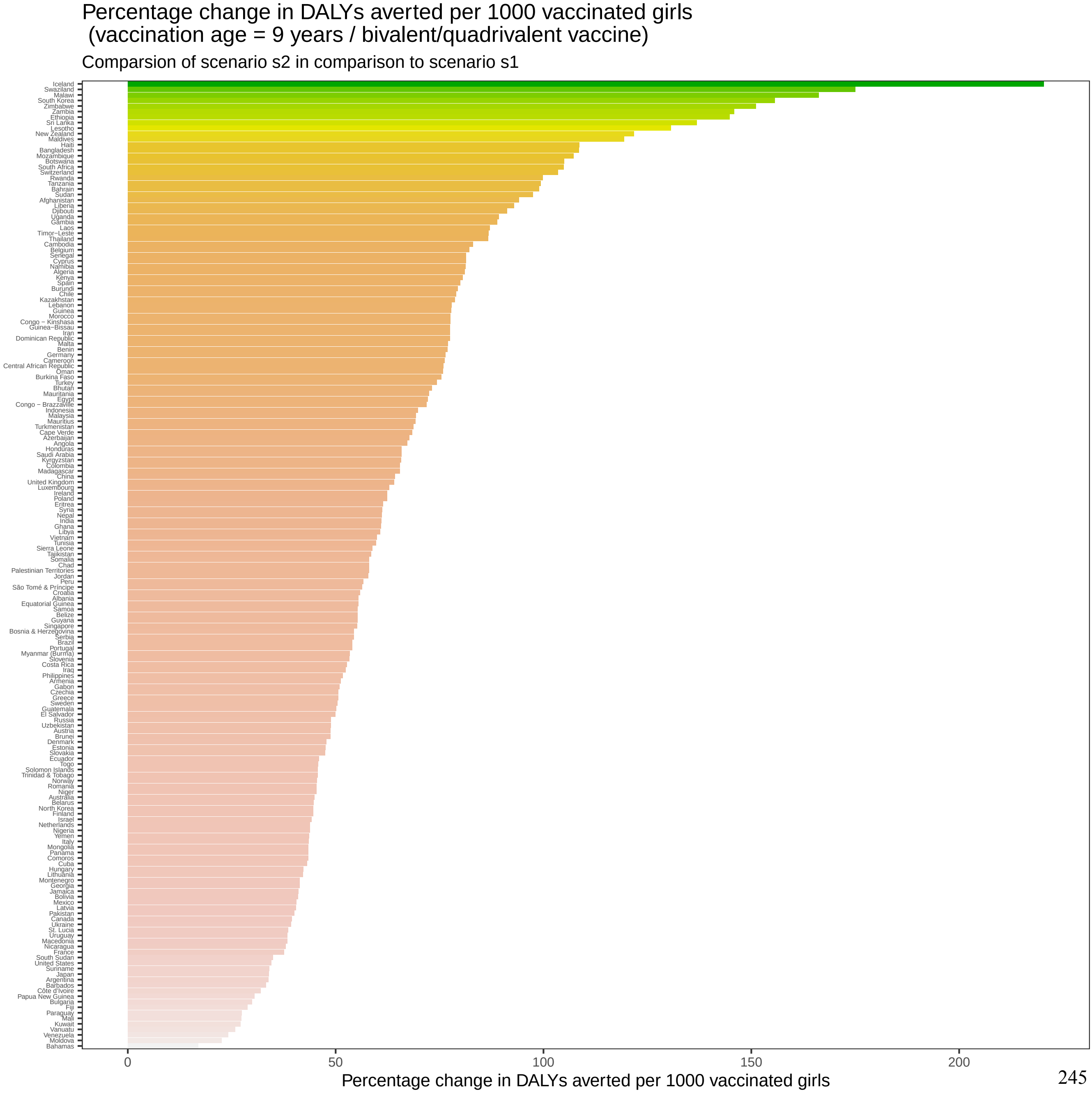

Percentage change in DALYs averted per 1000 vaccinated girls
(vaccination age = 9 years / bivalent/quadrivalent vaccine)
Comparsion of scenario s2 in comparison to scenario s1
Iceland
Swaziland
Malawi
South Korea
Zimbabwe
Zambia
Ethiopia
Sri Lanka
Lesotho
New Zealand
Maldives
Haiti
Bangladesh
Mozambique
Botswana
South Africa
Switzerland
Rwanda
Tanzania
Bahrain
Sudan
Afghanistan
Liberia
Djibouti
Uganda
Gambia
Laos
Timor−Leste
Thailand
Cambodia
Belgium
Senegal
Cyprus
Namibia
Algeria
Kenya
Spain
Burundi
Chile
Kazakhstan
Lebanon
Guinea
Morocco
Congo − Kinshasa
Guinea−Bissau
Iran
Dominican Republic
Malta
Benin
Germany
Cameroon
Central African Republic
Oman
Burkina Faso
Turkey
Bhutan
Mauritania
Egypt
Congo − Brazzaville
Indonesia
Malaysia
Mauritius
Turkmenistan
Cape Verde
Azerbaijan
Angola
Honduras
Saudi Arabia
Kyrgyzstan
Colombia
Madagascar
China
United Kingdom
Luxembourg
Ireland
Poland
Eritrea
Syria
Nepal
India
Ghana
Libya
Vietnam
Tunisia
Sierra Leone
Tajikistan
Somalia
Chad
Palestinian Territories
Jordan
Peru
São Tomé & Príncipe
Croatia
Albania
Equatorial Guinea
Samoa
Belize
Guyana
Singapore
Bosnia & Herzegovina
Serbia
Brazil
Portugal
Myanmar (Burma)
Slovenia
Costa Rica
Iraq
Philippines
Armenia
Gabon
Czechia
Greece
Sweden
Guatemala
El Salvador
Russia
Uzbekistan
Austria
Brunei
Denmark
Estonia
Slovakia
Ecuador
Togo
Solomon Islands
Trinidad & Tobago
Norway
Romania
Niger
Australia
Belarus
North Korea
Finland
Israel
Netherlands
Nigeria
Yemen
Italy
Mongolia
Panama
Comoros
Cuba
Hungary
Lithuania
Montenegro
Georgia
Jamaica
Bolivia
Mexico
Latvia
Pakistan
Canada
Ukraine
St. Lucia
Uruguay
Macedonia
Nicaragua
France
South Sudan
United States
Suriname
Japan
Argentina
Barbados
Côte d'Ivoire
Papua New Guinea
Bulgaria
Fiji
Paraguay
Mali
Kuwait
Vanuatu
Venezuela
Moldova
Bahamas
0
50
100
150
200
Percentage change in DALYs averted per 1000 vaccinated girls

Percentage change in cases averted per 1000 vaccinated girls
(vaccination age = 9 years / bivalent/quadrivalent vaccine)

Comparsion of scenario s3 in comparison to scenario s1

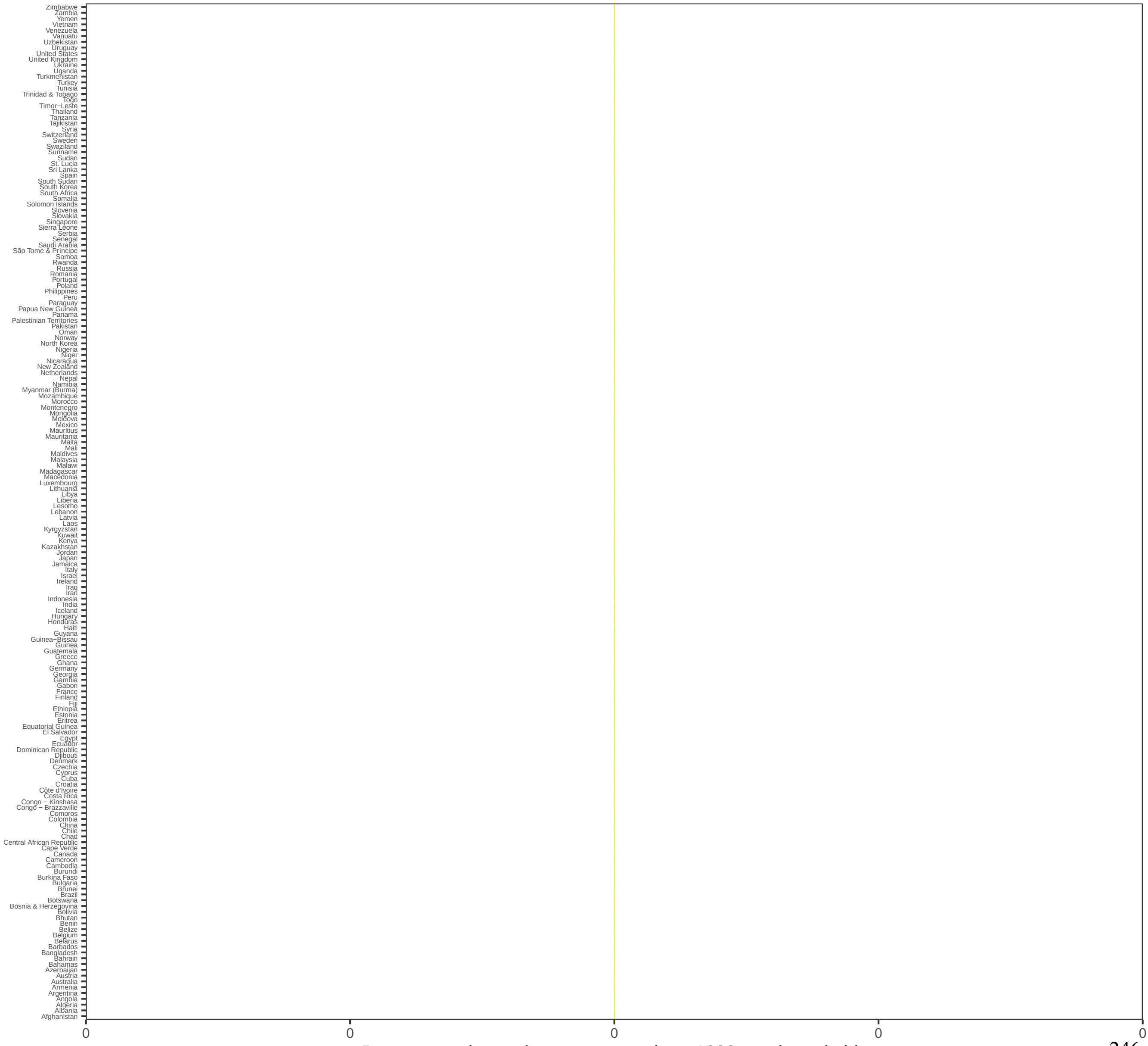

Percentage change in deaths averted per 1000 vaccinated girls
(vaccination age = 9 years / bivalent/quadrivalent vaccine)

Comparsion of scenario s3 in comparison to scenario s1

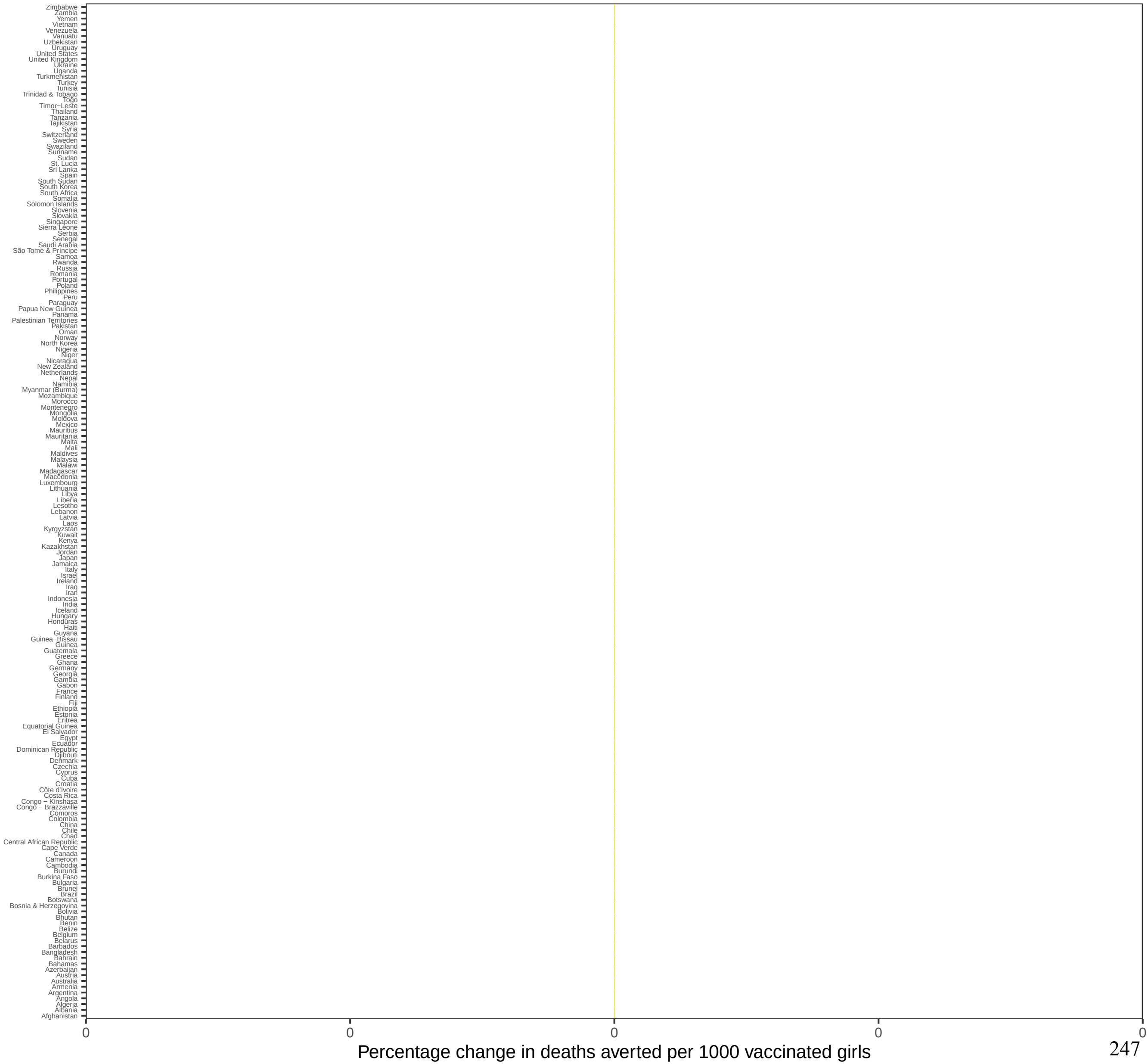

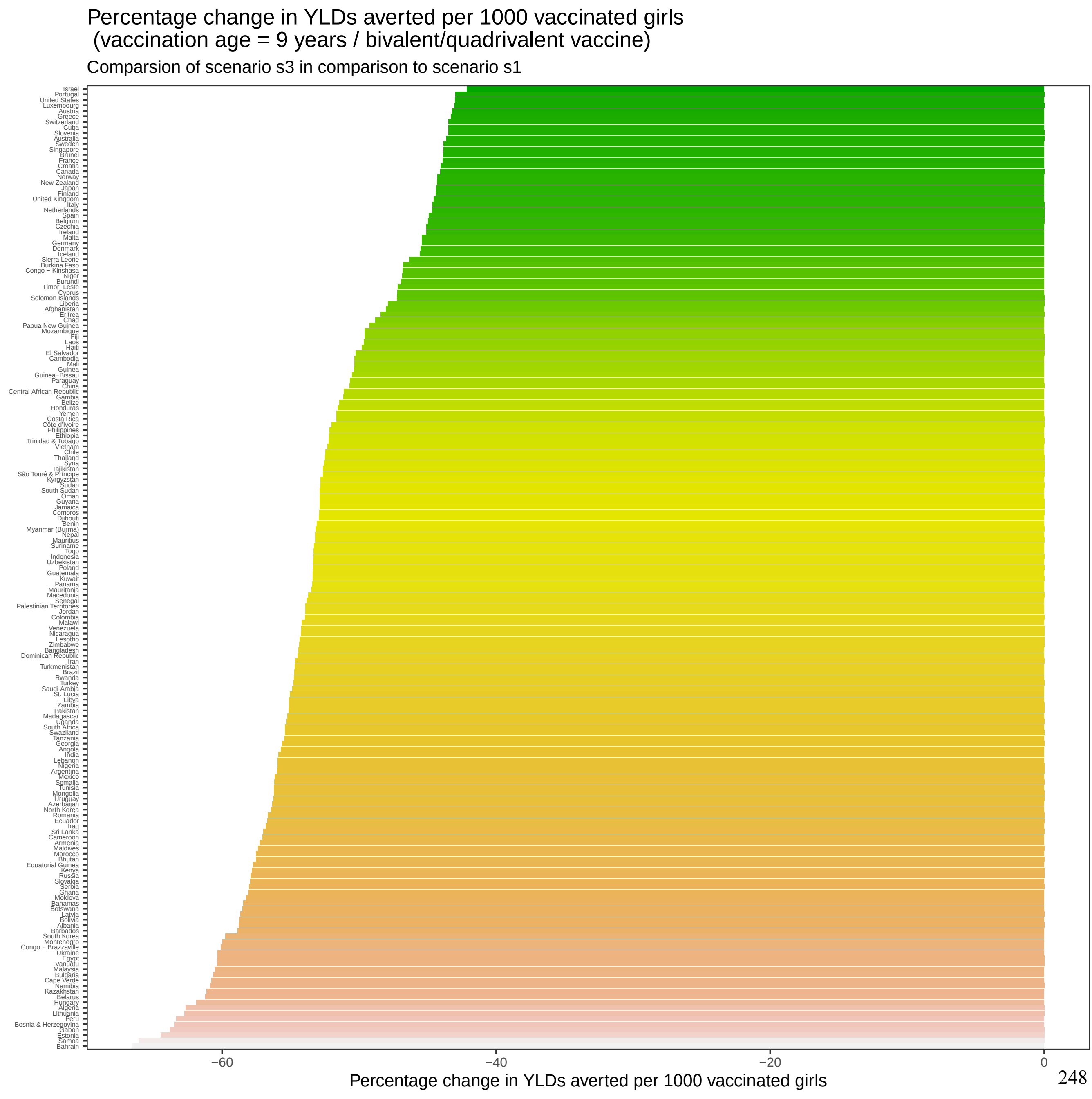
Percentage change in YLDs averted per 1000 vaccinated girls
(vaccination age = 9 years / bivalent/quadrivalent vaccine)
Comparsion of scenario s3 in comparison to scenario s1
Percentage change in YLDs averted per 1000 vaccinated girls
−60
−40
−20
0

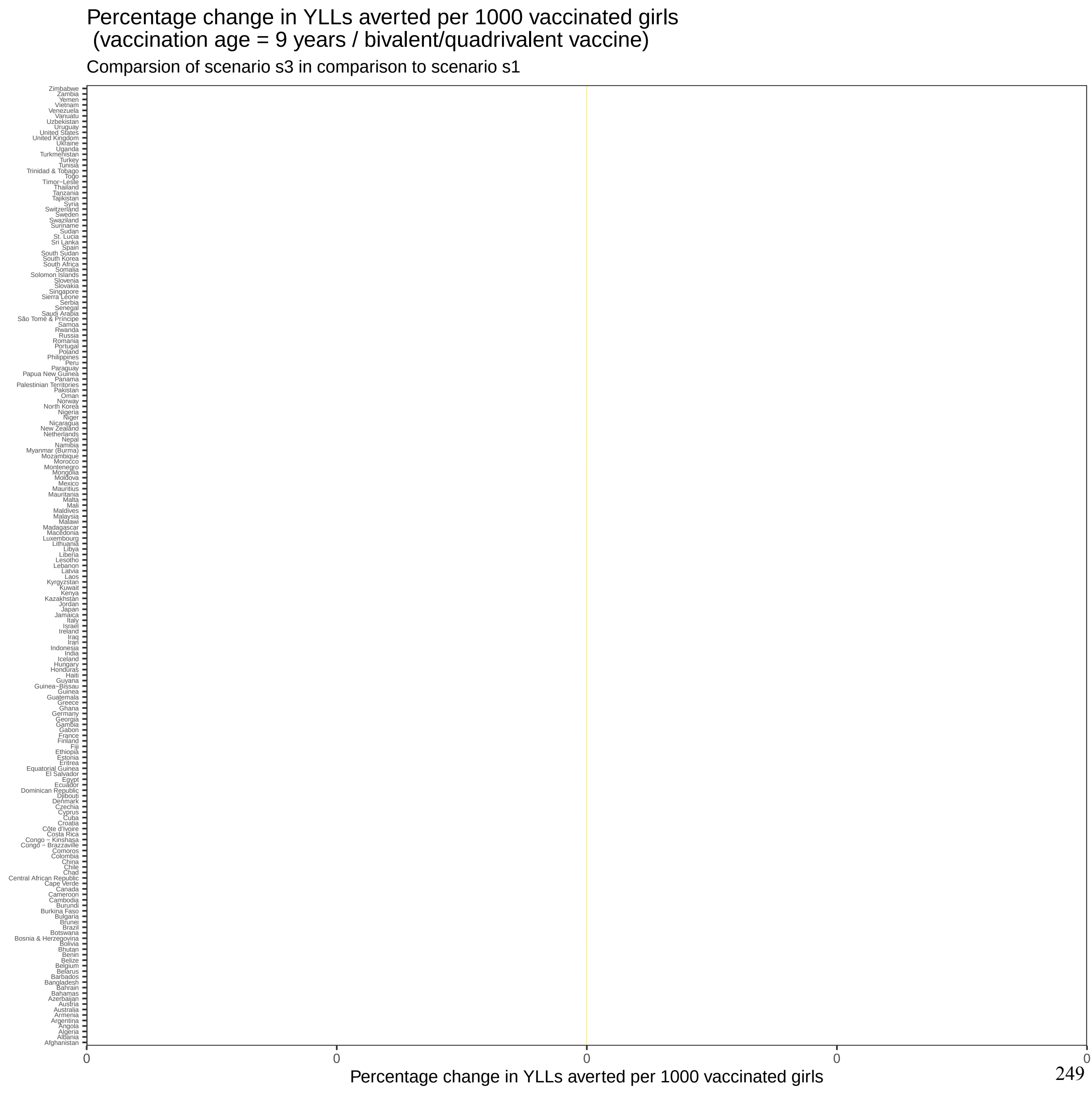
Percentage change in YLLs averted per 1000 vaccinated girls
(vaccination age = 9 years / bivalent/quadrivalent vaccine)
Comparsion of scenario s3 in comparison to scenario s1
Zimbabwe
Zambia
Yemen
Vietnam
Venezuela
Vanuatu
Uzbekistan
Uruguay
United States
United Kingdom
Ukraine
Uganda
Turkmenistan
Turkey
Tunisia
Trinidad & Tobago
Togo
Timor−Leste
Thailand
Tanzania
Tajikistan
Syria
Switzerland
Sweden
Swaziland
Suriname
Sudan
St. Lucia
Sri Lanka
Spain
South Sudan
South Korea
South Africa
Somalia
Solomon Islands
Slovenia
Slovakia
Singapore
Sierra Leone
Serbia
Senegal
Saudi Arabia
São Tomé & Príncipe
Samoa
Rwanda
Russia
Romania
Portugal
Poland
Philippines
Peru
Paraguay
Papua New Guinea
Panama
Palestinian Territories
Pakistan
Oman
Norway
North Korea
Nigeria
Niger
Nicaragua
New Zealand
Netherlands
Nepal
Namibia
Myanmar (Burma)
Mozambique
Morocco
Montenegro
Mongolia
Moldova
Mexico
Mauritius
Mauritania
Malta
Mali
Maldives
Malaysia
Malawi
Madagascar
Macedonia
Luxembourg
Lithuania
Libya
Liberia
Lesotho
Lebanon
Latvia
Laos
Kyrgyzstan
Kuwait
Kenya
Kazakhstan
Jordan
Japan
Jamaica
Italy
Israel
Ireland
Iraq
Iran
Indonesia
India
Iceland
Hungary
Honduras
Haiti
Guyana
Guinea−Bissau
Guinea
Guatemala
Greece
Ghana
Germany
Georgia
Gambia
Gabon
France
Finland
Fiji
Ethiopia
Estonia
Eritrea
Equatorial Guinea
El Salvador
Egypt
Ecuador
Dominican Republic
Djibouti
Denmark
Czechia
Cyprus
Cuba
Croatia
Côte d'Ivoire
Costa Rica
Congo − Kinshasa
Congo − Brazzaville
Comoros
Colombia
China
Chile
Chad
Central African Republic
Cape Verde
Canada
Cameroon
Cambodia
Burundi
Burkina Faso
Bulgaria
Brunei
Brazil
Botswana
Bosnia & Herzegovina
Bolivia
Bhutan
Benin
Belize
Belgium
Belarus
Barbados
Bangladesh
Bahrain
Bahamas
Azerbaijan
Austria
Australia
Armenia
Argentina
Angola
Algeria
Albania
Afghanistan
0
0
0
0
0
Percentage change in YLLs averted per 1000 vaccinated girls

Percentage change in DALYs averted per 1000 vaccinated girls
 (vaccination age = 9 years / bivalent/quadrivalent vaccine)

Comparsion of scenario s3 in comparison to scenario s1

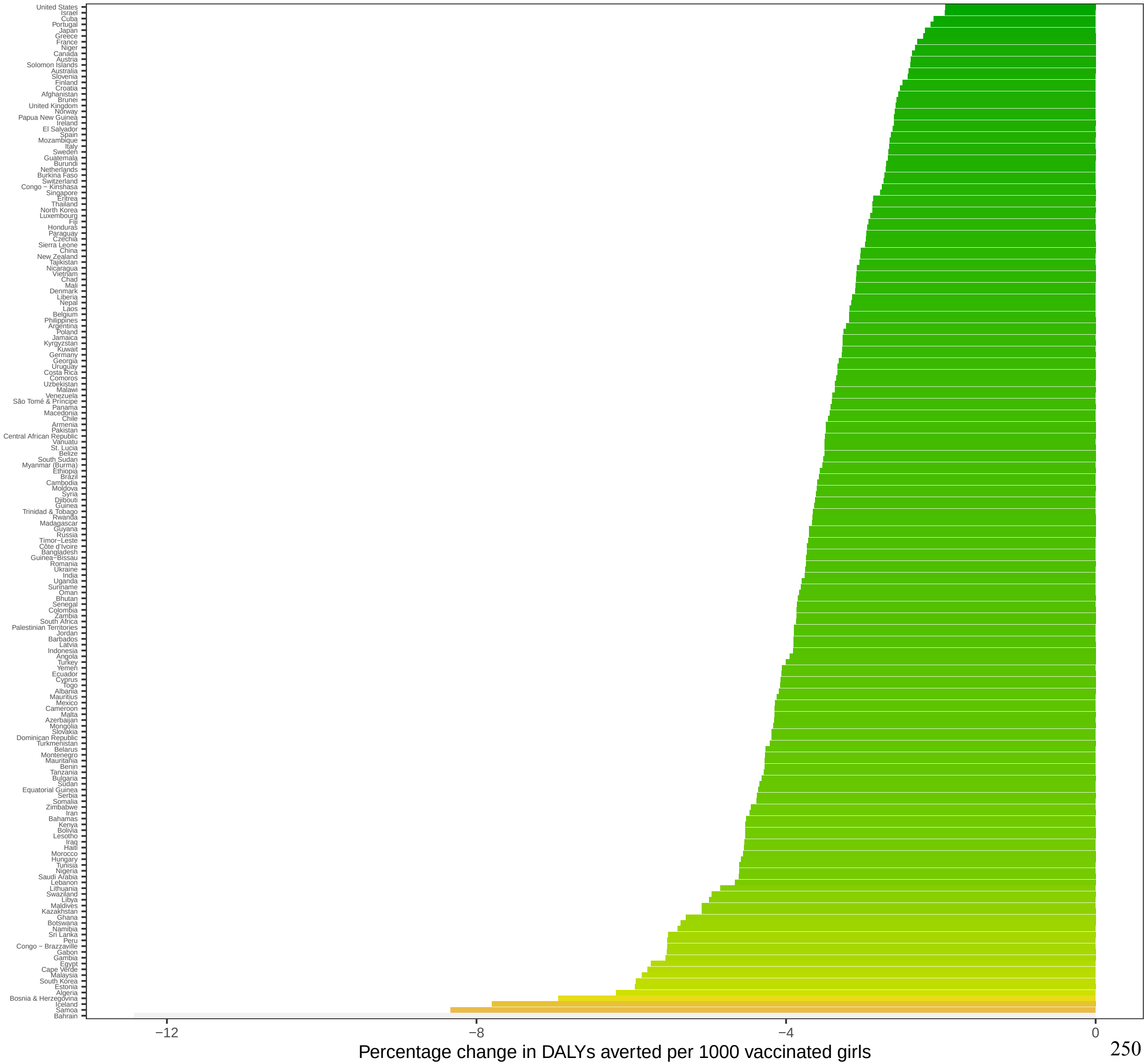

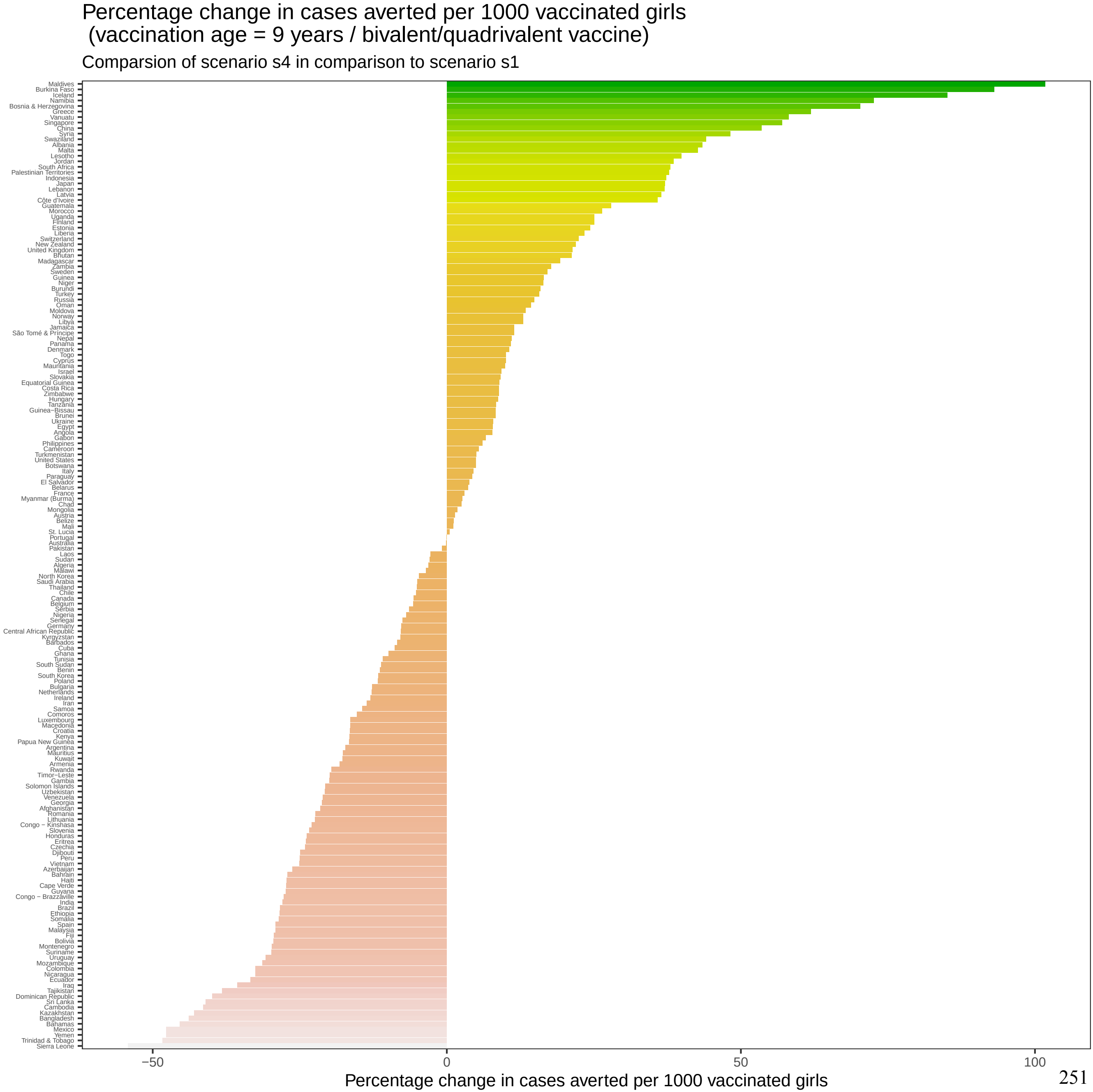
Percentage change in cases averted per 1000 vaccinated girls
(vaccination age = 9 years / bivalent/quadrivalent vaccine)
Comparsion of scenario s4 in comparison to scenario s1
Maldives
Burkina Faso
Iceland
Namibia
Bosnia & Herzegovina
Greece
Vanuatu
Singapore
China
Syria
Swaziland
Albania
Malta
Lesotho
Jordan
South Africa
Palestinian Territories
Indonesia
Japan
Lebanon
Latvia
Côte d'Ivoire
Guatemala
Morocco
Uganda
Finland
Estonia
Liberia
Switzerland
New Zealand
United Kingdom
Bhutan
Madagascar
Zambia
Sweden
Guinea
Niger
Burundi
Turkey
Russia
Oman
Moldova
Norway
Libya
Jamaica
São Tomé & Príncipe
Nepal
Panama
Denmark
Togo
Cyprus
Mauritania
Israel
Slovakia
Equatorial Guinea
Costa Rica
Zimbabwe
Hungary
Tanzania
Guinea−Bissau
Brunei
Ukraine
Egypt
Angola
Gabon
Philippines
Cameroon
Turkmenistan
United States
Botswana
Italy
Paraguay
El Salvador
Belarus
France
Myanmar (Burma)
Chad
Mongolia
Austria
Belize
Mali
St. Lucia
Portugal
Australia
Pakistan
Laos
Sudan
Algeria
Malawi
North Korea
Saudi Arabia
Thailand
Chile
Canada
Belgium
Serbia
Nigeria
Senegal
Germany
Central African Republic
Kyrgyzstan
Barbados
Cuba
Ghana
Tunisia
South Sudan
Benin
South Korea
Poland
Bulgaria
Netherlands
Ireland
Iran
Samoa
Comoros
Luxembourg
Macedonia
Croatia
Kenya
Papua New Guinea
Argentina
Mauritius
Kuwait
Armenia
Rwanda
Timor−Leste
Gambia
Solomon Islands
Uzbekistan
Venezuela
Georgia
Afghanistan
Romania
Lithuania
Congo − Kinshasa
Slovenia
Honduras
Eritrea
Czechia
Djibouti
Peru
Vietnam
Azerbaijan
Bahrain
Haiti
Cape Verde
Guyana
Congo − Brazzaville
India
Brazil
Ethiopia
Somalia
Spain
Malaysia
Fiji
Bolivia
Montenegro
Suriname
Uruguay
Mozambique
Colombia
Nicaragua
Ecuador
Iraq
Tajikistan
Dominican Republic
Sri Lanka
Cambodia
Kazakhstan
Bangladesh
Bahamas
Mexico
Yemen
Trinidad & Tobago
Sierra Leone
−50
0
50
100
Percentage change in cases averted per 1000 vaccinated girls

Percentage change in deaths averted per 1000 vaccinated girls
(vaccination age = 9 years / bivalent/quadrivalent vaccine)

Comparsion of scenario s4 in comparison to scenario s1

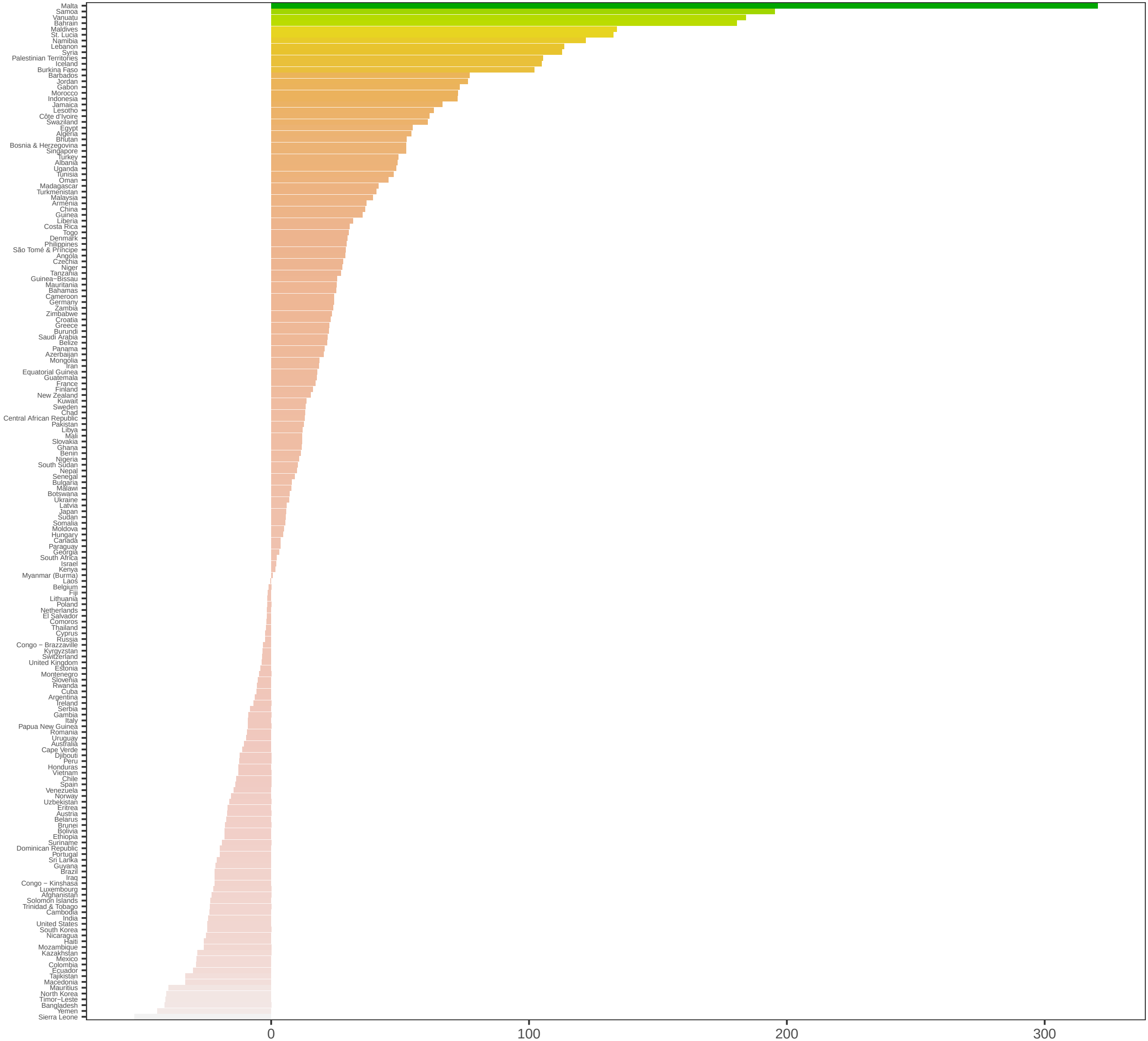

Percentage change in deaths averted per 1000 vaccinated girls

Percentage change in YLDs averted per 1000 vaccinated girls (vaccination age = 9 years / bivalent/quadrivalent vaccine)

Comparsion of scenario s4 in comparison to scenario s1

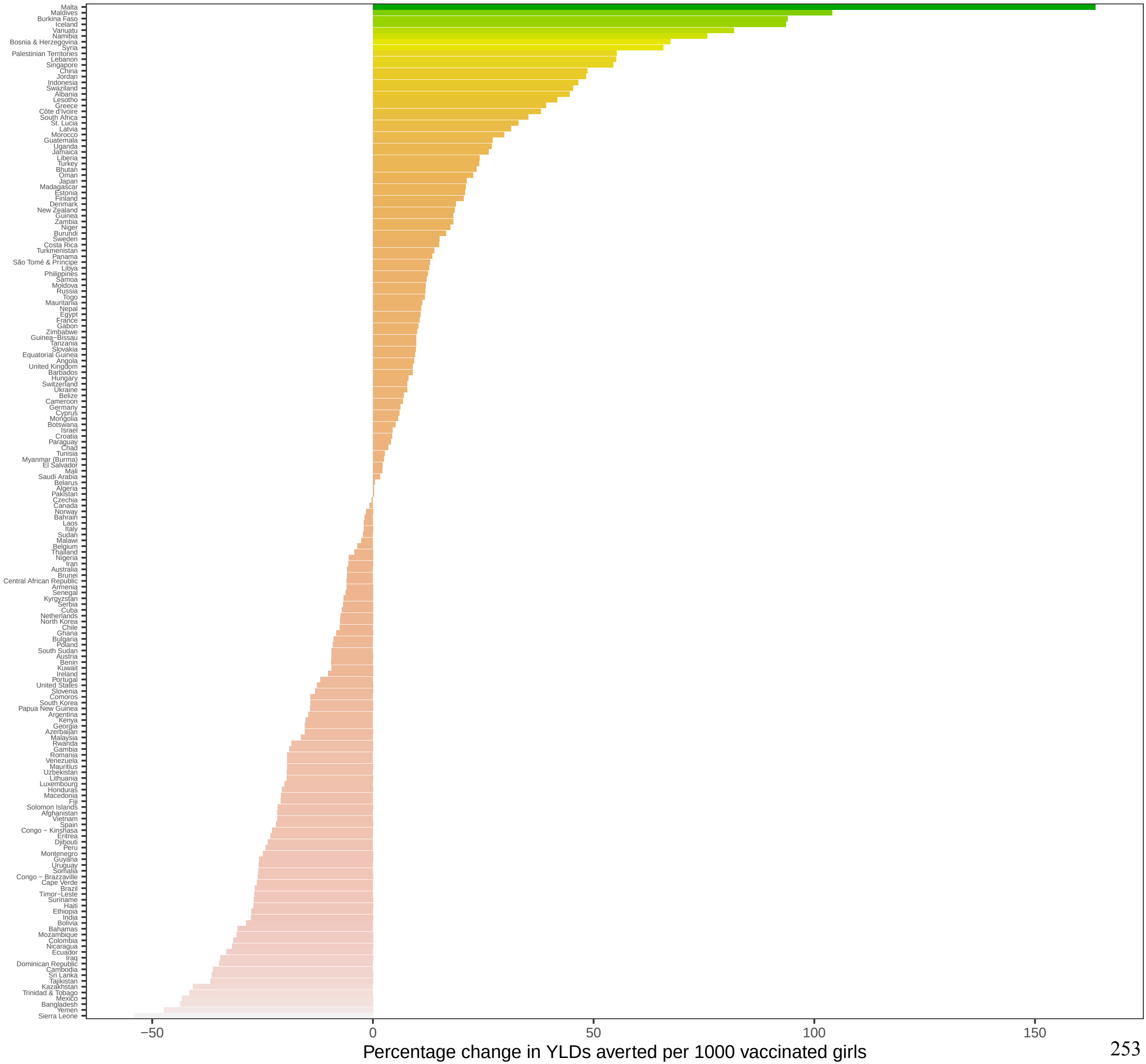

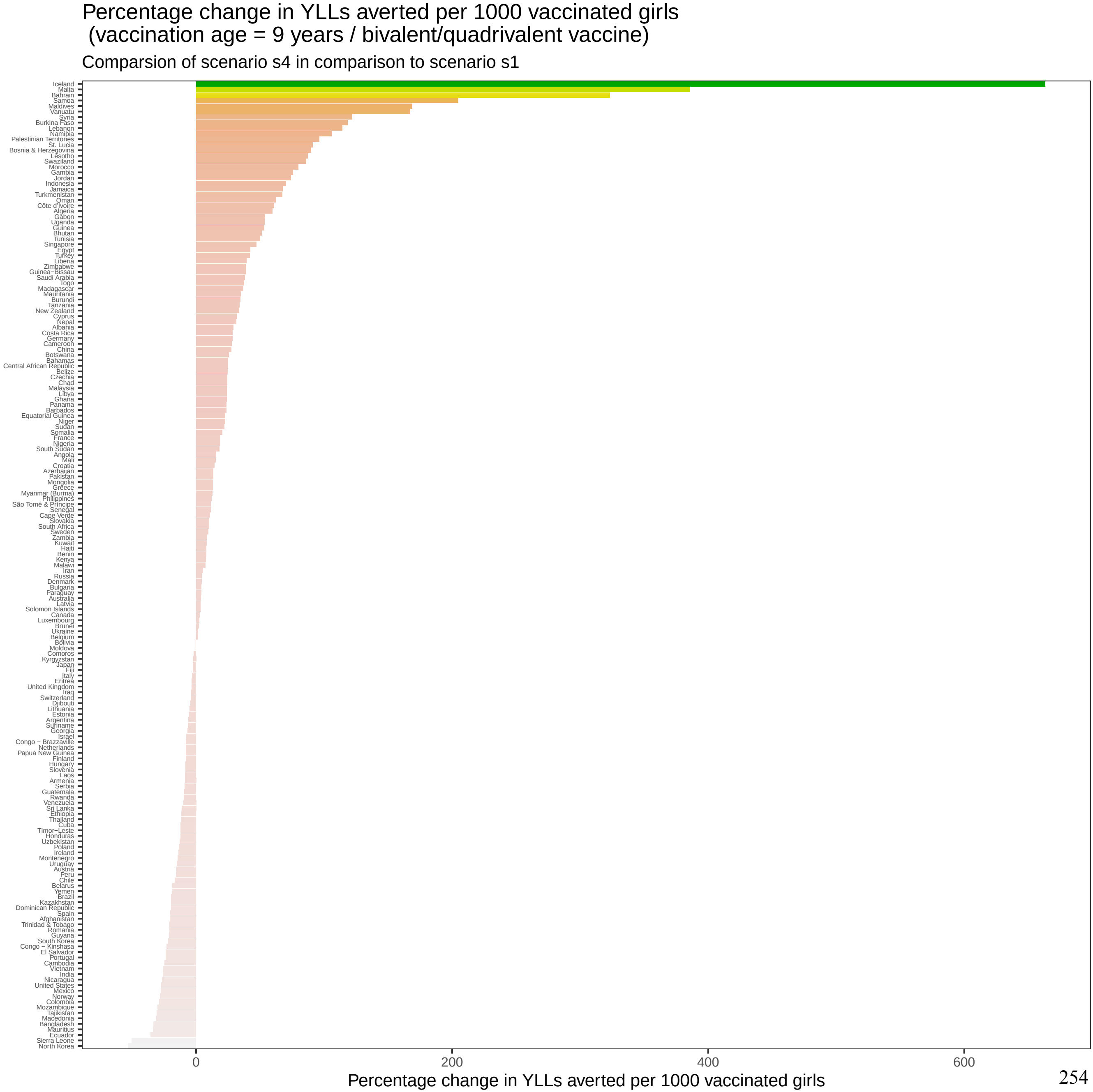
Percentage change in YLLs averted per 1000 vaccinated girls
(vaccination age = 9 years / bivalent/quadrivalent vaccine)
Comparsion of scenario s4 in comparison to scenario s1
Iceland
Malta
Bahrain
Samoa
Maldives
Vanuatu
Syria
Burkina Faso
Lebanon
Namibia
Palestinian Territories
St. Lucia
Bosnia & Herzegovina
Lesotho
Swaziland
Morocco
Gambia
Jordan
Indonesia
Jamaica
Turkmenistan
Oman
Côte d'Ivoire
Algeria
Gabon
Uganda
Guinea
Bhutan
Tunisia
Singapore
Egypt
Turkey
Liberia
Zimbabwe
Guinea−Bissau
Saudi Arabia
Togo
Madagascar
Mauritania
Burundi
Tanzania
New Zealand
Cyprus
Nepal
Albania
Costa Rica
Germany
Cameroon
China
Botswana
Bahamas
Central African Republic
Belize
Czechia
Chad
Malaysia
Libya
Ghana
Panama
Barbados
Equatorial Guinea
Niger
Sudan
Somalia
France
Nigeria
South Sudan
Angola
Mali
Croatia
Azerbaijan
Pakistan
Mongolia
Greece
Myanmar (Burma)
Philippines
São Tomé & Príncipe
Senegal
Cape Verde
Slovakia
South Africa
Sweden
Zambia
Kuwait
Haiti
Benin
Kenya
Malawi
Iran
Russia
Denmark
Bulgaria
Paraguay
Australia
Latvia
Solomon Islands
Canada
Luxembourg
Brunei
Ukraine
Belgium
Bolivia
Moldova
Comoros
Kyrgyzstan
Japan
Fiji
Italy
Eritrea
United Kingdom
Iraq
Switzerland
Djibouti
Lithuania
Estonia
Argentina
Suriname
Georgia
Israel
Congo − Brazzaville
Netherlands
Papua New Guinea
Finland
Hungary
Slovenia
Laos
Armenia
Serbia
Guatemala
Rwanda
Venezuela
Sri Lanka
Ethiopia
Thailand
Cuba
Timor−Leste
Honduras
Uzbekistan
Poland
Ireland
Montenegro
Uruguay
Austria
Peru
Chile
Belarus
Yemen
Brazil
Kazakhstan
Dominican Republic
Spain
Afghanistan
Trinidad & Tobago
Romania
Guyana
South Korea
Congo − Kinshasa
El Salvador
Portugal
Cambodia
Vietnam
India
Nicaragua
United States
Mexico
Norway
Colombia
Mozambique
Tajikistan
Macedonia
Bangladesh
Mauritius
Ecuador
Sierra Leone
North Korea
0
200
400
600
Percentage change in YLLs averted per 1000 vaccinated girls

Percentage change in DALYs averted per 1000 vaccinated girls
(vaccination age = 9 years / bivalent/quadrivalent vaccine)

Comparsion of scenario s4 in comparison to scenario s1

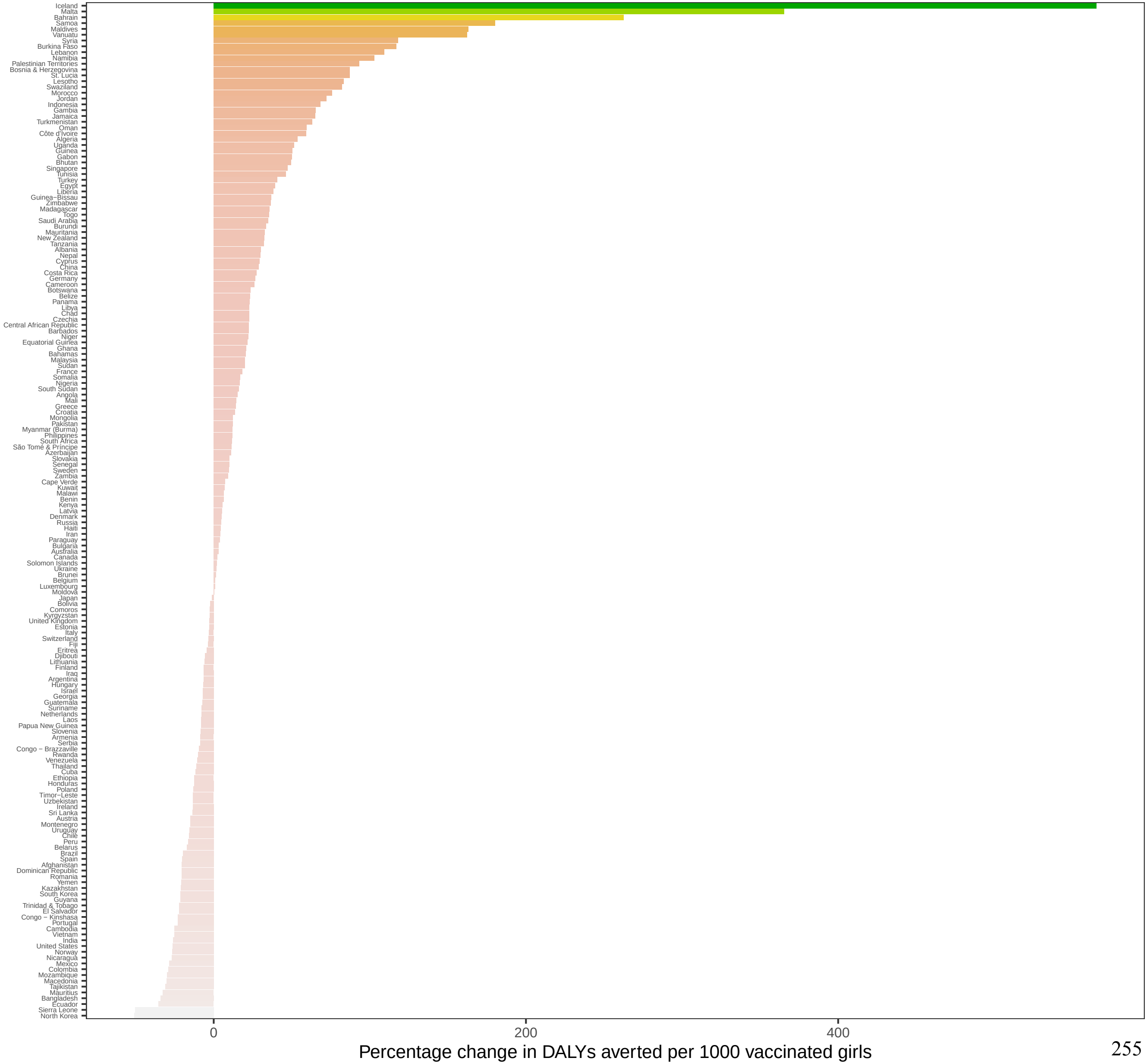

Percentage change in cases averted per 1000 vaccinated girls
(vaccination age = 9 years / bivalent/quadrivalent vaccine)

Comparsion of scenario s5 in comparison to scenario s1

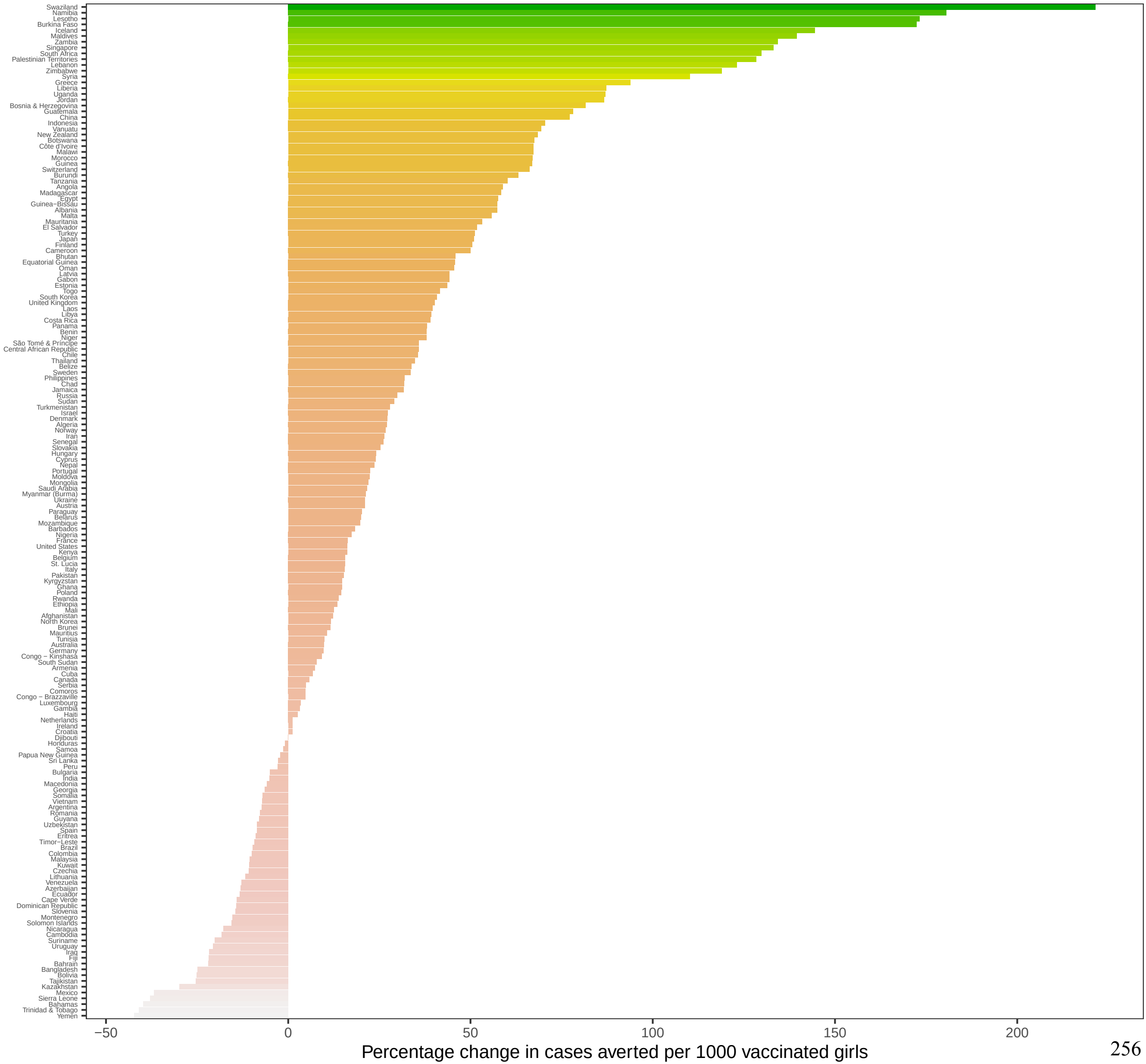

Percentage change in deaths averted per 1000 vaccinated girls
(vaccination age = 9 years / bivalent/quadrivalent vaccine)

Comparsion of scenario s5 in comparison to scenario s1

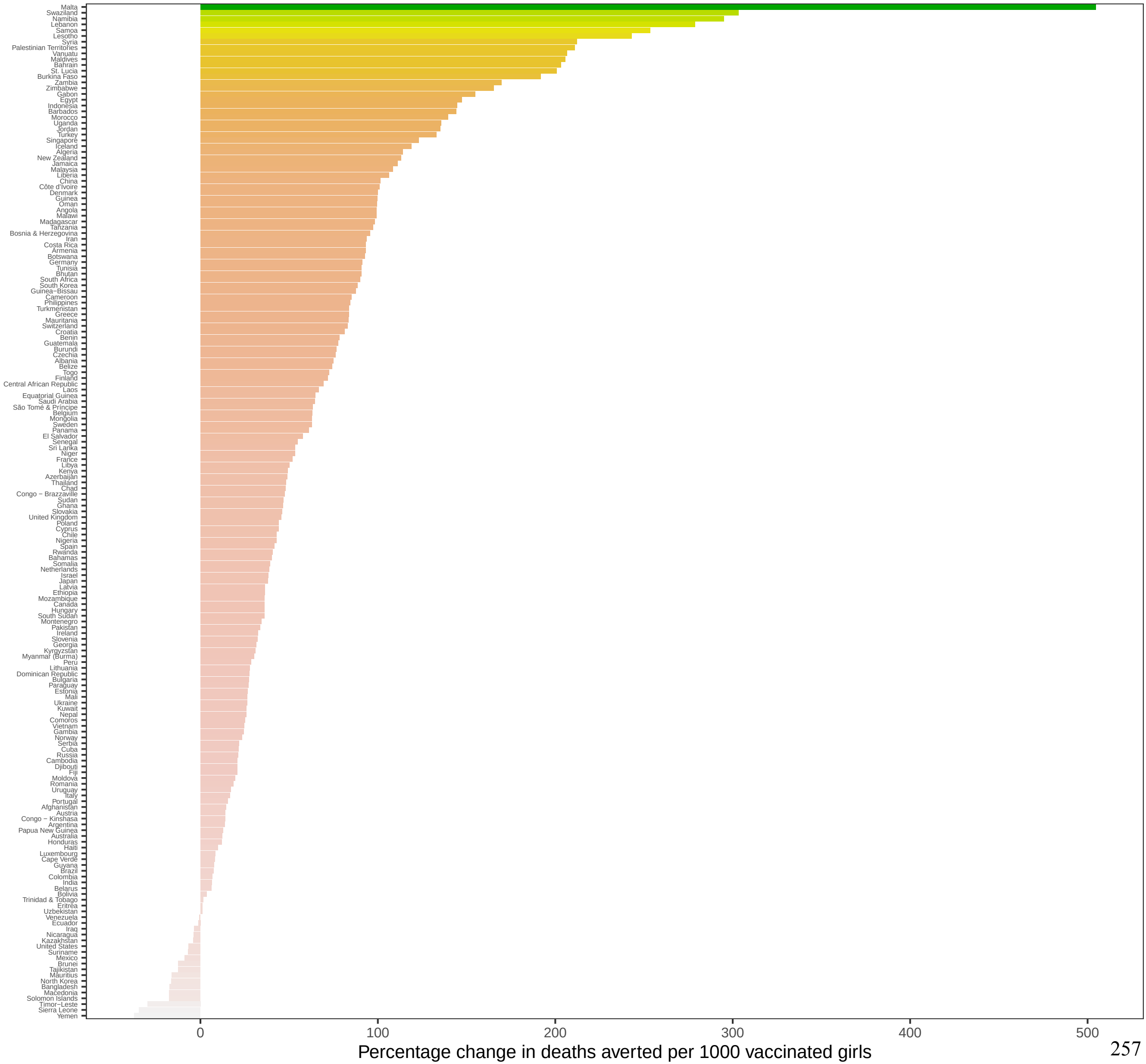

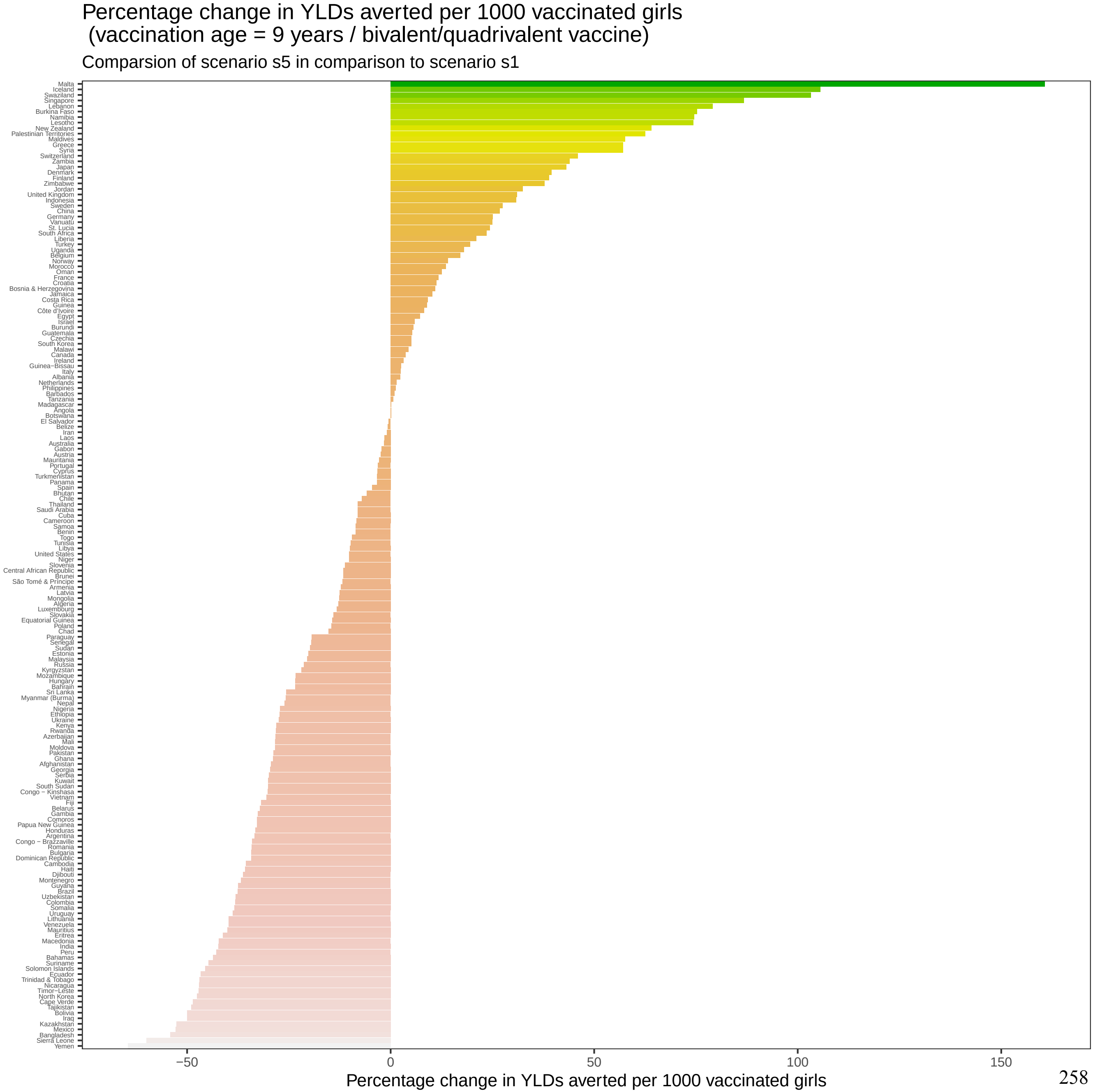

Percentage change in YLDs averted per 1000 vaccinated girls
(vaccination age = 9 years / bivalent/quadrivalent vaccine)
Comparsion of scenario s5 in comparison to scenario s1
Malta
Iceland
Swaziland
Singapore
Lebanon
Burkina Faso
Namibia
Lesotho
New Zealand
Palestinian Territories
Maldives
Greece
Syria
Switzerland
Zambia
Japan
Denmark
Finland
Zimbabwe
Jordan
United Kingdom
Indonesia
Sweden
China
Germany
Vanuatu
St. Lucia
South Africa
Liberia
Turkey
Uganda
Belgium
Norway
Morocco
Oman
France
Croatia
Bosnia & Herzegovina
Jamaica
Costa Rica
Guinea
Côte d'Ivoire
Egypt
Israel
Burundi
Guatemala
Czechia
South Korea
Malawi
Canada
Ireland
Guinea−Bissau
Italy
Albania
Netherlands
Philippines
Barbados
Tanzania
Madagascar
Angola
Botswana
El Salvador
Belize
Iran
Laos
Australia
Gabon
Austria
Mauritania
Portugal
Cyprus
Turkmenistan
Panama
Spain
Bhutan
Chile
Thailand
Saudi Arabia
Cuba
Cameroon
Samoa
Benin
Togo
Tunisia
Libya
United States
Niger
Slovenia
Central African Republic
Brunei
São Tomé & Príncipe
Armenia
Latvia
Mongolia
Algeria
Luxembourg
Slovakia
Equatorial Guinea
Poland
Chad
Paraguay
Senegal
Sudan
Estonia
Malaysia
Russia
Kyrgyzstan
Mozambique
Hungary
Bahrain
Sri Lanka
Myanmar (Burma)
Nepal
Nigeria
Ethiopia
Ukraine
Kenya
Rwanda
Azerbaijan
Mali
Moldova
Pakistan
Ghana
Afghanistan
Georgia
Serbia
Kuwait
South Sudan
Congo − Kinshasa
Vietnam
Fiji
Belarus
Gambia
Comoros
Papua New Guinea
Honduras
Argentina
Congo − Brazzaville
Romania
Bulgaria
Dominican Republic
Cambodia
Haiti
Djibouti
Montenegro
Guyana
Brazil
Uzbekistan
Colombia
Somalia
Uruguay
Lithuania
Venezuela
Mauritius
Eritrea
Macedonia
India
Peru
Bahamas
Suriname
Solomon Islands
Ecuador
Trinidad & Tobago
Nicaragua
Timor−Leste
North Korea
Cape Verde
Tajikistan
Bolivia
Iraq
Kazakhstan
Mexico
Bangladesh
Sierra Leone
Yemen
−50
0
50
100
150
Percentage change in YLDs averted per 1000 vaccinated girls

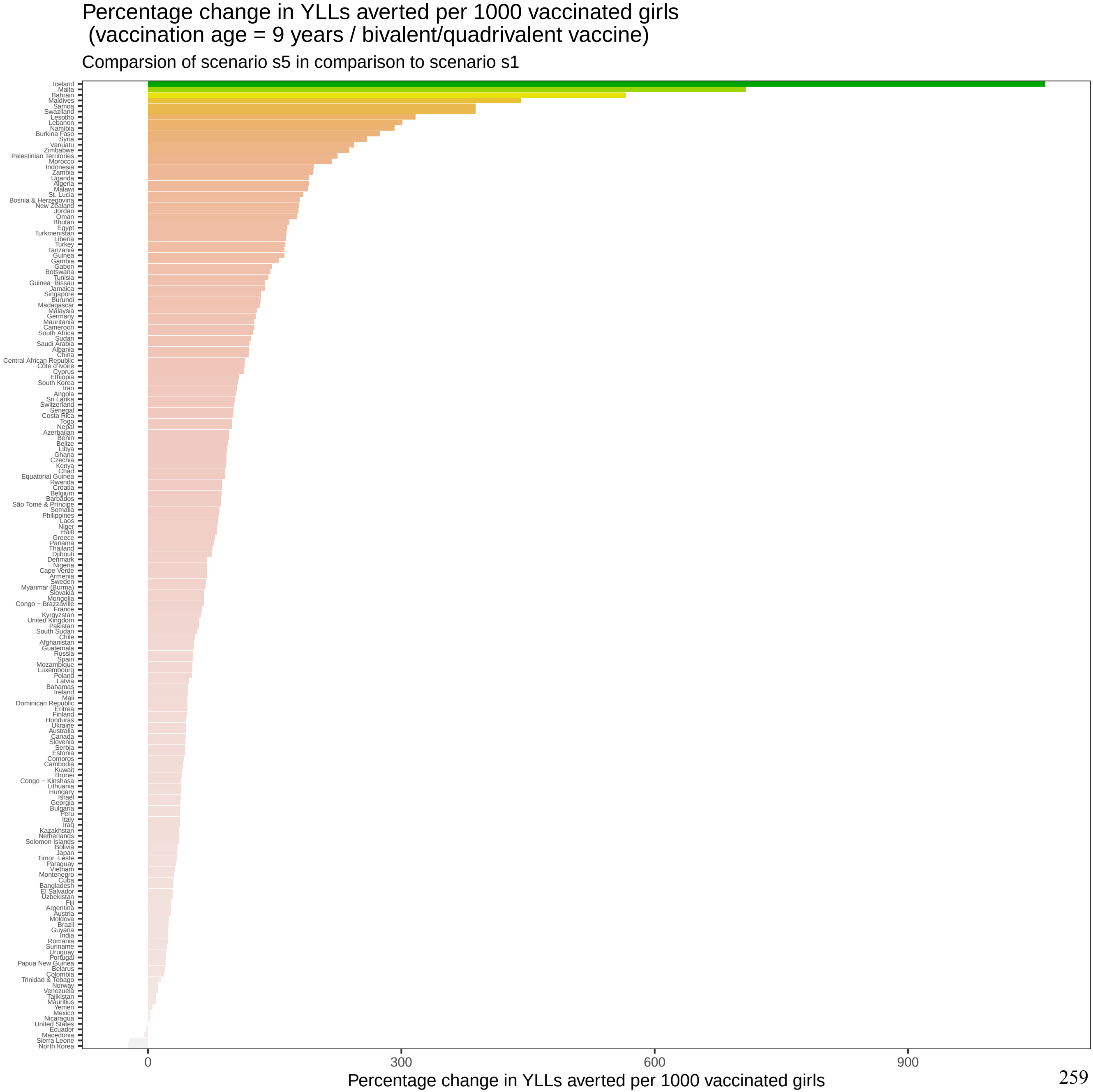
Percentage change in YLLs averted per 1000 vaccinated girls
(vaccination age = 9 years / bivalent/quadrivalent vaccine)
Comparsion of scenario s5 in comparison to scenario s1
Iceland
Malta
Bahrain
Maldives
Samoa
Swaziland
Lesotho
Lebanon
Namibia
Burkina Faso
Syria
Vanuatu
Zimbabwe
Palestinian Territories
Morocco
Indonesia
Zambia
Uganda
Algeria
Malawi
St. Lucia
Bosnia & Herzegovina
New Zealand
Jordan
Oman
Bhutan
Egypt
Turkmenistan
Liberia
Turkey
Tanzania
Guinea
Gambia
Gabon
Botswana
Tunisia
Guinea−Bissau
Jamaica
Singapore
Burundi
Madagascar
Malaysia
Germany
Mauritania
Cameroon
South Africa
Sudan
Saudi Arabia
Albania
China
Central African Republic
Côte d'Ivoire
Cyprus
Ethiopia
South Korea
Iran
Angola
Sri Lanka
Switzerland
Senegal
Costa Rica
Togo
Nepal
Azerbaijan
Benin
Belize
Libya
Ghana
Czechia
Kenya
Chad
Equatorial Guinea
Rwanda
Croatia
Belgium
Barbados
São Tomé & Príncipe
Somalia
Philippines
Laos
Niger
Haiti
Greece
Panama
Thailand
Djibouti
Denmark
Nigeria
Cape Verde
Armenia
Sweden
Myanmar (Burma)
Slovakia
Mongolia
Congo − Brazzaville
France
Kyrgyzstan
United Kingdom
Pakistan
South Sudan
Chile
Afghanistan
Guatemala
Russia
Spain
Mozambique
Luxembourg
Poland
Latvia
Bahamas
Ireland
Mali
Dominican Republic
Eritrea
Finland
Honduras
Ukraine
Australia
Canada
Slovenia
Serbia
Estonia
Comoros
Cambodia
Kuwait
Brunei
Congo − Kinshasa
Lithuania
Hungary
Israel
Georgia
Bulgaria
Peru
Italy
Iraq
Kazakhstan
Netherlands
Solomon Islands
Bolivia
Japan
Timor−Leste
Paraguay
Vietnam
Montenegro
Cuba
Bangladesh
El Salvador
Uzbekistan
Fiji
Argentina
Austria
Moldova
Brazil
Guyana
India
Romania
Suriname
Uruguay
Portugal
Papua New Guinea
Belarus
Colombia
Trinidad & Tobago
Norway
Venezuela
Tajikistan
Mauritius
Yemen
Mexico
Nicaragua
United States
Ecuador
Macedonia
Sierra Leone
North Korea
0
300
600
900
Percentage change in YLLs averted per 1000 vaccinated girls

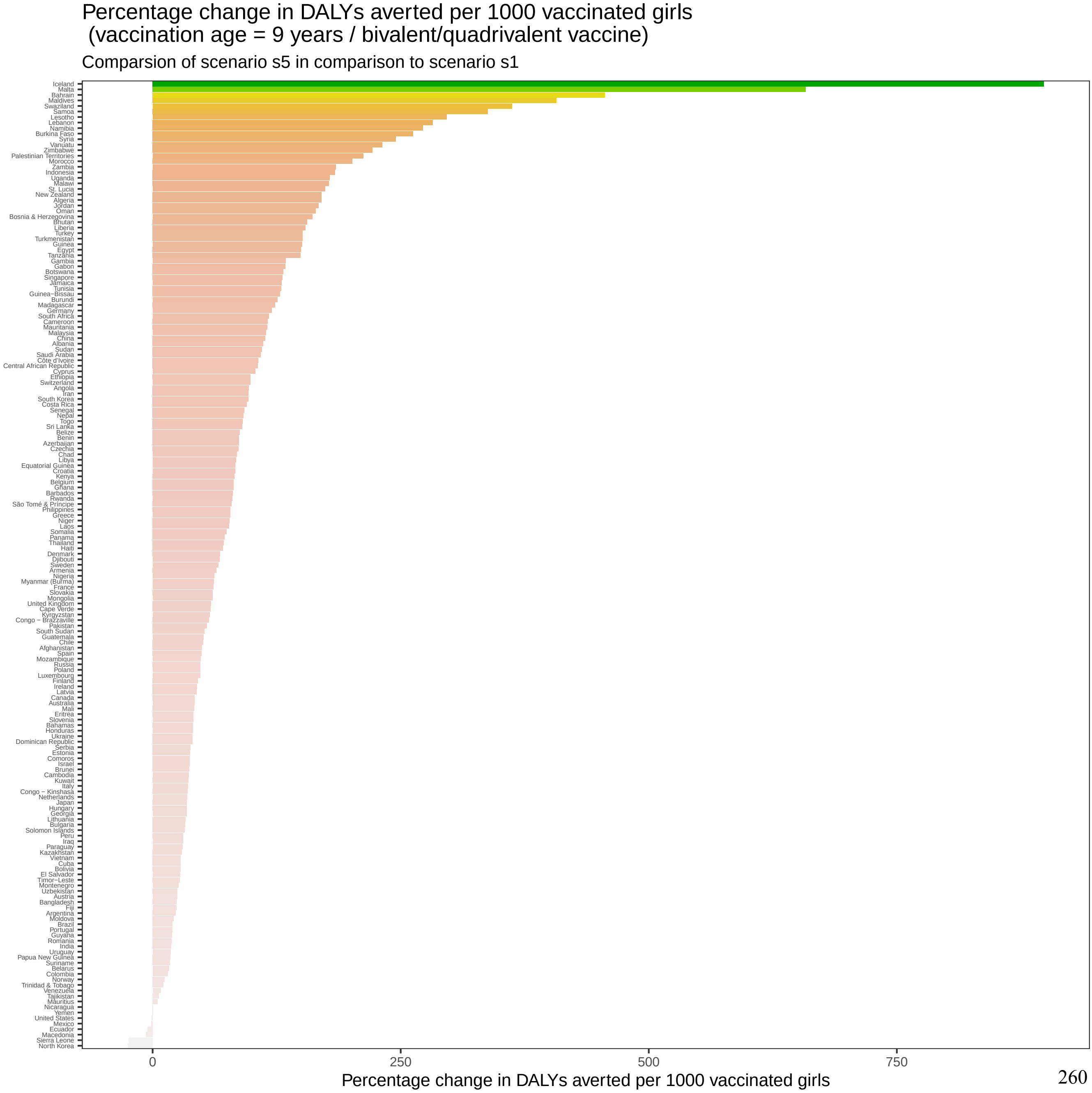
Percentage change in DALYs averted per 1000 vaccinated girls
(vaccination age = 9 years / bivalent/quadrivalent vaccine)
Comparsion of scenario s5 in comparison to scenario s1
Iceland
Malta
Bahrain
Maldives
Swaziland
Samoa
Lesotho
Lebanon
Namibia
Burkina Faso
Syria
Vanuatu
Zimbabwe
Palestinian Territories
Morocco
Zambia
Indonesia
Uganda
Malawi
St. Lucia
New Zealand
Algeria
Jordan
Oman
Bosnia & Herzegovina
Bhutan
Liberia
Turkey
Turkmenistan
Guinea
Egypt
Tanzania
Gambia
Gabon
Botswana
Singapore
Jamaica
Tunisia
Guinea−Bissau
Burundi
Madagascar
Germany
South Africa
Cameroon
Mauritania
Malaysia
China
Albania
Sudan
Saudi Arabia
Côte d'Ivoire
Central African Republic
Cyprus
Ethiopia
Switzerland
Angola
Iran
South Korea
Costa Rica
Senegal
Nepal
Togo
Sri Lanka
Belize
Benin
Azerbaijan
Czechia
Chad
Libya
Equatorial Guinea
Croatia
Kenya
Belgium
Ghana
Barbados
Rwanda
São Tomé & Príncipe
Philippines
Greece
Niger
Laos
Somalia
Panama
Thailand
Haiti
Denmark
Djibouti
Sweden
Armenia
Nigeria
Myanmar (Burma)
France
Slovakia
Mongolia
United Kingdom
Cape Verde
Kyrgyzstan
Congo − Brazzaville
Pakistan
South Sudan
Guatemala
Chile
Afghanistan
Spain
Mozambique
Russia
Poland
Luxembourg
Finland
Ireland
Latvia
Canada
Australia
Mali
Eritrea
Slovenia
Bahamas
Honduras
Ukraine
Dominican Republic
Serbia
Estonia
Comoros
Israel
Brunei
Cambodia
Kuwait
Italy
Congo − Kinshasa
Netherlands
Japan
Hungary
Georgia
Lithuania
Bulgaria
Solomon Islands
Peru
Iraq
Paraguay
Kazakhstan
Vietnam
Cuba
Bolivia
El Salvador
Timor−Leste
Montenegro
Uzbekistan
Austria
Bangladesh
Fiji
Argentina
Moldova
Brazil
Portugal
Guyana
Romania
India
Uruguay
Papua New Guinea
Suriname
Belarus
Colombia
Norway
Trinidad & Tobago
Venezuela
Tajikistan
Mauritius
Nicaragua
Yemen
United States
Mexico
Ecuador
Macedonia
Sierra Leone
North Korea
0
250
500
750
Percentage change in DALYs averted per 1000 vaccinated girls

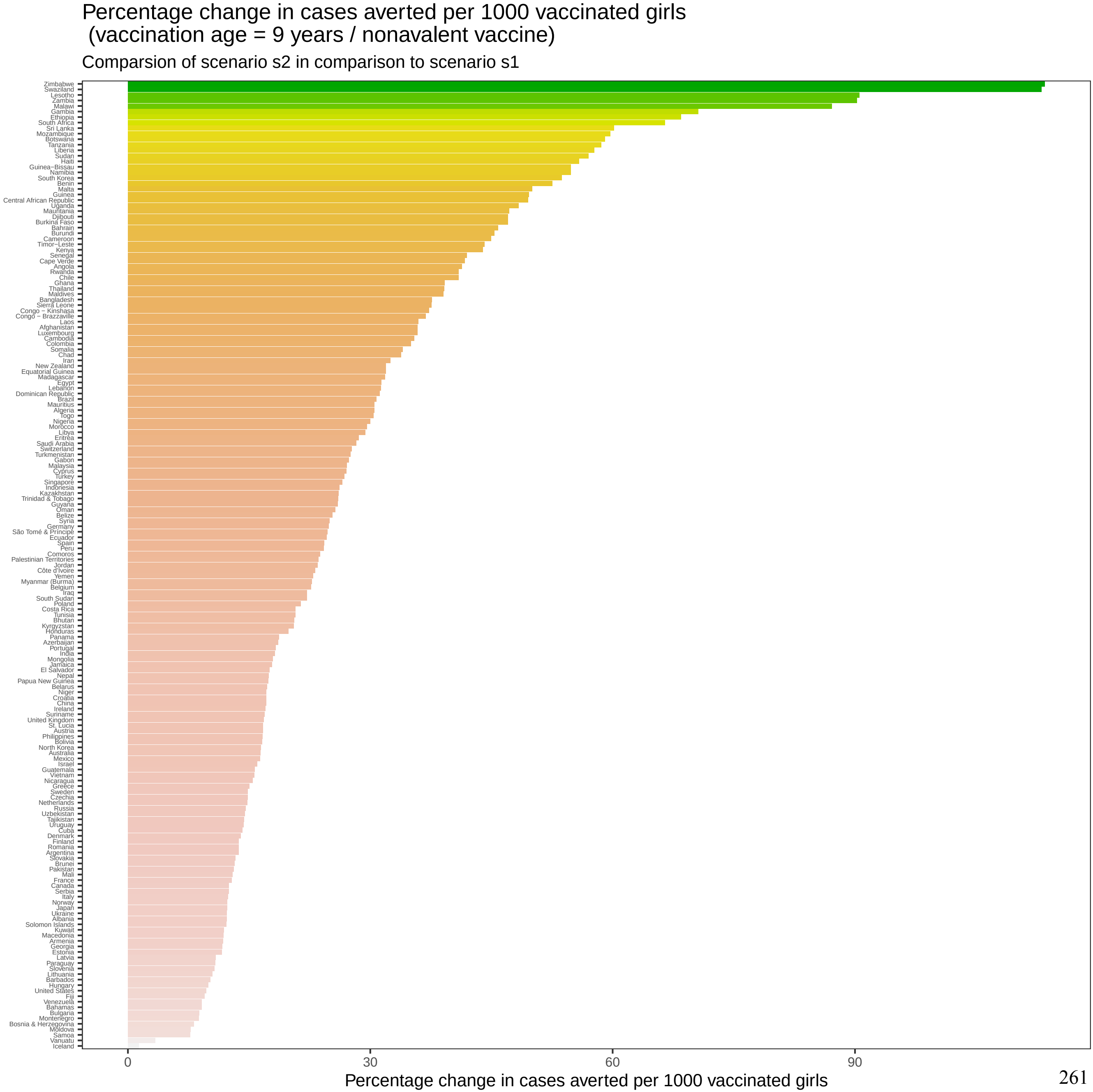
Percentage change in cases averted per 1000 vaccinated girls
(vaccination age = 9 years / nonavalent vaccine)
Comparsion of scenario s2 in comparison to scenario s1
Zimbabwe
Swaziland
Lesotho
Zambia
Malawi
Gambia
Ethiopia
South Africa
Sri Lanka
Mozambique
Botswana
Tanzania
Liberia
Sudan
Haiti
Guinea−Bissau
Namibia
South Korea
Benin
Malta
Guinea
Central African Republic
Uganda
Mauritania
Djibouti
Burkina Faso
Bahrain
Burundi
Cameroon
Timor−Leste
Kenya
Senegal
Cape Verde
Angola
Rwanda
Chile
Ghana
Thailand
Maldives
Bangladesh
Sierra Leone
Congo − Kinshasa
Congo − Brazzaville
Laos
Afghanistan
Luxembourg
Cambodia
Colombia
Somalia
Chad
Iran
New Zealand
Equatorial Guinea
Madagascar
Egypt
Lebanon
Dominican Republic
Brazil
Mauritius
Algeria
Togo
Nigeria
Morocco
Libya
Eritrea
Saudi Arabia
Switzerland
Turkmenistan
Gabon
Malaysia
Cyprus
Turkey
Singapore
Indonesia
Kazakhstan
Trinidad & Tobago
Guyana
Oman
Belize
Syria
Germany
São Tomé & Príncipe
Ecuador
Spain
Peru
Comoros
Palestinian Territories
Jordan
Côte d'Ivoire
Yemen
Myanmar (Burma)
Belgium
Iraq
South Sudan
Poland
Costa Rica
Tunisia
Bhutan
Kyrgyzstan
Honduras
Panama
Azerbaijan
Portugal
India
Mongolia
Jamaica
El Salvador
Nepal
Papua New Guinea
Belarus
Niger
Croatia
China
Ireland
Suriname
United Kingdom
St. Lucia
Austria
Philippines
Bolivia
North Korea
Australia
Mexico
Israel
Guatemala
Vietnam
Nicaragua
Greece
Sweden
Czechia
Netherlands
Russia
Uzbekistan
Tajikistan
Uruguay
Cuba
Denmark
Finland
Romania
Argentina
Slovakia
Brunei
Pakistan
Mali
France
Canada
Serbia
Italy
Norway
Japan
Ukraine
Albania
Solomon Islands
Kuwait
Macedonia
Armenia
Georgia
Estonia
Latvia
Paraguay
Slovenia
Lithuania
Barbados
Hungary
United States
Fiji
Venezuela
Bahamas
Bulgaria
Montenegro
Bosnia & Herzegovina
Moldova
Samoa
Vanuatu
Iceland
0
30
60
90
Percentage change in cases averted per 1000 vaccinated girls

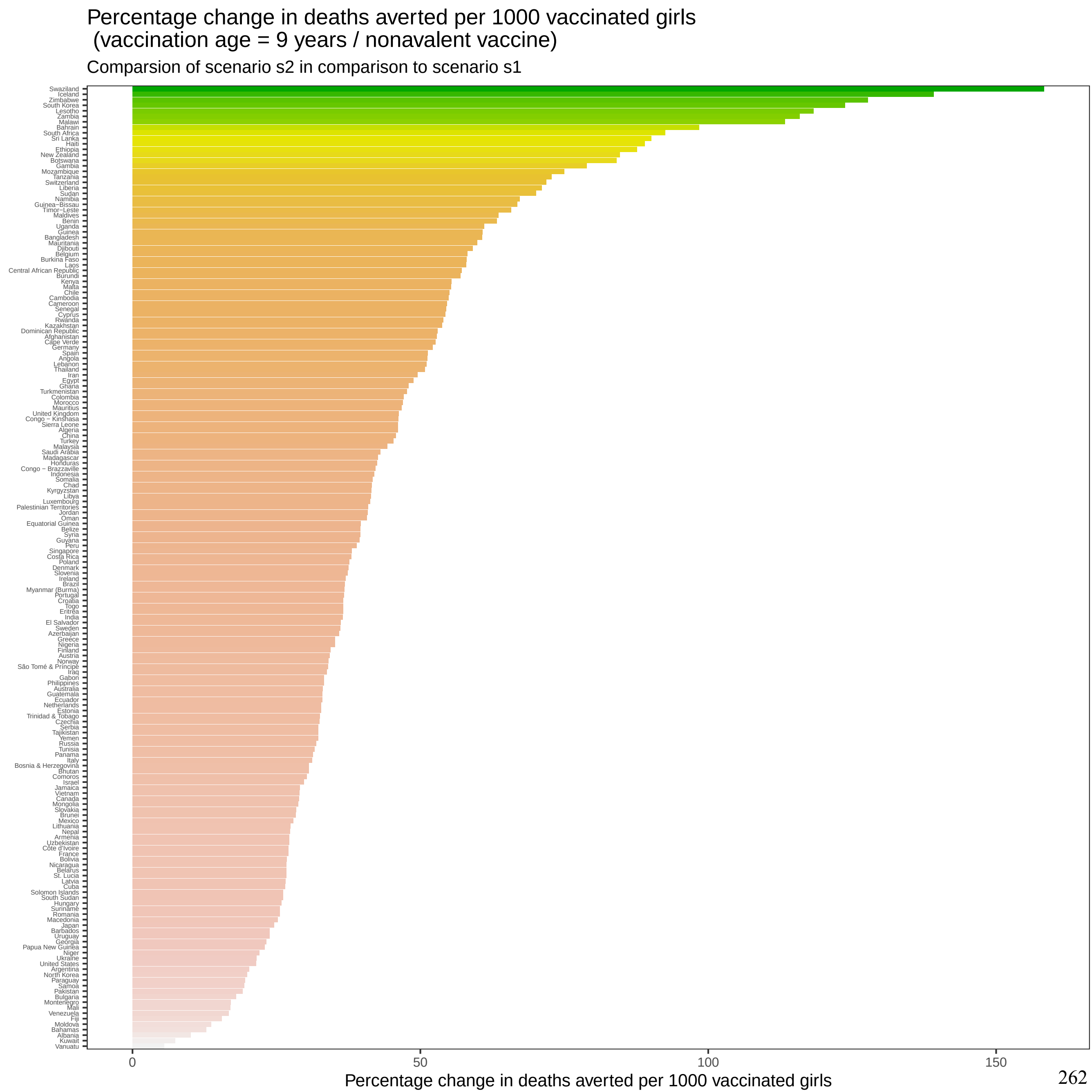

Percentage change in deaths averted per 1000 vaccinated girls
(vaccination age = 9 years / nonavalent vaccine)
Comparsion of scenario s2 in comparison to scenario s1
Percentage change in deaths averted per 1000 vaccinated girls

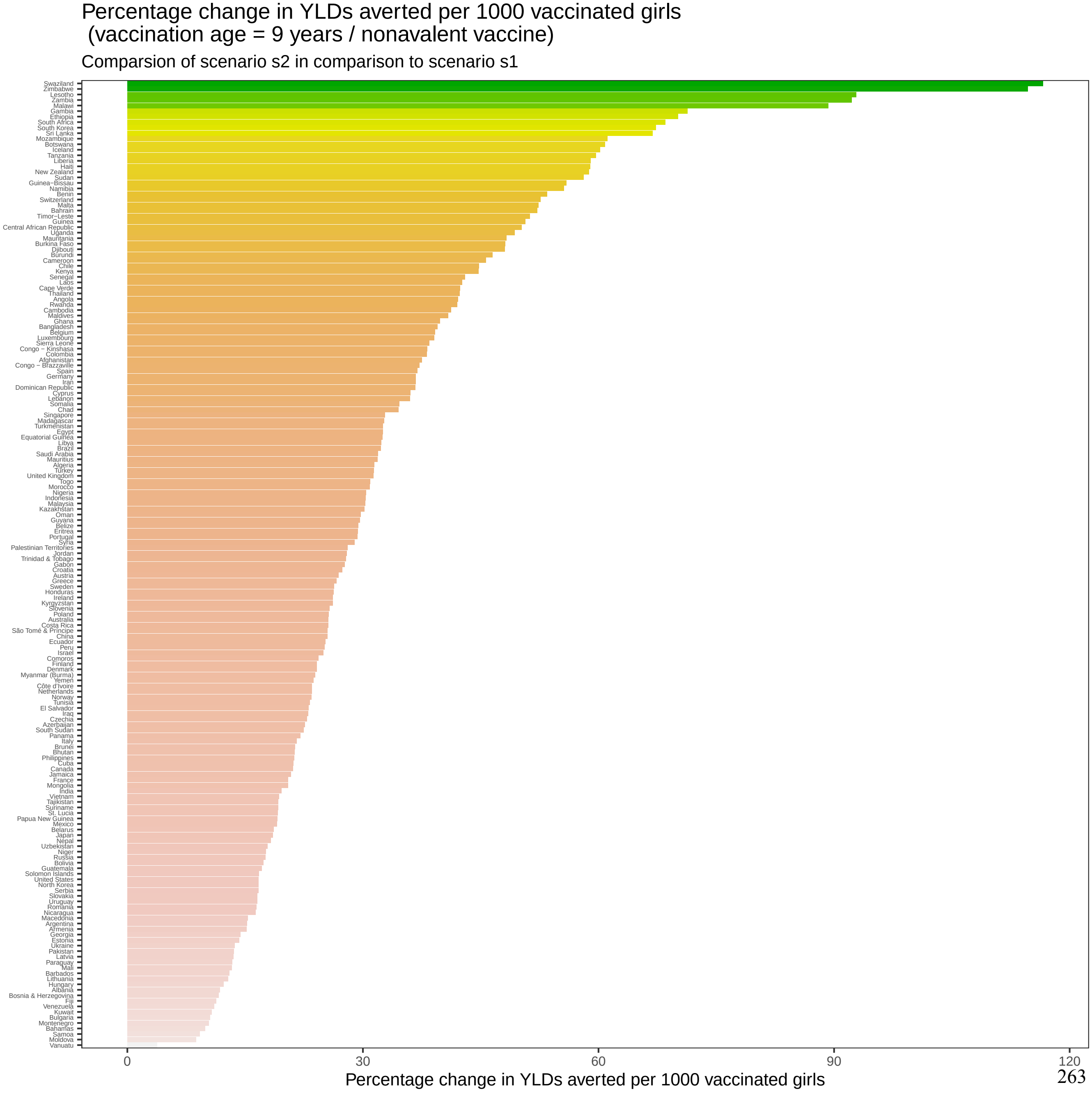
Percentage change in YLDs averted per 1000 vaccinated girls
(vaccination age = 9 years / nonavalent vaccine)
Comparsion of scenario s2 in comparison to scenario s1
Swaziland
Zimbabwe
Lesotho
Zambia
Malawi
Gambia
Ethiopia
South Africa
South Korea
Sri Lanka
Mozambique
Botswana
Iceland
Tanzania
Liberia
Haiti
New Zealand
Sudan
Guinea−Bissau
Namibia
Benin
Switzerland
Malta
Bahrain
Timor−Leste
Guinea
Central African Republic
Uganda
Mauritania
Burkina Faso
Djibouti
Burundi
Cameroon
Chile
Kenya
Senegal
Laos
Cape Verde
Thailand
Angola
Rwanda
Cambodia
Maldives
Ghana
Bangladesh
Belgium
Luxembourg
Sierra Leone
Congo − Kinshasa
Colombia
Afghanistan
Congo − Brazzaville
Spain
Germany
Iran
Dominican Republic
Cyprus
Lebanon
Somalia
Chad
Singapore
Madagascar
Turkmenistan
Egypt
Equatorial Guinea
Libya
Brazil
Saudi Arabia
Mauritius
Algeria
Turkey
United Kingdom
Togo
Morocco
Nigeria
Indonesia
Malaysia
Kazakhstan
Oman
Guyana
Belize
Eritrea
Portugal
Syria
Palestinian Territories
Jordan
Trinidad & Tobago
Gabon
Croatia
Austria
Greece
Sweden
Honduras
Ireland
Kyrgyzstan
Slovenia
Poland
Australia
Costa Rica
São Tomé & Príncipe
China
Ecuador
Peru
Israel
Comoros
Finland
Denmark
Myanmar (Burma)
Yemen
Côte d'Ivoire
Netherlands
Norway
Tunisia
El Salvador
Iraq
Czechia
Azerbaijan
South Sudan
Panama
Italy
Brunei
Bhutan
Philippines
Cuba
Canada
Jamaica
France
Mongolia
India
Vietnam
Tajikistan
Suriname
St. Lucia
Papua New Guinea
Mexico
Belarus
Japan
Nepal
Uzbekistan
Niger
Russia
Bolivia
Guatemala
Solomon Islands
United States
North Korea
Serbia
Slovakia
Uruguay
Romania
Nicaragua
Macedonia
Argentina
Armenia
Georgia
Estonia
Ukraine
Pakistan
Latvia
Paraguay
Mali
Barbados
Lithuania
Hungary
Albania
Bosnia & Herzegovina
Fiji
Venezuela
Kuwait
Bulgaria
Montenegro
Bahamas
Samoa
Moldova
Vanuatu
0
30
60
90
120
Percentage change in YLDs averted per 1000 vaccinated girls

Percentage change in YLLs averted per 1000 vaccinated girls
(vaccination age = 9 years / nonavalent vaccine)

Comparsion of scenario s2 in comparison to scenario s1

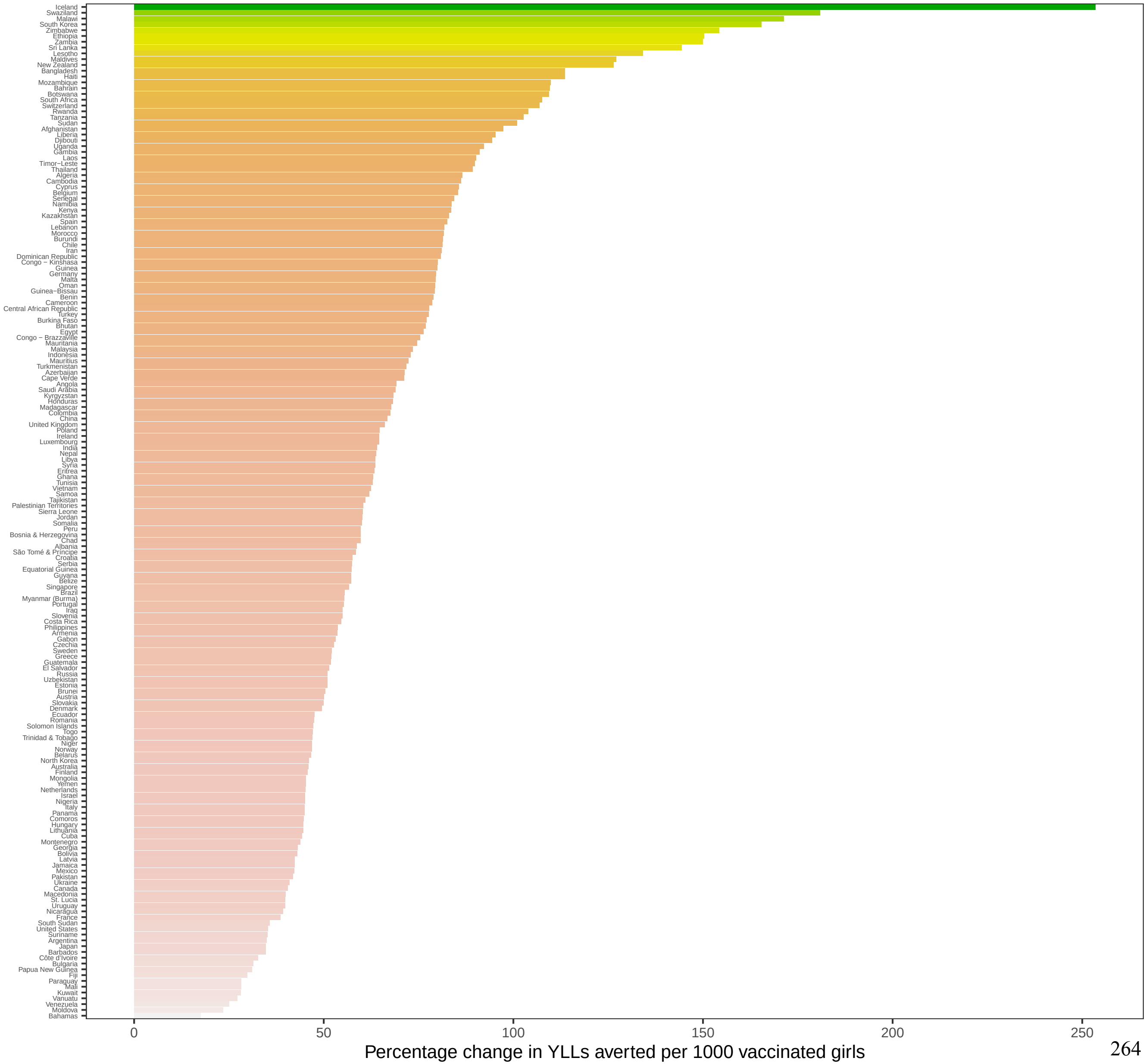

Percentage change in DALYs averted per 1000 vaccinated girls
(vaccination age = 9 years / nonavalent vaccine)

Comparsion of scenario s2 in comparison to scenario s1

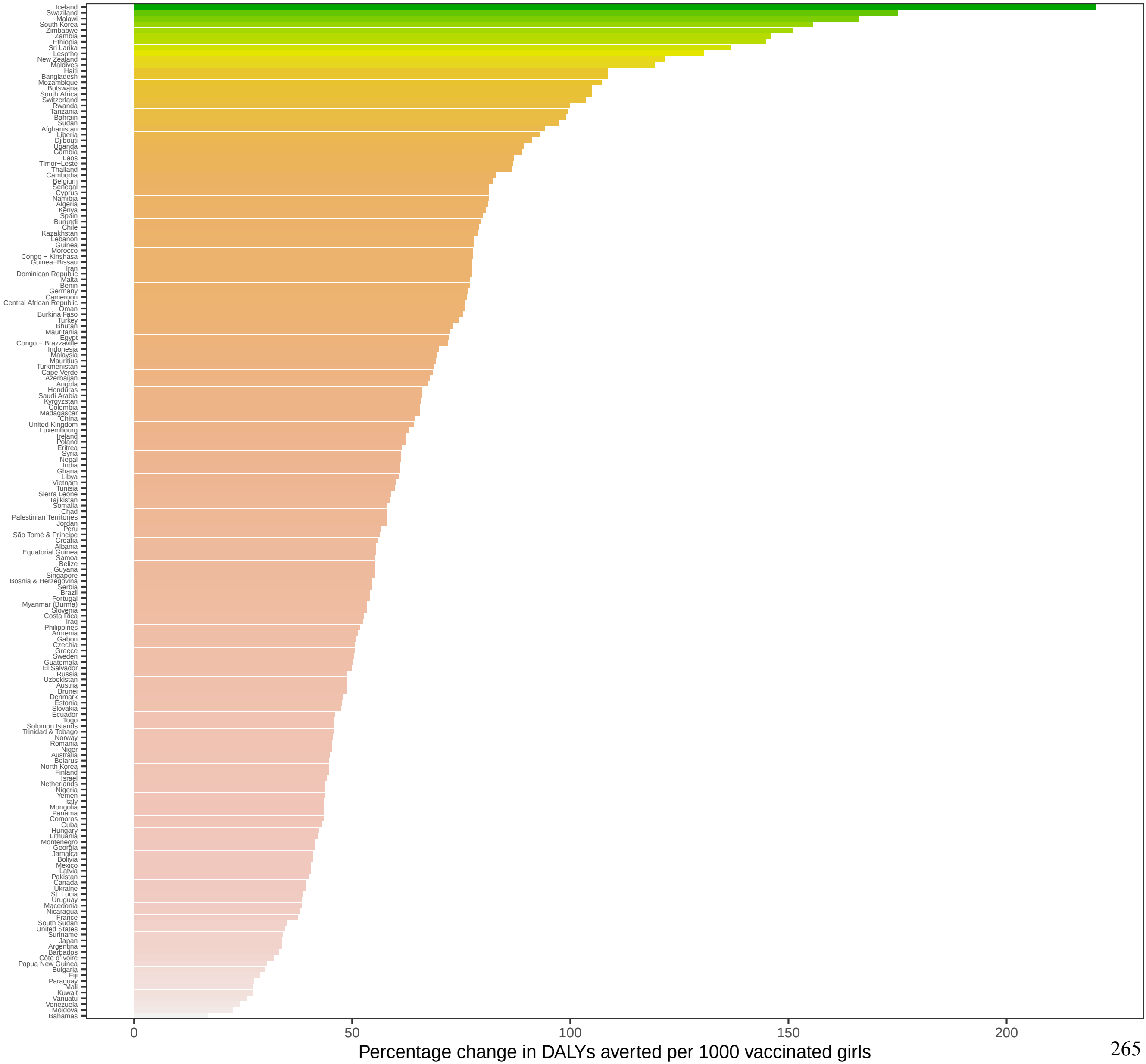

Percentage change in cases averted per 1000 vaccinated girls
(vaccination age = 9 years / nonavalent vaccine)

Comparsion of scenario s3 in comparison to scenario s1

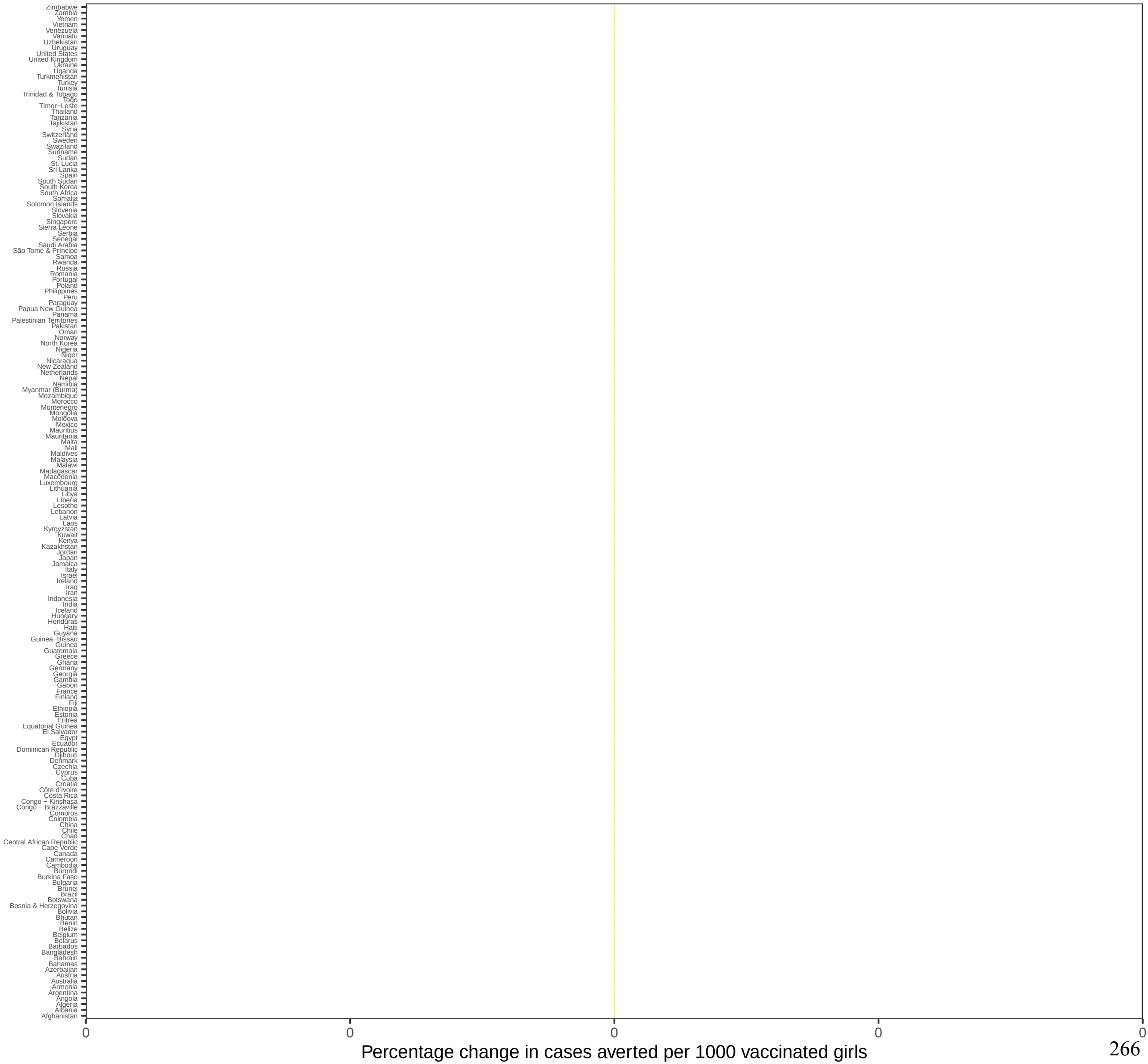

Percentage change in deaths averted per 1000 vaccinated girls
(vaccination age = 9 years / nonavalent vaccine)

Comparsion of scenario s3 in comparison to scenario s1

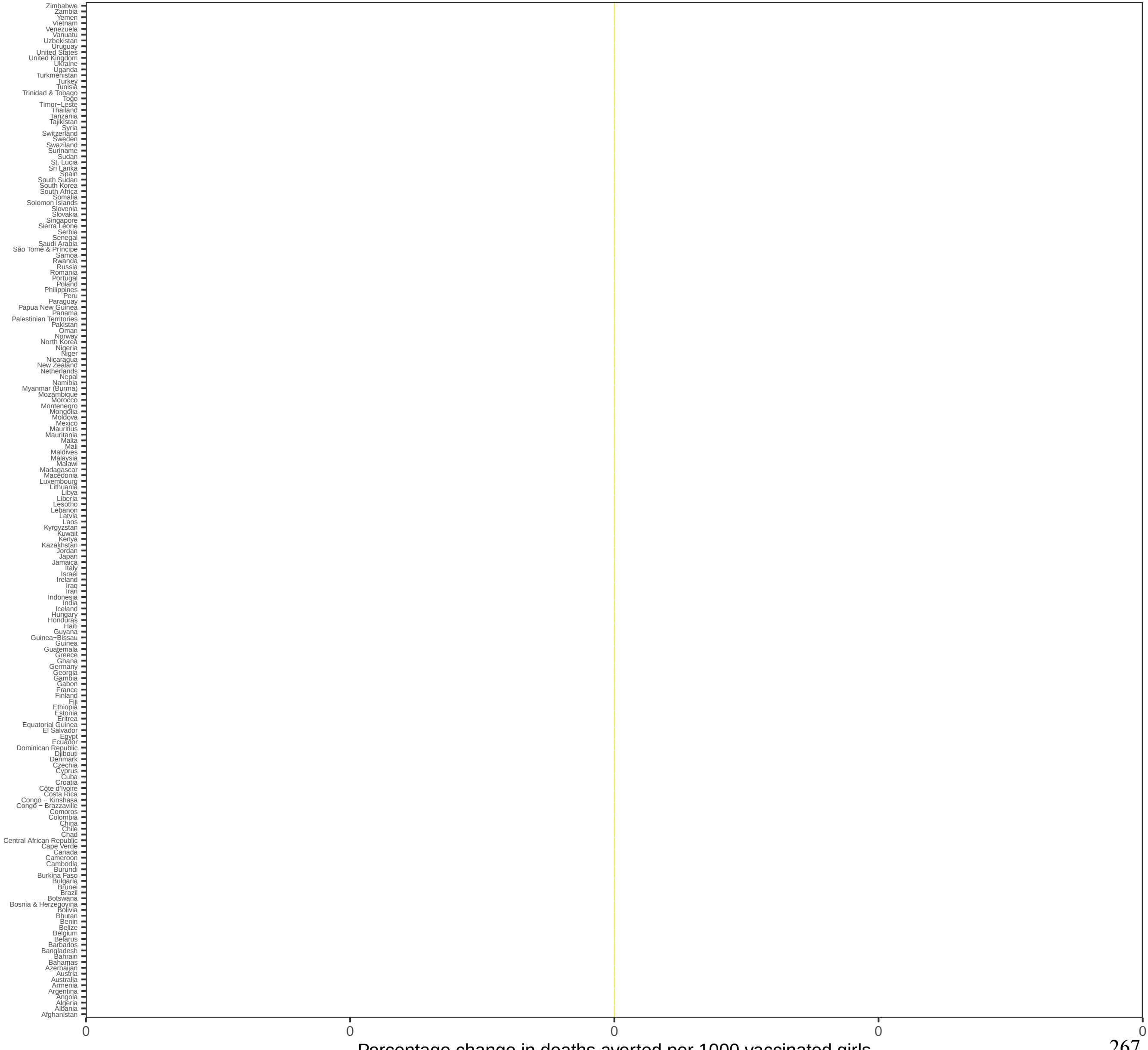

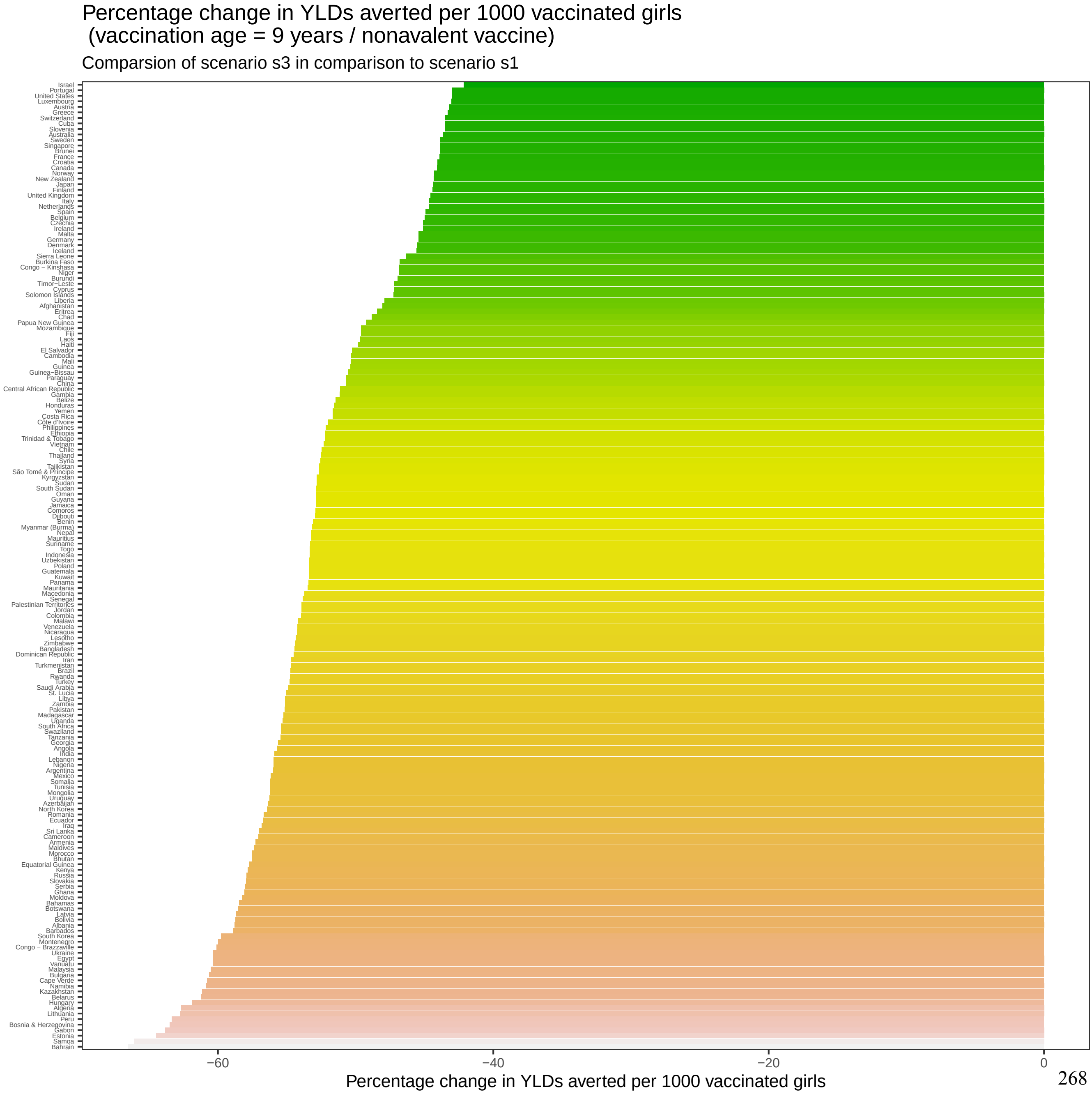

Percentage change in YLDs averted per 1000 vaccinated girls
(vaccination age = 9 years / nonavalent vaccine)
Comparsion of scenario s3 in comparison to scenario s1
Israel
Portugal
United States
Luxembourg
Austria
Greece
Switzerland
Cuba
Slovenia
Australia
Sweden
Singapore
Brunei
France
Croatia
Canada
Norway
New Zealand
Japan
Finland
United Kingdom
Italy
Netherlands
Spain
Belgium
Czechia
Ireland
Malta
Germany
Denmark
Iceland
Sierra Leone
Burkina Faso
Congo − Kinshasa
Niger
Burundi
Timor−Leste
Cyprus
Solomon Islands
Liberia
Afghanistan
Eritrea
Chad
Papua New Guinea
Mozambique
Fiji
Laos
Haiti
El Salvador
Cambodia
Mali
Guinea
Guinea−Bissau
Paraguay
China
Central African Republic
Gambia
Belize
Honduras
Yemen
Costa Rica
Côte d'Ivoire
Philippines
Ethiopia
Trinidad & Tobago
Vietnam
Chile
Thailand
Syria
Tajikistan
São Tomé & Príncipe
Kyrgyzstan
Sudan
South Sudan
Oman
Guyana
Jamaica
Comoros
Djibouti
Benin
Myanmar (Burma)
Nepal
Mauritius
Suriname
Togo
Indonesia
Uzbekistan
Poland
Guatemala
Kuwait
Panama
Mauritania
Macedonia
Senegal
Palestinian Territories
Jordan
Colombia
Malawi
Venezuela
Nicaragua
Lesotho
Zimbabwe
Bangladesh
Dominican Republic
Iran
Turkmenistan
Brazil
Rwanda
Turkey
Saudi Arabia
St. Lucia
Libya
Zambia
Pakistan
Madagascar
Uganda
South Africa
Swaziland
Tanzania
Georgia
Angola
India
Lebanon
Nigeria
Argentina
Mexico
Somalia
Tunisia
Mongolia
Uruguay
Azerbaijan
North Korea
Romania
Ecuador
Iraq
Sri Lanka
Cameroon
Armenia
Maldives
Morocco
Bhutan
Equatorial Guinea
Kenya
Russia
Slovakia
Serbia
Ghana
Moldova
Bahamas
Botswana
Latvia
Bolivia
Albania
Barbados
South Korea
Montenegro
Congo − Brazzaville
Ukraine
Egypt
Vanuatu
Malaysia
Bulgaria
Cape Verde
Namibia
Kazakhstan
Belarus
Hungary
Algeria
Lithuania
Peru
Bosnia & Herzegovina
Gabon
Estonia
Samoa
Bahrain
−60
−40
−20
0
Percentage change in YLDs averted per 1000 vaccinated girls

Percentage change in YLLs averted per 1000 vaccinated girls
(vaccination age = 9 years / nonavalent vaccine)

Comparsion of scenario s3 in comparison to scenario s1

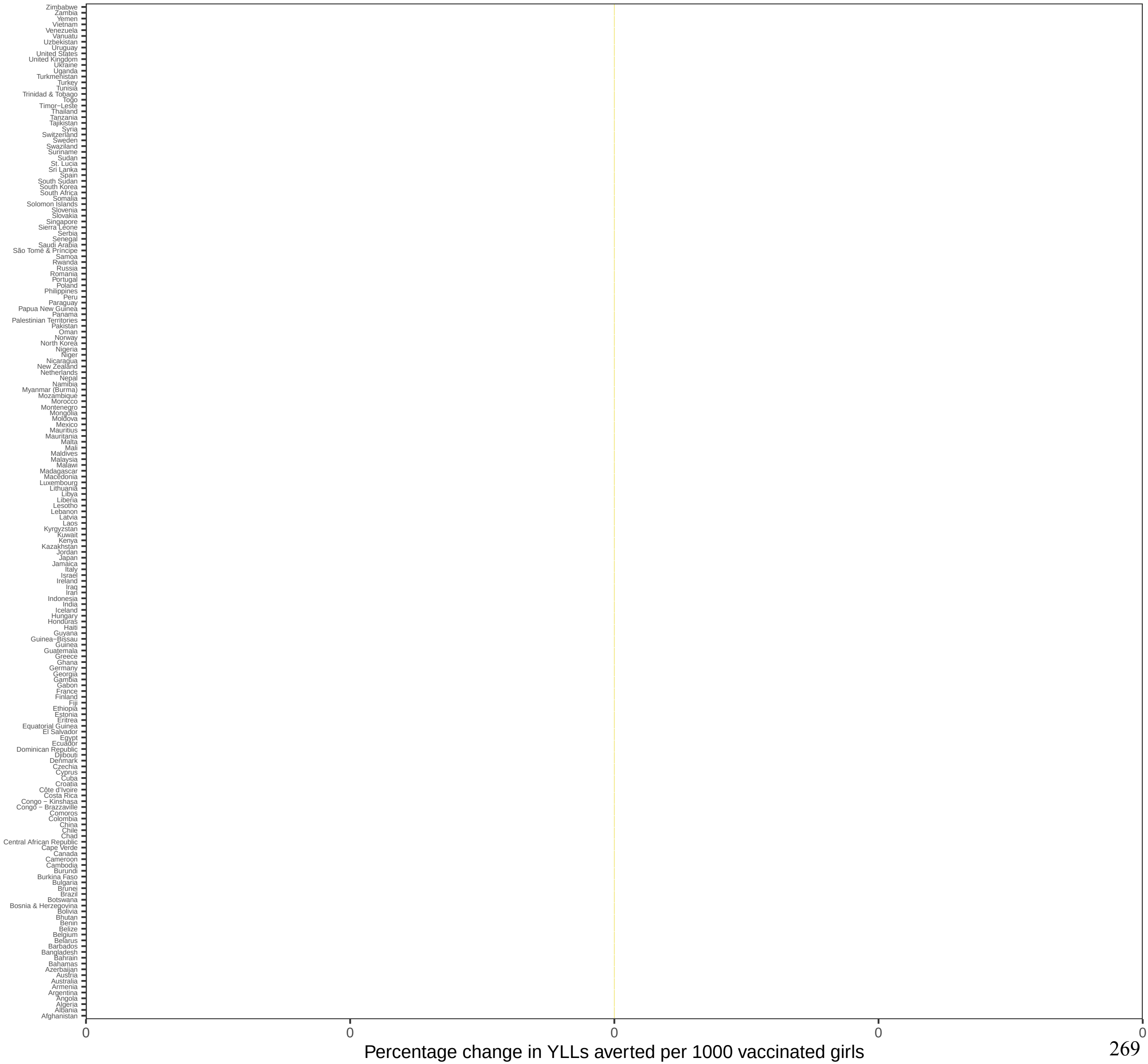

Percentage change in DALYs averted per 1000 vaccinated girls
(vaccination age = 9 years / nonavalent vaccine)

Comparsion of scenario s3 in comparison to scenario s1

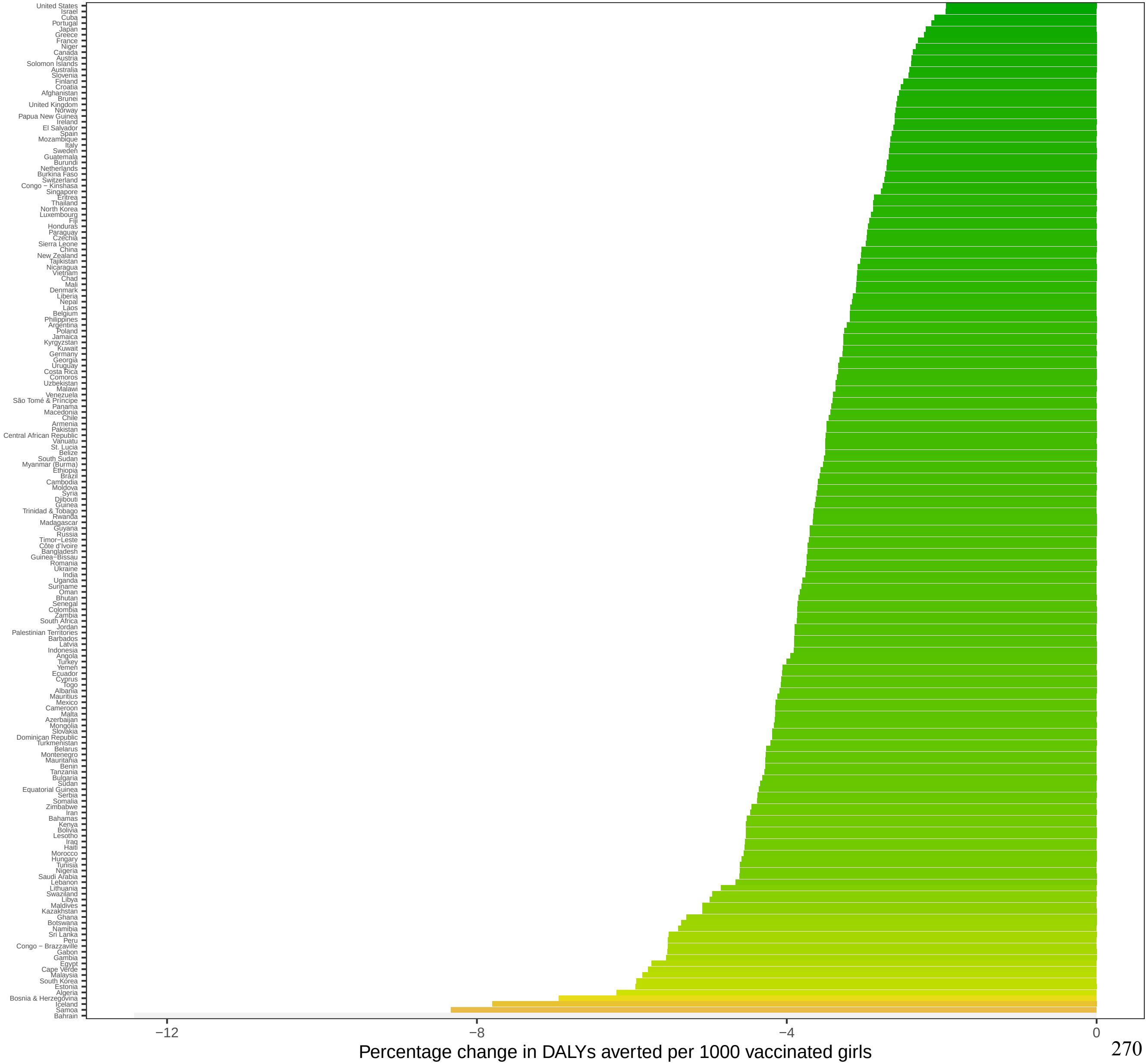

Percentage change in cases averted per 1000 vaccinated girls
(vaccination age = 9 years / nonavalent vaccine)

Comparsion of scenario s4 in comparison to scenario s1

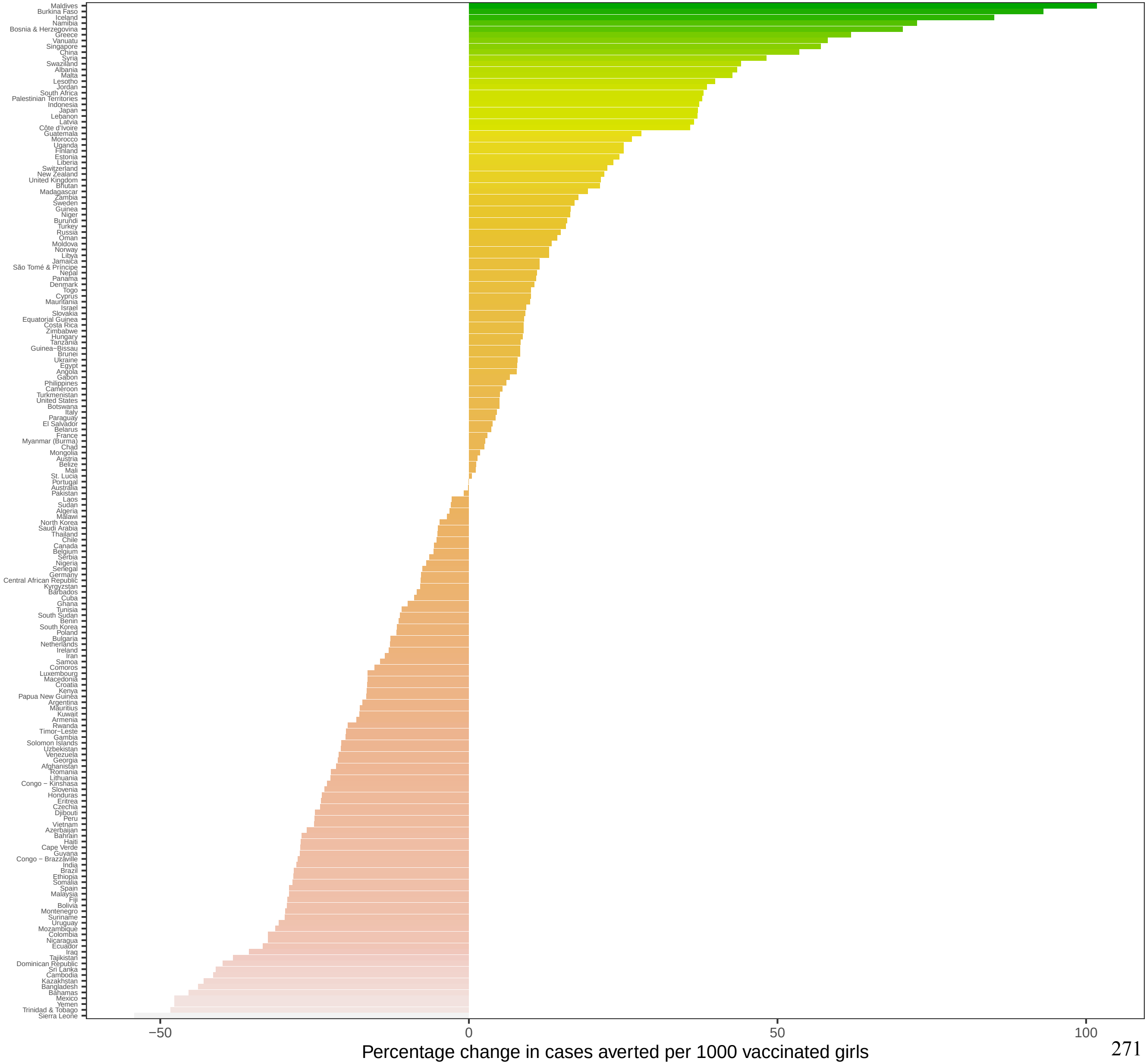

Percentage change in deaths averted per 1000 vaccinated girls
(vaccination age = 9 years / nonavalent vaccine)

Comparsion of scenario s4 in comparison to scenario s1

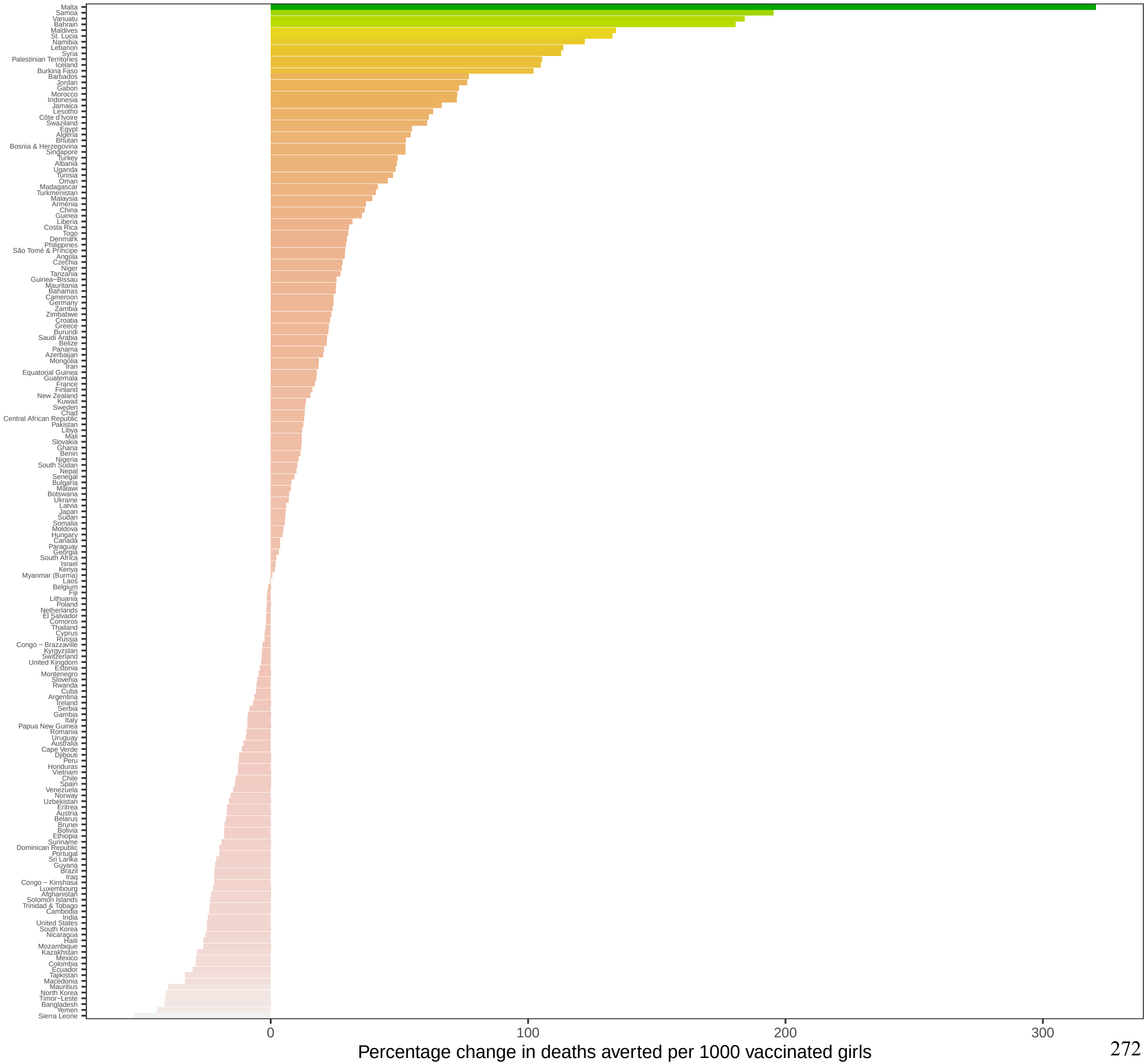

Percentage change in YLDs averted per 1000 vaccinated girls
(vaccination age = 9 years / nonavalent vaccine)

Comparsion of scenario s4 in comparison to scenario s1

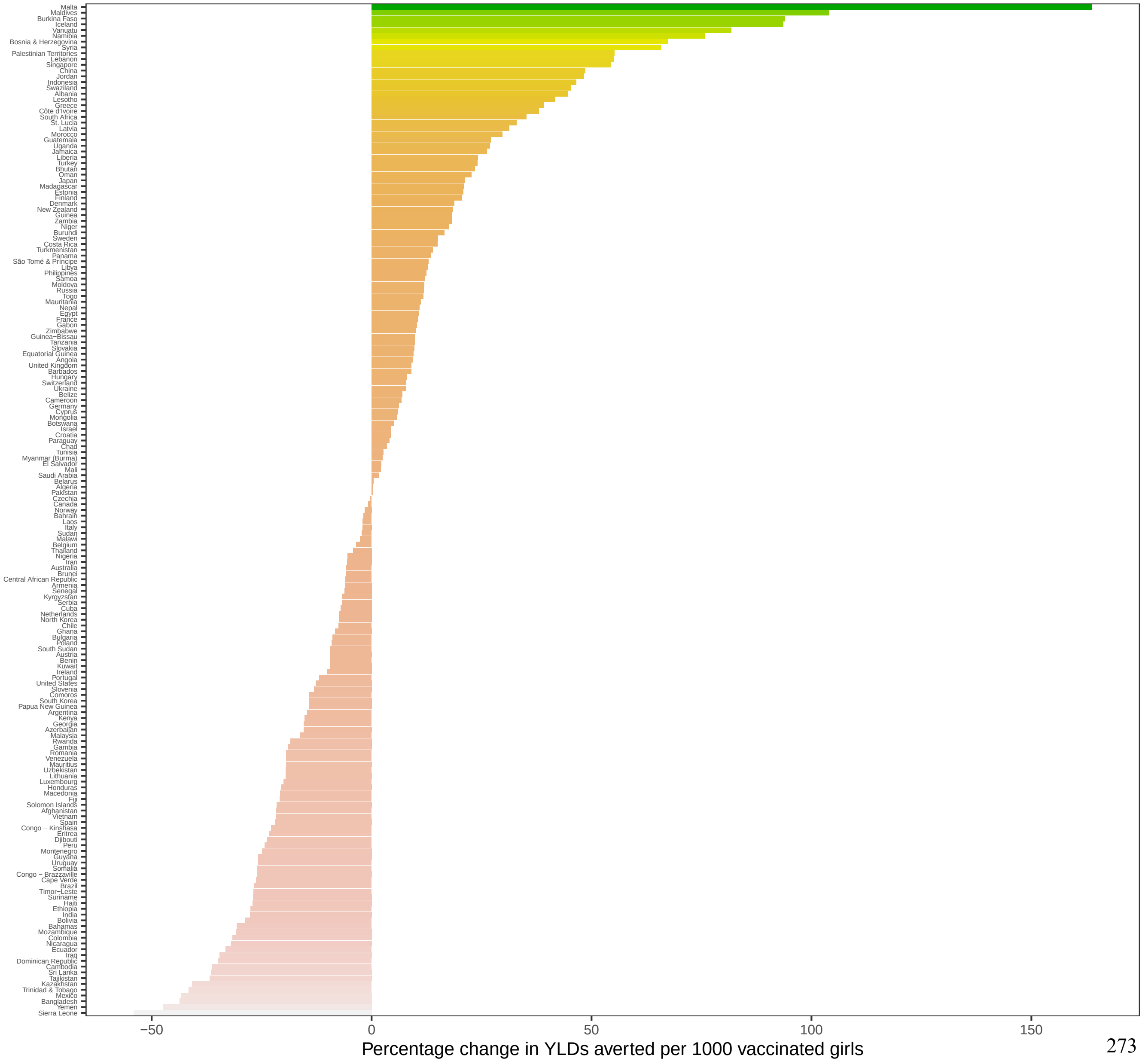

Percentage change in YLLs averted per 1000 vaccinated girls
(vaccination age = 9 years / nonavalent vaccine)

Comparsion of scenario s4 in comparison to scenario s1

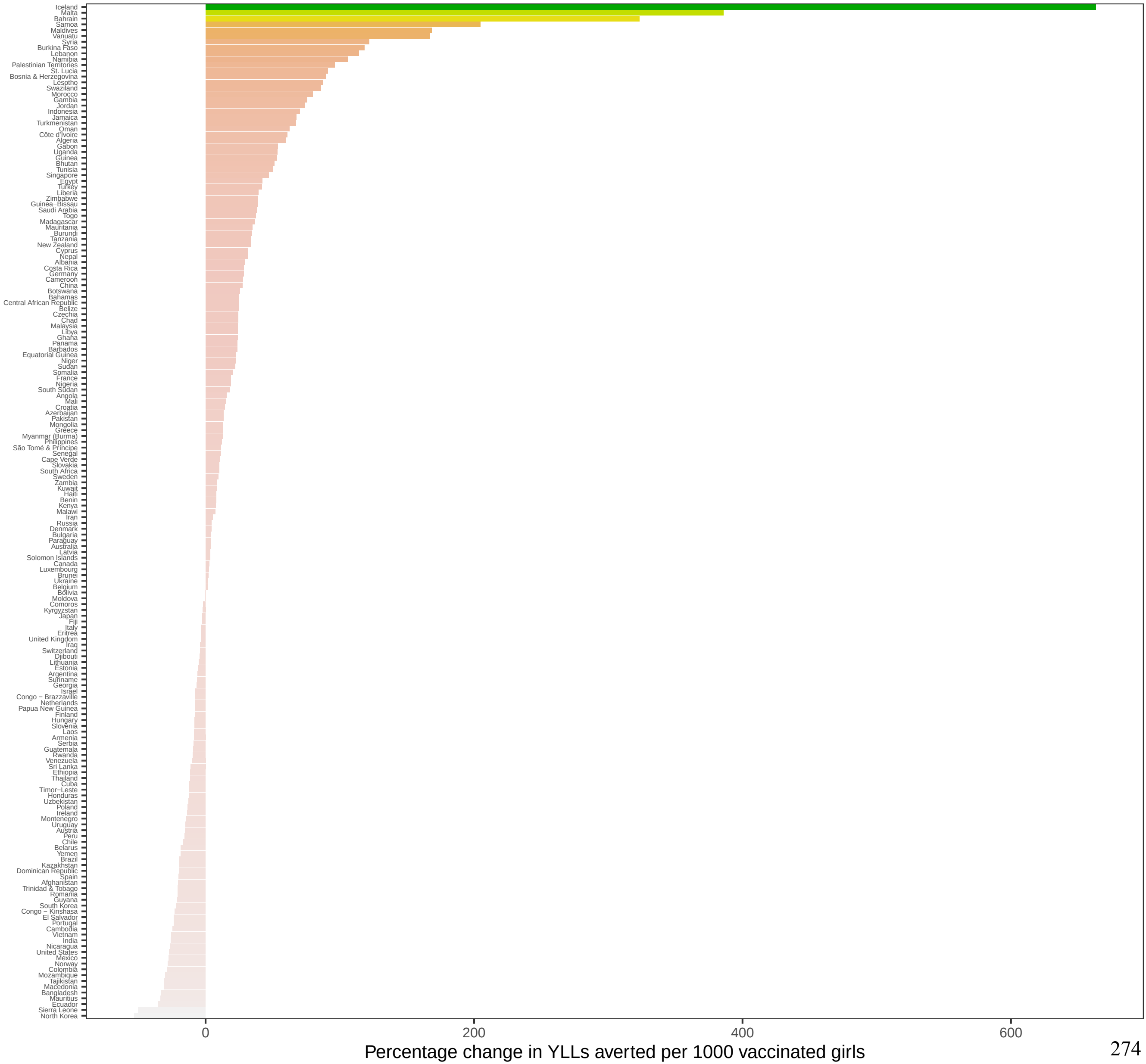

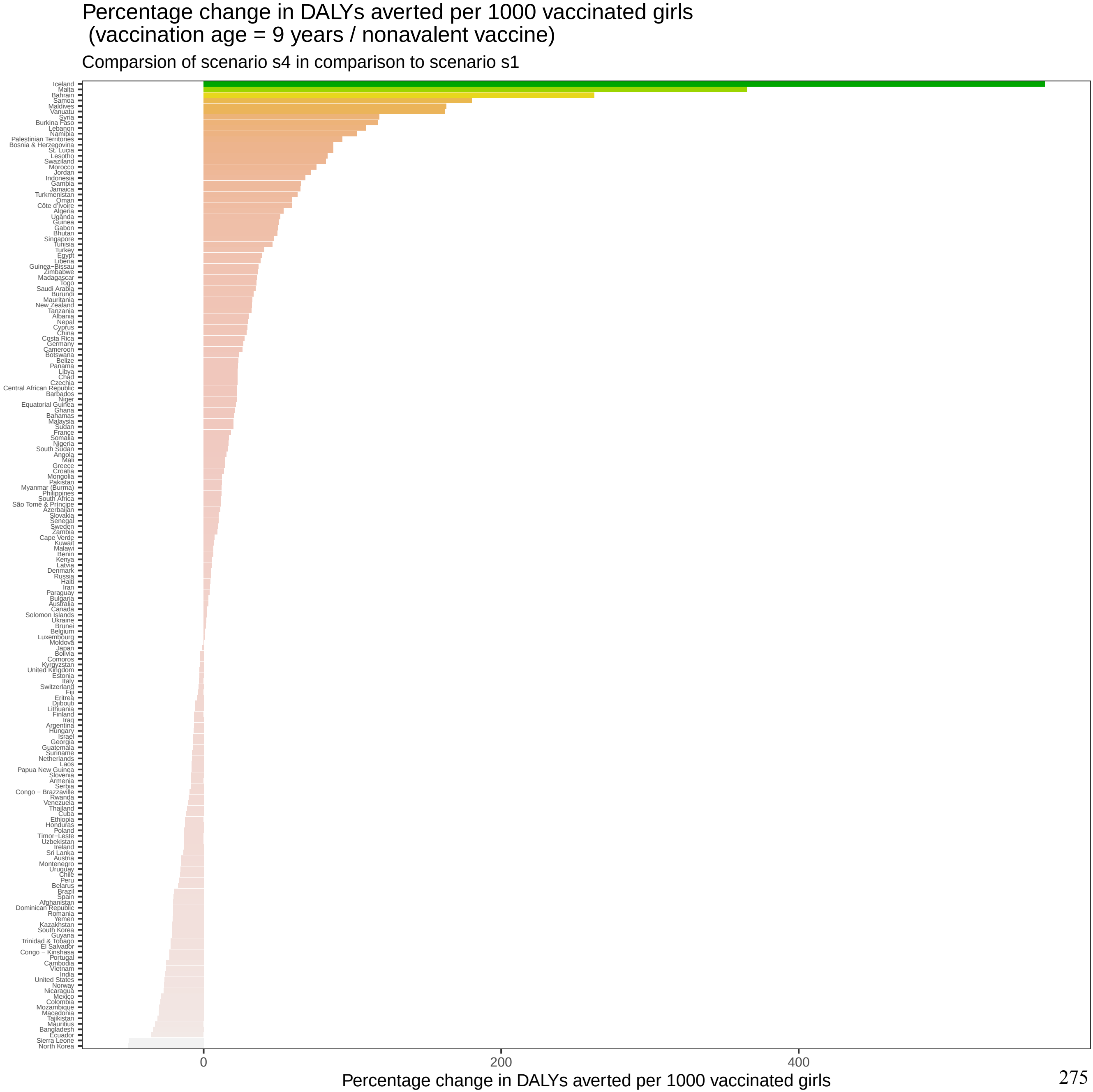

Percentage change in DALYs averted per 1000 vaccinated girls
(vaccination age = 9 years / nonavalent vaccine)
Comparsion of scenario s4 in comparison to scenario s1
Iceland
Malta
Bahrain
Samoa
Maldives
Vanuatu
Syria
Burkina Faso
Lebanon
Namibia
Palestinian Territories
Bosnia & Herzegovina
St. Lucia
Lesotho
Swaziland
Morocco
Jordan
Indonesia
Gambia
Jamaica
Turkmenistan
Oman
Côte d'Ivoire
Algeria
Uganda
Guinea
Gabon
Bhutan
Singapore
Tunisia
Turkey
Egypt
Liberia
Guinea−Bissau
Zimbabwe
Madagascar
Togo
Saudi Arabia
Burundi
Mauritania
New Zealand
Tanzania
Albania
Nepal
Cyprus
China
Costa Rica
Germany
Cameroon
Botswana
Belize
Panama
Libya
Chad
Czechia
Central African Republic
Barbados
Niger
Equatorial Guinea
Ghana
Bahamas
Malaysia
Sudan
France
Somalia
Nigeria
South Sudan
Angola
Mali
Greece
Croatia
Mongolia
Pakistan
Myanmar (Burma)
Philippines
South Africa
São Tomé & Príncipe
Azerbaijan
Slovakia
Senegal
Sweden
Zambia
Cape Verde
Kuwait
Malawi
Benin
Kenya
Latvia
Denmark
Russia
Haiti
Iran
Paraguay
Bulgaria
Australia
Canada
Solomon Islands
Ukraine
Brunei
Belgium
Luxembourg
Moldova
Japan
Bolivia
Comoros
Kyrgyzstan
United Kingdom
Estonia
Italy
Switzerland
Fiji
Eritrea
Djibouti
Lithuania
Finland
Iraq
Argentina
Hungary
Israel
Georgia
Guatemala
Suriname
Netherlands
Laos
Papua New Guinea
Slovenia
Armenia
Serbia
Congo − Brazzaville
Rwanda
Venezuela
Thailand
Cuba
Ethiopia
Honduras
Poland
Timor−Leste
Uzbekistan
Ireland
Sri Lanka
Austria
Montenegro
Uruguay
Chile
Peru
Belarus
Brazil
Spain
Afghanistan
Dominican Republic
Romania
Yemen
Kazakhstan
South Korea
Guyana
Trinidad & Tobago
El Salvador
Congo − Kinshasa
Portugal
Cambodia
Vietnam
India
United States
Norway
Nicaragua
Mexico
Colombia
Mozambique
Macedonia
Tajikistan
Mauritius
Bangladesh
Ecuador
Sierra Leone
North Korea
0
200
400
Percentage change in DALYs averted per 1000 vaccinated girls

Percentage change in cases averted per 1000 vaccinated girls
(vaccination age = 9 years / nonavalent vaccine)

Comparsion of scenario s5 in comparison to scenario s1

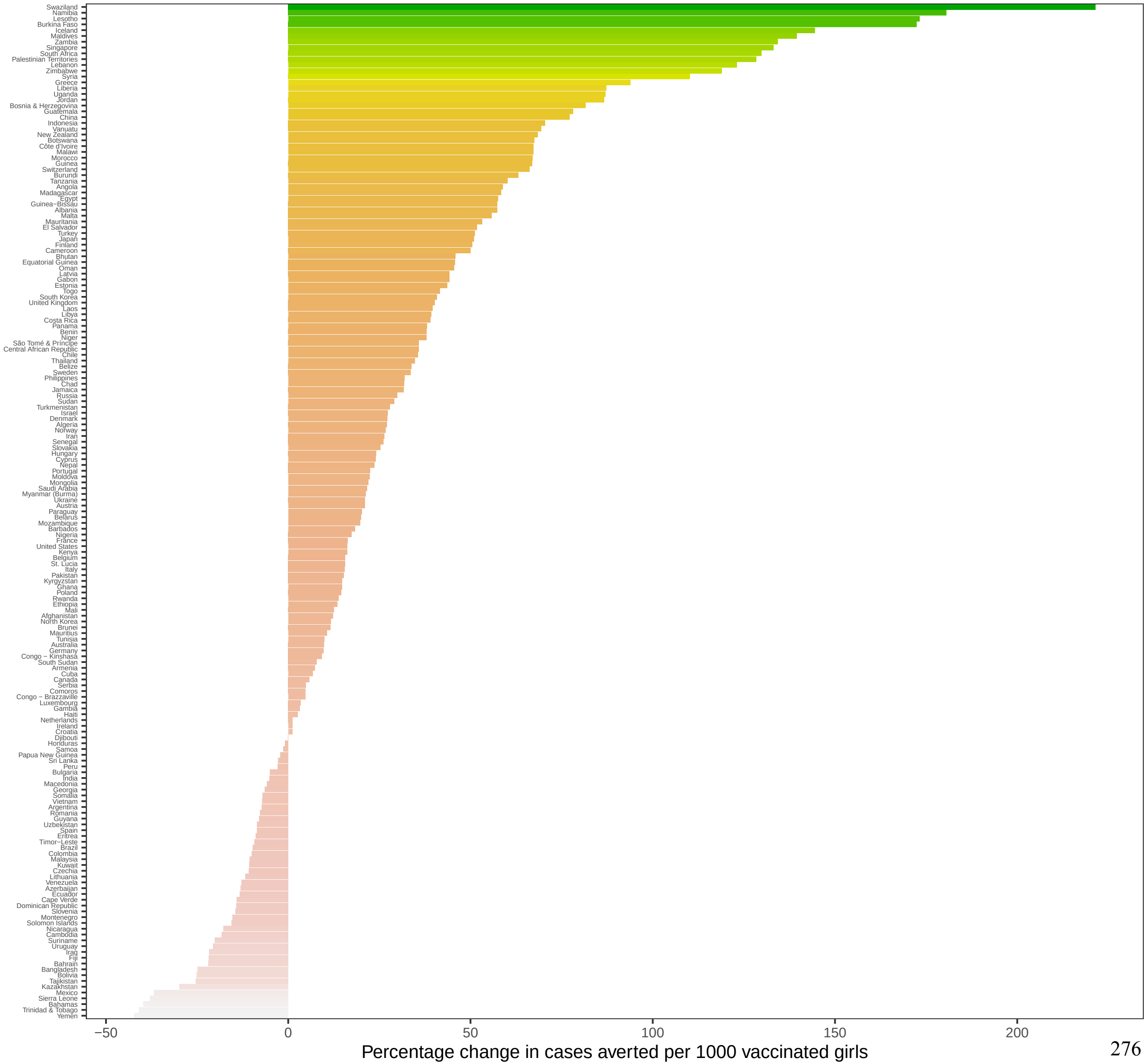

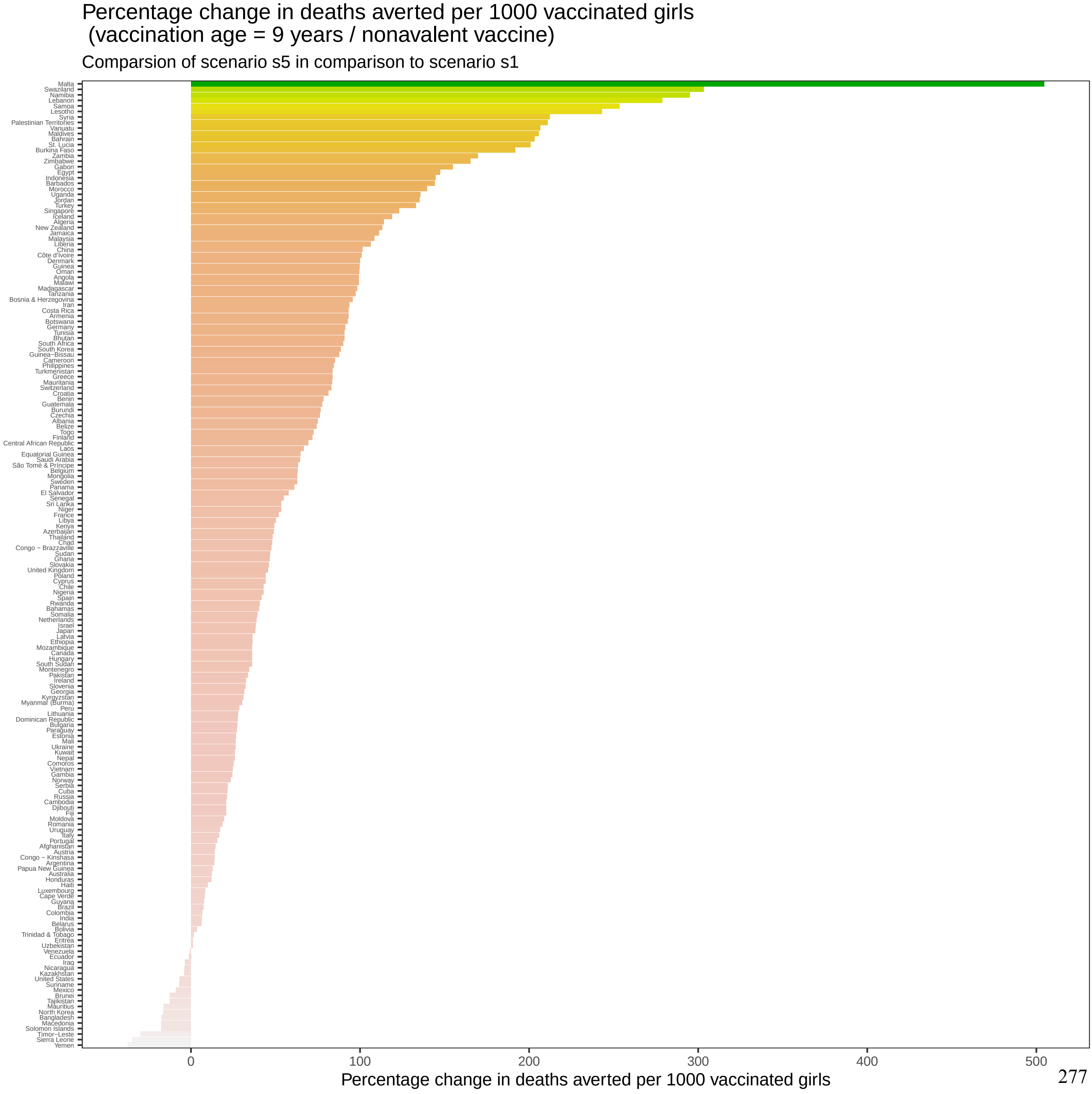
Percentage change in deaths averted per 1000 vaccinated girls
(vaccination age = 9 years / nonavalent vaccine)
Comparsion of scenario s5 in comparison to scenario s1
Percentage change in deaths averted per 1000 vaccinated girls
0
100
200
300
400
500

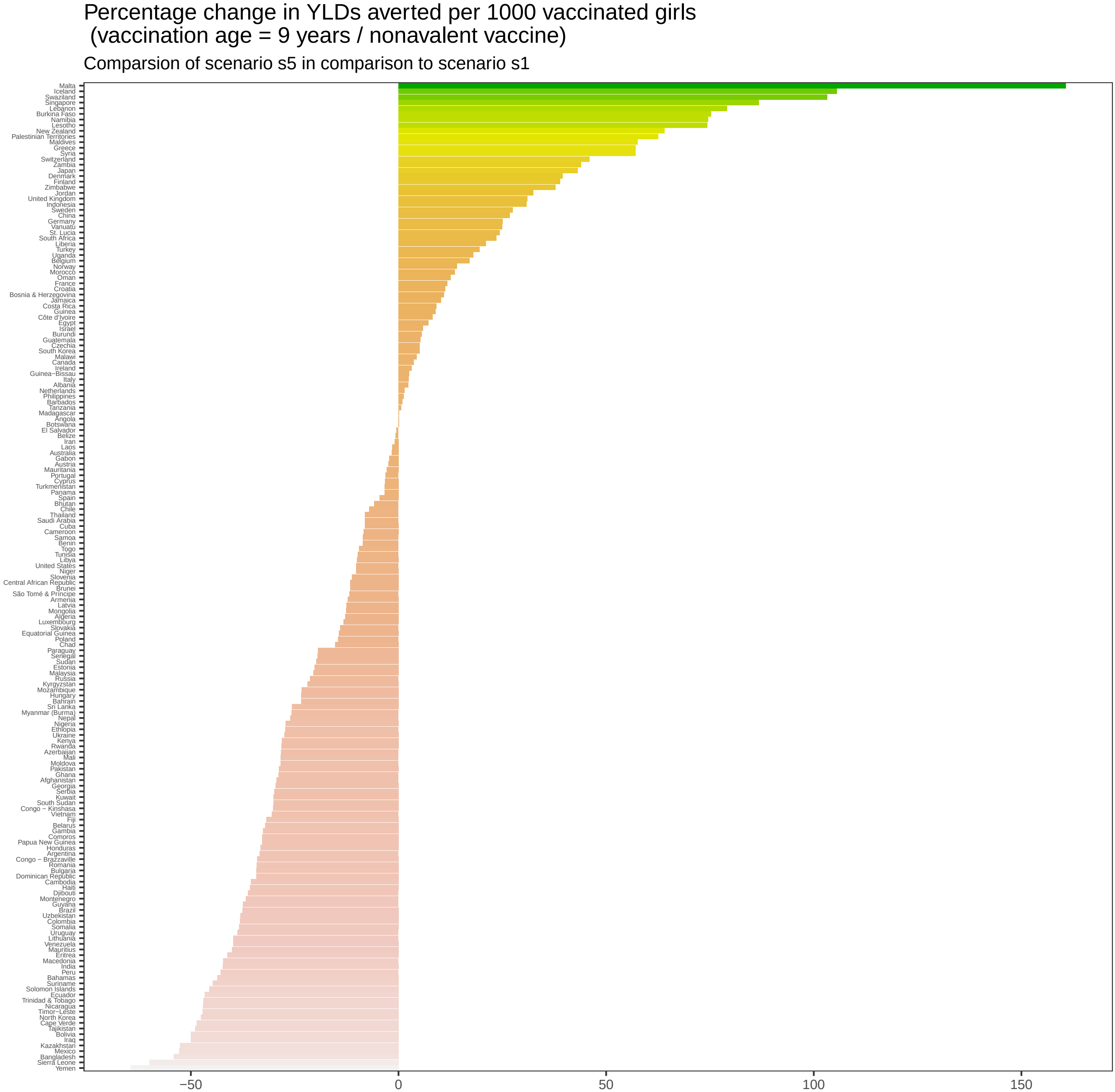
Percentage change in YLDs averted per 1000 vaccinated girls
(vaccination age = 9 years / nonavalent vaccine)
Comparsion of scenario s5 in comparison to scenario s1
Malta
Iceland
Swaziland
Singapore
Lebanon
Burkina Faso
Namibia
Lesotho
New Zealand
Palestinian Territories
Maldives
Greece
Syria
Switzerland
Zambia
Japan
Denmark
Finland
Zimbabwe
Jordan
United Kingdom
Indonesia
Sweden
China
Germany
Vanuatu
St. Lucia
South Africa
Liberia
Turkey
Uganda
Belgium
Norway
Morocco
Oman
France
Croatia
Bosnia & Herzegovina
Jamaica
Costa Rica
Guinea
Côte d'Ivoire
Egypt
Israel
Burundi
Guatemala
Czechia
South Korea
Malawi
Canada
Ireland
Guinea−Bissau
Italy
Albania
Netherlands
Philippines
Barbados
Tanzania
Madagascar
Angola
Botswana
El Salvador
Belize
Iran
Laos
Australia
Gabon
Austria
Mauritania
Portugal
Cyprus
Turkmenistan
Panama
Spain
Bhutan
Chile
Thailand
Saudi Arabia
Cuba
Cameroon
Samoa
Benin
Togo
Tunisia
Libya
United States
Niger
Slovenia
Central African Republic
Brunei
São Tomé & Príncipe
Armenia
Latvia
Mongolia
Algeria
Luxembourg
Slovakia
Equatorial Guinea
Poland
Chad
Paraguay
Senegal
Sudan
Estonia
Malaysia
Russia
Kyrgyzstan
Mozambique
Hungary
Bahrain
Sri Lanka
Myanmar (Burma)
Nepal
Nigeria
Ethiopia
Ukraine
Kenya
Rwanda
Azerbaijan
Mali
Moldova
Pakistan
Ghana
Afghanistan
Georgia
Serbia
Kuwait
South Sudan
Congo − Kinshasa
Vietnam
Fiji
Belarus
Gambia
Comoros
Papua New Guinea
Honduras
Argentina
Congo − Brazzaville
Romania
Bulgaria
Dominican Republic
Cambodia
Haiti
Djibouti
Montenegro
Guyana
Brazil
Uzbekistan
Colombia
Somalia
Uruguay
Lithuania
Venezuela
Mauritius
Eritrea
Macedonia
India
Peru
Bahamas
Suriname
Solomon Islands
Ecuador
Trinidad & Tobago
Nicaragua
Timor−Leste
North Korea
Cape Verde
Tajikistan
Bolivia
Iraq
Kazakhstan
Mexico
Bangladesh
Sierra Leone
Yemen
−50
0
50
100
150
Percentage change in YLDs averted per 1000 vaccinated girls

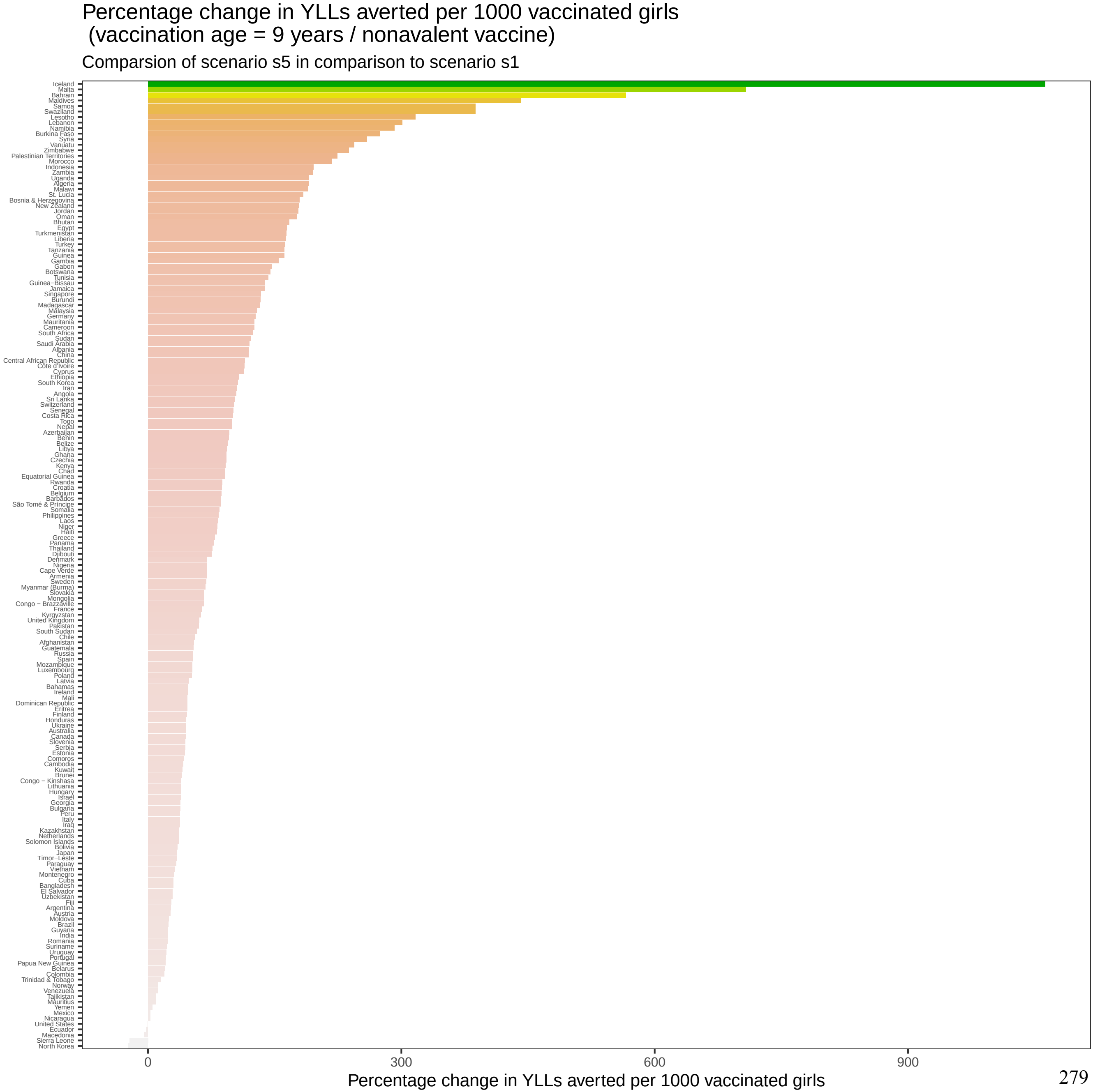
Percentage change in YLLs averted per 1000 vaccinated girls
(vaccination age = 9 years / nonavalent vaccine)
Comparsion of scenario s5 in comparison to scenario s1
Iceland
Malta
Bahrain
Maldives
Samoa
Swaziland
Lesotho
Lebanon
Namibia
Burkina Faso
Syria
Vanuatu
Zimbabwe
Palestinian Territories
Morocco
Indonesia
Zambia
Uganda
Algeria
Malawi
St. Lucia
Bosnia & Herzegovina
New Zealand
Jordan
Oman
Bhutan
Egypt
Turkmenistan
Liberia
Turkey
Tanzania
Guinea
Gambia
Gabon
Botswana
Tunisia
Guinea−Bissau
Jamaica
Singapore
Burundi
Madagascar
Malaysia
Germany
Mauritania
Cameroon
South Africa
Sudan
Saudi Arabia
Albania
China
Central African Republic
Côte d'Ivoire
Cyprus
Ethiopia
South Korea
Iran
Angola
Sri Lanka
Switzerland
Senegal
Costa Rica
Togo
Nepal
Azerbaijan
Benin
Belize
Libya
Ghana
Czechia
Kenya
Chad
Equatorial Guinea
Rwanda
Croatia
Belgium
Barbados
São Tomé & Príncipe
Somalia
Philippines
Laos
Niger
Haiti
Greece
Panama
Thailand
Djibouti
Denmark
Nigeria
Cape Verde
Armenia
Sweden
Myanmar (Burma)
Slovakia
Mongolia
Congo − Brazzaville
France
Kyrgyzstan
United Kingdom
Pakistan
South Sudan
Chile
Afghanistan
Guatemala
Russia
Spain
Mozambique
Luxembourg
Poland
Latvia
Bahamas
Ireland
Mali
Dominican Republic
Eritrea
Finland
Honduras
Ukraine
Australia
Canada
Slovenia
Serbia
Estonia
Comoros
Cambodia
Kuwait
Brunei
Congo − Kinshasa
Lithuania
Hungary
Israel
Georgia
Bulgaria
Peru
Italy
Iraq
Kazakhstan
Netherlands
Solomon Islands
Bolivia
Japan
Timor−Leste
Paraguay
Vietnam
Montenegro
Cuba
Bangladesh
El Salvador
Uzbekistan
Fiji
Argentina
Austria
Moldova
Brazil
Guyana
India
Romania
Suriname
Uruguay
Portugal
Papua New Guinea
Belarus
Colombia
Trinidad & Tobago
Norway
Venezuela
Tajikistan
Mauritius
Yemen
Mexico
Nicaragua
United States
Ecuador
Macedonia
Sierra Leone
North Korea
0
300
600
900
Percentage change in YLLs averted per 1000 vaccinated girls

Percentage change in DALYs averted per 1000 vaccinated girls (vaccination age = 9 years / nonavalent vaccine)

Comparsion of scenario s5 in comparison to scenario s1

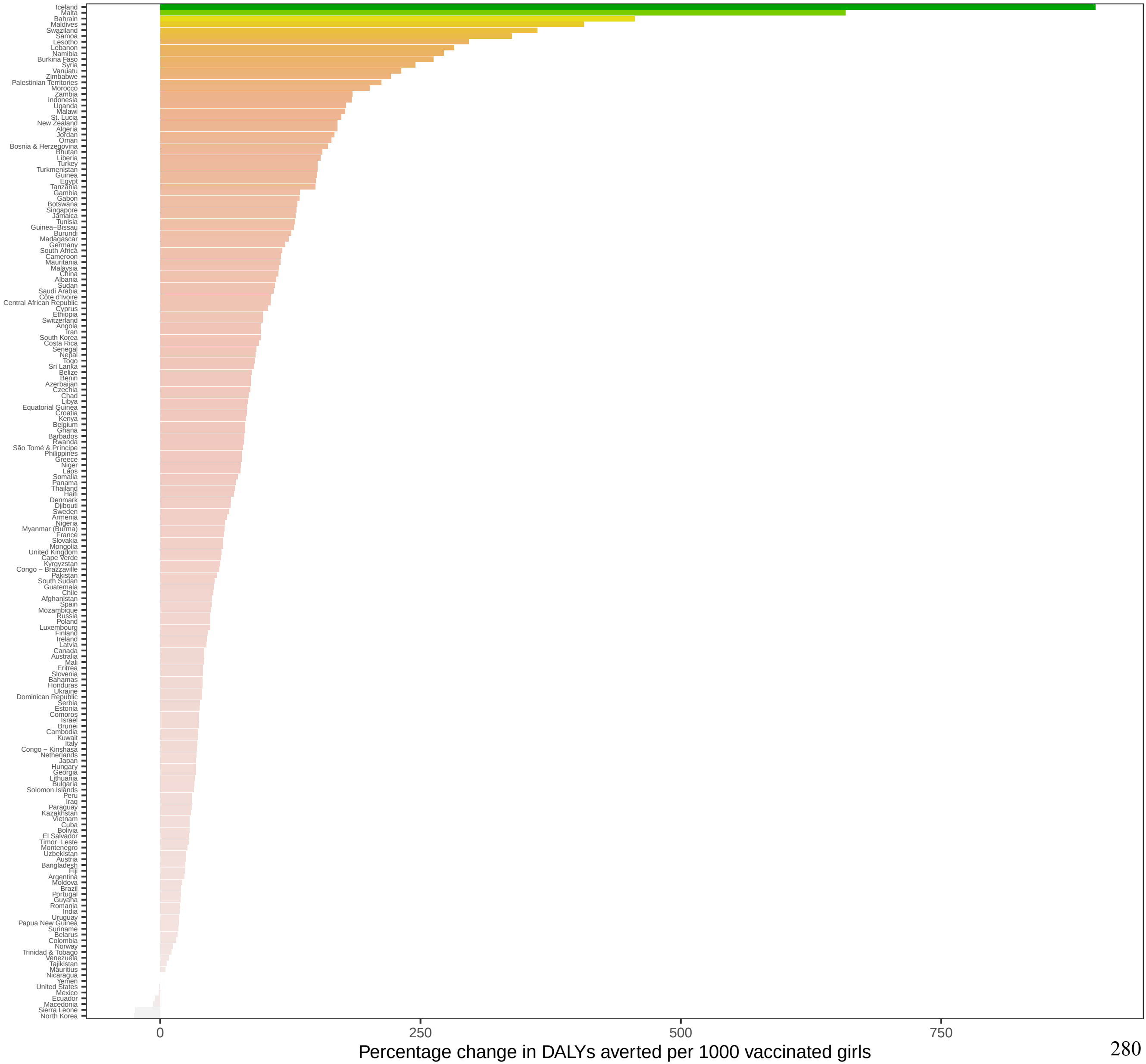

Percentage change in cases averted per 1000 vaccinated girls
(vaccination age = 12 years / bivalent/quadrivalent vaccine)

Comparsion of scenario s2 in comparison to scenario s1

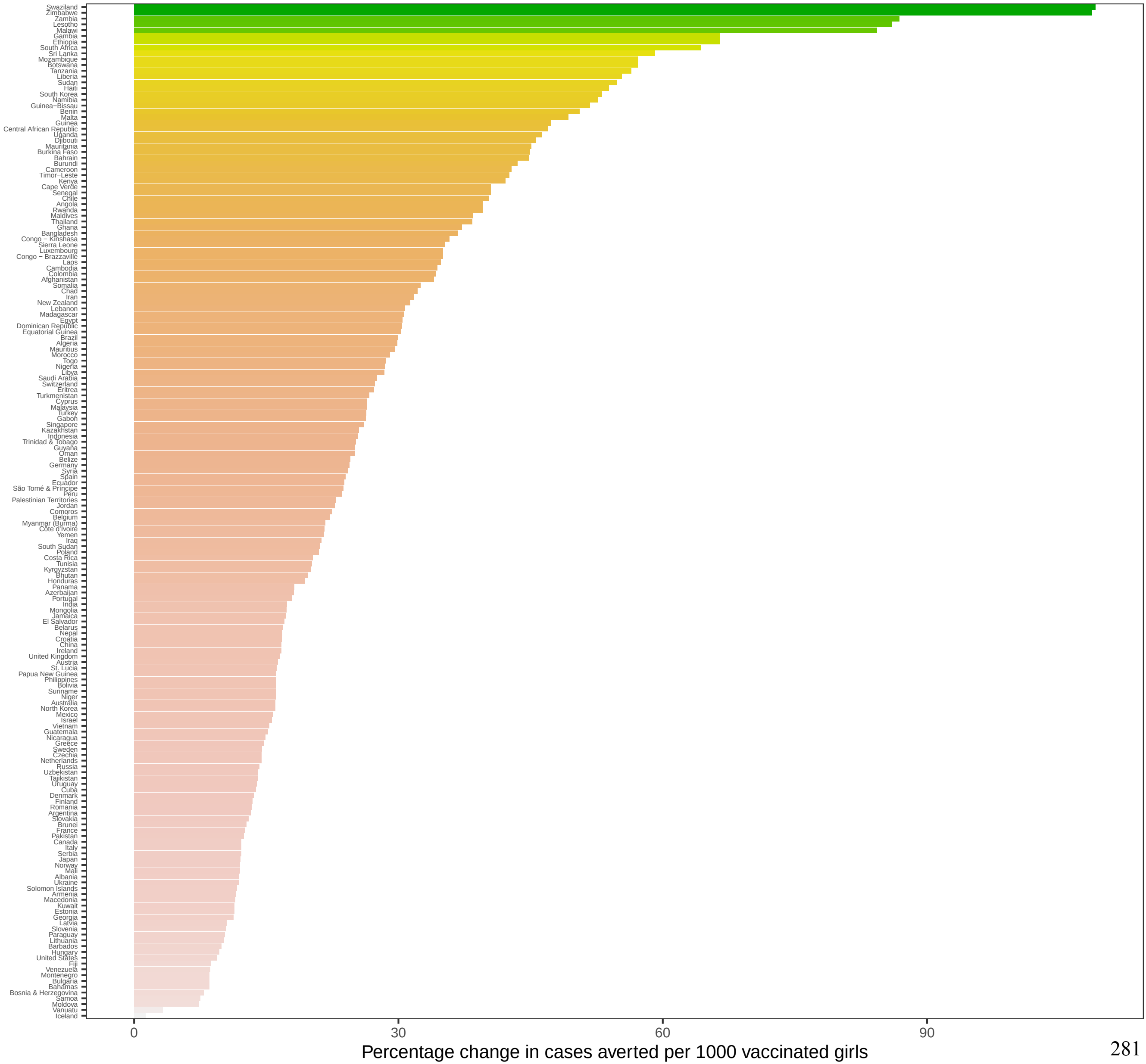

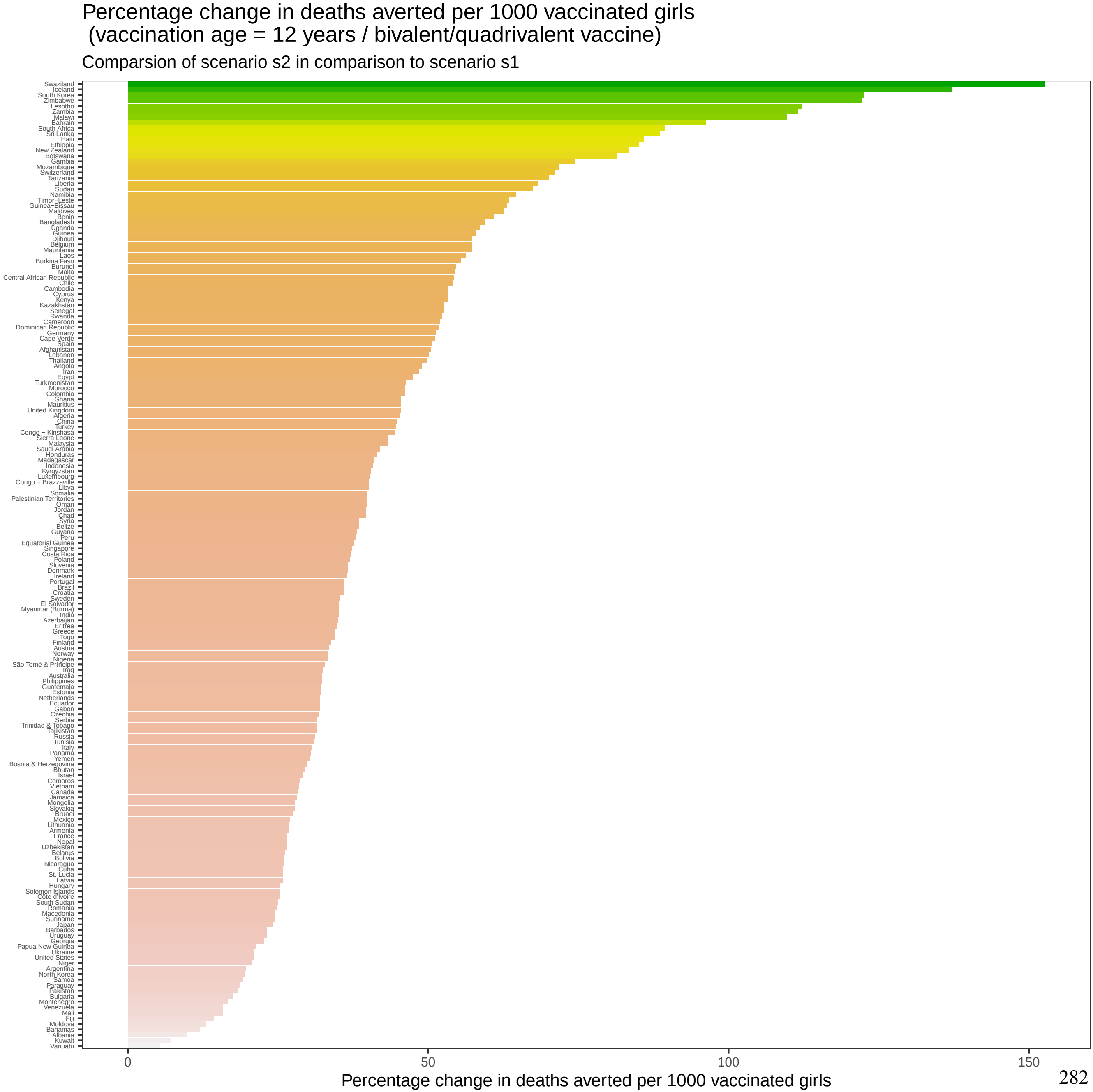
Percentage change in deaths averted per 1000 vaccinated girls
(vaccination age = 12 years / bivalent/quadrivalent vaccine)
Comparsion of scenario s2 in comparison to scenario s1
Swaziland
Iceland
South Korea
Zimbabwe
Lesotho
Zambia
Malawi
Bahrain
South Africa
Sri Lanka
Haiti
Ethiopia
New Zealand
Botswana
Gambia
Mozambique
Switzerland
Tanzania
Liberia
Sudan
Namibia
Timor−Leste
Guinea−Bissau
Maldives
Benin
Bangladesh
Uganda
Guinea
Djibouti
Belgium
Mauritania
Laos
Burkina Faso
Burundi
Malta
Central African Republic
Chile
Cambodia
Cyprus
Kenya
Kazakhstan
Senegal
Rwanda
Cameroon
Dominican Republic
Germany
Cape Verde
Spain
Afghanistan
Lebanon
Thailand
Angola
Iran
Egypt
Turkmenistan
Morocco
Colombia
Ghana
Mauritius
United Kingdom
Algeria
China
Turkey
Congo − Kinshasa
Sierra Leone
Malaysia
Saudi Arabia
Honduras
Madagascar
Indonesia
Kyrgyzstan
Luxembourg
Congo − Brazzaville
Libya
Somalia
Palestinian Territories
Oman
Jordan
Chad
Syria
Belize
Guyana
Peru
Equatorial Guinea
Singapore
Costa Rica
Poland
Slovenia
Denmark
Ireland
Portugal
Brazil
Croatia
Sweden
El Salvador
Myanmar (Burma)
India
Azerbaijan
Eritrea
Greece
Togo
Finland
Austria
Norway
Nigeria
São Tomé & Príncipe
Iraq
Australia
Philippines
Guatemala
Estonia
Netherlands
Ecuador
Gabon
Czechia
Serbia
Trinidad & Tobago
Tajikistan
Russia
Tunisia
Italy
Panama
Yemen
Bosnia & Herzegovina
Bhutan
Israel
Comoros
Vietnam
Canada
Jamaica
Mongolia
Slovakia
Brunei
Mexico
Lithuania
Armenia
France
Nepal
Uzbekistan
Belarus
Bolivia
Nicaragua
Cuba
St. Lucia
Latvia
Hungary
Solomon Islands
Côte d'Ivoire
South Sudan
Romania
Macedonia
Suriname
Japan
Barbados
Uruguay
Georgia
Papua New Guinea
Ukraine
United States
Niger
Argentina
North Korea
Samoa
Paraguay
Pakistan
Bulgaria
Montenegro
Venezuela
Mali
Fiji
Moldova
Bahamas
Albania
Kuwait
Vanuatu
0
50
100
150
Percentage change in deaths averted per 1000 vaccinated girls

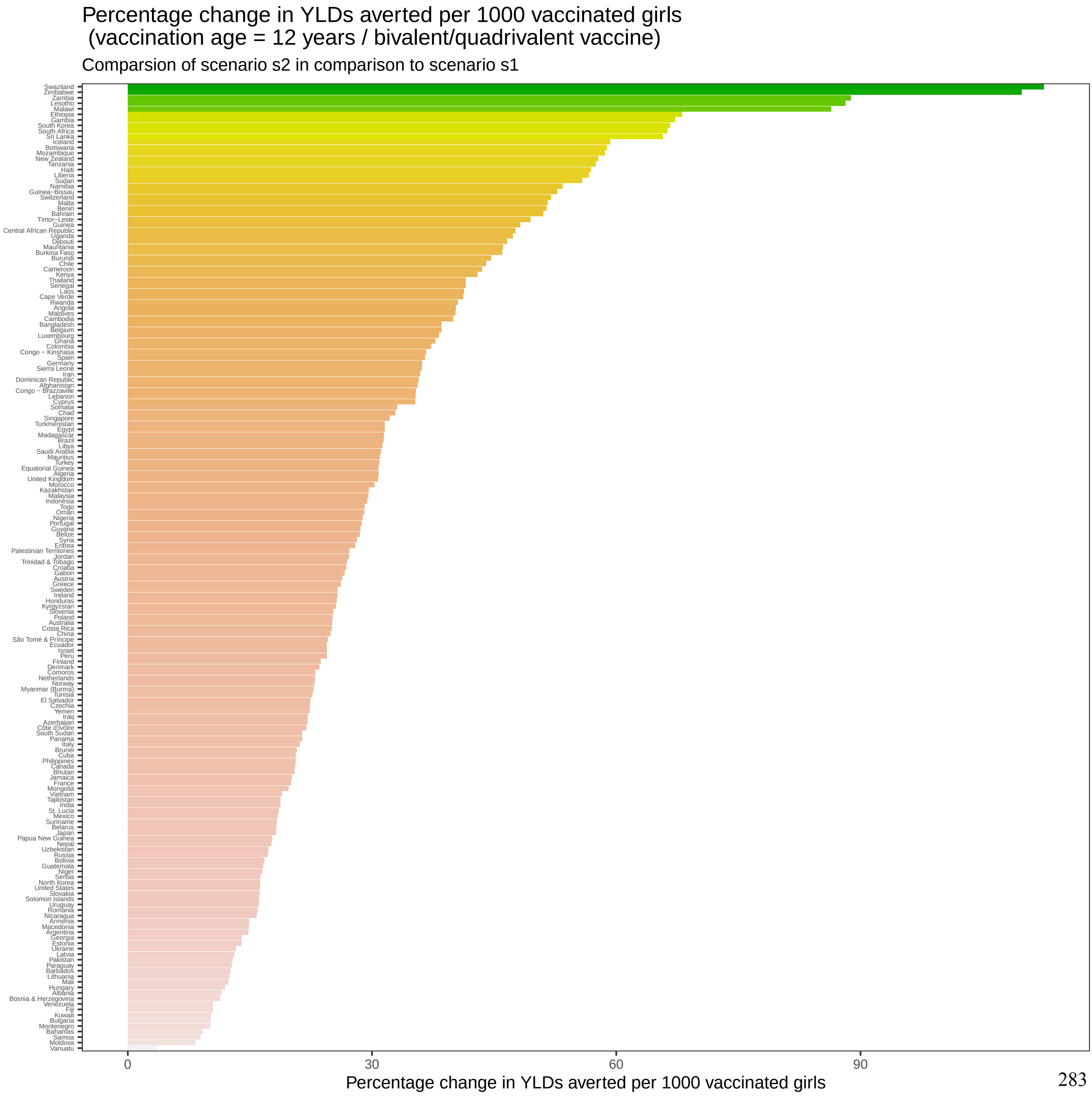
Percentage change in YLDs averted per 1000 vaccinated girls
(vaccination age = 12 years / bivalent/quadrivalent vaccine)
Comparsion of scenario s2 in comparison to scenario s1
Swaziland
Zimbabwe
Zambia
Lesotho
Malawi
Ethiopia
Gambia
South Korea
South Africa
Sri Lanka
Iceland
Botswana
Mozambique
New Zealand
Tanzania
Haiti
Liberia
Sudan
Namibia
Guinea−Bissau
Switzerland
Malta
Benin
Bahrain
Timor−Leste
Guinea
Central African Republic
Uganda
Djibouti
Mauritania
Burkina Faso
Burundi
Chile
Cameroon
Kenya
Thailand
Senegal
Laos
Cape Verde
Rwanda
Angola
Maldives
Cambodia
Bangladesh
Belgium
Luxembourg
Ghana
Colombia
Congo − Kinshasa
Spain
Germany
Sierra Leone
Iran
Dominican Republic
Afghanistan
Congo − Brazzaville
Lebanon
Cyprus
Somalia
Chad
Singapore
Turkmenistan
Egypt
Madagascar
Brazil
Libya
Saudi Arabia
Mauritius
Turkey
Equatorial Guinea
Algeria
United Kingdom
Morocco
Kazakhstan
Malaysia
Indonesia
Togo
Oman
Nigeria
Portugal
Guyana
Belize
Syria
Eritrea
Palestinian Territories
Jordan
Trinidad & Tobago
Croatia
Gabon
Austria
Greece
Sweden
Ireland
Honduras
Kyrgyzstan
Slovenia
Poland
Australia
Costa Rica
China
São Tomé & Príncipe
Ecuador
Israel
Peru
Finland
Denmark
Comoros
Netherlands
Norway
Myanmar (Burma)
Tunisia
El Salvador
Czechia
Yemen
Iraq
Azerbaijan
Côte d'Ivoire
South Sudan
Panama
Italy
Brunei
Cuba
Philippines
Canada
Bhutan
Jamaica
France
Mongolia
Vietnam
Tajikistan
India
St. Lucia
Mexico
Suriname
Belarus
Japan
Papua New Guinea
Nepal
Uzbekistan
Russia
Bolivia
Guatemala
Niger
Serbia
North Korea
United States
Slovakia
Solomon Islands
Uruguay
Romania
Nicaragua
Armenia
Macedonia
Argentina
Georgia
Estonia
Ukraine
Latvia
Pakistan
Paraguay
Barbados
Lithuania
Mali
Hungary
Albania
Bosnia & Herzegovina
Venezuela
Fiji
Kuwait
Bulgaria
Montenegro
Bahamas
Samoa
Moldova
Vanuatu
0
30
60
90
Percentage change in YLDs averted per 1000 vaccinated girls

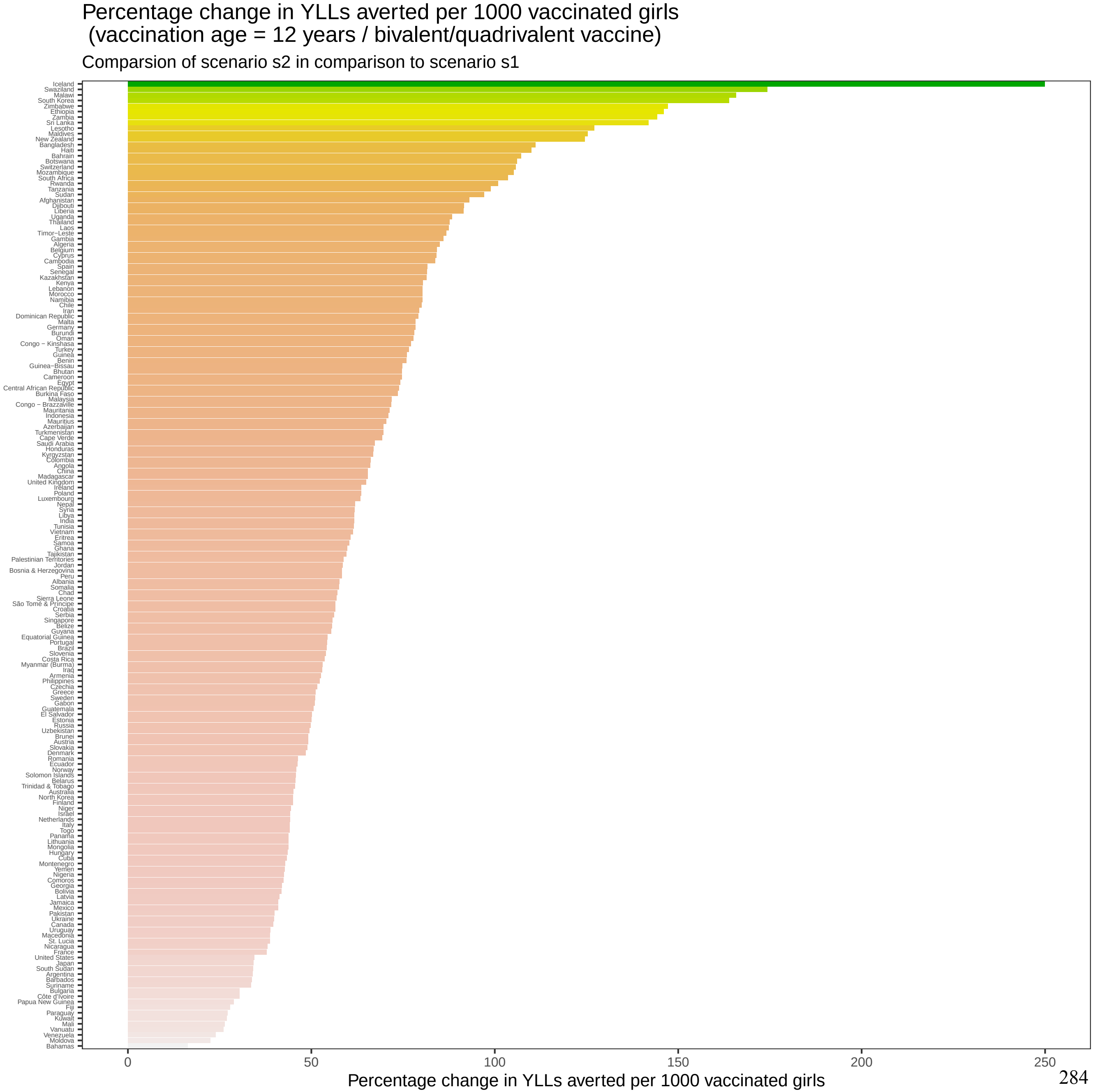
Percentage change in YLLs averted per 1000 vaccinated girls
(vaccination age = 12 years / bivalent/quadrivalent vaccine)
Comparsion of scenario s2 in comparison to scenario s1
Iceland
Swaziland
Malawi
South Korea
Zimbabwe
Ethiopia
Zambia
Sri Lanka
Lesotho
Maldives
New Zealand
Bangladesh
Haiti
Bahrain
Botswana
Switzerland
Mozambique
South Africa
Rwanda
Tanzania
Sudan
Afghanistan
Djibouti
Liberia
Uganda
Thailand
Laos
Timor−Leste
Gambia
Algeria
Belgium
Cyprus
Cambodia
Spain
Senegal
Kazakhstan
Kenya
Lebanon
Morocco
Namibia
Chile
Iran
Dominican Republic
Malta
Germany
Burundi
Oman
Congo − Kinshasa
Turkey
Guinea
Benin
Guinea−Bissau
Bhutan
Cameroon
Egypt
Central African Republic
Burkina Faso
Malaysia
Congo − Brazzaville
Mauritania
Indonesia
Mauritius
Azerbaijan
Turkmenistan
Cape Verde
Saudi Arabia
Honduras
Kyrgyzstan
Colombia
Angola
China
Madagascar
United Kingdom
Ireland
Poland
Luxembourg
Nepal
Syria
Libya
India
Tunisia
Vietnam
Eritrea
Samoa
Ghana
Tajikistan
Palestinian Territories
Jordan
Bosnia & Herzegovina
Peru
Albania
Somalia
Chad
Sierra Leone
São Tomé & Príncipe
Croatia
Serbia
Singapore
Belize
Guyana
Equatorial Guinea
Portugal
Brazil
Slovenia
Costa Rica
Myanmar (Burma)
Iraq
Armenia
Philippines
Czechia
Greece
Sweden
Gabon
Guatemala
El Salvador
Estonia
Russia
Uzbekistan
Brunei
Austria
Slovakia
Denmark
Romania
Ecuador
Norway
Solomon Islands
Belarus
Trinidad & Tobago
Australia
North Korea
Finland
Niger
Israel
Netherlands
Italy
Togo
Panama
Lithuania
Mongolia
Hungary
Cuba
Montenegro
Yemen
Nigeria
Comoros
Georgia
Bolivia
Latvia
Jamaica
Mexico
Pakistan
Ukraine
Canada
Uruguay
Macedonia
St. Lucia
Nicaragua
France
United States
Japan
South Sudan
Argentina
Barbados
Suriname
Bulgaria
Côte d'Ivoire
Papua New Guinea
Fiji
Paraguay
Kuwait
Mali
Vanuatu
Venezuela
Moldova
Bahamas
0
50
100
150
200
250
Percentage change in YLLs averted per 1000 vaccinated girls

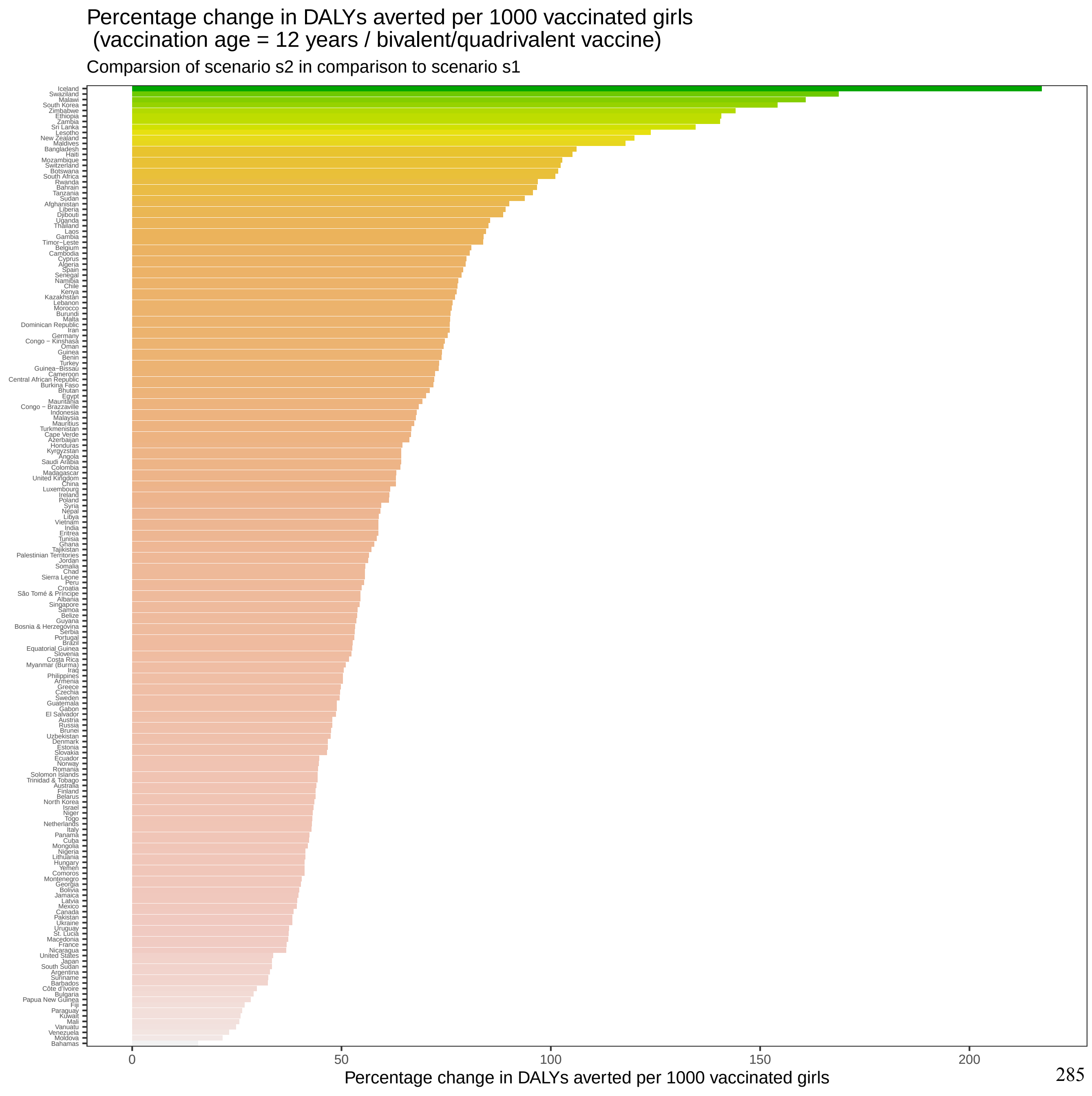
Percentage change in DALYs averted per 1000 vaccinated girls
(vaccination age = 12 years / bivalent/quadrivalent vaccine)
Comparsion of scenario s2 in comparison to scenario s1
Iceland
Swaziland
Malawi
South Korea
Zimbabwe
Ethiopia
Zambia
Sri Lanka
Lesotho
New Zealand
Maldives
Bangladesh
Haiti
Mozambique
Switzerland
Botswana
South Africa
Rwanda
Bahrain
Tanzania
Sudan
Afghanistan
Liberia
Djibouti
Uganda
Thailand
Laos
Gambia
Timor−Leste
Belgium
Cambodia
Cyprus
Algeria
Spain
Senegal
Namibia
Chile
Kenya
Kazakhstan
Lebanon
Morocco
Burundi
Malta
Dominican Republic
Iran
Germany
Congo − Kinshasa
Oman
Guinea
Benin
Turkey
Guinea−Bissau
Cameroon
Central African Republic
Burkina Faso
Bhutan
Egypt
Mauritania
Congo − Brazzaville
Indonesia
Malaysia
Mauritius
Turkmenistan
Cape Verde
Azerbaijan
Honduras
Kyrgyzstan
Angola
Saudi Arabia
Colombia
Madagascar
United Kingdom
China
Luxembourg
Ireland
Poland
Syria
Nepal
Libya
Vietnam
India
Eritrea
Tunisia
Ghana
Tajikistan
Palestinian Territories
Jordan
Somalia
Chad
Sierra Leone
Peru
Croatia
São Tomé & Príncipe
Albania
Singapore
Samoa
Belize
Guyana
Bosnia & Herzegovina
Serbia
Portugal
Brazil
Equatorial Guinea
Slovenia
Costa Rica
Myanmar (Burma)
Iraq
Philippines
Armenia
Greece
Czechia
Sweden
Guatemala
Gabon
El Salvador
Austria
Russia
Brunei
Uzbekistan
Denmark
Estonia
Slovakia
Ecuador
Norway
Romania
Solomon Islands
Trinidad & Tobago
Australia
Finland
Belarus
North Korea
Israel
Niger
Togo
Netherlands
Italy
Panama
Cuba
Mongolia
Nigeria
Lithuania
Hungary
Yemen
Comoros
Montenegro
Georgia
Bolivia
Jamaica
Latvia
Mexico
Canada
Pakistan
Ukraine
Uruguay
St. Lucia
Macedonia
France
Nicaragua
United States
Japan
South Sudan
Argentina
Suriname
Barbados
Côte d'Ivoire
Bulgaria
Papua New Guinea
Fiji
Paraguay
Kuwait
Mali
Vanuatu
Venezuela
Moldova
Bahamas
0
50
100
150
200
Percentage change in DALYs averted per 1000 vaccinated girls

Percentage change in cases averted per 1000 vaccinated girls
(vaccination age = 12 years / bivalent/quadrivalent vaccine)

Comparsion of scenario s3 in comparison to scenario s1

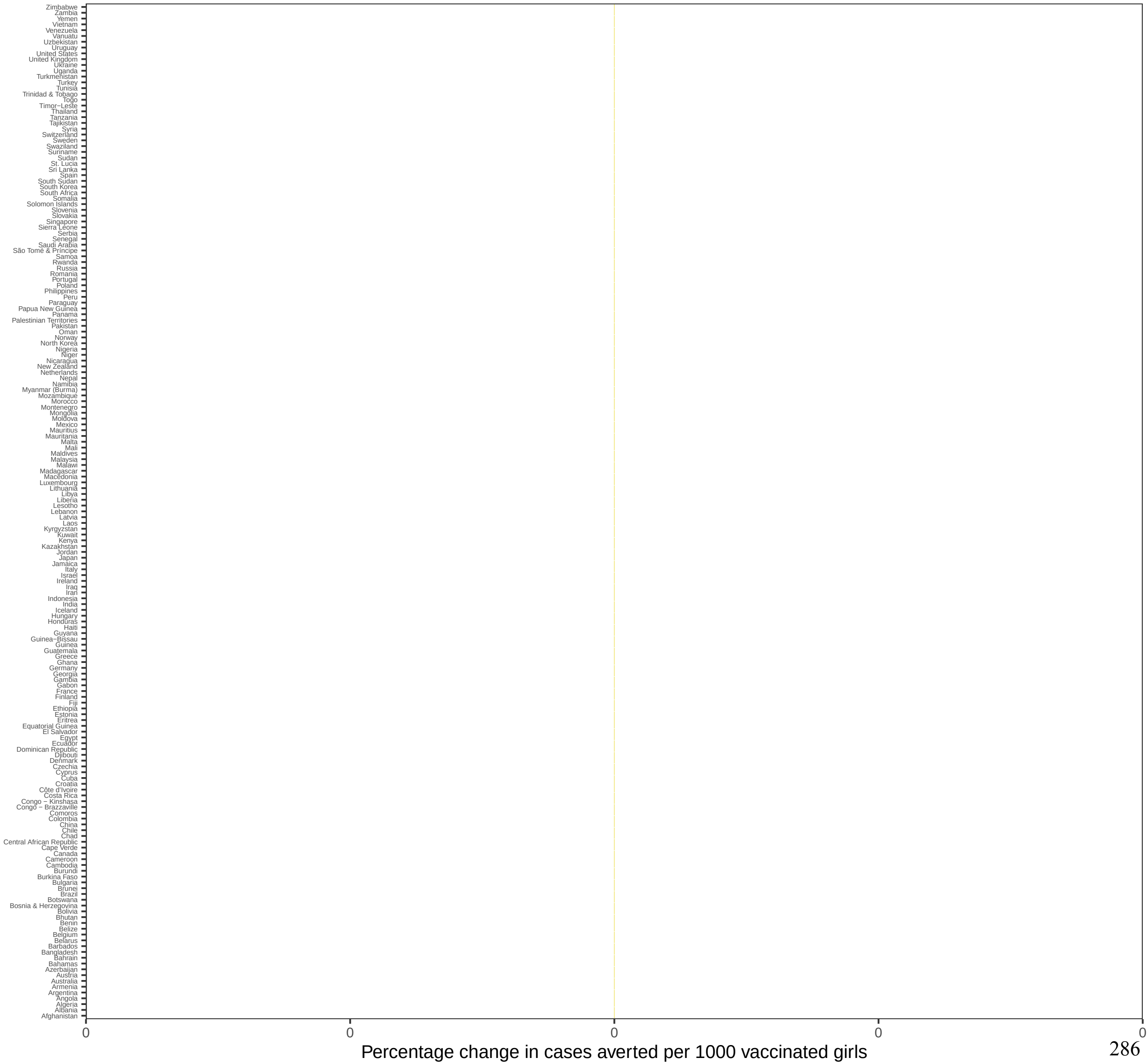

Percentage change in deaths averted per 1000 vaccinated girls
(vaccination age = 12 years / bivalent/quadrivalent vaccine)

Comparsion of scenario s3 in comparison to scenario s1

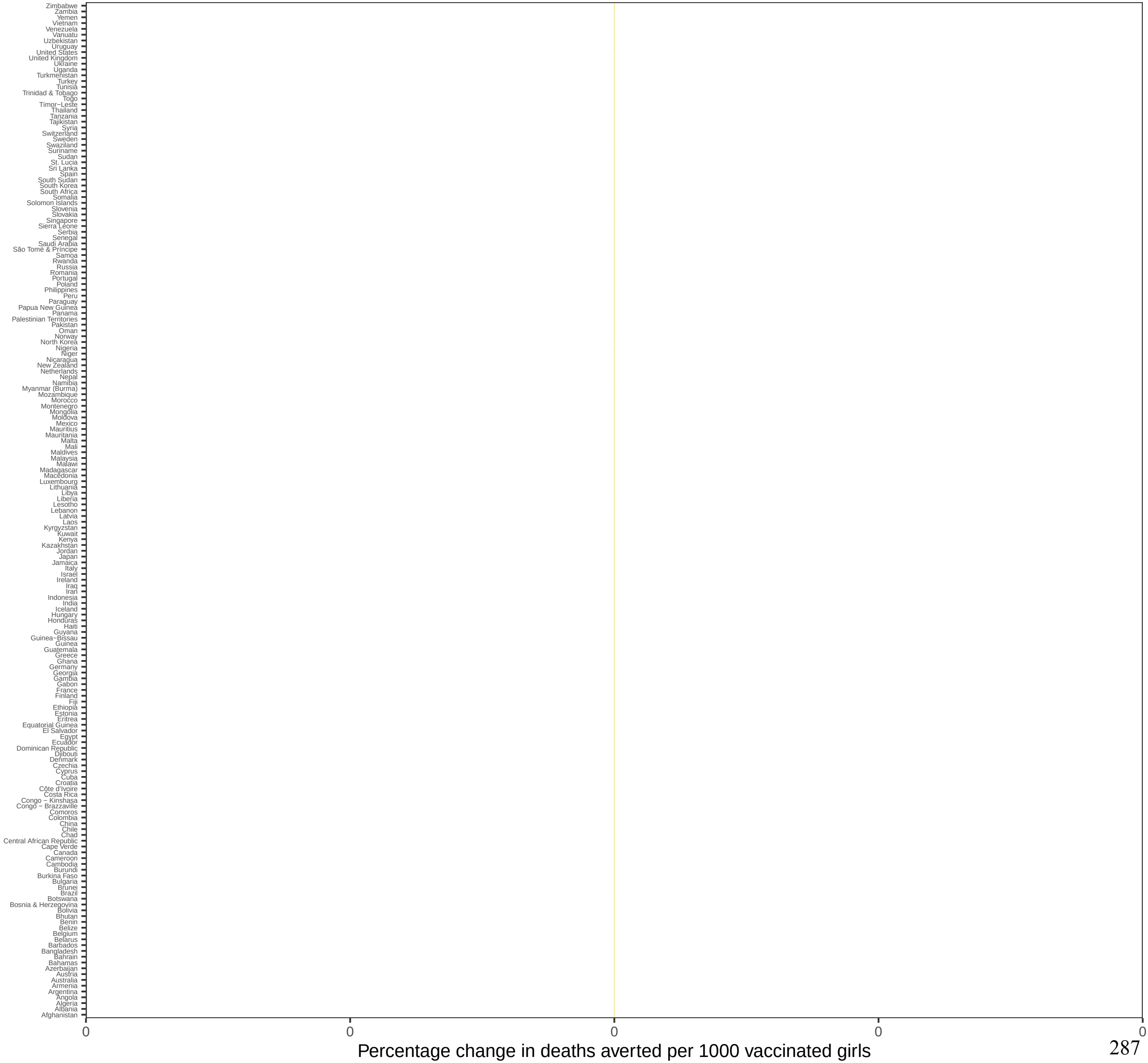

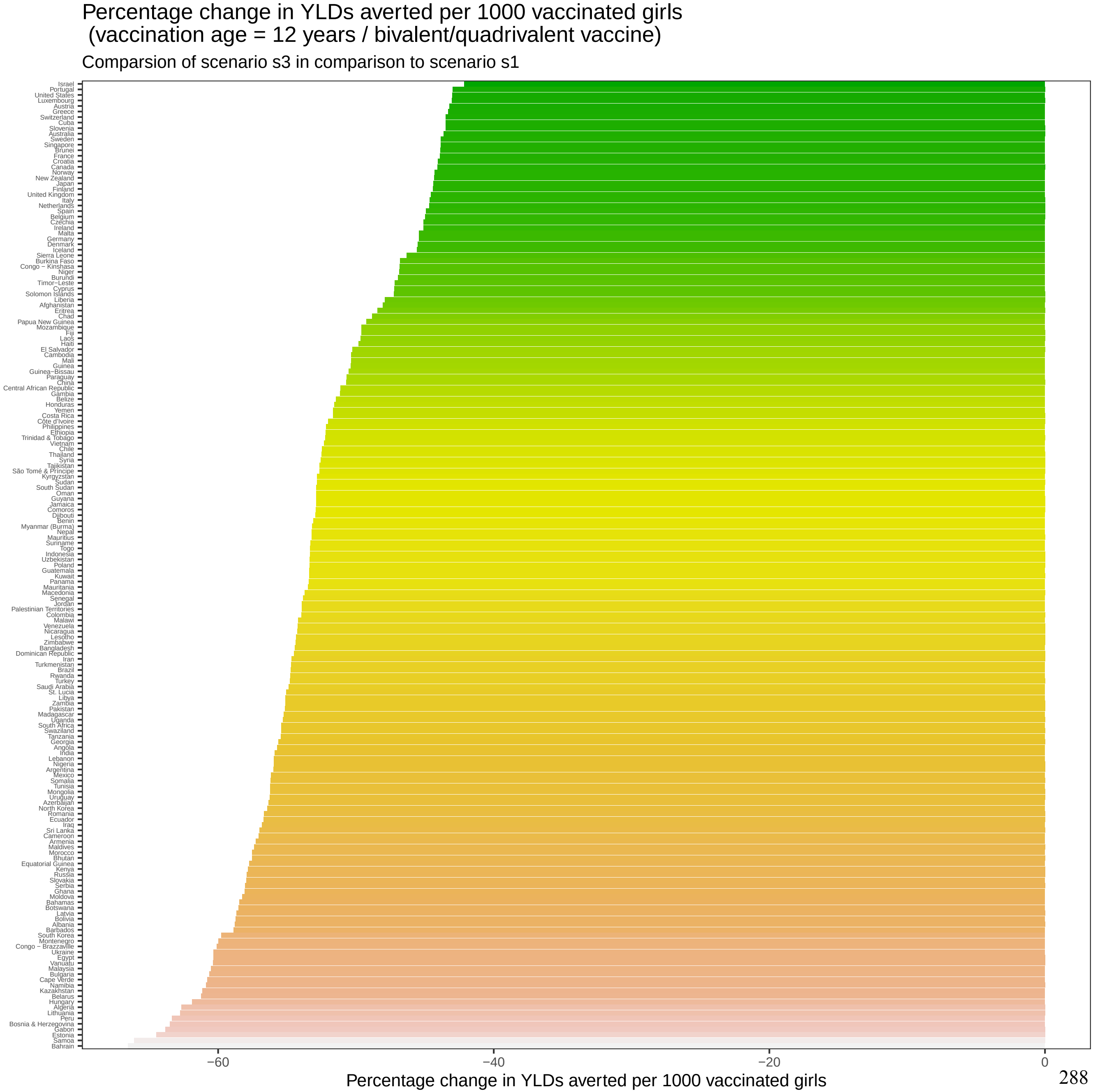
Percentage change in YLDs averted per 1000 vaccinated girls
(vaccination age = 12 years / bivalent/quadrivalent vaccine)
Comparsion of scenario s3 in comparison to scenario s1
Israel
Portugal
United States
Luxembourg
Austria
Greece
Switzerland
Cuba
Slovenia
Australia
Sweden
Singapore
Brunei
France
Croatia
Canada
Norway
New Zealand
Japan
Finland
United Kingdom
Italy
Netherlands
Spain
Belgium
Czechia
Ireland
Malta
Germany
Denmark
Iceland
Sierra Leone
Burkina Faso
Congo – Kinshasa
Niger
Burundi
Timor–Leste
Cyprus
Solomon Islands
Liberia
Afghanistan
Eritrea
Chad
Papua New Guinea
Mozambique
Fiji
Laos
Haiti
El Salvador
Cambodia
Mali
Guinea
Guinea–Bissau
Paraguay
China
Central African Republic
Gambia
Belize
Honduras
Yemen
Costa Rica
Côte d'Ivoire
Philippines
Ethiopia
Trinidad & Tobago
Vietnam
Chile
Thailand
Syria
Tajikistan
São Tomé & Príncipe
Kyrgyzstan
Sudan
South Sudan
Oman
Guyana
Jamaica
Comoros
Djibouti
Benin
Myanmar (Burma)
Nepal
Mauritius
Suriname
Togo
Indonesia
Uzbekistan
Poland
Guatemala
Kuwait
Panama
Mauritania
Macedonia
Senegal
Jordan
Palestinian Territories
Colombia
Malawi
Venezuela
Nicaragua
Lesotho
Zimbabwe
Bangladesh
Dominican Republic
Iran
Turkmenistan
Brazil
Rwanda
Turkey
Saudi Arabia
St. Lucia
Libya
Zambia
Pakistan
Madagascar
Uganda
South Africa
Swaziland
Tanzania
Georgia
Angola
India
Lebanon
Nigeria
Argentina
Mexico
Somalia
Tunisia
Mongolia
Uruguay
Azerbaijan
North Korea
Romania
Ecuador
Iraq
Sri Lanka
Cameroon
Armenia
Maldives
Morocco
Bhutan
Equatorial Guinea
Kenya
Russia
Slovakia
Serbia
Ghana
Moldova
Bahamas
Botswana
Latvia
Bolivia
Albania
Barbados
South Korea
Montenegro
Congo – Brazzaville
Ukraine
Egypt
Vanuatu
Malaysia
Bulgaria
Cape Verde
Namibia
Kazakhstan
Belarus
Hungary
Algeria
Lithuania
Peru
Bosnia & Herzegovina
Gabon
Estonia
Samoa
Bahrain
−60
−40
−20
0
Percentage change in YLDs averted per 1000 vaccinated girls

Percentage change in YLLs averted per 1000 vaccinated girls
(vaccination age = 12 years / bivalent/quadrivalent vaccine)

Comparsion of scenario s3 in comparison to scenario s1

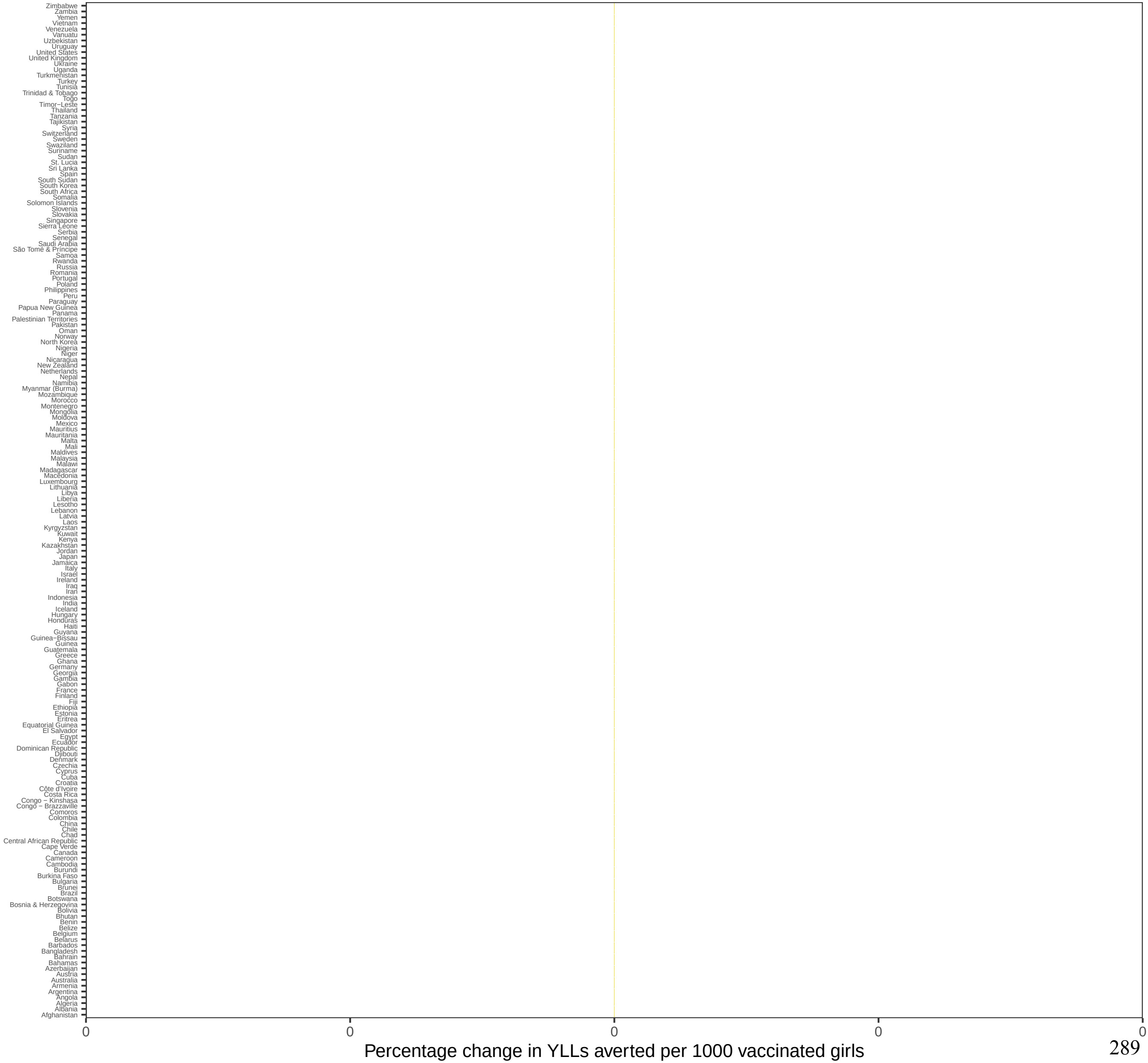

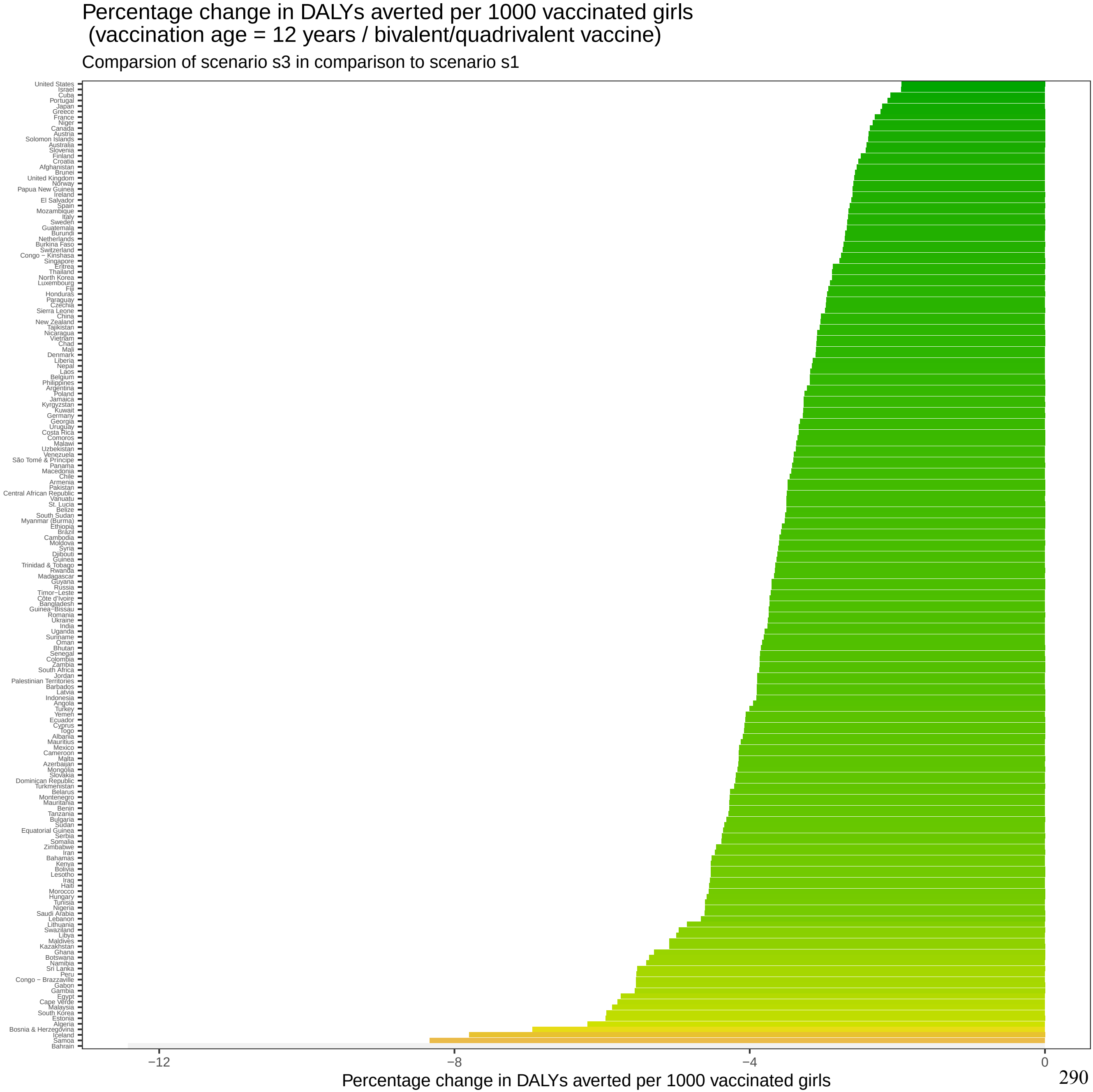
Percentage change in DALYs averted per 1000 vaccinated girls
(vaccination age = 12 years / bivalent/quadrivalent vaccine)
Comparsion of scenario s3 in comparison to scenario s1
United States
Israel
Cuba
Portugal
Japan
Greece
France
Niger
Canada
Austria
Solomon Islands
Australia
Slovenia
Finland
Croatia
Afghanistan
Brunei
United Kingdom
Norway
Papua New Guinea
Ireland
El Salvador
Spain
Mozambique
Italy
Sweden
Guatemala
Burundi
Netherlands
Burkina Faso
Switzerland
Congo − Kinshasa
Singapore
Eritrea
Thailand
North Korea
Luxembourg
Fiji
Honduras
Paraguay
Czechia
Sierra Leone
China
New Zealand
Tajikistan
Nicaragua
Vietnam
Chad
Mali
Denmark
Liberia
Nepal
Laos
Belgium
Philippines
Argentina
Poland
Jamaica
Kyrgyzstan
Kuwait
Germany
Georgia
Uruguay
Costa Rica
Comoros
Malawi
Uzbekistan
Venezuela
São Tomé & Príncipe
Panama
Macedonia
Chile
Armenia
Pakistan
Central African Republic
Vanuatu
St. Lucia
Belize
South Sudan
Myanmar (Burma)
Ethiopia
Brazil
Cambodia
Moldova
Syria
Djibouti
Guinea
Trinidad & Tobago
Rwanda
Madagascar
Guyana
Russia
Timor−Leste
Côte d'Ivoire
Bangladesh
Guinea−Bissau
Romania
Ukraine
India
Uganda
Suriname
Oman
Bhutan
Senegal
Colombia
Zambia
South Africa
Jordan
Palestinian Territories
Barbados
Latvia
Indonesia
Angola
Turkey
Yemen
Ecuador
Cyprus
Togo
Albania
Mauritius
Mexico
Cameroon
Malta
Azerbaijan
Mongolia
Slovakia
Dominican Republic
Turkmenistan
Belarus
Montenegro
Mauritania
Benin
Tanzania
Bulgaria
Sudan
Equatorial Guinea
Serbia
Somalia
Zimbabwe
Iran
Bahamas
Kenya
Bolivia
Lesotho
Iraq
Haiti
Morocco
Hungary
Tunisia
Nigeria
Saudi Arabia
Lebanon
Lithuania
Swaziland
Libya
Maldives
Kazakhstan
Ghana
Botswana
Namibia
Sri Lanka
Peru
Congo − Brazzaville
Gabon
Gambia
Egypt
Cape Verde
Malaysia
South Korea
Estonia
Algeria
Bosnia & Herzegovina
Iceland
Samoa
Bahrain
−12
−8
−4
0
Percentage change in DALYs averted per 1000 vaccinated girls

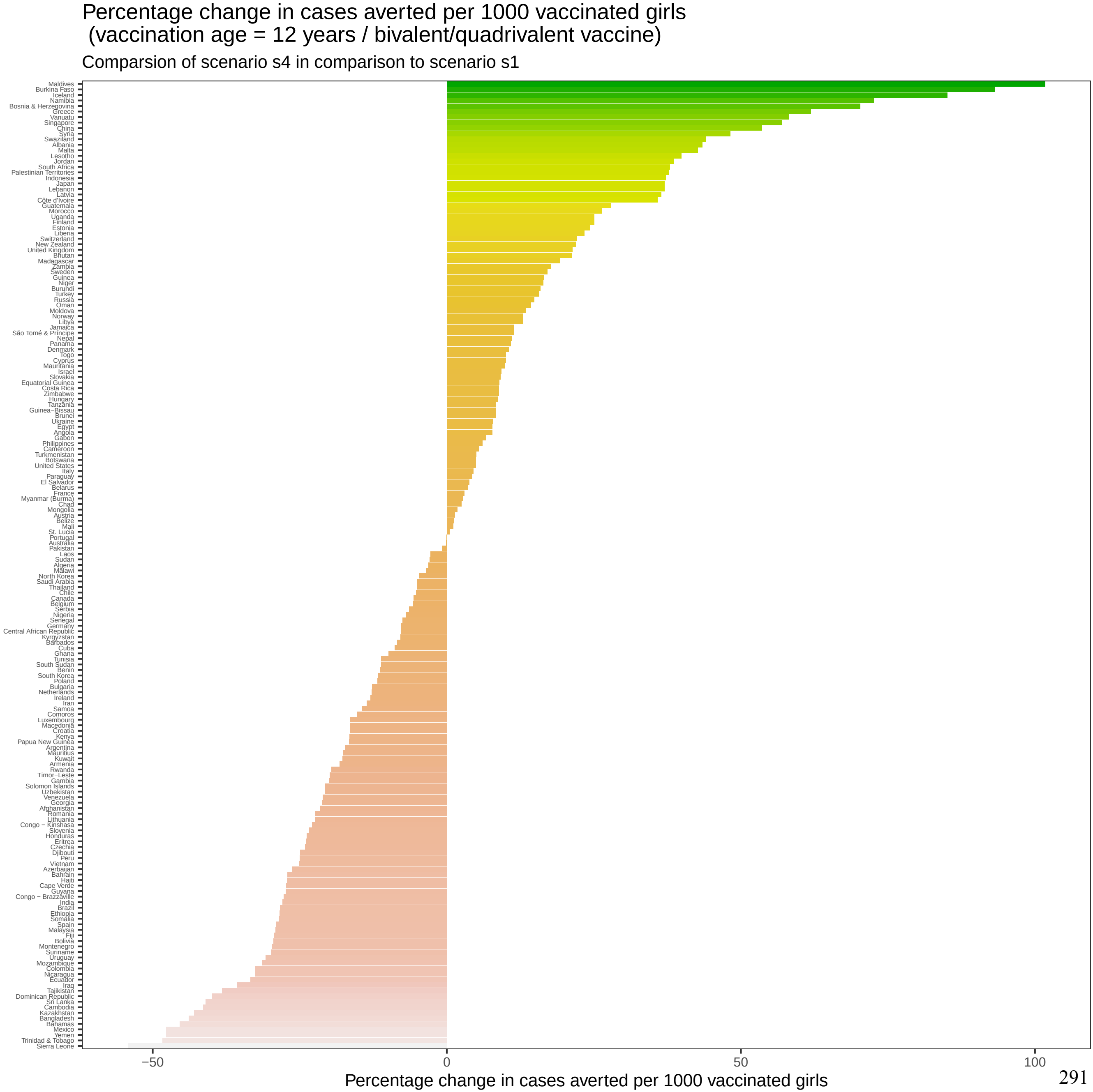
Percentage change in cases averted per 1000 vaccinated girls
(vaccination age = 12 years / bivalent/quadrivalent vaccine)
Comparsion of scenario s4 in comparison to scenario s1
Maldives
Burkina Faso
Iceland
Namibia
Bosnia & Herzegovina
Greece
Vanuatu
Singapore
China
Syria
Swaziland
Albania
Malta
Lesotho
Jordan
South Africa
Palestinian Territories
Indonesia
Japan
Lebanon
Latvia
Côte d'Ivoire
Guatemala
Morocco
Uganda
Finland
Estonia
Liberia
Switzerland
New Zealand
United Kingdom
Bhutan
Madagascar
Zambia
Sweden
Guinea
Niger
Burundi
Turkey
Russia
Oman
Moldova
Norway
Libya
Jamaica
São Tomé & Príncipe
Nepal
Panama
Denmark
Togo
Cyprus
Mauritania
Israel
Slovakia
Equatorial Guinea
Costa Rica
Zimbabwe
Hungary
Tanzania
Guinea−Bissau
Brunei
Ukraine
Egypt
Angola
Gabon
Philippines
Cameroon
Turkmenistan
Botswana
United States
Italy
Paraguay
El Salvador
Belarus
France
Myanmar (Burma)
Chad
Mongolia
Austria
Belize
Mali
St. Lucia
Portugal
Australia
Pakistan
Laos
Sudan
Algeria
Malawi
North Korea
Saudi Arabia
Thailand
Chile
Canada
Belgium
Serbia
Nigeria
Senegal
Germany
Central African Republic
Kyrgyzstan
Barbados
Cuba
Ghana
Tunisia
South Sudan
Benin
South Korea
Poland
Bulgaria
Netherlands
Ireland
Iran
Samoa
Comoros
Luxembourg
Macedonia
Croatia
Kenya
Papua New Guinea
Argentina
Mauritius
Kuwait
Armenia
Rwanda
Timor−Leste
Gambia
Solomon Islands
Uzbekistan
Venezuela
Georgia
Afghanistan
Romania
Lithuania
Congo − Kinshasa
Slovenia
Honduras
Eritrea
Czechia
Djibouti
Peru
Vietnam
Azerbaijan
Bahrain
Haiti
Cape Verde
Guyana
Congo − Brazzaville
India
Brazil
Ethiopia
Somalia
Spain
Malaysia
Fiji
Bolivia
Montenegro
Suriname
Uruguay
Mozambique
Colombia
Nicaragua
Ecuador
Iraq
Tajikistan
Dominican Republic
Sri Lanka
Cambodia
Kazakhstan
Bangladesh
Bahamas
Mexico
Yemen
Trinidad & Tobago
Sierra Leone
−50
0
50
100
Percentage change in cases averted per 1000 vaccinated girls

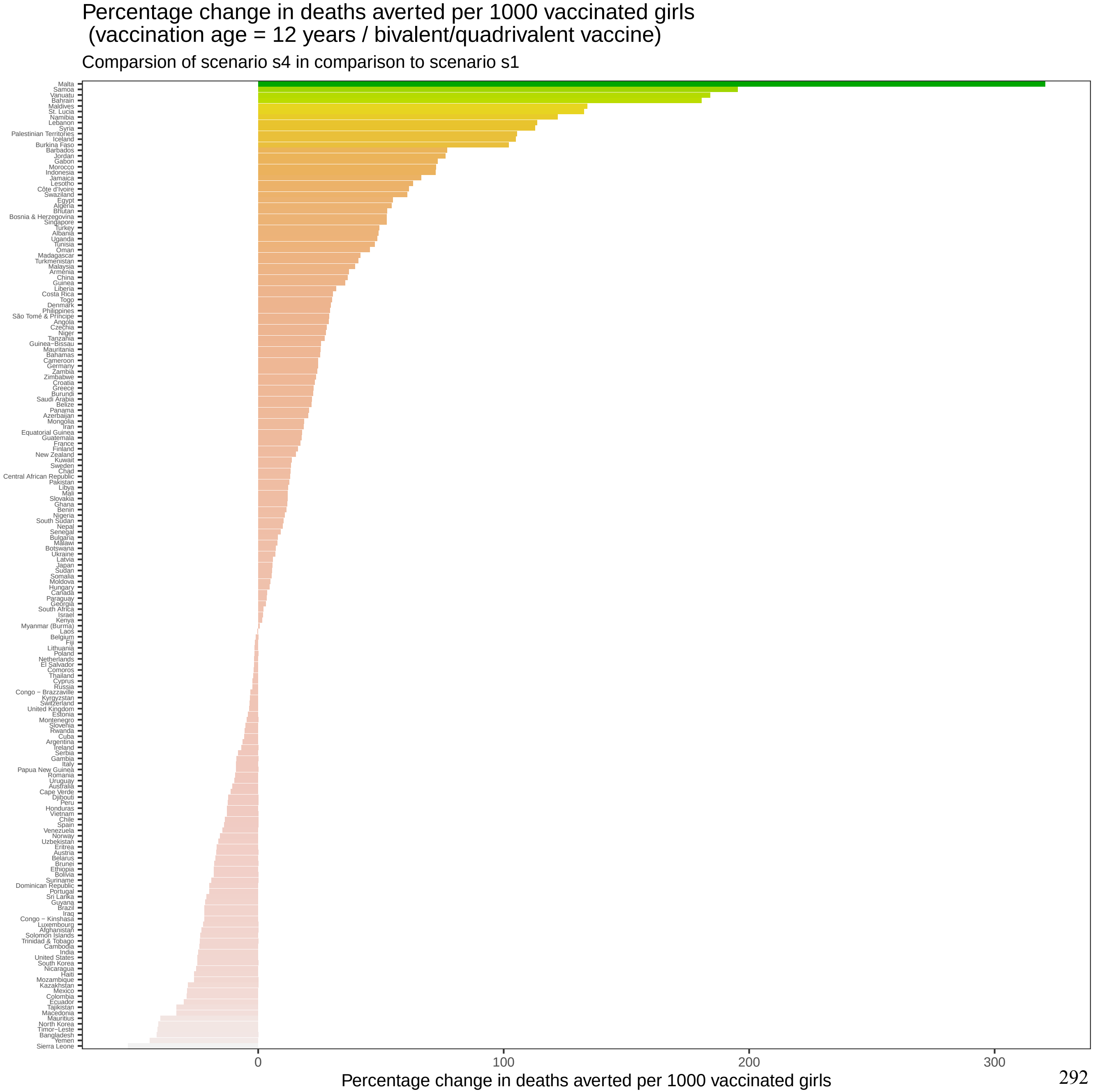
Percentage change in deaths averted per 1000 vaccinated girls
(vaccination age = 12 years / bivalent/quadrivalent vaccine)
Comparsion of scenario s4 in comparison to scenario s1
Percentage change in deaths averted per 1000 vaccinated girls
0
100
200
300

Percentage change in YLDs averted per 1000 vaccinated girls
(vaccination age = 12 years / bivalent/quadrivalent vaccine)

Comparsion of scenario s4 in comparison to scenario s1

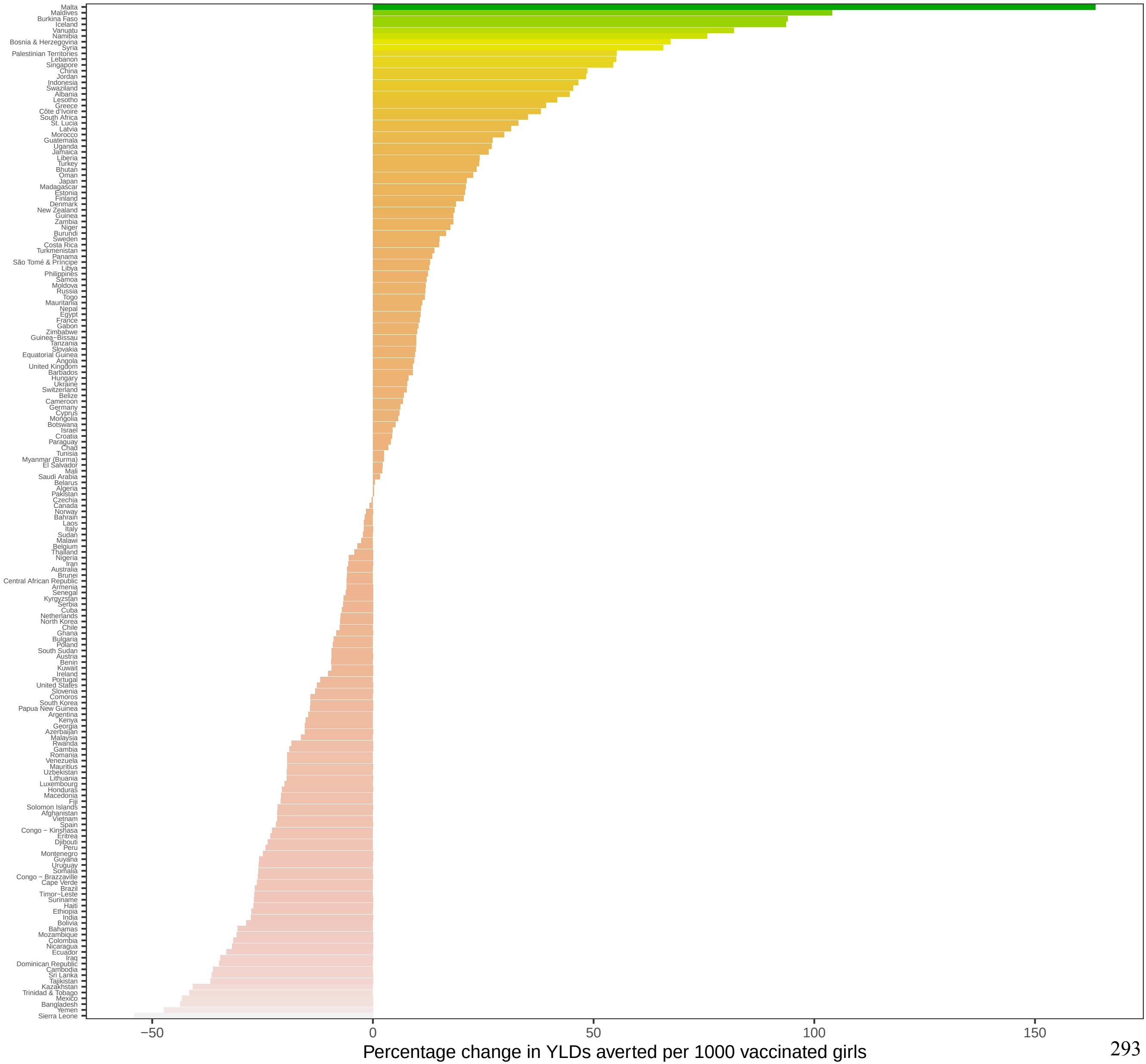

Percentage change in YLLs averted per 1000 vaccinated girls
(vaccination age = 12 years / bivalent/quadrivalent vaccine)

Comparsion of scenario s4 in comparison to scenario s1

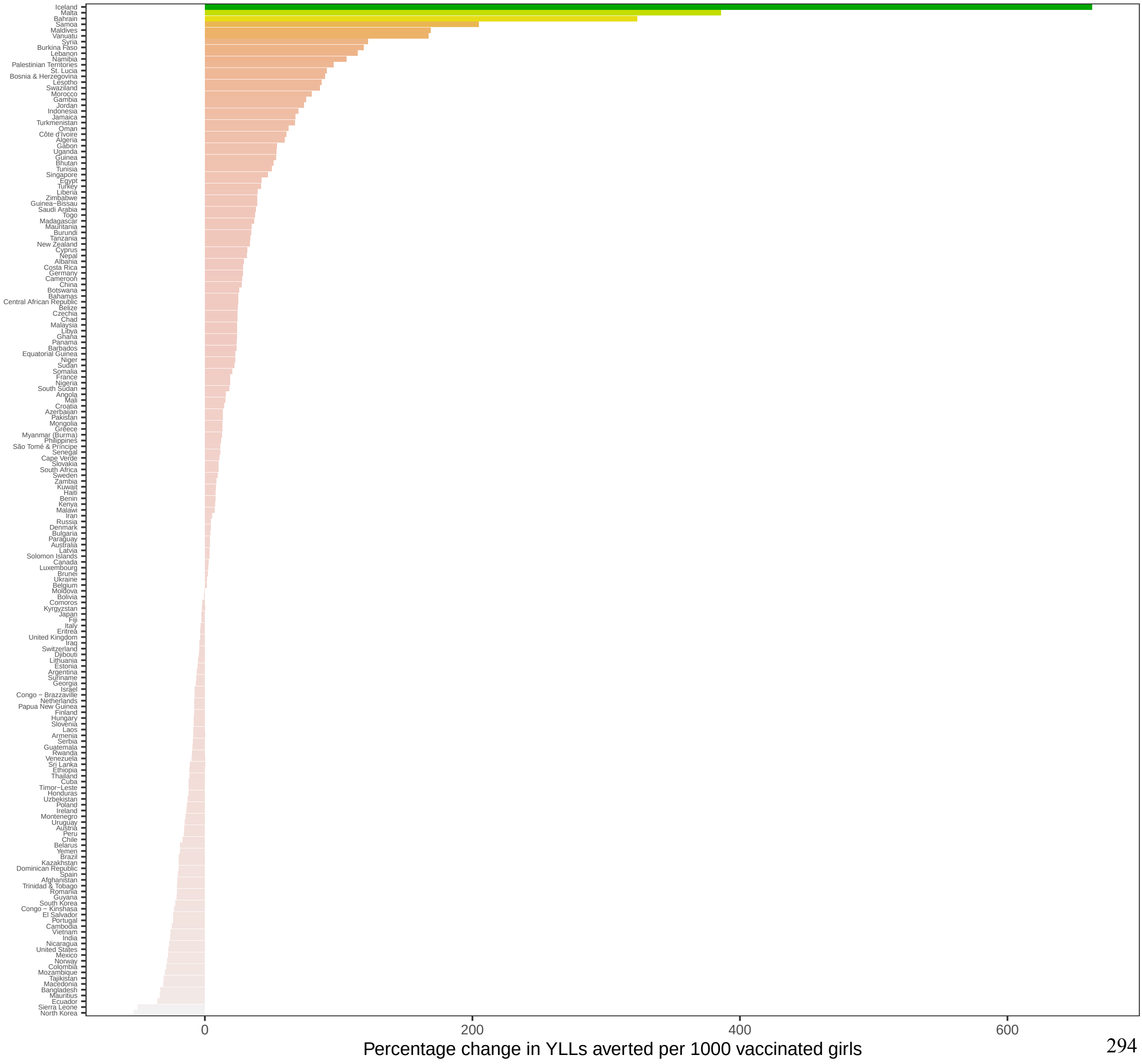

Percentage change in DALYs averted per 1000 vaccinated girls
 (vaccination age = 12 years / bivalent/quadrivalent vaccine)

Comparsion of scenario s4 in comparison to scenario s1

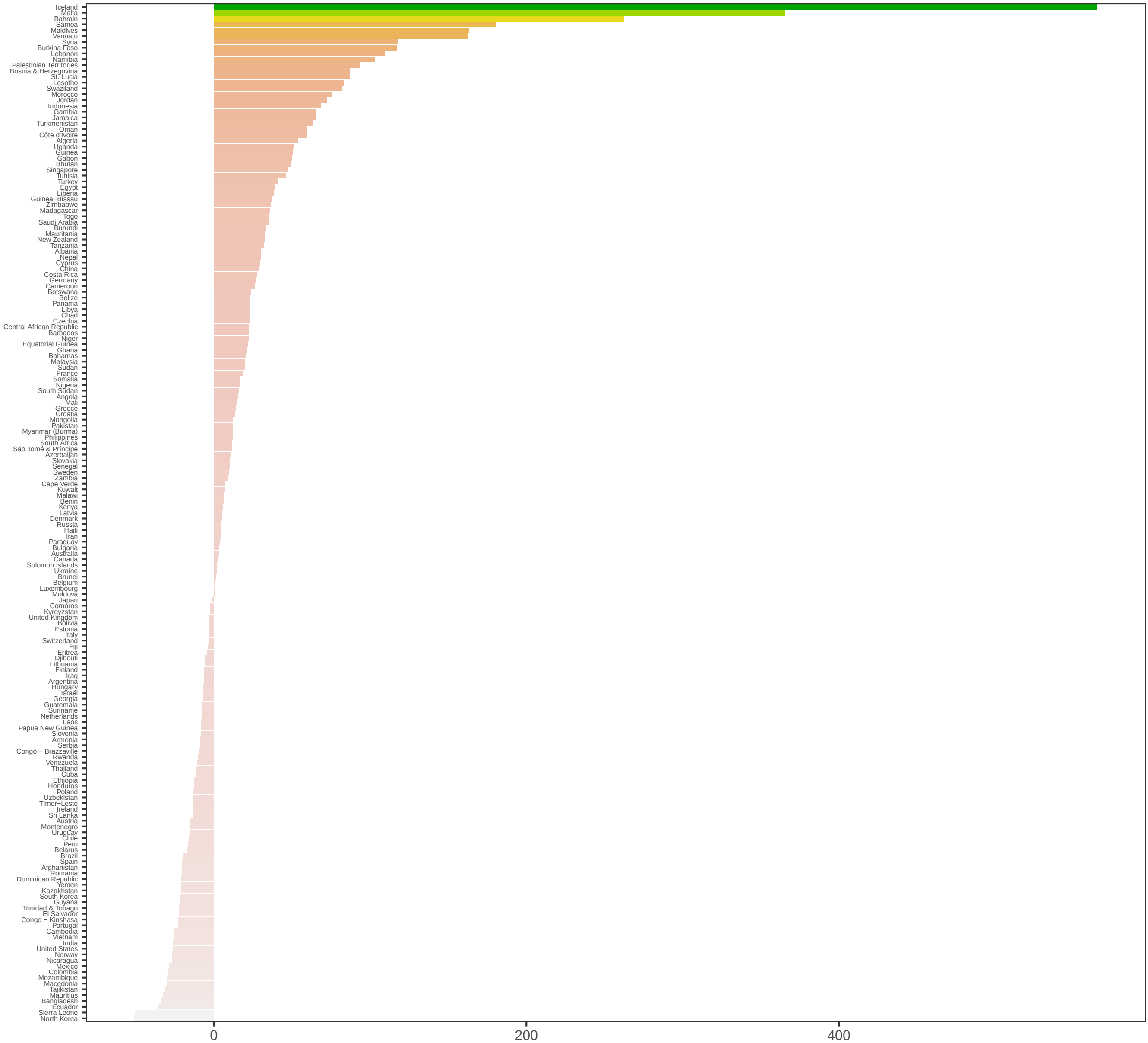

Percentage change in DALYs averted per 1000 vaccinated girls

Percentage change in cases averted per 1000 vaccinated girls
(vaccination age = 12 years / bivalent/quadrivalent vaccine)

Comparsion of scenario s5 in comparison to scenario s1

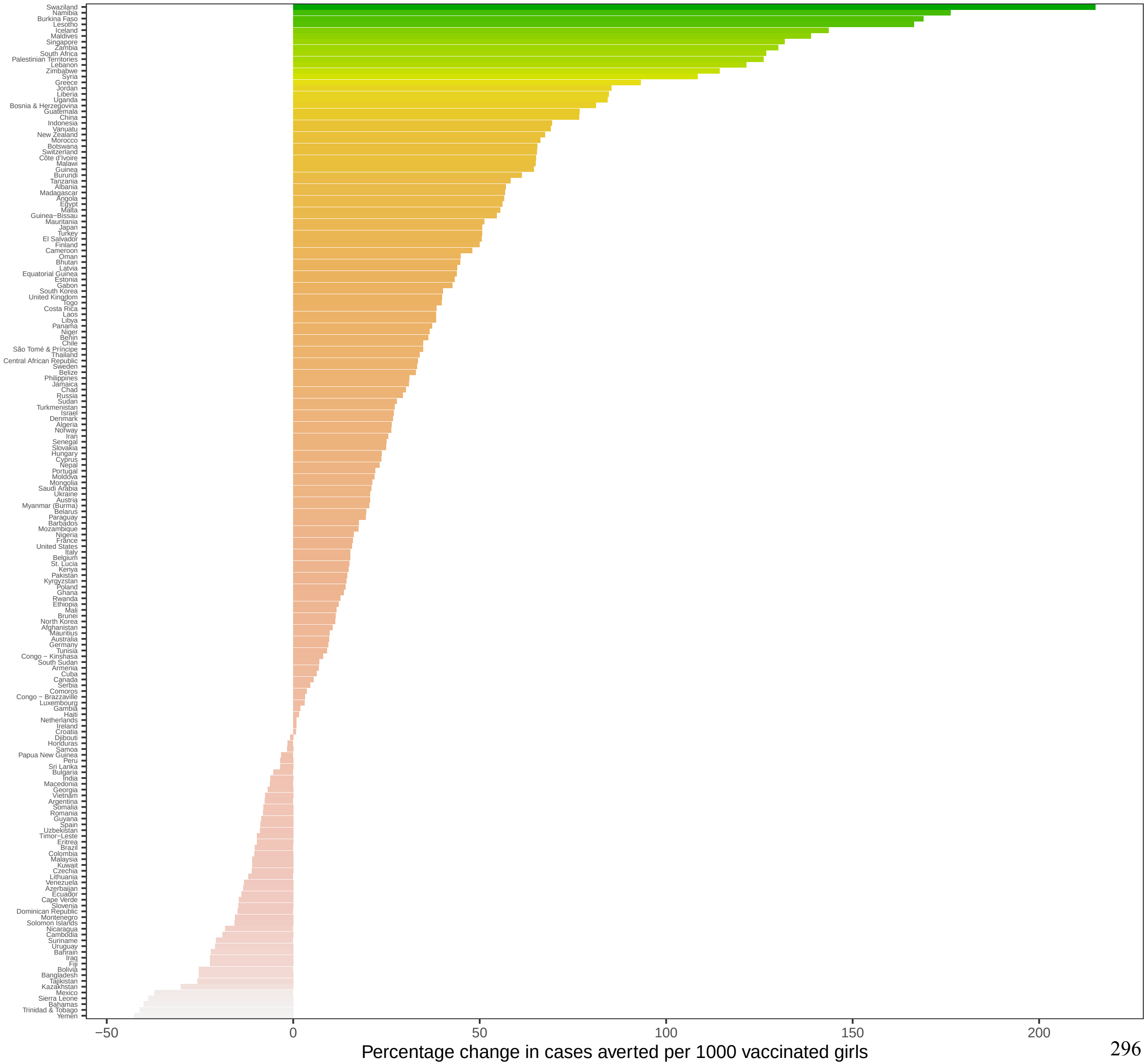

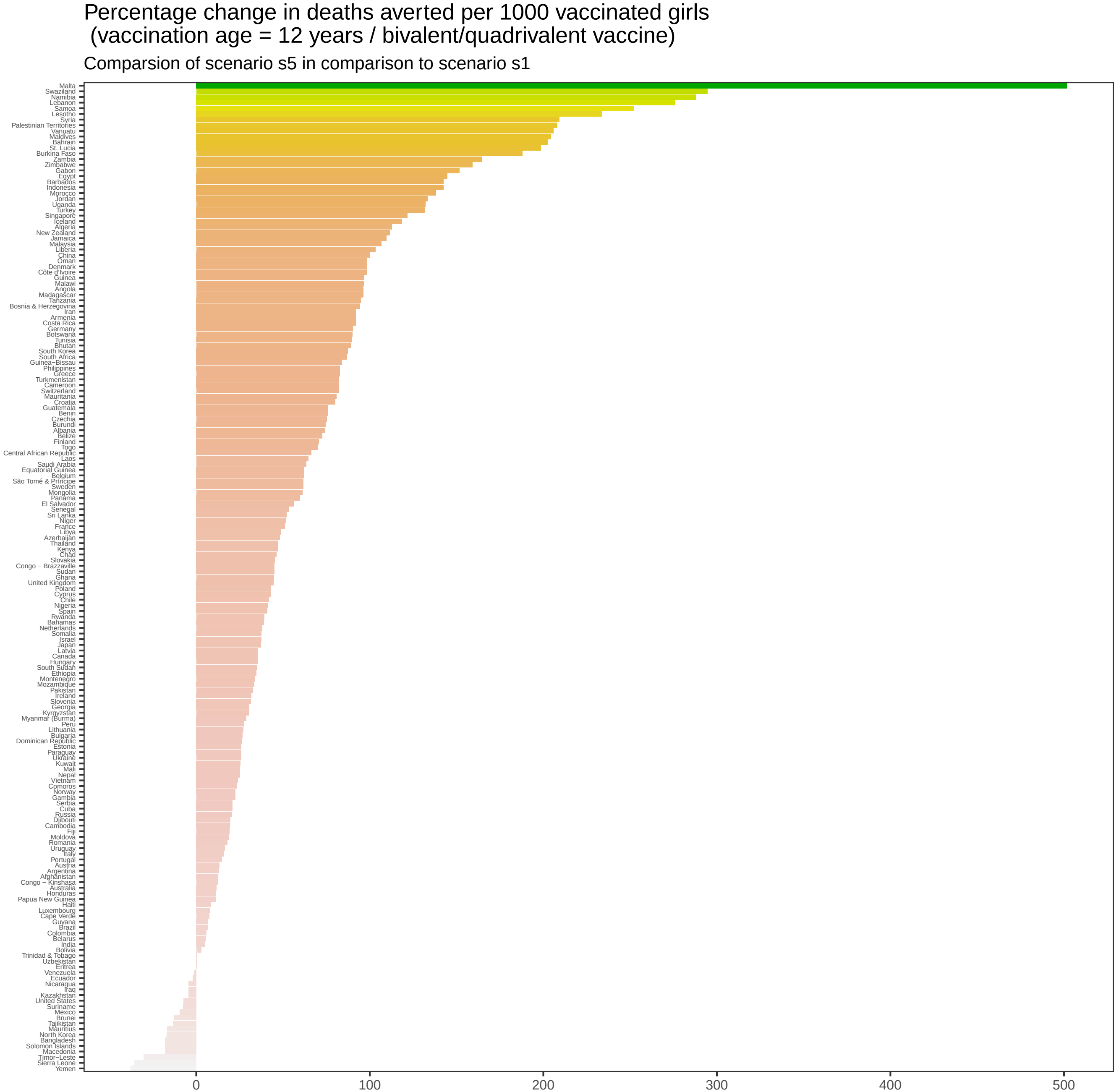

Percentage change in deaths averted per 1000 vaccinated girls
(vaccination age = 12 years / bivalent/quadrivalent vaccine)
Comparsion of scenario s5 in comparison to scenario s1
Malta
Swaziland
Namibia
Lebanon
Samoa
Lesotho
Syria
Palestinian Territories
Vanuatu
Maldives
Bahrain
St. Lucia
Burkina Faso
Zambia
Zimbabwe
Gabon
Egypt
Barbados
Indonesia
Morocco
Jordan
Uganda
Turkey
Singapore
Iceland
Algeria
New Zealand
Jamaica
Malaysia
Liberia
China
Oman
Denmark
Côte d'Ivoire
Guinea
Malawi
Angola
Madagascar
Tanzania
Bosnia & Herzegovina
Iran
Armenia
Costa Rica
Germany
Botswana
Tunisia
Bhutan
South Korea
South Africa
Guinea−Bissau
Philippines
Greece
Turkmenistan
Cameroon
Switzerland
Mauritania
Croatia
Guatemala
Benin
Czechia
Burundi
Albania
Belize
Finland
Togo
Central African Republic
Laos
Saudi Arabia
Equatorial Guinea
Belgium
São Tomé & Príncipe
Sweden
Mongolia
Panama
El Salvador
Senegal
Sri Lanka
Niger
France
Libya
Azerbaijan
Thailand
Kenya
Chad
Slovakia
Congo − Brazzaville
Sudan
Ghana
United Kingdom
Poland
Cyprus
Chile
Nigeria
Spain
Rwanda
Bahamas
Netherlands
Somalia
Israel
Japan
Latvia
Canada
Hungary
South Sudan
Ethiopia
Montenegro
Mozambique
Pakistan
Ireland
Slovenia
Georgia
Kyrgyzstan
Myanmar (Burma)
Peru
Lithuania
Bulgaria
Dominican Republic
Estonia
Paraguay
Ukraine
Kuwait
Mali
Nepal
Vietnam
Comoros
Norway
Gambia
Serbia
Cuba
Russia
Djibouti
Cambodia
Fiji
Moldova
Romania
Uruguay
Italy
Portugal
Austria
Argentina
Afghanistan
Congo − Kinshasa
Australia
Honduras
Papua New Guinea
Haiti
Luxembourg
Cape Verde
Guyana
Brazil
Colombia
Belarus
India
Bolivia
Trinidad & Tobago
Uzbekistan
Eritrea
Venezuela
Ecuador
Nicaragua
Iraq
Kazakhstan
United States
Suriname
Mexico
Brunei
Tajikistan
Mauritius
North Korea
Bangladesh
Solomon Islands
Macedonia
Timor−Leste
Sierra Leone
Yemen
0
100
200
300
400
500
Percentage change in deaths averted per 1000 vaccinated girls

Percentage change in YLDs averted per 1000 vaccinated girls
(vaccination age = 12 years / bivalent/quadrivalent vaccine)

Comparsion of scenario s5 in comparison to scenario s1

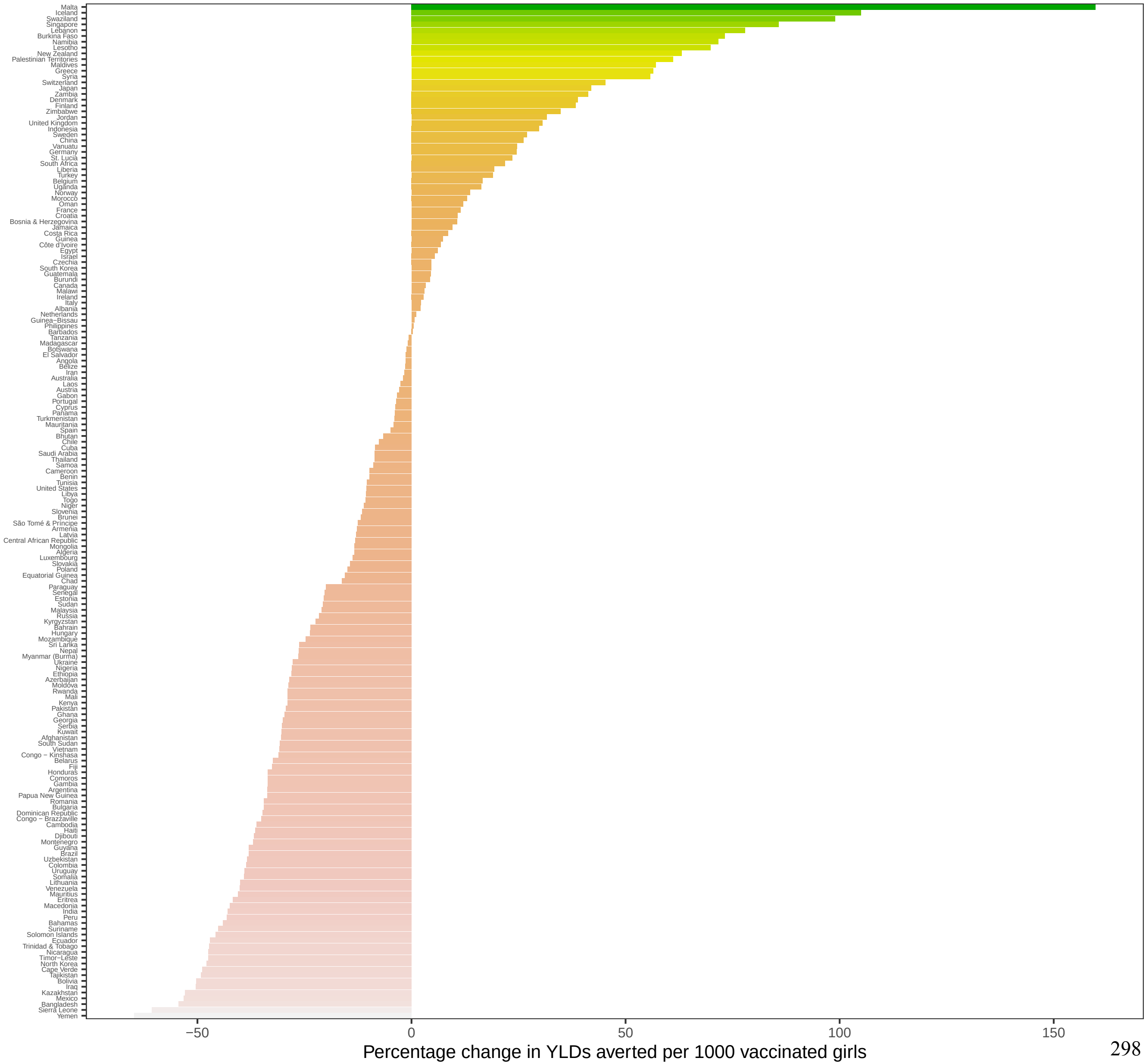

Percentage change in YLLs averted per 1000 vaccinated girls
(vaccination age = 12 years / bivalent/quadrivalent vaccine)

Comparsion of scenario s5 in comparison to scenario s1

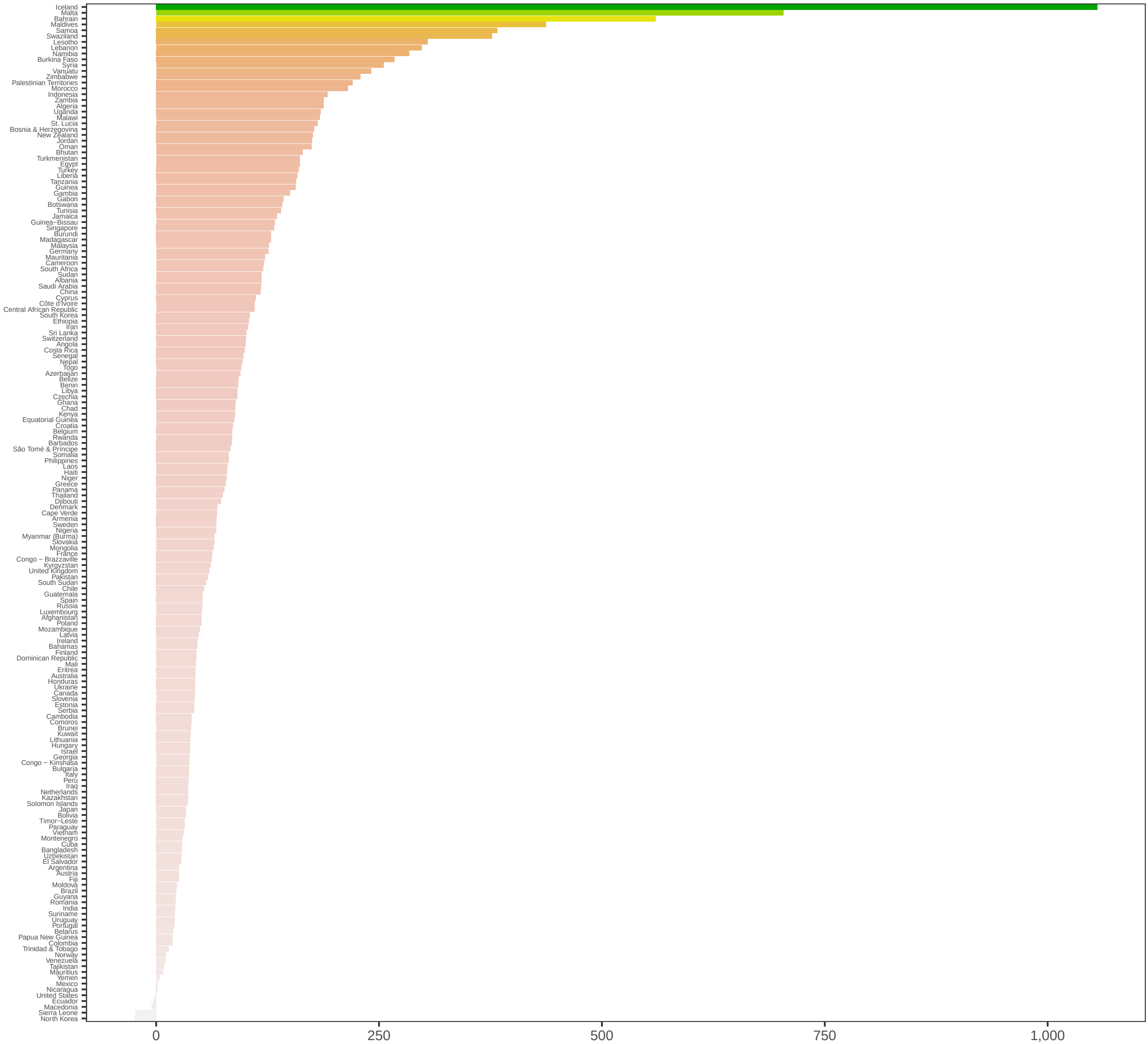

Percentage change in YLLs averted per 1000 vaccinated girls

Percentage change in DALYs averted per 1000 vaccinated girls
(vaccination age = 12 years / bivalent/quadrivalent vaccine)

Comparsion of scenario s5 in comparison to scenario s1

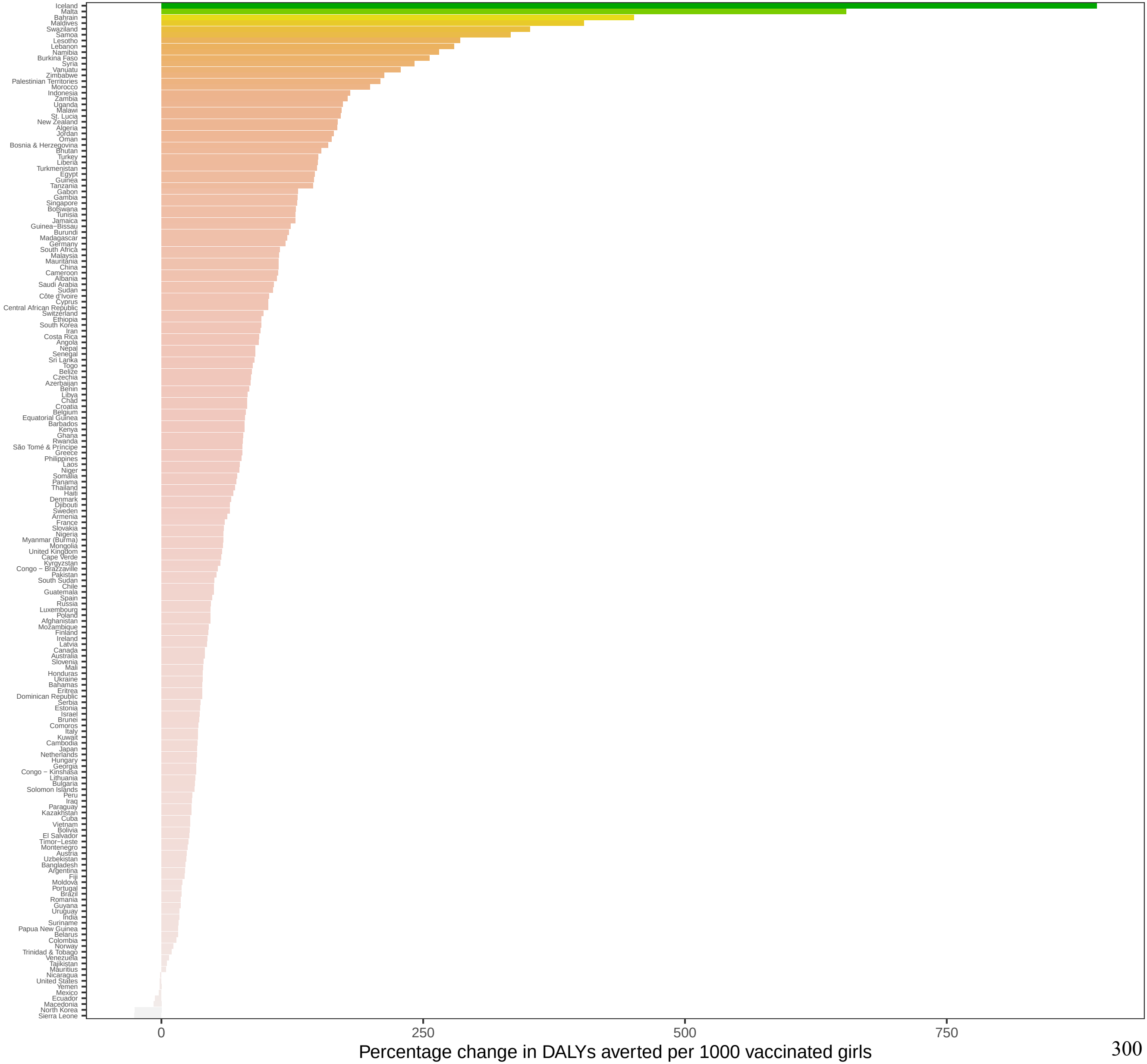

Percentage change in cases averted per 1000 vaccinated girls
(vaccination age = 12 years / nonavalent vaccine)

Comparsion of scenario s2 in comparison to scenario s1

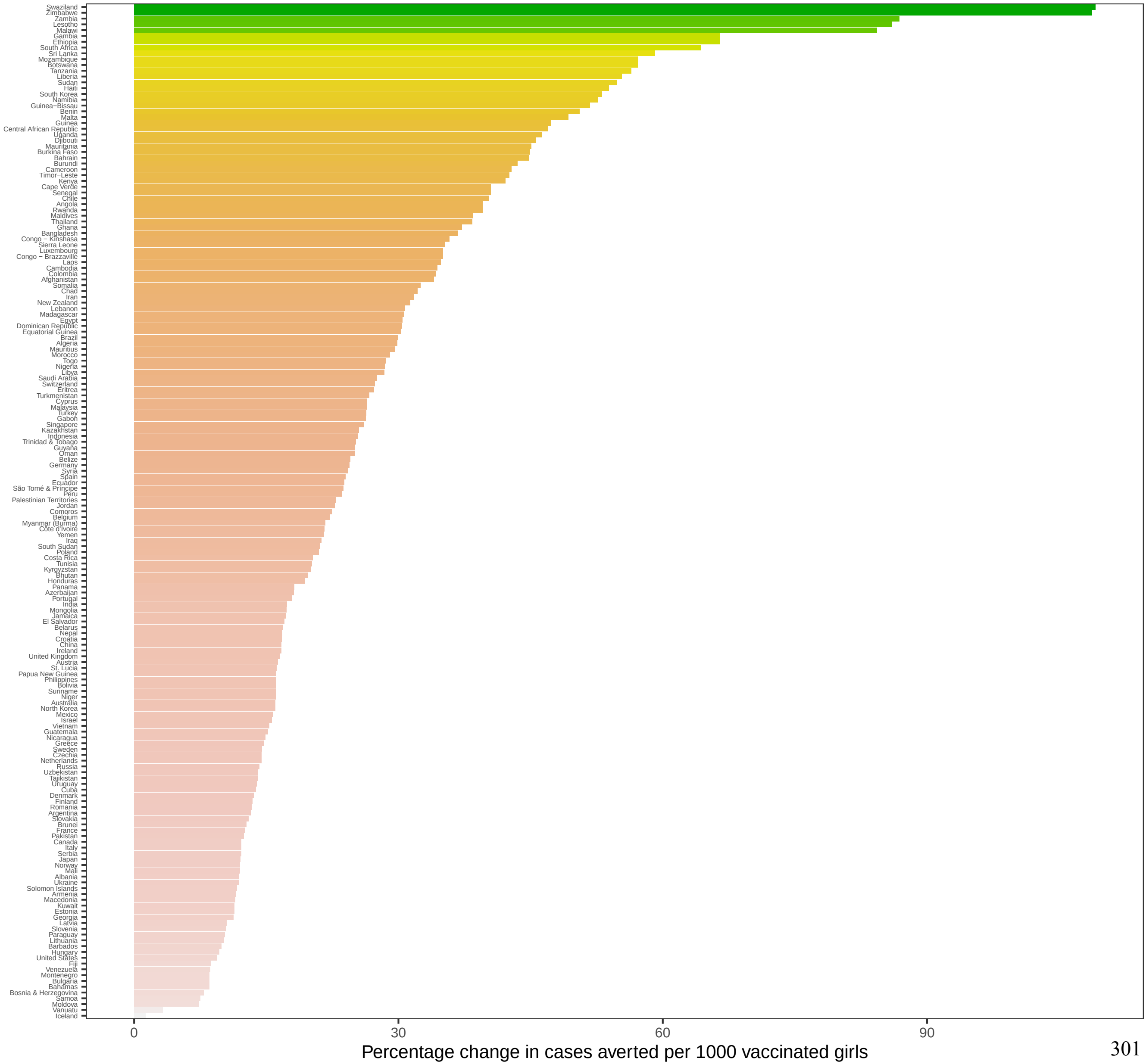

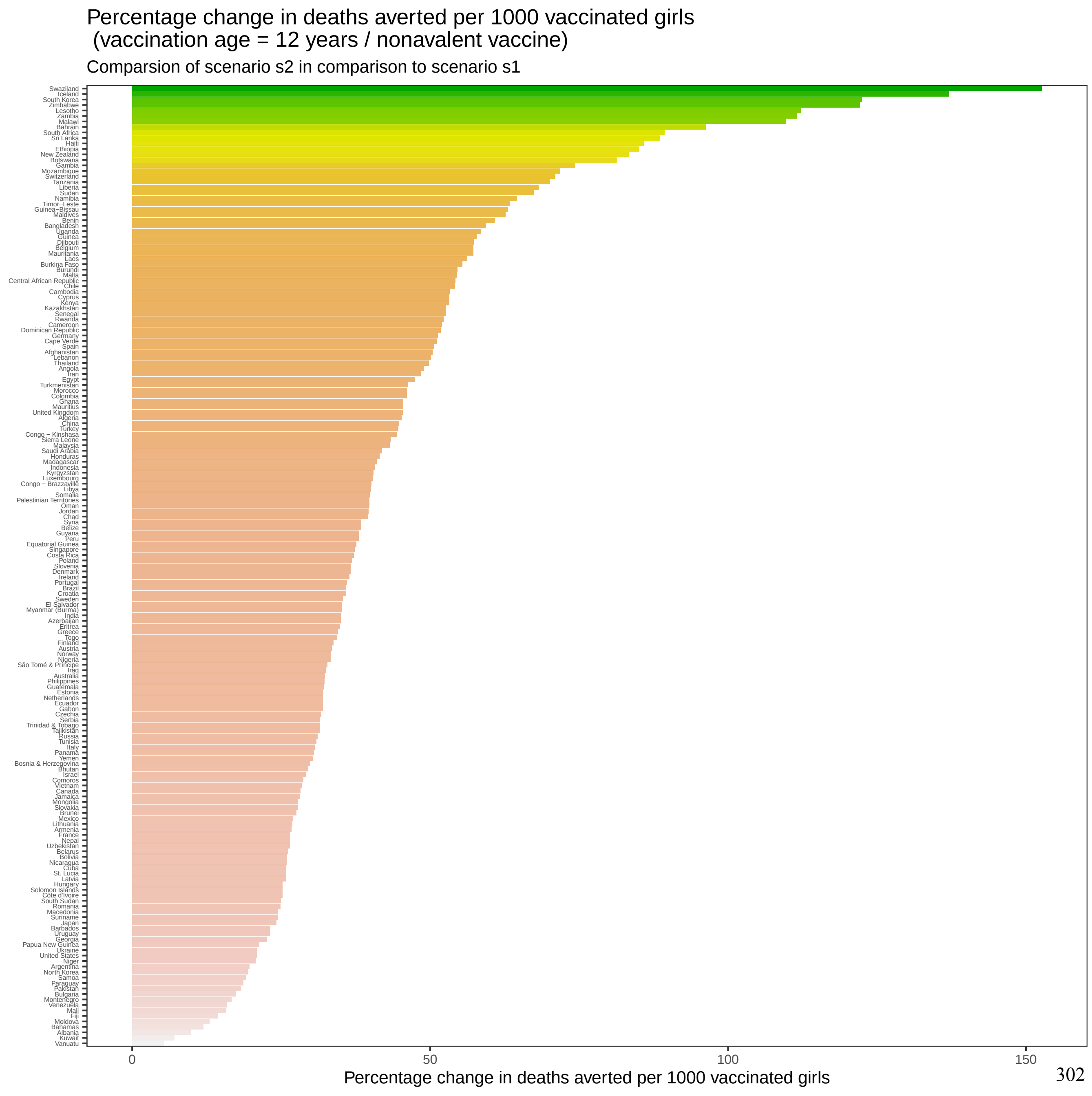
Percentage change in deaths averted per 1000 vaccinated girls
(vaccination age = 12 years / nonavalent vaccine)
Comparsion of scenario s2 in comparison to scenario s1
Swaziland
Iceland
South Korea
Zimbabwe
Lesotho
Zambia
Malawi
Bahrain
South Africa
Sri Lanka
Haiti
Ethiopia
New Zealand
Botswana
Gambia
Mozambique
Switzerland
Tanzania
Liberia
Sudan
Namibia
Timor−Leste
Guinea−Bissau
Maldives
Benin
Bangladesh
Uganda
Guinea
Djibouti
Belgium
Mauritania
Laos
Burkina Faso
Burundi
Malta
Central African Republic
Chile
Cambodia
Cyprus
Kenya
Kazakhstan
Senegal
Rwanda
Cameroon
Dominican Republic
Germany
Cape Verde
Spain
Afghanistan
Lebanon
Thailand
Angola
Iran
Egypt
Turkmenistan
Morocco
Colombia
Ghana
Mauritius
United Kingdom
Algeria
China
Turkey
Congo − Kinshasa
Sierra Leone
Malaysia
Saudi Arabia
Honduras
Madagascar
Indonesia
Kyrgyzstan
Luxembourg
Congo − Brazzaville
Libya
Somalia
Palestinian Territories
Oman
Jordan
Chad
Syria
Belize
Guyana
Peru
Equatorial Guinea
Singapore
Costa Rica
Poland
Slovenia
Denmark
Ireland
Portugal
Brazil
Croatia
Sweden
El Salvador
Myanmar (Burma)
India
Azerbaijan
Eritrea
Greece
Togo
Finland
Austria
Norway
Nigeria
São Tomé & Príncipe
Iraq
Australia
Philippines
Guatemala
Estonia
Netherlands
Ecuador
Gabon
Czechia
Serbia
Trinidad & Tobago
Tajikistan
Russia
Tunisia
Italy
Panama
Yemen
Bosnia & Herzegovina
Bhutan
Israel
Comoros
Vietnam
Canada
Jamaica
Mongolia
Slovakia
Brunei
Mexico
Lithuania
Armenia
France
Nepal
Uzbekistan
Belarus
Bolivia
Nicaragua
Cuba
St. Lucia
Latvia
Hungary
Solomon Islands
Côte d'Ivoire
South Sudan
Romania
Macedonia
Suriname
Japan
Barbados
Uruguay
Georgia
Papua New Guinea
Ukraine
United States
Niger
Argentina
North Korea
Samoa
Paraguay
Pakistan
Bulgaria
Montenegro
Venezuela
Mali
Fiji
Moldova
Bahamas
Albania
Kuwait
Vanuatu
0
50
100
150
Percentage change in deaths averted per 1000 vaccinated girls

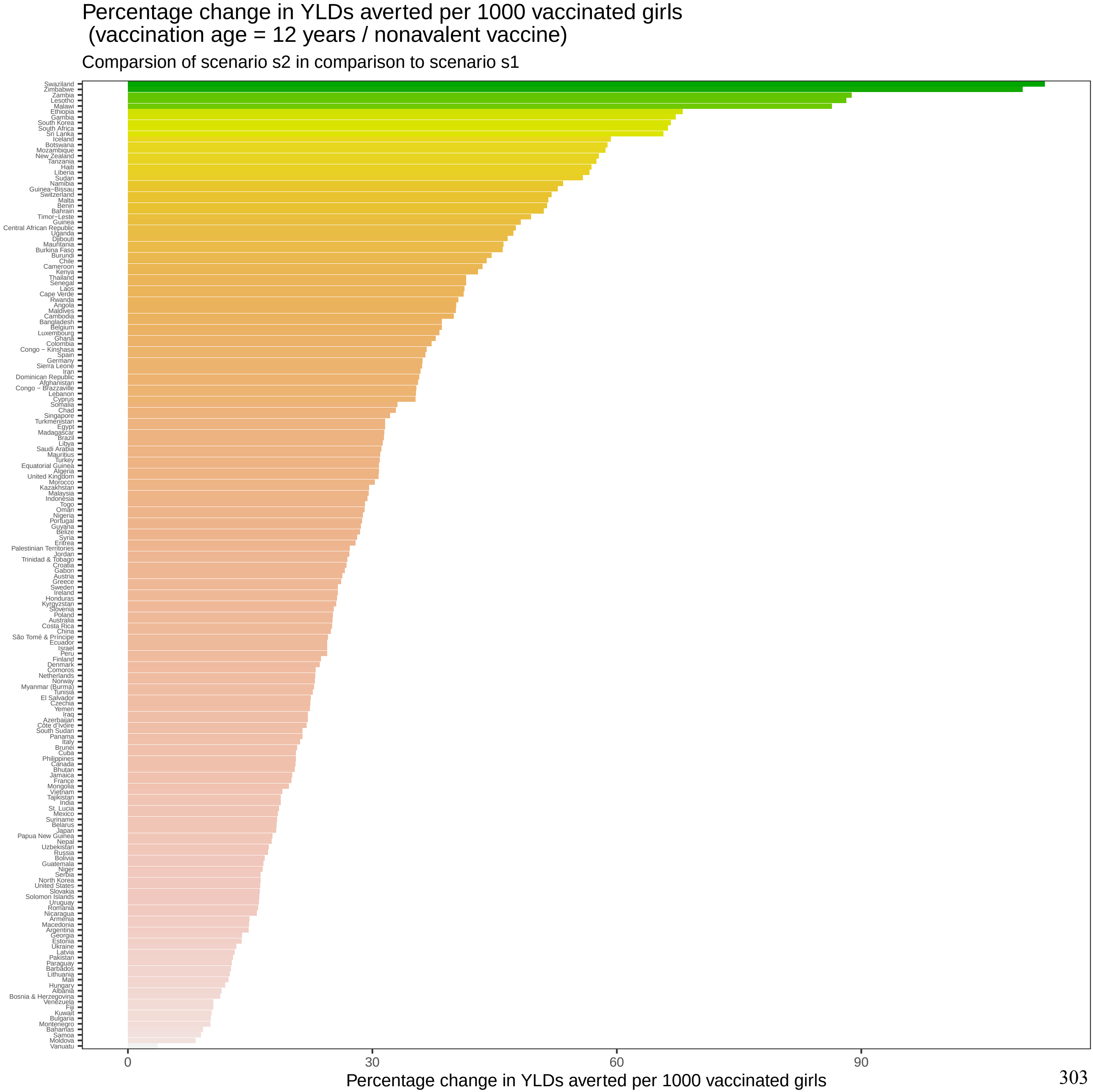
Percentage change in YLDs averted per 1000 vaccinated girls
(vaccination age = 12 years / nonavalent vaccine)
Comparsion of scenario s2 in comparison to scenario s1
Swaziland
Zimbabwe
Zambia
Lesotho
Malawi
Ethiopia
Gambia
South Korea
South Africa
Sri Lanka
Iceland
Botswana
Mozambique
New Zealand
Tanzania
Haiti
Liberia
Sudan
Namibia
Guinea−Bissau
Switzerland
Malta
Benin
Bahrain
Timor−Leste
Guinea
Central African Republic
Uganda
Djibouti
Mauritania
Burkina Faso
Burundi
Chile
Cameroon
Kenya
Thailand
Senegal
Laos
Cape Verde
Rwanda
Angola
Maldives
Cambodia
Bangladesh
Belgium
Luxembourg
Ghana
Colombia
Congo − Kinshasa
Spain
Germany
Sierra Leone
Iran
Dominican Republic
Afghanistan
Congo − Brazzaville
Lebanon
Cyprus
Somalia
Chad
Singapore
Turkmenistan
Egypt
Madagascar
Brazil
Libya
Saudi Arabia
Mauritius
Turkey
Equatorial Guinea
Algeria
United Kingdom
Morocco
Kazakhstan
Malaysia
Indonesia
Togo
Oman
Nigeria
Portugal
Guyana
Belize
Syria
Eritrea
Palestinian Territories
Jordan
Trinidad & Tobago
Croatia
Gabon
Austria
Greece
Sweden
Ireland
Honduras
Kyrgyzstan
Slovenia
Poland
Australia
Costa Rica
China
São Tomé & Príncipe
Ecuador
Israel
Peru
Finland
Denmark
Comoros
Netherlands
Norway
Myanmar (Burma)
Tunisia
El Salvador
Czechia
Yemen
Iraq
Azerbaijan
Côte d'Ivoire
South Sudan
Panama
Italy
Brunei
Cuba
Philippines
Canada
Bhutan
Jamaica
France
Mongolia
Vietnam
Tajikistan
India
St. Lucia
Mexico
Suriname
Belarus
Japan
Papua New Guinea
Nepal
Uzbekistan
Russia
Bolivia
Guatemala
Niger
Serbia
North Korea
United States
Slovakia
Solomon Islands
Uruguay
Romania
Nicaragua
Armenia
Macedonia
Argentina
Georgia
Estonia
Ukraine
Latvia
Pakistan
Paraguay
Barbados
Lithuania
Mali
Hungary
Albania
Bosnia & Herzegovina
Venezuela
Fiji
Kuwait
Bulgaria
Montenegro
Bahamas
Samoa
Moldova
Vanuatu
0
30
60
90
Percentage change in YLDs averted per 1000 vaccinated girls

Percentage change in YLLs averted per 1000 vaccinated girls
(vaccination age = 12 years / nonavalent vaccine)

Comparsion of scenario s2 in comparison to scenario s1

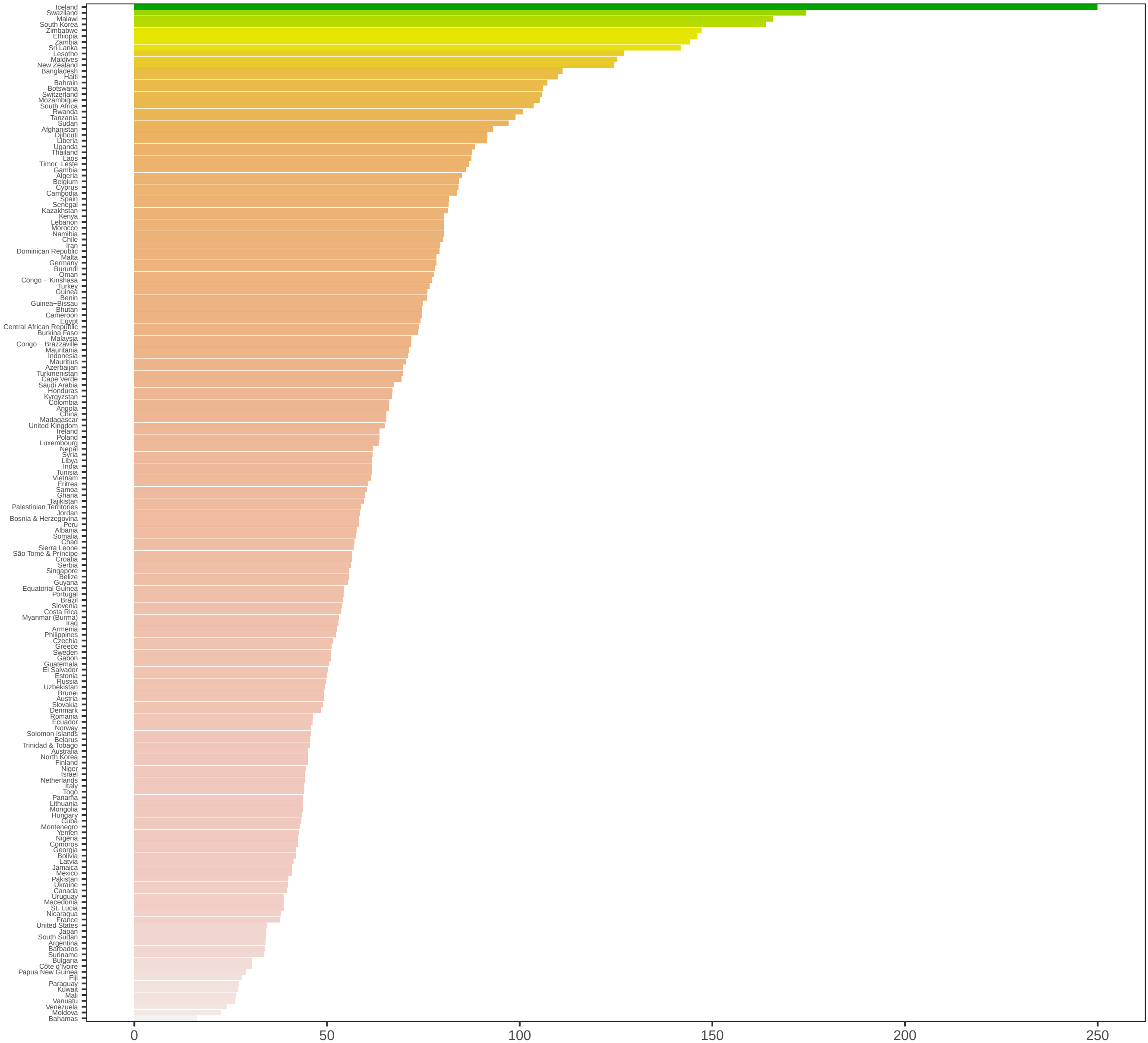

Percentage change in DALYs averted per 1000 vaccinated girls
(vaccination age = 12 years / nonavalent vaccine)

Comparsion of scenario s2 in comparison to scenario s1

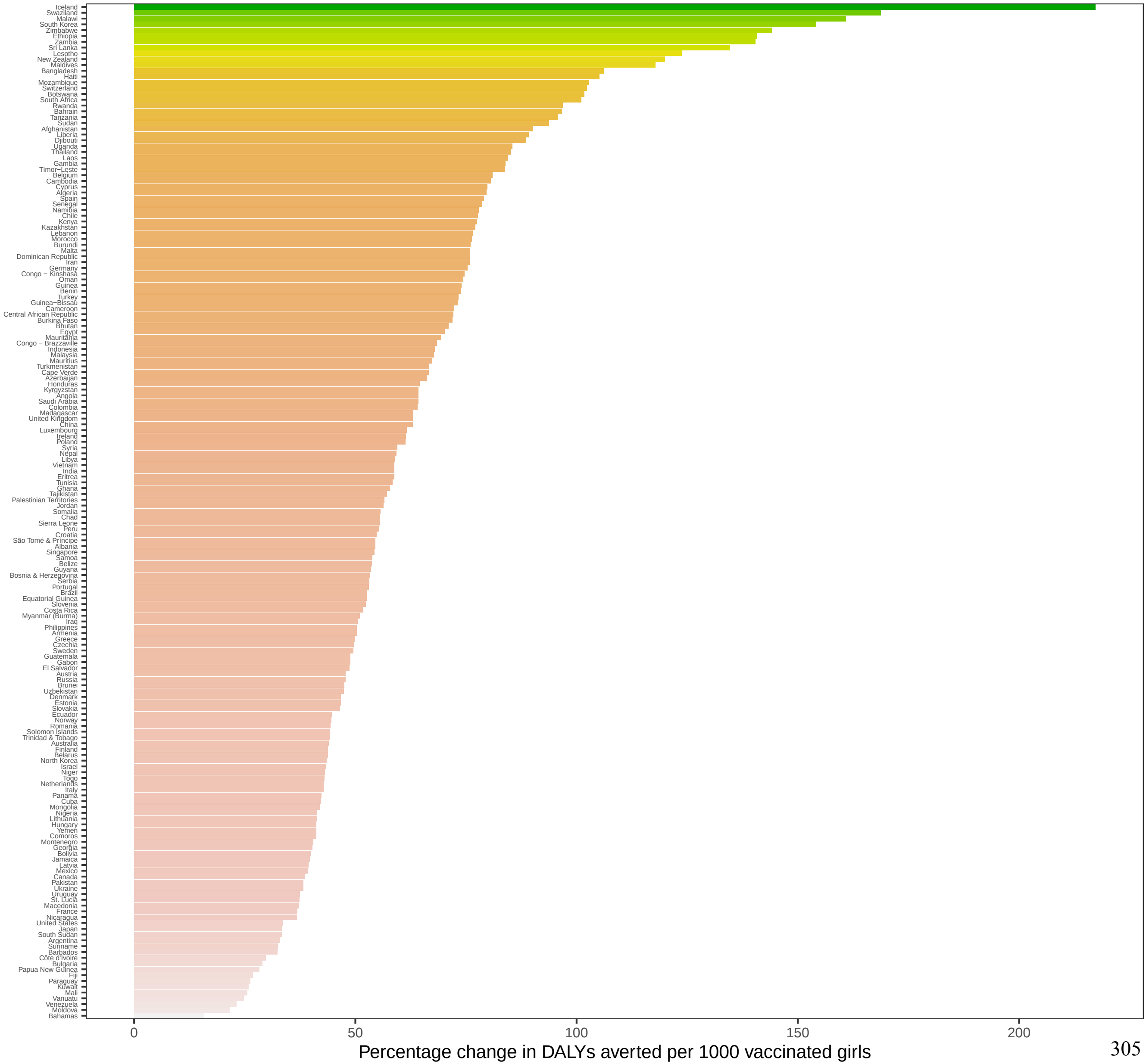

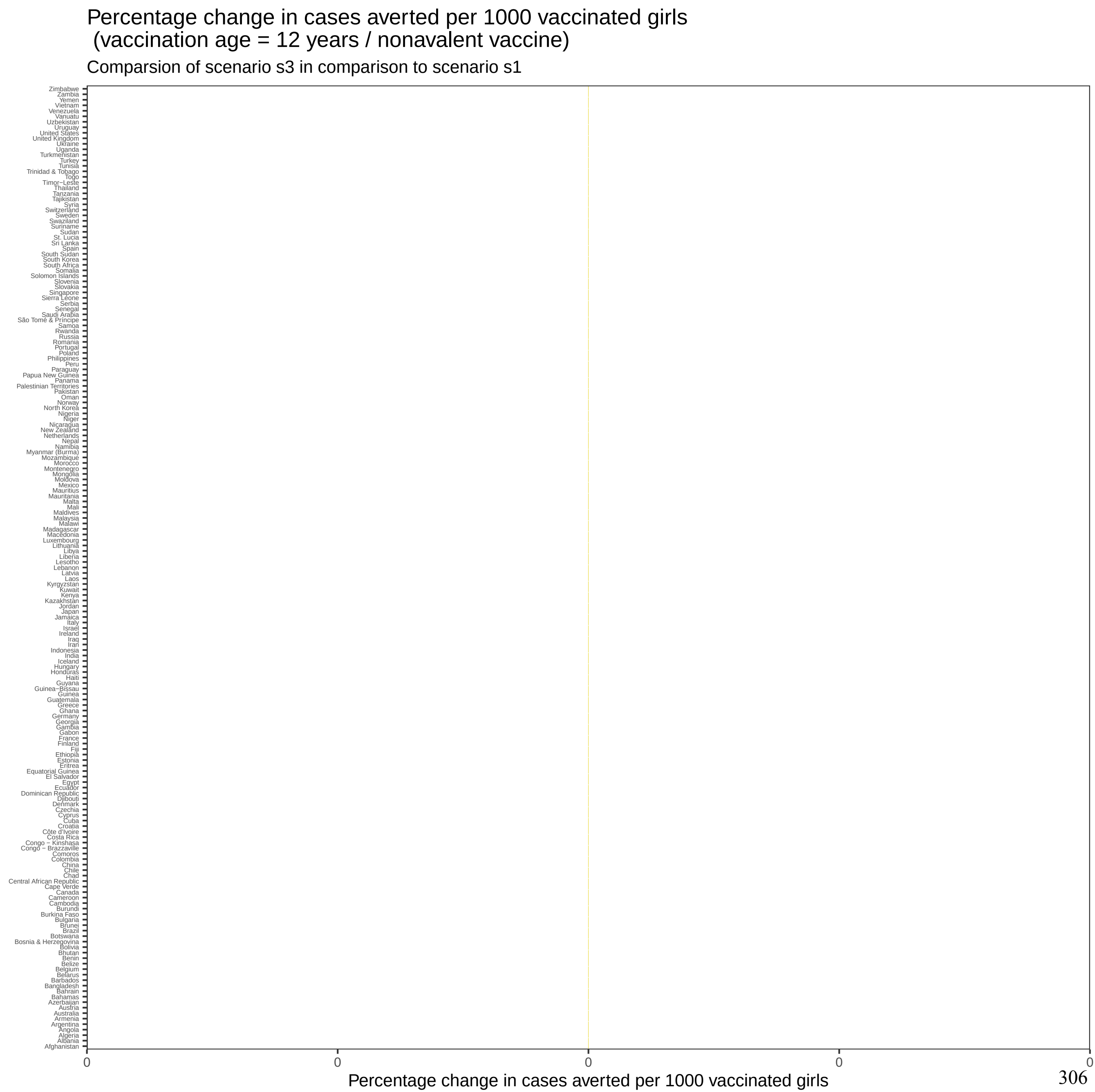
Percentage change in cases averted per 1000 vaccinated girls
(vaccination age = 12 years / nonavalent vaccine)
Comparsion of scenario s3 in comparison to scenario s1
Percentage change in cases averted per 1000 vaccinated girls

Percentage change in deaths averted per 1000 vaccinated girls (vaccination age = 12 years / nonavalent vaccine)

Comparsion of scenario s3 in comparison to scenario s1

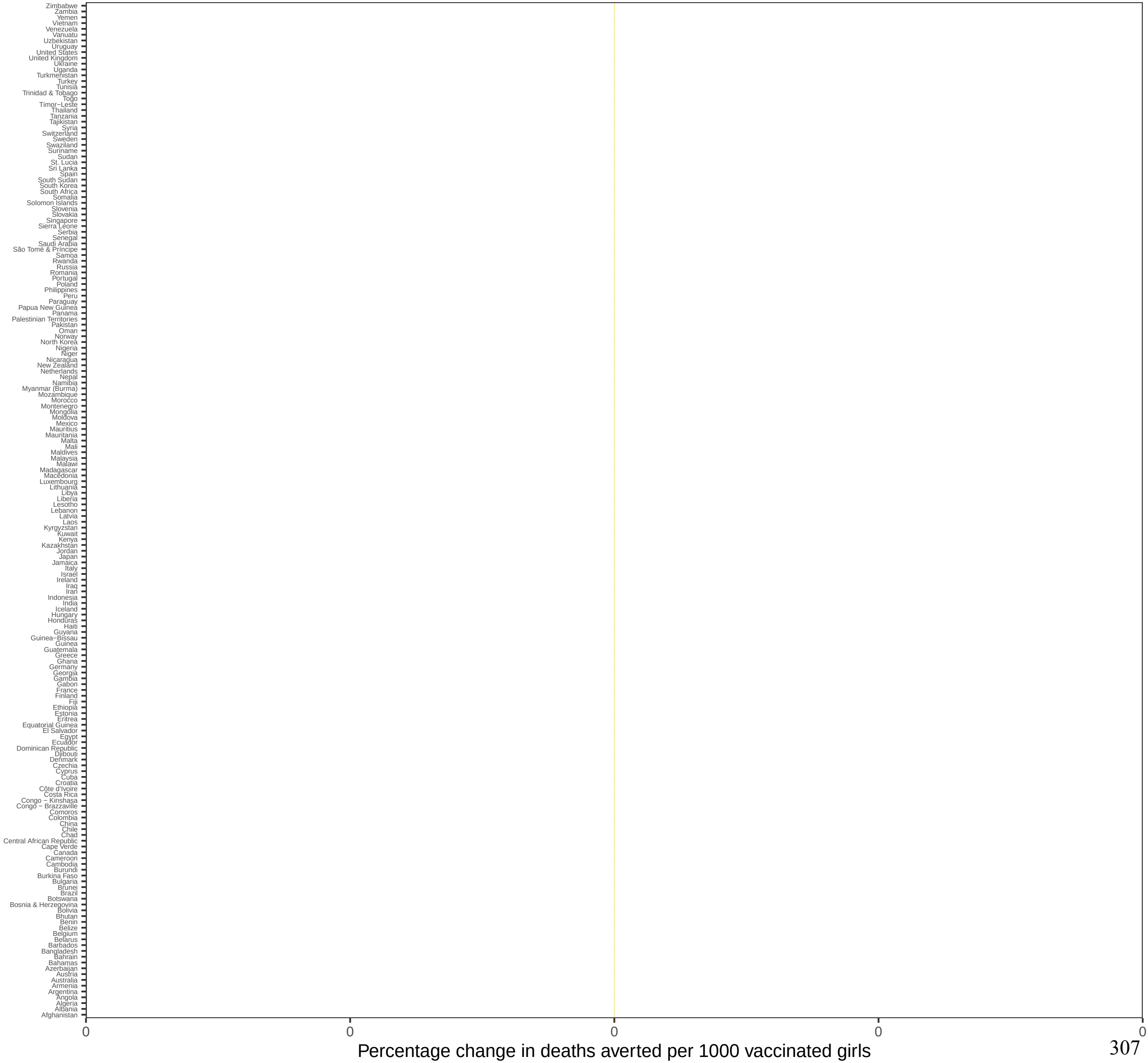

Percentage change in YLDs averted per 1000 vaccinated girls
(vaccination age = 12 years / nonavalent vaccine)

Comparsion of scenario s3 in comparison to scenario s1

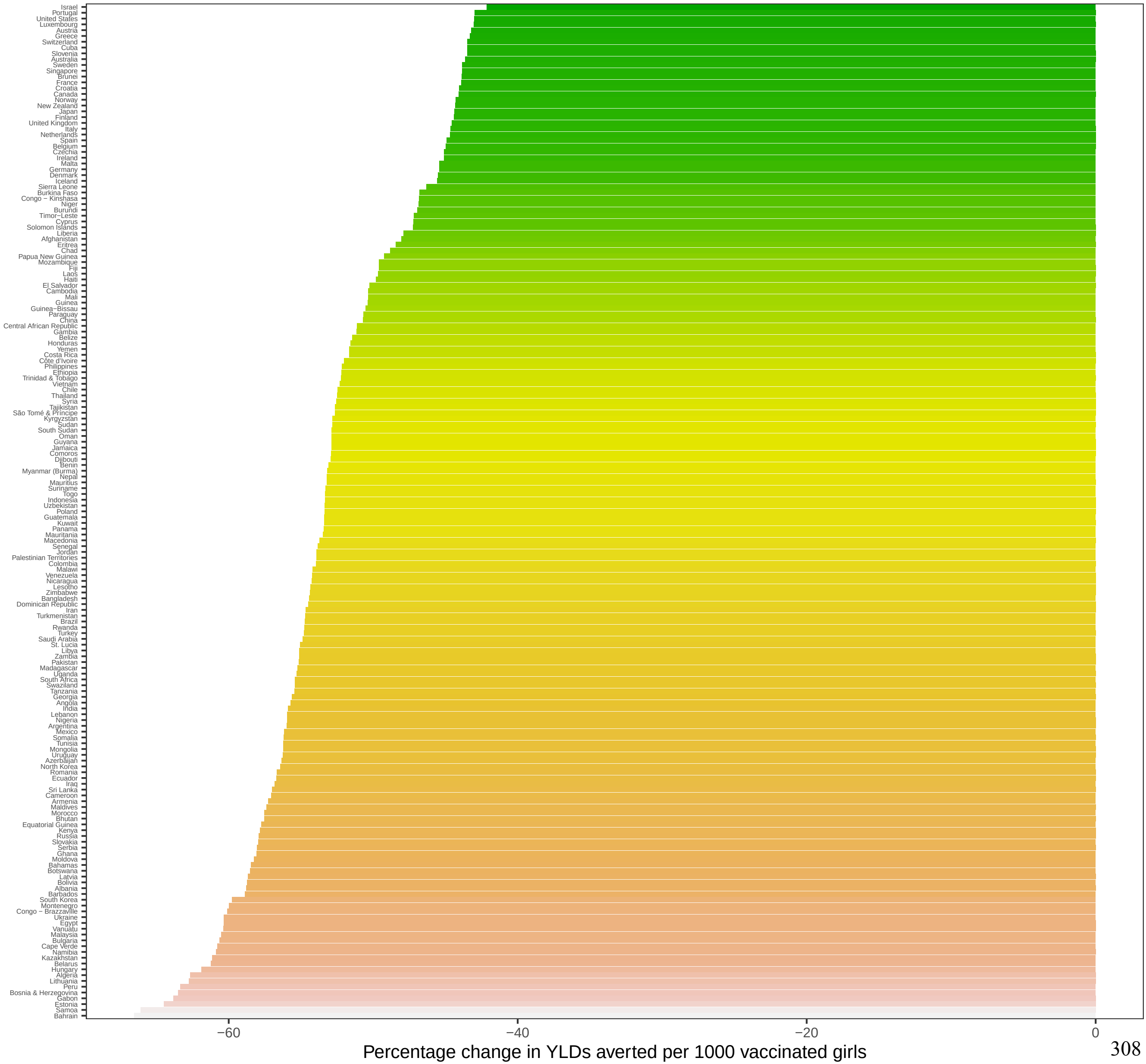

Percentage change in YLLs averted per 1000 vaccinated girls
(vaccination age = 12 years / nonavalent vaccine)

Comparsion of scenario s3 in comparison to scenario s1

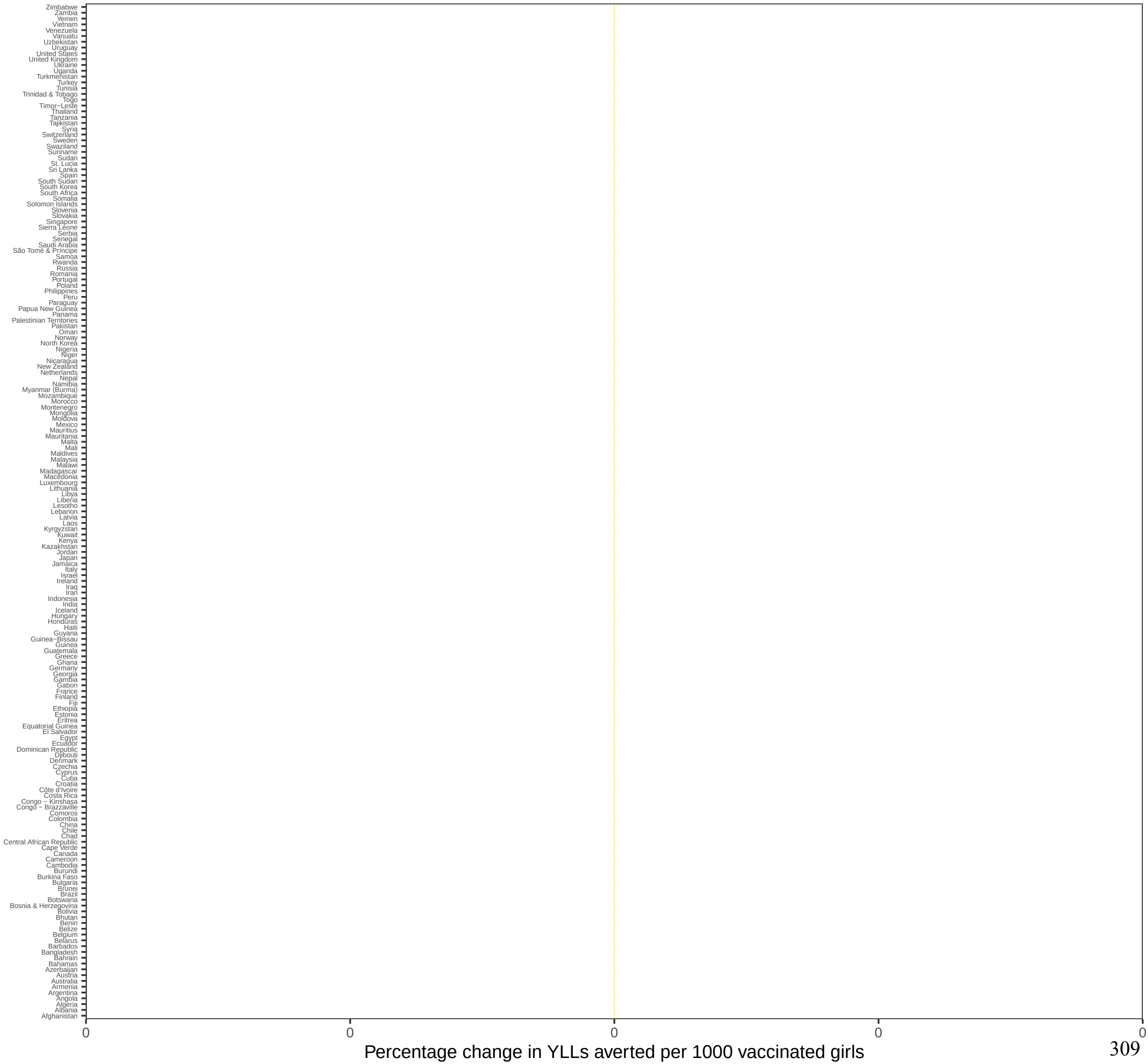

Percentage change in DALYs averted per 1000 vaccinated girls
(vaccination age = 12 years / nonavalent vaccine)

Comparsion of scenario s3 in comparison to scenario s1

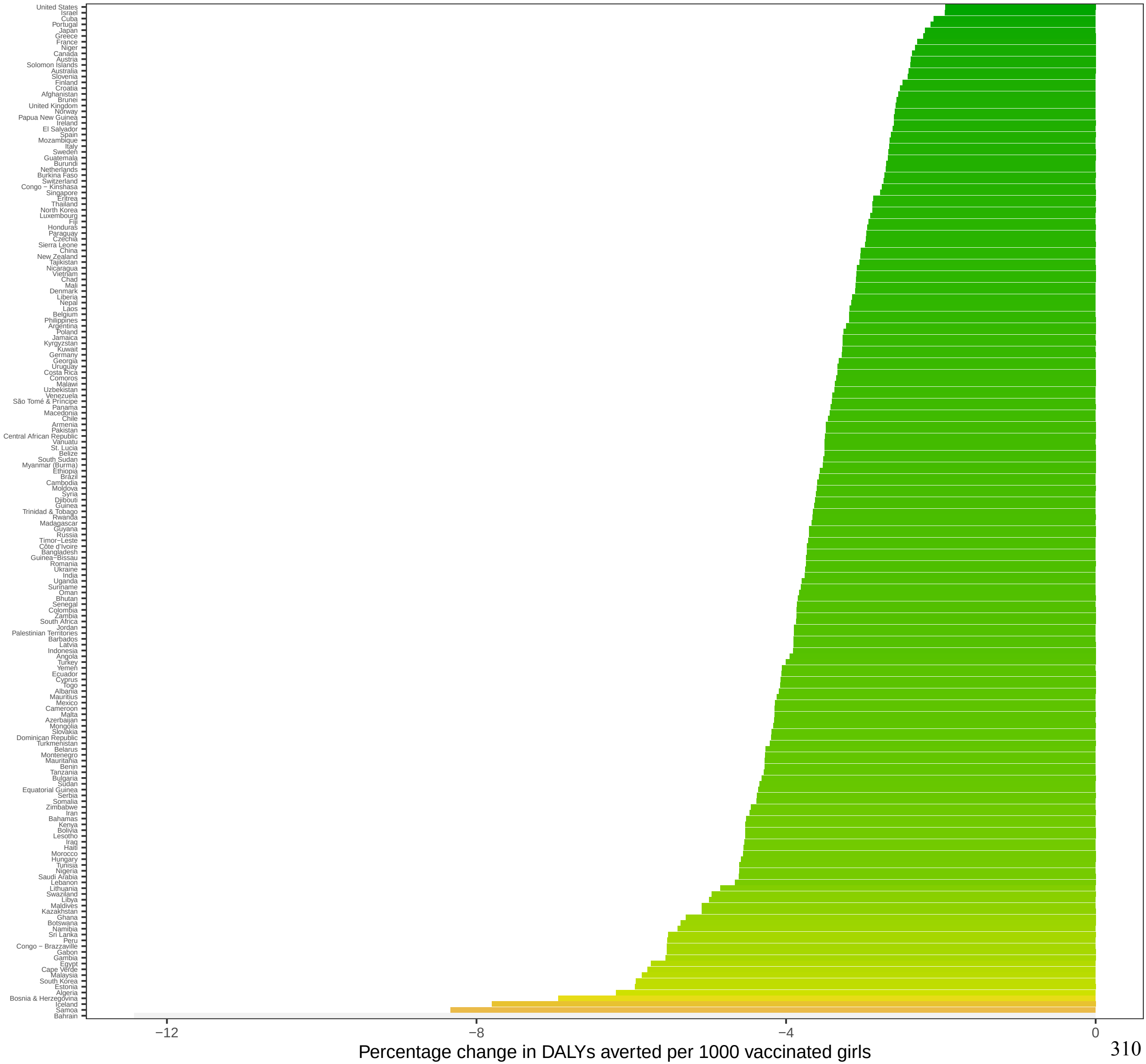

Percentage change in cases averted per 1000 vaccinated girls
(vaccination age = 12 years / nonavalent vaccine)

Comparsion of scenario s4 in comparison to scenario s1

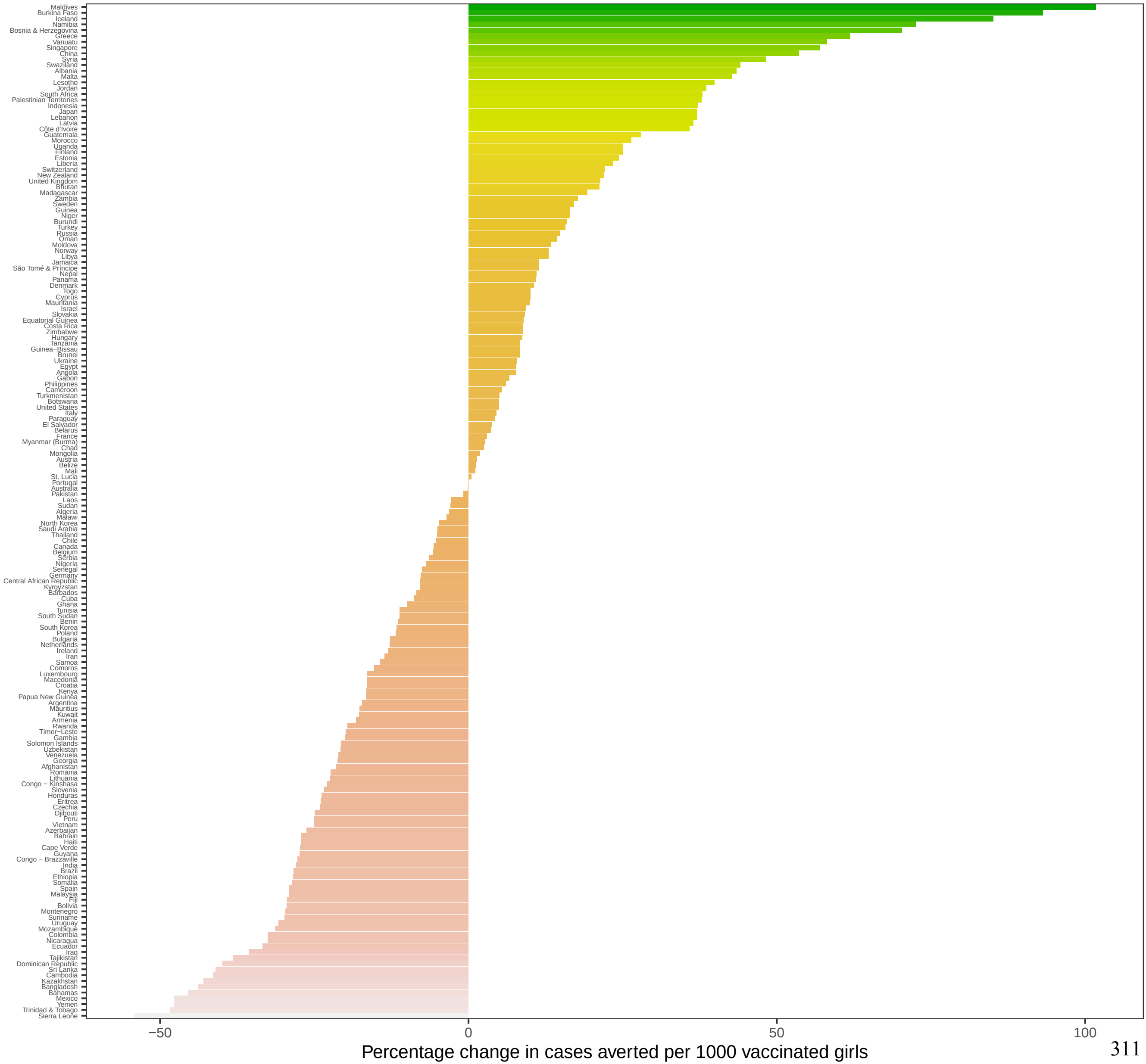

Percentage change in deaths averted per 1000 vaccinated girls (vaccination age = 12 years / nonavalent vaccine)

Comparsion of scenario s4 in comparison to scenario s1

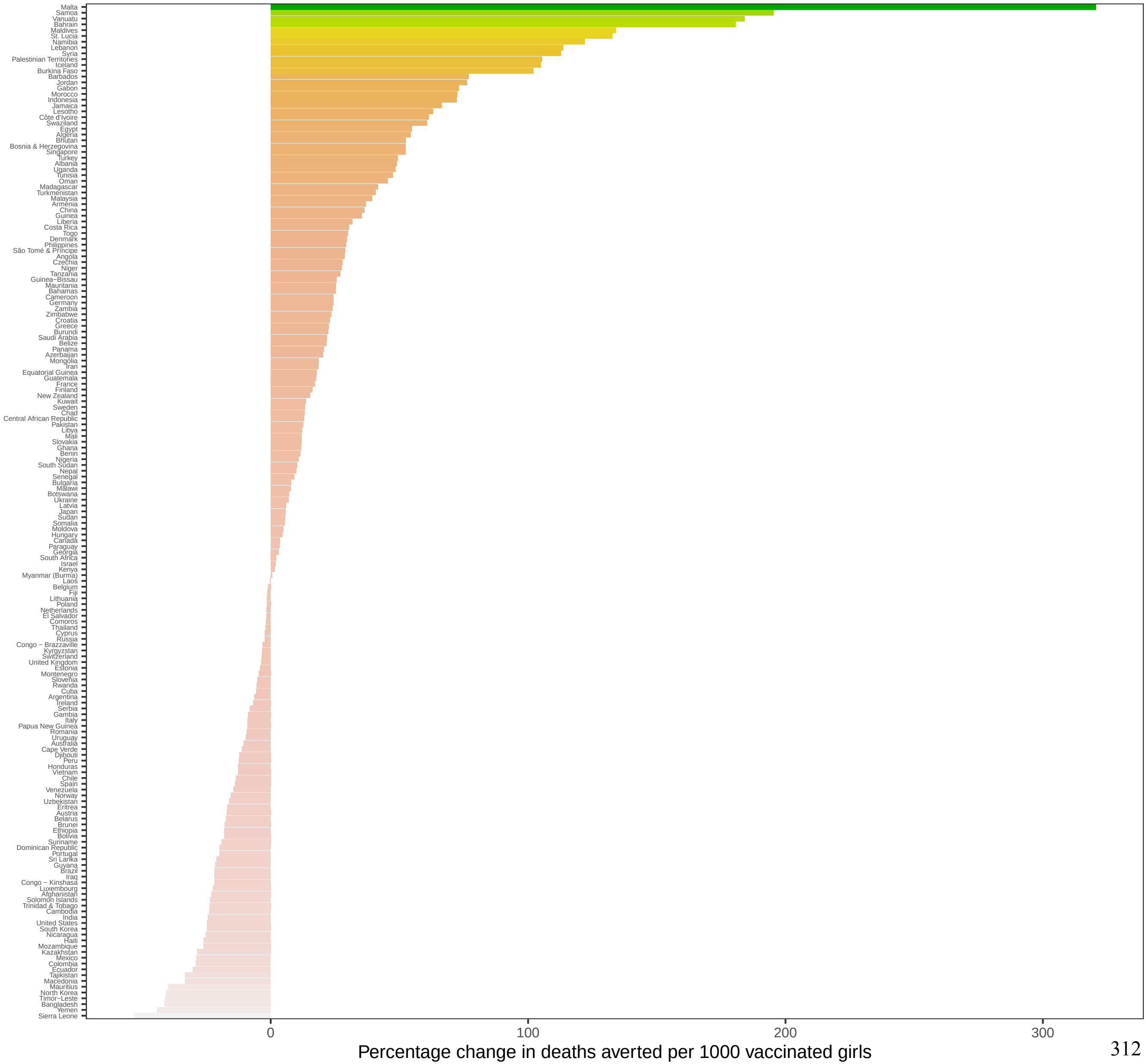

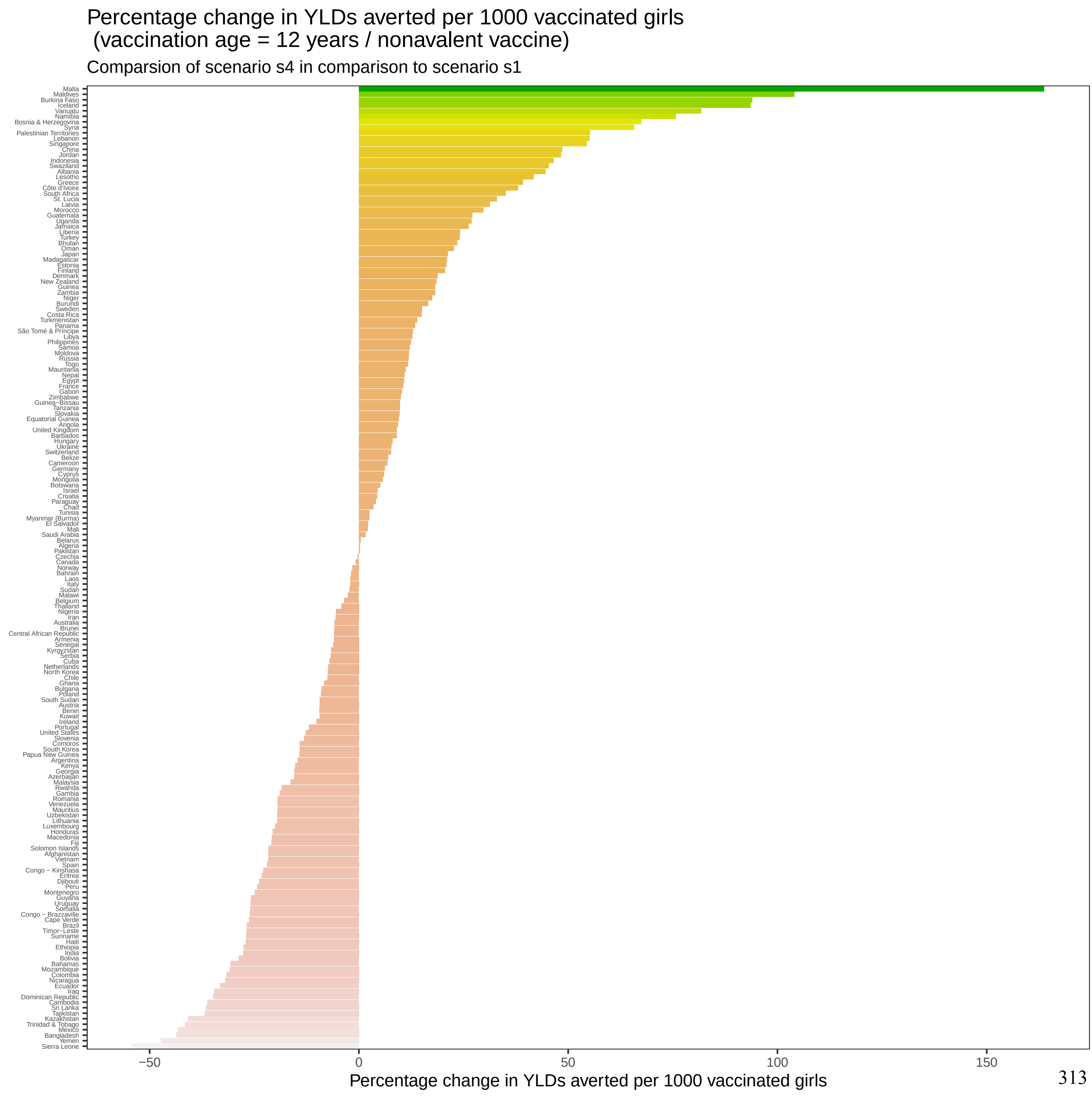
Percentage change in YLDs averted per 1000 vaccinated girls
(vaccination age = 12 years / nonavalent vaccine)
Comparsion of scenario s4 in comparison to scenario s1
Malta
Maldives
Burkina Faso
Iceland
Vanuatu
Namibia
Bosnia & Herzegovina
Syria
Palestinian Territories
Lebanon
Singapore
China
Jordan
Indonesia
Swaziland
Albania
Lesotho
Greece
Côte d'Ivoire
South Africa
St. Lucia
Latvia
Morocco
Guatemala
Uganda
Jamaica
Liberia
Turkey
Bhutan
Oman
Japan
Madagascar
Estonia
Finland
Denmark
New Zealand
Guinea
Zambia
Niger
Burundi
Sweden
Costa Rica
Turkmenistan
Panama
São Tomé & Príncipe
Libya
Philippines
Samoa
Moldova
Russia
Togo
Mauritania
Nepal
Egypt
France
Gabon
Zimbabwe
Guinea−Bissau
Tanzania
Slovakia
Equatorial Guinea
Angola
United Kingdom
Barbados
Hungary
Ukraine
Switzerland
Belize
Cameroon
Germany
Cyprus
Mongolia
Botswana
Israel
Croatia
Paraguay
Chad
Tunisia
Myanmar (Burma)
El Salvador
Mali
Saudi Arabia
Belarus
Algeria
Pakistan
Czechia
Canada
Norway
Bahrain
Laos
Italy
Sudan
Malawi
Belgium
Thailand
Nigeria
Iran
Australia
Brunei
Central African Republic
Armenia
Senegal
Kyrgyzstan
Serbia
Cuba
Netherlands
North Korea
Chile
Ghana
Bulgaria
Poland
South Sudan
Austria
Benin
Kuwait
Ireland
Portugal
United States
Slovenia
Comoros
South Korea
Papua New Guinea
Argentina
Kenya
Georgia
Azerbaijan
Malaysia
Rwanda
Gambia
Romania
Venezuela
Mauritius
Uzbekistan
Lithuania
Luxembourg
Honduras
Macedonia
Fiji
Solomon Islands
Afghanistan
Vietnam
Spain
Congo − Kinshasa
Eritrea
Djibouti
Peru
Montenegro
Guyana
Uruguay
Somalia
Congo − Brazzaville
Cape Verde
Brazil
Timor−Leste
Suriname
Haiti
Ethiopia
India
Bolivia
Bahamas
Mozambique
Colombia
Nicaragua
Ecuador
Iraq
Dominican Republic
Cambodia
Sri Lanka
Tajikistan
Kazakhstan
Trinidad & Tobago
Mexico
Bangladesh
Yemen
Sierra Leone
−50
0
50
100
150
Percentage change in YLDs averted per 1000 vaccinated girls

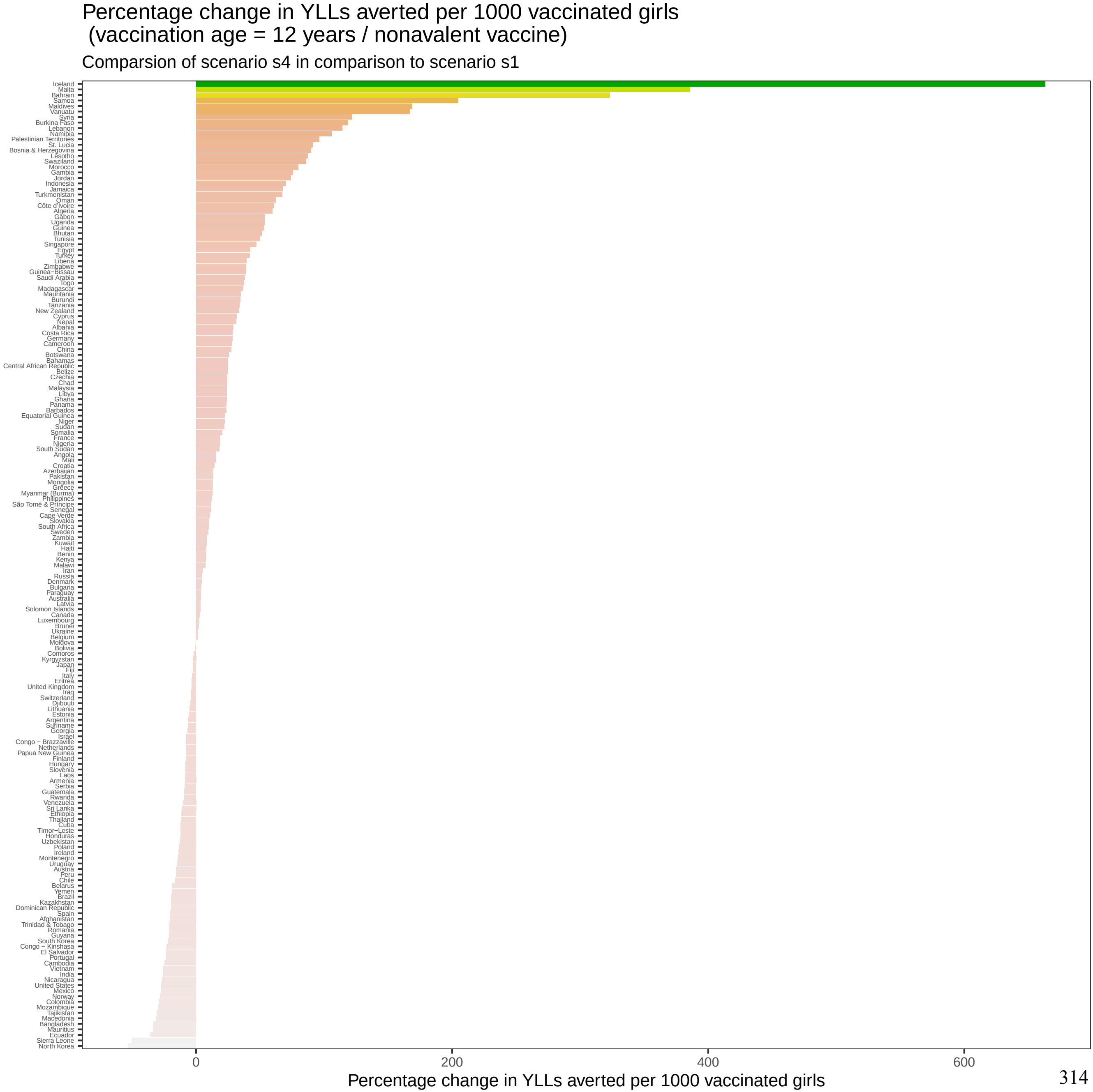
Percentage change in YLLs averted per 1000 vaccinated girls
(vaccination age = 12 years / nonavalent vaccine)
Comparsion of scenario s4 in comparison to scenario s1
Iceland
Malta
Bahrain
Samoa
Maldives
Vanuatu
Syria
Burkina Faso
Lebanon
Namibia
Palestinian Territories
St. Lucia
Bosnia & Herzegovina
Lesotho
Swaziland
Morocco
Gambia
Jordan
Indonesia
Jamaica
Turkmenistan
Oman
Côte d'Ivoire
Algeria
Gabon
Uganda
Guinea
Bhutan
Tunisia
Singapore
Egypt
Turkey
Liberia
Zimbabwe
Guinea−Bissau
Saudi Arabia
Togo
Madagascar
Mauritania
Burundi
Tanzania
New Zealand
Cyprus
Nepal
Albania
Costa Rica
Germany
Cameroon
China
Botswana
Bahamas
Central African Republic
Belize
Czechia
Chad
Malaysia
Libya
Ghana
Panama
Barbados
Equatorial Guinea
Niger
Sudan
Somalia
France
Nigeria
South Sudan
Angola
Mali
Croatia
Azerbaijan
Pakistan
Mongolia
Greece
Myanmar (Burma)
Philippines
São Tomé & Príncipe
Senegal
Cape Verde
Slovakia
South Africa
Sweden
Zambia
Kuwait
Haiti
Benin
Kenya
Malawi
Iran
Russia
Denmark
Bulgaria
Paraguay
Australia
Latvia
Solomon Islands
Canada
Luxembourg
Brunei
Ukraine
Belgium
Moldova
Bolivia
Comoros
Kyrgyzstan
Japan
Fiji
Italy
Eritrea
United Kingdom
Iraq
Switzerland
Djibouti
Lithuania
Estonia
Argentina
Suriname
Georgia
Israel
Congo − Brazzaville
Netherlands
Papua New Guinea
Finland
Hungary
Slovenia
Laos
Armenia
Serbia
Guatemala
Rwanda
Venezuela
Sri Lanka
Ethiopia
Thailand
Cuba
Timor−Leste
Honduras
Uzbekistan
Poland
Ireland
Montenegro
Uruguay
Austria
Peru
Chile
Belarus
Yemen
Brazil
Kazakhstan
Dominican Republic
Spain
Afghanistan
Trinidad & Tobago
Romania
Guyana
South Korea
Congo − Kinshasa
El Salvador
Portugal
Cambodia
Vietnam
India
Nicaragua
United States
Mexico
Norway
Colombia
Mozambique
Tajikistan
Macedonia
Bangladesh
Mauritius
Ecuador
Sierra Leone
North Korea
0
200
400
600
Percentage change in YLLs averted per 1000 vaccinated girls

Percentage change in DALYs averted per 1000 vaccinated girls
(vaccination age = 12 years / nonavalent vaccine)

Comparsion of scenario s4 in comparison to scenario s1

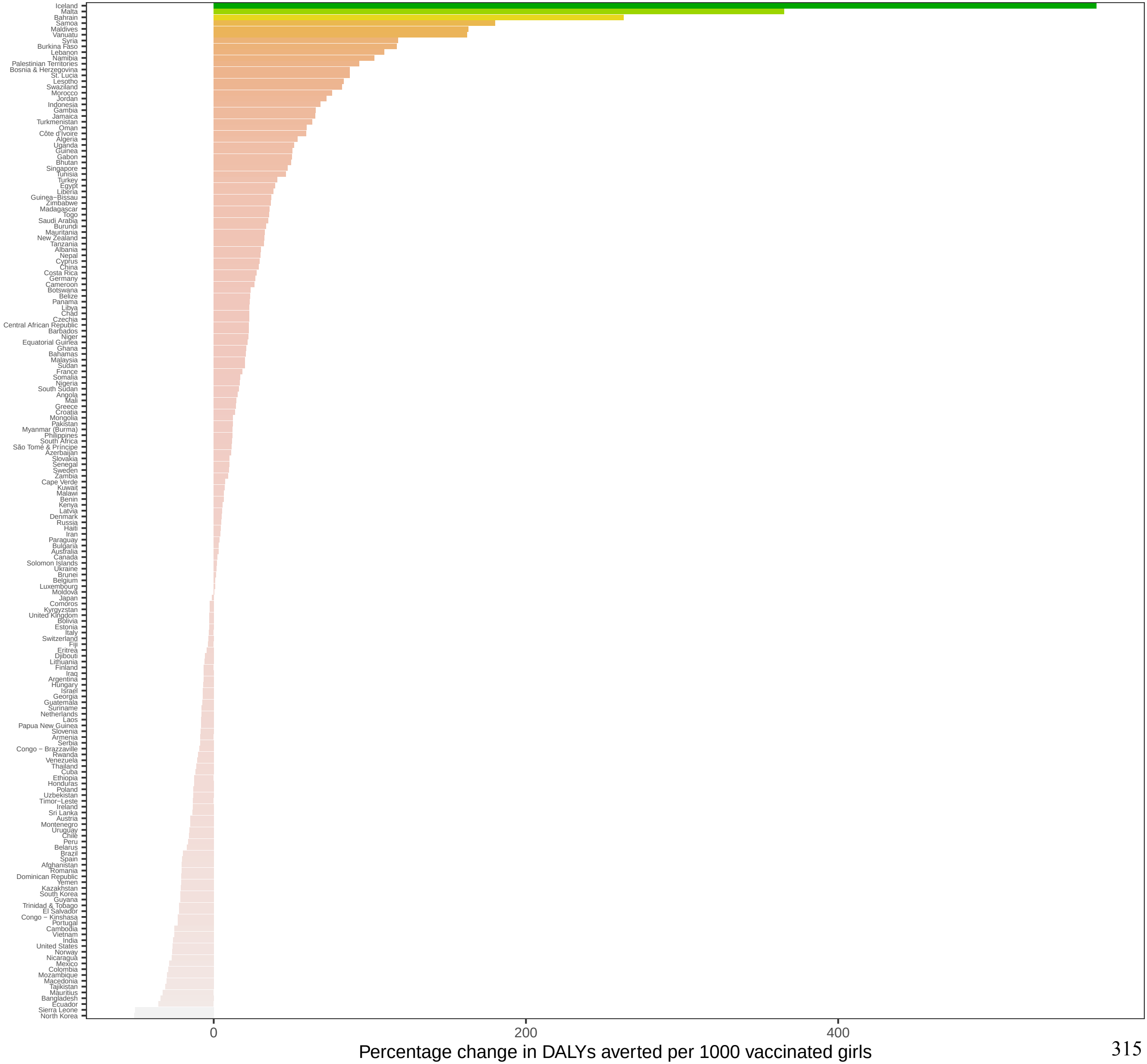

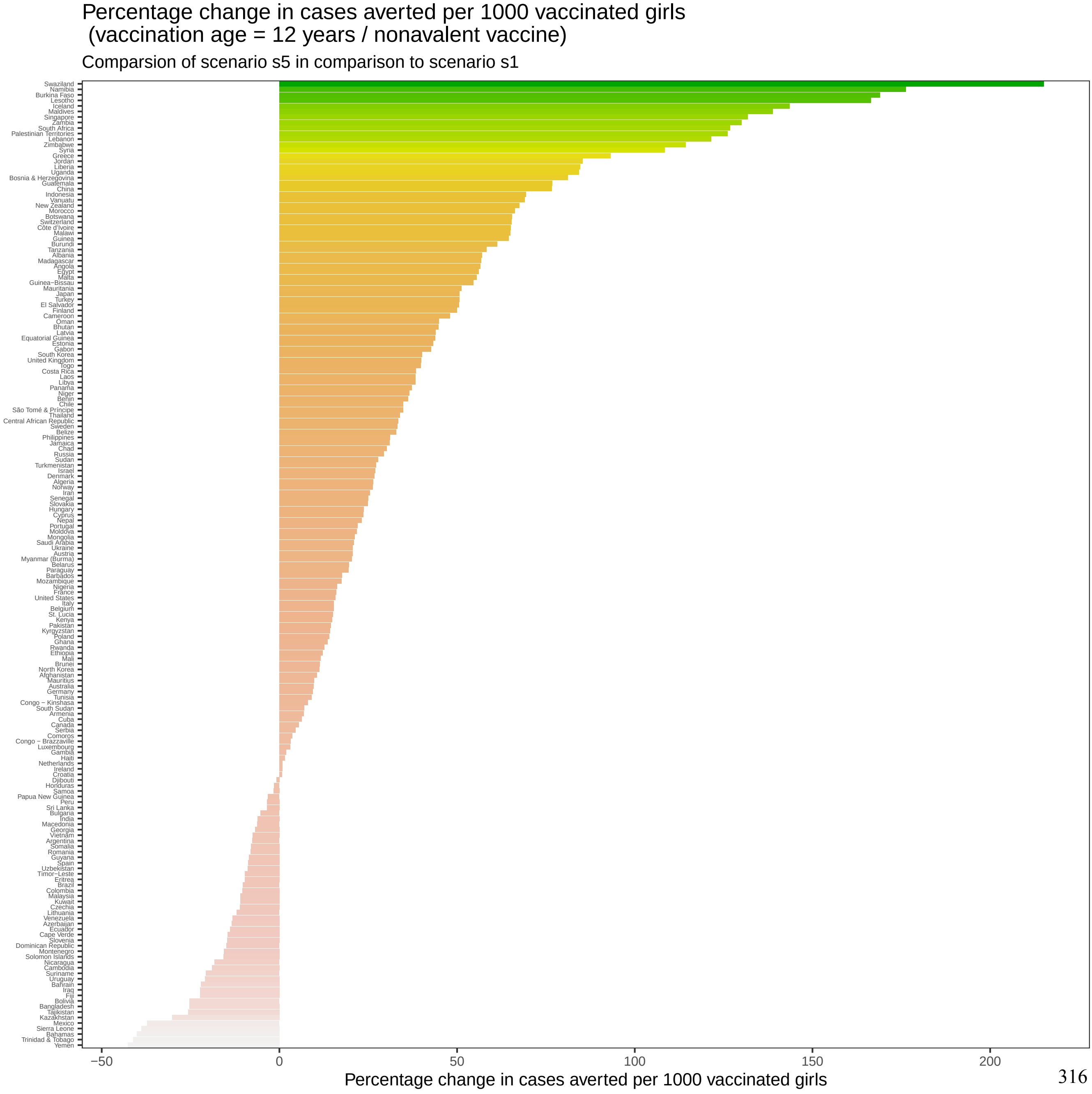
Percentage change in cases averted per 1000 vaccinated girls
(vaccination age = 12 years / nonavalent vaccine)
Comparsion of scenario s5 in comparison to scenario s1
Swaziland
Namibia
Burkina Faso
Lesotho
Iceland
Maldives
Singapore
Zambia
South Africa
Palestinian Territories
Lebanon
Zimbabwe
Syria
Greece
Jordan
Liberia
Uganda
Bosnia & Herzegovina
Guatemala
China
Indonesia
Vanuatu
New Zealand
Morocco
Botswana
Switzerland
Côte d'Ivoire
Malawi
Guinea
Burundi
Tanzania
Albania
Madagascar
Angola
Egypt
Malta
Guinea−Bissau
Mauritania
Japan
Turkey
El Salvador
Finland
Cameroon
Oman
Bhutan
Latvia
Equatorial Guinea
Estonia
Gabon
South Korea
United Kingdom
Togo
Costa Rica
Laos
Libya
Panama
Niger
Benin
Chile
São Tomé & Príncipe
Thailand
Central African Republic
Sweden
Belize
Philippines
Jamaica
Chad
Russia
Sudan
Turkmenistan
Israel
Denmark
Algeria
Norway
Iran
Senegal
Slovakia
Hungary
Cyprus
Nepal
Portugal
Moldova
Mongolia
Saudi Arabia
Ukraine
Austria
Myanmar (Burma)
Belarus
Paraguay
Barbados
Mozambique
Nigeria
France
United States
Italy
Belgium
St. Lucia
Kenya
Pakistan
Kyrgyzstan
Poland
Ghana
Rwanda
Ethiopia
Mali
Brunei
North Korea
Afghanistan
Mauritius
Australia
Germany
Tunisia
Congo − Kinshasa
South Sudan
Armenia
Cuba
Canada
Serbia
Comoros
Congo − Brazzaville
Luxembourg
Gambia
Haiti
Netherlands
Ireland
Croatia
Djibouti
Honduras
Samoa
Papua New Guinea
Peru
Sri Lanka
Bulgaria
India
Macedonia
Georgia
Vietnam
Argentina
Somalia
Romania
Guyana
Spain
Uzbekistan
Timor−Leste
Eritrea
Brazil
Colombia
Malaysia
Kuwait
Czechia
Lithuania
Venezuela
Azerbaijan
Ecuador
Cape Verde
Slovenia
Dominican Republic
Montenegro
Solomon Islands
Nicaragua
Cambodia
Suriname
Uruguay
Bahrain
Iraq
Fiji
Bolivia
Bangladesh
Tajikistan
Kazakhstan
Mexico
Sierra Leone
Bahamas
Trinidad & Tobago
Yemen
−50
0
50
100
150
200
Percentage change in cases averted per 1000 vaccinated girls

Percentage change in deaths averted per 1000 vaccinated girls (vaccination age = 12 years / nonavalent vaccine)

Comparsion of scenario s5 in comparison to scenario s1

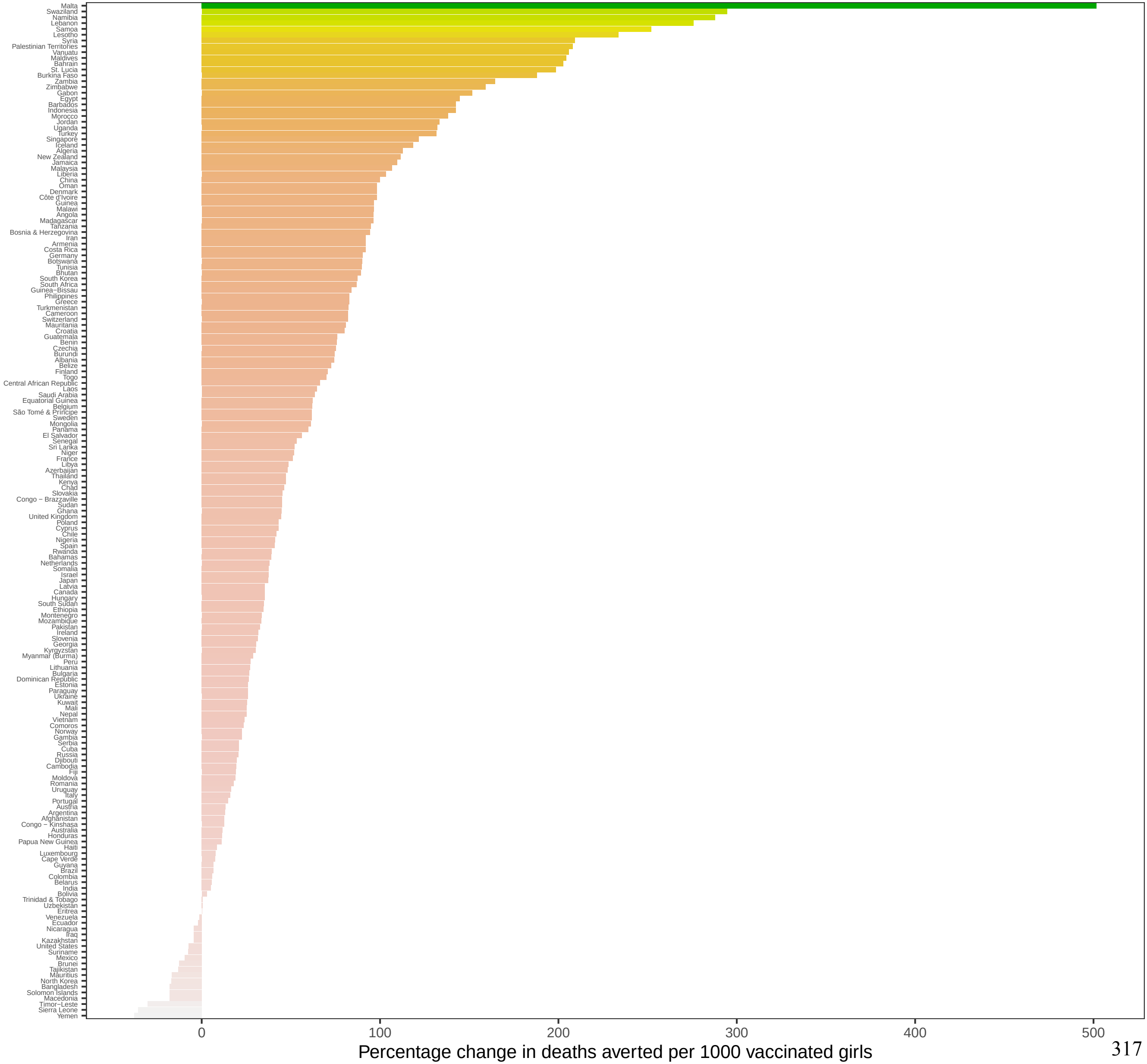

Percentage change in YLDs averted per 1000 vaccinated girls
(vaccination age = 12 years / nonavalent vaccine)

Comparsion of scenario s5 in comparison to scenario s1

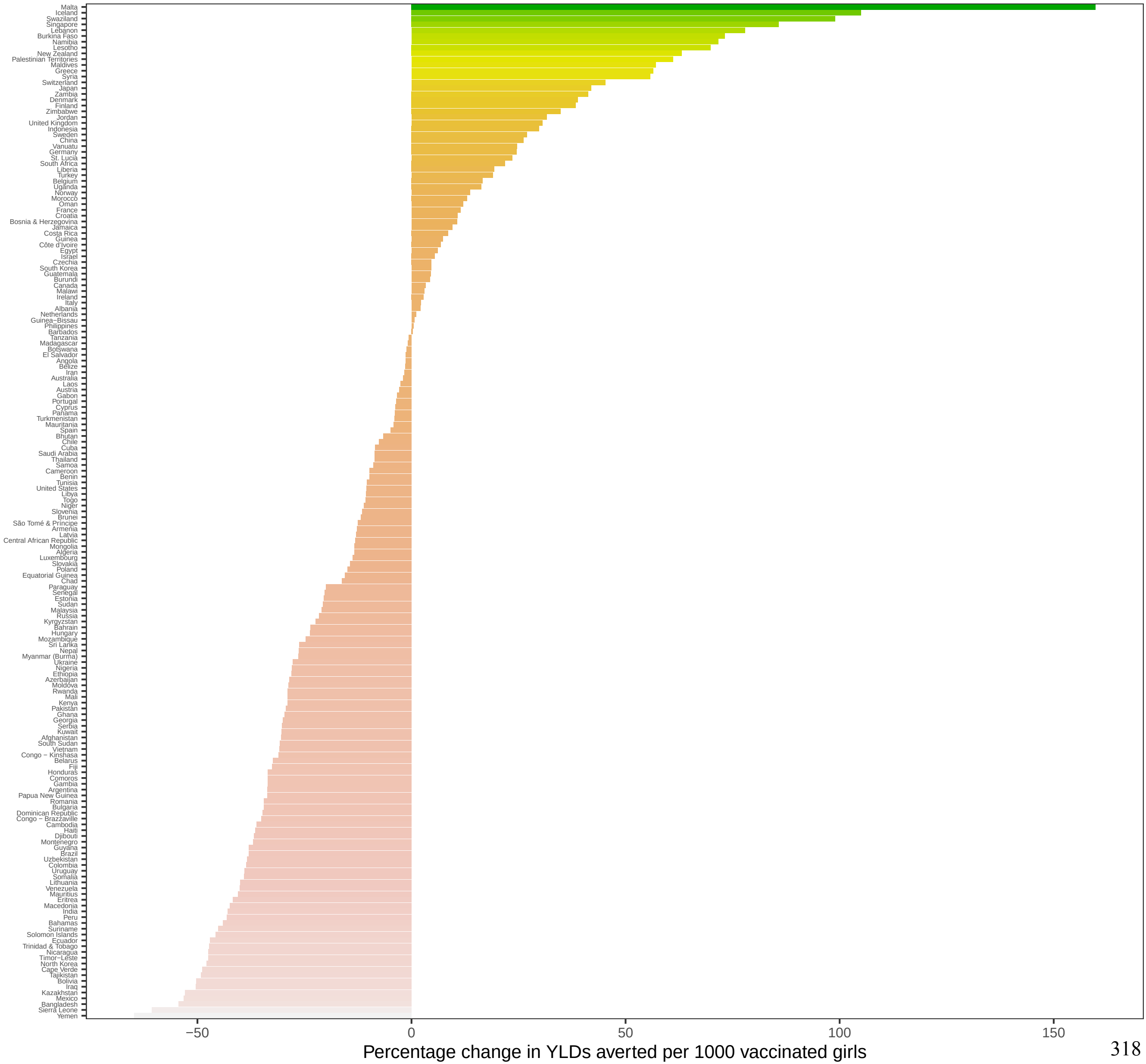

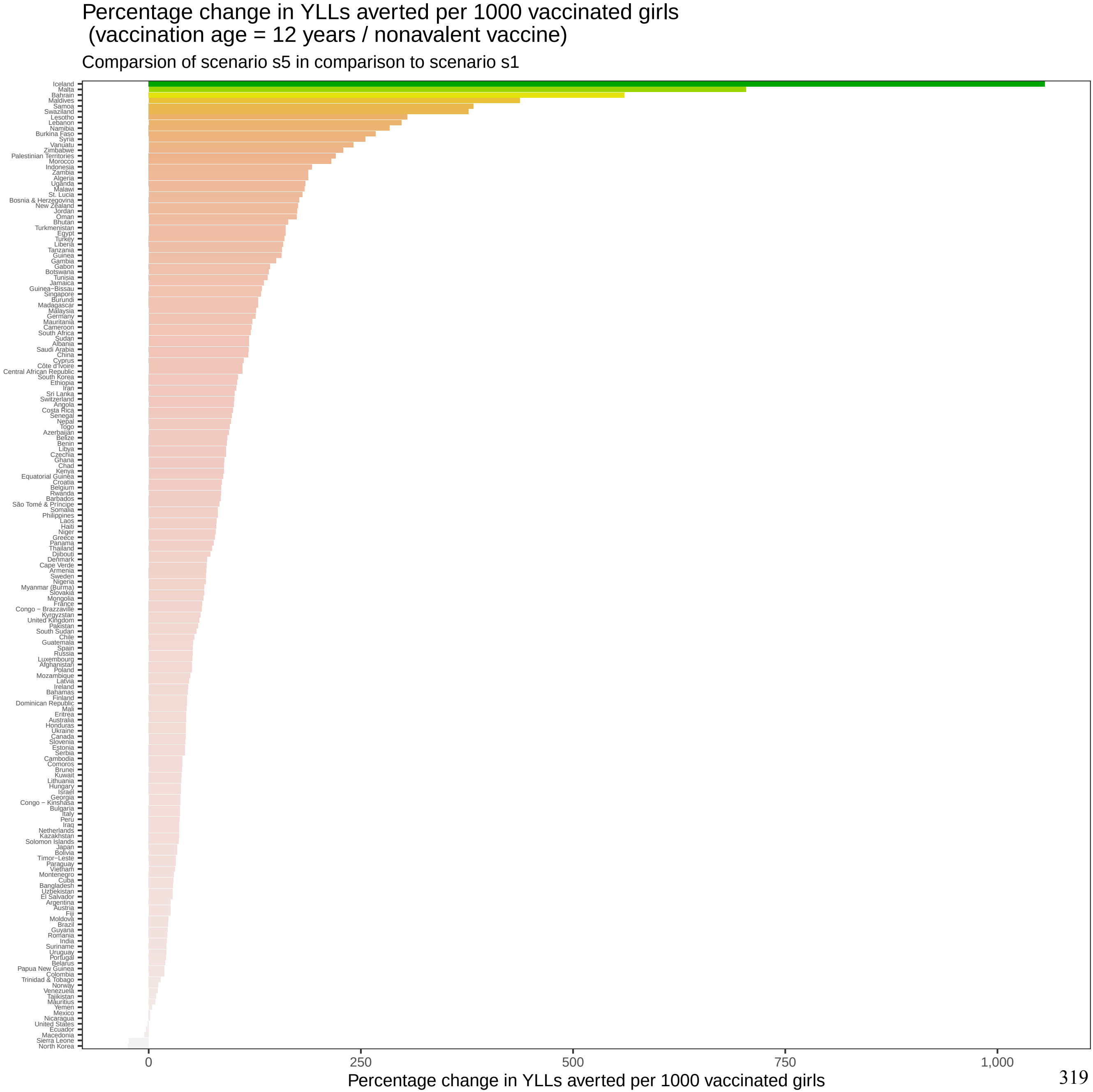

Percentage change in YLLs averted per 1000 vaccinated girls
(vaccination age = 12 years / nonavalent vaccine)
Comparsion of scenario s5 in comparison to scenario s1
Iceland
Malta
Bahrain
Maldives
Samoa
Swaziland
Lesotho
Lebanon
Namibia
Burkina Faso
Syria
Vanuatu
Zimbabwe
Palestinian Territories
Morocco
Indonesia
Zambia
Algeria
Uganda
Malawi
St. Lucia
Bosnia & Herzegovina
New Zealand
Jordan
Oman
Bhutan
Turkmenistan
Egypt
Turkey
Liberia
Tanzania
Guinea
Gambia
Gabon
Botswana
Tunisia
Jamaica
Guinea−Bissau
Singapore
Burundi
Madagascar
Malaysia
Germany
Mauritania
Cameroon
South Africa
Sudan
Albania
Saudi Arabia
China
Cyprus
Côte d'Ivoire
Central African Republic
South Korea
Ethiopia
Iran
Sri Lanka
Switzerland
Angola
Costa Rica
Senegal
Nepal
Togo
Azerbaijan
Belize
Benin
Libya
Czechia
Ghana
Chad
Kenya
Equatorial Guinea
Croatia
Belgium
Rwanda
Barbados
São Tomé & Príncipe
Somalia
Philippines
Laos
Haiti
Niger
Greece
Panama
Thailand
Djibouti
Denmark
Cape Verde
Armenia
Sweden
Nigeria
Myanmar (Burma)
Slovakia
Mongolia
France
Congo − Brazzaville
Kyrgyzstan
United Kingdom
Pakistan
South Sudan
Chile
Guatemala
Spain
Russia
Luxembourg
Afghanistan
Poland
Mozambique
Latvia
Ireland
Bahamas
Finland
Dominican Republic
Mali
Eritrea
Australia
Honduras
Ukraine
Canada
Slovenia
Estonia
Serbia
Cambodia
Comoros
Brunei
Kuwait
Lithuania
Hungary
Israel
Georgia
Congo − Kinshasa
Bulgaria
Italy
Peru
Iraq
Netherlands
Kazakhstan
Solomon Islands
Japan
Bolivia
Timor−Leste
Paraguay
Vietnam
Montenegro
Cuba
Bangladesh
Uzbekistan
El Salvador
Argentina
Austria
Fiji
Moldova
Brazil
Guyana
Romania
India
Suriname
Uruguay
Portugal
Belarus
Papua New Guinea
Colombia
Trinidad & Tobago
Norway
Venezuela
Tajikistan
Mauritius
Yemen
Mexico
Nicaragua
United States
Ecuador
Macedonia
Sierra Leone
North Korea
0
250
500
750
1,000
Percentage change in YLLs averted per 1000 vaccinated girls

Percentage change in DALYs averted per 1000 vaccinated girls
(vaccination age = 12 years / nonavalent vaccine)

Comparsion of scenario s5 in comparison to scenario s1

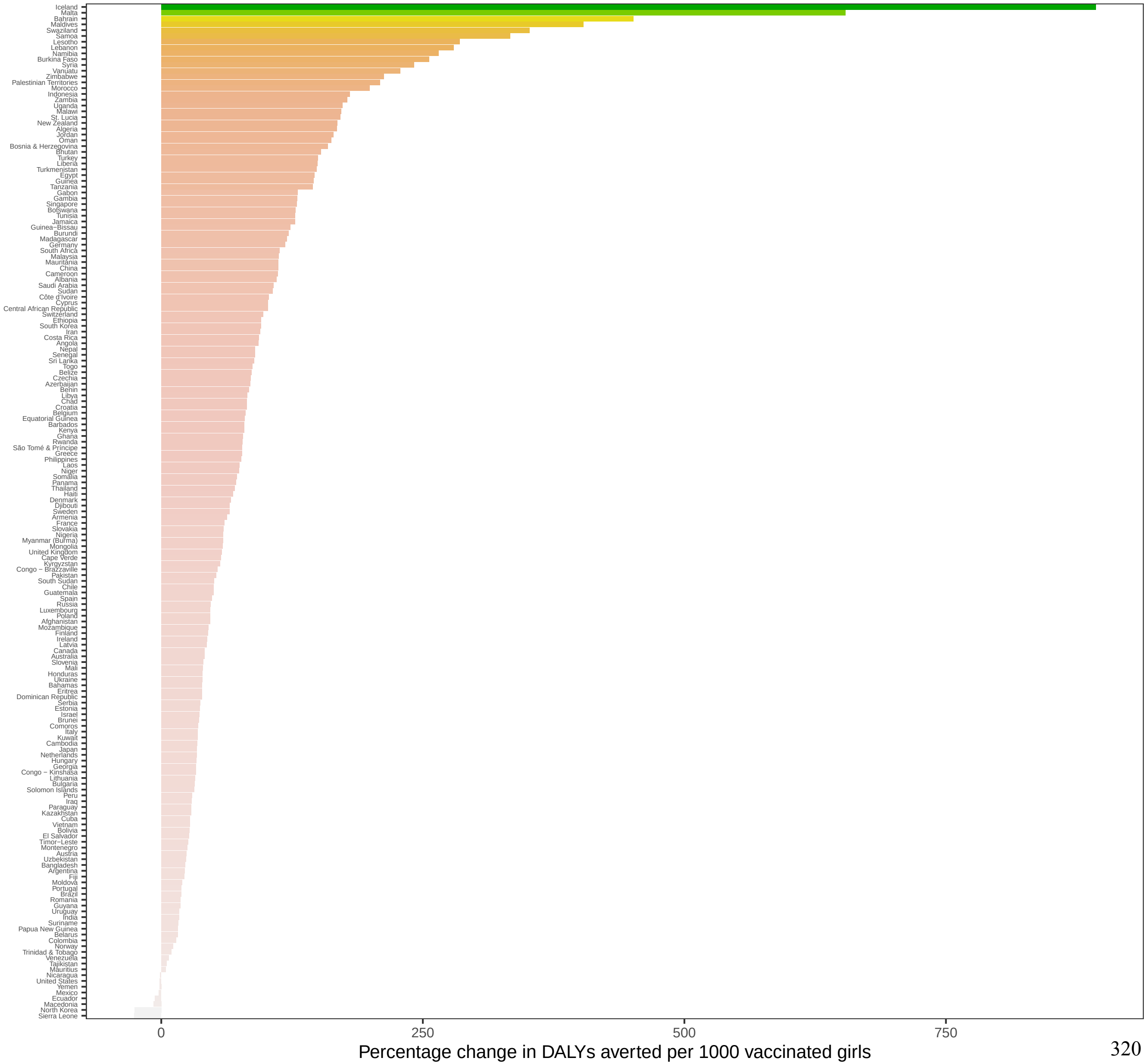

Supplement: Supplementary appendix 1 [file mmc1.pdf]
